# Satellites in the prokaryote world

**DOI:** 10.1186/s12862-019-1504-2

**Published:** 2019-09-18

**Authors:** Juan A. Subirana, Xavier Messeguer

**Affiliations:** Department of Computer Science, Universitat Politècnica de Catalunya, Jordi Girona 1-3, 08034 Barcelona, Spain

**Keywords:** Satellites, Tandem repeats, *Bacteria*, *Archaea*, *Methanosarcina*, *Leptospira*, *Methanocella*

## Abstract

**Background:**

Satellites or tandem repeats are very abundant in many eukaryotic genomes. Occasionally they have been reported to be present in some prokaryotes, but to our knowledge there is no general comparative study on their occurrence. For this reason we present here an overview of the distribution and properties of satellites in a set of representative species. Our results provide novel insights into the evolutionary relationship between eukaryotes, *Archaea* and *Bacteria*.

**Results:**

We have searched all possible satellites present in the NCBI reference group of genomes in *Archaea* (142 species) and in *Bacteria* (119 species), detecting 2735 satellites in *Archaea* and 1067 in *Bacteria*. We have found that the distribution of satellites is very variable in different organisms. The archaeal *Methanosarcina* class stands out for the large amount of satellites in their genomes. Satellites from a few species have similar characteristics to those in eukaryotes, but most species have very few satellites: only 21 species in *Archaea* and 18 in *Bacteria* have more than 4 satellites/Mb. The distribution of satellites in these species is reminiscent of what is found in eukaryotes, but we find two significant differences: most satellites have a short length and many of them correspond to segments of genes coding for amino acid repeats. Transposition of non-coding satellites throughout the genome occurs rarely: only in the bacteria *Leptospira interrogans* and the archaea *Methanocella conradii* we have detected satellite families of transposed satellites with long repeats.

**Conclusions:**

Our results demonstrate that the presence of satellites in the genome is not an exclusive feature of eukaryotes. We have described a few prokaryotes which do contain satellites. We present a discussion on their eventual evolutionary significance.

**Electronic supplementary material:**

The online version of this article (10.1186/s12862-019-1504-2) contains supplementary material, which is available to authorized users.

## Background

Satellites are tandem repeat sequences present in many eukaryotic genomes. The evolution and biological roles of satellites in different species has attracted much attention [1, 2]. Prokaryotes have a very dense genome and are not expected to have satellites in intergenic regions, but satellites have indeed been described in several species [3–5]. From these studies it appears that the expansion and transposition of satellites may have a strong influence in the adaptation of microorganisms to different environments, in particular when satellites are part of protein coding genes. A systematic study should provide a general view on the significance of satellites in the prokaryote world. Their properties are very different from the abundant insertion sequences which have been studied in detail in many prokaryotic species [6].

Here we analyze the distribution of satellites in a reference list of prokaryotic species. We define satellites as long tandem repeats with at least four repeats, with each repeat having a length of 10–200 nucleotides. Some of the shorter satellites are often defined as minisatellites [2], but we have not attempted in this paper to define them as a separate class. We also require that the satellites have an internal regularity, as explained in the methods section. No limit is placed on the total length of individual satellites. The number and size distribution of satellites we find is very different from that observed in several eukaryotic species we have previously studied [7]. Our results provide new clues on the evolutionary relationship between eukaryotes, *Archaea* and *Bacteria*.

## Results

### Satellites. General features

We have searched all satellites in 142 archaeal and 119 bacterial species for which a complete genome sequence is available, as listed in Additional file 1. We have identified 2635 satellites in *Archaea* and 1067 in *Bacteria*. The difference between the two groups is mainly due to the *Methanosarcina*, which have 1908 satellites. The number of satellites in each species is very variable; many have a negligible number of satellites: only 40% of the *Archaea* species and 56.3% of the *Bacteria* have more than three satellites in their genome. The sequence and characteristics of all satellites is given in fasta format in Additional file 2. A simplified list in excel format is presented in Additional file 3. Most satellites are rather short, only 28 satellites in *Archaea* (1.1%) and 32 in *Bacteria* (2.7%) are longer than 1.5 kb; they are listed in Additional file 4. This feature clearly distinguishes the microbial satellites from those found in eukaryotes, which have a large proportion of long satellites [7]. For example in the eukaryote *Caenorhabditis elegans* we have found 10.3% of its satellites to be longer than 1.5 kb. Occasionally we have localized a few satellites, with a repeat length around 65 nt, which contain Clustered Regularly Interspaced Short Palindromic Repeats (CRISPR motifs). However, to be considered as a satellite, it is required that the spacer between CRISPR motifs has a constant length (+/− 1 nt), a rare event. For this reason CRISPR regions seldom appear among the satellites we have found.

**Fig. 1:**
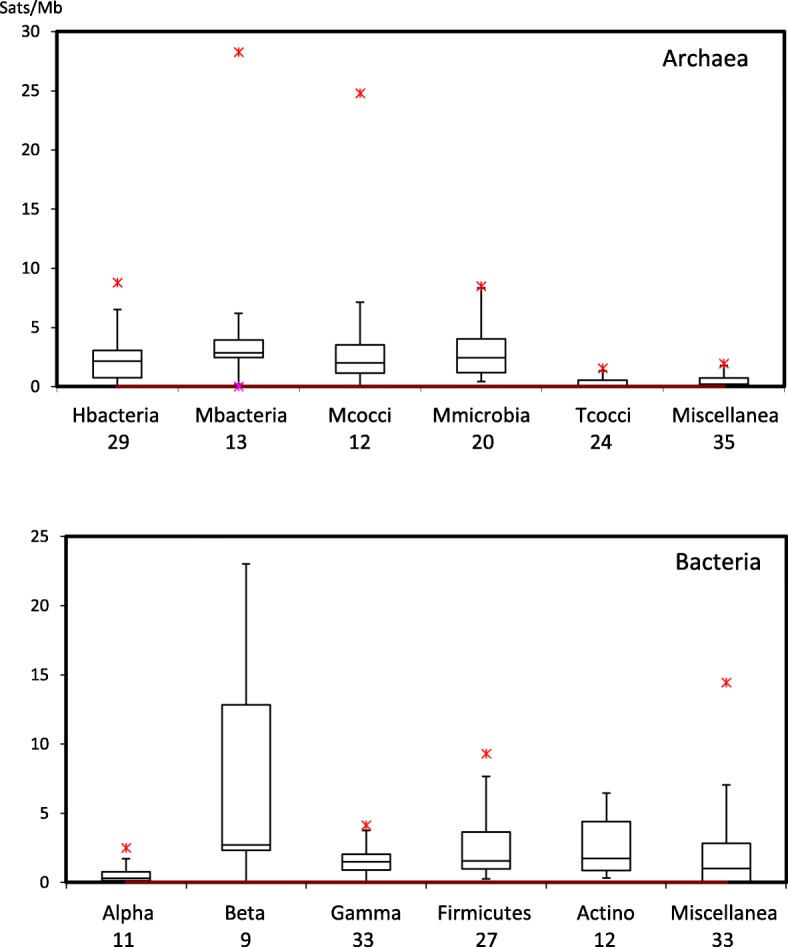
Box-plots showing the distribution of satellite densities (Satellites/Mb) in the genomes of different prokaryotic groups. The numbers below the name indicate the number of species in each group. Data for *Methanosarcina* are not included. The miscellanea category in *Archaea* includes several groups of species with a small number of satellites (0–3 Satellites/Mb); in *Bacteria* we have merged all groups with a small number of species in the reference NCBI list. Median values in all cases are in the range 0–3 satellites/Mb. Detailed data for all species are available in Additional file 1

As mentioned in the introduction, there is a large difference between individual species in the number of satellites, as it is also apparent in Additional file 1. Clear differences are also found between different groups*,* as shown in Fig. 1. This figure has to be interpreted with caution, since the number of species studied is small and varies in different groups. Nevertheless one feature stands up clearly: the low occurrence of satellites in several groups. In Additional file 5 we present an overall view of the distribution of satellites in some groups of *Archaea* and in *Bacteria*. In all cases most satellites with long repeats have values which are multiples of 3 nt, which suggests that these satellites are likely coding for amino acid repeats in proteins.

### Microsatellites and satellites with short repeats

**Fig. 2:**
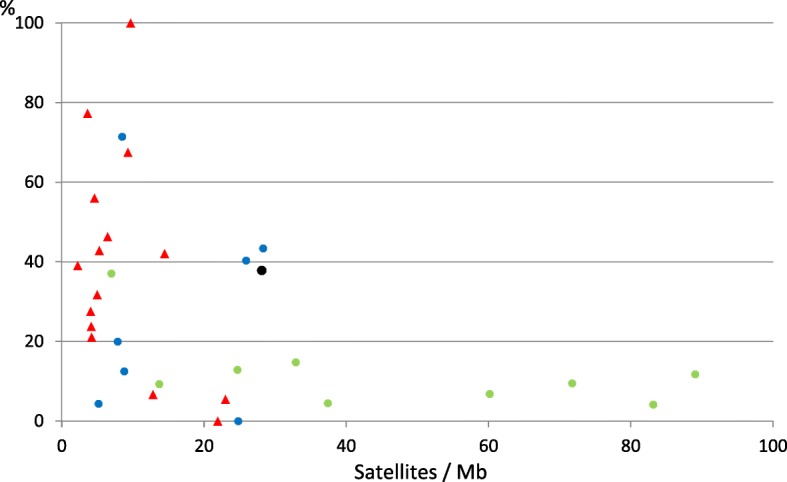
Percentage of satellites with long repeats (over 30 nt) for all eukaryotic species which have more than 20 satellites. A list with details for all these species is given in Table 1. *Bacteria* are shown as red triangles, *Methanosarcina* as green dots and all other *Archaea* as blue dots. The value for the nematode *Caenorhabditis elegans* is represented as a black dot

Microsatellites with short repeats (3–6 nt) are abundant in all the species we have studied. They may be retrieved from the microsatellite database [8]. The satellites we have found with short repeats (10–30 nt) are often related to microsatellites which have undergone local mutations. In Table 1 we present the relative proportion of this class of satellites for all the species which have more than 20 satellites in their genome. They are also shown in Fig. 2.

**Table 1:** Prokaryotic species with a large number of satellites (> 20)

Species	Class	NCBI code	Size (Mb)	% CG	Nr sats	Sats/Mb	>30 (%)
Archaea							
Methanosarcina vacuolata	Methanomicrobia	NZ_CP009520	4.56	39.7	406	89	11.8
Methanosarcina barkeri	Methanomicrobia	NZ_CP009517	4.56	39.1	379	83.1	4.2
Methanosarcina barkeri	Methanomicrobia	NZ_CP009528	4.57	39.2	328	71.7	9.5
Methanosarcina lacustris	Methanomicrobia	NZ_CP009515	4.14	41.8	249	60.1	6.83
Methanosarcina acetivorans	Methanomicrobia	NC_003552.1	5.75	42.7	189	32.9	14.8
Methanosarcina mazei	Methanomicrobia	NZ_CP009512	4.14	41.4	155	37.4	4.52
Methanosarcina siciliae	Methanomicrobia	NZ_CP009506	5.02	42.9	124	24.7	12.9
Methanosarcina thermophila	Methanomicrobia	NZ_CP009501	3.13	41.1	43	13.7	9.3
Methanosarcina horonobensis	Methanomicrobia	NZ_CP009516	5.02	41.3	35	6.97	37.1
Methanobrevibacter ruminantium	Methanobacteria	NC_013790.1	2.94	32.6	83	28.3	43.4
Methanobrevibacter olleyae	Methanobacteria	NZ_CP014265	2.20	26.9	57	25.9	40.3
Methanococcus voltae	Methanococci	NC_014222.1	1.94	28.6	48	24.8	0
Halorubrum lacusprofundi	Halobacteria	NC_012029.1	2.74	63.9	24	8.77	12.5
Natrialba magadii	Halobacteria	NC_013922.1	4.44	61.0	23	5.18	4.35
Methanoculleus marisnigri	Methanomicrobia	NC_009051.1	2.48	62.1	21	8.47	71.4
Methanobacterium paludis	Methanobacteria	NC_015574.1	2.55	35.7	20	7.85	20
Bacteria							
Chloroflexus aurantiacus	Chloroflexi	NC_010175.1	5.26	56.7	76	14.4	42.1
Burkholderia pseudomallei chrII	Betaproteobacteria	NC_006351.1	3.17	68.1	73	23.0	5.5
Burkholderia mallei chrII	Betaproteobacteria	NC_006349.1	2.33	68.5	51	21.9	0
Burkholderia mallei chrI	Betaproteobacteria	NC_006348.1	3.51	68.5	45	12.8	6.7
Leptospira interrogans chrI	Spirochaetia	NC_004342.2	4.34	35	42	9.68	100
Clostridioides difficile	Firmicutes	NC_009089.1	4.30	29.1	40	9.31	67.5
Streptomyces coelicolor	Actinobacteria	NC_003888.3	9.05	72	38	4.20	21.1
Rhodopirellula baltica	Planctomycetes	NC_005027.1	7.15	55.4	29	4.06	27.6
Mycobacterium bovis	Actinobacteria	NC_002945.4	4.35	65.6	28	6.44	46.4
Bacillus thuringiensis	Firmicutes	NC_005957.1	5.31	35.4	28	5.27	42.9
Bacillus cereus	Firmicutes	NC_004722.1	5.43	35.3	25	4.61	56
Amycolatopsis mediterranei	Actinobacteria	NC_014318.1	10.2	71.3	23	2.25	39.1
Pseudomonas syringae	Gammaproteobacteria	NC_007005.1	6.09	59.2	22	3.61	77.3
Mycobacterium tuberculosis	Actinobacteria	NC_000962.3	4.41	65.6	22	4.99	31.8
Xanthomonas campestris	Gammaproteobacteria	NC_003902.1	5.08	65.1	21	4.14	23.8

Satellites with a related sequence can be grouped into families, as described in the methods section. For example, the most abundant family with a short repeat in *Archaea* (Fam_2_12_13) appears to be derived from an (ATT)_n_ microsatellite. *Methanococcus voltae* is the main contributor to this family. Several additional families and single satellites, also AT-rich, are typical of this species (Additional file 6). It is likely that these satellites have appeared locally in the genome as errors of DNA duplication. The same is true for *Bacteria*, where some of the most abundant satellite families appear to be derived from heptamer and hexamer repeats. An example is Fam_3_12_12, derived from the hexamer repeat CAACAG and related sequences, found in several species (Additional file 6). An additional group of satellites with short repeats can be considered as typical, eukaryote-like, satellites, which have been probably transposed throughout the genome. In general they are species-specific. A very clear case is Fam_1_14_23; all of its satellites belong to a single bacterial species, *Chloroflexus aurantiacus,* and are scattered throughout its genome (Additional file 6). Finally a third class of satellites with short repeats may correspond to portions of genes with amino acid repeats, which are characterized by a repeat length multiple of three. This appears to be the predominant case in *Halobacteria*, as it is apparent in (Additional file 5: Figure S1).

### Satellites in selected *Archaea*

As an example of the heterogeneity in the distribution of satellites in Archaea we present in Table 1 the main results obtained for several species, selected for their higher content of satellites. We find a large difference in the relative amount of satellites with a long repeat (> 30 nt), as it is clearly apparent in Fig. 2. *Methanococcus voltae* stands out by the total absence of satellites with long repeats. All its short satellites are very AT-rich, mostly in the range 90–100% AT. However other *Methanococci* do contain a few satellites with longer repeats (Additional file 3). These short repeats may be considered as derived from microsatellites. The same is true for *Halorubrum lacusprofundi*, which has only three short isolated satellites with a repeat longer than 30 nt. At the other extreme, *Methanobrevibacter ruminantium* has a large relative amount of satellites with a repeat over 30 nt (43.4%). Other *Methanobrevibacter* species have similar features, as it is shown in Additional file 3. All the 36 satellites with long repeats in *M. ruminantium* are unrelated among themselves, they have unique sequences and are scattered throughout the genome. At least half of them are non-coding, as judged from the length of their repeat units. It appears that this important rumen methanogen has a large potential to grow satellites throughout the genome, a feature which has not been previously described [9].

### Satellites in *Methanosarcina*

The *Methanosarcina* are particularly rich in satellites among the *Archaea*, containing together a total of 1908 satellites in the nine species studied. About 5% of the genes in this group have been horizontally transferred from bacteria [10]. This genus presents a high metabolic diversity; they can grow by reduction of CO_2_, reduction of methyl groups, acetate fermentation, etc. [5]. They may also occupy multiple environments, ranging from all kinds of sediments to the rumen of ungulates. This diversity is facilitated by multiple gene rearrangements, as it is apparent in the analysis of several genomes in this genus [5, 11–14]. Their relation with other methanogenic archaea has also been analyzed, demonstrating large changes in ploidy, even in the same species depending on the growth conditions [15]. In Table 1 we present the data for all these species, which show a variable number of satellites, ranging from 35 to 406. This variability is also apparent in Fig. 2, where it is also clear that *Methanosarcina* have a low proportion of satellites with long repeats.

**Fig. 3:**
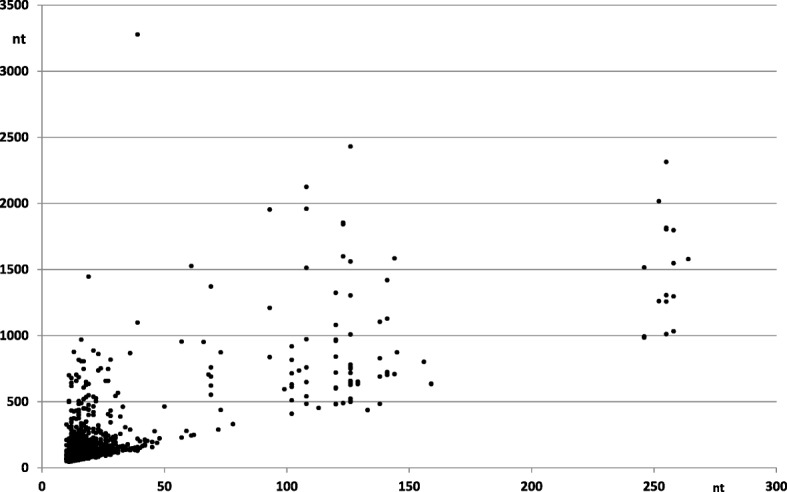
Satellite length as a function of repeat size in *Methanosarcina*. All 1908 satellites found are represented. Fragments of genes coding for three types of amino acid repeats in proteins are found in the groups around 69, 100–150 and 260 repeat size, as discussed in the text. A large number of satellites related to microsatellites are apparent near the origin of the plot

In Fig. 3 we show a plot of satellite length vs repeat length for each of the 1908 satellites in the *Methanosarcina*: there are four groups of satellites. The most numerous group corresponds to satellites with short repeat units (below 30 nt), as it is also shown in (Additional file 5: Figure S1). Satellites with longer repeat units usually have a length which is a multiple of three; this suggests that these satellites correspond to amino acid repeats in genes coding for proteins. In fact most of the satellites with repeat size around 69, 100–150 and 246–264 (Fig. 3) correspond to domains in several proteins, which have been studied in detail in several *Methanosarcina* species [4, 5]. These proteins are mainly involved in the formation of surface layers and have a structure closely related to some eukaryotic protein domains [16].

### Satellites in *Bacteria*

In Fig. 1 we have presented all the bacterial species we have studied; they do not differ much from the values found in some archaeal groups, but closer inspection shows that the percentage of satellites with long repeats (> 30 nt) is significantly larger in *Bacteria* (42.6%) than in *Archaea* (17.5%). This is also clear in Fig. 2. The global distribution of repeat lengths in all satellites is presented in (Additional file 5: Figure S2). Repeats with a length multiple of three predominate, but a substantial number of repeats with other lengths are also apparent. We have selected the species with a higher amount of satellites/Mb for a more detailed analysis. Their properties are summarized in Table 1 and we will discuss them next.

We will start with *Leptospira interrogans*, which is unique among all the species we have studied by the total absence of satellites with short repeats. Its genome was sequenced a few years ago [17] and found to have two chromosomes. The smallest one has no satellite. In the large chromosome I we have found 42 satellites, one third of them are found in genes which code for proteins. The other two thirds are intergenic; most of them belong to satellite families which we will discuss in the next section. Their satellites are scattered throughout the genome and demonstrate that a process of non-coding satellite transposition has taken place in this species. *Clostridium difficile* belongs to a different group of *Bacteria*, but it also has a large number of satellites with long repeats. However their nature is completely different: all of them are unique and unrelated to other satellites. A peculiarity of its genome is that most satellites are clustered in two regions, in positions 677,135–853,804 and 3,631,557–3,800,012. In between there are a few scattered satellites and a 2 Mb region with no satellite. In this species we have detected five satellites with a repeat length 66–67 nt which are CRISPR repeats.

At the other end of satellite distribution in *Bacteria* we find *Burkholderia mallei* and *pseudomallei*. Their genome is organized in two chromosomes with different sizes and a large difference in the distribution of satellites. These two species have over 90% of its satellites with short repeat lengths, as it is clearly apparent in Fig. 2. These satellites have repeat lengths of 12, 14 and 16 nt: they derive from CG-rich microsatellite repeats with unit size 6–8 nt. These satellites form several families, each with a different repeat sequence (Additional file 5). They are only a fraction of the large number of microsatellites in these species [3], which are probably responsible for the genome rearrangements of these two species and their different strains [18]. *Chloroflexus aurantiacus* is an unrelated species, but it also has 22 satellites derived from a single heptamer repeat (GATAGRA)_n_. They are the main partners of the largest family of satellites found in *Bacteria* (Fam_1_14_23). This species differs from *B. mallei* and *B. pseudomallei* by the presence of a substantial number of satellites with long repeats.

The other species included in Table 1 have variable features, intermediate between those described above, as it is apparent in Fig. 2. Most satellite families contain satellites from several unrelated species, with the exception of *Bacillus,* which have satellites common to species of the same genus. *Mycobacteria* stand out by having a large proportion of satellites found in genes coding for amino acid repeats.

### Satellite families

Very often satellites have developed spontaneously and have a unique sequence unrelated to any other satellite. This is the case for 40.1% of the bacterial and 39.9% of the archaeal satellites. The rest of them can be grouped into families with a similar repeat. The presence of satellites with a similar repeat usually indicates a common origin: in different species it may correspond to related genomic regions, in the same species it indicates the presence of transposition. A list of all satellite families with the sequence of each individual satellite is given in Additional file 6. A summary of all families is also given in Additional file 7. We also searched for joint families of *Archaea* and *Bacteria*, but did not find any common family.

A large group of families are derived from short repeats, related to microsatellites. Most of these families contain satellites from several unrelated species, but some families are restricted to one or a few closely related species, as it is the case for some bacteria, such as *Burkholderia* or *C. aurantiacus* (Fam _1_14_23). *Mesorhizobium ciceri* also has its own family, Fam_19_16_7, derived from the octamer dimer (AGGGGAGT)_2_. In the case of *Archaea* the most abundant families with short repeats are found in *Methanococcus* and in *Methanosarcina*. The conservation of sequence of satellites derived from short repeats throughout the genome suggests the presence of a possible transposition mechanism, which might indicate a function for these sequences, as discussed above for *Burkholderia.*

A second group of families includes those with a repeat size over 30 nt. A list of all of them is presented in Table 2. It is apparent that most of these families correspond to parts of genes which code for proteins with amino acid repeats. For example, the largest family in *Archaea*, Fam_1_126_16, corresponds to proteins of *Methanosarcina* containing peptides related to a reference sequence of 34 amino acids (tetratrico repeats).

**Table 2:** Main satellite families with long repeat lengths

Archaea		Bacteria	
Family	Genus or species	Family	Genus or species
Fam_1_126_16	*Methanosarcina*	Fam_13_96_8	Several species
Fam_15_246_6	*Methanosarcina barkeri*	Fam_14_39_8	Several species
Fam_16_141_6	*Methanosarcina*	Fam_17_100_7	*Escherichia coli*
Fam_17_78_6	*Methanobrevibacter olleyae*	Fam_23_69_6	*Flavobacterium psychrophilum*
Fam_23_156_5	*Methanothrix soehngenii*	Fam_24_46_6	*Leptospira interrogans*
Fam_24_141_5	*Methanosphaerula palustris*	Fam_25_46_6	*Leptospira interrogans*
Fam_25_120_5	*Methanosarcina*	Fam_26_37_6	*Leptospira interrogans*
Fam_26_108_5	*Methanosarcina*	Fam_31_69_5	*Leptospira interrogans*
Fam_27_37_5	*Methanocella conradii*	Fam_32_45_5	*Streptococcus*
Fam_44_255_4	*Methanosarcina*	Fam_33_30_5	*Mycobacterium*
Fam_45_123_4	*Methanosarcina*	Fam_42_93_4	*Escherichia coli*
Fam_46_102_4	*Methanothermobacter*	Fam_43_39_4	*Bacillus*
Fam_47_69_4	*Methanosarcina*	Fam_44_36_4	*Bacillus*
Fam_48_45_4	*Methanoculleus marisnigri*	Fam_45_36_4	*Bacillus*
Fam_95_258_3	*Methanosarcina*	Fam_46_36_4	*Bacillus*
Fam_96_138_3	*Methanosarcina*	Fam_47_33_4	*Chloroflexus aurantiacus*
Fam_97_126_3	*Methanosarcina*	Fam_56_150_3	*Pseudomonas syringae*
Fam_98_120_3	*Methanosarcina*	Fam_57_156_3	*Dictyoglomus turgidum*
Fam_99_120_3	*Methanosarcina*	Fam_58_114_3	*Pseudomonas syringae*
Fam100_102_3	*Methanosarcina*	Fam_59_108_3	*Pseudomonas syringae*
Fam_101_93_3	*Methanosarcina vacuolata*	Fam_60_60_3	*Streptococcus*
Fam_102_51_3	*Methanoculleus marisnigri*	Fam_61_56_3	*Mycobacterium*
Fam_103_42_3	*Methanospirillum hungatei*	Fam_62_46_3	*Leptospira interrogans*
		Fam_63_48_3	Several species
		Fam_64_45_3	*Bacillus*
		Fam_65_42_3	*Bacillus*
		Fam_66_39_3	*Bacillus*
		Fam_67_39_3	*Bacillus*
		Fam_68_36_3	*Bacillus*

Satellite families are designed by a code of three numbers. The first number in the code indicates the order of this family, as measured by the number of satellites in the family. The second number corresponds to the repeat length of the family. The third number indicates the number of satellites included in the family. When the family belongs to a single species, its name is indicated. Note that most families have a repeat which is a multiple of 3 nt, with the notable exceptions of *L. interrogans* and *M. conradii.* In this list are only included those families with at least 3 satellites and a repeat length over 30 nt. A complete list of all families is given in Additional file 7

In a few cases we have found families formed by non-coding satellites with long repeats transposed throughout the genome, as found in eukaryotes like nematodes [7]. Among the bacteria, *Leptospira interrogans* stands out by having several families which are clearly non-coding. Among the *Archaea* the only exception is Fam_27_37_5, with five perfect repeats (score = 1), found in *Methanocella conradii*.

## Discussion

The birth of satellites results from intrinsic anomalies of DNA replication, but our survey shows that only a few prokaryotic species have a significant number of satellites. Many of them are unique and appear only once; no special significance may be attributed to them. When satellites are found and form families with a related sequence, maintained in the compact prokaryotic genomes, it may indicate an important advantage for the species.

Different patterns of satellite distribution are found, depending on the predominant families in each species (Table 2). The distribution of repeat sizes presented in Fig. 2 also demonstrates significant differences for each species. A common pattern found in many cases is due to satellites which are part of genes coding for amino acid repeats in proteins. In *Methanosarcina* they belong to different structural protein domains which have been involved in the development of a diversity of proteins in this genus. These proteins have allowed a remarkable evolution in the metabolism of these species, which are capable of growing in very different environments [5, 12–14]. They are a clear example of functional innovation through different combinations of protein modules [19, 20].

An extreme pattern we have detected is the abundance of satellites with short repeats, accompanied by an absence of satellites with long repeats. The *Burkholderi*a are clear examples (Fig. 2), their genome has numerous satellites with short repeats, derived from microsatellites with 6, 7 and 8 nt repeats. These repeats may facilitate the frequent genome rearrangements found in different strains of these species, which contribute to differences in virulence.

An alternative pattern is found in two species which have families of related satellites with no coding function, spread throughout the genome. This is the common situation in most eukaryotes [7], but rare in the prokaryotes we have studied. A clear example among the bacteria is the spirochete *L. interrogans*, with several families of non-coding satellites (Table 2). An additional intriguing feature of this species is the complete absence of satellites with short repeats (Fig. 2). Its genome surpasses those of other bacteria in terms of the number of proteins with structural similarity to eukaryotic and archaeal proteins that it encodes [17]. A similar situation is found in the archaea *M. conradii*. The spread of satellites throughout the genome of these species suggests a specific mechanism of satellite transposition. These two species are unique among the species we have studied, but it is possible that other species not included in our study may show similar features.

In summary, we have found a significant variability between different species. Specific patterns of satellites have emerged in some individual species. This scenario highlights the genome plasticity of prokaryotes, where exchange of genome sequences is widespread [21, 22]. The emergence of a few species with non-coding satellite families suggests that these species have acquired a specific mechanism of satellite transposition. The presence of these satellites indicates that they provide some specific advantage, but it is not obvious in what sense. It is possible that the biomolecules required to develop a successful mechanism of transposition of non-coding satellites have appeared as a result of a unique event of eukaryote-prokaryote horizontal genome transfer. In any case the study of non-coding regions of the genome may help to shed some light on the relation between the three domains of life [23], including the hypothetical origin of eukaryotes from a particular archaeal group [24–26].

## Conclusions

* Most species of prokaryotes have a low number of satellites. Only 21 species (14.8%) in *Archaea* (mostly *Methanomicrobia*) and 18 in *Bacteria* (15.1%) have more than 4 satellites / Mb. The distribution of satellites in these species is reminiscent to what is found in eukaryotes.

* We have studied 142 *Archaea* and 119 *Bacteria* genomes, detecting 2635 satellites in *Archaea* and 1067 in *Bacteria*. The satellites have a rather short length, in comparison to eukaryotes; only 28 in *Archaea* and 32 in *Bacteria* are longer than 1.5 Kb.

* No apparent overall differences have been detected between *Archaea* and *Bacteria*; only the percentage of satellites with long repeats (> 30 nt) is significantly larger in *Bacteria* (42.6%) than in *Archaea* (17.5%). Satellites with short repeats are related to microsatellites.

* Satellites are often part of genes coding for proteins with amino acid repeats. This is the case of most satellites with long repeats (> 30 nt).

* Satellites with a similar repeat size and composition have been grouped into families. These families are usually species specific; only a few of them are shared with related species. We have detected no family shared by *Archaea* and *Bacteria*.

* We have described the specific features of a few species which have a comparatively large number of satellites. We find different models of satellite distribution in them, depending on which type of satellite repeat predominates: short repeats, non-coding satellites or satellites embedded in genes coding for amino acid repeats in proteins.

* We have only found satellite families with long repeats and no apparent coding function in the Bacteria *Leptospira interrogans* and the Archaea *Methanocella conradii*. This indicates the presence of a satellite transposition mechanism in these species, which appears to be a rare feature in prokaryotes. It suggests an isolated episode of information transfer between eukaryotes and prokaryotes.

## Methods

We have downloaded the genome sequences from the reference group of species found in the NCBI website: 142 archaea and 119 bacteria [27]. All their genomes have been fully sequenced. These groups are rather heterogeneous; many genus are not represented, whereas several genomes are provided for the most studied groups. Two files were built containing respectively all the archaeal and all the bacterial genomes in alphabetical order. These two files were used to identify the satellites. For comparison we also determined the satellites from an eukaryote, using the recently recalculated *C. elegans* genome [28].

Long tandem repeats (satellites) were identified with the program SATFIND, which is available on-line for general use in our website [29]. The underlying algorithm is described in a previous publication [7]. Its source code has been deposited in Dryad [30]. The program determines the localization of clusters of any short sequence of a prefixed size without internal repetitions and repeated a minimum number of times in regions with a fixed size. In this paper we have used the SATFIND program to identify satellites formed by at least four repeats of any decamer sequence in 800 nt long regions. Once a satellite is located, the program continues its search along the genome until no further neighboring repeats are detected. In this way repeats of 10–260 nucleotides repeated at least 4 times can be positioned in the genome, with no upper limit for the number of repeats in the satellite.

Most satellites have a regular structure, but there is a significant number which present variations in repeat length and composition. In order to eliminate irregular satellites, we have only accepted those which have at least 60% of their repeats with an identical length. Decreasing this percentage will provide more satellites, but less regular, with frequent indels in their repeats. The regularity of each satellite is characterized by two parameters; Ni gives the number of repeats in the satellite which have an identical length and an alignment score is calculated for these Ni repeats. These values are given in Additional file 3.

To compare satellites we have used Malig, a progressive multiple sequence alignment algorithm, which we have developed to align satellite repeats and identify families with a related sequence. Its source code has been deposited in Dryad [31]. As a progressive method, Malig first computes the similarity score between all pairs of sequences using a dynamic programming algorithm [32]. The program considers reverse sequences as well, normalizes the alignment score to the maximum possible value and selects the cycle permutation with the highest score. Then the progressive multi-alignment is applied to the matrix of pairwise alignment scores. The process finishes when the score is smaller than a similarity threshold (input parameter) which we set to 0.6. We have applied this method to the detection of satellites with a common repeat sequence.

Each family is characterized by three values, eg Fam_*a_b_c*. The order in the list of families is given by “*a”,* starting with those families with the largest number of members. The second value “*b”* gives the size of the repeat; “*c”* gives the number of members in the family. Unique satellites appear at the end of the list, as families with a single member (*c* = 1). The consensus sequence of the repeat is calculated taking into account the circularly permuted sequence of all repeats.

## Additional files

Additional file 1: **Table S1.** A list of all the species studied, including the NCBI description, genome size, CG%, number of satellites and satellites/Mb. (XLSX 86 kb)

Additional file 2: **Table S2.** Nucleotide sequence and properties of all satellites in every species. Archaeal satellites are given first, followed by bacterial satellites. (FA 822 kb)

Additional file 3: **Table S3.** A list of all satellites and their properties in every species. (XLSX 222 kb)

Additional file 4: **Table S4.** A list of satellites longer than 1.5 kb. (PDF 138 kb)

Additional file 5: **Figures S1** and **S2.** They show the repeat size distribution in Archaea (S1) and in Bacteria (S2). (PDF 269 kb)

Additional file 6: **Table S5.** A list of all archaeal satellite families followed by the bacterial satellite families. Unique satellites appear at the end of each group, as families with one member. The repeat sequence of all satellites in each family is given. (DOCX 426 kb)

Additional file 7: **Table S6.** A summary list of all satellite families. (PDF 458 kb)

## Data Availability

All data generated or analyzed during this study are included in this published article and its Additional files 1, 2, 3, 4, 5, 6, 7.

## Abbreviation

CRISPR motif: Clustered Regularly Interspaced Short Palindromic Repeats
