# Supplementary material for: Satellites in the prokaryote world
Source: BMC Evol Biol. 2019 Sep 18;19:181. doi: 10.1186/s12862-019-1504-2 (PMC6749651; doi:10.1186/s12862-019-1504-2)
Supplement: Supplementary file 4 — Table S4. A list of satellites longer than 1.5 kb. (PDF 138 kb) [file 12862_2019_1504_MOESM4_ESM.pdf]

# Table S4

# Archaea. Longest satellites

| Species | Code | Satellite Start | Satellite Length | Number of repeats | Repeat length | Ni | Score |
|---|---|---|---|---|---|---|---|
| NC_018876.1_Methanolobus_psychrophilus_R15 | 74 | 3058409 | 3921 | 23 | 147 | 14 | 0.6907 |
| NC_015416.1_Methanothrix_soehngenii_=_Methanosaeta_concilii_GP-6 | 97 | 2497766 | 3826 | 12 | 255 | 9 | 0.6354 |
| NZ_CP009515.1_Methanosarcina_lacustris_Z-7289 | 84 | 506749 | 3278 | 53 | 39 | 46 | 0.888 |
| NC_007796.1_Methanospirillum_hungatei_JF-1 | 91 | 1464547 | 2875 | 55 | 42 | 42 | 0.6057 |
| NZ_CP009506.1_Methanosarcina_siciliae_T4/M | 86 | 2150348 | 2431 | 11 | 126 | 7 | 0.6792 |
| NZ_CP009516.1_Methanosarcina_horonobensis_HB-1 | 83 | 4033713 | 2314 | 8 | 255 | 5 | 0.6639 |
| NZ_CP009506.1_Methanosarcina_siciliae_T4/M | 86 | 1980464 | 2125 | 14 | 108 | 10 | 0.5935 |
| NZ_CP009515.1_Methanosarcina_lacustris_Z-7289 | 84 | 2396966 | 2017 | 8 | 252 | 8 | 0.8863 |
| NZ_CP009520.1_Methanosarcina_vacuolata_Z-761 | 88 | 3931052 | 1960 | 16 | 108 | 14 | 0.8677 |
| NZ_CP009520.1_Methanosarcina_vacuolata_Z-761 | 88 | 3373643 | 1954 | 18 | 93 | 14 | 0.7334 |
| NZ_CP009528.1_Methanosarcina_barkeri_MS | 82 | 2243257 | 1855 | 14 | 123 | 10 | 0.7445 |
| NC_015574.1_Methanobacterium_paludis__SWAN1 | 47 | 1056639 | 1849 | 20 | 69 | 12 | 0.6498 |
| NC_003552.1_Methanosarcina_acetivorans_str._C2A | 80 | 3137895 | 1843 | 15 | 123 | 14 | 0.7496 |
| NC_003552.1_Methanosarcina_acetivorans_str._C2A | 80 | 2347432 | 1816 | 7 | 255 | 5 | 0.6062 |
| NZ_CP009501.1_Methanosarcina_thermophila_TM-1 | 87 | 1491871 | 1804 | 8 | 255 | 5 | 0.7089 |
| NZ_CP009516.1_Methanosarcina_horonobensis_HB-1 | 83 | 4667608 | 1798 | 7 | 258 | 5 | 0.5873 |
| NC_015416.1_Methanothrix_soehngenii_=_Methanosaeta_concilii_GP-6 | 97 | 2581430 | 1639 | 12 | 126 | 11 | 0.7916 |
| NZ_CP009528.1_Methanosarcina_barkeri_MS | 82 | 2957391 | 1600 | 13 | 123 | 10 | 0.7408 |
| NC_003552.1_Methanosarcina_acetivorans_str._C2A | 80 | 4063362 | 1585 | 11 | 144 | 11 | 0.8648 |
| NZ_CP009520.1_Methanosarcina_vacuolata_Z-761 | 88 | 912557 | 1579 | 6 | 264 | 5 | 0.9333 |
| NC_017527.1_Methanosaeta_harundinacea_6Ac | 78 | 1496150 | 1564 | 10 | 150 | 9 | 0.7904 |
| NC_003552.1_Methanosarcina_acetivorans_str._C2A | 80 | 2350333 | 1561 | 10 | 126 | 6 | 0.4655 |
| NZ_CP009520.1_Methanosarcina_vacuolata_Z-761 | 88 | 598900 | 1548 | 6 | 258 | 5 | 0.9494 |
| NZ_CP009528.1_Methanosarcina_barkeri_MS | 82 | 1451926 | 1516 | 6 | 246 | 4 | 0.9106 |
| NZ_CP009520.1_Methanosarcina_vacuolata_Z-761 | 88 | 2165164 | 1513 | 13 | 108 | 10 | 0.6825 |
| NC_017527.1_Methanosaeta_harundinacea_6Ac | 78 | 2246493 | 1513 | 11 | 126 | 10 | 0.776 |
| NC_007796.1_Methanospirillum_hungatei_JF-1 | 91 | 1468566 | 1495 | 6 | 249 | 4 | 0.8522 |

# Bacteria. Longest satellites

| NCBI code | Species Code | Satellite Start | Satellite Length | Nr repeats | Repeat length | Ni | Score |
|---|---|---|---|---|---|---|---|
| NC_009613 | 52 | 2274517 | 5662 | 20 | 297 | 14 | 0.9911 |
| NC_004567 | 65 | 1197875 | 4753 | 200 | 18 | 139 | 0.7437 |
| NC_004113 | 113 | 1066146 | 3898 | 28 | 138 | 22 | 0.7964 |
| NC_002967 | 116 | 367152 | 3762 | 58 | 66 | 55 | 0.2611 |
| NC_006841 | 120 | 1309867 | 3565 | 14 | 255 | 9 | 0.8117 |
| NC_004116 | 103 | 448190 | 3319 | 14 | 237 | 14 | 1 |
| NC_015663 | 61 | 3319081 | 3043 | 147 | 18 | 121 | 0.7717 |
| NC_009613 | 52 | 888739 | 2920 | 12 | 243 | 11 | 0.9733 |
| NC_002516 | 85 | 5187169 | 2905 | 12 | 243 | 8 | 0.8323 |
| NC_004605 | 122 | 1426926 | 2833 | 10 | 228 | 6 | 0.9739 |
| NC_009009 | 108 | 808979 | 2665 | 56 | 36 | 34 | 0.7142 |
| NC_003902 | 123 | 611083 | 2641 | 46 | 48 | 38 | 0.5476 |
| NC_007005 | 87 | 1808215 | 2545 | 36 | 48 | 25 | 0.5567 |
| NC_005957 | 10 | 3540669 | 2521 | 56 | 45 | 45 | 0.8278 |
| NC_011661 | 43 | 1306108 | 2495 | 14 | 156 | 10 | 0.7726 |
| NC_006055 | 72 | 518523 | 2467 | 10 | 261 | 9 | 0.9879 |
| NC_010175 | 33 | 3822728 | 2377 | 12 | 198 | 9 | 0.7385 |
| NC_010175 | 33 | 4575796 | 2269 | 18 | 126 | 18 | 0.8689 |
| NC_016603 | 1 | 2278104 | 2077 | 5 | 258 | 3 | 0.7588 |
| NC_004342 | 69 | 2941021 | 2014 | 17 | 69 | 11 | 0.6251 |
| NC_007929 | 66 | 120838 | 1899 | 28 | 66 | 27 | 0.2806 |
| NC_010175 | 33 | 324795 | 1882 | 25 | 48 | 17 | 0.9252 |
| NC_004342 | 69 | 3289635 | 1757 | 14 | 69 | 9 | 0.6714 |
| NC_009636 | 99 | 3729038 | 1756 | 8 | 219 | 5 | 0.7204 |
| NC_007005 | 87 | 5621795 | 1755 | 15 | 118 | 11 | 0.9441 |
| NC_014318 | 4 | 341402 | 1723 | 10 | 153 | 9 | 0.3366 |
| NC_013853 | 104 | 1548744 | 1711 | 18 | 54 | 13 | 0.8873 |
| NC_007644 | 74 | 505880 | 1656 | 23 | 72 | 16 | 0.3241 |
| NC_004116 | 103 | 908162 | 1651 | 25 | 66 | 23 | 0.3432 |
| NC_013853 | 104 | 1716731 | 1549 | 27 | 48 | 22 | 0.5148 |
| NC_009009 | 108 | 666850 | 1537 | 6 | 252 | 4 | 0.7756 |
| NC_009613 | 52 | 1325546 | 1516 | 20 | 76 | 15 | 0.4394 |
