# Supplementary material for: Satellites in the prokaryote world
Source: BMC Evol Biol. 2019 Sep 18;19:181. doi: 10.1186/s12862-019-1504-2 (PMC6749651; doi:10.1186/s12862-019-1504-2)
Supplement: Supplementary file 5 — Figures S1 and S2. They show the repeat size distribution in Archaea (S1) and in Bacteria (S2). (PDF 269 kb) [file 12862_2019_1504_MOESM5_ESM.pdf]

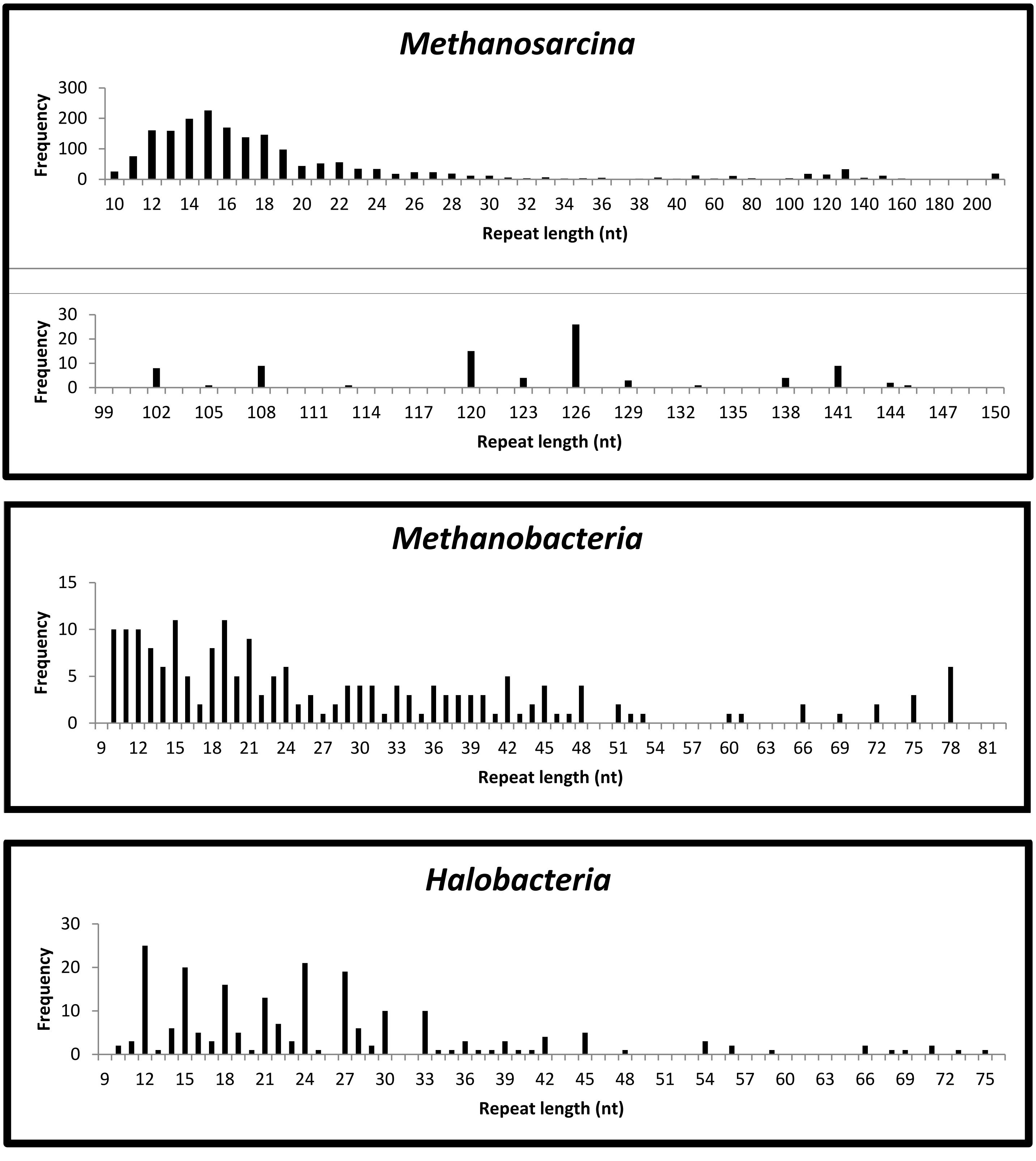

Fig. S1 Repeat size distribution of satellites in the genomes of different groups of *Archaea*. The number of satellites found is represented as a function of their repeat size. Note that every frame is presented in a different scale. In the upper frame, repeats longer than 40 bases have been merged in bins of 10 bases. Repeats with a length multiple of three correspond in most cases to parts of genes which codify for amino acid repeats. They are especially prominent in *Halobacteria*.

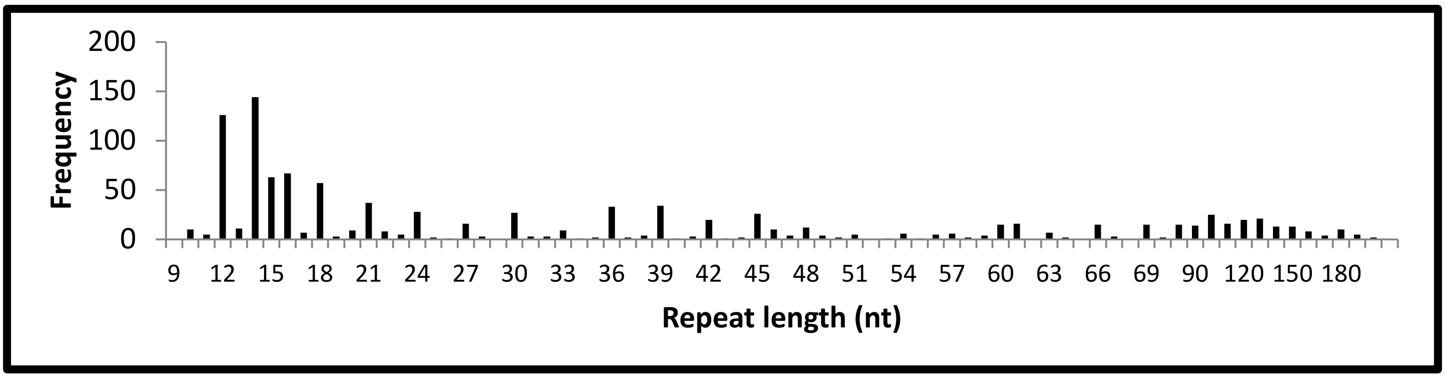

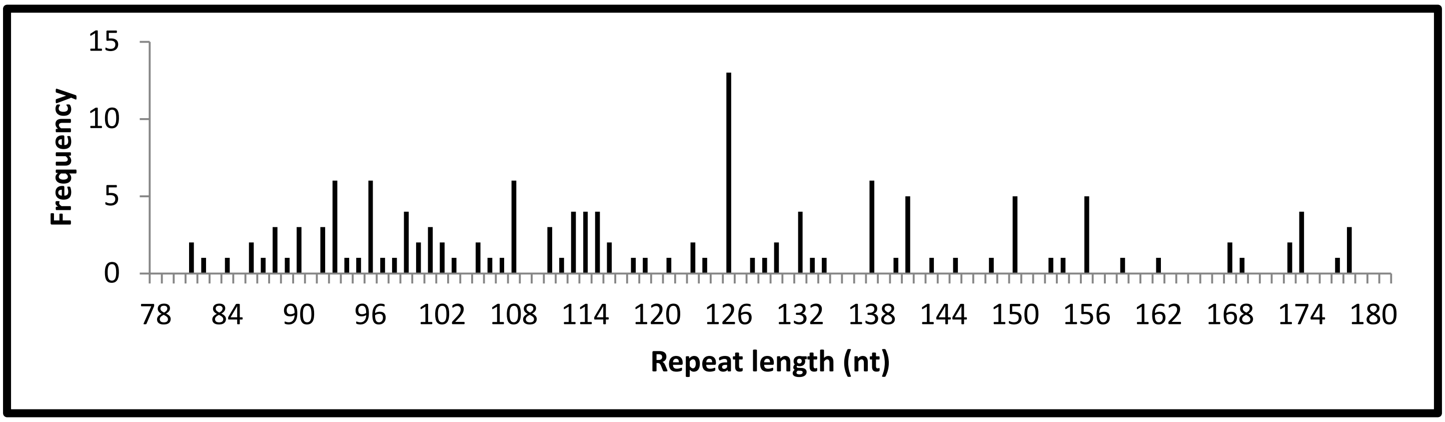

Fig. S2 Repeat size distribution of satellites in the genomes of all the bacteria we have studied. The number of satellites found is represented as a function of their repeat size. In the upper frame, repeats longer than 70 bases have been merged in bins of 10 bases. This region is shown in more detail in the lower frame. Satellites with the same repeat length are often unrelated. For example, the peak found with a repeat of 126 nt represents 13 satellites, all with a different sequence.
