# Supplementary material for: Satellites in the prokaryote world
Source: BMC Evol Biol. 2019 Sep 18;19:181. doi: 10.1186/s12862-019-1504-2 (PMC6749651; doi:10.1186/s12862-019-1504-2)
Supplement: Supplementary file 6 — Table S5. A list of all archaeal satellite families followed by the bacterial satellite families. Unique satellites appear at the end of each group, as families with one member. The repeat sequence of all satellites in each family is given. (DOCX 426 kb) [file 12862_2019_1504_MOESM6_ESM.docx]

**Table S5. Satellite families**

>arqueas_representative_Fam_1_126_16 Nr. of seq. 16 Alignment length(with gaps) = 136 Alignment score = 0.626532

arqueas_representative:CP009516.1_Methanosarcina_horonobensis_HB-1:83:4557569-4558331 Satlength=763 Nr of Repeats=6 RepeatLength=126 seed=GTAATTGACA Num.seqs=5 Similarity=0.469014 tpcCG=41.299999 0 GTAATTGACACAGCNACAAATAATGTTANAGCCACNGTTAATGTAGGA--ATTNATCCTTGNGGAATTGCAGTTAACCCGGATGGA-ACAAAAGTATATGTGACTAA-------NCAG-NAATAATACNANTNTCT

Rev.of_arqueas_representative:NZ_CP009517.1_Methanosarcina_barkeri_3:81:2755703-2756453 Satlength=751 Nr of Repeats=5 RepeatLength=126 seed=TGTAACCTTG Num.seqs=4 Similarity=0.626859 tpcCG=39.099998 30 GTAATTGACACAGCAACAAACAAGGTTACAGCTACAGTAAATGTAGGA--AATTATCCTTGNGGAGTTGCAGTCAATCCGGCTGGA-ACAAAAGTGTATGTGGCGAA----C--GAAGCTAGTGGCGCT-GTTTCG

arqueas_representative:NZ_CP009528.1_Methanosarcina_barkeri_MS:82:582048-582546 Satlength=499 Nr of Repeats=4 RepeatLength=126 seed=GGAGTTGCAG Num.seqs=3 Similarity=0.631352 tpcCG=39.168999 60 GTAATTGACACTGCAACAAACAAGGTTACAGCCACAGTGAAGGTAGGA--AAATATCCCTGNGGAGTTGCAGTNAACCCNGCTGGA-ACNAAGGTATATGTGNCGAA----A--GAAGCNAGTAACACT-GTCTCT

arqueas_representative:NZ_CP009520.1_Methanosarcina_vacuolata_Z-761:88:602540-603038 Satlength=499 Nr of Repeats=4 RepeatLength=126 seed=TCTGTAATTG Num.seqs=3 Similarity=0.629630 tpcCG=39.732498 123 GTAATTGACACTGCAANAAACAAGGTTACAGCCACAGTGAATGTAGGA--AAATATCCCTGNGGAGTTGCAGTTAACCCGGCNGGA-ACAAAGGTATATGTGGCGAA----N--GAANNTAGTAANACT-GTTTCT

Rev.of_arqueas_representative:NZ_CP009520.1_Methanosarcina_vacuolata_Z-761:88:654276-654774 Satlength=499 Nr of Repeats=4 RepeatLength=126 seed=ACATATACCT Num.seqs=3 Similarity=0.629921 tpcCG=39.73249 98 GTAATTGACACTNCAACAAACAAGGTTACAGCTACAGTGANTGTAGGA--AATNACCCTTGCGGAGTTGCAGTTAACCCGGCGGGA-ACAAAGGTATATGTAGCGAA----T--GAAGCNAGTAANACT-GTCTCT

Rev.of_arqueas_representative:NZ_CP009528.1_Methanosarcina_barkeri_MS:82:627781-628279 Satlength=499 Nr of Repeats=4 RepeatLength=126 seed=GCTGTAACCT Num.seqs=3 Similarity=0.685039 tpcCG=39.168999 32 GTNATTAACACTGCAACAAATAAGGTTACAGCNACAGTAAAAGTAGGA--AAATACCCATGGGGAGTTGCAGTAAACCCGGCTGGA-ACAAAGGTATATGTGACGAACTANT--GNAG--A-TAANACT-GTCTCT

arqueas_representative:NZ_CP009528.1_Methanosarcina_barkeri_MS:82:710511-711147 Satlength=637 Nr of Repeats=5 RepeatLength=126 seed=AAAGGTATAT Num.seqs=4 Similarity=0.641026 tpcCG=39.168999 86 GTAATTGACACAGCTACAAACAAGGTTACAGCCAAGGTAACCGTAGGA--TNATATCCTTATGGAGTTGCAGTCAACCCGGCTGGA-ACAAAGGTATATGTGACAAACTA-T--GAAA--AGCAAAACT-GTCTCT

Rev.of_arqueas_representative:NZ_CP009506.1_Methanosarcina_siciliae_T4/M:86:2150348-2152778 Satlength=2431 Nr of Repeats=11 RepeatLength=126 seed=CCACATATAC Num.seqs=7 Similarity=0.679217 tpcCG=42.900 100 GTGATTGACACTGCCACNGACAATGTTACANCCACNGTNAATGTAGGA--AGTNATCCTTGGGGAGTTGCAGTCANNCCNGATGGA-ACAAAAGTATATGTGGCAAACTC-T--GGA---ACCAGCAGT-GTCTCT

Rev.of_arqueas_representative:NZ_CP009520.1_Methanosarcina_vacuolata_Z-761:88:2347466-2348123 Satlength=658 Nr of Repeats=5 RepeatLength=126 seed=TCAATTACAG Num.seqs=3 Similarity=0.731624 tpcCG=39.732 8 GTAATTGATACAGCTACAAACACTGTTACAGCCACTGTGCCTGTAGGA--AGCTCTCCTTACGGAGTTGCAGTCAACTCGGCAGGA-ACAAAAGTATATGTGACGAA--C-T--CT-GNCAGCAACANT-GTATCT

Rev.of_arqueas_representative:NZ_CP009520.1_Methanosarcina_vacuolata_Z-761:88:2370361-2371123 Satlength=763 Nr of Repeats=6 RepeatLength=126 seed=TCAATTACAG Num.seqs=4 Similarity=0.683463 tpcCG=39.732 8 GTAATTGACGCAGCTACAAATACTGTTACAGCCACTGTACCTGTAGGG--TGCAATCCTTGTGGAGTTGCAGTTAACCCGGATGGA-ACAAAGGTATATGTGGCGAA--C-T---TCGGCAGTAATACT-GTCTCT

Rev.of_arqueas_representative:NZ_CP009520.1_Methanosarcina_vacuolata_Z-761:88:659370-660087 Satlength=718 Nr of Repeats=5 RepeatLength=126 seed=GTGTCAATTA Num.seqs=4 Similarity=0.592735 tpcCG=39.73249 11 GTAATTGACACTGCCACAAATACTGTTACAGCCACTGTGCCTGTAGGA--GGCTCTCCTTTTGGAGTTGCAGTCACCCCGGATGGA-ACAAAGGTATATGTGACGAA--N-TA-CGCGGCAGTGGCACT-GTCTCT

arqueas_representative:NZ_CP009512.1_Methanosarcina_mazei_S-6:85:1295867-1296371 Satlength=505 Nr of Repeats=4 RepeatLength=126 seed=GTATATGTGG Num.seqs=4 Similarity=0.610680 tpcCG=41.400002 92 GTAATTGATACAACAACCAGCACTGTTACCGCCACGGTGCCTGTAGGANTTGCAGTCCTTATGGAGTTGCAATCAGTCCGGATGGA-ACAAAGGTATATGTGGCGAA--C-T--AT-GACAGCAACACT-ACCTCT

Rev.of_arqueas_representative:CP009516.1_Methanosarcina_horonobensis_HB-1:83:4119493-4120120 Satlength=628 Nr of Repeats=5 RepeatLength=126 seed=CAATTACAGA Num.seqs=4 Similarity=0.545220 tpcCG=41.2999 134 GTAATTGACACAGCAACAAATACTGTTGTAGCTACTGTGGCTGTAGGA--AATATTCCTTTTGGAGTTGCAGTCAGTCTGGATGGA-ACAAAGGTATATGTGGCGAA--N-C--ATGGGTAGCGGCACT-GTATCT

Rev.of_arqueas_representative:NC_003552.1_Methanosarcina_acetivorans_str._C2A:80:2350333-2351893 Satlength=1561 Nr of Repeats=10 RepeatLength=126 seed=TCAATTACAG Num.seqs=6 Similarity=0.465539 tpcCG=4 8 GTAATTGACACGTCCACAAATACTGTTATAGCTACGGTACCTGTNGGA--ANCAATCCTNTGGGAGTTGCAGTCANCCCGGATGGA-ACAAAGGTATATGTGGCAAA--T-T-NNCCGGTAACAATANN-GTCTCT

arqueas_representative:NZ_CP009528.1_Methanosarcina_barkeri_MS:82:936256-937015 Satlength=760 Nr of Repeats=5 RepeatLength=126 seed=TCTGTAATTG Num.seqs=3 Similarity=0.640625 tpcCG=39.168999 123 GTAATTGACACTGCAACTAANACTGTTACATCAACGGTGNATGTNGGN--NATTNTCCTGTTGGAGTTGCAGCNTCNCCGGATGGANAC-AAGGTGTATGTTGCNAA--C----GCTGGCAGCAACAAT-GTTTCT

arqueas_representative:NZ_CP009520.1_Methanosarcina_vacuolata_Z-761:88:3602907-3603666 Satlength=760 Nr of Repeats=6 RepeatLength=126 seed=CTGTAATTGA Num.seqs=5 Similarity=0.644103 tpcCG=39.732498 124 GTAATTGACACTGNAACTAACACTGTTACATCAACNGTGANTGTNGGN--AAATNTCCTGTTGGAGTTGCAGTCTCTCCGGATGGA-ACAAAGGTATATGTGACGAA--C----GCNGGCAGCAACAAT-GTTTCT

** *** * * * * *** * * ** ** ** ** *** ***** * *** ** ** ** ***** * ** * **

Consensus:

GTAATTGACACtGCaACaAAcAatGTTACAGCcACnGTganTGTAGGAaantaTCCTTgnGGAGTTGCAGTcAacCCGGaTGGAACAAAgGTATATGTGgCgAAntgnaGnnAgcAacAcTGTcTCT

>arqueas_representative_Fam_2_12_13 Nr. of seq. 13 Alignment length(with gaps) = 13 Alignment score = 0.648258

arqueas_representative:NZ_CP009515.1_Methanosarcina_lacustris_Z-7289:84:3508370-3508480 Satlength=111 Nr of Repeats=10 RepeatLength=11 seed=CTTATCATTA Num.seqs=10 Similarity=1.000000 tpcCG=41.799999 0 C-TTATCATTAG-

arqueas_representative:NC_014222.1_Methanococcus_voltae_A3:65:1731120-1731168 Satlength=49 Nr of Repeats=4 RepeatLength=12 seed=CATTATCATT Num.seqs=4 Similarity=0.925926 tpcCG=28.600000 0 CATTATCATTAG-

arqueas_representative:NC_014222.1_Methanococcus_voltae_A3:65:148398-148446 Satlength=49 Nr of Repeats=4 RepeatLength=12 seed=TTATTATTAG Num.seqs=4 Similarity=0.888889 tpcCG=28.600000 1 -ATTATTATTAGG

Rev.of_arqueas_representative:NC_014222.1_Methanococcus_voltae_A3:65:747798-747870 Satlength=73 Nr of Repeats=6 RepeatLength=12 seed=ATAATAATAC Num.seqs=6 Similarity=0.962963 tpcCG=28.600000 8 -ATTATTATTCGT

arqueas_representative:NC_014222.1_Methanococcus_voltae_A3:65:1083733-1084322 Satlength=590 Nr of Repeats=5 RepeatLength=12 seed=TATTATTAGT Num.seqs=3 Similarity=0.925926 tpcCG=28.600000 11 -ATTATTATTAGT

Rev.of_arqueas_representative:NC_014222.1_Methanococcus_voltae_A3:65:1843206-1843254 Satlength=49 Nr of Repeats=4 RepeatLength=12 seed=ATAATAATAC Num.seqs=4 Similarity=1.000000 tpcCG=28.600000 20 -ATTATTATTAGT

arqueas_representative:NC_014222.1_Methanococcus_voltae_A3:65:873193-873253 Satlength=61 Nr of Repeats=5 RepeatLength=12 seed=TATTATTCTT Num.seqs=5 Similarity=1.000000 tpcCG=28.600000 14 -AGTATTATTCTT

arqueas_representative:NC_014222.1_Methanococcus_voltae_A3:65:889325-889385 Satlength=61 Nr of Repeats=4 RepeatLength=12 seed=TATTAGTATT Num.seqs=3 Similarity=0.851852 tpcCG=28.600000 17 -ATTAGTATTAGT

Rev.of_arqueas_representative:NC_014222.1_Methanococcus_voltae_A3:65:273112-273256 Satlength=145 Nr of Repeats=8 RepeatLength=12 seed=AATAAAAATA Num.seqs=6 Similarity=0.822222 tpcCG=28.600000 3 -ATTTTTATTATT

arqueas_representative:NC_014222.1_Methanococcus_voltae_A3:65:1252128-1252188 Satlength=61 Nr of Repeats=5 RepeatLength=12 seed=TTTTATTATT Num.seqs=3 Similarity=0.925926 tpcCG=28.600000 14 -ATTTTTATTATT

arqueas_representative:NZ_CP009515.1_Methanosarcina_lacustris_Z-7289:84:3888610-3888802 Satlength=193 Nr of Repeats=16 RepeatLength=12 seed=TATTAGTATT Num.seqs=11 Similarity=0.878788 tpcCG=41.799999 14 -ATTATTATTATT

Rev.of_arqueas_representative:NC_014222.1_Methanococcus_voltae_A3:65:856390-856438 Satlength=49 Nr of Repeats=4 RepeatLength=12 seed=TAATAGTAAT Num.seqs=4 Similarity=0.944444 tpcCG=28.600000 13 -ACTATTATTATT

Rev.of_arqueas_representative:NC_007681.1_Methanosphaera_stadtmanae_DSM_3091:89:557160-557886 Satlength=727 Nr of Repeats=14 RepeatLength=12 seed=TTCTAATAAT Num.seqs=13 Similarity=1.000000 tpcCG=27.60 1 -ACTATTATTAGA

* ***

Consensus:

AtTATTATTAgt

>arqueas_representative_Fam_3_12_10 Nr. of seq. 10 Alignment length(with gaps) = 14 Alignment score = 0.625132

arqueas_representative:NC_014222.1_Methanococcus_voltae_A3:65:764687-764754 Satlength=68 Nr of Repeats=6 RepeatLength=11 seed=TAAAAATAAA Num.seqs=5 Similarity=0.927273 tpcCG=28.600000 6 AT-AAAA-TAAA-A

Rev.of_arqueas_representative:NZ_CP009512.1_Methanosarcina_mazei_S-6:85:2414170-2414218 Satlength=49 Nr of Repeats=4 RepeatLength=12 seed=TTTTTATTTT Num.seqs=4 Similarity=1.000000 tpcCG=41.400002 6 AT-AAAAATAAA-A

arqueas_representative:NC_014222.1_Methanococcus_voltae_A3:65:212495-212627 Satlength=133 Nr of Repeats=11 RepeatLength=12 seed=ACTAAAAATA Num.seqs=11 Similarity=1.000000 tpcCG=28.600000 10 CT-AAAAATAAA-A

arqueas_representative:NZ_CP009517.1_Methanosarcina_barkeri_3:81:903918-903996 Satlength=79 Nr of Repeats=5 RepeatLength=12 seed=AAAATTAAAA Num.seqs=3 Similarity=0.777778 tpcCG=39.099998 7 TT-AAAAATAAA-A

arqueas_representative:NZ_CP009520.1_Methanosarcina_vacuolata_Z-761:88:824250-824316 Satlength=67 Nr of Repeats=5 RepeatLength=12 seed=ATTAAAATTA Num.seqs=4 Similarity=1.000000 tpcCG=39.732498 10 TT-AAAATTAAA-A

Rev.of_arqueas_representative:NZ_CP009517.1_Methanosarcina_barkeri_3:81:1028182-1028230 Satlength=49 Nr of Repeats=4 RepeatLength=12 seed=TTTACTTTTA Num.seqs=4 Similarity=0.944444 tpcCG=39.099998 10 GT-AAAAGTAAA-A

Rev.of_arqueas_representative:NC_014222.1_Methanococcus_voltae_A3:65:749638-749686 Satlength=49 Nr of Repeats=4 RepeatLength=12 seed=TTTATTTTTA Num.seqs=4 Similarity=0.743590 tpcCG=28.600000 10 GT-AAAAATAAANA

arqueas_representative:NZ_CP009528.1_Methanosarcina_barkeri_MS:82:2196832-2196949 Satlength=118 Nr of Repeats=9 RepeatLength=13 seed=AAAATAAAGA Num.seqs=9 Similarity=1.000000 tpcCG=39.168999 15 GT-AAAAATAAAGA

Rev.of_arqueas_representative:NZ_CP009528.1_Methanosarcina_barkeri_MS:82:279264-279554 Satlength=291 Nr of Repeats=24 RepeatLength=12 seed=TTTACTTTTG Num.seqs=21 Similarity=1.000000 tpcCG=39.168999 10 TC-AAAAGTAAA-A

Rev.of_arqueas_representative:NZ_CP009528.1_Methanosarcina_barkeri_MS:82:3976323-3976697 Satlength=375 Nr of Repeats=13 RepeatLength=13 seed=AATTTACTTT Num.seqs=11 Similarity=1.000000 tpcCG=39.168999 12 TCAAAAAGTAAA-T

**** ****

Consensus:

TAAAAntAAAAa

>arqueas_representative_Fam_4_12_10 Nr. of seq. 10 Alignment length(with gaps) = 12 Alignment score = 0.642593

arqueas_representative:NC_014222.1_Methanococcus_voltae_A3:65:714511-715274 Satlength=764 Nr of Repeats=7 RepeatLength=11 seed=TATTATTTTA Num.seqs=5 Similarity=0.808333 tpcCG=28.600000 0 TATTATT-TTAT

arqueas_representative:NC_014222.1_Methanococcus_voltae_A3:65:956693-956781 Satlength=89 Nr of Repeats=8 RepeatLength=11 seed=TATTTTATTC Num.seqs=8 Similarity=1.000000 tpcCG=28.600000 3 TCTTATT-TTAT

arqueas_representative:NZ_CP009515.1_Methanosarcina_lacustris_Z-7289:84:3838501-3838611 Satlength=111 Nr of Repeats=10 RepeatLength=11 seed=ATTCTTTTCT Num.seqs=8 Similarity=0.917749 tpcCG=41.799999 1 TATTCTT-TTCT

Rev.of_arqueas_representative:NZ_CP009517.1_Methanosarcina_barkeri_3:81:2384323-2384437 Satlength=115 Nr of Repeats=9 RepeatLength=12 seed=AATAAGAATA Num.seqs=8 Similarity=1.000000 tpcCG=39.099998 4 TATTCTTATTCT

Rev.of_arqueas_representative:NZ_CP009517.1_Methanosarcina_barkeri_3:81:3635510-3635636 Satlength=127 Nr of Repeats=10 RepeatLength=12 seed=AATAAGAATA Num.seqs=9 Similarity=0.888889 tpcCG=39.099998 4 TATTCTTATTCT

Rev.of_arqueas_representative:NC_014729.1_Halogeometricum_borinquense_DSM_11551:27:190752-190800 Satlength=49 Nr of Repeats=4 RepeatLength=12 seed=AATAACAATA Num.seqs=4 Similarity=1.000000 tpcCG=59.97 1 TGTTATTGTTAT

Rev.of_arqueas_representative:NZ_CP009517.1_Methanosarcina_barkeri_3:81:229324-229372 Satlength=49 Nr of Repeats=4 RepeatLength=12 seed=TAACAATAAC Num.seqs=4 Similarity=1.000000 tpcCG=39.099998 11 TGTTATTGTTAT

Rev.of_arqueas_representative:NZ_CP009517.1_Methanosarcina_barkeri_3:81:1292215-1292383 Satlength=169 Nr of Repeats=14 RepeatLength=12 seed=AGTAACAATA Num.seqs=14 Similarity=0.945055 tpcCG=39.099998 1 TGTTATTGTTAC

Rev.of_arqueas_representative:NZ_CP009517.1_Methanosarcina_barkeri_3:81:3406077-3406161 Satlength=85 Nr of Repeats=6 RepeatLength=12 seed=AATAACAACA Num.seqs=4 Similarity=0.925926 tpcCG=39.099998 1 TGTTGTTGTTAT

Rev.of_arqueas_representative:NZ_CP009517.1_Methanosarcina_barkeri_3:81:2503660-2503896 Satlength=237 Nr of Repeats=6 RepeatLength=12 seed=CAACAATATC Num.seqs=4 Similarity=1.000000 tpcCG=39.099998 11 TGATATTGTTGT

* * ** **

Consensus:

TgTTaTTgTTaT

>arqueas_representative_Fam_5_16_8 Nr. of seq. 8 Alignment length(with gaps) = 16 Alignment score = 0.667411

arqueas_representative:NZ_CP014265.1_Methanobrevibacter_olleyae__YLM1:49:290683-290736 Satlength=54 Nr of Repeats=4 RepeatLength=13 seed=ATTAATTTTA Num.seqs=3 Similarity=0.931624 tpcCG=26.900000 27 T-ATTA-A-TTTTATA

arqueas_representative:NC_014222.1_Methanococcus_voltae_A3:65:878257-878326 Satlength=70 Nr of Repeats=5 RepeatLength=14 seed=TTATATTTTA Num.seqs=3 Similarity=1.000000 tpcCG=28.600000 28 T-TTTATA-TTTTATA

arqueas_representative:NZ_CP009501.1_Methanosarcina_thermophila_TM-1:87:101131-101202 Satlength=72 Nr of Repeats=5 RepeatLength=14 seed=TTTTATTAAT Num.seqs=4 Similarity=1.000000 tpcCG=41.099998 16 TATT-TTTTATTA-AT

Rev.of_arqueas_representative:NC_014222.1_Methanococcus_voltae_A3:65:55779-56557 Satlength=779 Nr of Repeats=5 RepeatLength=15 seed=AATAATAATT Num.seqs=3 Similarity=1.000000 tpcCG=28.600000 18 TATTATTTTATTA-AT

Rev.of_arqueas_representative:NC_014222.1_Methanococcus_voltae_A3:65:120035-120813 Satlength=779 Nr of Repeats=5 RepeatLength=15 seed=AATAATAATT Num.seqs=3 Similarity=1.000000 tpcCG=28.600000 18 TATTATTTTATTA-AT

arqueas_representative:NC_014222.1_Methanococcus_voltae_A3:65:988540-988720 Satlength=181 Nr of Repeats=11 RepeatLength=16 seed=TTATTATTTT Num.seqs=9 Similarity=0.962963 tpcCG=28.600000 26 TATTATTTTATTAGGT

Rev.of_arqueas_representative:NC_014222.1_Methanococcus_voltae_A3:65:1830840-1830908 Satlength=69 Nr of Repeats=4 RepeatLength=16 seed=ATAAAATAAT Num.seqs=3 Similarity=0.833333 tpcCG=28.600000 30 TATTATTTTATTATTT

Rev.of_arqueas_representative:NC_015636.1_Methanothermococcus_okinawensis_IH1:95:1445343-1445439 Satlength=97 Nr of Repeats=5 RepeatLength=16 seed=ATAAAATAAT Num.seqs=3 Similarity=0.640523 tpcCG=29.27 30 TATTATTTNATTATTT

** * *

Consensus:

aTTanttTaTTatTtt

>arqueas_representative_Fam_6_10_8 Nr. of seq. 8 Alignment length(with gaps) = 12 Alignment score = 0.679067

arqueas_representative:NC_013790.1_Methanobrevibacter_ruminantium_M1:50:2462489-2462598 Satlength=110 Nr of Repeats=9 RepeatLength=10 seed=ATAAAATAAG Num.seqs=8 Similarity=0.895238 tpcCG=32.599998 1 G-AT-AAAATAA

arqueas_representative:NC_014222.1_Methanococcus_voltae_A3:65:1131791-1131841 Satlength=51 Nr of Repeats=4 RepeatLength=10 seed=AAAATAATAT Num.seqs=3 Similarity=1.000000 tpcCG=28.600000 3 T-AT-AAAATAA

arqueas_representative:NC_014222.1_Methanococcus_voltae_A3:65:45240-45283 Satlength=44 Nr of Repeats=4 RepeatLength=11 seed=TAATATAAAA Num.seqs=3 Similarity=0.919192 tpcCG=28.600000 7 T-ATAAAAATAA

arqueas_representative:NC_014222.1_Methanococcus_voltae_A3:65:109496-109539 Satlength=44 Nr of Repeats=4 RepeatLength=11 seed=TAATATAAAA Num.seqs=3 Similarity=0.919192 tpcCG=28.600000 7 T-ATAAAAATAA

arqueas_representative:NC_014222.1_Methanococcus_voltae_A3:65:1783070-1783112 Satlength=43 Nr of Repeats=4 RepeatLength=10 seed=AATATAAAAA Num.seqs=3 Similarity=1.000000 tpcCG=28.600000 8 T-AT-AAAAAAA

Rev.of_arqueas_representative:NC_013790.1_Methanobrevibacter_ruminantium_M1:50:1173349-1173393 Satlength=45 Nr of Repeats=4 RepeatLength=11 seed=TAGTTATTTT Num.seqs=4 Similarity=1.000000 tpcCG=32.5999 2 CTAT-AAAATAA

arqueas_representative:NC_014222.1_Methanococcus_voltae_A3:65:314947-315068 Satlength=122 Nr of Repeats=9 RepeatLength=11 seed=TATTAAATAA Num.seqs=8 Similarity=0.948052 tpcCG=28.600000 11 CTAT-TAAATAA

arqueas_representative:NC_014222.1_Methanococcus_voltae_A3:65:1034613-1034731 Satlength=119 Nr of Repeats=5 RepeatLength=11 seed=AAAATAATTA Num.seqs=3 Similarity=1.000000 tpcCG=28.600000 3 TTAT-AAAATAA

** *** **

Consensus:

ATAAAATAAt

>arqueas_representative_Fam_7_12_8 Nr. of seq. 8 Alignment length(with gaps) = 12 Alignment score = 0.777282

arqueas_representative:NZ_CP009517.1_Methanosarcina_barkeri_3:81:2653013-2653070 Satlength=58 Nr of Repeats=5 RepeatLength=11 seed=TTTCACACTA Num.seqs=3 Similarity=0.919192 tpcCG=39.099998 0 -TTTCACACTAC

arqueas_representative:NC_003552.1_Methanosarcina_acetivorans_str._C2A:80:2626406-2626466 Satlength=61 Nr of Repeats=5 RepeatLength=12 seed=TTTTCACCCA Num.seqs=5 Similarity=1.000000 tpcCG=42.700001 0 TTTTCACCCAAC

arqueas_representative:CP009516.1_Methanosarcina_horonobensis_HB-1:83:4538756-4539398 Satlength=643 Nr of Repeats=13 RepeatLength=12 seed=TTTTCACCCA Num.seqs=9 Similarity=0.950617 tpcCG=41.299999 0 TTTTCACCCAAC

Rev.of_arqueas_representative:NZ_CP009515.1_Methanosarcina_lacustris_Z-7289:84:2210521-2210581 Satlength=61 Nr of Repeats=5 RepeatLength=12 seed=GGTGAAAAGT Num.seqs=5 Similarity=0.888889 tpcCG=41.7999 8 TTTTCACCCAAC

arqueas_representative:NZ_CP009512.1_Methanosarcina_mazei_S-6:85:3723873-3723980 Satlength=108 Nr of Repeats=9 RepeatLength=12 seed=TTTTCACCCA Num.seqs=8 Similarity=1.000000 tpcCG=41.400002 0 TTTTCACCCAAC

Rev.of_arqueas_representative:NZ_CP009528.1_Methanosarcina_barkeri_MS:82:456172-456256 Satlength=85 Nr of Repeats=7 RepeatLength=12 seed=GTTCGGAGAA Num.seqs=7 Similarity=0.894180 tpcCG=39.168999 0 TTTTCTCCGAAC

arqueas_representative:NZ_CP009506.1_Methanosarcina_siciliae_T4/M:86:1970851-1970911 Satlength=61 Nr of Repeats=5 RepeatLength=12 seed=TTTTCTCCGA Num.seqs=5 Similarity=1.000000 tpcCG=42.900002 0 TTTTCTCCGAAC

Rev.of_arqueas_representative:NZ_CP009515.1_Methanosarcina_lacustris_Z-7289:84:2208021-2208165 Satlength=145 Nr of Repeats=9 RepeatLength=12 seed=GAAAGGTTCG Num.seqs=6 Similarity=1.000000 tpcCG=41.799 5 CTTTCTCCGAAC

**** * **

Consensus:

tTTTCaCCcAAC

>arqueas_representative_Fam_8_16_7 Nr. of seq. 7 Alignment length(with gaps) = 17 Alignment score = 0.700280

arqueas_representative:NZ_CP009517.1_Methanosarcina_barkeri_3:81:1934202-1934345 Satlength=144 Nr of Repeats=8 RepeatLength=16 seed=TGATAAACAA Num.seqs=6 Similarity=0.822222 tpcCG=39.099998 0 TGATAAACAACT-AAAC

Rev.of_arqueas_representative:NZ_CP009517.1_Methanosarcina_barkeri_3:81:2818821-2818997 Satlength=177 Nr of Repeats=11 RepeatLength=16 seed=TGATCAGTTT Num.seqs=11 Similarity=1.000000 tpcCG=39.099998 6 TGATCAAAAACT-AAAC

arqueas_representative:NZ_CP009520.1_Methanosarcina_vacuolata_Z-761:88:400558-400622 Satlength=65 Nr of Repeats=4 RepeatLength=16 seed=TAAACTGATC Num.seqs=4 Similarity=1.000000 tpcCG=39.732498 11 TGATCAAAAACT-AAAC

Rev.of_arqueas_representative:NZ_CP009528.1_Methanosarcina_barkeri_MS:82:358792-358872 Satlength=81 Nr of Repeats=4 RepeatLength=16 seed=TTTTTCATCA Num.seqs=3 Similarity=0.944444 tpcCG=39.168999 10 TGATGAAAAACT-GAAC

Rev.of_arqueas_representative:NZ_CP009517.1_Methanosarcina_barkeri_3:81:492856-492941 Satlength=86 Nr of Repeats=4 RepeatLength=17 seed=TCAGTTTCAG Num.seqs=3 Similarity=1.000000 tpcCG=39.099998 3 TGACCAAAAACTGAAAC

Rev.of_arqueas_representative:NZ_CP009528.1_Methanosarcina_barkeri_MS:82:4197380-4197524 Satlength=145 Nr of Repeats=9 RepeatLength=16 seed=TTTAATTTTT Num.seqs=9 Similarity=1.000000 tpcCG=39.168999 15 TCAGAAAAAATT-AAAC

Rev.of_arqueas_representative:NZ_CP009520.1_Methanosarcina_vacuolata_Z-761:88:4187944-4188088 Satlength=145 Nr of Repeats=9 RepeatLength=16 seed=TTTTTTATGA Num.seqs=9 Similarity=0.930556 tpcCG=39.7324 26 TCATAAAAAACT-AAAT

* * ** ** * **

Consensus:

TgAtnAAAAACTAAAC

>arqueas_representative_Fam_9_14_7 Nr. of seq. 7 Alignment length(with gaps) = 16 Alignment score = 0.602183

arqueas_representative:NZ_CP009528.1_Methanosarcina_barkeri_MS:82:182334-182426 Satlength=93 Nr of Repeats=7 RepeatLength=13 seed=TCTGAAAGGA Num.seqs=6 Similarity=1.000000 tpcCG=39.168999 0 TCTGAAAGGAAA-A--

arqueas_representative:NZ_CP009517.1_Methanosarcina_barkeri_3:81:4266726-4266824 Satlength=99 Nr of Repeats=7 RepeatLength=14 seed=AGGAAAACTA Num.seqs=7 Similarity=1.000000 tpcCG=39.099998 6 TAAGAAAGGAAA-AC-

arqueas_representative:NZ_CP009528.1_Methanosarcina_barkeri_MS:82:4134895-4134966 Satlength=72 Nr of Repeats=5 RepeatLength=14 seed=ATGAAAGAAA Num.seqs=4 Similarity=1.000000 tpcCG=39.168999 15 TATGAAAGAAAA-AC-

arqueas_representative:NZ_CP009528.1_Methanosarcina_barkeri_MS:82:4243563-4243717 Satlength=155 Nr of Repeats=11 RepeatLength=14 seed=GAAAGGAAAA Num.seqs=11 Similarity=1.000000 tpcCG=39.168999 17 TAGGAAAGGAAA-AT-

Rev.of_arqueas_representative:NZ_CP009520.1_Methanosarcina_vacuolata_Z-761:88:1905548-1905604 Satlength=57 Nr of Repeats=4 RepeatLength=14 seed=TTCCTTTCAA Num.seqs=4 Similarity=0.952381 tpcCG=39.73249 10 T-TGAAAGGAAA-AAA

Rev.of_arqueas_representative:NC_003552.1_Methanosarcina_acetivorans_str._C2A:80:1861260-1861320 Satlength=61 Nr of Repeats=4 RepeatLength=15 seed=CTTTTAGATT Num.seqs=4 Similarity=1.000000 tpcCG=42.70 22 TCTAAAAGGAAA-AAA

arqueas_representative:NZ_CP009517.1_Methanosarcina_barkeri_3:81:3494200-3494275 Satlength=76 Nr of Repeats=5 RepeatLength=15 seed=AAAAGGAAAG Num.seqs=5 Similarity=1.000000 tpcCG=39.099998 17 T-TAAAAGGAAAGAAA

* **** *** *

Consensus:

TntgAAAGGAAAAn

>arqueas_representative_Fam_10_13_7 Nr. of seq. 7 Alignment length(with gaps) = 15 Alignment score = 0.661376

arqueas_representative:NC_015636.1_Methanothermococcus_okinawensis_IH1:95:1174011-1174080 Satlength=70 Nr of Repeats=5 RepeatLength=12 seed=TTATTATTTA Num.seqs=3 Similarity=0.851852 tpcCG=29.277700 23 TA-TTAT-TTATT-T

arqueas_representative:NC_014222.1_Methanococcus_voltae_A3:65:60486-60606 Satlength=121 Nr of Repeats=6 RepeatLength=13 seed=TATTATCTTA Num.seqs=5 Similarity=1.000000 tpcCG=28.600000 24 TA-TTATCTTATT-T

arqueas_representative:NC_014222.1_Methanococcus_voltae_A3:65:124742-124862 Satlength=121 Nr of Repeats=6 RepeatLength=13 seed=TATTATCTTA Num.seqs=5 Similarity=1.000000 tpcCG=28.600000 24 TA-TTATCTTATT-T

Rev.of_arqueas_representative:NC_014222.1_Methanococcus_voltae_A3:65:221276-221328 Satlength=53 Nr of Repeats=4 RepeatLength=13 seed=ATAAAATAAT Num.seqs=4 Similarity=1.000000 tpcCG=28.600000 23 TA-TTATTTTAT-CT

Rev.of_arqueas_representative:NZ_CP009520.1_Methanosarcina_vacuolata_Z-761:88:2934709-2934812 Satlength=104 Nr of Repeats=8 RepeatLength=13 seed=AAAAAATAAG Num.seqs=7 Similarity=1.000000 tpcCG=39.7324 33 TA-TTTTTTTAT-CT

arqueas_representative:NC_014222.1_Methanococcus_voltae_A3:65:1001861-1001939 Satlength=79 Nr of Repeats=6 RepeatLength=13 seed=TTATTTTACT Num.seqs=6 Similarity=1.000000 tpcCG=28.600000 26 TA-TTATTTTACT-T

arqueas_representative:NC_013790.1_Methanobrevibacter_ruminantium_M1:50:1367749-1367805 Satlength=57 Nr of Repeats=4 RepeatLength=14 seed=TTATTTTAAT Num.seqs=4 Similarity=1.000000 tpcCG=32.599998 26 TATTTATTTTAAT-T

*** ** * ***

Consensus:

TTATTATtTTAtt

>arqueas_representative_Fam_11_14_7 Nr. of seq. 7 Alignment length(with gaps) = 15 Alignment score = 0.634392

arqueas_representative:NZ_CP014265.1_Methanobrevibacter_olleyae__YLM1:49:1178089-1178690 Satlength=602 Nr of Repeats=10 RepeatLength=14 seed=TTCTTTAATA Num.seqs=6 Similarity=0.755556 tpcCG=26.900000 0 TTCTTTAATAATTA-

Rev.of_arqueas_representative:CP009516.1_Methanosarcina_horonobensis_HB-1:83:2616873-2616971 Satlength=99 Nr of Repeats=7 RepeatLength=14 seed=AAGGATTAAA Num.seqs=5 Similarity=0.904762 tpcCG=41.299999 13 TTCTTTAATCCTTA-

arqueas_representative:NZ_CP009515.1_Methanosarcina_lacustris_Z-7289:84:2302136-2302262 Satlength=127 Nr of Repeats=9 RepeatLength=14 seed=TCTTTATTCT Num.seqs=9 Similarity=1.000000 tpcCG=41.799999 1 TTCTTTATTCTTTA-

Rev.of_arqueas_representative:NZ_CP009528.1_Methanosarcina_barkeri_MS:82:2989175-2989238 Satlength=64 Nr of Repeats=4 RepeatLength=14 seed=TAAAGATTAA Num.seqs=3 Similarity=1.000000 tpcCG=39.168999 0 GTCCTTAATCTTTA-

arqueas_representative:NZ_CP009512.1_Methanosarcina_mazei_S-6:85:773155-773288 Satlength=134 Nr of Repeats=9 RepeatLength=14 seed=ATCTTTAATC Num.seqs=8 Similarity=1.000000 tpcCG=41.400002 0 ATCTTTAATCTTTA-

Rev.of_arqueas_representative:NZ_CP009528.1_Methanosarcina_barkeri_MS:82:271804-271860 Satlength=57 Nr of Repeats=4 RepeatLength=14 seed=TAATGAACAA Num.seqs=4 Similarity=1.000000 tpcCG=39.168999 0 TTCCTTGTTCATTA-

arqueas_representative:NZ_CP009517.1_Methanosarcina_barkeri_3:81:3570278-3570383 Satlength=106 Nr of Repeats=7 RepeatLength=15 seed=TTCTTAGTTC Num.seqs=7 Similarity=0.873016 tpcCG=39.099998 0 TTCTTAGTTCATTAT

** * * ***

Consensus:

tTCtTTaaTCnTTA

>arqueas_representative_Fam_12_12_7 Nr. of seq. 7 Alignment length(with gaps) = 13 Alignment score = 0.636142

arqueas_representative:NC_013790.1_Methanobrevibacter_ruminantium_M1:50:2455512-2455556 Satlength=45 Nr of Repeats=4 RepeatLength=11 seed=TTGATAAAAA Num.seqs=4 Similarity=1.000000 tpcCG=32.599998 0 -TTGATAAA-AAA

arqueas_representative:NZ_CP009528.1_Methanosarcina_barkeri_MS:82:176648-176708 Satlength=61 Nr of Repeats=5 RepeatLength=12 seed=TAAATAAATT Num.seqs=5 Similarity=1.000000 tpcCG=39.168999 4 -TTGATAAATAAA

arqueas_representative:NZ_CP009520.1_Methanosarcina_vacuolata_Z-761:88:4505060-4505126 Satlength=67 Nr of Repeats=6 RepeatLength=11 seed=ATTTGATAAA Num.seqs=6 Similarity=1.000000 tpcCG=39.732498 9 -TTGATAAA-CAT

Rev.of_arqueas_representative:NC_013790.1_Methanobrevibacter_ruminantium_M1:50:957849-957957 Satlength=109 Nr of Repeats=8 RepeatLength=12 seed=TTATTTATCA Num.seqs=7 Similarity=0.936508 tpcCG=32.59999 0 ATTGATAAATAA-

arqueas_representative:NZ_CP009517.1_Methanosarcina_barkeri_3:81:1560098-1560150 Satlength=53 Nr of Repeats=4 RepeatLength=12 seed=ATAAATAGAT Num.seqs=3 Similarity=1.000000 tpcCG=39.099998 8 ATAGATAAATAA-

arqueas_representative:NZ_CP009512.1_Methanosarcina_mazei_S-6:85:2456800-2457086 Satlength=287 Nr of Repeats=8 RepeatLength=12 seed=ATTAATAAAT Num.seqs=7 Similarity=0.809524 tpcCG=41.400002 0 ATTAATAAATAA-

Rev.of_arqueas_representative:NZ_CP009515.1_Methanosarcina_lacustris_Z-7289:84:1522479-1522551 Satlength=73 Nr of Repeats=6 RepeatLength=12 seed=AAATTTAATT Num.seqs=6 Similarity=0.903704 tpcCG=41.7999 4 ATTTATAATTAA-

* **** *

Consensus:

aTTgATAAAtAA

>arqueas_representative_Fam_13_10_7 Nr. of seq. 7 Alignment length(with gaps) = 12 Alignment score = 0.666667

arqueas_representative:NZ_CP014265.1_Methanobrevibacter_olleyae__YLM1:49:198304-198464 Satlength=161 Nr of Repeats=15 RepeatLength=10 seed=TTAATTTTTA Num.seqs=14 Similarity=0.934066 tpcCG=26.900000 0 TTAATTTT--TA

arqueas_representative:NZ_CP014265.1_Methanobrevibacter_olleyae__YLM1:49:550480-550560 Satlength=81 Nr of Repeats=7 RepeatLength=10 seed=ATTTTAATTA Num.seqs=6 Similarity=0.928889 tpcCG=26.900000 3 TTAATTTT--AA

arqueas_representative:NC_015636.1_Methanothermococcus_okinawensis_IH1:95:1384814-1384962 Satlength=149 Nr of Repeats=7 RepeatLength=11 seed=ATTTTATATT Num.seqs=5 Similarity=0.903030 tpcCG=29.277700 3 TTAATTTT-ATA

Rev.of_arqueas_representative:NC_014222.1_Methanococcus_voltae_A3:65:1912269-1912338 Satlength=70 Nr of Repeats=6 RepeatLength=10 seed=AAAAAGAATA Num.seqs=5 Similarity=1.000000 tpcCG=28.600000 8 TTCTTTTT--TA

Rev.of_arqueas_representative:NZ_CP009528.1_Methanosarcina_barkeri_MS:82:3971184-3971304 Satlength=121 Nr of Repeats=11 RepeatLength=10 seed=AAATAATAAA Num.seqs=10 Similarity=0.937778 tpcCG=39.168999 16 TTATTTTT--TA

Rev.of_arqueas_representative:NZ_CP009512.1_Methanosarcina_mazei_S-6:85:3852098-3852161 Satlength=64 Nr of Repeats=6 RepeatLength=10 seed=AAATAATAAA Num.seqs=5 Similarity=0.946667 tpcCG=41.400002 16 TTATTTTT--TA

Rev.of_arqueas_representative:NC_014222.1_Methanococcus_voltae_A3:65:137658-137746 Satlength=89 Nr of Repeats=8 RepeatLength=11 seed=AAATAATAGA Num.seqs=8 Similarity=1.000000 tpcCG=28.600000 17 TTATTTTTC-TA

** **** *

Consensus:

TTAtTTTTTA

>arqueas_representative_Fam_14_10_7 Nr. of seq. 7 Alignment length(with gaps) = 12 Alignment score = 0.619048

arqueas_representative:NC_013790.1_Methanobrevibacter_ruminantium_M1:50:1478778-1479260 Satlength=483 Nr of Repeats=4 RepeatLength=10 seed=TATTTTTTTC Num.seqs=3 Similarity=1.000000 tpcCG=32.599998 0 TATTTTTTTC--

Rev.of_arqueas_representative:NZ_CP009515.1_Methanosarcina_lacustris_Z-7289:84:17737-18064 Satlength=328 Nr of Repeats=5 RepeatLength=10 seed=TAGATAAAAA Num.seqs=3 Similarity=0.911111 tpcCG=41.799999 2 TATTTTTATC--

Rev.of_arqueas_representative:NZ_CP009517.1_Methanosarcina_barkeri_3:81:2614826-2614874 Satlength=49 Nr of Repeats=4 RepeatLength=12 seed=TATAGAAAAA Num.seqs=4 Similarity=0.944444 tpcCG=39.099998 2 TATTTTTTTCTA

arqueas_representative:NZ_CP009515.1_Methanosarcina_lacustris_Z-7289:84:3844656-3844716 Satlength=61 Nr of Repeats=5 RepeatLength=12 seed=TATTTTCTTC Num.seqs=5 Similarity=0.933333 tpcCG=41.799999 12 TATTTTCTTCTA

Rev.of_arqueas_representative:NZ_CP009512.1_Methanosarcina_mazei_S-6:85:3698144-3698240 Satlength=97 Nr of Repeats=5 RepeatLength=12 seed=GAAGAAAAAA Num.seqs=4 Similarity=1.000000 tpcCG=41.400002 10 TTTTTTCTTCTA

Rev.of_arqueas_representative:NZ_CP009517.1_Methanosarcina_barkeri_3:81:2330875-2330975 Satlength=101 Nr of Repeats=9 RepeatLength=10 seed=GTAGAGAAAA Num.seqs=6 Similarity=1.000000 tpcCG=39.099998 3 TACTTTTCTC--

Rev.of_arqueas_representative:NZ_CP009515.1_Methanosarcina_lacustris_Z-7289:84:939875-939965 Satlength=91 Nr of Repeats=8 RepeatLength=10 seed=AGCAGAAAAA Num.seqs=7 Similarity=0.961905 tpcCG=41.799999 4 TGCTTTTTTC--

* *** **

Consensus:

TatTTTttTC

>arqueas_representative_Fam_15_246_6 Nr. of seq. 6 Alignment length(with gaps) = 260 Alignment score = 0.646752

arqueas_representative:NZ_CP009517.1_Methanosarcina_barkeri_3:81:2758362-2759354 Satlength=993 Nr of Repeats=4 RepeatLength=246 seed=CCAAAACTCC Num.seqs=3 Similarity=0.686508 tpcCG=39.099998 0 CCAAAACTCCATTTCCATTT-AGTNGGTGCTCCTGTGCTTGTGTCAGTAAATTTAACGTTTAATGGAACTTTTCCTGAGGTAGGAGATGC-AGAAAATGCAGCAA-CCG-G-TT------TTGTTACNACTTTTATATANTCAGTTTTTGTTA-C-ATGTTACTACCTGCTGAGTTAGTTACTG-TAAGTACAACCGTATAANTTCCGGCTTTNGAATACTTATGCGTTGGATTCTGCAN-GGTTGAAGTTGTTCCGTCT

Rev.of_arqueas_representative:NZ_CP009528.1_Methanosarcina_barkeri_MS:82:711491-712475 Satlength=985 Nr of Repeats=5 RepeatLength=246 seed=TAACAAAAAC Num.seqs=3 Similarity=0.668898 tpcCG=39.168999 143 CCAAAANTCCATTTCCATTTCAGTGGGTGTTCCTGTGCTGGTGTCAGTAAATTTAACGTTTAATGGTACTGCTCCTGAGGTTGGAGACCC-GGAGAATGCAGCAA-CAG-G-TC------TTGCTATTACTGTTATATAGTCTGTTTTTGTTANC-GTGTTACTGCCTGCAGCATTTNTTGCTG-TCAGTGTCACNGTATACCTNCCTACTTTGGAATACTTATGAATTGGATTCTGGAG-GGTTGACGTTGNTCCATCT

arqueas_representative:NZ_CP009528.1_Methanosarcina_barkeri_MS:82:523201-524194 Satlength=994 Nr of Repeats=4 RepeatLength=246 seed=AAATCCCATT Num.seqs=3 Similarity=0.788124 tpcCG=39.168999 3 CCAAAATCCCATTTCCATTT-CGTTGGTGATCCGGTACTTTTGTCNGTAAATTTAACGTTTAATGGTATTCTTCCTGAGGTNGGAGATGC-AGAGAAGGCAGCAA-CGG-G-TT------TTGATGTCACGGTTATATACTTTGATTTTGTTG-CTGTGTTGATGCCTGCNGCATTGGTTACTG-TAAGTTTAACAGTATAAACTCCTGCTTTTGAATACTTATGCGTTGGATTCTGGTG-GAATGACTTTGTTCCATCT

Rev.of_arqueas_representative:NZ_CP009528.1_Methanosarcina_barkeri_MS:82:1451926-1453441 Satlength=1516 Nr of Repeats=6 RepeatLength=246 seed=TTTACTGACA Num.seqs=4 Similarity=0.910569 tpcCG=39.168999 50 CCAAAGCTCCATTGCCATTT-CGTCGGAATTCCTGTGCTCTTGTCAGTAAATTTTACAGTTAAAGGTGTTTTTCCTGAAGTCGGAGACGC-AGAGAATGCAGCAG-TGG-G-TT------TATCTATCACTGTTATATAGTCTGTTTTTGTTA-CTGTGTTACTGCCTCTAGCATTCTTTACTGTTAAG-CTAACGGTATATTTTCCTGCTTTTGAATACTTATGCGTTGGATTCTG-AGCGAATGACCTTGACCCGTCT

Rev.of_arqueas_representative:NZ_CP009517.1_Methanosarcina_barkeri_3:81:2710419-2711676 Satlength=1258 Nr of Repeats=5 RepeatLength=255 seed=TCAGGAAATG Num.seqs=3 Similarity=0.836576 tpcCG=39.099998 77 CCGAAATCCCATTTCCATTT-AGTGGGTTTTCCTGTACTTTTGTCAGTGAATTTAACGCTTAAAGGTTCATTTCCTGATTTTGGAGATGC-ATAAAATACAGCAGTTGGAGCTTGGGAAGTTGTTGTAACGGTTATATATTTTGATTTTGTTT-CTGTACTACTGCCTGCTGCATTTGTTACGG-TAAGTTTAACAGTATATTTTCCTGCTTTGGAATACTTATGAGTTGGATTCTGCAA-GGTTGAATATGTTCCATCT

arqueas_representative:NZ_CP009528.1_Methanosarcina_barkeri_MS:82:2538010-2538994 Satlength=985 Nr of Repeats=4 RepeatLength=246 seed=TGTTTTTGTT Num.seqs=4 Similarity=0.721778 tpcCG=39.168999 131 CCAAAGTCCCATTTCCAGGA-AGTTGGAGAGCCTGTGCTTGTGTCAGTAAAGGCAACATTTAACGGCGCTTTTCCTGATGTAGCA-CTGCTGGTGAAGTTTGCAA-CAG-G-TT------TTGTTACTACTTTTATATAATCTGTTTTTGTTA-CCGTATTACGGCCTGCAACATTCTTTACTGTTAAGT-TAACAGTATATATTCCTGCCTTTGAATACTTATGAATCGGATTCTGGTG-GAATGACTTTGATCCGTCT

** ** ***** *** ** ** ** ** ** **** ** ** ** **** ** ****** * * * * ** *** * * * * * ** ******* * * ******* * * * *** ** ** * * * ** ** ***** ** * ** *********** * ******** * *** ** ** ***

Consensus:

CCAAAatcCCATTTCCATTTaGTnGGtgtTCCTGTgCTtgTGTCAGTAAATTTAACgtTTAAtGGtacTtTTCCTGAgGTnGGAGAtGCaGAgAAtgCAGCAacgGGTTTTGtTatnACtgTTATATAnTcTGtTTTTGTTaCtGTgTTACTGCCTGCaGCATTngTTACTGTAAGTnTAACaGTATAtnTTCCTGCTTTtGAATACTTATGagTTGGATTCTGgagGaaTGActTTGtTCCaTCT

>arqueas_representative_Fam_16_141_6 Nr. of seq. 6 Alignment length(with gaps) = 141 Alignment score = 0.668558

arqueas_representative:NC_003552.1_Methanosarcina_acetivorans_str._C2A:80:2510234-2510939 Satlength=706 Nr of Repeats=5 RepeatLength=141 seed=AAACTTCTGA Num.seqs=5 Similarity=0.731944 tpcCG=42.700001 0 AAACTTCTGAATGCGNTTATTNCCTGNATCNGCAACATAAACATTACCCGAAGAATCTACAGCAATACCANATGGNTAAANAAATTGTCCGTTTCCGCTGCCCNAAGAACCCCATTGAGTGAGGTATCCACCTGTGCTGTT

arqueas_representative:NZ_CP009520.1_Methanosarcina_vacuolata_Z-761:88:3877577-3878294 Satlength=718 Nr of Repeats=5 RepeatLength=141 seed=GAAGAATCTA Num.seqs=3 Similarity=0.720280 tpcCG=39.732498 49 AAACTTCTGAATTCGATTATTTATTGAATCGGCAACATAAACATTGCCCGAAGAATCTACAGCAACACCAGATGGTTCTTTAAATTGTCCGTTGCCGGTGCCATACGAACCCCATTGNGTAAGGTANNTACCGTTGCTATC

arqueas_representative:NZ_CP009520.1_Methanosarcina_vacuolata_Z-761:88:2025285-2026704 Satlength=1420 Nr of Repeats=9 RepeatLength=141 seed=CCCCATTTGG Num.seqs=6 Similarity=0.736150 tpcCG=39.732498 109 AAACTTCTGAATGCGATTATTGCCTGAATCGGCAACATAAACATTGCCAGAGGAATCTACAGCAACACCANCNGGACCATNAAATTGTCCGTCGCCGCTGCCCTCTGAACCCCATTTGGTAATAAATTTNCCACTGCTGTC

Rev.of_arqueas_representative:CP009516.1_Methanosarcina_horonobensis_HB-1:83:2480624-2481335 Satlength=712 Nr of Repeats=5 RepeatLength=141 seed=TTTATGTTGC Num.seqs=3 Similarity=0.634810 tpcCG=41.2999 41 AAATTTCAGAATGCGATCATTATCANTATCGGCAACATAAATATTACCCGAAGAATCTACAGCAATACCTGNTGGATTATTAAATTGCCCATCATCGCTGCCCTNAGAACCNAATTGTTTAAGGTAATTACCGTTGCTGTC

Rev.of_arqueas_representative:NZ_CP009506.1_Methanosarcina_siciliae_T4/M:86:504578-505283 Satlength=706 Nr of Repeats=5 RepeatLength=141 seed=TTCAGAAATT Num.seqs=5 Similarity=0.747887 tpcCG=42.900002 11 AAATTTCTGAATGCGATGATTGCCAGTATCGGCAACATAAACATTGTCCAAAGAATCAACAGTTATACCAAATGGAGAGCTAAATTCCCCAGCCCCACTGCCATAAGAACCCCATCTAGTAAGAAATTTACCACTGCTATC

arqueas_representative:CP009516.1_Methanosarcina_horonobensis_HB-1:83:4120851-4121556 Satlength=706 Nr of Repeats=4 RepeatLength=141 seed=CCAGAAGAGT Num.seqs=3 Similarity=0.766745 tpcCG=41.299999 46 AAACTTCTGAATGCGATTATTATCGGTATCGGCAACATAAACATTGCCAGAAGAGTCAACGGCAATACCGNTCGGATAANTAAAACTTCCGTTGGCGGTTCCAGAAGAACCCCAGGTAATTATGAAGTTGCCACTGCTATC

*** *** **** ** * *** *** ********** *** * * ** ** ** * * *** ** *** ** * * ** ***** * * * * ** **** *

Consensus:

AAAcTTCTGAATGCGATtATTncCtGtATCGGCAACATAAACATTgCCcGAAGAATCtACAGCAAtACCanatGGataattAAATTgtCCgTcgcCGcTGCCataaGAACCCCATtgagTaAggaAttTaCCacTGCTaTC

>arqueas_representative_Fam_17_78_6 Nr. of seq. 6 Alignment length(with gaps) = 82 Alignment score = 0.664228

arqueas_representative:NZ_CP014265.1_Methanobrevibacter_olleyae__YLM1:49:583238-583541 Satlength=304 Nr of Repeats=4 RepeatLength=75 seed=AGCACCACCA Num.seqs=3 Similarity=0.810370 tpcCG=26.900000 0 AGCACCACCATACT-C--T-GCATGATTATTGACAAAAGAACAATCCTTTAAAGTACCATTAGCACCATCCCAATAAAC---

arqueas_representative:NZ_CP014265.1_Methanobrevibacter_olleyae__YLM1:49:775259-775637 Satlength=379 Nr of Repeats=5 RepeatLength=75 seed=AGCACCACCA Num.seqs=4 Similarity=0.706871 tpcCG=26.900000 0 AGCACCACCATACT-C--T-GCATGGTTATTGACAAAAGAACAATCATTTAAAGTACCATTAGCACCATCCCAATNAAC---

arqueas_representative:NZ_CP014265.1_Methanobrevibacter_olleyae__YLM1:49:368901-369210 Satlength=310 Nr of Repeats=4 RepeatLength=78 seed=AAAAGAACAA Num.seqs=3 Similarity=0.620027 tpcCG=26.900000 33 AGCACCACCANACT-NATTAGCATGGTTATTGGCAAAAGAACAAGCACTTAAAACACCATTAGCACTATNCCAATAAAC---

arqueas_representative:NZ_CP014265.1_Methanobrevibacter_olleyae__YLM1:49:1049372-1049762 Satlength=391 Nr of Repeats=5 RepeatLength=78 seed=AAAAGAACAA Num.seqs=5 Similarity=0.617886 tpcCG=26.900000 33 AGCACCACCATNNT-NATTAGCANGATTATTAACAAAAGAACAANCACTTAAAAAACCATTAGNACCATCCCAATAGAT---

Rev.of_arqueas_representative:NZ_CP014265.1_Methanobrevibacter_olleyae__YLM1:49:742375-742918 Satlength=544 Nr of Repeats=6 RepeatLength=78 seed=TAATAACCAT Num.seqs=4 Similarity=0.651389 tpcCG=26.9000 28 ---ACCTCCATAAT-AATTAGCATGGTTATTAGTAAAAGAACAAGCACTTAAAGTACCATTATCACCCGACCAATTAACAGC

arqueas_representative:NZ_CP014265.1_Methanobrevibacter_olleyae__YLM1:49:575306-575768 Satlength=463 Nr of Repeats=6 RepeatLength=78 seed=GCACCACCAT Num.seqs=4 Similarity=0.675214 tpcCG=26.900000 1 GGCACCACCATAATACCCT-GCATGGTTGTTGGTAAAGGAAGAATCTCTTAAAATACTATTATCTCCATACCAATCAAT---

*** *** * * *** * ** ** *** *** ** * ***** ** **** * ***** *

Consensus:

aGCACCACCATAcTcatTaGCATGgTTATTgacAAAAGAACAAtCacTTAAAatACCATTAgCACCATcCCAATaAAc

>arqueas_representative_Fam_18_12_6 Nr. of seq. 6 Alignment length(with gaps) = 14 Alignment score = 0.651587

arqueas_representative:NZ_CP009515.1_Methanosarcina_lacustris_Z-7289:84:1551487-1551609 Satlength=123 Nr of Repeats=9 RepeatLength=12 seed=AAGAGGAAAA Num.seqs=8 Similarity=1.000000 tpcCG=41.799999 9 AGG-AAAAAG-AAG

arqueas_representative:NZ_CP009517.1_Methanosarcina_barkeri_3:81:1570116-1570168 Satlength=53 Nr of Repeats=4 RepeatLength=13 seed=AAAAAGAAAG Num.seqs=4 Similarity=1.000000 tpcCG=39.099998 15 AAG-AAAAAGAAAG

arqueas_representative:NZ_CP009506.1_Methanosarcina_siciliae_T4/M:86:1636904-1636976 Satlength=73 Nr of Repeats=6 RepeatLength=12 seed=GAAAAAGAAG Num.seqs=6 Similarity=0.837037 tpcCG=42.900002 14 GAG-AAAAAG-AAG

Rev.of_arqueas_representative:NZ_CP009512.1_Methanosarcina_mazei_S-6:85:2727759-2727923 Satlength=165 Nr of Repeats=10 RepeatLength=12 seed=TTATTCTTCT Num.seqs=6 Similarity=1.000000 tpcCG=41.400002 17 AAT-AAAAAG-AAG

arqueas_representative:NZ_CP009512.1_Methanosarcina_mazei_S-6:85:3896409-3896511 Satlength=103 Nr of Repeats=8 RepeatLength=12 seed=AAGTAAAAAG Num.seqs=5 Similarity=0.955556 tpcCG=41.400002 11 AAGTAAAAAG-AA-

Rev.of_arqueas_representative:NZ_CP009512.1_Methanosarcina_mazei_S-6:85:4101018-4101894 Satlength=877 Nr of Repeats=11 RepeatLength=13 seed=CTTCTTTTTA Num.seqs=10 Similarity=0.945299 tpcCG=41.400002 11 CAGTAAAAAG-AAG

** ******

Consensus:

AAGaAGAAAAAG

>arqueas_representative_Fam_19_13_6 Nr. of seq. 6 Alignment length(with gaps) = 14 Alignment score = 0.647619

arqueas_representative:NC_003552.1_Methanosarcina_acetivorans_str._C2A:80:3525175-3525331 Satlength=157 Nr of Repeats=12 RepeatLength=13 seed=TGTACTATTT Num.seqs=12 Similarity=0.881896 tpcCG=42.700001 0 TGTA-CTATTTTAA

arqueas_representative:NZ_CP009528.1_Methanosarcina_barkeri_MS:82:139520-139739 Satlength=220 Nr of Repeats=17 RepeatLength=13 seed=ATGTTCAATT Num.seqs=15 Similarity=1.000000 tpcCG=39.168999 12 TGTT-CAATTTTAA

arqueas_representative:NZ_CP009515.1_Methanosarcina_lacustris_Z-7289:84:3986319-3986428 Satlength=110 Nr of Repeats=7 RepeatLength=13 seed=AATTTATTGT Num.seqs=5 Similarity=0.958974 tpcCG=41.799999 6 TGTT-CTAATTTAT

Rev.of_arqueas_representative:NC_003552.1_Methanosarcina_acetivorans_str._C2A:80:4632526-4632653 Satlength=128 Nr of Repeats=10 RepeatLength=13 seed=GAAGATAAAA Num.seqs=7 Similarity=1.000000 tpcCG=42. 5 TCTT-CTATTTTTA

Rev.of_arqueas_representative:NZ_CP009506.1_Methanosarcina_siciliae_T4/M:86:3986697-3986801 Satlength=105 Nr of Repeats=8 RepeatLength=13 seed=AAATAGAAGA Num.seqs=8 Similarity=0.845663 tpcCG=42.900002 10 TCTT-CTATTTTAA

arqueas_representative:NZ_CP009517.1_Methanosarcina_barkeri_3:81:2580903-2580969 Satlength=67 Nr of Repeats=5 RepeatLength=13 seed=TATTTTATCT Num.seqs=4 Similarity=1.000000 tpcCG=39.099998 5 TCTTGCTATTTT-A

* * * * ***

Consensus:

TcTTCTATTTTaA

>arqueas_representative_Fam_20_12_6 Nr. of seq. 6 Alignment length(with gaps) = 13 Alignment score = 0.671795

arqueas_representative:NZ_CP009518.1_Methanococcoides_methylutens_MM1:60:902945-902989 Satlength=45 Nr of Repeats=4 RepeatLength=11 seed=CTCTTCTTCT Num.seqs=4 Similarity=1.000000 tpcCG=44.000000 0 CT-CTTC-TTCTT

Rev.of_arqueas_representative:NZ_CP011266.1_Methanobrevibacter_millerae__SM9:48:2309545-2310036 Satlength=492 Nr of Repeats=8 RepeatLength=12 seed=AGGAAGAGAA Num.seqs=5 Similarity=0.774359 tpcCG=31.79 8 CT-CTTCCTTCTT

arqueas_representative:NZ_CP009149.1_Methanocaldococcus_bathoardescens__JH146:52:1386557-1387055 Satlength=499 Nr of Repeats=5 RepeatLength=12 seed=TCTTCTTTCT Num.seqs=3 Similarity=0.675214 tpcCG=30.7 13 CT-CTTCTTTCTT

Rev.of_arqueas_representative:NC_014122.1_Methanocaldococcus_infernus_ME:54:911006-911421 Satlength=416 Nr of Repeats=5 RepeatLength=12 seed=AAGAGGAGAA Num.seqs=3 Similarity=0.743590 tpcCG=33.599998 17 CT-CTTCTTTCTT

arqueas_representative:NZ_CP009515.1_Methanosarcina_lacustris_Z-7289:84:805422-805584 Satlength=163 Nr of Repeats=13 RepeatLength=12 seed=TGCTTCTGCT Num.seqs=12 Similarity=0.962963 tpcCG=41.799999 1 CTGCTTC-TGCTT

arqueas_representative:NZ_CP009512.1_Methanosarcina_mazei_S-6:85:1212042-1212120 Satlength=79 Nr of Repeats=5 RepeatLength=12 seed=TCCTTCTCCT Num.seqs=3 Similarity=0.740741 tpcCG=41.400002 1 NTCCTTC-TCCTT

* **** * ***

Consensus:

CTCTTCnTtCTT

>arqueas_representative_Fam_21_12_6 Nr. of seq. 6 Alignment length(with gaps) = 12 Alignment score = 0.733333

arqueas_representative:NZ_CP019285.1_Halobiforma_lacisalsi_AJ5:23:2043707-2043779 Satlength=73 Nr of Repeats=6 RepeatLength=12 seed=CTCGTGTTCG Num.seqs=6 Similarity=1.000000 tpcCG=65.237297 0 CTCGTGTTCGTG

arqueas_representative:NZ_CP019893.1_Natrialbaceae_archaeon_JW/NM-HA_1:99:1588748-1588796 Satlength=49 Nr of Repeats=4 RepeatLength=12 seed=CGTGGTCGTG Num.seqs=4 Similarity=0.888889 tpcCG=64.099998 2 GTCGTGGTCGTG

Rev.of_arqueas_representative:NC_007426.1_Natronomonas_pharaonis_DSM_2160_:105:983331-983385 Satlength=55 Nr of Repeats=4 RepeatLength=12 seed=CCACGACCAC Num.seqs=3 Similarity=0.851852 tpcCG=63.080700 7 GTCGTGGTCGTG

Rev.of_arqueas_representative:NZ_CP019893.1_Natrialbaceae_archaeon_JW/NM-HA_1:99:2838909-2838963 Satlength=55 Nr of Repeats=4 RepeatLength=12 seed=CACGACCACG Num.seqs=3 Similarity=0.925926 tpcCG=64.09 6 GTCGTGATCGTG

arqueas_representative:NC_013922.1_Natrialba_magadii_ATCC_43099:100:642980-643046 Satlength=67 Nr of Repeats=5 RepeatLength=12 seed=GTTCGGGTTC Num.seqs=4 Similarity=1.000000 tpcCG=61.032001 5 TTCGGGTTCGGG

Rev.of_arqueas_representative:NC_013922.1_Natrialba_magadii_ATCC_43099:100:819180-819228 Satlength=49 Nr of Repeats=4 RepeatLength=12 seed=CGAACTCGAA Num.seqs=4 Similarity=0.870370 tpcCG=61.032001 10 TTCGTGTTCGAG

*** * *** *

Consensus:

gTCGTGtTCGtG

>arqueas_representative_Fam_22_11_6 Nr. of seq. 6 Alignment length(with gaps) = 11 Alignment score = 0.751515

arqueas_representative:NC_009634.1_Methanococcus_vannielii_SB:64:50295-50405 Satlength=111 Nr of Repeats=10 RepeatLength=10 seed=ATTTATTAAA Num.seqs=9 Similarity=1.000000 tpcCG=31.299999 5 TTAAA-ATTTA

arqueas_representative:NC_014222.1_Methanococcus_voltae_A3:65:718564-718618 Satlength=55 Nr of Repeats=4 RepeatLength=11 seed=AATTATTAAA Num.seqs=3 Similarity=1.000000 tpcCG=28.600000 5 TTAAAGAATTA

arqueas_representative:NZ_CP009515.1_Methanosarcina_lacustris_Z-7289:84:834093-834143 Satlength=51 Nr of Repeats=4 RepeatLength=10 seed=AATTATTATA Num.seqs=3 Similarity=1.000000 tpcCG=41.799999 5 TTATA-AATTA

arqueas_representative:NC_014222.1_Methanococcus_voltae_A3:65:1112528-1112572 Satlength=45 Nr of Repeats=4 RepeatLength=11 seed=AATATTAAAA Num.seqs=4 Similarity=0.919192 tpcCG=28.600000 1 AAATATTAAAA

arqueas_representative:NZ_CP009517.1_Methanosarcina_barkeri_3:81:2678687-2678775 Satlength=89 Nr of Repeats=8 RepeatLength=11 seed=TATTAAATAA Num.seqs=8 Similarity=0.818182 tpcCG=39.099998 3 AAATATTAAAT

arqueas_representative:CP009516.1_Methanosarcina_horonobensis_HB-1:83:2463204-2463709 Satlength=506 Nr of Repeats=4 RepeatLength=11 seed=ATTAAAAAAT Num.seqs=3 Similarity=1.000000 tpcCG=41.299999 4 AATTATTAAAA

*

Consensus:

aaAtAtaAaaA

>arqueas_representative_Fam_23_156_5 Nr. of seq. 5 Alignment length(with gaps) = 158 Alignment score = 0.831646

arqueas_representative:NC_015416.1_Methanothrix_soehngenii_=_Methanosaeta_concilii_GP-6:97:2055119-2056055 Satlength=937 Nr of Repeats=6 RepeatLength=156 seed=TCAGTCCAGC Num.seqs=6 Similarity=0.727569 0 TCAGTCCAGCAGACCCTCGATGGCGGGTATATCATTACGGGCTATACATNGTCCTTCGGGGCAGGCAAT-GAAGATCTATGGCTGATCAAGACCGATGAACAGGGAAACAAGCTATGGGATAGGACGTTTGGCGGAGCAGAACCTGATGTGGGCTAT-

arqueas_representative:NC_015416.1_Methanothrix_soehngenii_=_Methanosaeta_concilii_GP-6:97:2057475-2058255 Satlength=781 Nr of Repeats=5 RepeatLength=156 seed=TGGGAGAGGA Num.seqs=5 Similarity=0.759792 114 TCAGTCCAGCAGACCAGCGATGGCGGGTATATCATTACGGGCTATACAGAGTCCTTCGGGGCAGGCGAT-CNGGATCTGTGGCTGATCAAGACCGATGACCAGGGAAACAAGCTATGGGAGAGGACATTTGGCGGAGAAGAACCTGATGAGGGCTAT-

arqueas_representative:NC_015416.1_Methanothrix_soehngenii_=_Methanosaeta_concilii_GP-6:97:2059885-2060857 Satlength=973 Nr of Repeats=6 RepeatLength=156 seed=GGGTATATCA Num.seqs=4 Similarity=0.800570 24 TCAGTCCAGCAGACCCTCGATGGCGGGTATATCATTATGGGTAATACAGAGTCCTTCGGGGCAGGCAAC-AATGATCTGTGGCTGATCAAGACCGATGACCAGGGAAACAAGCTATGGGATAGGACATTTGGCGGTGCAGAATGGGATGAGGGCAAT-

arqueas_representative:NC_015416.1_Methanothrix_soehngenii_=_Methanosaeta_concilii_GP-6:97:2062438-2063374 Satlength=937 Nr of Repeats=6 RepeatLength=156 seed=TATATCATTA Num.seqs=6 Similarity=0.710007 27 TCAGTCCAGCAGACCCTCGATGGTGGCTATATCATTACGGGCGNAACATCCTCCTTCGGGGAAGGCNAG-AGAGATCTGTGGCTGATAAAGACCGATGACCAGGGAAACAAGCTATGGGAGAGGACGTTTGGCGGAGCAGAATATGATGAGGGCNA-G

arqueas_representative:NC_015416.1_Methanothrix_soehngenii_=_Methanosaeta_concilii_GP-6:97:2064652-2065588 Satlength=937 Nr of Repeats=6 RepeatLength=156 seed=TCAGTCCAGC Num.seqs=6 Similarity=0.803376 157 TCAGTCCAGCAGACCCTCGATGGCGGGTATATCATTACGGGCTCTACATACTCCTTCGGGGCAGGC-AGTAGCGATCTGTGGCTGATCAAGACCGATGACCAGGGAAACAGGCTATGGGAGAGGACGTTTGGCGGAGAAGAATATGATTAGGGCNATG

*************** ****** ** ********** *** *** ********** **** * ***** ******** *********** ********** ********* ***** ******** * **** *** **** *

Consensus:

TCAGTCCAGCAGACCctCGATGGcGGgTATATCATTAcGGGctatACAtagTCCTTCGGGGcAGGCnAnannGATCTgTGGCTGATcAAGACCGATGAcCAGGGAAACAaGCTATGGGAgAGGACgTTTGGCGGaGcAGAAtntGATgaGGGCnAt

>arqueas_representative_Fam_24_141_5 Nr. of seq. 5 Alignment length(with gaps) = 142 Alignment score = 0.677700

arqueas_representative:NC_011832.1_Methanosphaerula_palustris_E1-9c:90:1301344-1302049 Satlength=706 Nr of Repeats=5 RepeatLength=141 seed=CCAGAAGTTC Num.seqs=5 Similarity=0.735880 tpcCG=55.400002 0 CCAGAAGTTCACNTCGACCGGCGGATTCATCACCAGCTGGAACGGTTCCGCCTCGGCAGGCGGAGC-GTTCNATTATCCCGGTGGTGTCGCNGTGGACAGCGCCGGCAACGTCTACGTGGCNGATNCGNNTAACAACCAGAT

Rev.of_arqueas_representative:NC_011832.1_Methanosphaerula_palustris_E1-9c:90:1390992-1392438 Satlength=1447 Nr of Repeats=9 RepeatLength=141 seed=AACTTCTGGA Num.seqs=6 Similarity=0.617748 tpcCG=55.40 9 CCAGAAGTTCAATTCGACCGGCGGATTCCTCATGAAATGGGGCAGTNTNGGCTCAGGAGACGG-GCAGTTCAACNGGCCATCTGGTGTCGCGGTGGACAGCGCAGGCAATGTCTACGTGGTCGACTCGAATAACNNCCGGAT

arqueas_representative:NC_011832.1_Methanosphaerula_palustris_E1-9c:90:1523828-1525235 Satlength=1408 Nr of Repeats=8 RepeatLength=141 seed=TCCAGAAGTT Num.seqs=7 Similarity=0.612544 tpcCG=55.400002 140 CCAGAAGTTCACGTCGACCGGCACCTTCCTCACNCAATGGGGCAGTNNNGGCTCGGGAGACGG-GCAGTTCAACANNCCCTATGGTATCGCCGTGGACAGCGCCGGCAACGTCTACGTCACNGACNTGNGCAACAACCGGGT

arqueas_representative:NC_011832.1_Methanosphaerula_palustris_E1-9c:90:2754137-2755265 Satlength=1129 Nr of Repeats=8 RepeatLength=141 seed=ATTCATCACG Num.seqs=8 Similarity=0.683046 tpcCG=55.400002 165 CCAGAAGTTCACATCGACCGGCGAATTCATCACGAAATGGGGCAGTGAAGGTTCCGGAGACGG-GCAGTTCAACTATCCCNATGGTATCGCCGTGGACAGCGCCGGCAACGTCTACGTCATCGACACCTGGAACCACCGGGT

arqueas_representative:NC_011832.1_Methanosphaerula_palustris_E1-9c:90:2757192-2757843 Satlength=652 Nr of Repeats=5 RepeatLength=141 seed=TCCAGAAGTT Num.seqs=3 Similarity=0.546547 tpcCG=55.400002 140 CCAGAAGTTCACNTCGACCGGNACNTTCATCACGAAATGGGGGAGTTCTGGTCCNGGAAACGG-GCAGTTCANNTCCCCNGAAGGGATCGCGGTCGACAACGCCGGAAATGTCTACGTCANCGACGNAGNGAACAACCGGNT

*********** ******** *** *** *** ** * * * * *** ** **** ** ** **** ** **** *** ** ** ******** ** *** ** * *

Consensus:

CCAGAAGTTCAcnTCGACCGGcgnaTTCaTCAcgaaaTGGggcaGTnnnGgctCnGgAgaCGGGCaGTTCaactnnCCcnatGGtaTCGCnGTgGACAgCGCcGGcAAcGTCTACGTcancGAcncgnnnAACaaCCgGnT

>arqueas_representative_Fam_25_120_5 Nr. of seq. 5 Alignment length(with gaps) = 128 Alignment score = 0.693750

arqueas_representative:NC_003552.1_Methanosarcina_acetivorans_str._C2A:80:3652477-3652957 Satlength=481 Nr of Repeats=4 RepeatLength=120 seed=TTGTTGTTTA Num.seqs=4 Similarity=0.825926 tpcCG=42.700001 0 TTGTTGTTTATC-GTGTTAATTACAGTGACTGTGTTGCTGTTAACATTAGTCACATATGCCTTTTCT-CCTGC------GACTGCAATTCCAGCAGGACACTCTCCAACATTAATTGTGCCTATAACG

Rev.of_arqueas_representative:CP009516.1_Methanosarcina_horonobensis_HB-1:83:2316416-2317016 Satlength=601 Nr of Repeats=5 RepeatLength=120 seed=ATAAACAACA Num.seqs=5 Similarity=0.768044 tpcCG=41.2999 11 TTGTTGTTTATC-GTGTTAATTACAGTGACAGTGTTGCTGTTAACATTAGTCACATATGCCTTNTCT-CCTGC------NACTGCAATTCCAGCAGGATGCTCTCCAACATTAATTGTGCCTATAACG

arqueas_representative:NZ_CP009501.1_Methanosarcina_thermophila_TM-1:87:303502-304102 Satlength=601 Nr of Repeats=4 RepeatLength=120 seed=GTGTTAATTA Num.seqs=3 Similarity=0.685792 tpcCG=41.099998 12 TTGTTGTTTATN-GTGTTAATTACAGTNACAGTATTGCTGTTAGCGTTNGTCACATATGCNTTTGTT-CCTGC------AACTGCAATTCCGGCAGGATATTCTCCAACATTAATTGTGCCTATAACA

Rev.of_arqueas_representative:NZ_CP009515.1_Methanosarcina_lacustris_Z-7289:84:2602512-2603010 Satlength=499 Nr of Repeats=4 RepeatLength=126 seed=CTGGAATTGC Num.seqs=3 Similarity=0.894180 tpcCG=41.79 91 GTGTT-TGTAACTGTGTTAATCACAGAGACAGTATTGCTGTTAATATTAGTCACATATACGTATGTTCCCTTC-GGGTTGAATGCAATTCCAGCAGGATACTCTCCAACATTAACTGTGGCTATAACA

Rev.of_arqueas_representative:NZ_CP009506.1_Methanosarcina_siciliae_T4/M:86:1767461-1767965 Satlength=505 Nr of Repeats=4 RepeatLength=126 seed=AGCTACAAAC Num.seqs=4 Similarity=0.800705 tpcCG=42.90000 138 GTGTT-TGTAGCTGCGTTAATCACAGTGACGGTATTGCTGACAATATTAGTCACATACACGTATG-TCCCAGCAGGGTTCACTGCTATTCCGGAAGGATTCTGGCCAACATCAACCGTGGCTGCAACA

**** * ** * ****** **** ** ** ****** * ** ******** * * * ** * * *** ***** * **** * ******* ** *** ** ***

Consensus:

tTGTTgTtTAtcGtGTTAATtACAGtgACaGTaTTGCTGttAacaTTaGTCACATAtgCnTttgnTCCtgCnAcTGCaATTCCaGcAGGAtacTctCCAACATtAAttGTGcCTatAACa

>arqueas_representative_Fam_26_108_5 Nr. of seq. 5 Alignment length(with gaps) = 109 Alignment score = 0.735474

arqueas_representative:NZ_CP009517.1_Methanosarcina_barkeri_3:81:3216722-3217205 Satlength=484 Nr of Repeats=4 RepeatLength=108 seed=TCCATGCCTG Num.seqs=3 Similarity=0.744139 tpcCG=39.099998 0 TCCATGCCTGTTGCTTATCCGGTACTTGAGAAAATGCAGAATCAAGAGCTTTGGCAGCACTAGACCTGACATCA-TGATCCTTATCAGTAATCAGTTTAAGTAAATCAT

Rev.of_arqueas_representative:NZ_CP009520.1_Methanosarcina_vacuolata_Z-761:88:1387449-1387989 Satlength=541 Nr of Repeats=4 RepeatLength=108 seed=TGGAATGATT Num.seqs=3 Similarity=0.711519 tpcCG=39.732 4 TCCATGCCTGTTGCTTATCTGGTACTTGAAAAAATGCAGAATCAAGAGCTCTGGAAGCACTAGATCTAACATCA-TGATCTTTATCNTTNGTTAGTCTAAGTAAATCAT

arqueas_representative:NZ_CP009528.1_Methanosarcina_barkeri_MS:82:2063962-2064721 Satlength=760 Nr of Repeats=6 RepeatLength=108 seed=AGTCTATGTA Num.seqs=5 Similarity=0.798765 tpcCG=39.168999 92 TCCATGCCTGTTGTTTATCTGGCACTTGAGAAAATGCAGAACCAAGAGCNGAGGNAGCACCAGACCTCACATAACTG-TTTTCATCATTGGTGAGTCTATGTAAATCAT

Rev.of_arqueas_representative:NZ_CP009520.1_Methanosarcina_vacuolata_Z-761:88:2165164-2166676 Satlength=1513 Nr of Repeats=13 RepeatLength=108 seed=CAGATAAACA Num.seqs=10 Similarity=0.682549 tpcCG=39. 129 TCCATGCCTGTTGTTTATCTGGCACTTGAGAAAACGCAGAACCAAGAGCATTGGCAGCATTAAACCTCACATNAATG-TCTTCATCATTGGTCAGTTTAATTAAGTCAT

Rev.of_arqueas_representative:NZ_CP009520.1_Methanosarcina_vacuolata_Z-761:88:3267801-3268773 Satlength=973 Nr of Repeats=8 RepeatLength=108 seed=CAGATAAACA Num.seqs=7 Similarity=0.554581 tpcCG=39.732 21 TCCATGCCTTTTGTTTATCTGGTANTTGAGAAAACGCAGAACCAAGAGCANAAGCNGCTTTATNCCTCACATCC-CAATCTTCATCNTTGGTCAGTCTANTTAAGTCTT

********* *** ***** ** * **** **** ****** ******* * ** * ** **** * * *** * * *** ** *** ** *

Consensus:

TCCATGCCTgTTGtTTATCtGGtAcTTGAgAAAAtGCAGAAcCAAGAGCnntgGcaGCactAgacCTcACATcatgaTctTcATCatTggTcAGTcTAagTAAaTCaT

>arqueas_representative_Fam_27_37_5 Nr. of seq. 5 Alignment length(with gaps) = 37 Alignment score = 0.756757

arqueas_representative:NC_017034.1_Methanocella_conradii_HZ254:58:1012743-1012883 Satlength=141 Nr of Repeats=4 RepeatLength=35 seed=CACAGAGGTC Num.seqs=4 Similarity=1.000000 tpcCG=52.700001 0 CACAGAGGTCAC--AGAGGAATTTTACCACGGAGGTT

arqueas_representative:NC_017034.1_Methanocella_conradii_HZ254:58:1189260-1189400 Satlength=141 Nr of Repeats=4 RepeatLength=35 seed=CACAGAGGAC Num.seqs=4 Similarity=1.000000 tpcCG=52.700001 0 CACAGAGGACAC--AGAGGATATTTACCACGGAGGTT

arqueas_representative:NC_017034.1_Methanocella_conradii_HZ254:58:1553798-1554464 Satlength=667 Nr of Repeats=18 RepeatLength=37 seed=TTCACCACGG Num.seqs=18 Similarity=1.000000 tpcCG=52.700001 22 CACAGAGGTCACAGAGAGGATTTTCACCACGGAGGTT

Rev.of_arqueas_representative:NC_017034.1_Methanocella_conradii_HZ254:58:1428993-1429252 Satlength=260 Nr of Repeats=7 RepeatLength=37 seed=CTCTGTGTCC Num.seqs=7 Similarity=1.000000 tpcCG=52.700001 16 CACAAAGGACACAGAGATTATATTCACCACAGAGGTC

arqueas_representative:NC_017034.1_Methanocella_conradii_HZ254:58:1772547-1772919 Satlength=373 Nr of Repeats=10 RepeatLength=37 seed=AGGACACAGA Num.seqs=9 Similarity=1.000000 tpcCG=52.700001 42 CACAAAGGACACAGAGATCATATTCACCACAGAGGTT

**** *** *** *** * ** ***** *****

Consensus:

CACAgAGGaCACagAGAggAtaTTcACCACgGAGGTt

>arqueas_representative_Fam_28_17_5 Nr. of seq. 5 Alignment length(with gaps) = 19 Alignment score = 0.673684

arqueas_representative:NC_014222.1_Methanococcus_voltae_A3:65:363324-363440 Satlength=117 Nr of Repeats=7 RepeatLength=17 seed=AATAATAAAA Num.seqs=6 Similarity=1.000000 tpcCG=28.600000 0 AAT-AATAAAATAAAA-AG

Rev.of_arqueas_representative:NZ_CP009528.1_Methanosarcina_barkeri_MS:82:4317073-4317201 Satlength=129 Nr of Repeats=7 RepeatLength=18 seed=CGATTTTATT Num.seqs=6 Similarity=1.000000 tpcCG=39.168999 0 AAT-AACAAAATAAAATCG

arqueas_representative:NZ_CP009520.1_Methanosarcina_vacuolata_Z-761:88:179612-179684 Satlength=73 Nr of Repeats=4 RepeatLength=18 seed=ATAAAATCGA Num.seqs=4 Similarity=0.962963 tpcCG=39.732498 9 AAT-AACAAAATAAAATCG

arqueas_representative:NC_013790.1_Methanobrevibacter_ruminantium_M1:50:2475984-2476184 Satlength=201 Nr of Repeats=11 RepeatLength=18 seed=AAAAGAATAA Num.seqs=10 Similarity=0.960494 tpcCG=32.599998 13 AATAAAGAAACTAAAA-AG

arqueas_representative:NZ_CP009517.1_Methanosarcina_barkeri_3:81:4000593-4000746 Satlength=154 Nr of Repeats=8 RepeatLength=18 seed=TAAACAGAAT Num.seqs=7 Similarity=1.000000 tpcCG=39.099998 29 AATAAACAAAGTAAAC-AG

*** ** *** **** *

Consensus:

AATAAcAAAaTAAAaaG

>arqueas_representative_Fam_29_16_5 Nr. of seq. 5 Alignment length(with gaps) = 17 Alignment score = 0.603922

arqueas_representative:NZ_CP009528.1_Methanosarcina_barkeri_MS:82:2623289-2623373 Satlength=85 Nr of Repeats=6 RepeatLength=14 seed=TACCAGATTT Num.seqs=6 Similarity=0.968254 tpcCG=39.168999 0 -TACCAGATTTATTC--

arqueas_representative:NC_003552.1_Methanosarcina_acetivorans_str._C2A:80:707965-708125 Satlength=161 Nr of Repeats=10 RepeatLength=16 seed=TTACAGATTT Num.seqs=10 Similarity=1.000000 tpcCG=42.700001 0 TTA-CAGATTTATTCGA

Rev.of_arqueas_representative:NZ_CP009506.1_Methanosarcina_siciliae_T4/M:86:3506296-3506431 Satlength=136 Nr of Repeats=9 RepeatLength=15 seed=TCTGTTACAA Num.seqs=9 Similarity=0.773148 tpcCG=42.900002 7 -TAACAGAGTTATTTG-

arqueas_representative:NZ_CP009528.1_Methanosarcina_barkeri_MS:82:2637622-2637718 Satlength=97 Nr of Repeats=5 RepeatLength=16 seed=AGGTTTATTC Num.seqs=3 Similarity=0.944444 tpcCG=39.168999 4 -TAACAGGTTTATTCAG

Rev.of_arqueas_representative:NZ_CP009520.1_Methanosarcina_vacuolata_Z-761:88:2527102-2527182 Satlength=81 Nr of Repeats=5 RepeatLength=16 seed=TGAGTAAACC Num.seqs=5 Similarity=0.883333 tpcCG=39.73249 15 -TAACAGGTTTACTCAA

** *** *** *

Consensus:

TAaCAGatTTAtTcnn

>arqueas_representative_Fam_30_15_5 Nr. of seq. 5 Alignment length(with gaps) = 16 Alignment score = 0.652083

arqueas_representative:NZ_CP009517.1_Methanosarcina_barkeri_3:81:1177298-1177354 Satlength=57 Nr of Repeats=4 RepeatLength=14 seed=AGAGATAAGA Num.seqs=4 Similarity=1.000000 tpcCG=39.099998 15 A-AG-AGATAAGAAAA

arqueas_representative:NZ_CP009528.1_Methanosarcina_barkeri_MS:82:1447732-1448417 Satlength=686 Nr of Repeats=5 RepeatLength=15 seed=GAAGAAAAAG Num.seqs=4 Similarity=1.000000 tpcCG=39.168999 20 AGAG-GGAGAAGAAAA

arqueas_representative:NC_013156.1_Methanocaldococcus_fervens_AG86:53:657152-657613 Satlength=462 Nr of Repeats=4 RepeatLength=15 seed=GAGAAGAAAG Num.seqs=3 Similarity=0.881481 tpcCG=32.208801 19 A-AGAAGAGAAGAAAG

Rev.of_arqueas_representative:NC_013407.1_Methanocaldococcus_vulcanius_M7:56:1596311-1596791 Satlength=481 Nr of Repeats=6 RepeatLength=15 seed=CTTCTTCTTT Num.seqs=4 Similarity=0.881481 tpcCG=31.58620 20 A-GGAAGAGAAGAAAG

Rev.of_arqueas_representative:CP006670.1_Thermococcus_litoralis_DSM_5473:128:483194-483574 Satlength=381 Nr of Repeats=4 RepeatLength=15 seed=CCTCTTCTTC Num.seqs=3 Similarity=0.847222 tpcCG=43.099998 20 A-AGAGGAGAAGGAAG

* ** *** ** *

Consensus:

aGaaGAgAAGaAAgA

>arqueas_representative_Fam_31_15_5 Nr. of seq. 5 Alignment length(with gaps) = 16 Alignment score = 0.716667

arqueas_representative:NZ_CP009517.1_Methanosarcina_barkeri_3:81:4255130-4255340 Satlength=211 Nr of Repeats=9 RepeatLength=15 seed=CTAATCTTAA Num.seqs=6 Similarity=0.745185 tpcCG=39.099998 0 CTAATCTTAAAGGAG-

Rev.of_arqueas_representative:NZ_CP009520.1_Methanosarcina_vacuolata_Z-761:88:773152-773317 Satlength=166 Nr of Repeats=5 RepeatLength=15 seed=GCTCCTTCAA Num.seqs=3 Similarity=0.822222 tpcCG=39.732498 1 CTAATCTTGAAGGAG-

arqueas_representative:NZ_CP009520.1_Methanosarcina_vacuolata_Z-761:88:1745274-1745529 Satlength=256 Nr of Repeats=12 RepeatLength=15 seed=GCTAACCTTC Num.seqs=8 Similarity=0.723810 tpcCG=39.732498 14 CTAACCTTCAAGGAG-

arqueas_representative:NZ_CP009506.1_Methanosarcina_siciliae_T4/M:86:3553127-3553532 Satlength=406 Nr of Repeats=10 RepeatLength=15 seed=AGCTAACCTT Num.seqs=6 Similarity=0.662222 tpcCG=42.900002 13 CTAACCTTANAGAAG-

Rev.of_arqueas_representative:NZ_CP009506.1_Methanosarcina_siciliae_T4/M:86:2231255-2231360 Satlength=106 Nr of Repeats=5 RepeatLength=15 seed=CTTGAAGATT Num.seqs=3 Similarity=0.763889 tpcCG=42.900002 12 CTAATCTTCAAG-AGN

**** *** ** **

Consensus:

CTAAtCTTnaAGgAG

>arqueas_representative_Fam_32_12_5 Nr. of seq. 5 Alignment length(with gaps) = 15 Alignment score = 0.702222

arqueas_representative:NC_003552.1_Methanosarcina_acetivorans_str._C2A:80:1145905-1146337 Satlength=433 Nr of Repeats=33 RepeatLength=12 seed=GTTGAATCAT Num.seqs=31 Similarity=0.857109 tpcCG=42.700001 0 ---GTTGAATCATCA

arqueas_representative:NZ_CP009506.1_Methanosarcina_siciliae_T4/M:86:1022248-1022926 Satlength=679 Nr of Repeats=37 RepeatLength=12 seed=GTTGAATCAT Num.seqs=23 Similarity=0.893720 tpcCG=42.900002 0 ---GTTGAATCATCT

arqueas_representative:NZ_CP009506.1_Methanosarcina_siciliae_T4/M:86:1022221-1022365 Satlength=145 Nr of Repeats=12 RepeatLength=12 seed=TCTGTTGAAT Num.seqs=12 Similarity=0.878788 tpcCG=42.900002 9 ---GTTGAATCGTCT

arqueas_representative:NC_003552.1_Methanosarcina_acetivorans_str._C2A:80:38538-38628 Satlength=91 Nr of Repeats=6 RepeatLength=15 seed=CCTGTTGAAT Num.seqs=6 Similarity=0.940741 tpcCG=42.700001 0 CCTGTTGAATCATCC

arqueas_representative:NZ_CP009506.1_Methanosarcina_siciliae_T4/M:86:4948931-4949051 Satlength=121 Nr of Repeats=8 RepeatLength=15 seed=CCTGTTGAAT Num.seqs=8 Similarity=0.901587 tpcCG=42.900002 0 CCTGTTGAATCATCT

******** **

Consensus:

GTTGAATCaTCt

>arqueas_representative_Fam_33_14_5 Nr. of seq. 5 Alignment length(with gaps) = 15 Alignment score = 0.666667

arqueas_representative:NC_003552.1_Methanosarcina_acetivorans_str._C2A:80:2525427-2525492 Satlength=66 Nr of Repeats=5 RepeatLength=13 seed=GAAAGAGAGA Num.seqs=5 Similarity=1.000000 tpcCG=42.700001 11 AAGAGAGAAA-A-GA

arqueas_representative:NZ_CP009515.1_Methanosarcina_lacustris_Z-7289:84:213232-213349 Satlength=118 Nr of Repeats=9 RepeatLength=13 seed=AGAAAAAACA Num.seqs=9 Similarity=0.977208 tpcCG=41.799999 16 AAGAGAAAAA-A-CA

arqueas_representative:NZ_CP009517.1_Methanosarcina_barkeri_3:81:80563-80717 Satlength=155 Nr of Repeats=11 RepeatLength=14 seed=ATGAAAAAGA Num.seqs=11 Similarity=0.817374 tpcCG=39.099998 23 AAAAGAGANA-ATGA

Rev.of_arqueas_representative:NZ_CP009520.1_Methanosarcina_vacuolata_Z-761:88:1195711-1195837 Satlength=127 Nr of Repeats=8 RepeatLength=14 seed=TTTCATTTTC Num.seqs=7 Similarity=0.954649 tpcCG=39.7324 29 AAAAGAGAAA-ATGA

Rev.of_arqueas_representative:NZ_CP009512.1_Methanosarcina_mazei_S-6:85:2569129-2569189 Satlength=61 Nr of Repeats=4 RepeatLength=15 seed=TTATTCATTT Num.seqs=4 Similarity=1.000000 tpcCG=41.400002 31 ATAAGAGAAAAATGA

** *** * * *

Consensus:

gAAaaAGAgAaAAt

>arqueas_representative_Fam_34_14_5 Nr. of seq. 5 Alignment length(with gaps) = 15 Alignment score = 0.624444

arqueas_representative:NZ_CP009517.1_Methanosarcina_barkeri_3:81:2909771-2909827 Satlength=57 Nr of Repeats=4 RepeatLength=14 seed=CTTAAAAAAG Num.seqs=4 Similarity=0.904762 tpcCG=39.099998 0 CTTAAAAAA-GTTAC

Rev.of_arqueas_representative:NZ_CP009528.1_Methanosarcina_barkeri_MS:82:2103355-2103565 Satlength=211 Nr of Repeats=15 RepeatLength=14 seed=ACTAAGGTAA Num.seqs=15 Similarity=1.000000 tpcCG=39.168999 6 CTTAGTTAA-GTTAC

arqueas_representative:NZ_CP009520.1_Methanosarcina_vacuolata_Z-761:88:1631396-1631480 Satlength=85 Nr of Repeats=6 RepeatLength=14 seed=AAGTTACCTT Num.seqs=6 Similarity=0.917460 tpcCG=39.732498 7 CTTATTTAA-GTTAC

arqueas_representative:CP009516.1_Methanosarcina_horonobensis_HB-1:83:1818441-1818516 Satlength=76 Nr of Repeats=5 RepeatLength=15 seed=TAATCAAAGT Num.seqs=5 Similarity=1.000000 tpcCG=41.299999 2 CTTAATCAAAGTTTC

arqueas_representative:NZ_CP009501.1_Methanosarcina_thermophila_TM-1:87:2341532-2341607 Satlength=76 Nr of Repeats=5 RepeatLength=15 seed=TAATCAATGA Num.seqs=5 Similarity=1.000000 tpcCG=41.099998 2 CTTAATCAATGATCC

**** ** * * *

Consensus:

CTTAatnAAGtTaC

>arqueas_representative_Fam_35_15_5 Nr. of seq. 5 Alignment length(with gaps) = 15 Alignment score = 0.773333

arqueas_representative:NZ_CP009515.1_Methanosarcina_lacustris_Z-7289:84:3995213-3995644 Satlength=432 Nr of Repeats=30 RepeatLength=14 seed=TTAAAAAACG Num.seqs=19 Similarity=0.842885 tpcCG=41.799999 0 TTAAAAAACGATAC-

arqueas_representative:NC_003552.1_Methanosarcina_acetivorans_str._C2A:80:37272-37484 Satlength=213 Nr of Repeats=10 RepeatLength=15 seed=AAAAACGATA Num.seqs=6 Similarity=0.881481 tpcCG=42.700001 3 TTAAAAAACGATATA

Rev.of_arqueas_representative:NZ_CP009512.1_Methanosarcina_mazei_S-6:85:2331024-2331120 Satlength=97 Nr of Repeats=5 RepeatLength=14 seed=TTTTTTAGTT Num.seqs=4 Similarity=0.952381 tpcCG=41.400002 8 CTAAAAAACGATA-A

arqueas_representative:NZ_CP009506.1_Methanosarcina_siciliae_T4/M:86:4947896-4948016 Satlength=121 Nr of Repeats=6 RepeatLength=15 seed=TAAAAAACGA Num.seqs=4 Similarity=0.762963 tpcCG=42.900002 16 CTAAAAAACGATAGA

arqueas_representative:NZ_CP009512.1_Methanosarcina_mazei_S-6:85:2306749-2306808 Satlength=60 Nr of Repeats=4 RepeatLength=15 seed=AACACGATAA Num.seqs=3 Similarity=1.000000 tpcCG=41.400002 4 TTAAAACACGATAAA

***** ******

Consensus:

tTAAAAaACGATAna

>arqueas_representative_Fam_36_13_5 Nr. of seq. 5 Alignment length(with gaps) = 14 Alignment score = 0.633333

arqueas_representative:NZ_CP009528.1_Methanosarcina_barkeri_MS:82:2630470-2630548 Satlength=79 Nr of Repeats=6 RepeatLength=13 seed=ATTCTTCTTG Num.seqs=6 Similarity=1.000000 tpcCG=39.168999 0 ATTCTTCT-TGCTA

arqueas_representative:NZ_CP009515.1_Methanosarcina_lacustris_Z-7289:84:2588475-2588540 Satlength=66 Nr of Repeats=5 RepeatLength=13 seed=TTCTAATTCT Num.seqs=5 Similarity=1.000000 tpcCG=41.799999 8 ATTCTTCC-TTCTA

Rev.of_arqueas_representative:NZ_CP009520.1_Methanosarcina_vacuolata_Z-761:88:4226798-4226881 Satlength=84 Nr of Repeats=6 RepeatLength=14 seed=GAAAAATTAG Num.seqs=5 Similarity=0.885714 tpcCG=39.73249 20 ATTTTTCCTTTCTA

arqueas_representative:NZ_CP009528.1_Methanosarcina_barkeri_MS:82:3309345-3309553 Satlength=209 Nr of Repeats=16 RepeatLength=13 seed=CTTACTAACT Num.seqs=16 Similarity=0.933333 tpcCG=39.168999 6 ACTCATCT-TACTA

arqueas_representative:NZ_CP009528.1_Methanosarcina_barkeri_MS:82:3309465-3309608 Satlength=144 Nr of Repeats=11 RepeatLength=13 seed=ACTAACTCTT Num.seqs=11 Similarity=0.895571 tpcCG=39.168999 9 ACTCTTCT-TACTA

* * ** * ***

Consensus:

AtTctTCtTnCTA

>arqueas_representative_Fam_37_14_5 Nr. of seq. 5 Alignment length(with gaps) = 14 Alignment score = 0.885714

arqueas_representative:NZ_CP009517.1_Methanosarcina_barkeri_3:81:104947-105089 Satlength=143 Nr of Repeats=4 RepeatLength=14 seed=ACTCGGGTGA Num.seqs=3 Similarity=0.936508 tpcCG=39.099998 0 ACTCGGGTGACGGA

arqueas_representative:NZ_CP009520.1_Methanosarcina_vacuolata_Z-761:88:2384473-2384543 Satlength=71 Nr of Repeats=5 RepeatLength=14 seed=GACGGAACTC Num.seqs=5 Similarity=1.000000 tpcCG=39.732498 8 ACTCGGGTGACGGA

Rev.of_arqueas_representative:NZ_CP009517.1_Methanosarcina_barkeri_3:81:3266887-3266988 Satlength=102 Nr of Repeats=4 RepeatLength=14 seed=CCCGAGTTCC Num.seqs=3 Similarity=0.936508 tpcCG=39.099998 7 ACTCGGGTGATGGA

arqueas_representative:NZ_CP009528.1_Methanosarcina_barkeri_MS:82:3183646-3183716 Satlength=71 Nr of Repeats=5 RepeatLength=14 seed=GGGTGACGGA Num.seqs=5 Similarity=1.000000 tpcCG=39.168999 4 ACTAGGGTGACGGA

arqueas_representative:NZ_CP009520.1_Methanosarcina_vacuolata_Z-761:88:2294627-2294867 Satlength=241 Nr of Repeats=12 RepeatLength=14 seed=GACGGGACTC Num.seqs=11 Similarity=1.000000 tpcCG=39.732498 8 ACTCGGGTGACGGG

*** ****** **

Consensus:

ACTcGGGTGAcGGa

>arqueas_representative_Fam_38_14_5 Nr. of seq. 5 Alignment length(with gaps) = 14 Alignment score = 0.885714

arqueas_representative:NZ_CP009517.1_Methanosarcina_barkeri_3:81:1937472-1937528 Satlength=57 Nr of Repeats=4 RepeatLength=14 seed=CAACGGTTTC Num.seqs=4 Similarity=1.000000 tpcCG=39.099998 0 CAACGGTTTCGGGT

arqueas_representative:NZ_CP009517.1_Methanosarcina_barkeri_3:81:2430569-2430653 Satlength=85 Nr of Repeats=4 RepeatLength=14 seed=CAACGGTTTC Num.seqs=3 Similarity=1.000000 tpcCG=39.099998 0 CAACGGTTTCGGGT

Rev.of_arqueas_representative:NZ_CP009517.1_Methanosarcina_barkeri_3:81:3837336-3837392 Satlength=57 Nr of Repeats=4 RepeatLength=14 seed=GTTGACCCGA Num.seqs=4 Similarity=1.000000 tpcCG=39.099998 4 CAACGGTTTCGGGT

arqueas_representative:NZ_CP009517.1_Methanosarcina_barkeri_3:81:3373922-3373992 Satlength=71 Nr of Repeats=5 RepeatLength=14 seed=GGGTCAACGG Num.seqs=5 Similarity=0.942857 tpcCG=39.099998 10 CAACGGTTTAGGGT

Rev.of_arqueas_representative:NZ_CP009520.1_Methanosarcina_vacuolata_Z-761:88:1586645-1586701 Satlength=57 Nr of Repeats=4 RepeatLength=14 seed=GTTGAAACGA Num.seqs=4 Similarity=0.952381 tpcCG=39.73249 4 CAACGGTTTCGTTT

********* * *

Consensus:

CAACGGTTTcGggT

>arqueas_representative_Fam_39_13_5 Nr. of seq. 5 Alignment length(with gaps) = 13 Alignment score = 0.610256

arqueas_representative:NZ_CP009512.1_Methanosarcina_mazei_S-6:85:1888219-1888277 Satlength=59 Nr of Repeats=5 RepeatLength=11 seed=TTCTAACTTT Num.seqs=4 Similarity=1.000000 tpcCG=41.400002 0 TTCTAACTTTT--

arqueas_representative:NZ_CP009520.1_Methanosarcina_vacuolata_Z-761:88:2611411-2611476 Satlength=66 Nr of Repeats=6 RepeatLength=11 seed=TTTGTAACTT Num.seqs=5 Similarity=1.000000 tpcCG=39.732498 10 TTGTAACTTTT--

arqueas_representative:NZ_CP009501.1_Methanosarcina_thermophila_TM-1:87:494091-494175 Satlength=85 Nr of Repeats=7 RepeatLength=12 seed=TCTATTTCTC Num.seqs=7 Similarity=1.000000 tpcCG=41.099998 7 TTCTCAC-TCTAT

Rev.of_arqueas_representative:NC_003552.1_Methanosarcina_acetivorans_str._C2A:80:5189505-5189661 Satlength=157 Nr of Repeats=12 RepeatLength=13 seed=AATAGGAGTT Num.seqs=12 Similarity=0.876804 tpcCG=42 13 TTCTAACTCCTAT

Rev.of_arqueas_representative:NZ_CP009515.1_Methanosarcina_lacustris_Z-7289:84:1569353-1569457 Satlength=105 Nr of Repeats=7 RepeatLength=13 seed=ATTAGAAGTT Num.seqs=6 Similarity=0.814286 tpcCG=41.799 13 TTCTAACTTCTAA

** * ** *

Consensus:

TTcTaACttcTan

>arqueas_representative_Fam_40_12_5 Nr. of seq. 5 Alignment length(with gaps) = 13 Alignment score = 0.648718

arqueas_representative:NZ_CP009517.1_Methanosarcina_barkeri_3:81:4096-4264 Satlength=169 Nr of Repeats=14 RepeatLength=12 seed=TTCAGTTAAT Num.seqs=14 Similarity=0.959707 tpcCG=39.099998 0 TTC-AGTTAATAT

arqueas_representative:NZ_CP009517.1_Methanosarcina_barkeri_3:81:4089-4329 Satlength=241 Nr of Repeats=20 RepeatLength=12 seed=TTAATAGTTC Num.seqs=20 Similarity=0.898246 tpcCG=39.099998 5 TTC-AGTTAATAT

Rev.of_arqueas_representative:NZ_CP009517.1_Methanosarcina_barkeri_3:81:3228034-3228154 Satlength=121 Nr of Repeats=10 RepeatLength=12 seed=TTTATTAACT Num.seqs=10 Similarity=0.977778 tpcCG=39.099998 1 ATC-AGTTAATAA

Rev.of_arqueas_representative:NZ_CP009517.1_Methanosarcina_barkeri_3:81:4110814-4110874 Satlength=61 Nr of Repeats=5 RepeatLength=12 seed=TATTATCTTG Num.seqs=5 Similarity=0.933333 tpcCG=39.099998 0 TTCAAGATAATA-

Rev.of_arqueas_representative:NZ_CP009528.1_Methanosarcina_barkeri_MS:82:3874027-3874375 Satlength=349 Nr of Repeats=29 RepeatLength=12 seed=TTGAATATTA Num.seqs=29 Similarity=1.000000 tpcCG=39.168999 5 TTCAATCTAATA-

** * *****

Consensus:

tTCAgtTAATAn

>arqueas_representative_Fam_41_11_5 Nr. of seq. 5 Alignment length(with gaps) = 12 Alignment score = 0.644444

arqueas_representative:NZ_CP009520.1_Methanosarcina_vacuolata_Z-761:88:8397-8607 Satlength=211 Nr of Repeats=16 RepeatLength=10 seed=TAATTCCTTT Num.seqs=13 Similarity=0.979487 tpcCG=39.732498 22 TT-TA-ATTCCT

Rev.of_arqueas_representative:NZ_CP006933.1_Methanobacterium_formicicum__BRM9:45:2248156-2248204 Satlength=49 Nr of Repeats=4 RepeatLength=11 seed=AAAAAGAATT Num.seqs=3 Similarity=1.000000 tpcCG=41.29 22 TTTTA-ATTCTT

Rev.of_arqueas_representative:NC_015847.1_Methanococcus_maripaludis_X1:63:690772-690865 Satlength=94 Nr of Repeats=4 RepeatLength=11 seed=AATTAAAAAA Num.seqs=3 Similarity=1.000000 tpcCG=32.900002 27 TTTTA-ATTCTT

Rev.of_arqueas_representative:NZ_CP009515.1_Methanosarcina_lacustris_Z-7289:84:3141066-3141110 Satlength=45 Nr of Repeats=4 RepeatLength=11 seed=AAAAACGGAA Num.seqs=4 Similarity=1.000000 tpcCG=41.7999 17 CCGTT-TTTATT

Rev.of_arqueas_representative:NZ_CP009517.1_Methanosarcina_barkeri_3:81:831897-831969 Satlength=73 Nr of Repeats=5 RepeatLength=12 seed=AAATAACGGA Num.seqs=4 Similarity=1.000000 tpcCG=39.099998 17 CCGTTATTTATT

** * *

Consensus:

TTctTttnTaa

>arqueas_representative_Fam_42_12_5 Nr. of seq. 5 Alignment length(with gaps) = 12 Alignment score = 0.672222

arqueas_representative:NZ_CP009520.1_Methanosarcina_vacuolata_Z-761:88:2364289-2364410 Satlength=122 Nr of Repeats=11 RepeatLength=11 seed=AACTTTCAGA Num.seqs=11 Similarity=0.898623 tpcCG=39.732498 0 AACTTTCAGAT-

Rev.of_arqueas_representative:NZ_CP009517.1_Methanosarcina_barkeri_3:81:3116887-3116959 Satlength=73 Nr of Repeats=6 RepeatLength=12 seed=CTGAAACTAT Num.seqs=6 Similarity=1.000000 tpcCG=39.099998 9 TAGTTTCAGATA

Rev.of_arqueas_representative:NZ_CP009515.1_Methanosarcina_lacustris_Z-7289:84:5298-5384 Satlength=87 Nr of Repeats=6 RepeatLength=12 seed=TTAATCTCAA Num.seqs=5 Similarity=0.955556 tpcCG=41.799999 2 AAGTTTGAGATT

arqueas_representative:NC_008212.1_Haloquadratum_walsbyi_DSM_16790:31:931122-931440 Satlength=319 Nr of Repeats=4 RepeatLength=12 seed=ATTCAGATTC Num.seqs=3 Similarity=1.000000 tpcCG=47.694099 3 CAGATTCAGATT

arqueas_representative:NZ_CP009501.1_Methanosarcina_thermophila_TM-1:87:2314482-2314542 Satlength=61 Nr of Repeats=5 RepeatLength=12 seed=TTCAGATTCA Num.seqs=5 Similarity=0.888889 tpcCG=41.099998 4 CAGGTTCAGATT

* ** ****

Consensus:

nAgtTTcAGATt

>arqueas_representative_Fam_43_10_5 Nr. of seq. 5 Alignment length(with gaps) = 11 Alignment score = 0.630303

arqueas_representative:NZ_CP014265.1_Methanobrevibacter_olleyae__YLM1:49:762786-762846 Satlength=61 Nr of Repeats=5 RepeatLength=10 seed=AACTATTTTT Num.seqs=4 Similarity=0.933333 tpcCG=26.900000 4 TT-TTAACTAT

arqueas_representative:NC_014222.1_Methanococcus_voltae_A3:65:844658-844708 Satlength=51 Nr of Repeats=4 RepeatLength=10 seed=TTTTTAAGTA Num.seqs=3 Similarity=0.911111 tpcCG=28.600000 9 TT-TTAATTAT

Rev.of_arqueas_representative:NC_013790.1_Methanobrevibacter_ruminantium_M1:50:2560252-2560336 Satlength=85 Nr of Repeats=6 RepeatLength=10 seed=ATAATTAACA Num.seqs=4 Similarity=1.000000 tpcCG=32.5999 10 TG-TTAATTAT

arqueas_representative:NC_014222.1_Methanococcus_voltae_A3:65:760465-760520 Satlength=56 Nr of Repeats=5 RepeatLength=11 seed=TATTATTAAT Num.seqs=5 Similarity=0.927273 tpcCG=28.600000 5 TTAATCTATTA

arqueas_representative:NZ_CP009520.1_Methanosarcina_vacuolata_Z-761:88:1484788-1484876 Satlength=89 Nr of Repeats=8 RepeatLength=11 seed=TTTTAATCTG Num.seqs=8 Similarity=0.948052 tpcCG=39.732498 8 TTAATCTGTTT

* * *

Consensus:

tTaanTatTt

>arqueas_representative_Fam_44_255_4 Nr. of seq. 4 Alignment length(with gaps) = 260 Alignment score = 0.755769

arqueas_representative:NZ_CP009517.1_Methanosarcina_barkeri_3:81:2095253-2096558 Satlength=1306 Nr of Repeats=4 RepeatLength=255 seed=CCTGTACTCT Num.seqs=3 Similarity=0.595807 tpcCG=39.099998 0 CCTGTACTCTGGCCANTAAAACTAACAGTAAGAGGCGATTTTCCTGAAGTTGGGGACGCAGAGAAANCTGGG-ACAGGNAGATTTAAATCGTTTGAGACAGNAATATAGCCGGTTTTTGTTAATGCANTACTGCCATTT-CATNACTTACTGTCAATGTAACAGAATATANTCCTGCTTTGCTGTACGTGTGCACAGGATTCTTTTCTGTTGAA-TACGTTCCGT-CTCCAAAANTCCATTTCGTTGAGGTTGGTGAT--

arqueas_representative:NC_003552.1_Methanosarcina_acetivorans_str._C2A:80:2347432-2349247 Satlength=1816 Nr of Repeats=7 RepeatLength=255 seed=TCTCCGAAAG Num.seqs=5 Similarity=0.606178 tpcCG=42.700001 222 CCTGTNCTCTGGTCAGTAAAACNAACAGTAAGAGGCGTNTTTCCTGAAGTCTGAGATGCAGAG-AAGCTGGNAACAGGNCCATCTAAAACGTTTGAGACAGCAATATAGCCGGATTTTGTCAANGNATTACTGCCGTTTGCATTNCTTGCNGTCAATGTAACAGAATAANTTCCTGATTTATTGTANGTGTGTACGGGATTCTTNTCTGTTGAANTAT-TCCCG-TCTCCGAAAGACCATTTCCATGAANTTGGAGAN--

Rev.of_arqueas_representative:CP009516.1_Methanosarcina_horonobensis_HB-1:83:4033713-4036026 Satlength=2314 Nr of Repeats=8 RepeatLength=255 seed=TTTACTGACC Num.seqs=5 Similarity=0.663878 tpcCG=41.299 274 CCTGTGCTCTGGTCAGTAAAACTAACNGTAAGAGGTGCTTTTCCTGAAGTAGGAGATGCAGAA-AANTTGGTAACAGGAGCATCTAAAACGTTTGAGACAGCAATATAACCGGATTTTGTCAACGCATTACTNCCGTTTTCATTACTTGCTGTCAATGTAACAGAGTACNGTCCTGANTTATTGTATATATGTACAGGATTTTTTTCTGTTGAATTAC-TTCCAT-CTCCGAAAGTCCATTTCCATGCAGTTGGNGAT--

Rev.of_arqueas_representative:NZ_CP009501.1_Methanosarcina_thermophila_TM-1:87:1491871-1493674 Satlength=1804 Nr of Repeats=8 RepeatLength=255 seed=TCATGGAGAT Num.seqs=5 Similarity=0.708949 tpcCG=41.0 499 CCTGTGCTCTGGTCAGTAAAACTAACAGTAAGAGGTGCTTTTCCTGAAGCNGGAGATGCAGAG-AATCTGGNAACAGGAGCATCTAGAACGTTTGAAACAGCAATATATCTGGATTTTGTCAATGCGTTACTGCCGTTTGCATTACTTGCTGTCAATGTAACAGAGTATAGTCCTGGTTTATTGTATGTGTGTACAGGATTTCTTTCTGTTGAANTAT-TTCCAT-CTCCAAAATTCCATCTCCATGANGTTGG--ATCC

***** ****** ** ****** *** ******** * ********** * ** ***** ** *** ***** ** ** * ******* **** ****** * ** ****** ** * **** ** *** *** *** * ************** ** ***** ** **** * ** ** ***** * ********* ** * ** **** *** **** ** ** **** *

Consensus:

CCTGTgCTCTGGtCAgTAAAACtAACaGTAAGAGGcGctTTTCCTGAAGtngGaGAtGCAGAgAAncTGGnaACAGGagcATcTAaAaCGTTTGAgACAGcAATATAgCcGGaTTTTGTcAAtGcatTACTgCCgTTTgCATtaCTTgCtGTCAATGTAACAGAaTAtagTCCTGatTTatTGTAtgTgTGtACaGGATTctTtTCTGTTGAAnTAcTtCCatCTCCaAAAgtCCATtTCcaTGaagTTGGngAt

>arqueas_representative_Fam_45_123_4 Nr. of seq. 4 Alignment length(with gaps) = 124 Alignment score = 0.768817

arqueas_representative:NC_003552.1_Methanosarcina_acetivorans_str._C2A:80:3209329-3209818 Satlength=490 Nr of Repeats=4 RepeatLength=123 seed=AAAAAGTAGC Num.seqs=3 Similarity=0.626422 tpcCG=42.700001 0 AAAAAGTAGCAACCGCGAGTGCTGACAAAACAGCACGTTTATGGGATGTATCTACAGGTAAAGAAATTGCTGTTCTGAACCACAANNATTCTGTTNAAAAAT--TNTATTCAGCCCTGATGGAA

arqueas_representative:NC_003552.1_Methanosarcina_acetivorans_str._C2A:80:3137895-3139737 Satlength=1843 Nr of Repeats=15 RepeatLength=123 seed=CTGATGGAAA Num.seqs=14 Similarity=0.749578 tpcCG=42.7000 114 AATATATTGCTACGGCGAGCGATGACAATACATCACGTTTATGGGATACANCTACAGGTAAANAAATCTTTGTTCTGAACCATGATGGTNC-GGTAAATAATGTTGTATTCAGTCCTGATGGAA

Rev.of_arqueas_representative:NZ_CP009528.1_Methanosarcina_barkeri_MS:82:2243257-2245111 Satlength=1855 Nr of Repeats=14 RepeatLength=123 seed=TATTTTCCAT Num.seqs=10 Similarity=0.744474 tpcCG=39.16899 127 AATACATTGCAACGGCGAGTGATGACAATACAGCACGTTTATGGAATGTATCTACAGGTAAACAAATTTTTGTTCTGAACCATGCTGGTNG-GGTAAATAGTGTTGTTTTCAGTCCTGATGGAA

arqueas_representative:NZ_CP009528.1_Methanosarcina_barkeri_MS:82:2957391-2958990 Satlength=1600 Nr of Repeats=13 RepeatLength=123 seed=GATGGAAAAT Num.seqs=10 Similarity=0.740801 tpcCG=39.168999 239 AATACATNGCTACGGCGAGTNATGACAATACAGCACGCTTATGGAACGCATCTACAGGTAAACAAATTTTTGTTCTGAACCATGATGGTTG-GGTAAATAATGTTGTGTTCAGTCCTGATGGAA

** * * ** ** ***** ****** *** **** ****** * * *********** **** ************ * * * ** * * * * ***** **********

Consensus:

AAtAcaTtGCaACgGCGAGtgaTGACAAtACAgCACGtTTATGGaAtgcAtCTACAGGTAAAcAAATtttTGTTCTGAACCAtgatggTtcGgTaAAtAaTgtTgTaTTCAGtCCTGATGGAA

>arqueas_representative_Fam_46_102_4 Nr. of seq. 4 Alignment length(with gaps) = 103 Alignment score = 0.779935

arqueas_representative:NC_014408.1_Methanothermobacter_marburgensis_str._Marburg:92:1026710-1027322 Satlength=613 Nr of Repeats=4 RepeatLength=102 seed=TTTTTCATAG Num.seqs=3 Similarity=0.803668 tpcCG= 0 TTTTTCATAGCATTCTAGTGCCTCATCATATCTCCCAAGTTCTTTGAGGACTAGTCCTTTATTGTTCCATGCTTCTG-CAAGTTTTGGATTTATTTTTAGTGC

arqueas_representative:NC_000916.1_Methanothermobacter_thermautotrophicus_str._Delta_H:93:51420-52339 Satlength=920 Nr of Repeats=5 RepeatLength=102 seed=TTTTTCATAG Num.seqs=3 Similarity=0.812298 tpcC 0 TTTTTCATAGCATTCTAGTGCCTCATCATATCTCCCAAGTTCTTTGAGGACTAGTCCTTTGTTGTTCCATGCTTCTG-CAAGTTTTGGATTTATTTTTAGTGC

Rev.of_arqueas_representative:NC_014408.1_Methanothermobacter_marburgensis_str._Marburg:92:1466890-1467400 Satlength=511 Nr of Repeats=4 RepeatLength=102 seed=CATGGAACAA Num.seqs=3 Similarity=0.630658 71 CCTTTCATAGCATTCCAATGCCTCATCATATCTCTTAAGTTCTTCAAGGANTACTCCTTTGTTGTTCCATG-TTCCATCGTCTTCTGGATCTATTTGTAGTGC

arqueas_representative:NC_000916.1_Methanothermobacter_thermautotrophicus_str._Delta_H:93:43605-44115 Satlength=511 Nr of Repeats=5 RepeatLength=102 seed=CATAGCATTC Num.seqs=5 Similarity=0.581132 tpcC 107 TTTTTCATAGCATTCCAATGCCTCNTCATATTTCCGAANTGCGTCAAGGANTACTCCTTTGTTGTTCCATGCTTCTA-CGTNTTNTGGGTTTATTTGTAATGC

************* * ****** ****** ** ** * * * **** ** ****** ********** *** * ** *** * ***** ** ***

Consensus:

ttTTTCATAGCATTCcAaTGCCTCaTCATATcTCccAAgTtCtTcaAGGAcTAcTCCTTTgTTGTTCCATGcTTCtaCaagTTtTGGaTtTATTTgTAgTGC

>arqueas_representative_Fam_47_69_4 Nr. of seq. 4 Alignment length(with gaps) = 70 Alignment score = 0.722619

arqueas_representative:NZ_CP009528.1_Methanosarcina_barkeri_MS:82:2919660-2920419 Satlength=760 Nr of Repeats=11 RepeatLength=69 seed=TAAGATTTTT Num.seqs=11 Similarity=0.710713 tpcCG=39.168999 0 TAAGATTTTTCAACTCTGTGATTTCCGGNGGCAACGAAGTCAATTGATTNCNAGATATGTNAAGTGTAG-

Rev.of_arqueas_representative:NZ_CP009520.1_Methanosarcina_vacuolata_Z-761:88:2998463-2999153 Satlength=691 Nr of Repeats=10 RepeatLength=69 seed=AAAAATCTTA Num.seqs=10 Similarity=0.748363 tpcCG=39.73 10 TAAGATTTTTCAGTTCTGTGATTTCCGGAGGCAGCGAAGTCAATTGATTNCAAGATATGTTAAGTGTAG-

arqueas_representative:NZ_CP009515.1_Methanosarcina_lacustris_Z-7289:84:2823914-2824535 Satlength=622 Nr of Repeats=9 RepeatLength=69 seed=AGATTTTTCA Num.seqs=9 Similarity=0.706834 tpcCG=41.799999 2 TAAGATTTTTCAACTCAGAGATTTCAGGTGGCAGTGAAGTCAGTNGATTGTAAGATATGTTGAGTTGAG-

Rev.of_arqueas_representative:NZ_CP009506.1_Methanosarcina_siciliae_T4/M:86:2277778-2278330 Satlength=553 Nr of Repeats=10 RepeatLength=69 seed=CTCCTGAAAT Num.seqs=6 Similarity=0.616813 tpcCG=42.90000 30 CAAGATTTTTTAACTCTGATATTTCAGGAGGAAGCGAACTCAATTGATTACTAGA-ANATTAAGTTGAGT

********* * ** * ***** ** ** * *** *** * **** *** * * *** **

Consensus:

tAAGATTTTTcAacTCtGagATTTCaGGaGGcAgcGAAgTCAaTtGATTncaAGAtAtgTtaAGTggAG

>arqueas_representative_Fam_48_45_4 Nr. of seq. 4 Alignment length(with gaps) = 47 Alignment score = 0.725768

arqueas_representative:NC_009051.1_Methanoculleus_marisnigri_JR1:68:1229423-1229775 Satlength=353 Nr of Repeats=8 RepeatLength=44 seed=GGCGACGGGC Num.seqs=8 Similarity=1.000000 tpcCG=62.099998 0 GGCGACGGGCCG-AAGGGCCGGAGTTTGAGCGCCG-CAGGTGCGA-T

arqueas_representative:NC_009051.1_Methanoculleus_marisnigri_JR1:68:1923557-1923962 Satlength=406 Nr of Repeats=9 RepeatLength=45 seed=GCGACGGGCC Num.seqs=9 Similarity=1.000000 tpcCG=62.099998 1 GGCGACGGGCCG-AAGGGCCGGAGCTGGAGCACCG-CAGGTGCGACT

Rev.of_arqueas_representative:NC_009051.1_Methanoculleus_marisnigri_JR1:68:1429793-1430393 Satlength=601 Nr of Repeats=13 RepeatLength=46 seed=ACCTGACGGT Num.seqs=11 Similarity=0.978920 tpcCG=62.09999 40 TGCGACGGACCG-AAGGACCGGAGTTTGAGCACCGTCAGGTGCGAAG

arqueas_representative:NC_009051.1_Methanoculleus_marisnigri_JR1:68:307418-307694 Satlength=277 Nr of Repeats=6 RepeatLength=46 seed=GTGTGGGTTG Num.seqs=6 Similarity=1.000000 tpcCG=62.099998 38 TGCGACGGTCCGTTAGGACCGGAGTTTGAGCACCG-TAGGTGTGGGT

******* *** *** ****** * **** *** ***** *

Consensus:

gGCGACGGgCCGaAGGaCCGGAGtTtGAGCaCCGcAGGTGcGant

>arqueas_representative_Fam_49_27_4 Nr. of seq. 4 Alignment length(with gaps) = 30 Alignment score = 0.642593

arqueas_representative:NZ_CP009517.1_Methanosarcina_barkeri_3:81:1814928-1815095 Satlength=168 Nr of Repeats=4 RepeatLength=27 seed=AAGCCTTTTT Num.seqs=3 Similarity=0.934156 tpcCG=39.099998 0 AAG-CCTTT-TTAAAAAA-GGGTTGCGGTT

arqueas_representative:NZ_CP009520.1_Methanosarcina_vacuolata_Z-761:88:3536542-3536785 Satlength=244 Nr of Repeats=9 RepeatLength=27 seed=CAAGCCTTTT Num.seqs=9 Similarity=0.969822 tpcCG=39.732498 26 AAG-CCTTT-TTTGAAAA-GGGTTGCGGTC

arqueas_representative:NZ_CP009517.1_Methanosarcina_barkeri_3:81:943271-943663 Satlength=393 Nr of Repeats=5 RepeatLength=28 seed=TTTTGAAAAG Num.seqs=3 Similarity=0.968254 tpcCG=39.099998 8 AAG-CAGTTTTTTGAAAA-GGCTTGCGGGC

arqueas_representative:NZ_CP009520.1_Methanosarcina_vacuolata_Z-761:88:2274065-2274229 Satlength=165 Nr of Repeats=6 RepeatLength=27 seed=TTTTTGAAAA Num.seqs=4 Similarity=0.765873 tpcCG=39.732498 33 AAGACATTT--TTGAAAAGGGTTTGCGGGC

*** * ** * **** ** ******

Consensus:

AAGCatTTtTtgAAAAGGgTTGCGGgc

>arqueas_representative_Fam_50_18_4 Nr. of seq. 4 Alignment length(with gaps) = 20 Alignment score = 0.627778

arqueas_representative:NC_003552.1_Methanosarcina_acetivorans_str._C2A:80:2212663-2212807 Satlength=145 Nr of Repeats=8 RepeatLength=18 seed=CTAAAAAATA Num.seqs=8 Similarity=0.939153 tpcCG=42.700001 0 CTAAAAAA-TAA-AAAACGG

Rev.of_arqueas_representative:NZ_CP009506.1_Methanosarcina_siciliae_T4/M:86:2753938-2754111 Satlength=174 Nr of Repeats=9 RepeatLength=19 seed=TAGCCATTTT Num.seqs=7 Similarity=0.926483 tpcCG=42.900002 3 CTAAAAAACCAA-AAAATGG

arqueas_representative:NZ_CP009512.1_Methanosarcina_mazei_S-6:85:703615-703705 Satlength=91 Nr of Repeats=5 RepeatLength=18 seed=AAAACAGTTA Num.seqs=5 Similarity=0.955556 tpcCG=41.400002 11 TTAAGAAA-TAA-AAAACAG

Rev.of_arqueas_representative:NZ_CP009517.1_Methanosarcina_barkeri_3:81:3717330-3717465 Satlength=136 Nr of Repeats=5 RepeatLength=19 seed=TTATCTGTTT Num.seqs=3 Similarity=1.000000 tpcCG=39.099998 22 ATAAGAAA-CAAAAAAACAG

*** *** ** **** *

Consensus:

cTAAaAAAcAAAAAAcaG

>arqueas_representative_Fam_51_20_4 Nr. of seq. 4 Alignment length(with gaps) = 20 Alignment score = 0.613889

arqueas_representative:NZ_CP009517.1_Methanosarcina_barkeri_3:81:1647266-1647341 Satlength=76 Nr of Repeats=4 RepeatLength=19 seed=AAACCAAAAA Num.seqs=3 Similarity=1.000000 tpcCG=39.099998 0 AAACCAAA-AAGAATAAAAT

arqueas_representative:NZ_CP009520.1_Methanosarcina_vacuolata_Z-761:88:1438816-1438930 Satlength=115 Nr of Repeats=6 RepeatLength=19 seed=AAATCCACAA Num.seqs=6 Similarity=0.891667 tpcCG=39.732498 15 CCACAAAA-AAGAGAAAAAT

Rev.of_arqueas_representative:NZ_CP009528.1_Methanosarcina_barkeri_MS:82:3371986-3372086 Satlength=101 Nr of Repeats=5 RepeatLength=20 seed=TTTTTACTTC Num.seqs=5 Similarity=1.000000 tpcCG=39.168999 18 ATACAAAAGAAGTAAAAAAT

Rev.of_arqueas_representative:NZ_CP009506.1_Methanosarcina_siciliae_T4/M:86:3596366-3596486 Satlength=121 Nr of Repeats=6 RepeatLength=20 seed=ATTTGATTCT Num.seqs=6 Similarity=1.000000 tpcCG=42.900002 20 ATACATAATAAGAATCAAAT

** ** *** ****

Consensus:

atACaaAAnAAGaaaaAAAT

>arqueas_representative_Fam_52_16_4 Nr. of seq. 4 Alignment length(with gaps) = 18 Alignment score = 0.643519

arqueas_representative:NZ_CP014265.1_Methanobrevibacter_olleyae__YLM1:49:1090530-1090595 Satlength=66 Nr of Repeats=4 RepeatLength=16 seed=TTTAATAAAT Num.seqs=3 Similarity=0.777778 tpcCG=26.900000 0 TTTA-ATAAATATAANT-

Rev.of_arqueas_representative:NZ_CP009515.1_Methanosarcina_lacustris_Z-7289:84:2958588-2958656 Satlength=69 Nr of Repeats=4 RepeatLength=17 seed=TTAAGTACTT Num.seqs=4 Similarity=0.921569 tpcCG=41.7999 5 CTTA-ATAAATATAAGTA

Rev.of_arqueas_representative:NC_013790.1_Methanobrevibacter_ruminantium_M1:50:505660-505737 Satlength=78 Nr of Repeats=5 RepeatLength=16 seed=AATTATATTT Num.seqs=3 Similarity=0.888889 tpcCG=32.599998 0 TTTA-GAAAATATAATT-

Rev.of_arqueas_representative:NZ_CP014265.1_Methanobrevibacter_olleyae__YLM1:49:717371-717440 Satlength=70 Nr of Repeats=4 RepeatLength=17 seed=ATATATATTT Num.seqs=3 Similarity=0.843137 tpcCG=26.90000 0 TTTATATAAATATATAT-

*** ******* *

Consensus:

tTTAatAAATATAanT

>arqueas_representative_Fam_53_16_4 Nr. of seq. 4 Alignment length(with gaps) = 18 Alignment score = 0.646605

arqueas_representative:NZ_CP009517.1_Methanosarcina_barkeri_3:81:403369-403479 Satlength=111 Nr of Repeats=5 RepeatLength=16 seed=TTCATTGCTC Num.seqs=4 Similarity=0.902778 tpcCG=39.099998 0 TTCATTGCTCTTT-AAT-

Rev.of_arqueas_representative:NC_003552.1_Methanosarcina_acetivorans_str._C2A:80:3629593-3629677 Satlength=85 Nr of Repeats=4 RepeatLength=17 seed=TAAAAAGCAA Num.seqs=3 Similarity=1.000000 tpcCG=42.70 14 TTCATTGCTTTTT-AACA

arqueas_representative:NZ_CP009528.1_Methanosarcina_barkeri_MS:82:3010559-3010624 Satlength=66 Nr of Repeats=4 RepeatLength=16 seed=ATAATTGCTT Num.seqs=3 Similarity=1.000000 tpcCG=39.168999 0 ATAATTGCTTTTT-AAC-

arqueas_representative:NZ_CP009528.1_Methanosarcina_barkeri_MS:82:3010598-3010733 Satlength=136 Nr of Repeats=8 RepeatLength=17 seed=CTTTTTCAAC Num.seqs=7 Similarity=0.955182 tpcCG=39.168999 7 ATAATTACTTTTTCAAC-

* *** ** *** **

Consensus:

aTaATTgCTtTTTAAc

>arqueas_representative_Fam_54_18_4 Nr. of seq. 4 Alignment length(with gaps) = 18 Alignment score = 0.654321

arqueas_representative:NC_013202.1_Halomicrobium_mukohataei_DSM_12286:28:1242296-1243047 Satlength=752 Nr of Repeats=8 RepeatLength=18 seed=CGGCGTGTCG Num.seqs=5 Similarity=0.859259 tpcCG=65.506798 0 CGGCGTGTCGGTGGGCGT

Rev.of_arqueas_representative:NZ_CP019893.1_Natrialbaceae_archaeon_JW/NM-HA_1:99:745951-746071 Satlength=121 Nr of Repeats=6 RepeatLength=18 seed=CCACGGACAC Num.seqs=5 Similarity=1.000000 tpcCG=64.099 14 CGTCGTGTCCGTGGCCGT

Rev.of_arqueas_representative:NC_021921.1_Halorhabdus_tiamatea_SARL4B_:32:2135635-2135731 Satlength=97 Nr of Repeats=4 RepeatLength=18 seed=ACCGAGACGC Num.seqs=3 Similarity=1.000000 tpcCG=62.770000 12 CGGCGTCTCGGTTTCCGT

Rev.of_arqueas_representative:NC_009051.1_Methanoculleus_marisnigri_JR1:68:1187136-1187370 Satlength=235 Nr of Repeats=13 RepeatLength=18 seed=GACCCCGATG Num.seqs=13 Similarity=0.876518 tpcCG=62.09999 25 CGGGGTCTCCGTTTCCAT

** ** ** ** * *

Consensus:

CGgcGTcTCcGTggcCgT

>arqueas_representative_Fam_55_16_4 Nr. of seq. 4 Alignment length(with gaps) = 17 Alignment score = 0.699346

arqueas_representative:NC_007681.1_Methanosphaera_stadtmanae_DSM_3091:89:1534739-1534997 Satlength=259 Nr of Repeats=10 RepeatLength=14 seed=AAAATAAAAA Num.seqs=9 Similarity=1.000000 tpcCG=27.600000 2 TA-AAAATAA-AAAT-C

arqueas_representative:NC_014222.1_Methanococcus_voltae_A3:65:876892-876952 Satlength=61 Nr of Repeats=4 RepeatLength=15 seed=AAAAATAAAA Num.seqs=4 Similarity=1.000000 tpcCG=28.600000 2 T-AAAAATAA-AAATAC

Rev.of_arqueas_representative:NZ_CP009520.1_Methanosarcina_vacuolata_Z-761:88:3292096-3292407 Satlength=312 Nr of Repeats=7 RepeatLength=16 seed=TTTTTATTTT Num.seqs=5 Similarity=1.000000 tpcCG=39.7324 13 TAAAAAATAA-AAATTC

Rev.of_arqueas_representative:NZ_CP009520.1_Methanosarcina_vacuolata_Z-761:88:2722986-2723162 Satlength=177 Nr of Repeats=11 RepeatLength=16 seed=TTTTCAGGAT Num.seqs=11 Similarity=1.000000 tpcCG=39.73 6 TG-AAAATAACAAATCC

******* **** **

Consensus:

aAAAATAAAAATnCTa

>arqueas_representative_Fam_56_16_4 Nr. of seq. 4 Alignment length(with gaps) = 17 Alignment score = 0.655229

arqueas_representative:NC_003552.1_Methanosarcina_acetivorans_str._C2A:80:151908-151983 Satlength=76 Nr of Repeats=5 RepeatLength=15 seed=TGATAAATGA Num.seqs=5 Similarity=1.000000 tpcCG=42.700001 0 TGATAAATGAATGTT--

arqueas_representative:NC_003552.1_Methanosarcina_acetivorans_str._C2A:80:151920-152100 Satlength=181 Nr of Repeats=10 RepeatLength=15 seed=GTTTGATAAA Num.seqs=9 Similarity=0.876543 tpcCG=42.700001 12 TGATAAATGAANGTT--

arqueas_representative:NZ_CP009517.1_Methanosarcina_barkeri_3:81:141610-141678 Satlength=69 Nr of Repeats=4 RepeatLength=17 seed=ATGTCGATAA Num.seqs=4 Similarity=1.000000 tpcCG=39.099998 13 CGATAAATGAATGATGT

Rev.of_arqueas_representative:NZ_CP009520.1_Methanosarcina_vacuolata_Z-761:88:950550-950678 Satlength=129 Nr of Repeats=8 RepeatLength=16 seed=TCTCACATAC Num.seqs=8 Similarity=0.979167 tpcCG=39.732498 5 TGAGAAATGAAGTATG-

** ******* *

Consensus:

tGAtAAATGAAtgaTg

>arqueas_representative_Fam_57_15_4 Nr. of seq. 4 Alignment length(with gaps) = 17 Alignment score = 0.684641

arqueas_representative:CP009516.1_Methanosarcina_horonobensis_HB-1:83:3544304-3544379 Satlength=76 Nr of Repeats=5 RepeatLength=15 seed=CAAGAATTAA Num.seqs=5 Similarity=0.964444 tpcCG=41.299999 0 CAAGAATT-AAAGAAA-

arqueas_representative:NZ_CP009520.1_Methanosarcina_vacuolata_Z-761:88:2331609-2331819 Satlength=211 Nr of Repeats=12 RepeatLength=15 seed=AATTAAAGAA Num.seqs=10 Similarity=0.982222 tpcCG=39.732498 4 CTAGAATT-AAAGAAA-

arqueas_representative:NZ_CP009515.1_Methanosarcina_lacustris_Z-7289:84:2091146-2091204 Satlength=59 Nr of Repeats=4 RepeatLength=15 seed=AAACAGAATT Num.seqs=3 Similarity=1.000000 tpcCG=41.799999 12 C-AGAATTCAAAGAAA-

arqueas_representative:NZ_CP009528.1_Methanosarcina_barkeri_MS:82:3455673-3455737 Satlength=65 Nr of Repeats=4 RepeatLength=16 seed=TTAGAACTAA Num.seqs=4 Similarity=1.000000 tpcCG=39.168999 0 TTAGAACT-AAAGAAAT

**** * *******

Consensus:

ctAGAAtTAAAGAAA

>arqueas_representative_Fam_58_16_4 Nr. of seq. 4 Alignment length(with gaps) = 17 Alignment score = 0.673203

arqueas_representative:NZ_CP009515.1_Methanosarcina_lacustris_Z-7289:84:917944-918094 Satlength=151 Nr of Repeats=10 RepeatLength=15 seed=GATTCAATTT Num.seqs=10 Similarity=0.895309 tpcCG=41.799999 0 GATTCAATTTCAT-AA-

Rev.of_arqueas_representative:NZ_CP009506.1_Methanosarcina_siciliae_T4/M:86:240824-240904 Satlength=81 Nr of Repeats=5 RepeatLength=16 seed=CAGGAAATTG Num.seqs=5 Similarity=1.000000 tpcCG=42.900002 14 GATTCAATTTCCTGAA-

Rev.of_arqueas_representative:NZ_CP009517.1_Methanosarcina_barkeri_3:81:1972197-1972277 Satlength=81 Nr of Repeats=5 RepeatLength=16 seed=AATCAATATG Num.seqs=5 Similarity=0.900000 tpcCG=39.099998 4 GATTCAATTCCAT-ATT

Rev.of_arqueas_representative:NZ_CP009512.1_Methanosarcina_mazei_S-6:85:3104338-3104498 Satlength=161 Nr of Repeats=10 RepeatLength=16 seed=GAATTGATAT Num.seqs=10 Similarity=1.000000 tpcCG=41.400002 5 AATTCAATTTCAT-ATC

******** * * *

Consensus:

gATTCAATTtCaTAan

>arqueas_representative_Fam_59_15_4 Nr. of seq. 4 Alignment length(with gaps) = 17 Alignment score = 0.660131

arqueas_representative:NZ_CP009515.1_Methanosarcina_lacustris_Z-7289:84:3974788-3974848 Satlength=61 Nr of Repeats=4 RepeatLength=15 seed=AAAATAACTG Num.seqs=4 Similarity=0.896296 tpcCG=41.799999 0 AAAAT-AA-CTGGGACA

Rev.of_arqueas_representative:NZ_CP009515.1_Methanosarcina_lacustris_Z-7289:84:4131450-4131555 Satlength=106 Nr of Repeats=7 RepeatLength=15 seed=CAGTTATTTT Num.seqs=7 Similarity=0.877249 tpcCG=41.799 10 AAAAT-AA-CTGGGACA

arqueas_representative:NZ_CP009515.1_Methanosarcina_lacustris_Z-7289:84:3974796-3974916 Satlength=121 Nr of Repeats=6 RepeatLength=15 seed=TGGAACAAAA Num.seqs=5 Similarity=0.857778 tpcCG=41.799999 8 AAAAG-AA-ATGGAACA

arqueas_representative:NC_003552.1_Methanosarcina_acetivorans_str._C2A:80:5331652-5331732 Satlength=81 Nr of Repeats=5 RepeatLength=16 seed=GGACAAAAAT Num.seqs=5 Similarity=1.000000 tpcCG=42.700001 11 AAAATGAATCTGG-ACA

**** ** *** ***

Consensus:

AAAAtAAcTGGgACA

>arqueas_representative_Fam_60_17_4 Nr. of seq. 4 Alignment length(with gaps) = 17 Alignment score = 0.653595

arqueas_representative:NZ_CP009517.1_Methanosarcina_barkeri_3:81:965328-965414 Satlength=87 Nr of Repeats=5 RepeatLength=17 seed=CCTTTCTTTT Num.seqs=4 Similarity=1.000000 tpcCG=39.099998 0 CCTTTCTTTTCCATCTT

arqueas_representative:NZ_CP009528.1_Methanosarcina_barkeri_MS:82:1265918-1265986 Satlength=69 Nr of Repeats=4 RepeatLength=17 seed=CGTTTCTATT Num.seqs=4 Similarity=1.000000 tpcCG=39.168999 5 CCTTTCGTTTCTATTTT

Rev.of_arqueas_representative:NZ_CP009501.1_Methanosarcina_thermophila_TM-1:87:1267703-1267788 Satlength=86 Nr of Repeats=4 RepeatLength=17 seed=AAAGATAGAA Num.seqs=3 Similarity=0.633987 tpcCG=41.0999 1 TCTNTNTCTTCTATCTT

arqueas_representative:NZ_CP009520.1_Methanosarcina_vacuolata_Z-761:88:3359671-3359827 Satlength=157 Nr of Repeats=9 RepeatLength=17 seed=TCTTCAATCT Num.seqs=6 Similarity=0.900654 tpcCG=39.732498 6 TCTTTCTCTTCAATCTC

** * *** ** *

Consensus:

cCTtTctcTTCtATcTt

>arqueas_representative_Fam_61_14_4 Nr. of seq. 4 Alignment length(with gaps) = 16 Alignment score = 0.630208

arqueas_representative:NZ_CP009528.1_Methanosarcina_barkeri_MS:82:622506-622590 Satlength=85 Nr of Repeats=6 RepeatLength=14 seed=TGAAATTTAA Num.seqs=6 Similarity=1.000000 tpcCG=39.168999 0 TGA-AATTTAATTTA-

Rev.of_arqueas_representative:NZ_CP009520.1_Methanosarcina_vacuolata_Z-761:88:610438-610671 Satlength=234 Nr of Repeats=13 RepeatLength=14 seed=TAAATTAACT Num.seqs=12 Similarity=1.000000 tpcCG=39.7324 0 TGA-AAGTTAATTTA-

Rev.of_arqueas_representative:NZ_CP009520.1_Methanosarcina_vacuolata_Z-761:88:3252025-3252100 Satlength=76 Nr of Repeats=5 RepeatLength=15 seed=AATTTCACTA Num.seqs=5 Similarity=1.000000 tpcCG=39.73249 7 TGA-AATTTTAATTAG

arqueas_representative:NC_003552.1_Methanosarcina_acetivorans_str._C2A:80:5733090-5733150 Satlength=61 Nr of Repeats=4 RepeatLength=15 seed=TATGAGGGTT Num.seqs=4 Similarity=0.955556 tpcCG=42.700001 13 TGAGGGTTTAATTTA-

*** ** * ***

Consensus:

TGAaatTTaAtTTA

>arqueas_representative_Fam_62_14_4 Nr. of seq. 4 Alignment length(with gaps) = 16 Alignment score = 0.671875

arqueas_representative:NZ_CP009515.1_Methanosarcina_lacustris_Z-7289:84:371541-371695 Satlength=155 Nr of Repeats=11 RepeatLength=14 seed=GTTGTTAAAA Num.seqs=11 Similarity=0.909957 tpcCG=41.799999 0 GTTG-TTAAAAAACA-

Rev.of_arqueas_representative:NZ_CP009520.1_Methanosarcina_vacuolata_Z-761:88:4423922-4423992 Satlength=71 Nr of Repeats=5 RepeatLength=14 seed=TGATTTTTAA Num.seqs=5 Similarity=0.961905 tpcCG=39.73249 0 GTTG-TTAAAAATCA-

Rev.of_arqueas_representative:NZ_CP009512.1_Methanosarcina_mazei_S-6:85:3681937-3681997 Satlength=61 Nr of Repeats=4 RepeatLength=15 seed=TTTTTTAATC Num.seqs=4 Similarity=1.000000 tpcCG=41.400002 13 GTTGATTAAAAAACG-

Rev.of_arqueas_representative:NZ_CP009517.1_Methanosarcina_barkeri_3:81:1736292-1736412 Satlength=121 Nr of Repeats=8 RepeatLength=15 seed=TTTTAATGAC Num.seqs=8 Similarity=0.955556 tpcCG=39.099998 10 GTCA-TTAAAAAACAA

** ******* *

Consensus:

GTtgTTAAAAAaCa

>arqueas_representative_Fam_63_14_4 Nr. of seq. 4 Alignment length(with gaps) = 15 Alignment score = 0.622222

arqueas_representative:NZ_CP009517.1_Methanosarcina_barkeri_3:81:1941986-1942073 Satlength=88 Nr of Repeats=7 RepeatLength=11 seed=ATATAATCTC Num.seqs=6 Similarity=0.927273 tpcCG=39.099998 0 -ATATAAT-CTCA--

arqueas_representative:NZ_CP009517.1_Methanosarcina_barkeri_3:81:1901541-1901601 Satlength=61 Nr of Repeats=5 RepeatLength=12 seed=TACTCAATAT Num.seqs=5 Similarity=0.933333 tpcCG=39.099998 6 -ATATTATACTCA--

arqueas_representative:NZ_CP009517.1_Methanosarcina_barkeri_3:81:355037-355133 Satlength=97 Nr of Repeats=7 RepeatLength=14 seed=CATATAATCT Num.seqs=5 Similarity=0.961905 tpcCG=39.099998 0 CATATAAT-CTCAAC

arqueas_representative:NZ_CP009528.1_Methanosarcina_barkeri_MS:82:1428804-1428888 Satlength=85 Nr of Repeats=6 RepeatLength=14 seed=CCATATAATA Num.seqs=6 Similarity=0.949206 tpcCG=39.168999 13 CATATAAT-ATCAAC

**** ** ***

Consensus:

cATATaATcTCAac

>arqueas_representative_Fam_64_14_4 Nr. of seq. 4 Alignment length(with gaps) = 15 Alignment score = 0.609259

arqueas_representative:NC_014222.1_Methanococcus_voltae_A3:65:1415454-1415506 Satlength=53 Nr of Repeats=4 RepeatLength=13 seed=ATATACTAAC Num.seqs=4 Similarity=1.000000 tpcCG=28.600000 0 ATA-TACTAACTAA-

arqueas_representative:NZ_CP009528.1_Methanosarcina_barkeri_MS:82:3004277-3004375 Satlength=99 Nr of Repeats=7 RepeatLength=14 seed=ATAAACACTA Num.seqs=7 Similarity=0.954649 tpcCG=39.168999 12 AAA-CACTAACTAAT

arqueas_representative:NC_015216.1_Methanobacterium_lacus__AL-21:46:785855-785946 Satlength=92 Nr of Repeats=6 RepeatLength=14 seed=TACTAAATAC Num.seqs=5 Similarity=1.000000 tpcCG=35.799999 3 AAA-TACTAAATACT

arqueas_representative:NZ_CP009528.1_Methanosarcina_barkeri_MS:82:494956-495046 Satlength=91 Nr of Repeats=6 RepeatLength=15 seed=TACTAAATAC Num.seqs=6 Similarity=0.952593 tpcCG=39.168999 3 ATACTACTAAATACC

* * ***** **

Consensus:

AaAtACTAAaTAat

>arqueas_representative_Fam_65_14_4 Nr. of seq. 4 Alignment length(with gaps) = 15 Alignment score = 0.659259

arqueas_representative:NZ_CP009517.1_Methanosarcina_barkeri_3:81:898016-898068 Satlength=53 Nr of Repeats=4 RepeatLength=13 seed=ATCTCATTTT Num.seqs=4 Similarity=1.000000 tpcCG=39.099998 0 ATCTCATT-TTAAC-

Rev.of_arqueas_representative:NZ_CP009520.1_Methanosarcina_vacuolata_Z-761:88:1841944-1841996 Satlength=53 Nr of Repeats=4 RepeatLength=13 seed=TGTTAAAATG Num.seqs=4 Similarity=1.000000 tpcCG=39.73249 1 ATCTCATT-TTAAC-

Rev.of_arqueas_representative:NZ_CP009520.1_Methanosarcina_vacuolata_Z-761:88:3117578-3117639 Satlength=62 Nr of Repeats=5 RepeatLength=13 seed=ATCAGATATT Num.seqs=3 Similarity=1.000000 tpcCG=39.73249 7 ATCTGAT--TTAAAT

arqueas_representative:NZ_CP009501.1_Methanosarcina_thermophila_TM-1:87:275921-276011 Satlength=91 Nr of Repeats=5 RepeatLength=15 seed=TGATTATTAA Num.seqs=4 Similarity=1.000000 tpcCG=41.099998 18 ATCTGATTATTAAAT

**** ** ****

Consensus:

ATCTcATtTTAAat

>arqueas_representative_Fam_66_14_4 Nr. of seq. 4 Alignment length(with gaps) = 15 Alignment score = 0.640741

arqueas_representative:NZ_CP009528.1_Methanosarcina_barkeri_MS:82:4304982-4305060 Satlength=79 Nr of Repeats=6 RepeatLength=13 seed=GAAACAGTAA Num.seqs=6 Similarity=0.945299 tpcCG=39.168999 0 GAAACAG-TA-AGAG

arqueas_representative:NZ_CP009528.1_Methanosarcina_barkeri_MS:82:1391851-1391976 Satlength=126 Nr of Repeats=8 RepeatLength=14 seed=GAGGAAACAG Num.seqs=7 Similarity=0.891156 tpcCG=39.168999 11 GAAACAGAGA-AGAG

arqueas_representative:NZ_CP009517.1_Methanosarcina_barkeri_3:81:3077316-3077427 Satlength=112 Nr of Repeats=6 RepeatLength=14 seed=TGATAAAAGG Num.seqs=4 Similarity=0.936508 tpcCG=39.099998 5 GAAACTGATA-AAAG

arqueas_representative:NZ_CP009517.1_Methanosarcina_barkeri_3:81:526563-526623 Satlength=61 Nr of Repeats=4 RepeatLength=15 seed=AACAGGTACA Num.seqs=4 Similarity=0.940741 tpcCG=39.099998 2 GAAACAGGTACACAG

***** * * * **

Consensus:

GAAACaGatAAgAG

>arqueas_representative_Fam_67_14_4 Nr. of seq. 4 Alignment length(with gaps) = 15 Alignment score = 0.707407

arqueas_representative:NC_003552.1_Methanosarcina_acetivorans_str._C2A:80:622140-622392 Satlength=253 Nr of Repeats=18 RepeatLength=14 seed=AGCTAAAAAC Num.seqs=18 Similarity=1.000000 tpcCG=42.700001 0 AGCTA-AAAACAGAT

arqueas_representative:NZ_CP009506.1_Methanosarcina_siciliae_T4/M:86:543658-543826 Satlength=169 Nr of Repeats=12 RepeatLength=14 seed=AGCTAAAAAC Num.seqs=12 Similarity=1.000000 tpcCG=42.900002 0 AGCTA-AAAACAGAT

Rev.of_arqueas_representative:NZ_CP009506.1_Methanosarcina_siciliae_T4/M:86:4431451-4431569 Satlength=119 Nr of Repeats=8 RepeatLength=14 seed=TTTTAGCTAT Num.seqs=6 Similarity=0.949206 tpcCG=42.900002 8 AGCTA-AAAGCCCAT

Rev.of_arqueas_representative:NZ_CP009515.1_Methanosarcina_lacustris_Z-7289:84:758512-758677 Satlength=166 Nr of Repeats=11 RepeatLength=15 seed=TTATTGCTAT Num.seqs=11 Similarity=0.925657 tpcCG=41.799 8 AGCAATAAAGCAGAT

*** * *** * **

Consensus:

AGCtAAAAaCagAT

>arqueas_representative_Fam_68_14_4 Nr. of seq. 4 Alignment length(with gaps) = 15 Alignment score = 0.751852

arqueas_representative:NC_003552.1_Methanosarcina_acetivorans_str._C2A:80:1359573-1359763 Satlength=191 Nr of Repeats=5 RepeatLength=14 seed=CTTTTTCCTT Num.seqs=3 Similarity=0.936508 tpcCG=42.700001 13 TT-TTTCCTTTTTTC

arqueas_representative:NZ_CP009506.1_Methanosarcina_siciliae_T4/M:86:5003943-5004073 Satlength=131 Nr of Repeats=10 RepeatLength=14 seed=TTTCCTTTCT Num.seqs=6 Similarity=0.968254 tpcCG=42.900002 16 TT-TTTCCTTTCTTC

Rev.of_arqueas_representative:NZ_CP009528.1_Methanosarcina_barkeri_MS:82:2928944-2929601 Satlength=658 Nr of Repeats=8 RepeatLength=14 seed=AGGAAAAAGA Num.seqs=5 Similarity=1.000000 tpcCG=39.168999 22 TT-TTTCCTTTCCTC

Rev.of_arqueas_representative:NZ_CP009517.1_Methanosarcina_barkeri_3:81:4464924-4464999 Satlength=76 Nr of Repeats=5 RepeatLength=15 seed=GAAGAAAAAA Num.seqs=3 Similarity=0.822222 tpcCG=39.099998 17 TTCTTTGCTTTTTTC

*** *** **** *

Consensus:

CTTTTTcCTTTctT

>arqueas_representative_Fam_69_14_4 Nr. of seq. 4 Alignment length(with gaps) = 15 Alignment score = 0.751852

arqueas_representative:NZ_CP009528.1_Methanosarcina_barkeri_MS:82:3038556-3038626 Satlength=71 Nr of Repeats=5 RepeatLength=14 seed=TACAATTTTT Num.seqs=5 Similarity=0.942857 tpcCG=39.168999 0 TACAATT-TTTACAC

Rev.of_arqueas_representative:NZ_CP009520.1_Methanosarcina_vacuolata_Z-761:88:2936605-2936717 Satlength=113 Nr of Repeats=8 RepeatLength=14 seed=GTAAAAATTG Num.seqs=8 Similarity=0.948980 tpcCG=39.7324 12 TACAATT-TTTACAT

Rev.of_arqueas_representative:NZ_CP009512.1_Methanosarcina_mazei_S-6:85:1619131-1619187 Satlength=57 Nr of Repeats=4 RepeatLength=14 seed=TGGAAAAATT Num.seqs=4 Similarity=0.904762 tpcCG=41.400002 13 TACAATT-TTTCCAT

arqueas_representative:NC_015216.1_Methanobacterium_lacus__AL-21:46:2538670-2538766 Satlength=97 Nr of Repeats=5 RepeatLength=15 seed=CCATTACCAT Num.seqs=3 Similarity=0.822222 tpcCG=35.799999 11 TACCATTGTTTCCAT

*** *** *** **

Consensus:

TACaATTTTTaCAt

>arqueas_representative_Fam_70_15_4 Nr. of seq. 4 Alignment length(with gaps) = 15 Alignment score = 0.603704

arqueas_representative:NZ_CP009528.1_Methanosarcina_barkeri_MS:82:3772095-3772151 Satlength=57 Nr of Repeats=4 RepeatLength=14 seed=ATCAGTTAAT Num.seqs=4 Similarity=1.000000 tpcCG=39.168999 0 ATCAGTTAATAGAG-

Rev.of_arqueas_representative:NZ_CP009515.1_Methanosarcina_lacustris_Z-7289:84:150038-150192 Satlength=155 Nr of Repeats=10 RepeatLength=14 seed=CCTGATCCTT Num.seqs=9 Similarity=0.772222 tpcCG=41.7999 6 ATCAGGTAAT-AAGG

arqueas_representative:NZ_CP009506.1_Methanosarcina_siciliae_T4/M:86:4813645-4813855 Satlength=211 Nr of Repeats=10 RepeatLength=15 seed=GGATCAGATA Num.seqs=8 Similarity=0.926984 tpcCG=42.900002 13 ATCAGATAATAAAGG

Rev.of_arqueas_representative:NZ_CP009506.1_Methanosarcina_siciliae_T4/M:86:3977191-3977275 Satlength=85 Nr of Repeats=6 RepeatLength=14 seed=CTCTGTTTAC Num.seqs=6 Similarity=0.784127 tpcCG=42.900002 0 ATCCGTAAACAGAG-

*** * ** **

Consensus:

ATCaGttAAtaaAGg

>arqueas_representative_Fam_71_15_4 Nr. of seq. 4 Alignment length(with gaps) = 15 Alignment score = 0.681481

arqueas_representative:NZ_CP009515.1_Methanosarcina_lacustris_Z-7289:84:1652185-1652283 Satlength=99 Nr of Repeats=6 RepeatLength=14 seed=CCTGAATCGT Num.seqs=5 Similarity=0.923810 tpcCG=41.799999 0 CCTGAATC-GTTCAT

Rev.of_arqueas_representative:NZ_CP009528.1_Methanosarcina_barkeri_MS:82:2392018-2392168 Satlength=151 Nr of Repeats=10 RepeatLength=15 seed=CAGGTTTAAT Num.seqs=10 Similarity=1.000000 tpcCG=39.168999 4 CCTGAATCTATTAAA

arqueas_representative:NZ_CP009520.1_Methanosarcina_vacuolata_Z-761:88:1561391-1561481 Satlength=91 Nr of Repeats=6 RepeatLength=15 seed=TCTGTTAAAC Num.seqs=6 Similarity=0.798519 tpcCG=39.732498 6 CCTGAATCTGTTAAA

arqueas_representative:NZ_CP009520.1_Methanosarcina_vacuolata_Z-761:88:259984-260134 Satlength=151 Nr of Repeats=10 RepeatLength=15 seed=ATCTACGAAT Num.seqs=10 Similarity=0.891358 tpcCG=39.732498 5 CCTGAATCTACGAAT

******** *

Consensus:

CCTGAATCtattaAa

>arqueas_representative_Fam_72_15_4 Nr. of seq. 4 Alignment length(with gaps) = 15 Alignment score = 0.777778

arqueas_representative:NC_009637.1_Methanococcus_maripaludis_C7:62:678156-678306 Satlength=151 Nr of Repeats=10 RepeatLength=15 seed=AGAAGAACCA Num.seqs=10 Similarity=1.000000 tpcCG=33.299999 0 AGAAGAACCAGTGGT

arqueas_representative:NC_015847.1_Methanococcus_maripaludis_X1:63:1493930-1494005 Satlength=76 Nr of Repeats=5 RepeatLength=15 seed=GAACCAGTAG Num.seqs=5 Similarity=1.000000 tpcCG=32.900002 4 AGAAGAACCAGTAGT

Rev.of_arqueas_representative:NC_018876.1_Methanolobus_psychrophilus_R15:74:566728-566881 Satlength=154 Nr of Repeats=7 RepeatLength=15 seed=TTCTACTGGT Num.seqs=6 Similarity=0.863704 tpcCG=44.599998 1 AGAAGCACCAGTAGA

arqueas_representative:NZ_CP019893.1_Natrialbaceae_archaeon_JW/NM-HA_1:99:3750642-3750867 Satlength=226 Nr of Repeats=15 RepeatLength=15 seed=TCGAAGAAGA Num.seqs=15 Similarity=0.966138 tpcCG=64.099998 11 AGAAGAACCGGTCGA

***** *** ** *

Consensus:

AGAAGaACCaGTaGa

>arqueas_representative_Fam_73_13_4 Nr. of seq. 4 Alignment length(with gaps) = 14 Alignment score = 0.656746

arqueas_representative:NC_003552.1_Methanosarcina_acetivorans_str._C2A:80:4446564-4446660 Satlength=97 Nr of Repeats=8 RepeatLength=12 seed=GAAATAGAAA Num.seqs=8 Similarity=0.972222 tpcCG=42.700001 0 -GAAATAGAAA-TG

arqueas_representative:NC_013790.1_Methanobrevibacter_ruminantium_M1:50:2023180-2023232 Satlength=53 Nr of Repeats=4 RepeatLength=13 seed=AAAAATAGAA Num.seqs=4 Similarity=0.948718 tpcCG=32.599998 0 AAAAATAGAAA-TG

Rev.of_arqueas_representative:NZ_CP009528.1_Methanosarcina_barkeri_MS:82:1090564-1090642 Satlength=79 Nr of Repeats=6 RepeatLength=13 seed=TCCATTTTCT Num.seqs=6 Similarity=1.000000 tpcCG=39.168999 2 -GATATAGAAAATG

arqueas_representative:NZ_CP009515.1_Methanosarcina_lacustris_Z-7289:84:2494096-2494187 Satlength=92 Nr of Repeats=7 RepeatLength=13 seed=AATACAAAGT Num.seqs=7 Similarity=0.941392 tpcCG=41.799999 2 -GAAATACAAAGTG

* *** *** **

Consensus:

gAaATAgAAAnTG

>arqueas_representative_Fam_74_14_4 Nr. of seq. 4 Alignment length(with gaps) = 14 Alignment score = 0.650794

arqueas_representative:NZ_CP009528.1_Methanosarcina_barkeri_MS:82:1130379-1130475 Satlength=97 Nr of Repeats=8 RepeatLength=12 seed=TGAAGTAAAT Num.seqs=8 Similarity=1.000000 tpcCG=39.168999 0 TGAA-GTAAATAT-

Rev.of_arqueas_representative:NC_014222.1_Methanococcus_voltae_A3:65:484583-484661 Satlength=79 Nr of Repeats=6 RepeatLength=13 seed=TTTACCTTCA Num.seqs=6 Similarity=1.000000 tpcCG=28.600000 10 TGAAGGTAAATAC-

Rev.of_arqueas_representative:NZ_CP009528.1_Methanosarcina_barkeri_MS:82:3315944-3316009 Satlength=66 Nr of Repeats=5 RepeatLength=13 seed=CCTATATTTA Num.seqs=5 Similarity=0.938462 tpcCG=39.168999 2 -GGAGGTAAATATA

arqueas_representative:NZ_CP009512.1_Methanosarcina_mazei_S-6:85:1836112-1836190 Satlength=79 Nr of Repeats=6 RepeatLength=13 seed=ATAGAAGGTA Num.seqs=6 Similarity=0.876984 tpcCG=41.400002 10 -GAAGGTAACTATA

* * **** **

Consensus:

tGaAgGTAAaTAta

>arqueas_representative_Fam_75_13_4 Nr. of seq. 4 Alignment length(with gaps) = 14 Alignment score = 0.648810

arqueas_representative:NZ_CP009520.1_Methanosarcina_vacuolata_Z-761:88:3709498-3709702 Satlength=205 Nr of Repeats=17 RepeatLength=12 seed=GAAGAAGCTA Num.seqs=17 Similarity=0.933007 tpcCG=39.732498 0 -GAAGAAGCTAAA-

Rev.of_arqueas_representative:NC_003552.1_Methanosarcina_acetivorans_str._C2A:80:1471195-1471259 Satlength=65 Nr of Repeats=5 RepeatLength=13 seed=TTTAGCTTCT Num.seqs=4 Similarity=0.829060 tpcCG=42.70 0 AGCAGAAGCTAAA-

Rev.of_arqueas_representative:NZ_CP009517.1_Methanosarcina_barkeri_3:81:455203-455277 Satlength=75 Nr of Repeats=5 RepeatLength=13 seed=TTTCTTCTTT Num.seqs=4 Similarity=0.948718 tpcCG=39.099998 7 -GAAGAAACTAAAA

Rev.of_arqueas_representative:NC_003552.1_Methanosarcina_acetivorans_str._C2A:80:404320-404398 Satlength=79 Nr of Repeats=6 RepeatLength=13 seed=ACTCCCTTTA Num.seqs=6 Similarity=1.000000 tpcCG=42.7000 5 -GGAGTAACTAAAG

* ** * *****

Consensus:

GaAGaAaCTAAAn

>arqueas_representative_Fam_76_14_4 Nr. of seq. 4 Alignment length(with gaps) = 14 Alignment score = 0.654762

arqueas_representative:NC_003552.1_Methanosarcina_acetivorans_str._C2A:80:1859087-1859152 Satlength=66 Nr of Repeats=5 RepeatLength=13 seed=CAGACTACTT Num.seqs=5 Similarity=1.000000 tpcCG=42.700001 0 CAGACTACTTTA-T

arqueas_representative:NZ_CP009517.1_Methanosarcina_barkeri_3:81:3425376-3425545 Satlength=170 Nr of Repeats=11 RepeatLength=13 seed=TCAGAATTCA Num.seqs=9 Similarity=0.886040 tpcCG=39.099998 12 CAGAATTCATTA-T

Rev.of_arqueas_representative:NZ_CP009515.1_Methanosarcina_lacustris_Z-7289:84:2430855-2431033 Satlength=179 Nr of Repeats=13 RepeatLength=14 seed=AAGGATTTCT Num.seqs=10 Similarity=0.936508 tpcCG=41.7 24 CAGAAATCCTTATT

Rev.of_arqueas_representative:NZ_CP009515.1_Methanosarcina_lacustris_Z-7289:84:2430880-2431130 Satlength=251 Nr of Repeats=18 RepeatLength=14 seed=TAAGGATTTC Num.seqs=16 Similarity=0.830159 tpcCG=41.7 25 CAGAAATCCTTATT

**** * *** *

Consensus:

CAGAaatCcTTAtT

>arqueas_representative_Fam_77_13_4 Nr. of seq. 4 Alignment length(with gaps) = 14 Alignment score = 0.619048

arqueas_representative:NZ_CP009517.1_Methanosarcina_barkeri_3:81:3691544-3691713 Satlength=170 Nr of Repeats=13 RepeatLength=13 seed=TTTTGAGTTT Num.seqs=13 Similarity=0.783883 tpcCG=39.099998 0 TTTTGAGTTTC-NA

Rev.of_arqueas_representative:NZ_CP009528.1_Methanosarcina_barkeri_MS:82:2661883-2662104 Satlength=222 Nr of Repeats=17 RepeatLength=13 seed=TCAAAATAGA Num.seqs=17 Similarity=1.000000 tpcCG=39.168999 6 TTTTGAATTTC-TA

arqueas_representative:NZ_CP009528.1_Methanosarcina_barkeri_MS:82:1212024-1212206 Satlength=183 Nr of Repeats=12 RepeatLength=13 seed=GATTTTCAGT Num.seqs=10 Similarity=1.000000 tpcCG=39.168999 11 TTTTCAGTTTT-GA

arqueas_representative:NC_003552.1_Methanosarcina_acetivorans_str._C2A:80:3969140-3969210 Satlength=71 Nr of Repeats=5 RepeatLength=14 seed=AGATTTTGAG Num.seqs=5 Similarity=0.866667 tpcCG=42.700001 11 TTTTGAGTATTAGA

**** * * * *

Consensus:

TTTTgAgTtTcgA

>arqueas_representative_Fam_78_11_4 Nr. of seq. 4 Alignment length(with gaps) = 13 Alignment score = 0.653846

arqueas_representative:NZ_CP009512.1_Methanosarcina_mazei_S-6:85:472657-472707 Satlength=51 Nr of Repeats=4 RepeatLength=10 seed=TTACTTTTTT Num.seqs=3 Similarity=1.000000 tpcCG=41.400002 6 TTTTT-T--TTAC

Rev.of_arqueas_representative:NZ_CP009517.1_Methanosarcina_barkeri_3:81:4041494-4041551 Satlength=58 Nr of Repeats=5 RepeatLength=11 seed=AAGTAAAGAA Num.seqs=3 Similarity=0.919192 tpcCG=39.099998 12 TTTTTCT--TTAC

Rev.of_arqueas_representative:NZ_CP009517.1_Methanosarcina_barkeri_3:81:4465815-4465959 Satlength=145 Nr of Repeats=12 RepeatLength=12 seed=AAGTAATGAA Num.seqs=12 Similarity=0.966330 tpcCG=39.099998 12 TTTTT-TCATTAC

Rev.of_arqueas_representative:NC_013790.1_Methanobrevibacter_ruminantium_M1:50:2353247-2353346 Satlength=100 Nr of Repeats=5 RepeatLength=11 seed=AATAAGTAAA Num.seqs=3 Similarity=0.797980 tpcCG=32.599 15 TTATTNT--TTAC

****** ** *

Consensus:

TTACTTtTTnT

>arqueas_representative_Fam_79_13_4 Nr. of seq. 4 Alignment length(with gaps) = 13 Alignment score = 0.670940

arqueas_representative:NC_009051.1_Methanoculleus_marisnigri_JR1:68:636074-636239 Satlength=166 Nr of Repeats=15 RepeatLength=11 seed=ATCGTTCATT Num.seqs=15 Similarity=1.000000 tpcCG=62.099998 0 -ATC-GTTCATTC

Rev.of_arqueas_representative:NZ_CP009528.1_Methanosarcina_barkeri_MS:82:2499642-2499713 Satlength=72 Nr of Repeats=6 RepeatLength=12 seed=GTACAGATGA Num.seqs=5 Similarity=1.000000 tpcCG=39.168999 8 -ATCTGTACATTC

Rev.of_arqueas_representative:NZ_CP009517.1_Methanosarcina_barkeri_3:81:1560896-1561052 Satlength=157 Nr of Repeats=13 RepeatLength=12 seed=AATGAACAGA Num.seqs=13 Similarity=0.943020 tpcCG=39.099998 0 TATCTGTTCATT-

arqueas_representative:NC_003552.1_Methanosarcina_acetivorans_str._C2A:80:4130454-4130662 Satlength=209 Nr of Repeats=16 RepeatLength=13 seed=ATCATTATAT Num.seqs=16 Similarity=0.948718 tpcCG=42.700001 7 TATCTGATCATTA

*** * ****

Consensus:

tATCtGttCATTc

>arqueas_representative_Fam_80_12_4 Nr. of seq. 4 Alignment length(with gaps) = 13 Alignment score = 0.685897

arqueas_representative:NC_003552.1_Methanosarcina_acetivorans_str._C2A:80:1792685-1792806 Satlength=122 Nr of Repeats=11 RepeatLength=11 seed=AAATCAATCA Num.seqs=11 Similarity=1.000000 tpcCG=42.700001 0 AAATCAATCAT--

arqueas_representative:NZ_CP009517.1_Methanosarcina_barkeri_3:81:4513958-4514084 Satlength=127 Nr of Repeats=10 RepeatLength=12 seed=AAATCAATCA Num.seqs=9 Similarity=0.938272 tpcCG=39.099998 0 AAATCAATCAAA-

arqueas_representative:NZ_CP009517.1_Methanosarcina_barkeri_3:81:1237378-1237482 Satlength=105 Nr of Repeats=8 RepeatLength=13 seed=TCATTCAAAG Num.seqs=8 Similarity=1.000000 tpcCG=39.099998 3 AAATCATTCAAAG

Rev.of_arqueas_representative:NZ_CP009520.1_Methanosarcina_vacuolata_Z-761:88:2511636-2511708 Satlength=73 Nr of Repeats=6 RepeatLength=12 seed=TTTATTGATT Num.seqs=6 Similarity=0.933333 tpcCG=39.73249 3 AAAGCAATCAAT-

*** ** ***

Consensus:

AAAtCAaTCAaa

>arqueas_representative_Fam_81_11_4 Nr. of seq. 4 Alignment length(with gaps) = 13 Alignment score = 0.666667

arqueas_representative:NZ_CP009517.1_Methanosarcina_barkeri_3:81:282168-282234 Satlength=67 Nr of Repeats=6 RepeatLength=11 seed=CATTAAAATC Num.seqs=6 Similarity=1.000000 tpcCG=39.099998 0 CA--TTAAAATCA

arqueas_representative:NZ_CP009528.1_Methanosarcina_barkeri_MS:82:1504327-1504459 Satlength=133 Nr of Repeats=12 RepeatLength=11 seed=TCAAAATCAA Num.seqs=12 Similarity=1.000000 tpcCG=39.168999 2 AA--TCAAAATCA

arqueas_representative:NZ_CP009512.1_Methanosarcina_mazei_S-6:85:1055916-1055988 Satlength=73 Nr of Repeats=6 RepeatLength=12 seed=ATCAAAATCA Num.seqs=6 Similarity=1.000000 tpcCG=41.400002 2 AA-ATCAAAATCA

Rev.of_arqueas_representative:NZ_CP009512.1_Methanosarcina_mazei_S-6:85:150987-151241 Satlength=255 Nr of Repeats=5 RepeatLength=12 seed=AATTTGATTT Num.seqs=3 Similarity=0.925926 tpcCG=41.400002 3 AAT-TTAAAATCA

* * *******

Consensus:

aATcAAAATCA

>arqueas_representative_Fam_82_13_4 Nr. of seq. 4 Alignment length(with gaps) = 13 Alignment score = 0.779915

arqueas_representative:NZ_CP009528.1_Methanosarcina_barkeri_MS:82:2130607-2130717 Satlength=111 Nr of Repeats=10 RepeatLength=11 seed=AATTAAGGTC Num.seqs=10 Similarity=1.000000 tpcCG=39.168999 0 -AATTAAGGTCA-

Rev.of_arqueas_representative:NZ_CP009528.1_Methanosarcina_barkeri_MS:82:1346160-1346271 Satlength=112 Nr of Repeats=9 RepeatLength=12 seed=TAACCTGAAT Num.seqs=6 Similarity=1.000000 tpcCG=39.168999 0 GAATTCAGGTTA-

Rev.of_arqueas_representative:NZ_CP009517.1_Methanosarcina_barkeri_3:81:1825745-1825862 Satlength=118 Nr of Repeats=8 RepeatLength=13 seed=AACCTGAATT Num.seqs=7 Similarity=0.970696 tpcCG=39.099998 12 GAATTCAGGTTAA

Rev.of_arqueas_representative:NZ_CP009520.1_Methanosarcina_vacuolata_Z-761:88:3173287-3173365 Satlength=79 Nr of Repeats=6 RepeatLength=13 seed=AACCTGAATT Num.seqs=6 Similarity=0.911111 tpcCG=39.73249 12 GAATTCAGGTTAA

**** **** *

Consensus:

gAATTcAGGTtAa

>arqueas_representative_Fam_83_13_4 Nr. of seq. 4 Alignment length(with gaps) = 13 Alignment score = 0.858974

arqueas_representative:NZ_CP009512.1_Methanosarcina_mazei_S-6:85:2861300-2861376 Satlength=77 Nr of Repeats=4 RepeatLength=11 seed=TATCCATCAG Num.seqs=3 Similarity=0.838384 tpcCG=41.400002 0 TATCCATCAGT--

Rev.of_arqueas_representative:NZ_CP009517.1_Methanosarcina_barkeri_3:81:2794162-2794240 Satlength=79 Nr of Repeats=6 RepeatLength=13 seed=TGATTGATAA Num.seqs=6 Similarity=0.904274 tpcCG=39.099998 9 TATCAATCAGTTT

Rev.of_arqueas_representative:NZ_CP009520.1_Methanosarcina_vacuolata_Z-761:88:349722-349839 Satlength=118 Nr of Repeats=9 RepeatLength=13 seed=TGATAAAACT Num.seqs=9 Similarity=0.948718 tpcCG=39.732498 18 TATCAATCAGTTT

Rev.of_arqueas_representative:NZ_CP009520.1_Methanosarcina_vacuolata_Z-761:88:349719-349953 Satlength=235 Nr of Repeats=15 RepeatLength=13 seed=GATTGATAAA Num.seqs=12 Similarity=0.909868 tpcCG=39.7324 21 TATCAATCAGTTT

**** ******

Consensus:

TATCaATCAGTtt

>arqueas_representative_Fam_84_13_4 Nr. of seq. 4 Alignment length(with gaps) = 13 Alignment score = 0.698718

arqueas_representative:NC_009634.1_Methanococcus_vannielii_SB:64:81516-81576 Satlength=61 Nr of Repeats=5 RepeatLength=12 seed=AGCAAGATAC Num.seqs=5 Similarity=1.000000 tpcCG=31.299999 0 AGCAAGATACCC-

Rev.of_arqueas_representative:NZ_CP009528.1_Methanosarcina_barkeri_MS:82:1564682-1564804 Satlength=123 Nr of Repeats=8 RepeatLength=13 seed=AACTTGCTTG Num.seqs=6 Similarity=0.911111 tpcCG=39.168999 8 AGCAAGTTACACA

Rev.of_arqueas_representative:NZ_CP009528.1_Methanosarcina_barkeri_MS:82:2922564-2922653 Satlength=90 Nr of Repeats=7 RepeatLength=13 seed=TTGACTAACT Num.seqs=5 Similarity=0.897436 tpcCG=39.168999 14 AGCAAGTTAGTCA

arqueas_representative:NZ_CP009520.1_Methanosarcina_vacuolata_Z-761:88:2996015-2996093 Satlength=79 Nr of Repeats=6 RepeatLength=13 seed=AAGTTAGTCA Num.seqs=6 Similarity=1.000000 tpcCG=39.732498 16 AACAAGTTAGTCA

* **** ** *

Consensus:

AgCAAGtTActCa

>arqueas_representative_Fam_85_12_4 Nr. of seq. 4 Alignment length(with gaps) = 13 Alignment score = 0.711538

arqueas_representative:NC_003552.1_Methanosarcina_acetivorans_str._C2A:80:1922633-1922729 Satlength=97 Nr of Repeats=8 RepeatLength=12 seed=AAATAATATC Num.seqs=8 Similarity=0.896825 tpcCG=42.700001 0 AAATAATATCTA-

Rev.of_arqueas_representative:NZ_CP009528.1_Methanosarcina_barkeri_MS:82:3105016-3105208 Satlength=193 Nr of Repeats=16 RepeatLength=12 seed=ATTATTTTAG Num.seqs=16 Similarity=0.902244 tpcCG=39.168999 7 AAATAATCTCTA-

arqueas_representative:NZ_CP009512.1_Methanosarcina_mazei_S-6:85:2548266-2548346 Satlength=81 Nr of Repeats=5 RepeatLength=13 seed=GTAGAAATAA Num.seqs=3 Similarity=0.863248 tpcCG=41.400002 9 AAATAATATGTAG

Rev.of_arqueas_representative:NZ_CP009517.1_Methanosarcina_barkeri_3:81:1857906-1857990 Satlength=85 Nr of Repeats=7 RepeatLength=12 seed=TAATTTAGGT Num.seqs=7 Similarity=0.968254 tpcCG=39.099998 5 AATTAATACCTA-

** **** **

Consensus:

AAaTAATatcTA

>arqueas_representative_Fam_86_13_4 Nr. of seq. 4 Alignment length(with gaps) = 13 Alignment score = 0.698718

arqueas_representative:NZ_CP009520.1_Methanosarcina_vacuolata_Z-761:88:511961-512057 Satlength=97 Nr of Repeats=8 RepeatLength=12 seed=AAACTAACTG Num.seqs=8 Similarity=0.912698 tpcCG=39.732498 0 AAACTAACTGCT-

Rev.of_arqueas_representative:NZ_CP009517.1_Methanosarcina_barkeri_3:81:2404180-2404349 Satlength=170 Nr of Repeats=9 RepeatLength=13 seed=TTTGAGGAGT Num.seqs=7 Similarity=1.000000 tpcCG=39.099998 3 AAACTAACTCCTC

Rev.of_arqueas_representative:NZ_CP009528.1_Methanosarcina_barkeri_MS:82:2386711-2386971 Satlength=261 Nr of Repeats=20 RepeatLength=13 seed=TTGAGTTTTT Num.seqs=20 Similarity=0.864474 tpcCG=39.168999 2 AAACTAAAAACTC

Rev.of_arqueas_representative:NZ_CP009506.1_Methanosarcina_siciliae_T4/M:86:2069035-2069126 Satlength=92 Nr of Repeats=7 RepeatLength=13 seed=TTTTAGCTTT Num.seqs=7 Similarity=0.951160 tpcCG=42.900002 3 AAACTAAAAGCTA

******* **

Consensus:

AAACTAAaagCTc

>arqueas_representative_Fam_87_11_4 Nr. of seq. 4 Alignment length(with gaps) = 12 Alignment score = 0.671296

arqueas_representative:NZ_CP014265.1_Methanobrevibacter_olleyae__YLM1:49:396932-396984 Satlength=53 Nr of Repeats=4 RepeatLength=11 seed=ATTATATTCT Num.seqs=3 Similarity=0.919192 tpcCG=26.900000 4 T-CTAATTATAT

Rev.of_arqueas_representative:NZ_CP009520.1_Methanosarcina_vacuolata_Z-761:88:1369777-1369854 Satlength=78 Nr of Repeats=7 RepeatLength=11 seed=TATAATTAGA Num.seqs=7 Similarity=1.000000 tpcCG=39.73249 10 T-CTAATTATAC

arqueas_representative:NZ_CP009517.1_Methanosarcina_barkeri_3:81:4088296-4088340 Satlength=45 Nr of Repeats=4 RepeatLength=11 seed=TTTATATTAT Num.seqs=4 Similarity=0.939394 tpcCG=39.099998 4 T-ATATTTATAT

Rev.of_arqueas_representative:NZ_CP009517.1_Methanosarcina_barkeri_3:81:3913431-3913479 Satlength=49 Nr of Repeats=4 RepeatLength=12 seed=AATATGAATA Num.seqs=4 Similarity=0.870370 tpcCG=39.099998 6 TCATATTTATAT

***** * **

Consensus:

aTTATAtTaTA

>arqueas_representative_Fam_88_12_4 Nr. of seq. 4 Alignment length(with gaps) = 12 Alignment score = 0.648148

arqueas_representative:NC_013790.1_Methanobrevibacter_ruminantium_M1:50:98303-98677 Satlength=375 Nr of Repeats=21 RepeatLength=11 seed=TTATAGTTTA Num.seqs=14 Similarity=0.857476 tpcCG=32.599998 0 TT-ATAGTTTAG

arqueas_representative:NC_014222.1_Methanococcus_voltae_A3:65:282335-282383 Satlength=49 Nr of Repeats=4 RepeatLength=12 seed=TTAATAGTTT Num.seqs=4 Similarity=0.944444 tpcCG=28.600000 0 TTAATAGTTTAG

arqueas_representative:NZ_CP009515.1_Methanosarcina_lacustris_Z-7289:84:1209039-1209259 Satlength=221 Nr of Repeats=19 RepeatLength=11 seed=TTATTTGTTA Num.seqs=18 Similarity=0.930283 tpcCG=41.799999 0 TTATTTGTT-AG

arqueas_representative:NZ_CP009512.1_Methanosarcina_mazei_S-6:85:3308339-3308411 Satlength=73 Nr of Repeats=6 RepeatLength=12 seed=TTCAGTTATT Num.seqs=6 Similarity=0.866667 tpcCG=41.400002 7 TTATTTGTTCAG

** * *** **

Consensus:

TTaaTaGTTtAG

>arqueas_representative_Fam_89_12_4 Nr. of seq. 4 Alignment length(with gaps) = 12 Alignment score = 0.692130

arqueas_representative:NZ_CP009515.1_Methanosarcina_lacustris_Z-7289:84:28156-28231 Satlength=76 Nr of Repeats=6 RepeatLength=11 seed=TTTTTTATTT Num.seqs=5 Similarity=0.878788 tpcCG=41.799999 8 TTTATTTC-TTT

arqueas_representative:NC_003552.1_Methanosarcina_acetivorans_str._C2A:80:2827135-2827269 Satlength=135 Nr of Repeats=8 RepeatLength=12 seed=TTTCTTTTTT Num.seqs=7 Similarity=1.000000 tpcCG=42.700001 15 TTGATTTCTTTT

Rev.of_arqueas_representative:NZ_CP009517.1_Methanosarcina_barkeri_3:81:3127865-3127937 Satlength=73 Nr of Repeats=6 RepeatLength=12 seed=ATTCAAAAAT Num.seqs=6 Similarity=0.881481 tpcCG=39.099998 13 TTCATTTTTGAA

Rev.of_arqueas_representative:NZ_CP009515.1_Methanosarcina_lacustris_Z-7289:84:1362907-1362955 Satlength=49 Nr of Repeats=4 RepeatLength=12 seed=ATCGAAAAAT Num.seqs=4 Similarity=0.944444 tpcCG=41.7999 13 TTCATTTTTCGA

*** ** *

Consensus:

TTTctttaTTcA

>arqueas_representative_Fam_90_12_4 Nr. of seq. 4 Alignment length(with gaps) = 12 Alignment score = 0.685185

arqueas_representative:NC_017941.2_Haloferax_mediterranei_ATCC_33500:25:1330657-1331215 Satlength=559 Nr of Repeats=7 RepeatLength=12 seed=GGTTCCGGCT Num.seqs=5 Similarity=0.866667 tpcCG=60.255100 0 GGTTCCGGCTCT

arqueas_representative:NC_018227.2_Methanoculleus_bourgensis_MS2T_:67:1863135-1863414 Satlength=280 Nr of Repeats=5 RepeatLength=12 seed=CCGGTCTGGT Num.seqs=3 Similarity=0.925926 tpcCG=60.599998 5 GGTTCCCGGTCT

arqueas_representative:NC_009515.1_Methanobrevibacter_smithii_ATCC_35061:51:1105439-1105517 Satlength=79 Nr of Repeats=6 RepeatLength=12 seed=TCAGGTTCCG Num.seqs=5 Similarity=0.866667 tpcCG=31.000000 3 GGATCAGGTTCT

Rev.of_arqueas_representative:NC_015676.1_Methanosalsum_zhilinae_DSM_4017:79:232762-232870 Satlength=109 Nr of Repeats=9 RepeatLength=12 seed=CCAGAGCCTG Num.seqs=9 Similarity=0.919753 tpcCG=39.200001 14 GGCTCAGGCTCT

** ** * ***

Consensus:

GGtTCagGcTCT

>arqueas_representative_Fam_91_12_4 Nr. of seq. 4 Alignment length(with gaps) = 12 Alignment score = 0.685185

arqueas_representative:NZ_CP009517.1_Methanosarcina_barkeri_3:81:3220627-3220699 Satlength=73 Nr of Repeats=6 RepeatLength=12 seed=TTGCTGTTTG Num.seqs=6 Similarity=1.000000 tpcCG=39.099998 0 TTGCTGTTTGAA

Rev.of_arqueas_representative:NZ_CP009520.1_Methanosarcina_vacuolata_Z-761:88:1384026-1384098 Satlength=73 Nr of Repeats=5 RepeatLength=12 seed=GCAATTCAAA Num.seqs=4 Similarity=1.000000 tpcCG=39.73249 4 TTGCTATTTGAA

Rev.of_arqueas_representative:NZ_CP009528.1_Methanosarcina_barkeri_MS:82:2915666-2915726 Satlength=61 Nr of Repeats=4 RepeatLength=12 seed=TTCAGATGGC Num.seqs=3 Similarity=0.777778 tpcCG=39.168999 0 TTGCCATCTGAA

arqueas_representative:NZ_CP009506.1_Methanosarcina_siciliae_T4/M:86:4838339-4838387 Satlength=49 Nr of Repeats=4 RepeatLength=12 seed=ATTGAATTTC Num.seqs=4 Similarity=1.000000 tpcCG=42.900002 6 TTTCTCATTGAA

** * ****

Consensus:

TTgCtattTGAA

>arqueas_representative_Fam_92_10_4 Nr. of seq. 4 Alignment length(with gaps) = 11 Alignment score = 0.638889

arqueas_representative:NC_015574.1_Methanobacterium_paludis__SWAN1:47:628951-629051 Satlength=101 Nr of Repeats=9 RepeatLength=10 seed=TAAAAAGGAT Num.seqs=8 Similarity=1.000000 tpcCG=35.700001 0 TAAAAAGGAT-

Rev.of_arqueas_representative:NZ_CP009520.1_Methanosarcina_vacuolata_Z-761:88:2249817-2249861 Satlength=45 Nr of Repeats=4 RepeatLength=11 seed=TTTTTTAGAT Num.seqs=4 Similarity=0.939394 tpcCG=39.73249 7 TAAAAAAGATC

Rev.of_arqueas_representative:CP009516.1_Methanosarcina_horonobensis_HB-1:83:4245092-4245222 Satlength=131 Nr of Repeats=12 RepeatLength=10 seed=CCCTCTTTAA Num.seqs=11 Similarity=0.932121 tpcCG=41.299 9 TAAAGAGGGT-

Rev.of_arqueas_representative:NZ_CP009501.1_Methanosarcina_thermophila_TM-1:87:2618992-2619061 Satlength=70 Nr of Repeats=6 RepeatLength=10 seed=TCCTCTTTAT Num.seqs=5 Similarity=1.000000 tpcCG=41.0999 9 TAAAGAGGAA-

**** * *

Consensus:

TAAAaAgGat

>arqueas_representative_Fam_93_11_4 Nr. of seq. 4 Alignment length(with gaps) = 11 Alignment score = 0.717172

arqueas_representative:NZ_CP009517.1_Methanosarcina_barkeri_3:81:1439875-1440007 Satlength=133 Nr of Repeats=12 RepeatLength=11 seed=ATTGAAAACT Num.seqs=12 Similarity=0.898990 tpcCG=39.099998 0 ATTGAAAACTA

Rev.of_arqueas_representative:NZ_CP009528.1_Methanosarcina_barkeri_MS:82:230360-230503 Satlength=144 Nr of Repeats=13 RepeatLength=11 seed=TTTCAATTGT Num.seqs=13 Similarity=1.000000 tpcCG=39.168999 7 ATTGAAAAACA

Rev.of_arqueas_representative:NZ_CP009528.1_Methanosarcina_barkeri_MS:82:1447847-1448023 Satlength=177 Nr of Repeats=16 RepeatLength=11 seed=ATTTCCAATT Num.seqs=16 Similarity=0.895960 tpcCG=39.168999 9 ATTGGAAATTA

Rev.of_arqueas_representative:NZ_CP009506.1_Methanosarcina_siciliae_T4/M:86:128706-128761 Satlength=56 Nr of Repeats=5 RepeatLength=11 seed=CAATTTATTT Num.seqs=5 Similarity=1.000000 tpcCG=42.900002 15 ATTGGAAATAA

**** *** *

Consensus:

ATTGaAAAttA

>arqueas_representative_Fam_94_11_4 Nr. of seq. 4 Alignment length(with gaps) = 11 Alignment score = 0.858586

arqueas_representative:NZ_CP009520.1_Methanosarcina_vacuolata_Z-761:88:2919230-2919296 Satlength=67 Nr of Repeats=6 RepeatLength=11 seed=ATATTATGAG Num.seqs=6 Similarity=0.927273 tpcCG=39.732498 0 ATATTATGAGA

arqueas_representative:NZ_CP009520.1_Methanosarcina_vacuolata_Z-761:88:3254653-3254730 Satlength=78 Nr of Repeats=7 RepeatLength=11 seed=ATTATGAGAA Num.seqs=7 Similarity=0.861472 tpcCG=39.732498 2 ATATTATGAGA

arqueas_representative:NZ_CP009520.1_Methanosarcina_vacuolata_Z-761:88:3596737-3596913 Satlength=177 Nr of Repeats=16 RepeatLength=11 seed=TTATGAGGAT Num.seqs=16 Similarity=0.971717 tpcCG=39.732498 3 ATATTATGAGG

Rev.of_arqueas_representative:NZ_CP009520.1_Methanosarcina_vacuolata_Z-761:88:3870500-3870555 Satlength=56 Nr of Repeats=5 RepeatLength=11 seed=TCATAATGTC Num.seqs=5 Similarity=0.927273 tpcCG=39.73249 9 ACATTATGAGG

* ********

Consensus:

AtATTATGAGa

>arqueas_representative_Fam_95_258_3 Nr. of seq. 3 Alignment length(with gaps) = 264 Alignment score = 0.640152

arqueas_representative:NZ_CP009520.1_Methanosarcina_vacuolata_Z-761:88:603566-604577 Satlength=1012 Nr of Repeats=4 RepeatLength=255 seed=TATACTGTTA Num.seqs=3 Similarity=0.845920 tpcCG=39.732498 0 TATACTGTTAAACTTACAGCAACAAATGCAGCAGGAAGTAATACAAAAACAAAATCAAAATATATCANAGTAACAGCTAC------TTCACAAGCTCCTGTTGCAG-ATTTCTGGGGCTGGCC-ACTCTCAGGAAAAGCTCC-ACTAAAGGTAACATTTACTGAGACCAGTACNGGCTCCCCAACTTCCTGGAAATGGGATTTTGGAGATGGTACATCTTCAACAGAACAGAGTCCAACACACACATATTCATCTGCAGGAACT

arqueas_representative:CP009516.1_Methanosarcina_horonobensis_HB-1:83:4667608-4669405 Satlength=1798 Nr of Repeats=7 RepeatLength=258 seed=TGGAACTGGA Num.seqs=5 Similarity=0.587326 tpcCG=41.299999 183 TACACGGTAANACTNACAGCAACCAATGCNGCAGGCAGCAATACGGTAACAAAATCAAATTACATAACAGTNACAGGAAC-A--TCTGCACAAAANCCGGTTGCANNNTTT-TCGGCATCTCCNACN-TCAGGAAATGCACC-ATTGANTGTCANNTTTACTGACAGNAGTACAGGNTCTCCAACAGCNTGGAACTGGAGTTTCGGAGACGGAACANANTCAACAGTCCAGAATCCNANGCACACATATTCNACAGCAGGAAAT

arqueas_representative:NZ_CP009515.1_Methanosarcina_lacustris_Z-7289:84:267326-268622 Satlength=1297 Nr of Repeats=5 RepeatLength=258 seed=ATTTCGGAGA Num.seqs=4 Similarity=0.800341 tpcCG=41.799999 193 TACACAATAAAGCTGACAGCAACCAATGCTGCAGGCAGTAACACGCTAACAAAATACAATTACATAACANTTACAGGAACGACGGCGGCACAAACGCCGGTTGCTGCATTT-TCGGCATCTCCTACT-TCCGGAAATTCACCTATGGA-TGTGACCTTTACTGACAGCAGTACAGGAACTCCAACAGCATGGAGCTGGAATTTCGGAGACGGGACTACTTCAGCAATCCAGAACCCAAAACACACATATTCAACAGCAGGAACC

** ** * * ** ******** ***** ***** ** ** ** ******** ** ** ** * * * **** ** ***** ** ***** *** * ** * ** ** ** ***** * ** * * ** * ******** * ***** ** * ***** * **** *** *** ***** ** ** *** ** **** ** * *********** * *******

Consensus:

TAcACngTaAaaCTnACAGCAACcAATGCnGCAGGcAGtAAtACgntAACAAAATcaAAtTAcATaAcAgTnACAGgaACanctgCACAAacnCCgGTTGCagnaTTTTcGGcaTctCCnACtTCaGGAAAtgCaCCAttgAntGTnAcnTTTACTGAcAgcAGTACaGGntCtCCAACagCnTGGAacTGGaaTTTcGGAGAcGGnACanctTCAaCAgtcCAGAatCCaAnaCACACATATTCaaCaGCAGGAAct

>arqueas_representative_Fam_96_137_3 Nr. of seq. 3 Alignment length(with gaps) = 142 Alignment score = 0.667449

arqueas_representative:NC_003552.1_Methanosarcina_acetivorans_str._C2A:80:1869487-1870177 Satlength=691 Nr of Repeats=5 RepeatLength=138 seed=AATCTTGAAA Num.seqs=5 Similarity=0.815827 tpcCG=42.700001 0 AATCTTGAAACTGCTATCAGTTTTTACGGCGA-TG-C-CAGGGAAATTTTTCCCAAAAA-AAGCATAGATTACGCTATTGCGTTGATGAACGAGGGTAATGCAAGACAAACACTTGCAGAAATGGGCATTGAAAGTAGGGAA

arqueas_representative:CP009516.1_Methanosarcina_horonobensis_HB-1:83:3514624-3515728 Satlength=1105 Nr of Repeats=8 RepeatLength=138 seed=AATCTTGAAA Num.seqs=8 Similarity=0.546559 tpcCG=41.299999 0 AATCTTGAAACTGCTATCCATCTTTA-TGAGACTGTC-CA-GGAAA-TATTCCCAAAAACAAGTGTAGATTACGCTCGTGCATTGACNAACGAGGGTAATGCAAGAGAANTACTTGCAGAGATGGGTGTTGATAGTAANGAA

Rev.of_arqueas_representative:NZ_CP009515.1_Methanosarcina_lacustris_Z-7289:84:1044635-1045463 Satlength=829 Nr of Repeats=6 RepeatLength=138 seed=GCAAGTGTTT Num.seqs=6 Similarity=0.879549 tpcCG=41.79 113 AATCTTGAAACCGCGGTCGGTCTTTACGGTGA-CG-CTCA-GAAAA-TATTCCCCAAAGCAAGCGCATCTTACGCTCGTGCGTTGATGAATGAGGGTTCTGCAAGACAAACACTTGCTGGAATGGGTGTTGACAGCAGGTCA

*********** ** ** * **** * ** * * ** * *** * ***** *** *** * ******* *** **** ** ****** ******* ** ****** * ***** **** ** * *

Consensus:

AATCTTGAAACtGCtaTCngTcTTTAcgGnGAtGCCAGgAAATaTTCCCaAAAacAAGcgtAgaTTACGCTcgTGCgTTGAtgAAcGAGGGTaaTGCAAGAcAAacACTTGCaGaaATGGGtgTTGAnAGtAgggaA

>arqueas_representative_Fam_97_126_3 Nr. of seq. 3 Alignment length(with gaps) = 132 Alignment score = 0.647306

arqueas_representative:NZ_CP009512.1_Methanosarcina_mazei_S-6:85:1083972-1084494 Satlength=523 Nr of Repeats=4 RepeatLength=126 seed=TCTGTAATTG Num.seqs=3 Similarity=0.549431 tpcCG=41.400002 123 GTAATTGATACANCTACAAACANAGTTACAGNCACTGTNCCTGTAGGAGAC-GATCCTTNTGACGTTGCATTCAGTCCTGAT-GGAAAAAAGGTNTATGTNACAAATAN-ACGCAGCAACAATG-TTTCT--

arqueas_representative:NZ_CP009506.1_Methanosarcina_siciliae_T4/M:86:2224144-2224924 Satlength=781 Nr of Repeats=6 RepeatLength=126 seed=GTAATTGACA Num.seqs=4 Similarity=0.473251 tpcCG=42.900002 126 GTAATTGACACAGCTACAAACACTGTTACAGCCACAGTTAATGTAGGAGATTACTCCTATNGAAGTTGCAGTCACTCCTGAC-GGAAAAAAAGTTTATGTGACAAACNCNA-ATAGCAACAATA-TTTCT--

arqueas_representative:NZ_CP009520.1_Methanosarcina_vacuolata_Z-761:88:3765255-3765900 Satlength=646 Nr of Repeats=5 RepeatLength=126 seed=TCTGTAATTG Num.seqs=4 Similarity=0.620017 tpcCG=39.732498 250 GTAATTGACACAGCTACAAACACTGTTACAGCCACTGTAAATGTAGGA-ACTNATCCTAATGGAGTTGCAGTCAGCCCNAACNGGAAAAAAAGTATATGTGACGAA--C-A-ATAGAGAAGATACTGTCTCT

*** ******** *** ********* ******* *** ** ******* * **** * ****** *** ** * ******** ** ***** ** ** * ** * ** *

Consensus:

TCTGTAATTGAcACAgCTACAAACActGTTACAGcCACtGTnaaTGTAGGAgActnaTCCTantGaaGTTGCAgTCAgtCCtgAcGGAAAAAAaGTnTATGTgACaAAnncAatAGcaAcaATaTt

>arqueas_representative_Fam_98_120_3 Nr. of seq. 3 Alignment length(with gaps) = 121 Alignment score = 0.666667

arqueas_representative:NC_003552.1_Methanosarcina_acetivorans_str._C2A:80:5689574-5690180 Satlength=607 Nr of Repeats=4 RepeatLength=120 seed=AAGAAGCAGT Num.seqs=3 Similarity=0.888889 tpcCG=42.700001 0 AAGAAGCAGTAAAAAAGTATAACCAGTCACTGGAAATGAAGGAAGAACTGGGGGACAAAAGCGGAATTGCAATAACACTGCACCAGCTTGGAAATATTC-ATTATTCTCAGGGCAACTACG

Rev.of_arqueas_representative:NZ_CP009528.1_Methanosarcina_barkeri_MS:82:1282269-1283229 Satlength=961 Nr of Repeats=7 RepeatLength=120 seed=ATTTTCAGTG Num.seqs=6 Similarity=0.748816 tpcCG=39.168999 37 AAGAAGCAGTGGAAAAGTATAACCAGTCACTGAAAATTAAAGAAGAGCTTGGAGACAAAAGCGGAATTGCACAAACACTGCACCAGCTTGGAATGATTT-ATCAGCATCAGGGCAATTACG

Rev.of_arqueas_representative:CP009516.1_Methanosarcina_horonobensis_HB-1:83:1637802-1639125 Satlength=1324 Nr of Repeats=5 RepeatLength=120 seed=GCTTCTTCAT Num.seqs=3 Similarity=0.478836 tpcCG=41.299 7 AAGAAGCNATGAAAATATATAACCAAGCTCTNGAAATTTNAGAAGATNTTGGAAATAAGGATGGAATTTCAGNAGCANTTCACCANNTTGGAA-GATTTNATTATCATCAGGGAAATTATG

******* * *** ******** * ** **** ***** * ** * ** ****** ** * ** * ***** ****** *** ** * ****** ** ** *

Consensus:

AAGAAGCagTgaAAAagTATAACCAgtCaCTggAAATtaaaGAAGAncTtGGagAcAAaagcGGAATTgCAnnAaCAcTgCACCAgcTTGGAAngATTtATtAtcaTCAGGGcAAtTAcG

>arqueas_representative_Fam_99_120_3 Nr. of seq. 3 Alignment length(with gaps) = 120 Alignment score = 0.679630

arqueas_representative:NC_003552.1_Methanosarcina_acetivorans_str._C2A:80:2148492-2149212 Satlength=721 Nr of Repeats=4 RepeatLength=120 seed=TAACTCCATT Num.seqs=3 Similarity=0.725436 tpcCG=42.700001 0 TAACTCCATTTGAGGGTTCCGTCAGGGTTNAGTGCATAGAGTTTNTTGTCCNNGCTTCCGATGTAGATGGTGCCGTCCTCTCCGATTGCTGCCGAACTGAAGATCTGATTTCCGGTGGTA

arqueas_representative:NZ_CP009506.1_Methanosarcina_siciliae_T4/M:86:3003422-3004025 Satlength=604 Nr of Repeats=5 RepeatLength=120 seed=TCCATTTTAG Num.seqs=3 Similarity=0.730028 tpcCG=42.900002 4 TAAGTCCATTTTAGGGTTCCGTCAGAGTTCAATGCGTATAGGTTATTGTCACGACTTCCGATATAGATGGTTCCGTCAGATCCAATTGCNGGTGAACTGTAGATCTNTCTTCCGGTGGTG

Rev.of_arqueas_representative:NZ_CP009517.1_Methanosarcina_barkeri_3:81:513646-514366 Satlength=721 Nr of Repeats=6 RepeatLength=120 seed=ACTCAAATGG Num.seqs=6 Similarity=0.656566 tpcCG=39.099998 15 TATGACCATTTGAGTGTTCCGTCAGGATTCAGGGCATATAGTTTTTTATCATAGCTTCCTATGTAGATGGTTCCGTCTGCCCCTATTGCTGGTGAACCGTCTATNGTATCTCCAGTGGTG

** ****** ** ********** ** * ** ** ** ** ** ** ***** ** ******** ***** ** ***** * **** * ** *** *****

Consensus:

TAagtCCATTTgAGgGTTCCGTCAGggTTcAgtGCaTAtAGtTTnTTgTCanngCTTCCgATgTAGATGGTtCCGTCngctCCnATTGCtGgtGAACtGtagATctnattTCCgGTGGTg

>arqueas_representative_Fam_100_102_3 Nr. of seq. 3 Alignment length(with gaps) = 102 Alignment score = 0.738562

arqueas_representative:NZ_CP009515.1_Methanosarcina_lacustris_Z-7289:84:2060410-2060920 Satlength=511 Nr of Repeats=5 RepeatLength=102 seed=TTATACTGTT Num.seqs=5 Similarity=0.870513 tpcCG=41.799999 0 TTATACTGTTCTTCTGCCTCTTCCGGCCGCCCCATTTGTTTAAGGAGATTTCCGTAATTGTAGTGAGTAGCTACATGTTTTGGGTCAGTTTCCAGGGCAAGC

Rev.of_arqueas_representative:NZ_CP009520.1_Methanosarcina_vacuolata_Z-761:88:3118718-3119432 Satlength=715 Nr of Repeats=6 RepeatLength=102 seed=AACAGTATAA Num.seqs=5 Similarity=0.687820 tpcCG=39.732 10 TTATACTGTTCTTCTGCTTCNTCNAGGCGCCCCATNTCTGAAAGGAGATTNCCGTAATTGGAGTGTGTGTTGACATNTTTTGGGTCTGCTTCCAGAGCAAGT

Rev.of_arqueas_representative:NZ_CP009520.1_Methanosarcina_vacuolata_Z-761:88:3121970-3122600 Satlength=631 Nr of Repeats=5 RepeatLength=102 seed=GAAGAACAGT Num.seqs=3 Similarity=0.630342 tpcCG=39.732 14 TTATACTGTTCTTCTGCTTCTTCAAGACGTCCCATTTCTTGCAGGAGAATTCCGTAANTTAAGTGTGCATTCACATGTTTGGGATCTGCTTCCAGAGCAAGC

***************** ** ** * ** ***** * * ****** * ****** * **** * **** *** ** ** * ****** *****

Consensus:

TTATACTGTTCTTCTGCtTCtTCnaGnCGcCCCATtTcTtnaAGGAGAtTtCCGTAAtTgnAGTGtGtattnACATgTTTtGGgTCtGcTTCCAGaGCAAGc

>arqueas_representative_Fam_101_93_3 Nr. of seq. 3 Alignment length(with gaps) = 93 Alignment score = 0.930705

arqueas_representative:NZ_CP009520.1_Methanosarcina_vacuolata_Z-761:88:3373642-3374479 Satlength=838 Nr of Repeats=9 RepeatLength=93 seed=TTTCCAAGTG Num.seqs=9 Similarity=0.746257 tpcCG=39.732498 0 TTTCCAAGTGCCTCTGCCGCACTTCTCCGCACATATGAATCTTCATCTTTGAGTGCGTTGATTAGCGGCTGCACNGCTGTATCCGATTTNATA

arqueas_representative:NZ_CP009520.1_Methanosarcina_vacuolata_Z-761:88:3373642-3374851 Satlength=1210 Nr of Repeats=13 RepeatLength=93 seed=TTTCCAAGTG Num.seqs=13 Similarity=0.743953 tpcCG=39.732498 0 TTTCCAAGTGCCTCTGCCGCACTNCTCCGCACATATGAATCTTTATCTTTGAGTGCGTTGATTAACGGCTGCACTGCTGTATCCGATTTAATA

arqueas_representative:NZ_CP009520.1_Methanosarcina_vacuolata_Z-761:88:3373643-3375596 Satlength=1954 Nr of Repeats=18 RepeatLength=93 seed=TTCCAAGTGC Num.seqs=14 Similarity=0.733380 tpcCG=39.732498 1 TTTCCAAGTGCCTCTGCCGCACTCCNCCGCACATATGAATCTTCATCTTTGAGTGCGTTGATTAACGGCTGCACTGCTNTATCCGACTTAATA

*********************** * ***************** ******************** ********* *** ******* ** ***

Consensus:

TTTCCAAGTGCCTCTGCCGCACTnCtCCGCACATATGAATCTTcATCTTTGAGTGCGTTGATTAaCGGCTGCACtGCTgTATCCGAtTTaATA

>arqueas_representative_Fam_102_51_3 Nr. of seq. 3 Alignment length(with gaps) = 54 Alignment score = 0.602881

arqueas_representative:NC_009051.1_Methanoculleus_marisnigri_JR1:68:1007815-1008109 Satlength=295 Nr of Repeats=6 RepeatLength=49 seed=TTCGGCCTTC Num.seqs=6 Similarity=1.000000 tpcCG=62.099998 0 TTCG-GCCTTCTCGTGCTCCTGCCCTTCG---GCCAGTCGCCAGTCCAAGAC-C

arqueas_representative:NC_009051.1_Methanoculleus_marisnigri_JR1:68:1051872-1052127 Satlength=256 Nr of Repeats=5 RepeatLength=51 seed=AGCTCCGGCC Num.seqs=5 Similarity=0.989542 tpcCG=62.099998 14 TTCGCGTTGTCTCCAGCTCCGGCCCTTCG---GCCCGTCGCCATTCGAAAATGC

arqueas_representative:NC_009051.1_Methanoculleus_marisnigri_JR1:68:2023212-2023524 Satlength=313 Nr of Repeats=6 RepeatLength=52 seed=CCCGCAAGTC Num.seqs=6 Similarity=1.000000 tpcCG=62.099998 34 TTCGCGTTTTCTCCTGCTCCGGCCCTTCGTAATTCC--CGCAAGTCGAAAACGC

**** * **** ***** ******** * *** * ** ** * *

Consensus:

TTCGcGtttTCTCctGCTCCgGCCCTTCGgcCcgtCGCcAgTCgAAaAcgC

>arqueas_representative_Fam_103_42_3 Nr. of seq. 3 Alignment length(with gaps) = 48 Alignment score = 0.800926

arqueas_representative:NC_007796.1_Methanospirillum_hungatei_JF-1:91:1464539-1465799 Satlength=1261 Nr of Repeats=23 RepeatLength=42 seed=CCTCTTCGGT Num.seqs=21 Similarity=0.980650 tpcCG=45.099998 0 CCTCTTCGGTAGGTTCAGGAGTCA--TCGTAG----GCTCTGGAGTGA

arqueas_representative:NC_007796.1_Methanospirillum_hungatei_JF-1:91:1464547-1466413 Satlength=1867 Nr of Repeats=41 RepeatLength=42 seed=GTAGGTTCAG Num.seqs=34 Similarity=0.636128 tpcCG=45.099998 8 CCTCTTCGGTAGGTTCAGGAGTCA--TCGTAG----GCTCTGGAGTGA

arqueas_representative:NC_007796.1_Methanospirillum_hungatei_JF-1:91:1464547-1467421 Satlength=2875 Nr of Repeats=55 RepeatLength=42 seed=GTAGGTTCAG Num.seqs=42 Similarity=0.605731 tpcCG=45.099998 8 CCTCTTCGGTAGGTTCAGGAGTCANNTCGTAGCCTNNCTCNGGAGTGA

************************ ****** *** *******

Consensus:

CCTCTTCGGTAGGTTCAGGAGTCATCGTAGgCTCtGGAGTGA

>arqueas_representative_Fam_104_23_3 Nr. of seq. 3 Alignment length(with gaps) = 25 Alignment score = 0.622222

arqueas_representative:NZ_CP009506.1_Methanosarcina_siciliae_T4/M:86:5013982-5014120 Satlength=139 Nr of Repeats=6 RepeatLength=23 seed=GAAAAGTAAA Num.seqs=6 Similarity=0.911111 tpcCG=42.900002 0 GAAAAGTAAAGA-AGAGACAATAA-

arqueas_representative:NZ_CP009517.1_Methanosarcina_barkeri_3:81:4548729-4548993 Satlength=265 Nr of Repeats=11 RepeatLength=24 seed=AAGTAAAGAG Num.seqs=11 Similarity=0.989899 tpcCG=39.099998 3 GAAAAGTAAAGAGATAAACAAAGA-

arqueas_representative:NC_003552.1_Methanosarcina_acetivorans_str._C2A:80:2399064-2399254 Satlength=191 Nr of Repeats=8 RepeatLength=24 seed=AAATTAAGAA Num.seqs=6 Similarity=0.925926 tpcCG=42.700001 3 GAAAAATTAAGA-AGATAGAAAAAG

***** * **** * * * ** *

Consensus:

GAAAAgTaAAGAAgAnAcAAaaA

>arqueas_representative_Fam_105_21_3 Nr. of seq. 3 Alignment length(with gaps) = 24 Alignment score = 0.625000

arqueas_representative:NZ_CP014265.1_Methanobrevibacter_olleyae__YLM1:49:601494-601584 Satlength=91 Nr of Repeats=5 RepeatLength=20 seed=TATTTTTTAA Num.seqs=4 Similarity=0.933333 tpcCG=26.900000 0 TATTTTTTAA---TATAAGTTAA-

arqueas_representative:NZ_CP009517.1_Methanosarcina_barkeri_3:81:14942-15053 Satlength=112 Nr of Repeats=5 RepeatLength=22 seed=TAATTGTTTT Num.seqs=4 Similarity=1.000000 tpcCG=39.099998 18 TGTTTTTTAA--TTCTAAGTTAAT

Rev.of_arqueas_representative:NC_007681.1_Methanosphaera_stadtmanae_DSM_3091:89:1595064-1595225 Satlength=162 Nr of Repeats=7 RepeatLength=22 seed=ATAACTTATA Num.seqs=6 Similarity=1.000000 tpcCG=27.60 1 TTTGTTTTAACATTATAAGTTA--

* * ****** * *******

Consensus:

TnTtTTTTAAtTaTAAGTTAa

>arqueas_representative_Fam_106_19_3 Nr. of seq. 3 Alignment length(with gaps) = 22 Alignment score = 0.601010

arqueas_representative:NZ_CP009512.1_Methanosarcina_mazei_S-6:85:840934-841103 Satlength=170 Nr of Repeats=10 RepeatLength=17 seed=TTTTATATTT Num.seqs=9 Similarity=0.938998 tpcCG=41.400002 13 A-TATTTATTT---TAG-TTTT

arqueas_representative:NC_014222.1_Methanococcus_voltae_A3:65:912918-913013 Satlength=96 Nr of Repeats=5 RepeatLength=19 seed=TTTTATTATT Num.seqs=3 Similarity=1.000000 tpcCG=28.600000 25 ATTATTTATTT---TATATTTT

Rev.of_arqueas_representative:NZ_CP009515.1_Methanosarcina_lacustris_Z-7289:84:2805236-2805383 Satlength=148 Nr of Repeats=5 RepeatLength=21 seed=TATAAAACAC Num.seqs=3 Similarity=0.848485 tpcCG=41.799 7 TTTTA-TATTTCAGATTTTGTG

* ***** * *

Consensus:

atTattTATTTtatnTtTt

>arqueas_representative_Fam_107_18_3 Nr. of seq. 3 Alignment length(with gaps) = 21 Alignment score = 0.629630

arqueas_representative:NZ_CP009517.1_Methanosarcina_barkeri_3:81:4012690-4012860 Satlength=171 Nr of Repeats=10 RepeatLength=17 seed=AAAGAATAAC Num.seqs=10 Similarity=0.956427 tpcCG=39.099998 0 AAAG-AATAA-CTTCAAAA--

arqueas_representative:NZ_CP009515.1_Methanosarcina_lacustris_Z-7289:84:1190475-1190551 Satlength=77 Nr of Repeats=4 RepeatLength=19 seed=AAATAAAGAA Num.seqs=4 Similarity=1.000000 tpcCG=41.799999 15 AAAGAAATAA-CTTGAAAAT-

Rev.of_arqueas_representative:NZ_CP009528.1_Methanosarcina_barkeri_MS:82:2767814-2767983 Satlength=170 Nr of Repeats=7 RepeatLength=19 seed=TTTGAAGCTT Num.seqs=5 Similarity=1.000000 tpcCG=39.168999 16 -AAG-AGTAAGCTTCAAAATT

*** * *** *** ****

Consensus:

aAAGAaTAACTTcAAAAt

>arqueas_representative_Fam_108_19_3 Nr. of seq. 3 Alignment length(with gaps) = 21 Alignment score = 0.613757

arqueas_representative:NZ_CP009512.1_Methanosarcina_mazei_S-6:85:2664041-2664131 Satlength=91 Nr of Repeats=5 RepeatLength=18 seed=TTCGATCTCT Num.seqs=5 Similarity=0.896296 tpcCG=41.400002 0 TTCGATCTCTTATT--TTCT-

Rev.of_arqueas_representative:NZ_CP009528.1_Methanosarcina_barkeri_MS:82:3203992-3204626 Satlength=635 Nr of Repeats=10 RepeatLength=19 seed=ATTAAGAAAA Num.seqs=8 Similarity=1.000000 tpcCG=39.168999 14 TTAGTTTTCTTAAT--TTCTG

arqueas_representative:NZ_CP009501.1_Methanosarcina_thermophila_TM-1:87:1725974-1726058 Satlength=85 Nr of Repeats=4 RepeatLength=21 seed=TTCTTCTCTT Num.seqs=4 Similarity=0.936508 tpcCG=41.099998 34 TTTGTTTTCTTATTTCTTCTC

** * * ***** * ****

Consensus:

TTnGtTtTCTTAtTTTCTn

>arqueas_representative_Fam_109_17_3 Nr. of seq. 3 Alignment length(with gaps) = 20 Alignment score = 0.627778

arqueas_representative:NZ_CP009515.1_Methanosarcina_lacustris_Z-7289:84:1446443-1446630 Satlength=188 Nr of Repeats=11 RepeatLength=17 seed=TTTTTAAGAA Num.seqs=11 Similarity=0.934403 tpcCG=41.799999 0 TTTTTAAGAA-TTTATCA--

Rev.of_arqueas_representative:NC_003552.1_Methanosarcina_acetivorans_str._C2A:80:1711941-1712018 Satlength=78 Nr of Repeats=4 RepeatLength=19 seed=CTTGATAAAT Num.seqs=3 Similarity=0.833333 tpcCG=42.70 0 TATTTAAGAA-TTTATCAAG

Rev.of_arqueas_representative:NZ_CP009512.1_Methanosarcina_mazei_S-6:85:3861195-3861303 Satlength=109 Nr of Repeats=6 RepeatLength=18 seed=ATTAATAAAG Num.seqs=6 Similarity=0.925926 tpcCG=41.400002 2 ATTTTGAGAACTTTATTA--

*** **** ***** *

Consensus:

ttTTTaAGAATTTATcA

>arqueas_representative_Fam_110_20_3 Nr. of seq. 3 Alignment length(with gaps) = 20 Alignment score = 0.644444

arqueas_representative:NZ_CP009512.1_Methanosarcina_mazei_S-6:85:1833249-1833357 Satlength=109 Nr of Repeats=6 RepeatLength=18 seed=TTGAGATGGT Num.seqs=6 Similarity=1.000000 tpcCG=41.400002 0 TTGAGAT-G-GTAAATAAAA

arqueas_representative:NC_003552.1_Methanosarcina_acetivorans_str._C2A:80:3048405-3048485 Satlength=81 Nr of Repeats=4 RepeatLength=20 seed=AGAGTAAATA Num.seqs=4 Similarity=1.000000 tpcCG=42.700001 7 AAGAGATAGAGTAAATAAAA

Rev.of_arqueas_representative:NZ_CP009512.1_Methanosarcina_mazei_S-6:85:3256535-3256715 Satlength=181 Nr of Repeats=9 RepeatLength=20 seed=GTTTATGAAC Num.seqs=9 Similarity=1.000000 tpcCG=41.400002 20 AAGAGATAGAGTTCATAAAC

***** * ** *****

Consensus:

aaGAGATaGaGTaaATAAAa

>arqueas_representative_Fam_111_19_3 Nr. of seq. 3 Alignment length(with gaps) = 20 Alignment score = 0.755556

arqueas_representative:NC_003552.1_Methanosarcina_acetivorans_str._C2A:80:3952859-3953049 Satlength=191 Nr of Repeats=10 RepeatLength=19 seed=GAATCTAAAA Num.seqs=10 Similarity=0.942300 tpcCG=42.700001 0 GAATCTAAAAAAACGAATG-

arqueas_representative:NC_003552.1_Methanosarcina_acetivorans_str._C2A:80:3952861-3953336 Satlength=476 Nr of Repeats=11 RepeatLength=19 seed=ATCTAAAAAA Num.seqs=10 Similarity=0.942300 tpcCG=42.700001 2 GAATCTAAAAAAACGAATG-

Rev.of_arqueas_representative:NZ_CP009512.1_Methanosarcina_mazei_S-6:85:1533939-1534016 Satlength=78 Nr of Repeats=4 RepeatLength=19 seed=TTTCTTTTTT Num.seqs=3 Similarity=0.836257 tpcCG=41.400002 17 NATTCTAAAAAAA-GAAAGT

* ********** *** *

Consensus:

gAaTCTAAAAAAAcGAAtG

>arqueas_representative_Fam_112_16_3 Nr. of seq. 3 Alignment length(with gaps) = 19 Alignment score = 0.631579

arqueas_representative:NZ_CP009512.1_Methanosarcina_mazei_S-6:85:3552856-3552931 Satlength=76 Nr of Repeats=5 RepeatLength=15 seed=AACTTATTTT Num.seqs=5 Similarity=0.946667 tpcCG=41.400002 0 -AACTTA-TTT-TTTCAC-

arqueas_representative:NZ_CP009520.1_Methanosarcina_vacuolata_Z-761:88:4325227-4325313 Satlength=87 Nr of Repeats=5 RepeatLength=17 seed=TTACTTTATT Num.seqs=4 Similarity=1.000000 tpcCG=39.732498 3 -AACTTACTTTATTTCCC-

arqueas_representative:NZ_CP009517.1_Methanosarcina_barkeri_3:81:4559894-4560091 Satlength=198 Nr of Repeats=11 RepeatLength=18 seed=TAACTTCCTT Num.seqs=10 Similarity=0.958848 tpcCG=39.099998 0 TAACTTCCTTT-TTTCCCC

***** *** **** *

Consensus:

AACTTacTTTTTTCcC

>arqueas_representative_Fam_113_17_3 Nr. of seq. 3 Alignment length(with gaps) = 19 Alignment score = 0.654971

arqueas_representative:NC_003552.1_Methanosarcina_acetivorans_str._C2A:80:4182595-4182659 Satlength=65 Nr of Repeats=4 RepeatLength=16 seed=AAATTAAAAG Num.seqs=4 Similarity=1.000000 tpcCG=42.700001 0 AAATTAAAAGAGAGAT---

arqueas_representative:NZ_CP009512.1_Methanosarcina_mazei_S-6:85:2691306-2691374 Satlength=69 Nr of Repeats=4 RepeatLength=17 seed=AAATTAAAAA Num.seqs=4 Similarity=1.000000 tpcCG=41.400002 0 AAATTAAAAAAGAGAAG--

Rev.of_arqueas_representative:NZ_CP009501.1_Methanosarcina_thermophila_TM-1:87:641212-641818 Satlength=607 Nr of Repeats=6 RepeatLength=17 seed=TTTTAATTTT Num.seqs=4 Similarity=0.726608 tpcCG=41.09999 9 AAATTAAAAGA-AGATTAA

********* * ***

Consensus:

AAATTAAAAgAgAGAtn

>arqueas_representative_Fam_114_17_3 Nr. of seq. 3 Alignment length(with gaps) = 19 Alignment score = 0.649123

arqueas_representative:NZ_CP009512.1_Methanosarcina_mazei_S-6:85:3970649-3970721 Satlength=73 Nr of Repeats=4 RepeatLength=16 seed=AAAGAAACAA Num.seqs=3 Similarity=0.944444 tpcCG=41.400002 0 AAAGAAAC-AAAAACAC--

arqueas_representative:NC_013790.1_Methanobrevibacter_ruminantium_M1:50:1905293-1905378 Satlength=86 Nr of Repeats=4 RepeatLength=17 seed=AAACTAAAAA Num.seqs=3 Similarity=0.895425 tpcCG=32.599998 4 AAAGAAACTAAAAACTC--

Rev.of_arqueas_representative:NZ_CP009517.1_Methanosarcina_barkeri_3:81:705292-705436 Satlength=145 Nr of Repeats=8 RepeatLength=18 seed=AGTTGTTTCT Num.seqs=8 Similarity=0.968254 tpcCG=39.099998 8 AAA-CAACTAAAAACACAG

*** *** ****** *

Consensus:

AAAgaAACtAAAAACaC

>arqueas_representative_Fam_115_18_3 Nr. of seq. 3 Alignment length(with gaps) = 19 Alignment score = 0.684211

arqueas_representative:NZ_CP009528.1_Methanosarcina_barkeri_MS:82:3566983-3567170 Satlength=188 Nr of Repeats=11 RepeatLength=17 seed=TTATATTGGG Num.seqs=11 Similarity=0.920143 tpcCG=39.168999 0 TTATA-TTGGGTCTTATC-

arqueas_representative:NZ_CP009528.1_Methanosarcina_barkeri_MS:82:536737-536845 Satlength=109 Nr of Repeats=6 RepeatLength=18 seed=ATCTTAATAT Num.seqs=6 Similarity=1.000000 tpcCG=39.168999 14 TAATA-TTTGGACTTATCT

arqueas_representative:NZ_CP009515.1_Methanosarcina_lacustris_Z-7289:84:119120-119289 Satlength=170 Nr of Repeats=7 RepeatLength=19 seed=ACTTACCTTA Num.seqs=6 Similarity=1.000000 tpcCG=41.799999 29 TAATATTTTGGACTTACCT

* *** ** ** **** *

Consensus:

TaATATTtGGaCTTAtCt

>arqueas_representative_Fam_116_19_3 Nr. of seq. 3 Alignment length(with gaps) = 19 Alignment score = 0.602339

arqueas_representative:NZ_CP009501.1_Methanosarcina_thermophila_TM-1:87:2901404-2901472 Satlength=69 Nr of Repeats=4 RepeatLength=17 seed=AGAGTAATAC Num.seqs=4 Similarity=1.000000 tpcCG=41.099998 0 AG--AGTAATACTTGTTAA

Rev.of_arqueas_representative:NZ_CP009517.1_Methanosarcina_barkeri_3:81:1443310-1443386 Satlength=77 Nr of Repeats=4 RepeatLength=19 seed=TTTGCTTTAT Num.seqs=4 Similarity=0.953216 tpcCG=39.099998 6 AGCAAATAATACCTGATAA

arqueas_representative:NZ_CP009517.1_Methanosarcina_barkeri_3:81:3981516-3981585 Satlength=70 Nr of Repeats=4 RepeatLength=19 seed=AATACCTGTT Num.seqs=3 Similarity=1.000000 tpcCG=39.099998 7 ACCAGGGAATACCTGTTAA

* ***** ** ***

Consensus:

AgcaagtAATACcTGtTAA

>arqueas_representative_Fam_117_19_3 Nr. of seq. 3 Alignment length(with gaps) = 19 Alignment score = 0.789474

arqueas_representative:NC_013790.1_Methanobrevibacter_ruminantium_M1:50:318393-318488 Satlength=96 Nr of Repeats=5 RepeatLength=19 seed=TCAATTTTAC Num.seqs=5 Similarity=0.547619 tpcCG=32.599998 0 TCAATTTTACA-TTTNNTA

arqueas_representative:NC_013790.1_Methanobrevibacter_ruminantium_M1:50:707384-707555 Satlength=172 Nr of Repeats=7 RepeatLength=19 seed=ATATCAATTT Num.seqs=5 Similarity=1.000000 tpcCG=32.599998 16 TCAATTTTACACTTTCATA

arqueas_representative:NC_013790.1_Methanobrevibacter_ruminantium_M1:50:1612577-1612672 Satlength=96 Nr of Repeats=4 RepeatLength=19 seed=TCAATTTCAC Num.seqs=3 Similarity=0.906433 tpcCG=32.599998 19 TCAATTTCACATTTTCATA

******* *** *** **

Consensus:

TCAATTTtACAnTTTcaTA

>arqueas_representative_Fam_118_18_3 Nr. of seq. 3 Alignment length(with gaps) = 19 Alignment score = 0.725146

arqueas_representative:NZ_CP009517.1_Methanosarcina_barkeri_3:81:4510691-4510811 Satlength=121 Nr of Repeats=6 RepeatLength=18 seed=AGAATAAAAA Num.seqs=4 Similarity=0.950617 tpcCG=39.099998 16 AATAAAAAAGGGAAAAA-G

Rev.of_arqueas_representative:NZ_CP009515.1_Methanosarcina_lacustris_Z-7289:84:2692691-2692837 Satlength=147 Nr of Repeats=8 RepeatLength=18 seed=TTCCTTTTTT Num.seqs=6 Similarity=1.000000 tpcCG=41.799 31 GATAAAAAAGGAAGAAA-G

Rev.of_arqueas_representative:NZ_CP009515.1_Methanosarcina_lacustris_Z-7289:84:449318-449409 Satlength=92 Nr of Repeats=5 RepeatLength=19 seed=CTTTCTTCCT Num.seqs=3 Similarity=0.953216 tpcCG=41.799999 36 GAAAAAAAAGGAAGAAAGG

* * * ******** * **

Consensus:

AGgAtAAAAAAGGaAgAA

>arqueas_representative_Fam_119_18_3 Nr. of seq. 3 Alignment length(with gaps) = 19 Alignment score = 0.678363

arqueas_representative:NZ_CP009528.1_Methanosarcina_barkeri_MS:82:942387-942479 Satlength=93 Nr of Repeats=5 RepeatLength=18 seed=TAATCTGATT Num.seqs=3 Similarity=0.950617 tpcCG=39.168999 0 TAATCTA-ATTGATCGCTA

arqueas_representative:NZ_CP009528.1_Methanosarcina_barkeri_MS:82:2637413-2637520 Satlength=108 Nr of Repeats=6 RepeatLength=18 seed=TTAATCTCAT Num.seqs=5 Similarity=0.911111 tpcCG=39.168999 17 TAATCTC-ATTGATTTCTT

arqueas_representative:NZ_CP009517.1_Methanosarcina_barkeri_3:81:1194214-1194307 Satlength=94 Nr of Repeats=5 RepeatLength=19 seed=CTCAATTGAT Num.seqs=4 Similarity=0.894737 tpcCG=39.099998 22 TAATCTCAATTGATTTCAT

****** ****** *

Consensus:

TAATCTcATTGATttCtt

>arqueas_representative_Fam_120_18_3 Nr. of seq. 3 Alignment length(with gaps) = 19 Alignment score = 0.631579

arqueas_representative:NZ_CP009528.1_Methanosarcina_barkeri_MS:82:1676790-1676862 Satlength=73 Nr of Repeats=4 RepeatLength=18 seed=TTTTGTTTTA Num.seqs=4 Similarity=0.962963 tpcCG=39.168999 0 TTTTGTT-TTAACTATTAT

arqueas_representative:NZ_CP009512.1_Methanosarcina_mazei_S-6:85:974778-975042 Satlength=265 Nr of Repeats=14 RepeatLength=19 seed=ATTTTTCTTC Num.seqs=12 Similarity=0.964912 tpcCG=41.400002 17 TTTTCTTCTTTACTATTAT

Rev.of_arqueas_representative:NZ_CP009515.1_Methanosarcina_lacustris_Z-7289:84:732787-732931 Satlength=145 Nr of Repeats=8 RepeatLength=18 seed=TTTTTAAAAG Num.seqs=8 Similarity=0.830688 tpcCG=41.79999 14 TTTTCTT-TTAAAAATCGT

**** ** ** * ** *

Consensus:

TTTTcTTTTaActATtaT

>arqueas_representative_Fam_121_18_3 Nr. of seq. 3 Alignment length(with gaps) = 19 Alignment score = 0.608187

arqueas_representative:NZ_CP009512.1_Methanosarcina_mazei_S-6:85:626777-626921 Satlength=145 Nr of Repeats=8 RepeatLength=18 seed=AAAATGAATT Num.seqs=8 Similarity=0.968254 tpcCG=41.400002 0 AAAATGAATTG-GTTCTTT

Rev.of_arqueas_representative:NZ_CP009512.1_Methanosarcina_mazei_S-6:85:2299904-2300024 Satlength=121 Nr of Repeats=5 RepeatLength=18 seed=TAATTTATTT Num.seqs=4 Similarity=0.901235 tpcCG=41.400002 11 AAAATAAATTA-GATATTT

arqueas_representative:NC_015574.1_Methanobacterium_paludis__SWAN1:47:1296783-1297448 Satlength=666 Nr of Repeats=35 RepeatLength=19 seed=ATTAGGGGAT Num.seqs=35 Similarity=0.991980 tpcCG=35.700001 26 AAGATAAATTAGGGGATTT

** ** **** * ***

Consensus:

AAaATaAATTaGntaTTT

>arqueas_representative_Fam_122_15_3 Nr. of seq. 3 Alignment length(with gaps) = 18 Alignment score = 0.617284

arqueas_representative:NC_008212.1_Haloquadratum_walsbyi_DSM_16790:31:2979593-2979653 Satlength=61 Nr of Repeats=4 RepeatLength=15 seed=CATCGCCACC Num.seqs=4 Similarity=0.940741 tpcCG=47.694099 0 CATCGCCACCGTCAC---

Rev.of_arqueas_representative:NC_013922.1_Natrialba_magadii_ATCC_43099:100:904249-904399 Satlength=151 Nr of Repeats=10 RepeatLength=15 seed=CTGGTGTCGG Num.seqs=10 Similarity=0.952593 tpcCG=61.032001 3 CAGCGCTACCGACAC---

arqueas_representative:NC_012029.1_Halorubrum_lacusprofundi_ATCC_49239_chromosome_1:34:2326887-2326959 Satlength=73 Nr of Repeats=4 RepeatLength=18 seed=TCGCTTCCGT Num.seqs=4 Similarity=0.839506 tpcCG 2 CATCGCTTCCGTCACTGC

** *** *** ***

Consensus:

CAtCGCtaCCGtCAC

>arqueas_representative_Fam_123_17_3 Nr. of seq. 3 Alignment length(with gaps) = 18 Alignment score = 0.629630

arqueas_representative:NZ_CP009520.1_Methanosarcina_vacuolata_Z-761:88:715748-715823 Satlength=76 Nr of Repeats=5 RepeatLength=15 seed=CTCAGTATTC Num.seqs=5 Similarity=0.946667 tpcCG=39.732498 0 CTCAGTATTCTGTAT---

arqueas_representative:NZ_CP009512.1_Methanosarcina_mazei_S-6:85:879910-879974 Satlength=65 Nr of Repeats=4 RepeatLength=16 seed=TTTGTTTTCC Num.seqs=4 Similarity=1.000000 tpcCG=41.400002 7 CTCAGTATT-TGTTTTC-

arqueas_representative:NZ_CP009520.1_Methanosarcina_vacuolata_Z-761:88:2510467-2510581 Satlength=115 Nr of Repeats=5 RepeatLength=18 seed=TTCTCTATTC Num.seqs=4 Similarity=1.000000 tpcCG=39.732498 7 TTCAGTATTCTCTATTCC

******** * * *

Consensus:

cTCAGTATTcTgTaTtc

>arqueas_representative_Fam_124_16_3 Nr. of seq. 3 Alignment length(with gaps) = 18 Alignment score = 0.691358

arqueas_representative:NZ_CP009520.1_Methanosarcina_vacuolata_Z-761:88:1805610-1805674 Satlength=65 Nr of Repeats=4 RepeatLength=16 seed=AAAAATGAAG Num.seqs=4 Similarity=1.000000 tpcCG=39.732498 0 AAAAATGAA--GAATACG

arqueas_representative:NZ_CP009520.1_Methanosarcina_vacuolata_Z-761:88:2312643-2312771 Satlength=129 Nr of Repeats=8 RepeatLength=16 seed=AAATGCAGAA Num.seqs=8 Similarity=1.000000 tpcCG=39.732498 2 AAAAATGCA--GAAAATG

arqueas_representative:NC_003552.1_Methanosarcina_acetivorans_str._C2A:80:3389629-3389776 Satlength=148 Nr of Repeats=8 RepeatLength=18 seed=TGAACTGAAA Num.seqs=5 Similarity=1.000000 tpcCG=42.700001 5 AAAAATGAACTGAAAACG

******* * *** * *

Consensus:

AAAAATGaAGAAaAcG

>arqueas_representative_Fam_125_18_3 Nr. of seq. 3 Alignment length(with gaps) = 18 Alignment score = 0.641975

arqueas_representative:NZ_CP009520.1_Methanosarcina_vacuolata_Z-761:88:4293885-4293997 Satlength=113 Nr of Repeats=7 RepeatLength=16 seed=TCCAGTATTT Num.seqs=7 Similarity=0.920635 tpcCG=39.732498 0 TCCAGTATTTTCTTTT--

Rev.of_arqueas_representative:NZ_CP009520.1_Methanosarcina_vacuolata_Z-761:88:4201057-4201142 Satlength=86 Nr of Repeats=5 RepeatLength=17 seed=AATACTGGAT Num.seqs=5 Similarity=0.890196 tpcCG=39.73249 9 TCCAGTA-TTTCTTTTAA

Rev.of_arqueas_representative:NC_003552.1_Methanosarcina_acetivorans_str._C2A:80:1726505-1726739 Satlength=235 Nr of Repeats=13 RepeatLength=18 seed=GGAAGAAAAG Num.seqs=13 Similarity=0.965812 tpcCG=42 3 TCCTGTATACTCTTTTCT

*** *** ******

Consensus:

TCCaGTAtttTCTTTTnn

>arqueas_representative_Fam_126_17_3 Nr. of seq. 3 Alignment length(with gaps) = 18 Alignment score = 0.611111

arqueas_representative:NZ_CP009517.1_Methanosarcina_barkeri_3:81:3451767-3452514 Satlength=748 Nr of Repeats=7 RepeatLength=17 seed=AGATTTTAAG Num.seqs=5 Similarity=1.000000 tpcCG=39.099998 0 A-GATTTTAAGCTACAAC

Rev.of_arqueas_representative:NZ_CP009520.1_Methanosarcina_vacuolata_Z-761:88:97124-97209 Satlength=86 Nr of Repeats=5 RepeatLength=17 seed=TGCTGTAAAT Num.seqs=5 Similarity=0.968627 tpcCG=39.732498 1 A-AATTTTCATTTACAGC

Rev.of_arqueas_representative:NZ_CP009528.1_Methanosarcina_barkeri_MS:82:4530842-4531003 Satlength=162 Nr of Repeats=9 RepeatLength=18 seed=TGTAAATTAA Num.seqs=8 Similarity=0.920635 tpcCG=39.168999 16 AGAACTTTAATTTACAAC

* * *** * **** *

Consensus:

AaAtTTTaAttTACAaC

>arqueas_representative_Fam_127_17_3 Nr. of seq. 3 Alignment length(with gaps) = 18 Alignment score = 0.611111

arqueas_representative:NZ_CP009528.1_Methanosarcina_barkeri_MS:82:1837034-1837187 Satlength=154 Nr of Repeats=9 RepeatLength=17 seed=TATAATTTCA Num.seqs=9 Similarity=1.000000 tpcCG=39.168999 0 TA-TAATTTCAAGAATAG

Rev.of_arqueas_representative:NZ_CP009520.1_Methanosarcina_vacuolata_Z-761:88:647504-647605 Satlength=102 Nr of Repeats=6 RepeatLength=17 seed=TAATATTGTT Num.seqs=5 Similarity=0.937255 tpcCG=39.732498 2 TA-TGCTTTCAACAATAT

Rev.of_arqueas_representative:NZ_CP009512.1_Methanosarcina_mazei_S-6:85:1713684-1713756 Satlength=73 Nr of Repeats=4 RepeatLength=18 seed=AAATAGTATT Num.seqs=4 Similarity=0.839506 tpcCG=41.400002 8 TACTATTTTCAAGAAGAA

** * ****** ** *

Consensus:

TATanTTTCAAgAAtAn

>arqueas_representative_Fam_128_17_3 Nr. of seq. 3 Alignment length(with gaps) = 18 Alignment score = 0.635802

arqueas_representative:NZ_CP009512.1_Methanosarcina_mazei_S-6:85:3488784-3489589 Satlength=806 Nr of Repeats=5 RepeatLength=17 seed=ATTTTTGCTC Num.seqs=4 Similarity=0.960784 tpcCG=41.400002 0 ATTTTTGCTCTCT-TCTT

arqueas_representative:NZ_CP009506.1_Methanosarcina_siciliae_T4/M:86:4828028-4828113 Satlength=86 Nr of Repeats=5 RepeatLength=17 seed=TCGTATTTTT Num.seqs=5 Similarity=1.000000 tpcCG=42.900002 13 ATTTTTGTTCTTA-TCGT

arqueas_representative:NC_014222.1_Methanococcus_voltae_A3:65:503496-503640 Satlength=145 Nr of Repeats=8 RepeatLength=18 seed=TTGTTCTGAT Num.seqs=8 Similarity=1.000000 tpcCG=28.600000 21 ATTATTGTTCTGATTCTT

*** *** *** ** *

Consensus:

ATTtTTGtTCTnaTCtT

>arqueas_representative_Fam_129_18_3 Nr. of seq. 3 Alignment length(with gaps) = 18 Alignment score = 0.802469

arqueas_representative:NC_003552.1_Methanosarcina_acetivorans_str._C2A:80:1625497-1625641 Satlength=145 Nr of Repeats=8 RepeatLength=18 seed=CTGCAACCGA Num.seqs=8 Similarity=0.846561 tpcCG=42.700001 0 CTGCAACCGAAGAGATGA

arqueas_representative:NZ_CP009506.1_Methanosarcina_siciliae_T4/M:86:3447780-3447906 Satlength=127 Nr of Repeats=7 RepeatLength=18 seed=CTGCAACCGA Num.seqs=7 Similarity=0.880071 tpcCG=42.900002 0 CTGCAACCGAAGAGATGA

arqueas_representative:NZ_CP009515.1_Methanosarcina_lacustris_Z-7289:84:824242-824440 Satlength=199 Nr of Repeats=11 RepeatLength=18 seed=CTCCAACTGA Num.seqs=11 Similarity=0.862626 tpcCG=41.799999 0 CTCCAACTGAAGGAATGA

** **** **** ****

Consensus:

CTgCAACcGAAGagATGA

>arqueas_representative_Fam_130_15_3 Nr. of seq. 3 Alignment length(with gaps) = 17 Alignment score = 0.627451

arqueas_representative:NZ_CP014265.1_Methanobrevibacter_olleyae__YLM1:49:425811-425901 Satlength=91 Nr of Repeats=5 RepeatLength=15 seed=AAATTAGTTT Num.seqs=4 Similarity=0.837037 tpcCG=26.900000 0 AAATTAGTT-TTATCT-

Rev.of_arqueas_representative:NZ_CP009517.1_Methanosarcina_barkeri_3:81:84391-84451 Satlength=61 Nr of Repeats=4 RepeatLength=15 seed=GTAGATCAAA Num.seqs=4 Similarity=1.000000 tpcCG=39.099998 2 ACATTATTT-TGATCT-

arqueas_representative:NZ_CP009528.1_Methanosarcina_barkeri_MS:82:2376792-2376894 Satlength=103 Nr of Repeats=6 RepeatLength=17 seed=TATTATCAAA Num.seqs=6 Similarity=1.000000 tpcCG=39.168999 8 AAATTATTTATTATCAA

* **** ** * ***

Consensus:

AaATTAtTTTtATCt

>arqueas_representative_Fam_131_16_3 Nr. of seq. 3 Alignment length(with gaps) = 17 Alignment score = 0.624183

arqueas_representative:NC_013790.1_Methanobrevibacter_ruminantium_M1:50:2677989-2678094 Satlength=106 Nr of Repeats=7 RepeatLength=15 seed=AACTTTAAGT Num.seqs=7 Similarity=1.000000 tpcCG=32.599998 0 AACTTTAAGTTGGG--G

arqueas_representative:NZ_CP009506.1_Methanosarcina_siciliae_T4/M:86:2675291-2675351 Satlength=61 Nr of Repeats=4 RepeatLength=15 seed=AACTTTAAGT Num.seqs=4 Similarity=0.896296 tpcCG=42.900002 0 AACTTTAAGTAACG-A-

Rev.of_arqueas_representative:NZ_CP009517.1_Methanosarcina_barkeri_3:81:4557947-4558029 Satlength=83 Nr of Repeats=5 RepeatLength=17 seed=CTGCTTTACT Num.seqs=4 Similarity=0.960784 tpcCG=39.099998 0 AACTTTAAGTAAAGCAG

********** *

Consensus:

AACTTTAAGTaanGag

>arqueas_representative_Fam_132_17_3 Nr. of seq. 3 Alignment length(with gaps) = 17 Alignment score = 0.647059

arqueas_representative:NZ_CP009517.1_Methanosarcina_barkeri_3:81:454946-455019 Satlength=74 Nr of Repeats=5 RepeatLength=15 seed=AAATTGATCT Num.seqs=3 Similarity=0.940741 tpcCG=39.099998 0 AAATTGATCTTGATA--

arqueas_representative:NZ_CP009512.1_Methanosarcina_mazei_S-6:85:676421-676541 Satlength=121 Nr of Repeats=7 RepeatLength=17 seed=GATCTTGAAA Num.seqs=6 Similarity=1.000000 tpcCG=41.400002 5 AAGATGATCTTGAAATA

Rev.of_arqueas_representative:NZ_CP009520.1_Methanosarcina_vacuolata_Z-761:88:2559158-2559226 Satlength=69 Nr of Repeats=4 RepeatLength=17 seed=TTGATTTAAA Num.seqs=4 Similarity=0.882353 tpcCG=39.73249 19 AATATGATCTTTAAATC

** ******* * *

Consensus:

AAnaTGATCTTgAaAtn

>arqueas_representative_Fam_133_15_3 Nr. of seq. 3 Alignment length(with gaps) = 17 Alignment score = 0.679739

arqueas_representative:NZ_CP009517.1_Methanosarcina_barkeri_3:81:2435071-2435161 Satlength=91 Nr of Repeats=6 RepeatLength=15 seed=TTACTTAGTT Num.seqs=6 Similarity=0.970370 tpcCG=39.099998 0 TTAC-TTAGTTTGAGA-

Rev.of_arqueas_representative:NZ_CP009517.1_Methanosarcina_barkeri_3:81:1524366-1524468 Satlength=103 Nr of Repeats=6 RepeatLength=17 seed=TAACTCTGAA Num.seqs=6 Similarity=0.947712 tpcCG=39.099998 3 TTACATTAGTTTCAGAG

Rev.of_arqueas_representative:NZ_CP009520.1_Methanosarcina_vacuolata_Z-761:88:1864813-1864888 Satlength=76 Nr of Repeats=5 RepeatLength=15 seed=AACAAAGTGA Num.seqs=5 Similarity=1.000000 tpcCG=39.73249 10 TCAC-TTTGTTTCAGA-

* ** ** **** ***

Consensus:

TtACTTaGTTTcAGA

>arqueas_representative_Fam_134_16_3 Nr. of seq. 3 Alignment length(with gaps) = 17 Alignment score = 0.653595

arqueas_representative:NZ_CP009528.1_Methanosarcina_barkeri_MS:82:1220184-1220274 Satlength=91 Nr of Repeats=6 RepeatLength=15 seed=TTTAAGAGAA Num.seqs=6 Similarity=0.840000 tpcCG=39.168999 0 --TTTAAGAGAACTGAG

Rev.of_arqueas_representative:NZ_CP009520.1_Methanosarcina_vacuolata_Z-761:88:1810705-1810795 Satlength=91 Nr of Repeats=6 RepeatLength=15 seed=ACTCATTTCT Num.seqs=6 Similarity=0.899259 tpcCG=39.73249 1 TTTATAAGA-AA-TGAG

arqueas_representative:NZ_CP009520.1_Methanosarcina_vacuolata_Z-761:88:2915497-2915577 Satlength=81 Nr of Repeats=5 RepeatLength=16 seed=TTAAGAAATT Num.seqs=5 Similarity=0.900000 tpcCG=39.732498 3 TTTTTAAGA-AATTGAG

* ***** ** ****

Consensus:

ttTtTAAGAAAnTGAG

>arqueas_representative_Fam_135_15_3 Nr. of seq. 3 Alignment length(with gaps) = 17 Alignment score = 0.738562

arqueas_representative:NZ_CP009528.1_Methanosarcina_barkeri_MS:82:2724498-2724601 Satlength=104 Nr of Repeats=7 RepeatLength=15 seed=AAAATCAATT Num.seqs=6 Similarity=1.000000 tpcCG=39.168999 0 AAAATCAATTACACT--

Rev.of_arqueas_representative:NZ_CP009520.1_Methanosarcina_vacuolata_Z-761:88:2482647-2482797 Satlength=151 Nr of Repeats=10 RepeatLength=15 seed=ATTGATTTTA Num.seqs=10 Similarity=0.964444 tpcCG=39.73 9 AAAATCAATCACATT--

arqueas_representative:NZ_CP009528.1_Methanosarcina_barkeri_MS:82:1637611-1637882 Satlength=272 Nr of Repeats=16 RepeatLength=17 seed=AAAATCAATT Num.seqs=15 Similarity=0.961158 tpcCG=39.168999 0 AAAATCAATTACAGTAA

********* *** *

Consensus:

AAAATCAATtACAnT

>arqueas_representative_Fam_136_15_3 Nr. of seq. 3 Alignment length(with gaps) = 17 Alignment score = 0.627451

arqueas_representative:NZ_CP009515.1_Methanosarcina_lacustris_Z-7289:84:459551-459626 Satlength=76 Nr of Repeats=5 RepeatLength=15 seed=ACGTTTTCAT Num.seqs=5 Similarity=0.964444 tpcCG=41.799999 0 ACGTTTTC-ATCA-GTG

Rev.of_arqueas_representative:NZ_CP009520.1_Methanosarcina_vacuolata_Z-761:88:141386-141446 Satlength=61 Nr of Repeats=4 RepeatLength=15 seed=AAATGAGACT Num.seqs=4 Similarity=1.000000 tpcCG=39.732498 6 TCATTTTC-ATCA-GTC

Rev.of_arqueas_representative:NZ_CP009520.1_Methanosarcina_vacuolata_Z-761:88:605012-605076 Satlength=65 Nr of Repeats=4 RepeatLength=16 seed=GCACATGATT Num.seqs=4 Similarity=0.916667 tpcCG=39.732498 1 -CATTTTCAATCATGTG

* ***** **** **

Consensus:

nCaTTTTCATCAGTg

>arqueas_representative_Fam_137_15_3 Nr. of seq. 3 Alignment length(with gaps) = 17 Alignment score = 0.607843

arqueas_representative:NZ_CP009512.1_Methanosarcina_mazei_S-6:85:1675831-1675906 Satlength=76 Nr of Repeats=5 RepeatLength=15 seed=TTAAACAAAA Num.seqs=5 Similarity=0.795556 tpcCG=41.400002 0 -TTAAACAAAATTGCA-

arqueas_representative:NZ_CP009520.1_Methanosarcina_vacuolata_Z-761:88:458635-458795 Satlength=161 Nr of Repeats=10 RepeatLength=16 seed=ATTAAACAAA Num.seqs=10 Similarity=1.000000 tpcCG=39.732498 0 ATTAAACAAAATAGCC-

Rev.of_arqueas_representative:NZ_CP009515.1_Methanosarcina_lacustris_Z-7289:84:3439996-3440172 Satlength=177 Nr of Repeats=11 RepeatLength=16 seed=TCTGTTTAAA Num.seqs=11 Similarity=0.954545 tpcCG=41.7 9 -TTAAACAGAAGTACAT

******* ** *

Consensus:

TTAAACAaAAttgCa

>arqueas_representative_Fam_138_15_3 Nr. of seq. 3 Alignment length(with gaps) = 17 Alignment score = 0.660131

arqueas_representative:NZ_CP009506.1_Methanosarcina_siciliae_T4/M:86:1501229-1501304 Satlength=76 Nr of Repeats=5 RepeatLength=15 seed=ACTGAAAATA Num.seqs=5 Similarity=0.964444 tpcCG=42.900002 0 ACTGAAAATAACACA--

Rev.of_arqueas_representative:NZ_CP009520.1_Methanosarcina_vacuolata_Z-761:88:3756342-3757158 Satlength=817 Nr of Repeats=6 RepeatLength=15 seed=TCATTTGTCT Num.seqs=5 Similarity=1.000000 tpcCG=39.7324 5 AATGAAAATAAGACA--

Rev.of_arqueas_representative:NC_013790.1_Methanobrevibacter_ruminantium_M1:50:1434791-1435015 Satlength=225 Nr of Repeats=12 RepeatLength=16 seed=TTTTGTGTTT Num.seqs=8 Similarity=0.746849 tpcCG=32.59 1 AGTGAAAA-AACACAAA

* ****** ** ***

Consensus:

AnTGAAAAtAAcACA

>arqueas_representative_Fam_139_16_3 Nr. of seq. 3 Alignment length(with gaps) = 17 Alignment score = 0.653595

arqueas_representative:NZ_CP009506.1_Methanosarcina_siciliae_T4/M:86:2827684-2828081 Satlength=398 Nr of Repeats=4 RepeatLength=15 seed=TTAAAGGTTT Num.seqs=3 Similarity=1.000000 tpcCG=42.900002 0 -TTAAAGGTTTATTTT-

arqueas_representative:NZ_CP009528.1_Methanosarcina_barkeri_MS:82:4321999-4322079 Satlength=81 Nr of Repeats=5 RepeatLength=16 seed=TTATATGTTT Num.seqs=5 Similarity=1.000000 tpcCG=39.168999 0 -TTATATGTTTATTTTT

arqueas_representative:NZ_CP009517.1_Methanosarcina_barkeri_3:81:19842-19907 Satlength=66 Nr of Repeats=4 RepeatLength=17 seed=TTTAATTTTC Num.seqs=3 Similarity=1.000000 tpcCG=39.099998 8 CTAATAAGTTTAATTTT

* * * ***** ***

Consensus:

TtAtAnGTTTAtTTTt

>arqueas_representative_Fam_140_16_3 Nr. of seq. 3 Alignment length(with gaps) = 17 Alignment score = 0.725490

arqueas_representative:NZ_CP009520.1_Methanosarcina_vacuolata_Z-761:88:450228-450348 Satlength=121 Nr of Repeats=8 RepeatLength=15 seed=TTGTTTTATC Num.seqs=8 Similarity=1.000000 tpcCG=39.732498 0 TT-GTTTTATCCTGAT-

Rev.of_arqueas_representative:NZ_CP009528.1_Methanosarcina_barkeri_MS:82:3493454-3493679 Satlength=226 Nr of Repeats=14 RepeatLength=16 seed=AATATCAGGA Num.seqs=13 Similarity=1.000000 tpcCG=39.168999 2 TTCATTTT-TCCTGATA

Rev.of_arqueas_representative:NZ_CP009520.1_Methanosarcina_vacuolata_Z-761:88:786719-787688 Satlength=970 Nr of Repeats=23 RepeatLength=16 seed=TATCAGGAAA Num.seqs=22 Similarity=0.992424 tpcCG=39.7324 16 TTCGTTTT-TCCTGATA

** **** *******

Consensus:

TTcgTTTTTCCTGATa

>arqueas_representative_Fam_141_17_3 Nr. of seq. 3 Alignment length(with gaps) = 17 Alignment score = 0.614379

arqueas_representative:NZ_CP009517.1_Methanosarcina_barkeri_3:81:3399426-3399724 Satlength=299 Nr of Repeats=19 RepeatLength=16 seed=TTCTACTCAG Num.seqs=13 Similarity=1.000000 tpcCG=39.099998 0 TTCTACTCAGTTTTTA-

arqueas_representative:NZ_CP009515.1_Methanosarcina_lacustris_Z-7289:84:4048613-4048732 Satlength=120 Nr of Repeats=7 RepeatLength=17 seed=TATTTAATTA Num.seqs=7 Similarity=0.828198 tpcCG=41.799999 10 TTATACTGAGTATTTAA

arqueas_representative:NZ_CP009512.1_Methanosarcina_mazei_S-6:85:538791-538982 Satlength=192 Nr of Repeats=6 RepeatLength=16 seed=TTTTAATTTT Num.seqs=4 Similarity=1.000000 tpcCG=41.400002 10 TTTTCCTGGGT-TTTAA

** * ** ** ****

Consensus:

TTnTaCTgaGTnTTTAa

>arqueas_representative_Fam_142_16_3 Nr. of seq. 3 Alignment length(with gaps) = 17 Alignment score = 0.699346

arqueas_representative:NZ_CP009528.1_Methanosarcina_barkeri_MS:82:2735812-2735884 Satlength=73 Nr of Repeats=4 RepeatLength=16 seed=AGCTATTCAG Num.seqs=3 Similarity=1.000000 tpcCG=39.168999 0 AGCTATTCAGCTATTC-

arqueas_representative:NZ_CP009528.1_Methanosarcina_barkeri_MS:82:2735887-2736071 Satlength=185 Nr of Repeats=11 RepeatLength=16 seed=TATTCAGTTA Num.seqs=10 Similarity=1.000000 tpcCG=39.168999 3 AGTTATTCAGTTATTC-

Rev.of_arqueas_representative:NZ_CP009520.1_Methanosarcina_vacuolata_Z-761:88:2955224-2955292 Satlength=69 Nr of Repeats=4 RepeatLength=17 seed=AATATCTAGA Num.seqs=4 Similarity=0.869281 tpcCG=39.73249 7 AGATATTCAGCTTGTCT

** ******* * **

Consensus:

AGnTATTCAGcTatTC

>arqueas_representative_Fam_143_17_3 Nr. of seq. 3 Alignment length(with gaps) = 17 Alignment score = 0.607843

arqueas_representative:NZ_CP009520.1_Methanosarcina_vacuolata_Z-761:88:605012-605076 Satlength=65 Nr of Repeats=4 RepeatLength=16 seed=GCACATGATT Num.seqs=4 Similarity=0.916667 tpcCG=39.732498 0 GCA-CATGATTGAAAAT

Rev.of_arqueas_representative:NZ_CP009520.1_Methanosarcina_vacuolata_Z-761:88:3338340-3338612 Satlength=273 Nr of Repeats=17 RepeatLength=16 seed=AGTTTCAATC Num.seqs=17 Similarity=0.968137 tpcCG=39.73 0 G-ATTATGATTGAAACT

Rev.of_arqueas_representative:NZ_CP009517.1_Methanosarcina_barkeri_3:81:2872464-2872566 Satlength=103 Nr of Repeats=6 RepeatLength=17 seed=TGTTTCAATT Num.seqs=6 Similarity=0.947712 tpcCG=39.099998 0 GAATTATAATTGAAACA

* * ** *******

Consensus:

GnAttATgATTGAAAct

>arqueas_representative_Fam_144_17_3 Nr. of seq. 3 Alignment length(with gaps) = 17 Alignment score = 0.692810

arqueas_representative:NZ_CP009520.1_Methanosarcina_vacuolata_Z-761:88:1552628-1552708 Satlength=81 Nr of Repeats=5 RepeatLength=16 seed=GCTTTAATCT Num.seqs=5 Similarity=0.916667 tpcCG=39.732498 0 -GCTTTAATCTGTTCTG

Rev.of_arqueas_representative:NZ_CP009528.1_Methanosarcina_barkeri_MS:82:129109-129228 Satlength=120 Nr of Repeats=7 RepeatLength=17 seed=AGAAAACAGA Num.seqs=7 Similarity=0.955182 tpcCG=39.168999 1 TGCTTTACTCTGTTTTC

arqueas_representative:NZ_CP009520.1_Methanosarcina_vacuolata_Z-761:88:4341361-4341548 Satlength=188 Nr of Repeats=11 RepeatLength=17 seed=TTTAATCCGT Num.seqs=11 Similarity=0.985740 tpcCG=39.732498 3 TACTTTAATCCGTTTTC

***** ** *** *

Consensus:

tgCTTTAaTCtGTTtTc

>arqueas_representative_Fam_145_13_3 Nr. of seq. 3 Alignment length(with gaps) = 16 Alignment score = 0.645833

arqueas_representative:NZ_CP009528.1_Methanosarcina_barkeri_MS:82:1077569-1077647 Satlength=79 Nr of Repeats=6 RepeatLength=13 seed=TCTAAGAAAA Num.seqs=4 Similarity=0.948718 tpcCG=39.168999 10 AAGAAAA-AGA--TCT

Rev.of_arqueas_representative:NZ_CP009506.1_Methanosarcina_siciliae_T4/M:86:4264773-4264857 Satlength=85 Nr of Repeats=6 RepeatLength=14 seed=TCTTTTTCTT Num.seqs=6 Similarity=0.860317 tpcCG=42.900002 10 AAGAAAA-AGA-ATCG

arqueas_representative:NZ_CP009515.1_Methanosarcina_lacustris_Z-7289:84:2299621-2299681 Satlength=61 Nr of Repeats=4 RepeatLength=15 seed=AAATAGAAGC Num.seqs=4 Similarity=1.000000 tpcCG=41.799999 17 AAGAAAATAGAA-GCG

* ******* ***

Consensus:

tCgAAGAAAAAGA

>arqueas_representative_Fam_146_15_3 Nr. of seq. 3 Alignment length(with gaps) = 16 Alignment score = 0.604167

arqueas_representative:NC_014253.1_Methanohalobium_evestigatum_Z-7303:70:1208581-1208762 Satlength=182 Nr of Repeats=13 RepeatLength=14 seed=TAAATCCTTT Num.seqs=12 Similarity=0.923521 tpcCG=36.395599 0 TAA-ATCCTTTATCT-

Rev.of_arqueas_representative:NZ_CP009520.1_Methanosarcina_vacuolata_Z-761:88:2654548-2654692 Satlength=145 Nr of Repeats=8 RepeatLength=16 seed=TGTTAGAGAG Num.seqs=7 Similarity=0.888889 tpcCG=39.7324 5 TAACATCCTTTCTCTC

Rev.of_arqueas_representative:NZ_CP009512.1_Methanosarcina_mazei_S-6:85:3705887-3705971 Satlength=85 Nr of Repeats=6 RepeatLength=14 seed=GGAAGGATTT Num.seqs=6 Similarity=0.822222 tpcCG=41.400002 11 -AAAATCCTTCCTCG-

** ****** **

Consensus:

tAAnATCCTTtcTCt

>arqueas_representative_Fam_147_14_3 Nr. of seq. 3 Alignment length(with gaps) = 16 Alignment score = 0.625000

arqueas_representative:NC_003552.1_Methanosarcina_acetivorans_str._C2A:80:1531473-1531582 Satlength=110 Nr of Repeats=8 RepeatLength=14 seed=ATCTGGTAAT Num.seqs=5 Similarity=1.000000 tpcCG=42.700001 0 ATCTGG--TAATTGTT

arqueas_representative:NZ_CP009517.1_Methanosarcina_barkeri_3:81:947076-947201 Satlength=126 Nr of Repeats=9 RepeatLength=14 seed=AAACGTAATC Num.seqs=8 Similarity=0.904762 tpcCG=39.099998 7 ATCTGG--TAAACGTA

arqueas_representative:NZ_CP009528.1_Methanosarcina_barkeri_MS:82:3884787-3884867 Satlength=81 Nr of Repeats=5 RepeatLength=16 seed=GTTTAATGGT Num.seqs=5 Similarity=1.000000 tpcCG=39.168999 5 ATCTGGTTTAATGGTT

****** *** **

Consensus:

ATCTGGTAAtnGTt

>arqueas_representative_Fam_148_15_3 Nr. of seq. 3 Alignment length(with gaps) = 16 Alignment score = 0.618056

arqueas_representative:NZ_CP009517.1_Methanosarcina_barkeri_3:81:983900-984012 Satlength=113 Nr of Repeats=8 RepeatLength=14 seed=CTGACATACA Num.seqs=8 Similarity=1.000000 tpcCG=39.099998 8 TACAA-G-ATCTGACA

Rev.of_arqueas_representative:NZ_CP009506.1_Methanosarcina_siciliae_T4/M:86:3274035-3274110 Satlength=76 Nr of Repeats=5 RepeatLength=15 seed=TTTATGTCAG Num.seqs=5 Similarity=0.964444 tpcCG=42.900002 18 TAAAAGG-AGCTGACA

Rev.of_arqueas_representative:NZ_CP009520.1_Methanosarcina_vacuolata_Z-761:88:1212095-1212159 Satlength=65 Nr of Repeats=4 RepeatLength=16 seed=TCTCAGTTAC Num.seqs=4 Similarity=0.944444 tpcCG=39.73249 14 TACAAGGTAACTGAGA

**** *** ** * *

Consensus:

CTGAcATAcAAgGAn

>arqueas_representative_Fam_149_14_3 Nr. of seq. 3 Alignment length(with gaps) = 16 Alignment score = 0.625000

arqueas_representative:NZ_CP009520.1_Methanosarcina_vacuolata_Z-761:88:119596-119794 Satlength=199 Nr of Repeats=4 RepeatLength=14 seed=AGTGTTAATT Num.seqs=3 Similarity=1.000000 tpcCG=39.732498 0 AGTG-TTAATTT-GAT

Rev.of_arqueas_representative:NZ_CP009520.1_Methanosarcina_vacuolata_Z-761:88:3478668-3478780 Satlength=113 Nr of Repeats=8 RepeatLength=14 seed=GAACACTTTC Num.seqs=8 Similarity=1.000000 tpcCG=39.7324 7 AGTG-TTCATTT-GAA

Rev.of_arqueas_representative:NZ_CP009512.1_Methanosarcina_mazei_S-6:85:2928462-2928606 Satlength=145 Nr of Repeats=8 RepeatLength=16 seed=AAAGTAAGCA Num.seqs=7 Similarity=1.000000 tpcCG=41.400002 12 AGTGCTTACTTTAGAT

**** ** *** **

Consensus:

AGTGTTaaTTTGAt

>arqueas_representative_Fam_150_14_3 Nr. of seq. 3 Alignment length(with gaps) = 16 Alignment score = 0.659722

arqueas_representative:NZ_CP009520.1_Methanosarcina_vacuolata_Z-761:88:690488-690612 Satlength=125 Nr of Repeats=7 RepeatLength=14 seed=AAATTACTTT Num.seqs=5 Similarity=0.942857 tpcCG=39.732498 0 AAATTACT-TTCTGT-

Rev.of_arqueas_representative:NZ_CP009515.1_Methanosarcina_lacustris_Z-7289:84:124286-124363 Satlength=78 Nr of Repeats=4 RepeatLength=15 seed=GAAGAGTAAT Num.seqs=3 Similarity=0.881481 tpcCG=41.799999 12 AAATTACTCTTCTAT-

Rev.of_arqueas_representative:NZ_CP009512.1_Methanosarcina_mazei_S-6:85:1453973-1454088 Satlength=116 Nr of Repeats=5 RepeatLength=15 seed=TCTTACAGAA Num.seqs=3 Similarity=1.000000 tpcCG=41.400002 3 AGATTATT-TTCTGTA

* **** * **** *

Consensus:

AaATTAcTTTCTgT

>arqueas_representative_Fam_151_15_3 Nr. of seq. 3 Alignment length(with gaps) = 16 Alignment score = 0.618056

arqueas_representative:NZ_CP009520.1_Methanosarcina_vacuolata_Z-761:88:1847759-1847829 Satlength=71 Nr of Repeats=5 RepeatLength=14 seed=CAAGGACTAA Num.seqs=5 Similarity=1.000000 tpcCG=39.732498 0 CAAGGACTAA-GAG-C

Rev.of_arqueas_representative:NZ_CP009520.1_Methanosarcina_vacuolata_Z-761:88:110929-110989 Satlength=61 Nr of Repeats=4 RepeatLength=15 seed=GTCTCTTACT Num.seqs=4 Similarity=0.955556 tpcCG=39.732498 0 CAAAGAGTAA-GAGAC

Rev.of_arqueas_representative:NZ_CP009520.1_Methanosarcina_vacuolata_Z-761:88:3279619-3279782 Satlength=164 Nr of Repeats=9 RepeatLength=16 seed=ATTTTTCTTT Num.seqs=6 Similarity=0.911111 tpcCG=39.7324 11 CAAAGAAAAATGAGAC

*** ** ** *** *

Consensus:

CAAaGAntAAGAGaC

>arqueas_representative_Fam_152_15_3 Nr. of seq. 3 Alignment length(with gaps) = 16 Alignment score = 0.673611

arqueas_representative:NZ_CP014265.1_Methanobrevibacter_olleyae__YLM1:49:156508-156586 Satlength=79 Nr of Repeats=5 RepeatLength=15 seed=TTATTTTTAT Num.seqs=4 Similarity=0.800347 tpcCG=26.900000 0 TT-ATTTTTATTACTT

arqueas_representative:NZ_CP009512.1_Methanosarcina_mazei_S-6:85:2093830-2094312 Satlength=483 Nr of Repeats=8 RepeatLength=15 seed=TTCTATTTTT Num.seqs=7 Similarity=0.923810 tpcCG=41.400002 13 CT-ATTTTTCTTACTT

Rev.of_arqueas_representative:NZ_CP009517.1_Methanosarcina_barkeri_3:81:3361940-3362059 Satlength=120 Nr of Repeats=8 RepeatLength=15 seed=AAGTTAAAAA Num.seqs=5 Similarity=0.964444 tpcCG=39.099998 0 CTGATTTTT-TAACTT

* ****** * ****

Consensus:

cTATTTTTnTtACTT

>arqueas_representative_Fam_153_15_3 Nr. of seq. 3 Alignment length(with gaps) = 16 Alignment score = 0.638889

arqueas_representative:NC_003552.1_Methanosarcina_acetivorans_str._C2A:80:248856-248916 Satlength=61 Nr of Repeats=4 RepeatLength=15 seed=ATTCATAGTT Num.seqs=4 Similarity=0.911111 tpcCG=42.700001 0 ATTCATAGTTTG-TAG

arqueas_representative:NC_003552.1_Methanosarcina_acetivorans_str._C2A:80:2435969-2436104 Satlength=136 Nr of Repeats=9 RepeatLength=15 seed=ATTCATAGAT Num.seqs=9 Similarity=0.916049 tpcCG=42.700001 0 ATTCATAGATTGAAA-

arqueas_representative:NZ_CP009528.1_Methanosarcina_barkeri_MS:82:939055-939252 Satlength=198 Nr of Repeats=11 RepeatLength=15 seed=AAAGATTGAT Num.seqs=9 Similarity=0.851852 tpcCG=39.168999 4 ATTNAAAGATTGATA-

*** * ** *** *

Consensus:

ATTcAtAGaTTGatA

>arqueas_representative_Fam_154_15_3 Nr. of seq. 3 Alignment length(with gaps) = 16 Alignment score = 0.618056

arqueas_representative:NZ_CP009517.1_Methanosarcina_barkeri_3:81:405238-405326 Satlength=89 Nr of Repeats=6 RepeatLength=15 seed=TTTTATTATA Num.seqs=4 Similarity=0.881481 tpcCG=39.099998 0 TT-TTATTATACTCTA

arqueas_representative:NZ_CP009517.1_Methanosarcina_barkeri_3:81:849425-849550 Satlength=126 Nr of Repeats=8 RepeatLength=15 seed=TTATTTTATA Num.seqs=7 Similarity=1.000000 tpcCG=39.099998 2 TT-TTATTTTATACTA

Rev.of_arqueas_representative:NZ_CP009517.1_Methanosarcina_barkeri_3:81:3861231-3861567 Satlength=337 Nr of Repeats=8 RepeatLength=16 seed=AAGTATAAAA Num.seqs=7 Similarity=1.000000 tpcCG=39.099998 15 TTCTTCTTTTATACTT

** ** ** ** **

Consensus:

TTTTaTTtTAtaCTa

>arqueas_representative_Fam_155_15_3 Nr. of seq. 3 Alignment length(with gaps) = 16 Alignment score = 0.673611

arqueas_representative:NZ_CP009517.1_Methanosarcina_barkeri_3:81:3745734-3745793 Satlength=60 Nr of Repeats=4 RepeatLength=15 seed=ACCAATGTTG Num.seqs=3 Similarity=1.000000 tpcCG=39.099998 0 ACCAATGTTGAA-AAA

arqueas_representative:NZ_CP009517.1_Methanosarcina_barkeri_3:81:3745826-3745931 Satlength=106 Nr of Repeats=7 RepeatLength=15 seed=TATTGAAAAG Num.seqs=7 Similarity=1.000000 tpcCG=39.099998 5 ACCAGTATTGAA-AAG

Rev.of_arqueas_representative:NZ_CP009512.1_Methanosarcina_mazei_S-6:85:722244-722308 Satlength=65 Nr of Repeats=4 RepeatLength=16 seed=TTTTATTCAT Num.seqs=4 Similarity=1.000000 tpcCG=41.400002 1 ACCAGTAATGAATAAA

**** * **** **

Consensus:

ACCAgTatTGAAAAa

>arqueas_representative_Fam_156_16_3 Nr. of seq. 3 Alignment length(with gaps) = 16 Alignment score = 0.659722

arqueas_representative:NZ_CP009528.1_Methanosarcina_barkeri_MS:82:202733-202866 Satlength=134 Nr of Repeats=9 RepeatLength=15 seed=TTTTAAAACA Num.seqs=7 Similarity=0.744048 tpcCG=39.168999 0 TTTTAAAACAANTCT-

arqueas_representative:NZ_CP009520.1_Methanosarcina_vacuolata_Z-761:88:693693-693819 Satlength=127 Nr of Repeats=8 RepeatLength=16 seed=CCTTTTTTAA Num.seqs=7 Similarity=0.928571 tpcCG=39.732498 12 TTTTAAAATAATCCTT

Rev.of_arqueas_representative:NZ_CP009517.1_Methanosarcina_barkeri_3:81:453730-453810 Satlength=81 Nr of Repeats=5 RepeatLength=16 seed=TTTTTGAAAA Num.seqs=5 Similarity=0.966667 tpcCG=39.099998 8 TTCAAAAACAAGCCTT

** **** ** **

Consensus:

TTttAAAAcAAncCTt

>arqueas_representative_Fam_157_15_3 Nr. of seq. 3 Alignment length(with gaps) = 16 Alignment score = 0.645833

arqueas_representative:NZ_CP009515.1_Methanosarcina_lacustris_Z-7289:84:3004198-3004404 Satlength=207 Nr of Repeats=14 RepeatLength=15 seed=TGTTACAAAC Num.seqs=10 Similarity=1.000000 tpcCG=41.799999 0 TGTTAC-AAACCAGAT

arqueas_representative:NZ_CP009520.1_Methanosarcina_vacuolata_Z-761:88:3188265-3188340 Satlength=76 Nr of Repeats=5 RepeatLength=15 seed=TAACCAGATT Num.seqs=5 Similarity=0.928889 tpcCG=39.732498 6 TATTTC-TAACCAGAT

arqueas_representative:NZ_CP009528.1_Methanosarcina_barkeri_MS:82:2389397-2389493 Satlength=97 Nr of Repeats=6 RepeatLength=16 seed=ATTATTGCTT Num.seqs=6 Similarity=0.972222 tpcCG=39.168999 13 TATTGCTTAAACAGAT

* ** * ** *****

Consensus:

TaTTnCtAAcCAGAT

>arqueas_representative_Fam_158_16_3 Nr. of seq. 3 Alignment length(with gaps) = 16 Alignment score = 0.729167

arqueas_representative:NZ_CP009515.1_Methanosarcina_lacustris_Z-7289:84:4066190-4066267 Satlength=78 Nr of Repeats=5 RepeatLength=15 seed=TTTATTTCAG Num.seqs=3 Similarity=1.000000 tpcCG=41.799999 3 TTA-TTTATTTCAGGT

Rev.of_arqueas_representative:NZ_CP009520.1_Methanosarcina_vacuolata_Z-761:88:477707-477771 Satlength=65 Nr of Repeats=4 RepeatLength=16 seed=AAACCTGAAA Num.seqs=4 Similarity=0.958333 tpcCG=39.732498 17 TTCTTTTCTTTCAGGT

Rev.of_arqueas_representative:NZ_CP009520.1_Methanosarcina_vacuolata_Z-761:88:1399351-1399416 Satlength=66 Nr of Repeats=4 RepeatLength=16 seed=AAAAGAAAAC Num.seqs=3 Similarity=1.000000 tpcCG=39.73249 6 TTCTTTTACTTCAGTT

*** ***** ***

Consensus:

TTTatTTCAGgTTTct

>arqueas_representative_Fam_159_15_3 Nr. of seq. 3 Alignment length(with gaps) = 16 Alignment score = 0.673611

arqueas_representative:NZ_CP009520.1_Methanosarcina_vacuolata_Z-761:88:672815-672875 Satlength=61 Nr of Repeats=4 RepeatLength=15 seed=ATGTTGAACT Num.seqs=4 Similarity=1.000000 tpcCG=39.732498 0 ATGTTG-AACTAAATT

Rev.of_arqueas_representative:NC_003552.1_Methanosarcina_acetivorans_str._C2A:80:2413996-2414076 Satlength=81 Nr of Repeats=5 RepeatLength=16 seed=CATAATTTAG Num.seqs=5 Similarity=0.900000 tpcCG=42.70 3 ATGTGGAAACTAAATT

arqueas_representative:NZ_CP009520.1_Methanosarcina_vacuolata_Z-761:88:1821847-1821912 Satlength=66 Nr of Repeats=4 RepeatLength=15 seed=TTAATTATGT Num.seqs=3 Similarity=1.000000 tpcCG=39.732498 9 ATGTTC-AGCTTAATT

**** * ** ****

Consensus:

ATGTtgAaCTaAATT

>arqueas_representative_Fam_160_16_3 Nr. of seq. 3 Alignment length(with gaps) = 16 Alignment score = 0.638889

arqueas_representative:NC_015216.1_Methanobacterium_lacus__AL-21:46:1870016-1870200 Satlength=185 Nr of Repeats=11 RepeatLength=16 seed=CTTAACAACT Num.seqs=10 Similarity=1.000000 tpcCG=35.799999 0 CTTAACAACTTAACAA

Rev.of_arqueas_representative:NZ_CP009517.1_Methanosarcina_barkeri_3:81:1040385-1040465 Satlength=81 Nr of Repeats=5 RepeatLength=16 seed=GTTAAGTTAT Num.seqs=5 Similarity=0.950000 tpcCG=39.099998 6 CTTAACTAAGTAATAA

Rev.of_arqueas_representative:NZ_CP009515.1_Methanosarcina_lacustris_Z-7289:84:2258362-2258455 Satlength=94 Nr of Repeats=5 RepeatLength=16 seed=ATTTAAGTTA Num.seqs=4 Similarity=0.861111 tpcCG=41.7999 7 CTTAAATGGGTAATAA

***** *** **

Consensus:

CTTAActangTAAtAA

>arqueas_representative_Fam_161_16_3 Nr. of seq. 3 Alignment length(with gaps) = 16 Alignment score = 0.666667

arqueas_representative:NZ_CP009517.1_Methanosarcina_barkeri_3:81:4096915-4097043 Satlength=129 Nr of Repeats=7 RepeatLength=16 seed=TTTAGTTAAT Num.seqs=6 Similarity=0.755556 tpcCG=39.099998 0 TTTAGTTAATTCCCAC

Rev.of_arqueas_representative:NZ_CP009520.1_Methanosarcina_vacuolata_Z-761:88:3706129-3706193 Satlength=65 Nr of Repeats=4 RepeatLength=16 seed=AATTCACTAA Num.seqs=4 Similarity=0.875000 tpcCG=39.73249 11 TTTAGTGAATTCCTTT

arqueas_representative:NZ_CP009528.1_Methanosarcina_barkeri_MS:82:4077765-4077893 Satlength=129 Nr of Repeats=8 RepeatLength=16 seed=TTCCAGTTTT Num.seqs=8 Similarity=0.955357 tpcCG=39.168999 9 TTTAGTTGATTCCAGT

****** *****

Consensus:

TTTAGTtaATTCCnnt

>arqueas_representative_Fam_162_12_3 Nr. of seq. 3 Alignment length(with gaps) = 15 Alignment score = 0.614815

arqueas_representative:NZ_CP009528.1_Methanosarcina_barkeri_MS:82:197274-197439 Satlength=166 Nr of Repeats=15 RepeatLength=11 seed=TTTCTTCATT Num.seqs=15 Similarity=1.000000 tpcCG=39.168999 0 TTTCTTCAT---TG-

Rev.of_arqueas_representative:NZ_LT719092.1_Cuniculiplasma_divulgatum__PM4_(=JCM_30641;=VKM_B-2940)_:10:1390175-1390223 Satlength=49 Nr of Repeats=4 RepeatLength=12 seed=GAAGAAACCG Num.seqs=4 Similari 7 TTTCTTCAT---CGG

Rev.of_arqueas_representative:NZ_CP009528.1_Methanosarcina_barkeri_MS:82:3386625-3386745 Satlength=121 Nr of Repeats=8 RepeatLength=15 seed=GAAGAAACCA Num.seqs=8 Similarity=1.000000 tpcCG=39.168999 7 TTTCTTCATGAGTGG

********* *

Consensus:

TTTCTTCATtGg

>arqueas_representative_Fam_163_15_3 Nr. of seq. 3 Alignment length(with gaps) = 15 Alignment score = 0.762963

arqueas_representative:NZ_CP019285.1_Halobiforma_lacisalsi_AJ5:23:1145781-1145853 Satlength=73 Nr of Repeats=6 RepeatLength=12 seed=CCGCCGCTCG Num.seqs=6 Similarity=1.000000 tpcCG=65.237297 0 CCGCCGC-TCGT--T

Rev.of_arqueas_representative:NC_019974.1_Natronococcus_occultus_SP4:103:3812396-3812516 Satlength=121 Nr of Repeats=8 RepeatLength=15 seed=GCGGATGACG Num.seqs=8 Similarity=0.930159 tpcCG=64.629402 4 CCGCCGCATCGTCAT

Rev.of_arqueas_representative:NC_019974.1_Natronococcus_occultus_SP4:103:3812399-3812591 Satlength=193 Nr of Repeats=13 RepeatLength=15 seed=GATGACGATG Num.seqs=11 Similarity=0.726203 tpcCG=64.629402 16 CCGCCGCATCGTCAT

******* **** *

Consensus:

CCGCCGCaTCGTcaT

>arqueas_representative_Fam_164_13_3 Nr. of seq. 3 Alignment length(with gaps) = 15 Alignment score = 0.800000

arqueas_representative:NZ_CP009512.1_Methanosarcina_mazei_S-6:85:3605229-3605301 Satlength=73 Nr of Repeats=5 RepeatLength=12 seed=TTTTGGAATC Num.seqs=4 Similarity=0.851852 tpcCG=41.400002 7 GAATCCG---TTTTA

arqueas_representative:NZ_CP009515.1_Methanosarcina_lacustris_Z-7289:84:3120633-3120685 Satlength=53 Nr of Repeats=4 RepeatLength=13 seed=GTTTTTAGAA Num.seqs=4 Similarity=0.897436 tpcCG=41.799999 18 GAATCCGT--TTTTA

arqueas_representative:NZ_CP009506.1_Methanosarcina_siciliae_T4/M:86:1373613-1373725 Satlength=113 Nr of Repeats=6 RepeatLength=15 seed=ATCCGTTTTT Num.seqs=5 Similarity=1.000000 tpcCG=42.900002 14 GAATCCGTTTTTTTA

************

Consensus:

TTTTAGAATCCGt

>arqueas_representative_Fam_165_12_3 Nr. of seq. 3 Alignment length(with gaps) = 15 Alignment score = 0.622222

arqueas_representative:NZ_CP009520.1_Methanosarcina_vacuolata_Z-761:88:1356871-1356973 Satlength=103 Nr of Repeats=8 RepeatLength=12 seed=TTTTTCTTTC Num.seqs=5 Similarity=0.844444 tpcCG=39.732498 0 -TTTTTCTTTC-CC-

arqueas_representative:NZ_CP009520.1_Methanosarcina_vacuolata_Z-761:88:858407-858459 Satlength=53 Nr of Repeats=4 RepeatLength=13 seed=TTTTTCTTTC Num.seqs=4 Similarity=0.880342 tpcCG=39.732498 0 -TTTTTCTTTC-CGT

Rev.of_arqueas_representative:NZ_CP009515.1_Methanosarcina_lacustris_Z-7289:84:1019648-1019746 Satlength=99 Nr of Repeats=7 RepeatLength=14 seed=GGAGACAGAA Num.seqs=7 Similarity=0.918367 tpcCG=41.7999 0 CTTTTTCTGTCTCC-

******* ** *

Consensus:

TTTTTCTtTCCc

>arqueas_representative_Fam_166_13_3 Nr. of seq. 3 Alignment length(with gaps) = 15 Alignment score = 0.644444

arqueas_representative:NC_013790.1_Methanobrevibacter_ruminantium_M1:50:993090-993153 Satlength=64 Nr of Repeats=4 RepeatLength=13 seed=TTTTATTTAT Num.seqs=3 Similarity=0.863248 tpcCG=32.599998 27 A-TTTTAT-TTATTC

Rev.of_arqueas_representative:NZ_CP009512.1_Methanosarcina_mazei_S-6:85:803341-803397 Satlength=57 Nr of Repeats=4 RepeatLength=14 seed=ATAAATACAA Num.seqs=4 Similarity=0.857143 tpcCG=41.400002 37 AGTTGTAT-TTATTC

arqueas_representative:NZ_CP009517.1_Methanosarcina_barkeri_3:81:3711803-3711881 Satlength=79 Nr of Repeats=6 RepeatLength=13 seed=TATGTTATTC Num.seqs=6 Similarity=0.945299 tpcCG=39.099998 29 A--GTTATGTTATTC

*** *******

Consensus:

tttTATTTATTCA

>arqueas_representative_Fam_167_14_3 Nr. of seq. 3 Alignment length(with gaps) = 15 Alignment score = 0.629630

arqueas_representative:NZ_CP009528.1_Methanosarcina_barkeri_MS:82:1557297-1557349 Satlength=53 Nr of Repeats=4 RepeatLength=13 seed=AGCTTTAAGG Num.seqs=4 Similarity=0.948718 tpcCG=39.168999 0 AGCTT-TAAGGTTC-

arqueas_representative:NZ_CP009520.1_Methanosarcina_vacuolata_Z-761:88:3259612-3259710 Satlength=99 Nr of Repeats=7 RepeatLength=14 seed=AAGAATTTAG Num.seqs=7 Similarity=0.954649 tpcCG=39.732498 6 AGCTT-TAAGAATTT

arqueas_representative:NC_003552.1_Methanosarcina_acetivorans_str._C2A:80:2243000-2243210 Satlength=211 Nr of Repeats=14 RepeatLength=15 seed=ATTTAAGCTT Num.seqs=14 Similarity=1.000000 tpcCG=42.700001 9 AGCTTCTAAGATTTA

***** **** *

Consensus:

AGCTTTAAGatTtn

>arqueas_representative_Fam_168_14_3 Nr. of seq. 3 Alignment length(with gaps) = 15 Alignment score = 0.718518

arqueas_representative:CP009516.1_Methanosarcina_horonobensis_HB-1:83:2413207-2413272 Satlength=66 Nr of Repeats=5 RepeatLength=13 seed=AATAAATCAA Num.seqs=5 Similarity=1.000000 tpcCG=41.299999 0 AATAA-ATCAACAA-

arqueas_representative:NC_014222.1_Methanococcus_voltae_A3:65:1923308-1923364 Satlength=57 Nr of Repeats=4 RepeatLength=14 seed=ATAAATAAAC Num.seqs=4 Similarity=1.000000 tpcCG=28.600000 1 AATAA-ATAAACAAT

arqueas_representative:NZ_CP009520.1_Methanosarcina_vacuolata_Z-761:88:2270284-2270374 Satlength=91 Nr of Repeats=5 RepeatLength=15 seed=AATAATAGCA Num.seqs=4 Similarity=0.955556 tpcCG=39.732498 0 AATAATAGCAACAAT

***** * *****

Consensus:

AATAAAtcAACAAt

>arqueas_representative_Fam_169_13_3 Nr. of seq. 3 Alignment length(with gaps) = 15 Alignment score = 0.659259

arqueas_representative:NZ_CP009515.1_Methanosarcina_lacustris_Z-7289:84:1567472-1567550 Satlength=79 Nr of Repeats=6 RepeatLength=13 seed=GGAATCAGTT Num.seqs=6 Similarity=0.965812 tpcCG=41.799999 0 GGA-ATCAG-TTACT

Rev.of_arqueas_representative:NZ_CP009517.1_Methanosarcina_barkeri_3:81:3294330-3294414 Satlength=85 Nr of Repeats=6 RepeatLength=14 seed=TCCAGTAAGC Num.seqs=6 Similarity=1.000000 tpcCG=39.099998 3 GGA-ATTAGCTTACT

arqueas_representative:NZ_CP009528.1_Methanosarcina_barkeri_MS:82:1674553-1674609 Satlength=57 Nr of Repeats=4 RepeatLength=14 seed=ATCACTTACT Num.seqs=4 Similarity=0.904762 tpcCG=39.168999 4 GGACATCAC-TTACT

*** ** * *****

Consensus:

GGAATcAgTTACT

>arqueas_representative_Fam_170_14_3 Nr. of seq. 3 Alignment length(with gaps) = 15 Alignment score = 0.629630

arqueas_representative:NZ_CP009520.1_Methanosarcina_vacuolata_Z-761:88:1957593-1957645 Satlength=53 Nr of Repeats=4 RepeatLength=13 seed=TTTTCTGGTT Num.seqs=4 Similarity=1.000000 tpcCG=39.732498 0 TTTTCTG-GTTGTA-

arqueas_representative:NZ_CP009528.1_Methanosarcina_barkeri_MS:82:442280-442379 Satlength=100 Nr of Repeats=7 RepeatLength=14 seed=TTTTTTGTTT Num.seqs=6 Similarity=0.968254 tpcCG=39.168999 0 TTTTTTG-TTTGTAT

arqueas_representative:NC_003552.1_Methanosarcina_acetivorans_str._C2A:80:2268263-2268323 Satlength=61 Nr of Repeats=4 RepeatLength=15 seed=TTTCTGTTTT Num.seqs=4 Similarity=1.000000 tpcCG=42.700001 1 TTTTCTGTTTTGCAC

**** ** *** *

Consensus:

TTTTcTGtTTGtAn

>arqueas_representative_Fam_171_14_3 Nr. of seq. 3 Alignment length(with gaps) = 15 Alignment score = 0.711111

arqueas_representative:NZ_CP009520.1_Methanosarcina_vacuolata_Z-761:88:1996099-1996259 Satlength=161 Nr of Repeats=9 RepeatLength=13 seed=TGATTTTTCT Num.seqs=6 Similarity=1.000000 tpcCG=39.732498 0 TGA--TTTTTCTTCC

Rev.of_arqueas_representative:NZ_CP009517.1_Methanosarcina_barkeri_3:81:857111-857167 Satlength=57 Nr of Repeats=4 RepeatLength=14 seed=AAGAACAAAA Num.seqs=4 Similarity=0.952381 tpcCG=39.099998 1 TGA-ATTTTTGTTCT

arqueas_representative:NZ_CP009501.1_Methanosarcina_thermophila_TM-1:87:1291330-1291433 Satlength=104 Nr of Repeats=5 RepeatLength=15 seed=TTCCTGATAT Num.seqs=3 Similarity=1.000000 tpcCG=41.099998 11 TGATATTTTTGTTCC

*** ***** ***

Consensus:

TGAaTTTTTgTTCc

>arqueas_representative_Fam_172_14_3 Nr. of seq. 3 Alignment length(with gaps) = 15 Alignment score = 0.666667

arqueas_representative:NZ_CP009520.1_Methanosarcina_vacuolata_Z-761:88:2727818-2727883 Satlength=66 Nr of Repeats=5 RepeatLength=13 seed=TGATCTCTTA Num.seqs=5 Similarity=1.000000 tpcCG=39.732498 0 TGATCTCTTAAAT--

Rev.of_arqueas_representative:NZ_CP009517.1_Methanosarcina_barkeri_3:81:4554102-4554158 Satlength=57 Nr of Repeats=4 RepeatLength=14 seed=TTTAAGAGTC Num.seqs=4 Similarity=1.000000 tpcCG=39.099998 12 TGGACTCTTAAATT-

arqueas_representative:NZ_CP009517.1_Methanosarcina_barkeri_3:81:664510-664585 Satlength=76 Nr of Repeats=5 RepeatLength=15 seed=TGGACACTTA Num.seqs=5 Similarity=0.964444 tpcCG=39.099998 0 TGGACACTTAAATAC

** * *******

Consensus:

TGgaCtCTTAAATn

>arqueas_representative_Fam_173_15_3 Nr. of seq. 3 Alignment length(with gaps) = 15 Alignment score = 0.651852

arqueas_representative:NZ_CP009517.1_Methanosarcina_barkeri_3:81:85152-85222 Satlength=71 Nr of Repeats=5 RepeatLength=14 seed=TCAAGCTTTT Num.seqs=5 Similarity=1.000000 tpcCG=39.099998 1 ATCAAGCTTTTATC-

arqueas_representative:NZ_CP009520.1_Methanosarcina_vacuolata_Z-761:88:4324053-4324122 Satlength=70 Nr of Repeats=5 RepeatLength=14 seed=TTTCATTCAG Num.seqs=4 Similarity=1.000000 tpcCG=39.732498 8 TC-AGCTATTTTCAT

Rev.of_arqueas_representative:NC_003552.1_Methanosarcina_acetivorans_str._C2A:80:749674-749734 Satlength=61 Nr of Repeats=4 RepeatLength=15 seed=TTGAATGAGA Num.seqs=4 Similarity=1.000000 tpcCG=42.7000 18 TCAAGTTATTCTCAT

* **

Consensus:

tcnAgntaTTttcat

>arqueas_representative_Fam_174_15_3 Nr. of seq. 3 Alignment length(with gaps) = 15 Alignment score = 0.711111

arqueas_representative:NZ_CP009517.1_Methanosarcina_barkeri_3:81:520176-520260 Satlength=85 Nr of Repeats=6 RepeatLength=14 seed=TTTTCGACTT Num.seqs=6 Similarity=0.856296 tpcCG=39.099998 0 TTTTCGACTTTTCT-

arqueas_representative:NC_003552.1_Methanosarcina_acetivorans_str._C2A:80:4586413-4586518 Satlength=106 Nr of Repeats=7 RepeatLength=15 seed=TTTTTTCCAC Num.seqs=7 Similarity=1.000000 tpcCG=42.700001 13 TTTTCCACTTTTCTT

arqueas_representative:NZ_CP009515.1_Methanosarcina_lacustris_Z-7289:84:2911889-2911994 Satlength=106 Nr of Repeats=7 RepeatLength=15 seed=TCTTCTTTCG Num.seqs=7 Similarity=0.923810 tpcCG=41.799999 11 CTTTCGGCATTTCTT

**** * *****

Consensus:

tTTTCgaCtTTTCTt

>arqueas_representative_Fam_175_14_3 Nr. of seq. 3 Alignment length(with gaps) = 15 Alignment score = 0.711111

arqueas_representative:NZ_CP009528.1_Methanosarcina_barkeri_MS:82:1403956-1404068 Satlength=113 Nr of Repeats=8 RepeatLength=14 seed=TTAGGAAGAA Num.seqs=8 Similarity=0.935374 tpcCG=39.168999 0 TTAGGAAGAAT-AGG

arqueas_representative:NZ_CP009515.1_Methanosarcina_lacustris_Z-7289:84:2583889-2583959 Satlength=71 Nr of Repeats=5 RepeatLength=14 seed=CAGGAATAGG Num.seqs=5 Similarity=1.000000 tpcCG=41.799999 4 TTAGCAGGAAT-AGG

Rev.of_arqueas_representative:NZ_CP009515.1_Methanosarcina_lacustris_Z-7289:84:2082951-2083026 Satlength=76 Nr of Repeats=5 RepeatLength=15 seed=AACTTCATTC Num.seqs=5 Similarity=0.964444 tpcCG=41.7999 2 TTAGCAAGAATGAAG

**** * **** * *

Consensus:

TTAGcAaGAATAgG

>arqueas_representative_Fam_176_15_3 Nr. of seq. 3 Alignment length(with gaps) = 15 Alignment score = 0.829630

arqueas_representative:NZ_CP009528.1_Methanosarcina_barkeri_MS:82:1633148-1633204 Satlength=57 Nr of Repeats=4 RepeatLength=14 seed=TTGAGAGTTA Num.seqs=4 Similarity=0.952381 tpcCG=39.168999 5 ATTTC-TTGAGAGTT

arqueas_representative:NZ_CP009528.1_Methanosarcina_barkeri_MS:82:1066919-1066994 Satlength=76 Nr of Repeats=5 RepeatLength=15 seed=TTTGAGAATT Num.seqs=5 Similarity=0.946667 tpcCG=39.168999 5 ATCTCTTTGAGAATT

Rev.of_arqueas_representative:NZ_CP009520.1_Methanosarcina_vacuolata_Z-761:88:961459-961564 Satlength=106 Nr of Repeats=7 RepeatLength=15 seed=AAAGAGATAA Num.seqs=5 Similarity=1.000000 tpcCG=39.732498 8 ATCTCTTTGAGAATT

****** **** **

Consensus:

tTTGAGAaTTATcTC

>arqueas_representative_Fam_177_15_3 Nr. of seq. 3 Alignment length(with gaps) = 15 Alignment score = 0.792593

arqueas_representative:NZ_CP009528.1_Methanosarcina_barkeri_MS:82:4396123-4396431 Satlength=309 Nr of Repeats=13 RepeatLength=14 seed=GAATTTGATA Num.seqs=10 Similarity=1.000000 tpcCG=39.168999 0 GAA-TTTGATAGTGT

Rev.of_arqueas_representative:NC_003552.1_Methanosarcina_acetivorans_str._C2A:80:4162354-4162429 Satlength=76 Nr of Repeats=4 RepeatLength=15 seed=TTCACACAAT Num.seqs=3 Similarity=0.940741 tpcCG=42.70 3 GAAGTTTGATTGTGT

arqueas_representative:NZ_CP009506.1_Methanosarcina_siciliae_T4/M:86:2818826-2819021 Satlength=196 Nr of Repeats=12 RepeatLength=15 seed=GGTTTGATTG Num.seqs=11 Similarity=0.970909 tpcCG=42.900002 17 GAGGTTTGATTGTGT

** ****** ****

Consensus:

GAagTTTGATtGTGT

>arqueas_representative_Fam_178_15_3 Nr. of seq. 3 Alignment length(with gaps) = 15 Alignment score = 0.703704

arqueas_representative:NZ_CP009506.1_Methanosarcina_siciliae_T4/M:86:123420-123505 Satlength=86 Nr of Repeats=6 RepeatLength=14 seed=TGAAAAGAAT Num.seqs=5 Similarity=0.961905 tpcCG=42.900002 0 TGAAAAGAA-TTAGA

Rev.of_arqueas_representative:NC_003552.1_Methanosarcina_acetivorans_str._C2A:80:4208479-4208539 Satlength=61 Nr of Repeats=4 RepeatLength=15 seed=TTTTTCAGCT Num.seqs=4 Similarity=1.000000 tpcCG=42.70 7 TGAAAAAAAATTAGC

arqueas_representative:NZ_CP009528.1_Methanosarcina_barkeri_MS:82:3024289-3024364 Satlength=76 Nr of Repeats=5 RepeatLength=15 seed=AACTGAGATG Num.seqs=5 Similarity=0.946667 tpcCG=39.168999 7 TGAAAAAAACTGAGA

****** ** * **

Consensus:

TGAAAAaAAnTtAGa

>arqueas_representative_Fam_179_15_3 Nr. of seq. 3 Alignment length(with gaps) = 15 Alignment score = 0.674074

arqueas_representative:NC_009634.1_Methanococcus_vannielii_SB:64:791238-791358 Satlength=121 Nr of Repeats=8 RepeatLength=15 seed=CCTAAAGTAG Num.seqs=8 Similarity=1.000000 tpcCG=31.299999 0 CCTAAAGTAGAAGAA

arqueas_representative:NC_018876.1_Methanolobus_psychrophilus_R15:74:276091-276151 Satlength=61 Nr of Repeats=4 RepeatLength=15 seed=CCAGTTGAAG Num.seqs=4 Similarity=0.772569 tpcCG=44.599998 3 CCTCCAGTTGAAGAA

Rev.of_arqueas_representative:NZ_CP009520.1_Methanosarcina_vacuolata_Z-761:88:890511-890616 Satlength=106 Nr of Repeats=5 RepeatLength=15 seed=TCCGATTTTA Num.seqs=3 Similarity=0.881481 tpcCG=39.732498 12 CCTAAAATCGGAGAA

*** * * * ****

Consensus:

CCTaaAgTnGaAGAA

>arqueas_representative_Fam_180_15_3 Nr. of seq. 3 Alignment length(with gaps) = 15 Alignment score = 0.659259

arqueas_representative:NC_003552.1_Methanosarcina_acetivorans_str._C2A:80:918855-919035 Satlength=181 Nr of Repeats=12 RepeatLength=15 seed=GACGAAGAAG Num.seqs=12 Similarity=0.846465 tpcCG=42.700001 0 GACGAAGAAGTAGCT

arqueas_representative:CP009516.1_Methanosarcina_horonobensis_HB-1:83:2481429-2481489 Satlength=61 Nr of Repeats=4 RepeatLength=15 seed=AACTGAAGAA Num.seqs=4 Similarity=0.955556 tpcCG=41.299999 11 GAAGAAGGAGTAACT

Rev.of_arqueas_representative:NZ_CP009520.1_Methanosarcina_vacuolata_Z-761:88:1431954-1432218 Satlength=265 Nr of Repeats=14 RepeatLength=15 seed=CTTCCTCTTC Num.seqs=11 Similarity=0.919192 tpcCG=39.73 13 GAAGAAGAGGAAGNT

** **** * * *

Consensus:

GAaGAAGaaGtAgcT

>arqueas_representative_Fam_181_15_3 Nr. of seq. 3 Alignment length(with gaps) = 15 Alignment score = 0.733333

arqueas_representative:NC_003552.1_Methanosarcina_acetivorans_str._C2A:80:2067408-2067483 Satlength=76 Nr of Repeats=4 RepeatLength=15 seed=CCGATGGAAA Num.seqs=3 Similarity=0.940741 tpcCG=42.700001 0 CCGATGGAAACCAGA

Rev.of_arqueas_representative:NZ_CP009515.1_Methanosarcina_lacustris_Z-7289:84:1792972-1793392 Satlength=421 Nr of Repeats=28 RepeatLength=15 seed=CTGTCTGGTT Num.seqs=28 Similarity=0.934392 tpcCG=41.7 3 CAGATGGCAACCAGA

Rev.of_arqueas_representative:NZ_CP009506.1_Methanosarcina_siciliae_T4/M:86:3168755-3168815 Satlength=61 Nr of Repeats=4 RepeatLength=15 seed=TCAGTCTGAT Num.seqs=4 Similarity=1.000000 tpcCG=42.900002 4 CTGACGGAAATCAGA

* ** ** ** ****

Consensus:

CnGAtGGaAAcCAGA

>arqueas_representative_Fam_182_15_3 Nr. of seq. 3 Alignment length(with gaps) = 15 Alignment score = 0.762963

arqueas_representative:NZ_CP009517.1_Methanosarcina_barkeri_3:81:4506416-4506581 Satlength=166 Nr of Repeats=11 RepeatLength=15 seed=AAATAAAGAA Num.seqs=11 Similarity=1.000000 tpcCG=39.099998 10 AAGAATTCAGAAATA

arqueas_representative:NZ_CP009512.1_Methanosarcina_mazei_S-6:85:3862496-3862933 Satlength=438 Nr of Repeats=5 RepeatLength=15 seed=CAATAAAAAA Num.seqs=3 Similarity=1.000000 tpcCG=41.400002 10 AAAAAATCAGCAATA

arqueas_representative:NZ_CP009506.1_Methanosarcina_siciliae_T4/M:86:2263891-2263996 Satlength=106 Nr of Repeats=7 RepeatLength=15 seed=TCAGAAATAA Num.seqs=7 Similarity=0.974603 tpcCG=42.900002 21 ATAAAATCAGAAATA

***** ** ****

Consensus:

aAATAAaaAAaTCAG

>arqueas_representative_Fam_183_11_3 Nr. of seq. 3 Alignment length(with gaps) = 14 Alignment score = 0.626984

arqueas_representative:NC_014507.1_Methanolacinia_petrolearia_DSM_11571:73:1218603-1218657 Satlength=55 Nr of Repeats=5 RepeatLength=11 seed=GTTTACTGAA Num.seqs=4 Similarity=1.000000 tpcCG=47.400002 0 GTTTA-CTGAAT--

arqueas_representative:NZ_CP009515.1_Methanosarcina_lacustris_Z-7289:84:13862-13922 Satlength=61 Nr of Repeats=5 RepeatLength=12 seed=GTTTAGCAGA Num.seqs=5 Similarity=1.000000 tpcCG=41.799999 0 GTTTAGCAGAAT--

arqueas_representative:NZ_CP009515.1_Methanosarcina_lacustris_Z-7289:84:3914493-3914558 Satlength=66 Nr of Repeats=5 RepeatLength=13 seed=GAATAAGTTT Num.seqs=5 Similarity=1.000000 tpcCG=41.799999 7 GTTTA-CGGAATAA

***** * ****

Consensus:

GTTTACnGAAT

>arqueas_representative_Fam_184_13_3 Nr. of seq. 3 Alignment length(with gaps) = 14 Alignment score = 0.730159

arqueas_representative:NZ_CP009517.1_Methanosarcina_barkeri_3:81:1284612-1284660 Satlength=49 Nr of Repeats=4 RepeatLength=12 seed=TAAGGCTGAA Num.seqs=4 Similarity=1.000000 tpcCG=39.099998 0 TAAGGCT-GAAAA-

arqueas_representative:NZ_CP009520.1_Methanosarcina_vacuolata_Z-761:88:348448-348565 Satlength=118 Nr of Repeats=7 RepeatLength=13 seed=TGAAAAGTAA Num.seqs=5 Similarity=0.759524 tpcCG=39.732498 6 TAAGTCT-GAAAAG

Rev.of_arqueas_representative:NZ_CP009520.1_Methanosarcina_vacuolata_Z-761:88:1751857-1751927 Satlength=71 Nr of Repeats=5 RepeatLength=14 seed=AATTTTCCAG Num.seqs=5 Similarity=1.000000 tpcCG=39.73249 1 TAAGTCTGGAAAAT

**** ** *****

Consensus:

TAAGtCTGAAAAn

>arqueas_representative_Fam_185_12_3 Nr. of seq. 3 Alignment length(with gaps) = 14 Alignment score = 0.650794

arqueas_representative:NZ_CP009517.1_Methanosarcina_barkeri_3:81:2922940-2922996 Satlength=57 Nr of Repeats=4 RepeatLength=12 seed=TACTTATTTA Num.seqs=3 Similarity=0.851852 tpcCG=39.099998 0 -TACTTATTTACT-

Rev.of_arqueas_representative:NC_013790.1_Methanobrevibacter_ruminantium_M1:50:2067508-2067601 Satlength=94 Nr of Repeats=5 RepeatLength=13 seed=AATAAACTAA Num.seqs=3 Similarity=0.931624 tpcCG=32.5999 4 -TATTTATTTACTT

Rev.of_arqueas_representative:NZ_CP009512.1_Methanosarcina_mazei_S-6:85:2687471-2687549 Satlength=79 Nr of Repeats=6 RepeatLength=13 seed=AGTAAAGATG Num.seqs=6 Similarity=1.000000 tpcCG=41.400002 0 CTACATCTTTACT-

** * ******

Consensus:

TActTaTTTACT

>arqueas_representative_Fam_186_12_3 Nr. of seq. 3 Alignment length(with gaps) = 14 Alignment score = 0.738095

arqueas_representative:NZ_CP009528.1_Methanosarcina_barkeri_MS:82:2234796-2234892 Satlength=97 Nr of Repeats=8 RepeatLength=12 seed=TTGTACTCTA Num.seqs=8 Similarity=1.000000 tpcCG=39.168999 4 T-ATT-TTGTACTC

Rev.of_arqueas_representative:NZ_CP009517.1_Methanosarcina_barkeri_3:81:1219798-1219941 Satlength=144 Nr of Repeats=11 RepeatLength=13 seed=AGTTCAAAAA Num.seqs=11 Similarity=1.000000 tpcCG=39.099998 11 T-ATTTTTGAACTC

arqueas_representative:NZ_CP009517.1_Methanosarcina_barkeri_3:81:3983523-3983575 Satlength=53 Nr of Repeats=4 RepeatLength=13 seed=TTTTGAACTC Num.seqs=4 Similarity=1.000000 tpcCG=39.099998 15 TGATT-TTGAACTC

*** ***** ***

Consensus:

TTGaACTCTATT

>arqueas_representative_Fam_187_14_3 Nr. of seq. 3 Alignment length(with gaps) = 14 Alignment score = 0.682540

arqueas_representative:NZ_CP014265.1_Methanobrevibacter_olleyae__YLM1:49:82131-82188 Satlength=58 Nr of Repeats=4 RepeatLength=13 seed=TTTATTAAAT Num.seqs=3 Similarity=0.931624 tpcCG=26.900000 7 AAATTATTTTATT-

Rev.of_arqueas_representative:NC_013790.1_Methanobrevibacter_ruminantium_M1:50:2206972-2207087 Satlength=116 Nr of Repeats=5 RepeatLength=14 seed=TAAAAAAATT Num.seqs=3 Similarity=1.000000 tpcCG=32.599 11 AAATTTTTTTATTC

Rev.of_arqueas_representative:NZ_CP009528.1_Methanosarcina_barkeri_MS:82:3368966-3369036 Satlength=71 Nr of Repeats=5 RepeatLength=14 seed=AAACAAATTT Num.seqs=5 Similarity=1.000000 tpcCG=39.168999 10 AAATTTGTTTAGTA

**** * *****

Consensus:

TTTAtTnAAATTtt

>arqueas_representative_Fam_188_14_3 Nr. of seq. 3 Alignment length(with gaps) = 14 Alignment score = 0.714286

arqueas_representative:NC_013790.1_Methanobrevibacter_ruminantium_M1:50:2075478-2075569 Satlength=92 Nr of Repeats=6 RepeatLength=13 seed=AATAAAAAGA Num.seqs=4 Similarity=0.880342 tpcCG=32.599998 6 AGAACTAATAAAA-

Rev.of_arqueas_representative:NZ_CP009528.1_Methanosarcina_barkeri_MS:82:389670-389768 Satlength=99 Nr of Repeats=7 RepeatLength=14 seed=AGTTCAATTT Num.seqs=7 Similarity=0.954649 tpcCG=39.168999 6 TGAACTAATAAAAT

Rev.of_arqueas_representative:NZ_CP009520.1_Methanosarcina_vacuolata_Z-761:88:4464665-4464769 Satlength=105 Nr of Repeats=8 RepeatLength=13 seed=TTTTTAGTTC Num.seqs=8 Similarity=1.000000 tpcCG=39.7324 11 GGAACTAA-AAAAT

** **** *****

Consensus:

AAtAAAAtnGAACT

>arqueas_representative_Fam_189_13_3 Nr. of seq. 3 Alignment length(with gaps) = 14 Alignment score = 0.690476

arqueas_representative:NC_003552.1_Methanosarcina_acetivorans_str._C2A:80:2465409-2465461 Satlength=53 Nr of Repeats=4 RepeatLength=13 seed=ATTTTTACCT Num.seqs=4 Similarity=1.000000 tpcCG=42.700001 0 ATTTTTA-CCTGAT

arqueas_representative:NC_003552.1_Methanosarcina_acetivorans_str._C2A:80:2465469-2465534 Satlength=66 Nr of Repeats=5 RepeatLength=13 seed=CTGATGTTTT Num.seqs=5 Similarity=1.000000 tpcCG=42.700001 8 GTTTTTA-TCTGAT

Rev.of_arqueas_representative:CP009516.1_Methanosarcina_horonobensis_HB-1:83:2093537-2093607 Satlength=71 Nr of Repeats=5 RepeatLength=14 seed=AAAAGTATCA Num.seqs=5 Similarity=1.000000 tpcCG=41.299999 6 ACTTTTATCCTGAT

***** *****

Consensus:

atTTTTAcCTGAT

>arqueas_representative_Fam_190_13_3 Nr. of seq. 3 Alignment length(with gaps) = 14 Alignment score = 0.730159

arqueas_representative:NZ_CP009515.1_Methanosarcina_lacustris_Z-7289:84:1526427-1526492 Satlength=66 Nr of Repeats=4 RepeatLength=13 seed=TATAATTGCA Num.seqs=3 Similarity=0.931624 tpcCG=41.799999 0 -TATAATTGCACAG

Rev.of_arqueas_representative:NZ_CP009517.1_Methanosarcina_barkeri_3:81:1625731-1625829 Satlength=99 Nr of Repeats=7 RepeatLength=14 seed=CTGAGCAATT Num.seqs=7 Similarity=0.918367 tpcCG=39.099998 0 ATATAATTGCTCAG

Rev.of_arqueas_representative:NZ_CP009520.1_Methanosarcina_vacuolata_Z-761:88:699413-699504 Satlength=92 Nr of Repeats=7 RepeatLength=13 seed=GAAATTATTC Num.seqs=7 Similarity=1.000000 tpcCG=39.732498 9 -AATAATTTCACAG

****** * ***

Consensus:

tATAATTgCaCAG

>arqueas_representative_Fam_191_14_3 Nr. of seq. 3 Alignment length(with gaps) = 14 Alignment score = 0.698413

arqueas_representative:NC_009515.1_Methanobrevibacter_smithii_ATCC_35061:51:1246119-1246238 Satlength=120 Nr of Repeats=5 RepeatLength=14 seed=ATAAGTTATA Num.seqs=3 Similarity=0.714286 tpcCG=31.000000 0 ATNAGTTATAAGTT

arqueas_representative:NC_014222.1_Methanococcus_voltae_A3:65:237714-237770 Satlength=57 Nr of Repeats=4 RepeatLength=14 seed=TTATAAGTTA Num.seqs=4 Similarity=0.904762 tpcCG=28.600000 5 ATACTTTATAAGTT

Rev.of_arqueas_representative:NZ_CP009520.1_Methanosarcina_vacuolata_Z-761:88:2472070-2472224 Satlength=155 Nr of Repeats=10 RepeatLength=14 seed=AAGTATAACT Num.seqs=9 Similarity=0.910053 tpcCG=39.732 6 ATACTTTTCAAGTT

** ** *****

Consensus:

ATactTTatAAGTT

>arqueas_representative_Fam_192_14_3 Nr. of seq. 3 Alignment length(with gaps) = 14 Alignment score = 0.650794

arqueas_representative:NC_003552.1_Methanosarcina_acetivorans_str._C2A:80:3455618-3455800 Satlength=183 Nr of Repeats=13 RepeatLength=14 seed=TTTCACCAAT Num.seqs=13 Similarity=1.000000 tpcCG=42.700001 0 TTTCACCAATATCT

arqueas_representative:NZ_CP009517.1_Methanosarcina_barkeri_3:81:1109828-1109926 Satlength=99 Nr of Repeats=7 RepeatLength=14 seed=CTATCTTTTA Num.seqs=7 Similarity=0.954649 tpcCG=39.099998 8 TTTAAGCACTATCT

arqueas_representative:NZ_CP009520.1_Methanosarcina_vacuolata_Z-761:88:273320-273530 Satlength=211 Nr of Repeats=13 RepeatLength=14 seed=TTTTCCCCAT Num.seqs=12 Similarity=0.961039 tpcCG=39.732498 13 TTTCCCCATTCTCT

*** ** * ***

Consensus:

TTTcacCAnTaTCT

>arqueas_representative_Fam_193_14_3 Nr. of seq. 3 Alignment length(with gaps) = 14 Alignment score = 0.746032

arqueas_representative:NC_003552.1_Methanosarcina_acetivorans_str._C2A:80:4646112-4646196 Satlength=85 Nr of Repeats=6 RepeatLength=14 seed=AACTCAATAT Num.seqs=6 Similarity=1.000000 tpcCG=42.700001 0 AACTCAATATTGTA

Rev.of_arqueas_representative:NZ_CP009528.1_Methanosarcina_barkeri_MS:82:3865253-3865444 Satlength=192 Nr of Repeats=5 RepeatLength=14 seed=AGTTTAGAAT Num.seqs=4 Similarity=1.000000 tpcCG=39.168999 4 AACTGGCTATTCTA

arqueas_representative:NZ_CP009520.1_Methanosarcina_vacuolata_Z-761:88:492063-492119 Satlength=57 Nr of Repeats=4 RepeatLength=14 seed=TATTCTAAAC Num.seqs=4 Similarity=0.952381 tpcCG=39.732498 7 AACTGGCTATTCTA

**** **** **

Consensus:

AACTggcTATTcTA

>arqueas_representative_Fam_194_14_3 Nr. of seq. 3 Alignment length(with gaps) = 14 Alignment score = 0.650794

arqueas_representative:NZ_CP009517.1_Methanosarcina_barkeri_3:81:1963009-1963086 Satlength=78 Nr of Repeats=5 RepeatLength=14 seed=AATCAGTAAT Num.seqs=4 Similarity=0.825397 tpcCG=39.099998 0 AATCAGTAATCAAC

Rev.of_arqueas_representative:NZ_CP009520.1_Methanosarcina_vacuolata_Z-761:88:1887282-1887359 Satlength=78 Nr of Repeats=5 RepeatLength=14 seed=TTGATTTTTG Num.seqs=4 Similarity=1.000000 tpcCG=39.73249 6 AATCAAAAATCAAA

arqueas_representative:NZ_CP009520.1_Methanosarcina_vacuolata_Z-761:88:3369030-3369100 Satlength=71 Nr of Repeats=5 RepeatLength=14 seed=ATCTCCAATC Num.seqs=5 Similarity=1.000000 tpcCG=39.732498 1 AATCTCCAATCAAT

**** ******

Consensus:

AATCannAATCAAn

>arqueas_representative_Fam_195_14_3 Nr. of seq. 3 Alignment length(with gaps) = 14 Alignment score = 0.746032

arqueas_representative:NZ_CP009517.1_Methanosarcina_barkeri_3:81:3799799-3799855 Satlength=57 Nr of Repeats=4 RepeatLength=14 seed=TGATCAGTAA Num.seqs=4 Similarity=1.000000 tpcCG=39.099998 0 TGATCAGTAAATTA

arqueas_representative:NZ_CP009515.1_Methanosarcina_lacustris_Z-7289:84:4009350-4009402 Satlength=53 Nr of Repeats=4 RepeatLength=13 seed=ATCAGTAAAT Num.seqs=4 Similarity=0.777778 tpcCG=41.799999 2 AGATCAGTAAATCA

arqueas_representative:NZ_CP009520.1_Methanosarcina_vacuolata_Z-761:88:4414145-4414319 Satlength=175 Nr of Repeats=6 RepeatLength=14 seed=AGTAAATTAA Num.seqs=4 Similarity=1.000000 tpcCG=39.732498 5 AACTCAGTAAATTA

********* *

Consensus:

agaTCAGTAAATtA

>arqueas_representative_Fam_196_14_3 Nr. of seq. 3 Alignment length(with gaps) = 14 Alignment score = 0.809524

arqueas_representative:NZ_CP009517.1_Methanosarcina_barkeri_3:81:4516629-4516699 Satlength=71 Nr of Repeats=5 RepeatLength=14 seed=TCAAAGTAAA Num.seqs=5 Similarity=1.000000 tpcCG=39.099998 0 TCAAAGTAAAGAAG

arqueas_representative:NZ_CP009528.1_Methanosarcina_barkeri_MS:82:4487471-4487611 Satlength=141 Nr of Repeats=10 RepeatLength=14 seed=AGAAGTCAAA Num.seqs=10 Similarity=0.955556 tpcCG=39.168999 9 TCAAAGTAGAGAAG

arqueas_representative:NZ_CP009528.1_Methanosarcina_barkeri_MS:82:3317276-3317332 Satlength=57 Nr of Repeats=4 RepeatLength=14 seed=AACTCAAAGA Num.seqs=4 Similarity=0.952381 tpcCG=39.168999 11 TCAAAGAAGAGAAC

****** * ****

Consensus:

TCAAAGtAgAGAAg

>arqueas_representative_Fam_197_12_3 Nr. of seq. 3 Alignment length(with gaps) = 13 Alignment score = 0.632479

arqueas_representative:NZ_CP009520.1_Methanosarcina_vacuolata_Z-761:88:1747326-1747376 Satlength=51 Nr of Repeats=4 RepeatLength=10 seed=ATTTTTCTAG Num.seqs=3 Similarity=1.000000 tpcCG=39.732498 0 AT-TTTTC--TAG

Rev.of_arqueas_representative:NZ_CP009520.1_Methanosarcina_vacuolata_Z-761:88:92255-92375 Satlength=121 Nr of Repeats=8 RepeatLength=12 seed=TGAAAAATCT Num.seqs=6 Similarity=0.881481 tpcCG=39.732498 8 AT-TTTTCAATAG

arqueas_representative:NZ_CP009520.1_Methanosarcina_vacuolata_Z-761:88:1516735-1516853 Satlength=119 Nr of Repeats=8 RepeatLength=13 seed=AGATATTTTC Num.seqs=7 Similarity=0.857143 tpcCG=39.732498 10 ATATTTTCAAGAG

** ***** **

Consensus:

ATTTTTCaatAG

>arqueas_representative_Fam_198_11_3 Nr. of seq. 3 Alignment length(with gaps) = 13 Alignment score = 0.641026

arqueas_representative:NC_003552.1_Methanosarcina_acetivorans_str._C2A:80:1572544-1572720 Satlength=177 Nr of Repeats=16 RepeatLength=11 seed=AACATTTTTT Num.seqs=16 Similarity=0.930303 tpcCG=42.700001 0 AACATT--TTTTG

Rev.of_arqueas_representative:NZ_CP009520.1_Methanosarcina_vacuolata_Z-761:88:2537339-2537383 Satlength=45 Nr of Repeats=4 RepeatLength=11 seed=TCAAAAAACG Num.seqs=4 Similarity=1.000000 tpcCG=39.73249 1 ATCGTT--TTTTG

Rev.of_arqueas_representative:NZ_CP009528.1_Methanosarcina_barkeri_MS:82:2385899-2386133 Satlength=235 Nr of Repeats=18 RepeatLength=13 seed=TCAAAAATAA Num.seqs=18 Similarity=1.000000 tpcCG=39.168999 1 AACATTATTTTTG

* * ** *****

Consensus:

AaCaTTTTTTG

>arqueas_representative_Fam_199_12_3 Nr. of seq. 3 Alignment length(with gaps) = 13 Alignment score = 0.649573

arqueas_representative:NC_003552.1_Methanosarcina_acetivorans_str._C2A:80:3333740-3333785 Satlength=46 Nr of Repeats=4 RepeatLength=11 seed=TTCTCTTACT Num.seqs=3 Similarity=1.000000 tpcCG=42.700001 0 TTCTCTTACTG--

arqueas_representative:CP009516.1_Methanosarcina_horonobensis_HB-1:83:2009913-2009965 Satlength=53 Nr of Repeats=4 RepeatLength=13 seed=TTACTGATTT Num.seqs=4 Similarity=0.948718 tpcCG=41.299999 5 TTCTGTTACTGAT

Rev.of_arqueas_representative:NZ_CP009517.1_Methanosarcina_barkeri_3:81:2052925-2053093 Satlength=169 Nr of Repeats=13 RepeatLength=12 seed=TAATCAGTAA Num.seqs=12 Similarity=1.000000 tpcCG=39.099998 3 TTACCTTACTGA-

** ******

Consensus:

TTctcTTACTGa

>arqueas_representative_Fam_200_11_3 Nr. of seq. 3 Alignment length(with gaps) = 13 Alignment score = 0.675214

arqueas_representative:NZ_CP009528.1_Methanosarcina_barkeri_MS:82:3908056-3908122 Satlength=67 Nr of Repeats=6 RepeatLength=11 seed=GTTTATCTTT Num.seqs=6 Similarity=1.000000 tpcCG=39.168999 0 GTTTATC-T-TTT

Rev.of_arqueas_representative:NZ_CP009528.1_Methanosarcina_barkeri_MS:82:1067939-1068251 Satlength=313 Nr of Repeats=7 RepeatLength=12 seed=TAAACAAAAC Num.seqs=5 Similarity=0.823077 tpcCG=39.168999 5 GTTTATCGT-TTT

arqueas_representative:NZ_CP009515.1_Methanosarcina_lacustris_Z-7289:84:3785506-3785566 Satlength=61 Nr of Repeats=5 RepeatLength=12 seed=ATCTGTTTGC Num.seqs=5 Similarity=1.000000 tpcCG=41.799999 4 GCTTATC-TGTTT

* ***** * ***

Consensus:

GtTTATCTTTT

>arqueas_representative_Fam_201_13_3 Nr. of seq. 3 Alignment length(with gaps) = 13 Alignment score = 0.709402

arqueas_representative:NZ_CP009512.1_Methanosarcina_mazei_S-6:85:2641638-2641715 Satlength=78 Nr of Repeats=7 RepeatLength=11 seed=TTTTGAATAG Num.seqs=7 Similarity=1.000000 tpcCG=41.400002 0 TTTTGAATAGC--

Rev.of_arqueas_representative:NZ_CP009517.1_Methanosarcina_barkeri_3:81:2451007-2451072 Satlength=66 Nr of Repeats=5 RepeatLength=13 seed=AAAATCTCTA Num.seqs=5 Similarity=1.000000 tpcCG=39.099998 4 TTTTGAATAGAGA

arqueas_representative:NZ_CP009528.1_Methanosarcina_barkeri_MS:82:3015095-3015238 Satlength=144 Nr of Repeats=11 RepeatLength=13 seed=TACTGATTTT Num.seqs=11 Similarity=0.955245 tpcCG=39.168999 7 TTTTGAATACTGA

*********

Consensus:

TTTTGAATAgnga

>arqueas_representative_Fam_202_12_3 Nr. of seq. 3 Alignment length(with gaps) = 13 Alignment score = 0.632479

arqueas_representative:NC_017941.2_Haloferax_mediterranei_ATCC_33500:25:2552573-2552627 Satlength=55 Nr of Repeats=4 RepeatLength=12 seed=TCCAAGTCCA Num.seqs=3 Similarity=1.000000 tpcCG=60.255100 0 TC-CAAGTCCAAG

Rev.of_arqueas_representative:NZ_LT607756.1_Methanobacterium_congolense_isolate_Buetzberg_:44:1982575-1982635 Satlength=61 Nr of Repeats=5 RepeatLength=12 seed=AGGACTTGTA Num.seqs=5 Similarity=0.86666 4 TC-CTACTACAAG

arqueas_representative:NZ_CP009520.1_Methanosarcina_vacuolata_Z-761:88:1144818-1144883 Satlength=66 Nr of Repeats=5 RepeatLength=13 seed=TCAATTACAA Num.seqs=5 Similarity=0.917949 tpcCG=39.732498 2 TCTCAATTACAAG

** * * * ****

Consensus:

TCCaAnTaCAAG

>arqueas_representative_Fam_203_13_3 Nr. of seq. 3 Alignment length(with gaps) = 13 Alignment score = 0.632479

arqueas_representative:NC_009635.1_Methanococcus_aeolicus_Nankai-3:61:594067-594121 Satlength=55 Nr of Repeats=4 RepeatLength=12 seed=ATATACATAT Num.seqs=3 Similarity=1.000000 tpcCG=30.000000 0 ATATACATATAC-

arqueas_representative:NZ_CP009517.1_Methanosarcina_barkeri_3:81:254560-254690 Satlength=131 Nr of Repeats=6 RepeatLength=13 seed=TTAACATTTA Num.seqs=4 Similarity=0.897436 tpcCG=39.099998 1 ATTAACATTTACT

Rev.of_arqueas_representative:NZ_CP009528.1_Methanosarcina_barkeri_MS:82:2771421-2771587 Satlength=167 Nr of Repeats=13 RepeatLength=13 seed=AAAGTATATG Num.seqs=11 Similarity=1.000000 tpcCG=39.168999 2 TTTCACATATACT

* **** ***

Consensus:

aTtnACATaTACt

>arqueas_representative_Fam_204_12_3 Nr. of seq. 3 Alignment length(with gaps) = 13 Alignment score = 0.641026

arqueas_representative:NC_018227.2_Methanoculleus_bourgensis_MS2T_:67:976650-976722 Satlength=73 Nr of Repeats=6 RepeatLength=12 seed=ACTTCCGTGG Num.seqs=6 Similarity=1.000000 tpcCG=60.599998 0 ACTTCCGTGGTA-

Rev.of_arqueas_representative:NZ_CP009515.1_Methanosarcina_lacustris_Z-7289:84:1714239-1714287 Satlength=49 Nr of Repeats=4 RepeatLength=12 seed=ACCCCGGAAG Num.seqs=4 Similarity=0.944444 tpcCG=41.7999 11 ACTTCCGGGGTG-

Rev.of_arqueas_representative:NZ_CP009528.1_Methanosarcina_barkeri_MS:82:481591-481721 Satlength=131 Nr of Repeats=10 RepeatLength=13 seed=CACTGAAGTA Num.seqs=10 Similarity=1.000000 tpcCG=39.168999 9 ACTTCAGTGATAT

***** * * *

Consensus:

ACTTCcGtGgTa

>arqueas_representative_Fam_205_12_3 Nr. of seq. 3 Alignment length(with gaps) = 13 Alignment score = 0.709402

arqueas_representative:NZ_CP009517.1_Methanosarcina_barkeri_3:81:538685-538829 Satlength=145 Nr of Repeats=12 RepeatLength=12 seed=TGAAGATGGG Num.seqs=12 Similarity=0.981481 tpcCG=39.099998 0 TGAAGATGGGTA-

Rev.of_arqueas_representative:NZ_CP009520.1_Methanosarcina_vacuolata_Z-761:88:3477230-3477338 Satlength=109 Nr of Repeats=8 RepeatLength=12 seed=TCTTCTTACC Num.seqs=5 Similarity=1.000000 tpcCG=39.7324 6 AGAAGATTGGTA-

arqueas_representative:NZ_CP009528.1_Methanosarcina_barkeri_MS:82:2656856-2656921 Satlength=66 Nr of Repeats=5 RepeatLength=13 seed=GAAGATTGGT Num.seqs=5 Similarity=1.000000 tpcCG=39.168999 1 TGAAGATTGGTCT

****** ***

Consensus:

tGAAGATtGGTa

>arqueas_representative_Fam_206_12_3 Nr. of seq. 3 Alignment length(with gaps) = 13 Alignment score = 0.666667

arqueas_representative:NZ_CP009515.1_Methanosarcina_lacustris_Z-7289:84:437667-437715 Satlength=49 Nr of Repeats=4 RepeatLength=12 seed=CACCTTTTTC Num.seqs=4 Similarity=1.000000 tpcCG=41.799999 0 CACCT-TTTTCTC

Rev.of_arqueas_representative:NZ_CP009520.1_Methanosarcina_vacuolata_Z-761:88:4493798-4493846 Satlength=49 Nr of Repeats=4 RepeatLength=12 seed=GGAGAAGAAG Num.seqs=4 Similarity=0.888889 tpcCG=39.73249 1 CACCT-TCTTCTC

Rev.of_arqueas_representative:NZ_CP009512.1_Methanosarcina_mazei_S-6:85:798798-798850 Satlength=53 Nr of Repeats=4 RepeatLength=13 seed=AAAATAGGTT Num.seqs=4 Similarity=0.931624 tpcCG=41.400002 10 AACCTATTTTGTC

**** * ** **

Consensus:

cACCTTtTTcTC

>arqueas_representative_Fam_207_13_3 Nr. of seq. 3 Alignment length(with gaps) = 13 Alignment score = 0.623932

arqueas_representative:NZ_CP009515.1_Methanosarcina_lacustris_Z-7289:84:2821252-2821312 Satlength=61 Nr of Repeats=5 RepeatLength=12 seed=GGACTGGAAG Num.seqs=5 Similarity=0.888889 tpcCG=41.799999 0 GGACTGG-AAGCA

arqueas_representative:NC_003552.1_Methanosarcina_acetivorans_str._C2A:80:4259848-4259952 Satlength=105 Nr of Repeats=8 RepeatLength=13 seed=GAAAGAAGGA Num.seqs=8 Similarity=0.956044 tpcCG=42.700001 6 GGAGTGGAAAGAA

Rev.of_arqueas_representative:NZ_CP009520.1_Methanosarcina_vacuolata_Z-761:88:318010-318075 Satlength=66 Nr of Repeats=5 RepeatLength=13 seed=CTGCTTTATA Num.seqs=5 Similarity=0.917949 tpcCG=39.732498 14 GGAGTATAAAGCA

*** * *** *

Consensus:

GGAgTggaAAGcA

>arqueas_representative_Fam_208_12_3 Nr. of seq. 3 Alignment length(with gaps) = 13 Alignment score = 0.675214

arqueas_representative:NZ_CP009512.1_Methanosarcina_mazei_S-6:85:148745-148935 Satlength=191 Nr of Repeats=6 RepeatLength=12 seed=TATTTTCCTG Num.seqs=4 Similarity=0.944444 tpcCG=41.400002 0 TATTTTCCTGAA-

Rev.of_arqueas_representative:NZ_CP009520.1_Methanosarcina_vacuolata_Z-761:88:1783969-1784137 Satlength=169 Nr of Repeats=14 RepeatLength=12 seed=ATAATCTGGA Num.seqs=14 Similarity=0.875458 tpcCG=39.73 3 TATTTTCCAGAT-

Rev.of_arqueas_representative:NZ_CP009515.1_Methanosarcina_lacustris_Z-7289:84:3145801-3145996 Satlength=196 Nr of Repeats=15 RepeatLength=13 seed=TACGGAAAAT Num.seqs=15 Similarity=1.000000 tpcCG=41.7 11 TATTTTCCGTAAG

******** *

Consensus:

TATTTTCCngAa

>arqueas_representative_Fam_209_13_3 Nr. of seq. 3 Alignment length(with gaps) = 13 Alignment score = 0.794872

arqueas_representative:NZ_CP009517.1_Methanosarcina_barkeri_3:81:2135252-2135343 Satlength=92 Nr of Repeats=7 RepeatLength=13 seed=TATTCACAAA Num.seqs=7 Similarity=1.000000 tpcCG=39.099998 0 TATTCACAAATGA

Rev.of_arqueas_representative:NZ_CP009520.1_Methanosarcina_vacuolata_Z-761:88:1410833-1410924 Satlength=92 Nr of Repeats=7 RepeatLength=13 seed=TGTGAATATC Num.seqs=7 Similarity=0.853480 tpcCG=39.73249 8 TATTCACAATTGA

Rev.of_arqueas_representative:NZ_CP009517.1_Methanosarcina_barkeri_3:81:3131700-3131811 Satlength=112 Nr of Repeats=5 RepeatLength=13 seed=TAATGTCAAT Num.seqs=3 Similarity=1.000000 tpcCG=39.099998 5 CATTAACAATTGA

*** **** ***

Consensus:

tATTcACAAtTGA

>arqueas_representative_Fam_210_13_3 Nr. of seq. 3 Alignment length(with gaps) = 13 Alignment score = 0.692308

arqueas_representative:NZ_CP009517.1_Methanosarcina_barkeri_3:81:4393712-4393791 Satlength=80 Nr of Repeats=6 RepeatLength=13 seed=TTATTAAAAA Num.seqs=5 Similarity=0.917949 tpcCG=39.099998 0 TTATTAAAAAGTG

arqueas_representative:NZ_CP009528.1_Methanosarcina_barkeri_MS:82:1870599-1870766 Satlength=168 Nr of Repeats=13 RepeatLength=13 seed=GTTATTGAAA Num.seqs=11 Similarity=0.891841 tpcCG=39.168999 12 TTATTGAAAACAG

Rev.of_arqueas_representative:NZ_CP009515.1_Methanosarcina_lacustris_Z-7289:84:3434616-3434668 Satlength=53 Nr of Repeats=4 RepeatLength=13 seed=AACCCTTTTC Num.seqs=4 Similarity=0.846154 tpcCG=41.7999 2 TTAATGAAAAGGG

*** * **** *

Consensus:

TTAtTgAAAAgnG

>arqueas_representative_Fam_211_13_3 Nr. of seq. 3 Alignment length(with gaps) = 13 Alignment score = 0.658120

arqueas_representative:NZ_CP009528.1_Methanosarcina_barkeri_MS:82:3870245-3870310 Satlength=66 Nr of Repeats=5 RepeatLength=13 seed=TTTTTATTTG Num.seqs=5 Similarity=0.876923 tpcCG=39.168999 0 TTTTTATTTGGAC

Rev.of_arqueas_representative:NZ_CP009506.1_Methanosarcina_siciliae_T4/M:86:2847546-2847598 Satlength=53 Nr of Repeats=4 RepeatLength=13 seed=GTGAAGATAA Num.seqs=4 Similarity=0.931624 tpcCG=42.900002 0 TTTTTATCTTCAC

arqueas_representative:NZ_CP009520.1_Methanosarcina_vacuolata_Z-761:88:3420979-3421044 Satlength=66 Nr of Repeats=5 RepeatLength=13 seed=GTATATGAAC Num.seqs=5 Similarity=0.917949 tpcCG=39.732498 3 TTTGTATATGAAC

*** *** * **

Consensus:

TTTtTATnTgnAC

>arqueas_representative_Fam_212_10_3 Nr. of seq. 3 Alignment length(with gaps) = 12 Alignment score = 0.657407

arqueas_representative:NZ_LT607756.1_Methanobacterium_congolense_isolate_Buetzberg_:44:2312757-2312847 Satlength=91 Nr of Repeats=8 RepeatLength=10 seed=GATTTGGAAT Num.seqs=7 Similarity=1.000000 tpcCG 0 GATTT-G-GAAT

arqueas_representative:NZ_CP009528.1_Methanosarcina_barkeri_MS:82:2647930-2647980 Satlength=51 Nr of Repeats=4 RepeatLength=10 seed=ATTTAGGAAT Num.seqs=3 Similarity=1.000000 tpcCG=39.168999 0 -ATTTAG-GAAT

arqueas_representative:NZ_CP009512.1_Methanosarcina_mazei_S-6:85:3953442-3953486 Satlength=45 Nr of Repeats=4 RepeatLength=11 seed=ATATTTAGAG Num.seqs=4 Similarity=0.939394 tpcCG=41.400002 9 -ATTTAGAGAAT

**** * ****

Consensus:

ATTTaGGAAT

>arqueas_representative_Fam_213_11_3 Nr. of seq. 3 Alignment length(with gaps) = 12 Alignment score = 0.638889

arqueas_representative:NC_015574.1_Methanobacterium_paludis__SWAN1:47:528000-528563 Satlength=564 Nr of Repeats=15 RepeatLength=11 seed=AATTTTGAAC Num.seqs=14 Similarity=1.000000 tpcCG=35.700001 0 AATTTTGAA-CT

arqueas_representative:NZ_CP009528.1_Methanosarcina_barkeri_MS:82:2568434-2568500 Satlength=67 Nr of Repeats=6 RepeatLength=11 seed=GAACTTTGAA Num.seqs=6 Similarity=0.846465 tpcCG=39.168999 10 AACTTTGAA-CG

arqueas_representative:NZ_CP009517.1_Methanosarcina_barkeri_3:81:2404102-2404174 Satlength=73 Nr of Repeats=6 RepeatLength=12 seed=AGCTAATTTG Num.seqs=6 Similarity=1.000000 tpcCG=39.099998 8 AATTTGGAAGCT

** ** *** *

Consensus:

AAtTTtGAACt

>arqueas_representative_Fam_214_12_3 Nr. of seq. 3 Alignment length(with gaps) = 12 Alignment score = 0.675926

arqueas_representative:NC_015574.1_Methanobacterium_paludis__SWAN1:47:826195-826382 Satlength=188 Nr of Repeats=17 RepeatLength=11 seed=GTTTGCACTC Num.seqs=17 Similarity=1.000000 tpcCG=35.700001 0 GTTTGCACTCA-

arqueas_representative:NC_003552.1_Methanosarcina_acetivorans_str._C2A:80:3585882-3585978 Satlength=97 Nr of Repeats=7 RepeatLength=12 seed=CATTCAGGTT Num.seqs=6 Similarity=0.962963 tpcCG=42.700001 5 GTTTCCATTCAG

arqueas_representative:NZ_CP009528.1_Methanosarcina_barkeri_MS:82:704067-704196 Satlength=130 Nr of Repeats=8 RepeatLength=12 seed=AGGTTTACTT Num.seqs=5 Similarity=0.712821 tpcCG=39.168999 10 GTTTACTTTCAG

**** * ***

Consensus:

GTTTnCatTCAg

>arqueas_representative_Fam_215_11_3 Nr. of seq. 3 Alignment length(with gaps) = 12 Alignment score = 0.611111

arqueas_representative:NZ_CP009517.1_Methanosarcina_barkeri_3:81:1036890-1037033 Satlength=144 Nr of Repeats=13 RepeatLength=11 seed=TTGGCTTTCT Num.seqs=13 Similarity=0.944056 tpcCG=39.099998 0 TTGGCTTTCTA-

Rev.of_arqueas_representative:NZ_CP009528.1_Methanosarcina_barkeri_MS:82:2913934-2914099 Satlength=166 Nr of Repeats=11 RepeatLength=11 seed=GTGAAAGCCA Num.seqs=10 Similarity=1.000000 tpcCG=39.168999 0 TTGGCTTTC-AC

arqueas_representative:NZ_CP009515.1_Methanosarcina_lacustris_Z-7289:84:3009426-3009505 Satlength=80 Nr of Repeats=7 RepeatLength=11 seed=ATCTGGCTTT Num.seqs=6 Similarity=0.959596 tpcCG=41.799999 9 CTGGCTTTT-AT

******* *

Consensus:

tTGGCTTTcAn

>arqueas_representative_Fam_216_11_3 Nr. of seq. 3 Alignment length(with gaps) = 12 Alignment score = 0.685185

arqueas_representative:NZ_CP009517.1_Methanosarcina_barkeri_3:81:1951931-1952052 Satlength=122 Nr of Repeats=10 RepeatLength=11 seed=ATTCAGTATC Num.seqs=9 Similarity=0.878788 tpcCG=39.099998 0 ATTCAGTATCC-

Rev.of_arqueas_representative:NZ_CP009528.1_Methanosarcina_barkeri_MS:82:1803846-1803894 Satlength=49 Nr of Repeats=4 RepeatLength=12 seed=ATAAGATACT Num.seqs=4 Similarity=0.944444 tpcCG=39.168999 2 ATTCAGTATCTT

arqueas_representative:NZ_CP009515.1_Methanosarcina_lacustris_Z-7289:84:4036635-4036745 Satlength=111 Nr of Repeats=10 RepeatLength=11 seed=TAATCAATAT Num.seqs=10 Similarity=1.000000 tpcCG=41.799999 10 AATCAATATCT-

* *** ****

Consensus:

AtTCAgTATCt

>arqueas_representative_Fam_217_12_3 Nr. of seq. 3 Alignment length(with gaps) = 12 Alignment score = 0.712963

arqueas_representative:NZ_CP009517.1_Methanosarcina_barkeri_3:81:2514261-2514327 Satlength=67 Nr of Repeats=6 RepeatLength=11 seed=TAAGGGAAAT Num.seqs=6 Similarity=0.878788 tpcCG=39.099998 0 TAAGGGA-AATT

Rev.of_arqueas_representative:NZ_CP009520.1_Methanosarcina_vacuolata_Z-761:88:2250652-2250762 Satlength=111 Nr of Repeats=10 RepeatLength=11 seed=TAATTATCCC Num.seqs=10 Similarity=0.975758 tpcCG=39.73 1 -AAGGGATAATT

Rev.of_arqueas_representative:NZ_CP009517.1_Methanosarcina_barkeri_3:81:1057587-1057671 Satlength=85 Nr of Repeats=7 RepeatLength=12 seed=TATCCCTTCA Num.seqs=7 Similarity=0.936508 tpcCG=39.099998 9 GAAGGGATATTT

****** * **

Consensus:

nAAGGGAtAaTT

>arqueas_representative_Fam_218_12_3 Nr. of seq. 3 Alignment length(with gaps) = 12 Alignment score = 0.712963

arqueas_representative:NZ_CP009517.1_Methanosarcina_barkeri_3:81:3715193-3715354 Satlength=162 Nr of Repeats=11 RepeatLength=11 seed=AAGATAGATA Num.seqs=10 Similarity=1.000000 tpcCG=39.099998 0 AAGATAGATAA-

Rev.of_arqueas_representative:NZ_CP009517.1_Methanosarcina_barkeri_3:81:353333-353417 Satlength=85 Nr of Repeats=7 RepeatLength=12 seed=GTATTATCTA Num.seqs=7 Similarity=1.000000 tpcCG=39.099998 2 ACGATAGATAAT

arqueas_representative:NZ_CP009515.1_Methanosarcina_lacustris_Z-7289:84:3955870-3955991 Satlength=122 Nr of Repeats=11 RepeatLength=11 seed=GTAGATAACA Num.seqs=11 Similarity=0.960331 tpcCG=41.799999 2 AAG-TAGATAAC

* * *******

Consensus:

AaGaTAGATAAn

>arqueas_representative_Fam_219_12_3 Nr. of seq. 3 Alignment length(with gaps) = 12 Alignment score = 0.703704

arqueas_representative:NZ_CP009515.1_Methanosarcina_lacustris_Z-7289:84:3829774-3830473 Satlength=700 Nr of Repeats=12 RepeatLength=11 seed=TGTAAAAGAA Num.seqs=11 Similarity=0.977961 tpcCG=41.799999 4 GAA-ATGTAAAA

Rev.of_arqueas_representative:NC_003552.1_Methanosarcina_acetivorans_str._C2A:80:458623-458719 Satlength=97 Nr of Repeats=8 RepeatLength=12 seed=TTCTTTTACA Num.seqs=8 Similarity=0.952381 tpcCG=42.7000 14 GAAGATGTAAAA

Rev.of_arqueas_representative:NZ_CP009506.1_Methanosarcina_siciliae_T4/M:86:2106304-2106400 Satlength=97 Nr of Repeats=8 RepeatLength=12 seed=TTTTGTTTTA Num.seqs=8 Similarity=0.904762 tpcCG=42.900002 4 CAAAATCTAAAA

* ***** ** *

Consensus:

TgTAAAAgAAnA

>arqueas_representative_Fam_220_12_3 Nr. of seq. 3 Alignment length(with gaps) = 12 Alignment score = 0.722222

arqueas_representative:NC_017941.2_Haloferax_mediterranei_ATCC_33500:25:995237-995311 Satlength=75 Nr of Repeats=6 RepeatLength=12 seed=AGAGGAAGGG Num.seqs=4 Similarity=0.925926 tpcCG=60.255100 0 AGAGGAAGGGGA

arqueas_representative:NC_014408.1_Methanothermobacter_marburgensis_str._Marburg:92:259514-259895 Satlength=382 Nr of Repeats=5 RepeatLength=12 seed=GAAGAGGAAG Num.seqs=3 Similarity=0.740741 tpcCG=48. 4 AGANGAAGAGGA

Rev.of_arqueas_representative:NZ_CP009515.1_Methanosarcina_lacustris_Z-7289:84:356946-357054 Satlength=109 Nr of Repeats=6 RepeatLength=12 seed=CCTTTTCCCC Num.seqs=4 Similarity=0.944444 tpcCG=41.79999 5 AAAGGTAGGGGA

* * * ** ***

Consensus:

AgAgGaAGgGGA

>arqueas_representative_Fam_221_12_3 Nr. of seq. 3 Alignment length(with gaps) = 12 Alignment score = 0.703704

arqueas_representative:NC_008212.1_Haloquadratum_walsbyi_DSM_16790:31:1261534-1261588 Satlength=55 Nr of Repeats=4 RepeatLength=12 seed=GTGTCGGTGT Num.seqs=3 Similarity=1.000000 tpcCG=47.694099 0 GTGTCGGTGTCG

arqueas_representative:NC_013922.1_Natrialba_magadii_ATCC_43099:100:1221756-1221840 Satlength=85 Nr of Repeats=7 RepeatLength=12 seed=GTCTGTGTCT Num.seqs=7 Similarity=0.883598 tpcCG=61.032001 2 GTGTCTGTGTCT

arqueas_representative:NC_019977.1_Methanomethylovorans_hollandica_DSM_15978:75:1389022-1389118 Satlength=97 Nr of Repeats=8 RepeatLength=12 seed=TGACTGGGTC Num.seqs=8 Similarity=1.000000 tpcCG=41.843 1 GTGACTGGGTCG

*** * * ***

Consensus:

GTGtCtGtGTCg

>arqueas_representative_Fam_222_12_3 Nr. of seq. 3 Alignment length(with gaps) = 12 Alignment score = 0.703704

arqueas_representative:NC_008212.1_Haloquadratum_walsbyi_DSM_16790:31:2044825-2044933 Satlength=109 Nr of Repeats=9 RepeatLength=12 seed=TGGGTTTGGG Num.seqs=9 Similarity=1.000000 tpcCG=47.694099 0 TGGGTTTGGGTT

arqueas_representative:NZ_CP009515.1_Methanosarcina_lacustris_Z-7289:84:2694997-2695049 Satlength=53 Nr of Repeats=4 RepeatLength=12 seed=GGTTTTGGTT Num.seqs=3 Similarity=1.000000 tpcCG=41.799999 1 TGGTTTTGGTTT

Rev.of_arqueas_representative:NZ_CP009520.1_Methanosarcina_vacuolata_Z-761:88:4085788-4085848 Satlength=61 Nr of Repeats=5 RepeatLength=12 seed=AAAGCATAAC Num.seqs=5 Similarity=0.933333 tpcCG=39.73249 0 TGGTTATGCTTT

*** * ** **

Consensus:

TGGtTtTGgtTT

>arqueas_representative_Fam_223_12_3 Nr. of seq. 3 Alignment length(with gaps) = 12 Alignment score = 0.703704

arqueas_representative:NZ_CP014265.1_Methanobrevibacter_olleyae__YLM1:49:618826-618898 Satlength=73 Nr of Repeats=6 RepeatLength=12 seed=CTTTAAGATA Num.seqs=6 Similarity=0.844444 tpcCG=26.900000 0 CTTTAAGATATT

arqueas_representative:NC_014222.1_Methanococcus_voltae_A3:65:913022-913070 Satlength=49 Nr of Repeats=4 RepeatLength=12 seed=TTATTTTTTA Num.seqs=4 Similarity=1.000000 tpcCG=28.600000 7 TTTTAAGTTATT

Rev.of_arqueas_representative:NZ_CP009520.1_Methanosarcina_vacuolata_Z-761:88:2874987-2875104 Satlength=118 Nr of Repeats=7 RepeatLength=12 seed=AACTCAATGA Num.seqs=6 Similarity=0.867521 tpcCG=39.7324 9 CATTGAGTTATT

** ** ****

Consensus:

ctTTaAGtTATT

>arqueas_representative_Fam_224_12_3 Nr. of seq. 3 Alignment length(with gaps) = 12 Alignment score = 0.851852

arqueas_representative:NZ_CP009528.1_Methanosarcina_barkeri_MS:82:885331-885427 Satlength=97 Nr of Repeats=8 RepeatLength=12 seed=ATTTAATTCT Num.seqs=8 Similarity=1.000000 tpcCG=39.168999 2 TTATTTAATTCT

Rev.of_arqueas_representative:NZ_CP009528.1_Methanosarcina_barkeri_MS:82:1523396-1523480 Satlength=85 Nr of Repeats=6 RepeatLength=12 seed=GAAGAATCAA Num.seqs=5 Similarity=0.955556 tpcCG=39.168999 2 TCATTTGATTCT

arqueas_representative:NZ_CP009520.1_Methanosarcina_vacuolata_Z-761:88:2279988-2280108 Satlength=121 Nr of Repeats=10 RepeatLength=12 seed=CTTCATTTGA Num.seqs=10 Similarity=1.000000 tpcCG=39.732498 10 TCATTTGATTCT

**** ******

Consensus:

ATTTgATTCTTc

>arqueas_representative_Fam_225_10_3 Nr. of seq. 3 Alignment length(with gaps) = 11 Alignment score = 0.606061

arqueas_representative:NZ_CP009517.1_Methanosarcina_barkeri_3:81:3021906-3021976 Satlength=71 Nr of Repeats=5 RepeatLength=10 seed=TGGCTTGAAA Num.seqs=4 Similarity=0.933333 tpcCG=39.099998 0 TGGCTTG-AAA

arqueas_representative:NZ_CP009528.1_Methanosarcina_barkeri_MS:82:2038183-2038253 Satlength=71 Nr of Repeats=6 RepeatLength=10 seed=AAATTGTTTG Num.seqs=5 Similarity=0.946667 tpcCG=39.168999 7 TTGTTTG-AAA

arqueas_representative:NZ_CP009520.1_Methanosarcina_vacuolata_Z-761:88:1870154-1870231 Satlength=78 Nr of Repeats=7 RepeatLength=11 seed=ATTTGTAAAT Num.seqs=7 Similarity=1.000000 tpcCG=39.732498 12 TTATTTGTAAA

* *** ***

Consensus:

TtgtTTGAAA

>arqueas_representative_Fam_226_10_3 Nr. of seq. 3 Alignment length(with gaps) = 11 Alignment score = 0.737374

arqueas_representative:NZ_CP009515.1_Methanosarcina_lacustris_Z-7289:84:3477343-3477423 Satlength=81 Nr of Repeats=6 RepeatLength=10 seed=AAGGAAAGAG Num.seqs=4 Similarity=0.911111 tpcCG=41.799999 0 AAGGAAAGAG-

Rev.of_arqueas_representative:NZ_CP009506.1_Methanosarcina_siciliae_T4/M:86:5003908-5003987 Satlength=80 Nr of Repeats=7 RepeatLength=11 seed=TCTTTCCTTT Num.seqs=5 Similarity=0.903030 tpcCG=42.900002 9 AAGGAAAGATA

Rev.of_arqueas_representative:NZ_CP009520.1_Methanosarcina_vacuolata_Z-761:88:41054-41225 Satlength=172 Nr of Repeats=17 RepeatLength=10 seed=ACTTTCCTTA Num.seqs=16 Similarity=1.000000 tpcCG=39.732498 9 AAGGAAAGTT-

********

Consensus:

AAGGAAAGat

>arqueas_representative_Fam_227_11_3 Nr. of seq. 3 Alignment length(with gaps) = 11 Alignment score = 0.676768

arqueas_representative:NC_015574.1_Methanobacterium_paludis__SWAN1:47:1392529-1392716 Satlength=188 Nr of Repeats=17 RepeatLength=11 seed=ACAATCAAAC Num.seqs=17 Similarity=1.000000 tpcCG=35.700001 0 ACAATCAAACT

Rev.of_arqueas_representative:NC_018227.2_Methanoculleus_bourgensis_MS2T_:67:643411-643598 Satlength=188 Nr of Repeats=17 RepeatLength=11 seed=GATTGGAGTA Num.seqs=17 Similarity=0.985740 tpcCG=60.59999 6 CCAATCATACT

arqueas_representative:NZ_CP009517.1_Methanosarcina_barkeri_3:81:1392042-1392088 Satlength=47 Nr of Repeats=4 RepeatLength=11 seed=GAAACTAAAA Num.seqs=3 Similarity=1.000000 tpcCG=39.099998 5 AAAATGAAACT

*** * ***

Consensus:

acAATcAaACT

>arqueas_representative_Fam_228_10_3 Nr. of seq. 3 Alignment length(with gaps) = 10 Alignment score = 0.644444

arqueas_representative:NZ_CP009517.1_Methanosarcina_barkeri_3:81:37804-37894 Satlength=91 Nr of Repeats=8 RepeatLength=10 seed=TCTTCGCATG Num.seqs=7 Similarity=1.000000 tpcCG=39.099998 0 TCTTCGCATG

Rev.of_arqueas_representative:NZ_CP009517.1_Methanosarcina_barkeri_3:81:1540789-1540869 Satlength=81 Nr of Repeats=7 RepeatLength=10 seed=GGAAGACAAT Num.seqs=6 Similarity=1.000000 tpcCG=39.099998 6 TCTTCCATTG

arqueas_representative:NZ_CP009515.1_Methanosarcina_lacustris_Z-7289:84:40656-40716 Satlength=61 Nr of Repeats=5 RepeatLength=10 seed=CCTTTTCTTC Num.seqs=4 Similarity=1.000000 tpcCG=41.799999 15 TCTTCCCTTT

***** *

Consensus:

TCTTCcctTg

>arqueas_representative_Fam_229_261_2 Nr. of seq. 2 Alignment length(with gaps) = 261 Alignment score = 0.833972

arqueas_representative:NC_003552.1_Methanosarcina_acetivorans_str._C2A:80:3891831-3892863 Satlength=1033 Nr of Repeats=4 RepeatLength=258 seed=GTTTCGGTAT Num.seqs=4 Similarity=0.799371 tpcCG=42.700001 0 GTTTCGGTATCCGTGCCGTTTGCATTGCTAACCGTCAGTTTGGCTTCATAAGTCCCTCTGGAATTGTAAATATAAGCAAAGCTTGTTTCATTAGAGTCCTCGACTCCGTCACCATTAACATCCCAGCT---TCTCGAAGTTGCATTCTCCGAAAGGTCGGTAAAGAGAACCGTAAGGGGGTAGTAACCGCTGGTTTTATTCAATGTGAAGTTTGCTACGGGAAGAACAGGAAGTTCTTCTTCCAGCACGGTAATCACAGCA

arqueas_representative:NZ_CP009506.1_Methanosarcina_siciliae_T4/M:86:1433863-1434895 Satlength=1033 Nr of Repeats=4 RepeatLength=258 seed=GTATCCGTGC Num.seqs=4 Similarity=0.978467 tpcCG=42.900002 6 GTTTCGGTATCCGTGCCGTTTGCATTGCTCACGGTCAGTTTTGCTTCATAAGTCCCTCTGGAAGTGTATGTGTAAGCAAAGCTTGCCGCATTAGAGTCTTCGACCCCGTCACCATTAACATCCCAGCTCAATC-CG--GTTGCATTCTGCGAAGTGTCGGTGAAGAGGACAGTAAGAGGGTAATACCCGCTGGTTTTATTCACTGTGAAGTTTGCTACGGGAAGAATAGGAATTTCTTCTTCCATCACGGTAATCACAGCA

***************************** ** ******** ********************* **** * ************* ********** ***** *********************** ** ** ********** **** ****** ***** ** ***** ***** ** **************** *********************** ***** *********** ****************

Consensus:

GTTTCGGTATCCGTGCCGTTTGCATTGCTaACcGTCAGTTTgGCTTCATAAGTCCCTCTGGAAgTGTAaaTaTAAGCAAAGCTTGccgCATTAGAGTCcTCGACcCCGTCACCATTAACATCCCAGCTcaaTCtCGaaGTTGCATTCTcCGAAagGTCGGTaAAGAGaACaGTAAGaGGGTAaTAaCCGCTGGTTTTATTCAaTGTGAAGTTTGCTACGGGAAGAAcAGGAAgTTCTTCTTCCAgCACGGTAATCACAGCA

>arqueas_representative_Fam_230_159_2 Nr. of seq. 2 Alignment length(with gaps) = 159 Alignment score = 0.781971

arqueas_representative:NC_014507.1_Methanolacinia_petrolearia_DSM_11571:73:33284-33923 Satlength=640 Nr of Repeats=4 RepeatLength=159 seed=TATGACCTTA Num.seqs=3 Similarity=0.751223 tpcCG=47.400002 0 TATGACCTTATGATCGGTGCANNTNAAGGAGTNACATATGGTTATGAGAATACCGGGTCTTCGGGTAGCCCGGAGTGGACTGCGAAATCTTCNTGGAATACCCCTGATATAGGTANTGCTGCANCACCAGCTTTTGCCGATCTTGACGGTGATGGTGAC

Rev.of_arqueas_representative:NC_003552.1_Methanosarcina_acetivorans_str._C2A:80:4567241-4567877 Satlength=637 Nr of Repeats=4 RepeatLength=159 seed=TATTCTCATA Num.seqs=4 Similarity=0.708765 tpcCG=42. 52 TATGACCTTATGATCGGTGAATCTGNTGGAGTTACATATGGTTATGAGAATACCGGGTCTTCGGACAGCCCGGTGTGGGCTGCGAACTCTTCCTGGAATATTCCTTCTGTAGGTTCTTGTTCATCACCAGCTTTGGCCGACCTTGACTGTGATGGCGAC

******************* * * ***** ******************************* ******* **** ******* ***** ******* *** * ***** * * ** ********** ***** ****** ******* ***

Consensus:

TATGACCTTATGATCGGTGaAtcTgaaGGAGTtACATATGGTTATGAGAATACCGGGTCTTCGGacAGCCCGGaGTGGaCTGCGAAaTCTTCcTGGAATAccCCTgaTaTAGGTacTgcTgCAtCACCAGCTTTgGCCGAcCTTGACgGTGATGGcGAC

>arqueas_representative_Fam_231_158_2 Nr. of seq. 2 Alignment length(with gaps) = 158 Alignment score = 0.618143

arqueas_representative:CP001463.1_Thermococcus_sibiricus_MM_739:135:1293310-1294246 Satlength=937 Nr of Repeats=6 RepeatLength=156 seed=TACGGTGGAA Num.seqs=6 Similarity=0.550000 tpcCG=40.200001 0 TACGGTGGAAATGGAGATGAATANGCCNT-NGCNGTTGCAATCGCTCAAAACGGNGATATAATAGTGACAGGCGACACTAACAGCTTCGGCGCTGGTTANGATGACGTTTGGGTTCTTAGACTTGAT-GCAAATGGTAACGTTAAGTGGCAAAAAACT

Rev.of_arqueas_representative:CP014862.1_Thermococcus_profundus__DT_5432_:134:917733-918678 Satlength=946 Nr of Repeats=5 RepeatLength=156 seed=CCGTAAGTCT Num.seqs=4 Similarity=0.618827 tpcCG=53.12500 5 TACGGAGGGAGAGGTGAGGGATGTGGCTNACGCGGTTGCCATAGCTCCAAATGGGGACATTATTGTTGCTGGGTGCACTTACAGTTTCGGCGCTGGTAATGATGATGTTTGGGTTCTCAGGCTTGATNGTGAGTGGAAATATCAAGTGGCAGAAGACT

***** ** * ** ** * ** * * ** ***** ** **** *** ** ** ** ** ** * ** **** **** ************ * ***** *********** ** ****** * * *** ** * ******** ** ***

Consensus:

TACGGaGGaAaaGGaGAgGaATatGcCttacGCgGTTGCaATaGCTCaAAAcGGgGAcATaATaGTgaCaGGcgaCACTaACAGcTTCGGCGCTGGTaAtGATGAcGTTTGGGTTCTcAGaCTTGATnGcaAaTGGaAAcaTcAAGTGGCAaAAaACT

>arqueas_representative_Fam_232_145_2 Nr. of seq. 2 Alignment length(with gaps) = 145 Alignment score = 0.786207

arqueas_representative:NC_003552.1_Methanosarcina_acetivorans_str._C2A:80:4063362-4064946 Satlength=1585 Nr of Repeats=11 RepeatLength=144 seed=AAAAAGCACT Num.seqs=11 Similarity=0.864815 tpcCG=42.7000 0 AAAAAGCACTCGAAATTTACG-AAAAACTACTCGAAAAAGACCCCGAAAATGTAGCATACCAATCATACGTAGGAANGACGCTAAACAATTTAGGAAACTTGCTTTCGGATATGGGGAGAATTGAAGACGCGAAAAACAGGTACG

arqueas_representative:NZ_CP009520.1_Methanosarcina_vacuolata_Z-761:88:2949293-2950001 Satlength=709 Nr of Repeats=5 RepeatLength=144 seed=TTGAAGAGGC Num.seqs=4 Similarity=0.764751 tpcCG=39.732498 121 AAAAAGCACTCAAAATCGAGGCAAAAACTACTCAAAAACGACCCCGAAAACGTATCTTACCAATCTTACGTAGGAATGACACTAAACAATTTAGGAAACTTGCTTAAAAATATGGGGCTCATTGAAGAGGCGAAACAAAGGTACG

*********** **** * * *********** **** *********** *** * ******** ********** *** ************************ ******** ******** ****** * *******

Consensus:

AAAAAGCACTCaAAATcgAcGcAAAAACTACTCaAAAAaGACCCCGAAAAcGTAgCaTACCAATCaTACGTAGGAAtGACaCTAAACAATTTAGGAAACTTGCTTaaaaATATGGGGagaATTGAAGAcGCGAAAaAaAGGTACG

>arqueas_representative_Fam_233_141_2 Nr. of seq. 2 Alignment length(with gaps) = 141 Alignment score = 0.806147

arqueas_representative:NC_009464.1_Methanocella_arvoryzae_MRE50_:57:185530-186235 Satlength=706 Nr of Repeats=5 RepeatLength=141 seed=TATATGTTGC Num.seqs=5 Similarity=0.726620 tpcCG=54.599998 0 TATATGTTGCCAGCGCCATCAACCGCAATACCNNANGGTCGATCGAATTGTCCTGGCTCGNTGCCATTGCTTCCCATTGTTGTCCAGGTGTTTGTGGTTTTGTTCCATACCTGAATCCTGTNATTAAAGGTGTCGGCTACG

arqueas_representative:NC_009464.1_Methanocella_arvoryzae_MRE50_:57:187977-188682 Satlength=706 Nr of Repeats=5 RepeatLength=141 seed=TACGTATATG Num.seqs=5 Similarity=0.798592 tpcCG=54.599998 137 TATATGTTGCCCGTATCATCAACTGCAATACCAGACGNNTGNTCGAATTGTCCTGGTTCGTCGCCATGGCTGCCCATTGTTGTCCAGGTGTTTGTGGCTTTGTTCCATACCTGAATCCTNTGATTAANTGTGTCGGTTACG

*********** * ******* ******** * * * ************** *** ***** *** ************************* ********************* * ***** ******* ****

Consensus:

TATATGTTGCCaGcacCATCAACcGCAATACCagAcGgtcGaTCGAATTGTCCTGGcTCGtcGCCATgGCTgCCCATTGTTGTCCAGGTGTTTGTGGcTTTGTTCCATACCTGAATCCTgTgATTAAagGTGTCGGcTACG

>arqueas_representative_Fam_234_131_2 Nr. of seq. 2 Alignment length(with gaps) = 131 Alignment score = 0.753181

arqueas_representative:NC_003552.1_Methanosarcina_acetivorans_str._C2A:80:1140558-1141206 Satlength=649 Nr of Repeats=5 RepeatLength=129 seed=CCATTTTGCC Num.seqs=4 Similarity=0.519199 tpcCG=42.700001 0 CCATTTTGCCACAGGAAAGCATGTGATTCACCAGTATCCGTCTTGACTGACACCCACTACCTGCCCATTGTCATTGATTCCACTGGCACAGCTAT-TNCTACATCCGAGCGTTCCAAGATCAGTCATCACA

arqueas_representative:NZ_CP009506.1_Methanosarcina_siciliae_T4/M:86:1017185-1017836 Satlength=652 Nr of Repeats=5 RepeatLength=129 seed=CCATTTTGCC Num.seqs=3 Similarity=0.492181 tpcCG=42.900002 0 CCATTTTGCCACAGGAAAGCANGTGANNCANCANTATCCGTCTTGACTGANGCCTACTACCTGCCCATTNTCATTGATTCCNCTGGCATNGCTATANGCT-CCNNCTNNTGTTCCAATATCAGTCATCGTG

********************* **** ** ** **************** ** ************** *********** ****** ***** ** * * ******* **********

Consensus:

CCATTTTGCCACAGGAAAGCAtGTGAttCAcCAgTATCCGTCTTGACTGAcaCCcACTACCTGCCCATTgTCATTGATTCCaCTGGCAcaGCTATatgCTaCatcCgagcGTTCCAAgATCAGTCATCaca

>arqueas_representative_Fam_235_126_2 Nr. of seq. 2 Alignment length(with gaps) = 126 Alignment score = 0.788360

arqueas_representative:NC_017527.1_Methanosaeta_harundinacea_6Ac:78:643977-645111 Satlength=1135 Nr of Repeats=9 RepeatLength=126 seed=ACCTTGAGGG Num.seqs=9 Similarity=0.766608 tpcCG=60.569199 0 ACCTTGAGGGTCTGATCACCGGAAGCAGATACAGCTTTTCGACCGTCNGGGGTCACNGCCACCGCNCTGACCGAAGCTGAATGACCTTTCAGGGTCCGTATCTCCTCGCCCCTTTCCAGGTCCCAN

arqueas_representative:NC_017527.1_Methanosaeta_harundinacea_6Ac:78:2246493-2248005 Satlength=1513 Nr of Repeats=11 RepeatLength=126 seed=TCCCATACCT Num.seqs=10 Similarity=0.776028 tpcCG=60.569199 120 ACCTTGAGGGTCTGATCACCGGATGCCGATACCGCTCTCAGACCGTCCGGGGTCACCGCCACCGCGCTGACCCAATTGGAATGGCCTTTCAGCGTCCTCAGCTCTTCGCCCCTCTCCAGGTCCCAT

*********************** ** ***** *** * ******* ******** ******** ****** ** ***** ******** **** * *** ******** ***********

Consensus:

ACCTTGAGGGTCTGATCACCGGAaGCaGATACaGCTcTcaGACCGTCcGGGGTCACcGCCACCGCgCTGACCcAAgcgGAATGaCCTTTCAGcGTCCgcAgCTCcTCGCCCCTcTCCAGGTCCCAt

>arqueas_representative_Fam_236_126_2 Nr. of seq. 2 Alignment length(with gaps) = 126 Alignment score = 0.817460

arqueas_representative:NC_003552.1_Methanosarcina_acetivorans_str._C2A:80:222398-223406 Satlength=1009 Nr of Repeats=9 RepeatLength=126 seed=AGTGCTTTTT Num.seqs=7 Similarity=0.662383 tpcCG=42.700001 0 AGTGCTTTTTCGTAATCTCCCATACTTTCATAGAGTCCTGCGAGATTATTTAGGGTTGTTGCAACATCTGGATGTTGTGGNCCCAGAACCTTTTCATNTATTTCAAGTGNTCGTTGAGAAAGTNNG

arqueas_representative:NZ_CP009520.1_Methanosarcina_vacuolata_Z-761:88:701971-703274 Satlength=1304 Nr of Repeats=9 RepeatLength=126 seed=TTTGATAAAG Num.seqs=7 Similarity=0.912636 tpcCG=39.732498 112 AGTGCTTTTTCGTATTCTCCCATACTTTCATAGAGTCCTGCGAGATTGTTTAGTGTTGTTGCAACATCTGGGTGTTGCGGCCCGAGCACCTTTTCACTNATNTCCAGTGCCCTTTGATAAAGTGGG

************** ******************************** ***** ***************** ***** ** ** ** ********* ** ** **** * **** ***** *

Consensus:

AGTGCTTTTTCGTAaTCTCCCATACTTTCATAGAGTCCTGCGAGATTaTTTAGgGTTGTTGCAACATCTGGaTGTTGcGGcCCcAGaACCTTTTCActtATtTCaAGTGccCgTTGAgAAAGTggG

>arqueas_representative_Fam_237_123_2 Nr. of seq. 2 Alignment length(with gaps) = 123 Alignment score = 0.691057

arqueas_representative:NZ_CP009506.1_Methanosarcina_siciliae_T4/M:86:2498514-2499234 Satlength=721 Nr of Repeats=6 RepeatLength=120 seed=TCAATTGCTT Num.seqs=6 Similarity=0.418462 tpcCG=42.900002 0 TCAATTGCTTTTCTNAGGTCNCCTAGATGAATATATGCTTNNCCCAAATTTCCAAGATNATTTCCTTCTC--CATACTTATC-TTCNATTTCTCTNGAAATTTTCAACGCCTGTTCATAAAAT

Rev.of_arqueas_representative:NZ_CP009520.1_Methanosarcina_vacuolata_Z-761:88:1809358-1810327 Satlength=970 Nr of Repeats=6 RepeatLength=120 seed=AGCAATTGAA Num.seqs=4 Similarity=0.635185 tpcCG=39.732 9 TCAATTGCTTTTTTCGTCTCGCCTAGGTGACTATATTCTAACCCCAAATCTCCAAGATGATTTCCTTCTCTGCGT--TTATCATTC-ATTTTTCTTGAAATTTTCAATGCCTGCTTGTAATAT

************ * ** ***** *** ***** ** ******* ******** *********** * * ***** *** **** *** *********** ***** * *** **

Consensus:

TCAATTGCTTTTcTcagcTCgCCTAGaTGAaTATATgCTaacCCCAAATcTCCAAGATgATTTCCTTCTCtgCaTacTTATCaTTCnATTTcTCTtGAAATTTTCAAcGCCTGcTcaTAAaAT

>arqueas_representative_Fam_238_110_2 Nr. of seq. 2 Alignment length(with gaps) = 110 Alignment score = 0.721212

arqueas_representative:NZ_CP009506.1_Methanosarcina_siciliae_T4/M:86:1980464-1982588 Satlength=2125 Nr of Repeats=14 RepeatLength=108 seed=TTCTTCTTTT Num.seqs=10 Similarity=0.593502 tpcCG=42.900002 0 TTCTTCTTTTCTTTGCCCATCCAAATAAGGAACAATAGCTGAAAGTGCCTATACTCT-TGNATAATCATCTTCAATCTTGGAGGCTG-CATCAAGGGCTTTTTCCATCAC

Rev.of_arqueas_representative:NZ_CP009520.1_Methanosarcina_vacuolata_Z-761:88:3931052-3933011 Satlength=1960 Nr of Repeats=16 RepeatLength=108 seed=AAGCCCTTGA Num.seqs=14 Similarity=0.867695 tpcCG=39. 98 TTCTTCTTTTCTTTGGCCATCCAAATGAGGGACAAGAGCAGAAAGGGCATTTGATCTGTG-ATAGTCATCTTGAATCCTTGAGG-TGATTTCAAGGGCTTTTTCTATCAC

*************** ********** *** **** *** ***** ** * * *** ** *** ******* **** * **** ** ************** *****

Consensus:

TTCTTCTTTTCTTTGcCCATCCAAATaAGGaACAAgAGCaGAAAGgGCaTaTaaTCTgTGnATAaTCATCTTcAATCcTgGAGGcTGacaTCAAGGGCTTTTTCcATCAC

>arqueas_representative_Fam_239_104_2 Nr. of seq. 2 Alignment length(with gaps) = 104 Alignment score = 0.637821

arqueas_representative:NC_017527.1_Methanosaeta_harundinacea_6Ac:78:933020-933938 Satlength=919 Nr of Repeats=5 RepeatLength=102 seed=AGGGCGATCC Num.seqs=3 Similarity=0.595469 tpcCG=60.569199 0 AGGGCGATCCCCCGGTTGTACCAGGCCNGGGCGTAGCNCGGATCGANCTTCAGGGCCTCNTCGTAGCAGTCGACCGCCTCCTCNTGCCGTCCGAGAAGTC--CG

arqueas_representative:NC_017527.1_Methanosaeta_harundinacea_6Ac:78:2187789-2189013 Satlength=1225 Nr of Repeats=8 RepeatLength=102 seed=CCCCGGTTGT Num.seqs=5 Similarity=0.479880 tpcCG=60.569199 9 AGGGNCNGCCCCCGGTTGTNCCAGGCGANGGCGAAGGCCGGGTCGATCTCCAGGGCCCGGTCGTANGACTCGANCGCCTCCTCGTACCTGCCGAG--GGCGNCG

**** *********** ****** **** ** *** **** ** ******* ***** * **** ********* * ** ***** * * **

Consensus:

AGGGccagCCCCCGGTTGTaCCAGGCcagGGCGaAGccCGGaTCGAtCTcCAGGGCCccgTCGTAgcAcTCGAcCGCCTCCTCgTaCCggCCGAGaaGgCgnCG

>arqueas_representative_Fam_240_103_2 Nr. of seq. 2 Alignment length(with gaps) = 103 Alignment score = 0.621359

arqueas_representative:NC_015574.1_Methanobacterium_paludis__SWAN1:47:787677-788391 Satlength=715 Nr of Repeats=6 RepeatLength=102 seed=GTTATGATAA Num.seqs=5 Similarity=0.558255 tpcCG=35.700001 0 GTTATGATAAGGCTTTAGAGATNGATCCNAANTAT-TTTNATGCATGGTTTAATAAAGGATATGCNTTAGCNGAGCTTGGAAAATATTTAGAAGCATTAGAAN

Rev.of_arqueas_representative:NC_009637.1_Methanococcus_maripaludis_C7:62:132141-133362 Satlength=1222 Nr of Repeats=10 RepeatLength=102 seed=TTCTTCGTAT Num.seqs=6 Similarity=0.577362 tpcCG=33.299999 92 GTTTTGATAAGGCTTTAGAAATAGATTCTAATAATGAGTAATTC-TGGAATAACAAAGGATATTCTTTTTCAGAACTTGAAAGATACGAAGAAGCCATAGAAT

*** *************** ** *** * ** ** * ** * *** *** ********* * ** * ** **** ** *** ****** *****

Consensus:

GTTaTGATAAGGCTTTAGAaATaGATcCtAAtaATgagTaATgCaTGGaaTAAcAAAGGATATgCtTTagCaGAaCTTGaAAaATAcgaAGAAGCaaTAGAAt

>arqueas_representative_Fam_241_102_2 Nr. of seq. 2 Alignment length(with gaps) = 102 Alignment score = 0.686275

arqueas_representative:NZ_CP009528.1_Methanosarcina_barkeri_MS:82:2959755-2960571 Satlength=817 Nr of Repeats=7 RepeatLength=102 seed=ATAACAAAGG Num.seqs=6 Similarity=0.781230 tpcCG=39.168999 0 ATAACAAAGGTGATGCCCTTTCTAATTTAGGTAAATATAATGAGTCAATCCAAGCTTATGATAAAGCCATAGAGATAAACCCAAACTATTCCTTTGCCTGGA

arqueas_representative:NZ_CP009520.1_Methanosarcina_vacuolata_Z-761:88:2119203-2119917 Satlength=715 Nr of Repeats=6 RepeatLength=102 seed=ATGAAGAGGC Num.seqs=5 Similarity=0.674510 tpcCG=39.732498 36 ATAANAAAGGTATTGCCCTTAATAANTTGGGCAGATATGAAGAGGCAATAATAGCTTATGANAAAGCCCTAGAGATAGACCCGAAATATGTCNATGCATGGA

**** ****** ******* *** ** ** * **** * *** **** ********* ****** ******** **** ** *** * *** ****

Consensus:

ATAAcAAAGGTaaTGCCCTTaaTAAtTTaGGcAaATATaAaGAGgCAATaaaAGCTTATGAtAAAGCCaTAGAGATAaACCCaAAaTATgcCtaTGCaTGGA

>arqueas_representative_Fam_242_79_2 Nr. of seq. 2 Alignment length(with gaps) = 79 Alignment score = 0.738397

arqueas_representative:NC_015676.1_Methanosalsum_zhilinae_DSM_4017:79:141852-142326 Satlength=475 Nr of Repeats=6 RepeatLength=78 seed=GTTGCATATG Num.seqs=5 Similarity=0.601709 tpcCG=39.200001 0 GTTGCATATGAATTGTTNATTG-TGCCTCNATTCNANCCAACCAGTCCACCGACANAACNANANCCNTCAACATTTCCA

Rev.of_arqueas_representative:NC_015676.1_Methanosalsum_zhilinae_DSM_4017:79:1448480-1449116 Satlength=637 Nr of Repeats=8 RepeatLength=78 seed=TCATATGCAA Num.seqs=5 Similarity=0.580159 tpcCG=39.20000 11 GTTGCATATGAATT-TTCAATGTTTCCTCGATTATNTCCAACCAGTCCACCGACATACCAANTACCNTCAACATTTCCA

************** ** * ** * **** *** ****************** * * ** ***************

Consensus:

GTTGCATATGAATTgTTcAaTGtTgCCTCgATTatatCCAACCAGTCCACCGACAtAaCaANaaCCNTCAACATTTCCA

>arqueas_representative_Fam_243_57_2 Nr. of seq. 2 Alignment length(with gaps) = 57 Alignment score = 0.827485

arqueas_representative:NC_013922.1_Natrialba_magadii_ATCC_43099:100:1527872-1528096 Satlength=225 Nr of Repeats=4 RepeatLength=56 seed=CGCGGTTCTC Num.seqs=4 Similarity=1.000000 tpcCG=61.032001 0 CGCGGTTCTCACTCACTTCGTTCGCTCGCGGGTCACACT-GTTCCCCGCTCGTCGTC

Rev.of_arqueas_representative:NC_019974.1_Natronococcus_occultus_SP4:103:1785910-1786170 Satlength=261 Nr of Repeats=4 RepeatLength=56 seed=GAGCGAACGA Num.seqs=3 Similarity=1.000000 tpcCG=64.629402 26 -GCGGTTCTCACTCACTTCGTTCGCTCGCGGGTCACGTTCGTTCCCCGCTCGCAGCC

*********************************** * ************ * *

Consensus:

cGCGGTTCTCACTCACTTCGTTCGCTCGCGGGTCACacTcGTTCCCCGCTCGcaGcC

>arqueas_representative_Fam_244_54_2 Nr. of seq. 2 Alignment length(with gaps) = 54 Alignment score = 0.632716

arqueas_representative:NZ_LT607756.1_Methanobacterium_congolense_isolate_Buetzberg_:44:2105112-2105304 Satlength=193 Nr of Repeats=4 RepeatLength=48 seed=AAAAAGAAAT Num.seqs=4 Similarity=0.916667 tpcC 0 -AAAAAGAAATACAAGAACAAATAAAAGGAA----AAATCCA-AACCGAAATTC

arqueas_representative:NC_015574.1_Methanobacterium_paludis__SWAN1:47:2191053-2191257 Satlength=205 Nr of Repeats=4 RepeatLength=51 seed=GAAAAAGAAA Num.seqs=4 Similarity=0.768162 tpcCG=35.700001 0 GAAAAAGAAATACAAGAACAAATAAGAGGAATGTTAAA--CAGAGGCG-AACTC

************************ ***** *** ** * ** ** **

Consensus:

gAAAAAGAAATACAAGAACAAATAAaAGGAAtgttAAAtcCAgAacCGaAAcTC

>arqueas_representative_Fam_245_45_2 Nr. of seq. 2 Alignment length(with gaps) = 45 Alignment score = 0.614815

arqueas_representative:NC_013743.1_Haloterrigena_turkmenica_DSM_5511:38:1015031-1015952 Satlength=922 Nr of Repeats=6 RepeatLength=42 seed=TCTTCGTCCT Num.seqs=4 Similarity=1.000000 tpcCG=64.248398 0 TCTTCGTCCT--CTGC-TTCCTCCTCCTCAGCCTCTTCTTCAGTC

Rev.of_arqueas_representative:NZ_CP016070.1_Halodesulfurarchaeum_formicicum__HTSR1:24:843107-843302 Satlength=196 Nr of Repeats=5 RepeatLength=45 seed=GATGAGGACG Num.seqs=4 Similarity=0.960494 tpcCG=6 41 TCGTCGTCCTCGCTGCTTTCCTCGTCTTCAGCGTCCTCATCGCTG

** ******* **** ****** ** ***** ** ** ** *

Consensus:

TCgTCGTCCTcgCTGCtTTCCTCcTCcTCAGCcTCcTCaTCacTc

>arqueas_representative_Fam_246_39_2 Nr. of seq. 2 Alignment length(with gaps) = 39 Alignment score = 0.722222

arqueas_representative:NZ_CP009512.1_Methanosarcina_mazei_S-6:85:969202-969458 Satlength=257 Nr of Repeats=8 RepeatLength=32 seed=CCCGAATTGC Num.seqs=8 Similarity=0.955357 tpcCG=41.400002 0 CCCGAATTGCGGCTCGGGAGTACGCTGTCCTT-------

arqueas_representative:NZ_CP009515.1_Methanosarcina_lacustris_Z-7289:84:3774605-3774738 Satlength=134 Nr of Repeats=4 RepeatLength=39 seed=CCCGAATTGC Num.seqs=3 Similarity=1.000000 tpcCG=41.799999 0 CCCGAATTGCGCCTCGGGAGTACGCGGTCCTTTTCGCTG

*********** ************* ******

Consensus:

CCCGAATTGCGcCTCGGGAGTACGCgGTCCTTttcgctg

>arqueas_representative_Fam_247_36_2 Nr. of seq. 2 Alignment length(with gaps) = 36 Alignment score = 1.000000

arqueas_representative:NZ_CP009528.1_Methanosarcina_barkeri_MS:82:1075825-1076113 Satlength=289 Nr of Repeats=8 RepeatLength=36 seed=AGCGGAAGTT Num.seqs=8 Similarity=0.923280 tpcCG=39.168999 0 AGCGGAAGTTCTGATACAGGAGATTCAGTCTCAGAT

arqueas_representative:NZ_CP009528.1_Methanosarcina_barkeri_MS:82:1075819-1076686 Satlength=868 Nr of Repeats=12 RepeatLength=36 seed=TCAGATAGCG Num.seqs=9 Similarity=0.862140 tpcCG=39.168999 30 AGCGGAAGTTCTGATACAGGAGATTCAGTCTCAGAT

************************************

Consensus:

AGCGGAAGTTCTGATACAGGAGATTCAGTCTCAGAT

>arqueas_representative_Fam_248_35_2 Nr. of seq. 2 Alignment length(with gaps) = 35 Alignment score = 0.809524

arqueas_representative:NC_009464.1_Methanocella_arvoryzae_MRE50_:57:108822-108997 Satlength=176 Nr of Repeats=5 RepeatLength=35 seed=TTCACCACAG Num.seqs=5 Similarity=0.984762 tpcCG=54.599998 0 TTCACCACAGAGGCACAGAGGGACACAGAGAACGG

arqueas_representative:NC_009464.1_Methanocella_arvoryzae_MRE50_:57:2409198-2409330 Satlength=133 Nr of Repeats=4 RepeatLength=35 seed=AGAGGCACAG Num.seqs=3 Similarity=1.000000 tpcCG=54.599998 8 GTAACCACAGAGGCACAGAGGTGCACAGAGAACTG

* ****************** ********** *

Consensus:

gTaACCACAGAGGCACAGAGGgaCACAGAGAACgG

>arqueas_representative_Fam_249_33_2 Nr. of seq. 2 Alignment length(with gaps) = 33 Alignment score = 0.676768

arqueas_representative:NC_015954.1_Halophilic_archaeon_DL31:29:1370904-1371594 Satlength=691 Nr of Repeats=4 RepeatLength=33 seed=TCCAACGCGC Num.seqs=3 Similarity=0.838384 tpcCG=62.373299 0 TCCAACGCGCCTGTTCGGGCCGCGTCGCCGTCG

Rev.of_arqueas_representative:NC_015954.1_Halophilic_archaeon_DL31:29:1495867-1495999 Satlength=133 Nr of Repeats=4 RepeatLength=33 seed=GACGGCGCGG Num.seqs=4 Similarity=1.000000 tpcCG=62.373299 29 CCCGGCGCGCCTGCTCGGTCCGCGCCGTCTTCG

** ******** **** ***** ** * ***

Consensus:

cCCaaCGCGCCTGcTCGGgCCGCGcCGcCgTCG

>arqueas_representative_Fam_250_33_2 Nr. of seq. 2 Alignment length(with gaps) = 33 Alignment score = 0.969697

arqueas_representative:NZ_CP007055.1_Halostagnicola_larsenii_XH-48:36:893830-894061 Satlength=232 Nr of Repeats=5 RepeatLength=33 seed=CCCCAGTCGG Num.seqs=3 Similarity=0.690236 tpcCG=60.865299 0 CCCCAGTCGGTGACNGTATCGACGGTGTCGCCG

arqueas_representative:NZ_CP007055.1_Halostagnicola_larsenii_XH-48:36:916113-916344 Satlength=232 Nr of Repeats=5 RepeatLength=33 seed=CCCCAGTCGG Num.seqs=3 Similarity=0.690236 tpcCG=60.865299 0 CCCCAGTCGGTGACNGTATCGACGGTGTCGCCG

*********************************

Consensus:

CCCCAGTCGGTGACNGTATCGACGGTGTCGCCG

>arqueas_representative_Fam_251_33_2 Nr. of seq. 2 Alignment length(with gaps) = 33 Alignment score = 0.686869

arqueas_representative:NZ_CP009528.1_Methanosarcina_barkeri_MS:82:3771148-3771279 Satlength=132 Nr of Repeats=4 RepeatLength=33 seed=AGAGTATATG Num.seqs=3 Similarity=0.811448 tpcCG=39.168999 0 AGAGTATATGCCTAAATAGGTGAGGAAATAAAG

Rev.of_arqueas_representative:NZ_CP009520.1_Methanosarcina_vacuolata_Z-761:88:3529900-3530057 Satlength=158 Nr of Repeats=4 RepeatLength=33 seed=TTTCTTTATT Num.seqs=3 Similarity=0.771044 tpcCG=39.7324 3 AAAGTGTATTTCTAAATATNCGGGGAAATAAAG

* *** *** ******* * **********

Consensus:

AaAGTaTATgcCTAAATAggcGaGGAAATAAAG

>arqueas_representative_Fam_252_33_2 Nr. of seq. 2 Alignment length(with gaps) = 33 Alignment score = 0.737374

arqueas_representative:NC_011832.1_Methanosphaerula_palustris_E1-9c:90:1486529-1486826 Satlength=298 Nr of Repeats=8 RepeatLength=33 seed=GTCGAAGGTC Num.seqs=5 Similarity=0.692929 tpcCG=55.400002 0 GTCGAAGGTCGCCCCATTAAAACTGGCATNTCC

arqueas_representative:NC_011832.1_Methanosphaerula_palustris_E1-9c:90:1486679-1487075 Satlength=397 Nr of Repeats=9 RepeatLength=33 seed=AAAACTGGCA Num.seqs=6 Similarity=0.640000 tpcCG=55.400002 18 GTTGAAGTTCGCCCCTTCAAAACCGGCANATCC

** **** ******* * ***** **** ***

Consensus:

GTcGAAGgTCGCCCCaTcAAAACcGGCAtaTCC

>arqueas_representative_Fam_253_31_2 Nr. of seq. 2 Alignment length(with gaps) = 31 Alignment score = 0.612903

arqueas_representative:NC_021169.1_Archaeoglobus_sulfaticallidus_PM70-1:6:178676-178790 Satlength=115 Nr of Repeats=4 RepeatLength=30 seed=GTTGGGGTTG Num.seqs=3 Similarity=0.749104 tpcCG=43.200001 0 GTTGGAGTTGGGGTTGGTGTTTCT-TCTGGA

arqueas_representative:NC_007796.1_Methanospirillum_hungatei_JF-1:91:501373-501703 Satlength=331 Nr of Repeats=11 RepeatLength=30 seed=GTTAGAGTTG Num.seqs=11 Similarity=0.946667 tpcCG=45.099998 0 GTTAGAGTTGGGGTGGGTGTAGGTGTC-GGT

*** ********** ***** * ** **

Consensus:

GTTaGAGTTGGGGTgGGTGTagcTgTCtGGa

>arqueas_representative_Fam_254_31_2 Nr. of seq. 2 Alignment length(with gaps) = 31 Alignment score = 0.913979

arqueas_representative:NC_009051.1_Methanoculleus_marisnigri_JR1:68:546554-546929 Satlength=376 Nr of Repeats=6 RepeatLength=31 seed=CACCTGACGG Num.seqs=4 Similarity=1.000000 tpcCG=62.099998 0 CACCTGACGGCGCTCACGCTCCGGTTCTTCG

arqueas_representative:NC_009051.1_Methanoculleus_marisnigri_JR1:68:1837518-1837673 Satlength=156 Nr of Repeats=5 RepeatLength=31 seed=GCTCCGGTTC Num.seqs=5 Similarity=1.000000 tpcCG=62.099998 17 CACCTGAAGGTGCTCACGCTCCGGTTCTTCG

******* ** ********************

Consensus:

CACCTGAaGGcGCTCACGCTCCGGTTCTTCG

>arqueas_representative_Fam_255_29_2 Nr. of seq. 2 Alignment length(with gaps) = 29 Alignment score = 0.637931

arqueas_representative:NZ_CP009517.1_Methanosarcina_barkeri_3:81:4553537-4553745 Satlength=209 Nr of Repeats=8 RepeatLength=26 seed=TTTCTTCTCT Num.seqs=8 Similarity=1.000000 tpcCG=39.099998 0 TTTC-TTCTCTA--TTCTTTTTCTTTTTG

Rev.of_arqueas_representative:NZ_CP014265.1_Methanobrevibacter_olleyae__YLM1:49:1098279-1098392 Satlength=114 Nr of Repeats=4 RepeatLength=28 seed=AAAAAAGAAA Num.seqs=3 Similarity=1.000000 tpcCG=26.90 1 TATCTTTCTATATTTTCTATTTCTTTTT-

* ** **** ** **** *********

Consensus:

TaTCtTTCTaTAttTTCTaTTTCTTTTTg

>arqueas_representative_Fam_256_29_2 Nr. of seq. 2 Alignment length(with gaps) = 29 Alignment score = 0.770115

arqueas_representative:NZ_CP009528.1_Methanosarcina_barkeri_MS:82:3354201-3354375 Satlength=175 Nr of Repeats=5 RepeatLength=29 seed=AATAATACCG Num.seqs=4 Similarity=0.800766 tpcCG=39.168999 0 AATAATACCGTTTTTCACAAATTAAGAAG

Rev.of_arqueas_representative:NZ_CP009520.1_Methanosarcina_vacuolata_Z-761:88:3364874-3365106 Satlength=233 Nr of Repeats=8 RepeatLength=29 seed=AAAAACGGTA Num.seqs=8 Similarity=0.779967 tpcCG=39.7324 15 AATAATACCGTTTTTTGCAAATTATGGTG

*************** ******* * *

Consensus:

AATAATACCGTTTTTcaCAAATTAaGaaG

>arqueas_representative_Fam_257_28_2 Nr. of seq. 2 Alignment length(with gaps) = 28 Alignment score = 0.607143

arqueas_representative:NZ_CP009528.1_Methanosarcina_barkeri_MS:82:2815728-2816004 Satlength=277 Nr of Repeats=10 RepeatLength=24 seed=TTGCTTTCTC Num.seqs=8 Similarity=0.928571 tpcCG=39.168999 0 TTGCTTTCTCT--TCGGCTTCTTTCC--

Rev.of_arqueas_representative:NZ_CP009528.1_Methanosarcina_barkeri_MS:82:1882062-1882213 Satlength=152 Nr of Repeats=6 RepeatLength=28 seed=AGAAAAAGCA Num.seqs=4 Similarity=1.000000 tpcCG=39.168999 5 TTTCTTTCTCTACTCTGCTTCTCTGCTT

** ******** ** ****** * *

Consensus:

TTgCTTTCTCTacTCgGCTTCTcTcCtt

>arqueas_representative_Fam_258_28_2 Nr. of seq. 2 Alignment length(with gaps) = 28 Alignment score = 0.648810

arqueas_representative:NC_014729.1_Halogeometricum_borinquense_DSM_11551:27:1913934-1914042 Satlength=109 Nr of Repeats=4 RepeatLength=27 seed=GGCGAACACG Num.seqs=4 Similarity=0.950617 tpcCG=59.970901 0 GGCGAACACGACGGCCACAGCCACCAC-

arqueas_representative:NC_013158.1_Halorhabdus_utahensis_DSM_12940:33:2120866-2121087 Satlength=222 Nr of Repeats=4 RepeatLength=27 seed=GAAGACGACG Num.seqs=3 Similarity=0.934156 tpcCG=62.900002 3 GATGAAGACGACGGTGACA-CCACCACT

* *** ******* *** *******

Consensus:

GacGAAcACGACGGccACAgCCACCACt

>arqueas_representative_Fam_259_28_2 Nr. of seq. 2 Alignment length(with gaps) = 28 Alignment score = 0.642857

arqueas_representative:NZ_CP009512.1_Methanosarcina_mazei_S-6:85:652340-652544 Satlength=205 Nr of Repeats=7 RepeatLength=27 seed=TTCTTCTTTT Num.seqs=5 Similarity=0.813333 tpcCG=41.400002 0 TTCTTCTTTTTA-TTTTTATTAATTGAC

Rev.of_arqueas_representative:NC_003552.1_Methanosarcina_acetivorans_str._C2A:80:4240412-4240524 Satlength=113 Nr of Repeats=4 RepeatLength=28 seed=GTAAAAAAAT Num.seqs=4 Similarity=1.000000 tpcCG=42.7 0 TTCTTATTTTTACTCCTTATTTTTTTAC

***** ****** * ***** ** **

Consensus:

TTCTTaTTTTTAcTccTTATTaaTTgAC

>arqueas_representative_Fam_260_27_2 Nr. of seq. 2 Alignment length(with gaps) = 27 Alignment score = 0.709877

arqueas_representative:NZ_CP009520.1_Methanosarcina_vacuolata_Z-761:88:3753724-3753828 Satlength=105 Nr of Repeats=4 RepeatLength=26 seed=TTTCCGGATT Num.seqs=4 Similarity=0.897436 tpcCG=39.732498 0 TTTCCGGATTTTATTTTTAGCGTTTG-

Rev.of_arqueas_representative:NZ_CP009515.1_Methanosarcina_lacustris_Z-7289:84:1182667-1183414 Satlength=748 Nr of Repeats=5 RepeatLength=27 seed=TAAAAGCTGA Num.seqs=3 Similarity=0.967078 tpcCG=41.799 13 TTTTCAGCTTTTAATTTTAGCGTTTCT

*** * * ***** ***********

Consensus:

TTTcCaGaTTTTAaTTTTAGCGTTTct

>arqueas_representative_Fam_261_27_2 Nr. of seq. 2 Alignment length(with gaps) = 27 Alignment score = 0.802469

arqueas_representative:NC_014297.1_Halalkalicoccus_jeotgali_B3:17:2083871-2083964 Satlength=94 Nr of Repeats=4 RepeatLength=27 seed=GCCGCCCATC Num.seqs=3 Similarity=0.387205 tpcCG=62.558201 0 GCCGCCCATGCCGCCNGGGCCGCCNNN

Rev.of_arqueas_representative:NC_020388.1_Natronomonas_moolapensis_8.8.11_:104:906329-906437 Satlength=109 Nr of Repeats=5 RepeatLength=27 seed=ATGGGCGGCG Num.seqs=3 Similarity=0.586207 tpcCG=64.50000 9 GCCGCCCATGCCGCCGGGACCGCCACC

*************** ** *****

Consensus:

GCCGCCCATGCCGCCgGGaCCGCCacc

>arqueas_representative_Fam_262_26_2 Nr. of seq. 2 Alignment length(with gaps) = 26 Alignment score = 0.615385

arqueas_representative:NZ_CP009528.1_Methanosarcina_barkeri_MS:82:838291-838411 Satlength=121 Nr of Repeats=5 RepeatLength=24 seed=CTTCAACACT Num.seqs=3 Similarity=0.844444 tpcCG=39.168999 0 CTTCAA--CACTTCCTGAGTTTATCT

Rev.of_arqueas_representative:NZ_CP009528.1_Methanosarcina_barkeri_MS:82:3339213-3339339 Satlength=127 Nr of Repeats=5 RepeatLength=26 seed=GTATTGAAGA Num.seqs=3 Similarity=0.931624 tpcCG=39.168999 9 CTTCAATACGCATCTTGAGTATATAT

****** * * ** ***** *** *

Consensus:

CTTCAAtaCaCaTCcTGAGTaTATaT

>arqueas_representative_Fam_263_26_2 Nr. of seq. 2 Alignment length(with gaps) = 26 Alignment score = 0.621795

arqueas_representative:NZ_CP009517.1_Methanosarcina_barkeri_3:81:2697742-2697888 Satlength=147 Nr of Repeats=6 RepeatLength=25 seed=TTCACTTTTA Num.seqs=5 Similarity=0.871795 tpcCG=39.099998 0 TTCACTTTTATTCTTCTTTCTTTGA-

Rev.of_arqueas_representative:NZ_CP009517.1_Methanosarcina_barkeri_3:81:3423970-3424071 Satlength=102 Nr of Repeats=4 RepeatLength=25 seed=AAGATTAAAA Num.seqs=3 Similarity=1.000000 tpcCG=39.099998 15 TTCTGTTTTAATCTTC-TGCTTTAAT

*** ***** ***** * **** *

Consensus:

TTCacTTTTAaTCTTCtTgCTTTaAt

>arqueas_representative_Fam_264_26_2 Nr. of seq. 2 Alignment length(with gaps) = 26 Alignment score = 0.615385

arqueas_representative:NZ_CP009512.1_Methanosarcina_mazei_S-6:85:2379241-2379341 Satlength=101 Nr of Repeats=4 RepeatLength=25 seed=AGTAAATTAA Num.seqs=4 Similarity=0.937778 tpcCG=41.400002 0 AGTAAATTAATAAAC-TGAACTATTA

arqueas_representative:NZ_CP014265.1_Methanobrevibacter_olleyae__YLM1:49:1241284-1241388 Satlength=105 Nr of Repeats=4 RepeatLength=26 seed=TTAATAAATA Num.seqs=4 Similarity=1.000000 tpcCG=26.900000 2 AGTTAATAAATAAACATTAGCAAATA

*** *** ******* * * * * **

Consensus:

AGTaAATaAATAAACaTgAaCaAaTA

>arqueas_representative_Fam_265_26_2 Nr. of seq. 2 Alignment length(with gaps) = 26 Alignment score = 0.602564

arqueas_representative:NZ_CP014265.1_Methanobrevibacter_olleyae__YLM1:49:753353-753610 Satlength=258 Nr of Repeats=8 RepeatLength=26 seed=TTTTATTTTT Num.seqs=5 Similarity=0.642857 tpcCG=26.900000 0 TTTTAATTTTAAAANTAATGATAAAA

Rev.of_arqueas_representative:NZ_CP014265.1_Methanobrevibacter_olleyae__YLM1:49:1167541-1167720 Satlength=180 Nr of Repeats=7 RepeatLength=26 seed=ATTTATAAAT Num.seqs=6 Similarity=1.000000 tpcCG=26.90 15 TTAGAATTTATAAATTAATTAAGAAA

** ***** *** **** * ***

Consensus:

TTagAATTTaaAAAtTAATgAaaAAA

>arqueas_representative_Fam_266_25_2 Nr. of seq. 2 Alignment length(with gaps) = 25 Alignment score = 0.686667

arqueas_representative:NZ_CP014265.1_Methanobrevibacter_olleyae__YLM1:49:63329-63426 Satlength=98 Nr of Repeats=5 RepeatLength=21 seed=TATTTAATAA Num.seqs=3 Similarity=0.767677 tpcCG=26.900000 19 TTTAAAAACT-AAAAA--TAGA-TA

arqueas_representative:NZ_CP014265.1_Methanobrevibacter_olleyae__YLM1:49:1220511-1220648 Satlength=138 Nr of Repeats=5 RepeatLength=25 seed=TATTTAAAAA Num.seqs=3 Similarity=1.000000 tpcCG=26.900000 19 TTTAAAAAATAAAAAACTTAGAATA

********** * ***** ****

Consensus:

TATTTAAAAAaTaAAAAActTAGAa

>arqueas_representative_Fam_267_25_2 Nr. of seq. 2 Alignment length(with gaps) = 25 Alignment score = 0.606667

arqueas_representative:NZ_CP009515.1_Methanosarcina_lacustris_Z-7289:84:2805236-2805383 Satlength=148 Nr of Repeats=5 RepeatLength=21 seed=TATAAAACAC Num.seqs=3 Similarity=0.848485 tpcCG=41.799999 14 CA-CAAAATCTGAAATA-TA-AAA-

arqueas_representative:NZ_CP009515.1_Methanosarcina_lacustris_Z-7289:84:1727803-1727953 Satlength=151 Nr of Repeats=6 RepeatLength=25 seed=AACATGAAAT Num.seqs=6 Similarity=0.953778 tpcCG=41.799999 6 TACTAAAACATGAAATCGTGTAAAA

* **** ****** * ***

Consensus:

cAccAAAAcaTGAAATagTatAAAa

>arqueas_representative_Fam_268_25_2 Nr. of seq. 2 Alignment length(with gaps) = 25 Alignment score = 0.620000

arqueas_representative:NC_013158.1_Halorhabdus_utahensis_DSM_12940:33:1277926-1278016 Satlength=91 Nr of Repeats=4 RepeatLength=24 seed=ACACACCGAC Num.seqs=3 Similarity=0.568376 tpcCG=62.900002 0 ACACACCGACGGAAACNGACA-CGN

arqueas_representative:NC_013158.1_Halorhabdus_utahensis_DSM_12940:33:2436431-2436539 Satlength=109 Nr of Repeats=5 RepeatLength=24 seed=GAGACGCCGA Num.seqs=3 Similarity=0.962963 tpcCG=62.900002 23 AGACGCCGACGGACACTGAAACCG-

* ** ******** ** ** * **

Consensus:

AcACaCCGACGGAaACtGAaAcCGn

>arqueas_representative_Fam_269_25_2 Nr. of seq. 2 Alignment length(with gaps) = 25 Alignment score = 0.660000

arqueas_representative:NC_013790.1_Methanobrevibacter_ruminantium_M1:50:103500-103607 Satlength=108 Nr of Repeats=4 RepeatLength=24 seed=TTGATTTTGA Num.seqs=3 Similarity=1.000000 tpcCG=32.599998 0 TTGATTTTGAATTTGACTTAA-ATG

Rev.of_arqueas_representative:NZ_CP009528.1_Methanosarcina_barkeri_MS:82:397287-397503 Satlength=217 Nr of Repeats=9 RepeatLength=24 seed=TCAATTACAA Num.seqs=9 Similarity=1.000000 tpcCG=39.168999 16 TAGATTTTGTAATTGACTTAACAC-

* ******* * ********* *

Consensus:

TaGATTTTGaAaTTGACTTAAcAcg

>arqueas_representative_Fam_270_25_2 Nr. of seq. 2 Alignment length(with gaps) = 25 Alignment score = 0.633333

arqueas_representative:NZ_CP009517.1_Methanosarcina_barkeri_3:81:4017975-4018119 Satlength=145 Nr of Repeats=7 RepeatLength=24 seed=AGAGAAGATA Num.seqs=5 Similarity=1.000000 tpcCG=39.099998 0 AGAGAAGATATTAGAAAAAATACT-

arqueas_representative:NZ_CP009512.1_Methanosarcina_mazei_S-6:85:2228528-2228628 Satlength=101 Nr of Repeats=4 RepeatLength=25 seed=AATTTAGAAA Num.seqs=4 Similarity=0.946667 tpcCG=41.400002 7 AGGGAATAATTTAGAAAAATAACTT

** *** * ********* ***

Consensus:

AGaGAAgAaaTTAGAAAAAaaACTt

>arqueas_representative_Fam_271_24_2 Nr. of seq. 2 Alignment length(with gaps) = 24 Alignment score = 0.666667

arqueas_representative:NC_013202.1_Halomicrobium_mukohataei_DSM_12286:28:54443-54602 Satlength=160 Nr of Repeats=8 RepeatLength=21 seed=GTCCGTCGGC Num.seqs=7 Similarity=0.939531 tpcCG=65.506798 0 GTCCGTCGGCGT--G-TCCGGCTC

Rev.of_arqueas_representative:NZ_CP008874.1_Halanaeroarchaeum_sulfurireducens__HSR2:18:448804-448921 Satlength=118 Nr of Repeats=5 RepeatLength=24 seed=CGACGCCGAC Num.seqs=4 Similarity=1.000000 tpcCG= 14 CTCAGTCGGCGTCGGCTCCGGCTC

** ******** * ********

Consensus:

cTCaGTCGGCGTcgGcTCCGGCTC

>arqueas_representative_Fam_272_24_2 Nr. of seq. 2 Alignment length(with gaps) = 24 Alignment score = 0.694444

arqueas_representative:CP000077.1_Sulfolobus_acidocaldarius_DSM_639:116:1754278-1754572 Satlength=295 Nr of Repeats=11 RepeatLength=21 seed=CAATATCAAA Num.seqs=8 Similarity=0.891156 tpcCG=36.700001 0 CAATATCAAACATTAC--AAC-AA

arqueas_representative:NZ_CP009517.1_Methanosarcina_barkeri_3:81:543099-543190 Satlength=92 Nr of Repeats=4 RepeatLength=23 seed=AATTCAAACA Num.seqs=3 Similarity=1.000000 tpcCG=39.099998 1 CAAT-TCAAACATTACTGAACTAA

**** *********** *** **

Consensus:

CAATaTCAAACATTACtgAACtAA

>arqueas_representative_Fam_273_24_2 Nr. of seq. 2 Alignment length(with gaps) = 24 Alignment score = 0.618056

arqueas_representative:NZ_CP009520.1_Methanosarcina_vacuolata_Z-761:88:1860333-1860509 Satlength=177 Nr of Repeats=5 RepeatLength=22 seed=TTTTAAGTAA Num.seqs=3 Similarity=0.919192 tpcCG=39.732498 0 TTTTAA-GT-AATTATGTTAATCA

Rev.of_arqueas_representative:NZ_CP009520.1_Methanosarcina_vacuolata_Z-761:88:3134975-3135067 Satlength=93 Nr of Repeats=4 RepeatLength=23 seed=ACCTTAAATG Num.seqs=4 Similarity=0.884058 tpcCG=39.73249 8 -TTTAAGGTCTATTATATTAACCA

***** ** ***** **** **

Consensus:

tTTTAAgGTcaATTATaTTAAcCA

>arqueas_representative_Fam_274_24_2 Nr. of seq. 2 Alignment length(with gaps) = 24 Alignment score = 0.638889

arqueas_representative:NZ_CP009520.1_Methanosarcina_vacuolata_Z-761:88:1750527-1750619 Satlength=93 Nr of Repeats=4 RepeatLength=23 seed=AGTAATAAGA Num.seqs=4 Similarity=0.903382 tpcCG=39.732498 0 AGTAATAAGATAATAAAATA-TGC

arqueas_representative:NZ_CP009520.1_Methanosarcina_vacuolata_Z-761:88:2988211-2988331 Satlength=121 Nr of Repeats=5 RepeatLength=24 seed=TTAAGCAAAT Num.seqs=5 Similarity=0.900000 tpcCG=39.732498 9 AGAAATAAGTTAAGCAAATATTAC

** ****** *** ***** * *

Consensus:

AGaAATAAGaTAAgaAAATAtTaC

>arqueas_representative_Fam_275_24_2 Nr. of seq. 2 Alignment length(with gaps) = 24 Alignment score = 0.611111

arqueas_representative:NC_017941.2_Haloferax_mediterranei_ATCC_33500:25:945533-945628 Satlength=96 Nr of Repeats=4 RepeatLength=24 seed=TCGTACAACT Num.seqs=3 Similarity=1.000000 tpcCG=60.255100 0 TCGTACAACTACCCCGACCCCACG

Rev.of_arqueas_representative:NC_020388.1_Natronomonas_moolapensis_8.8.11_:104:1205247-1205343 Satlength=97 Nr of Repeats=4 RepeatLength=24 seed=TTCGGCGTCG Num.seqs=4 Similarity=0.972222 tpcCG=64.5000 18 GCTGACAACGACGCCGAACCCGCG

* ***** ** **** *** **

Consensus:

gCggACAACgACcCCGAaCCCaCG

>arqueas_representative_Fam_276_24_2 Nr. of seq. 2 Alignment length(with gaps) = 24 Alignment score = 1.000000

arqueas_representative:NC_012029.1_Halorubrum_lacusprofundi_ATCC_49239_chromosome_1:34:1788152-1788416 Satlength=265 Nr of Repeats=13 RepeatLength=24 seed=GAGGACGAGG Num.seqs=9 Similarity=1.000000 tpc 0 GAGGACGAGGAAGAGGACAATGAG

arqueas_representative:NC_012029.1_Halorubrum_lacusprofundi_ATCC_49239_chromosome_1:34:1788152-1788746 Satlength=595 Nr of Repeats=15 RepeatLength=24 seed=GAGGACGAGG Num.seqs=10 Similarity=0.820988 tp 0 GAGGACGAGGAAGAGGACAATGAG

************************

Consensus:

GAGGACGAGGAAGAGGACAATGAG

>arqueas_representative_Fam_277_24_2 Nr. of seq. 2 Alignment length(with gaps) = 24 Alignment score = 0.611111

arqueas_representative:NC_009637.1_Methanococcus_maripaludis_C7:62:677808-678048 Satlength=241 Nr of Repeats=10 RepeatLength=24 seed=TACGGATAGT Num.seqs=10 Similarity=1.000000 tpcCG=33.299999 0 TACGGATAGTTCATCTGAAGATAC

arqueas_representative:NC_009637.1_Methanococcus_maripaludis_C7:62:1245978-1246482 Satlength=505 Nr of Repeats=21 RepeatLength=24 seed=GGAACTGAAG Num.seqs=21 Similarity=1.000000 tpcCG=33.299999 10 TACAGATACAGGAACTGAAGACAC

*** **** * ******* **

Consensus:

TACaGATAcagcAaCTGAAGAcAC

>arqueas_representative_Fam_278_23_2 Nr. of seq. 2 Alignment length(with gaps) = 23 Alignment score = 0.623188

arqueas_representative:NZ_CP009515.1_Methanosarcina_lacustris_Z-7289:84:4002048-4002181 Satlength=134 Nr of Repeats=7 RepeatLength=19 seed=TCCAGTTTGA Num.seqs=7 Similarity=1.000000 tpcCG=41.799999 0 TCCAGTTTGAAGAGGAT----TA

arqueas_representative:NZ_CP009520.1_Methanosarcina_vacuolata_Z-761:88:1606995-1607855 Satlength=861 Nr of Repeats=5 RepeatLength=23 seed=AATTTGAAGA Num.seqs=4 Similarity=0.874396 tpcCG=39.732498 3 TACAATTTGAAGAGGATACCTTA

* ** ************ **

Consensus:

TaCAaTTTGAAGAGGATacctTA

>arqueas_representative_Fam_279_23_2 Nr. of seq. 2 Alignment length(with gaps) = 23 Alignment score = 0.637681

arqueas_representative:NC_003552.1_Methanosarcina_acetivorans_str._C2A:80:4102514-4102728 Satlength=215 Nr of Repeats=10 RepeatLength=20 seed=TAAGTTAGTA Num.seqs=9 Similarity=0.948148 tpcCG=42.700001 0 TAAGTTAGTACATTGCA-TAT--

Rev.of_arqueas_representative:NZ_CP009517.1_Methanosarcina_barkeri_3:81:2292394-2292508 Satlength=115 Nr of Repeats=5 RepeatLength=23 seed=CTAACAGAAT Num.seqs=4 Similarity=1.000000 tpcCG=39.099998 8 TCTGTTAGTACATTGAATTATAT

* ************ * ***

Consensus:

TaaGTTAGTACATTGaAtTATat

>arqueas_representative_Fam_280_23_2 Nr. of seq. 2 Alignment length(with gaps) = 23 Alignment score = 0.695652

arqueas_representative:NZ_CP009517.1_Methanosarcina_barkeri_3:81:598916-599116 Satlength=201 Nr of Repeats=7 RepeatLength=20 seed=TGAGAATGAT Num.seqs=5 Similarity=0.786667 tpcCG=39.099998 0 TGAGAATGA-TAGAAAATAAA--

arqueas_representative:NZ_CP009520.1_Methanosarcina_vacuolata_Z-761:88:1229735-1230028 Satlength=294 Nr of Repeats=12 RepeatLength=23 seed=TAGAATATAA Num.seqs=8 Similarity=0.850932 tpcCG=39.732498 10 TGAGATTGATTAGAATATAAACC

***** *** ***** *****

Consensus:

TGAGAaTGAtTAGAAaATAAAcc

>arqueas_representative_Fam_281_23_2 Nr. of seq. 2 Alignment length(with gaps) = 23 Alignment score = 0.652174

arqueas_representative:NC_015574.1_Methanobacterium_paludis__SWAN1:47:557259-557343 Satlength=85 Nr of Repeats=4 RepeatLength=21 seed=GTTTTAGATT Num.seqs=4 Similarity=0.968254 tpcCG=35.700001 0 GTTTTAGATT-TCTCTAA-TTCA

arqueas_representative:NZ_CP009517.1_Methanosarcina_barkeri_3:81:2795139-2795278 Satlength=140 Nr of Repeats=4 RepeatLength=23 seed=TAGCTTTAAG Num.seqs=3 Similarity=1.000000 tpcCG=39.099998 9 GATTTAGATTAGCTTTAAGTTCA

* ******** ** *** ****

Consensus:

GaTTTAGATTagCTcTAAgTTCA

>arqueas_representative_Fam_282_23_2 Nr. of seq. 2 Alignment length(with gaps) = 23 Alignment score = 0.601449

arqueas_representative:NC_015574.1_Methanobacterium_paludis__SWAN1:47:2362706-2362811 Satlength=106 Nr of Repeats=5 RepeatLength=21 seed=GTTCTGTTTT Num.seqs=5 Similarity=0.961905 tpcCG=35.700001 0 GTTC-TGTTTTAGAAGTTTGTG-

arqueas_representative:NZ_CP009528.1_Methanosarcina_barkeri_MS:82:3227088-3227220 Satlength=133 Nr of Repeats=6 RepeatLength=22 seed=AAGTTGTCAA Num.seqs=6 Similarity=1.000000 tpcCG=39.168999 13 ATTCGTTTTTTAGAAG-TTGTCA

*** * ********* ****

Consensus:

aTTCgTgTTTTAGAAGtTTGTca

>arqueas_representative_Fam_283_23_2 Nr. of seq. 2 Alignment length(with gaps) = 23 Alignment score = 0.608696

arqueas_representative:NZ_CP009517.1_Methanosarcina_barkeri_3:81:3337270-3337354 Satlength=85 Nr of Repeats=4 RepeatLength=21 seed=TAAAATCATC Num.seqs=4 Similarity=0.894180 tpcCG=39.099998 0 -TAAAATCATCCAAAGAACCAA-

arqueas_representative:NZ_CP009512.1_Methanosarcina_mazei_S-6:85:2882852-2882944 Satlength=93 Nr of Repeats=4 RepeatLength=23 seed=ATAAAATCAA Num.seqs=4 Similarity=0.884058 tpcCG=41.400002 0 ATAAAATCAATAACAGAAACAAC

******** * **** ***

Consensus:

aTAAAATCAacaAaAGAAaCAAc

>arqueas_representative_Fam_284_23_2 Nr. of seq. 2 Alignment length(with gaps) = 23 Alignment score = 0.601449

arqueas_representative:NZ_CP009517.1_Methanosarcina_barkeri_3:81:4466067-4466214 Satlength=148 Nr of Repeats=7 RepeatLength=21 seed=TACTCTTACC Num.seqs=7 Similarity=1.000000 tpcCG=39.099998 0 TACTCTTACCTACCTTACCT-T-

arqueas_representative:NC_003552.1_Methanosarcina_acetivorans_str._C2A:80:4443814-4443924 Satlength=111 Nr of Repeats=5 RepeatLength=22 seed=TTCTTACCTT Num.seqs=5 Similarity=0.951515 tpcCG=42.700001 2 TATTCTTACCTTAC-TACCTCTC

** ******** * ***** *

Consensus:

TAcTCTTACCTaaCtTACCTcTc

>arqueas_representative_Fam_285_23_2 Nr. of seq. 2 Alignment length(with gaps) = 23 Alignment score = 0.630435

arqueas_representative:NZ_CP009528.1_Methanosarcina_barkeri_MS:82:3076761-3076893 Satlength=133 Nr of Repeats=6 RepeatLength=21 seed=GTAAGATTAG Num.seqs=5 Similarity=1.000000 tpcCG=39.168999 0 GTAAGATTAGTATGA-TTATTG-

Rev.of_arqueas_representative:NZ_CP009528.1_Methanosarcina_barkeri_MS:82:1257751-1257844 Satlength=94 Nr of Repeats=4 RepeatLength=23 seed=TTTTAAAAAT Num.seqs=3 Similarity=0.922705 tpcCG=39.168999 15 GTAAGATTTTTAAAAGTTATTGA

******** ** * ******

Consensus:

GTAAGATTagTAaaAgTTATTGa

>arqueas_representative_Fam_286_23_2 Nr. of seq. 2 Alignment length(with gaps) = 23 Alignment score = 0.659420

arqueas_representative:NZ_CP014265.1_Methanobrevibacter_olleyae__YLM1:49:844940-845059 Satlength=120 Nr of Repeats=5 RepeatLength=22 seed=TTTAATTTTA Num.seqs=3 Similarity=0.855072 tpcCG=26.900000 0 TTTAATTTTATATTAAATCAAG-

Rev.of_arqueas_representative:NZ_CP009517.1_Methanosarcina_barkeri_3:81:1416274-1416412 Satlength=139 Nr of Repeats=6 RepeatLength=23 seed=AAGTAAAAGC Num.seqs=6 Similarity=0.930435 tpcCG=39.099998 8 TTTTACTTTATATTAGAGTAAGC

*** * ********* * ***

Consensus:

TTTaAcTTTATATTAaAgcAAGc

>arqueas_representative_Fam_287_23_2 Nr. of seq. 2 Alignment length(with gaps) = 23 Alignment score = 0.739130

arqueas_representative:NZ_CP009518.1_Methanococcoides_methylutens_MM1:60:1447325-1447435 Satlength=111 Nr of Repeats=5 RepeatLength=22 seed=CCAGATCACG Num.seqs=5 Similarity=1.000000 tpcCG=44.000000 0 CCAGATCA-CGCTTCTGGACGCG

Rev.of_arqueas_representative:NZ_CP009518.1_Methanococcoides_methylutens_MM1:60:47386-47478 Satlength=93 Nr of Repeats=4 RepeatLength=23 seed=CAGAAGCGCT Num.seqs=4 Similarity=1.000000 tpcCG=44.000000 17 CCAGATCAGCGCTTCTGCATACG

******** ******** * **

Consensus:

CCAGATCAgCGCTTCTGcAcaCG

>arqueas_representative_Fam_288_23_2 Nr. of seq. 2 Alignment length(with gaps) = 23 Alignment score = 0.797101

arqueas_representative:NC_003552.1_Methanosarcina_acetivorans_str._C2A:80:3150662-3150791 Satlength=130 Nr of Repeats=6 RepeatLength=22 seed=AAAAGAAGGA Num.seqs=5 Similarity=1.000000 tpcCG=42.700001 0 AAAAGAAGGAAAA-GAAGAGGGA

Rev.of_arqueas_representative:NZ_CP009515.1_Methanosarcina_lacustris_Z-7289:84:1383250-1383332 Satlength=83 Nr of Repeats=4 RepeatLength=23 seed=TCTTCTTTTC Num.seqs=3 Similarity=1.000000 tpcCG=41.7999 9 AAAAGAAGAAAAACGAAGAGGGG

******** **** ********

Consensus:

AAAAGAAGaAAAAcGAAGAGGGa

>arqueas_representative_Fam_289_23_2 Nr. of seq. 2 Alignment length(with gaps) = 23 Alignment score = 0.659420

arqueas_representative:NZ_CP009517.1_Methanosarcina_barkeri_3:81:965153-965241 Satlength=89 Nr of Repeats=4 RepeatLength=22 seed=CAGTAAACAA Num.seqs=4 Similarity=0.909091 tpcCG=39.099998 0 CAGTAAACAAACAGTGTTGAGA-

Rev.of_arqueas_representative:NZ_CP009520.1_Methanosarcina_vacuolata_Z-761:88:957329-957421 Satlength=93 Nr of Repeats=4 RepeatLength=23 seed=TCTGTTTGCT Num.seqs=4 Similarity=0.700483 tpcCG=39.732498 15 TATTAAGCAAACAGAGTTGAGGT

* *** ******* ******

Consensus:

cAgTAAaCAAACAGaGTTGAGat

>arqueas_representative_Fam_290_23_2 Nr. of seq. 2 Alignment length(with gaps) = 23 Alignment score = 0.681159

arqueas_representative:NZ_CP009515.1_Methanosarcina_lacustris_Z-7289:84:2664577-2664707 Satlength=131 Nr of Repeats=6 RepeatLength=22 seed=CTTACATTTT Num.seqs=4 Similarity=0.969697 tpcCG=41.799999 0 CTTAC-ATTTTCCTATTTTTTAC

arqueas_representative:NZ_CP011266.1_Methanobrevibacter_millerae__SM9:48:339896-340080 Satlength=185 Nr of Repeats=8 RepeatLength=23 seed=TACTTTACAA Num.seqs=8 Similarity=1.000000 tpcCG=31.799999 20 TTTACAATTTTTCCAATTTTTAC

**** ***** * * *******

Consensus:

cTTACaATTTTcCcAaTTTTTAC

>arqueas_representative_Fam_291_23_2 Nr. of seq. 2 Alignment length(with gaps) = 23 Alignment score = 0.739130

arqueas_representative:NZ_CP009512.1_Methanosarcina_mazei_S-6:85:3500441-3500943 Satlength=503 Nr of Repeats=6 RepeatLength=22 seed=GAAAAACAAA Num.seqs=4 Similarity=1.000000 tpcCG=41.400002 0 GAAAAACAAAAGAGAA-AGAGGT

Rev.of_arqueas_representative:NZ_CP009517.1_Methanosarcina_barkeri_3:81:22449-22564 Satlength=116 Nr of Repeats=5 RepeatLength=23 seed=TTTCTTCTGT Num.seqs=5 Similarity=0.930435 tpcCG=39.099998 20 GAAAAAGAAAACAGAAGAAAGGT

****** **** **** * ****

Consensus:

GAAAAAcAAAAcAGAAgAaAGGT

>arqueas_representative_Fam_292_23_2 Nr. of seq. 2 Alignment length(with gaps) = 23 Alignment score = 0.710145

arqueas_representative:NZ_CP014265.1_Methanobrevibacter_olleyae__YLM1:49:1137439-1137600 Satlength=162 Nr of Repeats=7 RepeatLength=23 seed=ATTAAAAAAT Num.seqs=7 Similarity=0.884058 tpcCG=26.900000 0 ATTAAAAAATAACCTAATATTAA

Rev.of_arqueas_representative:NZ_CP009528.1_Methanosarcina_barkeri_MS:82:3499026-3499142 Satlength=117 Nr of Repeats=5 RepeatLength=23 seed=AAATTAAGTA Num.seqs=4 Similarity=0.942029 tpcCG=39.168999 20 ATTAAAAAATTACTTAATTTCCA

********** ** **** * *

Consensus:

ATTAAAAAATaACcTAATaTcaA

>arqueas_representative_Fam_293_23_2 Nr. of seq. 2 Alignment length(with gaps) = 23 Alignment score = 0.652174

arqueas_representative:CP009516.1_Methanosarcina_horonobensis_HB-1:83:2835960-2836696 Satlength=737 Nr of Repeats=4 RepeatLength=23 seed=TAAGATAGAA Num.seqs=3 Similarity=1.000000 tpcCG=41.299999 0 TAAGATAGAAATAGAATCAGAAT

Rev.of_arqueas_representative:NZ_CP009520.1_Methanosarcina_vacuolata_Z-761:88:3094987-3095307 Satlength=321 Nr of Repeats=5 RepeatLength=23 seed=ATCGTAAATC Num.seqs=4 Similarity=1.000000 tpcCG=39.7324 6 TACGATAACAATAAGATCAGATT

** **** **** ****** *

Consensus:

TAaGATAaaAATAaaATCAGAaT

>arqueas_representative_Fam_294_22_2 Nr. of seq. 2 Alignment length(with gaps) = 22 Alignment score = 0.613636

arqueas_representative:NZ_CP009517.1_Methanosarcina_barkeri_3:81:710031-710126 Satlength=96 Nr of Repeats=5 RepeatLength=19 seed=CGTTAAAAAA Num.seqs=5 Similarity=0.929825 tpcCG=39.099998 0 -CGTTAAAAAAGGAAA--CAAG

arqueas_representative:NZ_CP009517.1_Methanosarcina_barkeri_3:81:1809922-1810054 Satlength=133 Nr of Repeats=6 RepeatLength=22 seed=TCATTAGAAA Num.seqs=6 Similarity=0.947475 tpcCG=39.099998 0 TCATTAGAAAAGGAAATCCAAC

* *** ********* ***

Consensus:

tCaTTAaAAAAGGAAAtcCAAc

>arqueas_representative_Fam_295_22_2 Nr. of seq. 2 Alignment length(with gaps) = 22 Alignment score = 0.606061

arqueas_representative:NZ_CP009528.1_Methanosarcina_barkeri_MS:82:10196-10367 Satlength=172 Nr of Repeats=9 RepeatLength=19 seed=GAAATTGTAA Num.seqs=9 Similarity=0.918129 tpcCG=39.168999 0 GAAATTG---TAAAATCCAAAA

arqueas_representative:NZ_CP009506.1_Methanosarcina_siciliae_T4/M:86:2535188-2535276 Satlength=89 Nr of Repeats=4 RepeatLength=22 seed=AAATAAAATC Num.seqs=4 Similarity=1.000000 tpcCG=42.900002 7 GTAATCGAAATAAAATCAAAAA

* *** * ******* ****

Consensus:

GaAATcGaaaTAAAATCaAAAA

>arqueas_representative_Fam_296_22_2 Nr. of seq. 2 Alignment length(with gaps) = 22 Alignment score = 0.696970

arqueas_representative:NZ_CP009517.1_Methanosarcina_barkeri_3:81:1230182-1230302 Satlength=121 Nr of Repeats=6 RepeatLength=20 seed=CTTTAATTGT Num.seqs=6 Similarity=1.000000 tpcCG=39.099998 14 TTGTT-AC-TCTTATTCTTTAA

arqueas_representative:NZ_CP009528.1_Methanosarcina_barkeri_MS:82:1891846-1891978 Satlength=133 Nr of Repeats=6 RepeatLength=22 seed=ATTTTTCACT Num.seqs=6 Similarity=1.000000 tpcCG=39.168999 19 TTTTTCACTTTTTATTCTTTAA

******** ** ** * *****

Consensus:

CTTTAATTgTTcACtTcTTATT

>arqueas_representative_Fam_297_22_2 Nr. of seq. 2 Alignment length(with gaps) = 22 Alignment score = 0.674242

arqueas_representative:NZ_CP009528.1_Methanosarcina_barkeri_MS:82:1250813-1250933 Satlength=121 Nr of Repeats=6 RepeatLength=20 seed=TAATATTAAT Num.seqs=6 Similarity=0.920000 tpcCG=39.168999 0 TAATA-TTAATTTACAGATAA-

arqueas_representative:NZ_CP009517.1_Methanosarcina_barkeri_3:81:2681176-2681253 Satlength=78 Nr of Repeats=4 RepeatLength=22 seed=TAATTTACAG Num.seqs=3 Similarity=0.838384 tpcCG=39.099998 7 TCATAGATAATTTACAGGTAAT

* *** ********** ***

Consensus:

TaATAgaTAATTTACAGaTAAt

>arqueas_representative_Fam_298_22_2 Nr. of seq. 2 Alignment length(with gaps) = 22 Alignment score = 0.666667

arqueas_representative:NC_012029.1_Halorubrum_lacusprofundi_ATCC_49239_chromosome_1:34:1667811-1667916 Satlength=106 Nr of Repeats=5 RepeatLength=21 seed=GTCGGGAGTG Num.seqs=5 Similarity=1.000000 tpcC 0 GTCG-GGAGTGTCTGTGCCGTT

arqueas_representative:NC_013922.1_Natrialba_magadii_ATCC_43099:100:186974-187591 Satlength=618 Nr of Repeats=10 RepeatLength=22 seed=TGTGTTCGTG Num.seqs=7 Similarity=0.875902 tpcCG=61.032001 18 TTCGTGGTGTGTCTGTGCTGTG

*** ** ********** **

Consensus:

gTCGtGGaGTGTCTGTGCcGTg

>arqueas_representative_Fam_299_22_2 Nr. of seq. 2 Alignment length(with gaps) = 22 Alignment score = 0.666667

arqueas_representative:NC_013790.1_Methanobrevibacter_ruminantium_M1:50:1260611-1261232 Satlength=622 Nr of Repeats=7 RepeatLength=21 seed=TCTTTTGCTT Num.seqs=5 Similarity=0.860317 tpcCG=32.599998 0 TCTTTTGCTTGTA-TTTCTTCC

arqueas_representative:NZ_CP009512.1_Methanosarcina_mazei_S-6:85:1181051-1181151 Satlength=101 Nr of Repeats=5 RepeatLength=22 seed=CTTCTATTTT Num.seqs=4 Similarity=0.969697 tpcCG=41.400002 7 ACTTTTCCTTCTATTTTTTTCC

***** *** ** *** ****

Consensus:

aCTTTTcCTTcTAtTTTcTTCC

>arqueas_representative_Fam_300_22_2 Nr. of seq. 2 Alignment length(with gaps) = 22 Alignment score = 0.666667

arqueas_representative:NC_003552.1_Methanosarcina_acetivorans_str._C2A:80:24671-24965 Satlength=295 Nr of Repeats=14 RepeatLength=21 seed=GGAATATTGA Num.seqs=14 Similarity=0.860457 tpcCG=42.700001 0 GGAATATTGATAGAGTA-TAAA

Rev.of_arqueas_representative:NZ_CP009520.1_Methanosarcina_vacuolata_Z-761:88:1526229-1526391 Satlength=163 Nr of Repeats=5 RepeatLength=22 seed=TCTTTCAATA Num.seqs=4 Similarity=1.000000 tpcCG=39.7324 14 AGAATATTGAAAGAGTATTTGA

********* ****** * *

Consensus:

aGAATATTGAaAGAGTAtTaaA

>arqueas_representative_Fam_301_22_2 Nr. of seq. 2 Alignment length(with gaps) = 22 Alignment score = 0.613636

arqueas_representative:NZ_CP009517.1_Methanosarcina_barkeri_3:81:1856092-1856193 Satlength=102 Nr of Repeats=5 RepeatLength=21 seed=TAAGTCTCAT Num.seqs=3 Similarity=0.957672 tpcCG=39.099998 0 TAAGTCTCATGTTAAATCTTA-

arqueas_representative:NZ_CP009520.1_Methanosarcina_vacuolata_Z-761:88:3471919-3472058 Satlength=140 Nr of Repeats=5 RepeatLength=21 seed=GTCTCATTTT Num.seqs=4 Similarity=0.968254 tpcCG=39.732498 3 TATGTCTCAT-TTTCATTTTAA

** ******* ** ** ***

Consensus:

TAaGTCTCATgTTaaATcTTAa

>arqueas_representative_Fam_302_22_2 Nr. of seq. 2 Alignment length(with gaps) = 22 Alignment score = 0.643939

arqueas_representative:NZ_CP009528.1_Methanosarcina_barkeri_MS:82:1214654-1214780 Satlength=127 Nr of Repeats=6 RepeatLength=21 seed=AATTCCAAAA Num.seqs=6 Similarity=0.784849 tpcCG=39.168999 0 AATTCCAAAAGATCTTAAATG-

arqueas_representative:NZ_CP009520.1_Methanosarcina_vacuolata_Z-761:88:2769065-2769218 Satlength=154 Nr of Repeats=5 RepeatLength=22 seed=AAAAAGATGT Num.seqs=4 Similarity=0.697917 tpcCG=39.732498 5 AATTCAAAAAGATGTGAGAAGA

***** ******* * * * *

Consensus:

AATTCaAAAAGATcTgAaAaGa

>arqueas_representative_Fam_303_22_2 Nr. of seq. 2 Alignment length(with gaps) = 22 Alignment score = 0.606061

arqueas_representative:NZ_CP009515.1_Methanosarcina_lacustris_Z-7289:84:1890154-1890238 Satlength=85 Nr of Repeats=4 RepeatLength=21 seed=TTTTTCCAAA Num.seqs=4 Similarity=0.915344 tpcCG=41.799999 0 TTTTTCCA-AAGATTACTTCTT

Rev.of_arqueas_representative:NZ_CP009517.1_Methanosarcina_barkeri_3:81:3996216-3996304 Satlength=89 Nr of Repeats=4 RepeatLength=22 seed=TTCTGGAAGG Num.seqs=4 Similarity=0.838384 tpcCG=39.099998 11 TCCTTCCAGAAGATTATTTTGT

* ***** ******* ** *

Consensus:

TccTTCCAgAAGATTAcTTcgT

>arqueas_representative_Fam_304_22_2 Nr. of seq. 2 Alignment length(with gaps) = 22 Alignment score = 0.606061

arqueas_representative:NZ_CP009512.1_Methanosarcina_mazei_S-6:85:3053773-3053878 Satlength=106 Nr of Repeats=5 RepeatLength=21 seed=AATACTAAAA Num.seqs=5 Similarity=0.866667 tpcCG=41.400002 0 AATACT-AAAAGTTTCTATAAT

Rev.of_arqueas_representative:NZ_CP009517.1_Methanosarcina_barkeri_3:81:4318439-4318626 Satlength=188 Nr of Repeats=9 RepeatLength=22 seed=TTTACAGAAA Num.seqs=8 Similarity=1.000000 tpcCG=39.099998 11 CTTTCTGTAAACTTTCTATAAT

* ** *** **********

Consensus:

aaTaCTgaAAAcTTTCTATAAT

>arqueas_representative_Fam_305_22_2 Nr. of seq. 2 Alignment length(with gaps) = 22 Alignment score = 0.606061

arqueas_representative:NZ_CP009520.1_Methanosarcina_vacuolata_Z-761:88:3013551-3013679 Satlength=129 Nr of Repeats=6 RepeatLength=21 seed=AATTCAAGGA Num.seqs=4 Similarity=1.000000 tpcCG=39.732498 0 AATTC-AAGGAAAGTTTAAGGG

arqueas_representative:NZ_CP009517.1_Methanosarcina_barkeri_3:81:2514164-2514274 Satlength=111 Nr of Repeats=5 RepeatLength=22 seed=AGAGAAATCT Num.seqs=5 Similarity=0.975758 tpcCG=39.099998 18 AAATCTAAGGGAGATTTAAGAG

** ** **** * ****** *

Consensus:

AAaTCtAAGGaAaaTTTAAGaG

>arqueas_representative_Fam_306_22_2 Nr. of seq. 2 Alignment length(with gaps) = 22 Alignment score = 0.643939

arqueas_representative:NZ_CP009520.1_Methanosarcina_vacuolata_Z-761:88:4275644-4275917 Satlength=274 Nr of Repeats=13 RepeatLength=21 seed=TCTTTGAGAA Num.seqs=13 Similarity=1.000000 tpcCG=39.732498 0 TCTTTGAGAAATCTCATTCTA-

Rev.of_arqueas_representative:NZ_CP009517.1_Methanosarcina_barkeri_3:81:851973-852149 Satlength=177 Nr of Repeats=8 RepeatLength=22 seed=GTAGAATAAC Num.seqs=8 Similarity=0.984848 tpcCG=39.099998 0 TCTTTAAGATAAGTTATTCTAC

***** *** * * ******

Consensus:

TCTTTaAGAaAacTcATTCTAc

>arqueas_representative_Fam_307_22_2 Nr. of seq. 2 Alignment length(with gaps) = 22 Alignment score = 0.636364

arqueas_representative:NC_017941.2_Haloferax_mediterranei_ATCC_33500:25:2033644-2033732 Satlength=89 Nr of Repeats=4 RepeatLength=22 seed=TCGTGATGTG Num.seqs=4 Similarity=1.000000 tpcCG=60.255100 0 TCGTGATGTGGTTGTGAGGGGG

arqueas_representative:NC_013922.1_Natrialba_magadii_ATCC_43099:100:532483-532659 Satlength=177 Nr of Repeats=8 RepeatLength=22 seed=GTGGTGTTGT Num.seqs=6 Similarity=1.000000 tpcCG=61.032001 13 TTGTGATGTGGCGGTGGTGGTG

* ********* *** ** *

Consensus:

TcGTGATGTGGcgGTGagGGgG

>arqueas_representative_Fam_308_22_2 Nr. of seq. 2 Alignment length(with gaps) = 22 Alignment score = 0.636364

arqueas_representative:NZ_CP009517.1_Methanosarcina_barkeri_3:81:2644423-2644511 Satlength=89 Nr of Repeats=4 RepeatLength=22 seed=AAATTCAGAG Num.seqs=4 Similarity=1.000000 tpcCG=39.099998 0 AAATTCAGAGTAGAATTATTAA

arqueas_representative:NZ_CP009515.1_Methanosarcina_lacustris_Z-7289:84:426269-426401 Satlength=133 Nr of Repeats=6 RepeatLength=22 seed=TTGAATTATA Num.seqs=6 Similarity=0.903030 tpcCG=41.799999 10 AAATAAATTGTTGAATTATAAA

**** * ** ******* **

Consensus:

AAATaaAgaGTaGAATTATaAA

>arqueas_representative_Fam_309_22_2 Nr. of seq. 2 Alignment length(with gaps) = 22 Alignment score = 0.696970

arqueas_representative:NZ_CP009528.1_Methanosarcina_barkeri_MS:82:3639866-3639954 Satlength=89 Nr of Repeats=4 RepeatLength=22 seed=TAAAAATGTA Num.seqs=4 Similarity=1.000000 tpcCG=39.168999 0 TAAAAATGTATTATATTAATCT

Rev.of_arqueas_representative:NZ_CP009512.1_Methanosarcina_mazei_S-6:85:4007443-4007532 Satlength=90 Nr of Repeats=4 RepeatLength=22 seed=TTAATCCAAT Num.seqs=3 Similarity=1.000000 tpcCG=41.400002 19 TAAAAACATATTGGATTAATGT

****** **** ****** *

Consensus:

TAAAAAcaTATTagATTAATcT

>arqueas_representative_Fam_310_21_2 Nr. of seq. 2 Alignment length(with gaps) = 21 Alignment score = 0.603175

arqueas_representative:NZ_CP017921.1_Methanohalophilus_halophilus__Z-7982_:71:748159-748261 Satlength=103 Nr of Repeats=5 RepeatLength=18 seed=GAACCTGAAG Num.seqs=3 Similarity=0.827160 tpcCG=42.400002 0 GAACCTGAAG-AACC-AG-NA

arqueas_representative:NZ_CP009506.1_Methanosarcina_siciliae_T4/M:86:1766592-1766991 Satlength=400 Nr of Repeats=16 RepeatLength=21 seed=GAAGCTGAAG Num.seqs=13 Similarity=0.938136 tpcCG=42.900002 0 GAAGCTGAAGTAACCGAGACA

*** ****** **** ** *

Consensus:

GAAcCTGAAGtAACCgAGacA

>arqueas_representative_Fam_311_21_2 Nr. of seq. 2 Alignment length(with gaps) = 21 Alignment score = 0.658730

arqueas_representative:NZ_CP009517.1_Methanosarcina_barkeri_3:81:3468407-3468532 Satlength=126 Nr of Repeats=5 RepeatLength=18 seed=AAGCAGAAAC Num.seqs=3 Similarity=1.000000 tpcCG=39.099998 0 AAGC--AGAAACCCGAAATT-

arqueas_representative:NZ_CP009515.1_Methanosarcina_lacustris_Z-7289:84:3418965-3419133 Satlength=169 Nr of Repeats=8 RepeatLength=21 seed=AACCCTAAAT Num.seqs=8 Similarity=0.941043 tpcCG=41.799999 9 AACCTAAGAAACCCTAAATTA

** * ******** *****

Consensus:

AAcCtaAGAAACCCgAAATTa

>arqueas_representative_Fam_312_21_2 Nr. of seq. 2 Alignment length(with gaps) = 21 Alignment score = 0.658730

arqueas_representative:NZ_CP009528.1_Methanosarcina_barkeri_MS:82:1268730-1268928 Satlength=199 Nr of Repeats=11 RepeatLength=18 seed=TCATTTTCTA Num.seqs=11 Similarity=1.000000 tpcCG=39.168999 0 TCATTTTCTATACTT--TGT-

Rev.of_arqueas_representative:NC_003552.1_Methanosarcina_acetivorans_str._C2A:80:2472578-2472662 Satlength=85 Nr of Repeats=4 RepeatLength=21 seed=GAAAATGAAA Num.seqs=4 Similarity=0.805556 tpcCG=42.70 8 TCATTTTCTACAATTCCTGTT

********** * ** ***

Consensus:

TCATTTTCTAcAaTTccTGTt

>arqueas_representative_Fam_313_21_2 Nr. of seq. 2 Alignment length(with gaps) = 21 Alignment score = 0.611111

arqueas_representative:NZ_CP009512.1_Methanosarcina_mazei_S-6:85:1482808-1483006 Satlength=199 Nr of Repeats=11 RepeatLength=18 seed=ATAATCTGAA Num.seqs=11 Similarity=1.000000 tpcCG=41.400002 0 ATAATCTGAATAGGTTTG---

Rev.of_arqueas_representative:NZ_CP009501.1_Methanosarcina_thermophila_TM-1:87:461689-461769 Satlength=81 Nr of Repeats=4 RepeatLength=20 seed=AAATCTTTCA Num.seqs=4 Similarity=0.788889 tpcCG=41.099998 16 ATAACCTGAA-AGATTTGCAT

**** ***** ** ****

Consensus:

ATAAcCTGAAtAGaTTTGcat

>arqueas_representative_Fam_314_21_2 Nr. of seq. 2 Alignment length(with gaps) = 21 Alignment score = 0.626984

arqueas_representative:NZ_CP009517.1_Methanosarcina_barkeri_3:81:2518080-2518234 Satlength=155 Nr of Repeats=8 RepeatLength=19 seed=GGATAACTCA Num.seqs=6 Similarity=0.962573 tpcCG=39.099998 0 GGATAA-CTCAACACTTTGA-

Rev.of_arqueas_representative:NZ_CP009515.1_Methanosarcina_lacustris_Z-7289:84:1632241-1632341 Satlength=101 Nr of Repeats=5 RepeatLength=20 seed=TTATCCTTTA Num.seqs=5 Similarity=1.000000 tpcCG=41.799 6 GGATAACCTGAA-ACTTTAAA

****** ** ** ***** *

Consensus:

GGATAAcCTcAAcACTTTaAa

>arqueas_representative_Fam_315_21_2 Nr. of seq. 2 Alignment length(with gaps) = 21 Alignment score = 0.626984

arqueas_representative:NZ_CP009517.1_Methanosarcina_barkeri_3:81:3020190-3020304 Satlength=115 Nr of Repeats=6 RepeatLength=19 seed=ATAATTAGTT Num.seqs=6 Similarity=0.953216 tpcCG=39.099998 0 -ATAATT-AGTTTAAACTCAG

arqueas_representative:NZ_CP009520.1_Methanosarcina_vacuolata_Z-761:88:3190469-3190559 Satlength=91 Nr of Repeats=4 RepeatLength=20 seed=TCTAATTAAG Num.seqs=3 Similarity=0.955556 tpcCG=39.732498 0 TCTAATTAAGTTT-AACTAAG

***** ***** **** **

Consensus:

taTAATTaAGTTTaAACTaAG

>arqueas_representative_Fam_316_21_2 Nr. of seq. 2 Alignment length(with gaps) = 21 Alignment score = 0.603175

arqueas_representative:NZ_CP009515.1_Methanosarcina_lacustris_Z-7289:84:2561931-2562007 Satlength=77 Nr of Repeats=4 RepeatLength=19 seed=GGTACTGATG Num.seqs=4 Similarity=0.964912 tpcCG=41.799999 0 GGTACTGA--TGAGAAAATCG

arqueas_representative:NZ_CP009512.1_Methanosarcina_mazei_S-6:85:3558236-3558445 Satlength=210 Nr of Repeats=11 RepeatLength=19 seed=AAAATCCTCC Num.seqs=11 Similarity=1.000000 tpcCG=41.400002 12 --TCCTGACCTGAGAAAATCC

* **** **********

Consensus:

ggTaCTGAccTGAGAAAATCc

>arqueas_representative_Fam_317_21_2 Nr. of seq. 2 Alignment length(with gaps) = 21 Alignment score = 0.698413

arqueas_representative:NZ_CP009520.1_Methanosarcina_vacuolata_Z-761:88:408850-409289 Satlength=440 Nr of Repeats=7 RepeatLength=19 seed=CCTTTCTTTC Num.seqs=5 Similarity=1.000000 tpcCG=39.732498 0 CCTTTCTTTCTACTTTTTT--

arqueas_representative:NZ_CP009512.1_Methanosarcina_mazei_S-6:85:1740894-1741041 Satlength=148 Nr of Repeats=7 RepeatLength=21 seed=ATTTCTTTCT Num.seqs=7 Similarity=1.000000 tpcCG=41.400002 1 CATTTCTTTCTGCTTTCTTCT

* ********* **** **

Consensus:

CaTTTCTTTCTaCTTTcTTct

>arqueas_representative_Fam_318_21_2 Nr. of seq. 2 Alignment length(with gaps) = 21 Alignment score = 0.722222

arqueas_representative:NC_013790.1_Methanobrevibacter_ruminantium_M1:50:472171-472281 Satlength=111 Nr of Repeats=6 RepeatLength=20 seed=ATTTTTACTT Num.seqs=5 Similarity=0.788889 tpcCG=32.599998 4 ATTC-ATTTTTACTTATTTTT

Rev.of_arqueas_representative:NZ_CP009517.1_Methanosarcina_barkeri_3:81:2708099-2708203 Satlength=105 Nr of Repeats=5 RepeatLength=20 seed=CATAATAAAA Num.seqs=3 Similarity=1.000000 tpcCG=39.099998 5 ATTATGTTTTTAC-TATTTTT

******* **********

Consensus:

aTTTTTACtTATTTTTATTat

>arqueas_representative_Fam_319_21_2 Nr. of seq. 2 Alignment length(with gaps) = 21 Alignment score = 0.650794

arqueas_representative:NZ_CP009517.1_Methanosarcina_barkeri_3:81:265700-265840 Satlength=141 Nr of Repeats=7 RepeatLength=20 seed=CTTACTTATT Num.seqs=7 Similarity=1.000000 tpcCG=39.099998 0 CTTACTTATTTC-TCAGATAC

Rev.of_arqueas_representative:NZ_CP009517.1_Methanosarcina_barkeri_3:81:1342629-1343049 Satlength=421 Nr of Repeats=4 RepeatLength=21 seed=AAGGAAACTG Num.seqs=3 Similarity=0.957672 tpcCG=39.099998 3 CTTGATTATTTCTTCAGTTTC

*** ******* **** * *

Consensus:

CTTaaTTATTTCtTCAGaTaC

>arqueas_representative_Fam_320_21_2 Nr. of seq. 2 Alignment length(with gaps) = 21 Alignment score = 0.626984

arqueas_representative:NZ_CP009528.1_Methanosarcina_barkeri_MS:82:2690331-2690431 Satlength=101 Nr of Repeats=5 RepeatLength=20 seed=ATACTTACTG Num.seqs=5 Similarity=1.000000 tpcCG=39.168999 0 ATACTTACTGGCTTCTAGCC-

Rev.of_arqueas_representative:NC_003552.1_Methanosarcina_acetivorans_str._C2A:80:4413039-4413219 Satlength=181 Nr of Repeats=5 RepeatLength=21 seed=GGAAGTAAAG Num.seqs=4 Similarity=0.968254 tpcCG=42.7 8 TTACTTCCTGCCTTCTCTCCT

***** *** ***** **

Consensus:

aTACTTaCTGcCTTCTagCCt

>arqueas_representative_Fam_321_21_2 Nr. of seq. 2 Alignment length(with gaps) = 21 Alignment score = 0.650794

arqueas_representative:NZ_CP009515.1_Methanosarcina_lacustris_Z-7289:84:2557858-2558038 Satlength=181 Nr of Repeats=9 RepeatLength=20 seed=AAAAACAGAG Num.seqs=9 Similarity=1.000000 tpcCG=41.799999 0 AAAAACAGAGTGGAAG-TGAG

arqueas_representative:NC_003552.1_Methanosarcina_acetivorans_str._C2A:80:2616302-2616491 Satlength=190 Nr of Repeats=9 RepeatLength=21 seed=AGGAAGCTAA Num.seqs=9 Similarity=0.936508 tpcCG=42.700001 10 AATAACAGACAGGAAGCTAAG

** ****** ***** * **

Consensus:

AAaAACAGAcaGGAAGcTaAG

>arqueas_representative_Fam_322_21_2 Nr. of seq. 2 Alignment length(with gaps) = 21 Alignment score = 0.619048

arqueas_representative:NC_021921.1_Halorhabdus_tiamatea_SARL4B_:32:521635-521779 Satlength=145 Nr of Repeats=5 RepeatLength=21 seed=CGACGGCGGC Num.seqs=3 Similarity=0.957672 tpcCG=62.770000 0 CGACGACGGCGGCGAACAGAC

Rev.of_arqueas_representative:NC_013743.1_Haloterrigena_turkmenica_DSM_5511:38:1447666-1447879 Satlength=214 Nr of Repeats=4 RepeatLength=21 seed=GCCGTCGTCC Num.seqs=3 Similarity=0.915344 tpcCG=64.248 13 CGCGGACGACGGCGAACCATC

** **** ******** *

Consensus:

CGacGACGaCGGCGAACaaaC

>arqueas_representative_Fam_323_21_2 Nr. of seq. 2 Alignment length(with gaps) = 21 Alignment score = 0.682540

arqueas_representative:NC_012029.1_Halorubrum_lacusprofundi_ATCC_49239_chromosome_1:34:1648675-1648849 Satlength=175 Nr of Repeats=4 RepeatLength=21 seed=CGTCGGTCGC Num.seqs=3 Similarity=0.873016 tpcC 0 CGTCGGTCGCGTTCGTCGGGT

arqueas_representative:NC_019792.1_Natronobacterium_gregoryi_SP2:102:3046562-3046898 Satlength=337 Nr of Repeats=16 RepeatLength=21 seed=CGCCCGTCGT Num.seqs=16 Similarity=0.831746 tpcCG=62.200001 0 CGCCCGTCGTGGTCGTCGAGT

** * **** * ****** **

Consensus:

CGcCcGTCGcGgTCGTCGaGT

>arqueas_representative_Fam_324_21_2 Nr. of seq. 2 Alignment length(with gaps) = 21 Alignment score = 0.682540

arqueas_representative:NZ_CP007055.1_Halostagnicola_larsenii_XH-48:36:657217-657709 Satlength=493 Nr of Repeats=5 RepeatLength=21 seed=AACGAGACGG Num.seqs=3 Similarity=0.788360 tpcCG=60.865299 0 AACGAGACGGCGACTGCTGAC

Rev.of_arqueas_representative:NC_013743.1_Haloterrigena_turkmenica_DSM_5511:38:331246-331402 Satlength=157 Nr of Repeats=6 RepeatLength=21 seed=TGTCGTTGTC Num.seqs=4 Similarity=0.642677 tpcCG=64.24839 7 AACGACACCGAGTCCGCTGAC

***** ** * * * ******

Consensus:

AACGAcACcGaGaCcGCTGAC

>arqueas_representative_Fam_325_21_2 Nr. of seq. 2 Alignment length(with gaps) = 21 Alignment score = 0.873016

arqueas_representative:NC_019943.1_Methanoregula_formicica_SMSP:77:818855-819272 Satlength=418 Nr of Repeats=6 RepeatLength=21 seed=TGCTTGAGAA Num.seqs=4 Similarity=0.925926 tpcCG=55.200001 0 TGCTTGAGAAACAGGATCAGA

Rev.of_arqueas_representative:NC_007796.1_Methanospirillum_hungatei_JF-1:91:3344247-3344394 Satlength=148 Nr of Repeats=7 RepeatLength=21 seed=CATCTGATCC Num.seqs=7 Similarity=0.969766 tpcCG=45.099998 2 TGCTGGATAAACAGGATCAGA

**** ** *************

Consensus:

TGCTgGAgAAACAGGATCAGA

>arqueas_representative_Fam_326_21_2 Nr. of seq. 2 Alignment length(with gaps) = 21 Alignment score = 0.682540

arqueas_representative:NC_003552.1_Methanosarcina_acetivorans_str._C2A:80:242874-242957 Satlength=84 Nr of Repeats=4 RepeatLength=21 seed=TTTAAATTAA Num.seqs=3 Similarity=0.957672 tpcCG=42.700001 0 TTTAAATTAAAAATGGTATAC

Rev.of_arqueas_representative:NZ_CP009520.1_Methanosarcina_vacuolata_Z-761:88:926646-926789 Satlength=144 Nr of Repeats=6 RepeatLength=21 seed=ATTACTTATT Num.seqs=5 Similarity=0.793939 tpcCG=39.732498 14 TTTGAATAAGTAATTGTATAC

*** *** * *** ******

Consensus:

TTTaAATaAaaAATgGTATAC

>arqueas_representative_Fam_327_21_2 Nr. of seq. 2 Alignment length(with gaps) = 21 Alignment score = 0.634921

arqueas_representative:NC_003552.1_Methanosarcina_acetivorans_str._C2A:80:2181878-2182415 Satlength=538 Nr of Repeats=13 RepeatLength=21 seed=TTCTTCAGCT Num.seqs=9 Similarity=0.940035 tpcCG=42.700001 0 TTCTTCAGCTTCTTCCAAGTC

arqueas_representative:NZ_CP009528.1_Methanosarcina_barkeri_MS:82:2707127-2707316 Satlength=190 Nr of Repeats=9 RepeatLength=21 seed=CAACTACATC Num.seqs=9 Similarity=0.832452 tpcCG=39.168999 5 CTCTTCAACTACATCNAATTC

****** ** * ** ** **

Consensus:

cTCTTCAaCTaCaTCcAAgTC

>arqueas_representative_Fam_328_21_2 Nr. of seq. 2 Alignment length(with gaps) = 21 Alignment score = 0.682540

arqueas_representative:NC_003552.1_Methanosarcina_acetivorans_str._C2A:80:3653303-3653471 Satlength=169 Nr of Repeats=8 RepeatLength=21 seed=CTGTTACATT Num.seqs=8 Similarity=0.802721 tpcCG=42.700001 0 CTGTTACATTTTCCTCAGGTA

arqueas_representative:NC_003552.1_Methanosarcina_acetivorans_str._C2A:80:3653494-3653704 Satlength=211 Nr of Repeats=10 RepeatLength=21 seed=GTTATGTTCT Num.seqs=10 Similarity=0.932275 tpcCG=42.700001 2 CCGTTATGTTCTCCTCTGGTA

* **** ** ***** ****

Consensus:

CcGTTAcaTTcTCCTCaGGTA

>arqueas_representative_Fam_329_20_2 Nr. of seq. 2 Alignment length(with gaps) = 20 Alignment score = 0.641667

arqueas_representative:NC_013790.1_Methanobrevibacter_ruminantium_M1:50:541780-542597 Satlength=818 Nr of Repeats=10 RepeatLength=16 seed=TAATCTTTTT Num.seqs=6 Similarity=0.730719 tpcCG=32.599998 0 TAATCTTTTTAAATCNT---

arqueas_representative:NZ_CP009528.1_Methanosarcina_barkeri_MS:82:1780207-1780287 Satlength=81 Nr of Repeats=4 RepeatLength=20 seed=TTCTTTTTAA Num.seqs=4 Similarity=1.000000 tpcCG=39.168999 2 TATTCTTTTTAAATCCAGCG

** ************

Consensus:

TAaTCTTTTTAAATCcagcg

>arqueas_representative_Fam_330_20_2 Nr. of seq. 2 Alignment length(with gaps) = 20 Alignment score = 0.750000

arqueas_representative:NC_014222.1_Methanococcus_voltae_A3:65:1785617-1786322 Satlength=706 Nr of Repeats=5 RepeatLength=18 seed=AATAAATTAA Num.seqs=3 Similarity=0.827160 tpcCG=28.600000 0 AATAAAT-TAAT-AAANTAT

arqueas_representative:NC_003552.1_Methanosarcina_acetivorans_str._C2A:80:3323049-3323149 Satlength=101 Nr of Repeats=5 RepeatLength=20 seed=AAAATATAAT Num.seqs=5 Similarity=0.906667 tpcCG=42.700001 13 AATAAATATAATCAAAATAT

******* **** *** ***

Consensus:

AATAAATaTAATcAAAaTAT

>arqueas_representative_Fam_331_20_2 Nr. of seq. 2 Alignment length(with gaps) = 20 Alignment score = 0.716667

arqueas_representative:NZ_CP009528.1_Methanosarcina_barkeri_MS:82:773483-773627 Satlength=145 Nr of Repeats=8 RepeatLength=18 seed=CCATGCAATA Num.seqs=8 Similarity=0.960317 tpcCG=39.168999 0 CCATGCAATATCAATAAT--

Rev.of_arqueas_representative:NZ_CP009528.1_Methanosarcina_barkeri_MS:82:1442906-1442982 Satlength=77 Nr of Repeats=4 RepeatLength=19 seed=TTATTCATAT Num.seqs=4 Similarity=1.000000 tpcCG=39.168999 16 CC-TGCAATATGAATAATCA

** ******** ******

Consensus:

CCaTGCAATATcAATAATca

>arqueas_representative_Fam_332_20_2 Nr. of seq. 2 Alignment length(with gaps) = 20 Alignment score = 0.641667

arqueas_representative:NZ_CP009515.1_Methanosarcina_lacustris_Z-7289:84:1664756-1664828 Satlength=73 Nr of Repeats=4 RepeatLength=18 seed=ACTTCTGTTT Num.seqs=4 Similarity=0.950617 tpcCG=41.799999 0 ACTT-CTGTTTCATTCTTC-

arqueas_representative:NZ_CP009520.1_Methanosarcina_vacuolata_Z-761:88:2643568-2643768 Satlength=201 Nr of Repeats=10 RepeatLength=20 seed=GGTTAATTCT Num.seqs=10 Similarity=0.928889 tpcCG=39.732498 7 AGTTACTGGTTAATTCTTCA

* ** *** ** *******

Consensus:

AcTTaCTGgTTaATTCTTCa

>arqueas_representative_Fam_333_20_2 Nr. of seq. 2 Alignment length(with gaps) = 20 Alignment score = 0.683333

arqueas_representative:NZ_CP009512.1_Methanosarcina_mazei_S-6:85:743279-743351 Satlength=73 Nr of Repeats=4 RepeatLength=18 seed=AAAAATAATC Num.seqs=4 Similarity=1.000000 tpcCG=41.400002 0 AAAAATAATCTGAATTAG--

arqueas_representative:NZ_CP009520.1_Methanosarcina_vacuolata_Z-761:88:4338773-4338913 Satlength=141 Nr of Repeats=7 RepeatLength=20 seed=AACCAGAATT Num.seqs=7 Similarity=0.787302 tpcCG=39.732498 6 CAAAATAACCAGAATTAGNT

******* * *******

Consensus:

aAAAATAAcCaGAATTAGnt

>arqueas_representative_Fam_334_20_2 Nr. of seq. 2 Alignment length(with gaps) = 20 Alignment score = 0.700000

arqueas_representative:NZ_CP009506.1_Methanosarcina_siciliae_T4/M:86:1329923-1329995 Satlength=73 Nr of Repeats=4 RepeatLength=18 seed=ATCAAATTTA Num.seqs=4 Similarity=1.000000 tpcCG=42.900002 0 ATCAAATTTAAT-CCA-GAG

Rev.of_arqueas_representative:NZ_CP009506.1_Methanosarcina_siciliae_T4/M:86:1662071-1662147 Satlength=77 Nr of Repeats=4 RepeatLength=19 seed=AATTGATCTC Num.seqs=4 Similarity=1.000000 tpcCG=42.900002 7 ATC-AATTTAATACCAGGAG

*** ******** *** ***

Consensus:

ATCaAATTTAATaCCAgGAG

>arqueas_representative_Fam_335_20_2 Nr. of seq. 2 Alignment length(with gaps) = 20 Alignment score = 0.608333

arqueas_representative:NZ_CP009520.1_Methanosarcina_vacuolata_Z-761:88:1330009-1330153 Satlength=145 Nr of Repeats=8 RepeatLength=18 seed=AAAAACGAAC Num.seqs=8 Similarity=0.912698 tpcCG=39.732498 0 AAAAAC-GAACCTGATACG-

Rev.of_arqueas_representative:NZ_CP009515.1_Methanosarcina_lacustris_Z-7289:84:1207349-1207425 Satlength=77 Nr of Repeats=4 RepeatLength=19 seed=ATAATGTTCA Num.seqs=4 Similarity=1.000000 tpcCG=41.7999 16 AAAAACTGAACATTAT-CGA

****** **** * ** **

Consensus:

AAAAACtGAACaTgATaCGa

>arqueas_representative_Fam_336_20_2 Nr. of seq. 2 Alignment length(with gaps) = 20 Alignment score = 0.633333

arqueas_representative:NC_013790.1_Methanobrevibacter_ruminantium_M1:50:2556502-2556598 Satlength=97 Nr of Repeats=5 RepeatLength=19 seed=CTAGTAAAAA Num.seqs=4 Similarity=1.000000 tpcCG=32.599998 0 CTAGT-AAAAAAAGAATATG

arqueas_representative:NZ_CP009520.1_Methanosarcina_vacuolata_Z-761:88:3805226-3805306 Satlength=81 Nr of Repeats=4 RepeatLength=20 seed=ATTCAAAAAA Num.seqs=4 Similarity=0.900000 tpcCG=39.732498 2 AAATTCAAAAAAAGGATATG

* * ******** *****

Consensus:

aaAgTcAAAAAAAGaATATG

>arqueas_representative_Fam_337_20_2 Nr. of seq. 2 Alignment length(with gaps) = 20 Alignment score = 0.608333

arqueas_representative:NZ_CP009517.1_Methanosarcina_barkeri_3:81:294260-294808 Satlength=549 Nr of Repeats=4 RepeatLength=19 seed=AAAAATTCAT Num.seqs=3 Similarity=1.000000 tpcCG=39.099998 0 AAAAATTCATATATAGACA-

Rev.of_arqueas_representative:NZ_CP009512.1_Methanosarcina_mazei_S-6:85:3796845-3797045 Satlength=201 Nr of Repeats=10 RepeatLength=20 seed=AATGAATTTA Num.seqs=10 Similarity=0.962963 tpcCG=41.400002 11 GTAAATTCATTTATAAAGAG

******** **** * *

Consensus:

aaAAATTCATaTATAaAcAg

>arqueas_representative_Fam_338_20_2 Nr. of seq. 2 Alignment length(with gaps) = 20 Alignment score = 0.666667

arqueas_representative:NZ_CP009517.1_Methanosarcina_barkeri_3:81:829127-829240 Satlength=114 Nr of Repeats=6 RepeatLength=19 seed=TAAAATATAC Num.seqs=5 Similarity=0.817544 tpcCG=39.099998 0 TAAA-ATATACTTTAATAGT

arqueas_representative:CP015520.1_Thermococcus_piezophilus__CDGS_:133:619679-619888 Satlength=210 Nr of Repeats=11 RepeatLength=19 seed=ATATCATTTA Num.seqs=11 Similarity=1.000000 tpcCG=51.099998 5 TAAATATATCATTTAA-AGT

**** **** ***** ***

Consensus:

TAAAtATATaaTTTAAtAGT

>arqueas_representative_Fam_339_20_2 Nr. of seq. 2 Alignment length(with gaps) = 20 Alignment score = 0.633333

arqueas_representative:NZ_CP009517.1_Methanosarcina_barkeri_3:81:1302033-1302128 Satlength=96 Nr of Repeats=4 RepeatLength=19 seed=TAAAAGGACT Num.seqs=3 Similarity=0.953216 tpcCG=39.099998 0 TAAAAGGA-CTAAGAAGCAA

arqueas_representative:NZ_CP009515.1_Methanosarcina_lacustris_Z-7289:84:1831093-1831188 Satlength=96 Nr of Repeats=5 RepeatLength=20 seed=AAAAAGAGGT Num.seqs=3 Similarity=1.000000 tpcCG=41.799999 1 AAAAAAGAGGTAAGATGCAA

**** ** ***** ****

Consensus:

aAAAAaGAgcTAAGAaGCAA

>arqueas_representative_Fam_340_20_2 Nr. of seq. 2 Alignment length(with gaps) = 20 Alignment score = 0.666667

arqueas_representative:NZ_CP009517.1_Methanosarcina_barkeri_3:81:1330959-1331035 Satlength=77 Nr of Repeats=4 RepeatLength=19 seed=TTTATGGAAG Num.seqs=4 Similarity=0.964912 tpcCG=39.099998 0 TT-TATGGAAGTTTAGTGAT

Rev.of_arqueas_representative:NZ_CP009517.1_Methanosarcina_barkeri_3:81:3162787-3162901 Satlength=115 Nr of Repeats=6 RepeatLength=19 seed=ACACAAACTT Num.seqs=6 Similarity=1.000000 tpcCG=39.099998 18 TTATCTGGAAGTTT-GTGTT

** * ********* *** *

Consensus:

TTaTaTGGAAGTTTaGTGaT

>arqueas_representative_Fam_341_20_2 Nr. of seq. 2 Alignment length(with gaps) = 20 Alignment score = 0.666667

arqueas_representative:NZ_CP009528.1_Methanosarcina_barkeri_MS:82:1198790-1198866 Satlength=77 Nr of Repeats=4 RepeatLength=19 seed=TAAAAATATG Num.seqs=4 Similarity=1.000000 tpcCG=39.168999 0 TAAAAAT-ATGCAACGTATG

Rev.of_arqueas_representative:NZ_CP009506.1_Methanosarcina_siciliae_T4/M:86:3861900-3861993 Satlength=94 Nr of Repeats=5 RepeatLength=19 seed=AAATAGTTAC Num.seqs=4 Similarity=1.000000 tpcCG=42.900002 1 TAAAAATCATGTAAC-TATT

******* *** *** ***

Consensus:

TAAAAATcATGcAACgTATg

>arqueas_representative_Fam_342_20_2 Nr. of seq. 2 Alignment length(with gaps) = 20 Alignment score = 0.700000

arqueas_representative:NZ_CP009512.1_Methanosarcina_mazei_S-6:85:851251-851327 Satlength=77 Nr of Repeats=4 RepeatLength=19 seed=TAAAAGTCAG Num.seqs=4 Similarity=0.964912 tpcCG=41.400002 0 TAAAAGTCAG-TAGAATAAA

arqueas_representative:NZ_CP009517.1_Methanosarcina_barkeri_3:81:146304-146464 Satlength=161 Nr of Repeats=8 RepeatLength=20 seed=TCAGCTTGAA Num.seqs=8 Similarity=0.807143 tpcCG=39.099998 6 TCAAATTCAGCTTGAATAAA

* *** **** * *******

Consensus:

TaAAAgTCAGcTaGAATAAA

>arqueas_representative_Fam_343_20_2 Nr. of seq. 2 Alignment length(with gaps) = 20 Alignment score = 0.766667

arqueas_representative:NZ_CP009520.1_Methanosarcina_vacuolata_Z-761:88:847864-847940 Satlength=77 Nr of Repeats=4 RepeatLength=19 seed=ATAAAATAAG Num.seqs=4 Similarity=0.964912 tpcCG=39.732498 2 AAATAAAATAAGG-TAAAAG

Rev.of_arqueas_representative:NC_013790.1_Methanobrevibacter_ruminantium_M1:50:577272-577987 Satlength=716 Nr of Repeats=9 RepeatLength=20 seed=TTTATCCTTT Num.seqs=8 Similarity=0.971429 tpcCG=32.59999 18 AATTAAAAAAAGGATAAAAG

***** **** ********

Consensus:

aTAAAAaAAGGaTAAAAGAA

>arqueas_representative_Fam_344_20_2 Nr. of seq. 2 Alignment length(with gaps) = 20 Alignment score = 0.675000

arqueas_representative:NZ_CP009520.1_Methanosarcina_vacuolata_Z-761:88:3231831-3232230 Satlength=400 Nr of Repeats=21 RepeatLength=19 seed=TACAAAATTT Num.seqs=21 Similarity=1.000000 tpcCG=39.732498 0 TACAAAATTTAAGTCAATA-

Rev.of_arqueas_representative:NC_013790.1_Methanobrevibacter_ruminantium_M1:50:1753725-1753801 Satlength=77 Nr of Repeats=4 RepeatLength=19 seed=GTAAAATTGA Num.seqs=4 Similarity=0.600000 tpcCG=32.5999 3 TACGAGATTCAAGTCAATTT

*** * *** ********

Consensus:

TACaAaATTcAAGTCAATat

>arqueas_representative_Fam_345_20_2 Nr. of seq. 2 Alignment length(with gaps) = 20 Alignment score = 0.666667

arqueas_representative:NZ_CP014265.1_Methanobrevibacter_olleyae__YLM1:49:7663-7771 Satlength=109 Nr of Repeats=5 RepeatLength=20 seed=TTTTTATTTT Num.seqs=3 Similarity=0.911111 tpcCG=26.900000 0 TTTTTATTTTTATTAGTTAA

Rev.of_arqueas_representative:NZ_CP009528.1_Methanosarcina_barkeri_MS:82:2433917-2434037 Satlength=121 Nr of Repeats=6 RepeatLength=20 seed=TTAAAAAACT Num.seqs=6 Similarity=0.920000 tpcCG=39.168999 16 TTTTTAAGTTTTTTAACTAA

****** *** *** ***

Consensus:

TTTTTAagTTTaTTAacTAA

>arqueas_representative_Fam_346_20_2 Nr. of seq. 2 Alignment length(with gaps) = 20 Alignment score = 0.666667

arqueas_representative:NZ_CP009528.1_Methanosarcina_barkeri_MS:82:3371986-3372086 Satlength=101 Nr of Repeats=5 RepeatLength=20 seed=TTTTTACTTC Num.seqs=5 Similarity=1.000000 tpcCG=39.168999 3 TATTTTTTACTTCTTTTGTA

arqueas_representative:NZ_CP009506.1_Methanosarcina_siciliae_T4/M:86:3596366-3596486 Satlength=121 Nr of Repeats=6 RepeatLength=20 seed=ATTTGATTCT Num.seqs=6 Similarity=1.000000 tpcCG=42.900002 21 TATTTGATTCTTATTATGTA

** * *** ** *******

Consensus:

TTgaTaCTTaTTaTGTATAT

>arqueas_representative_Fam_347_19_2 Nr. of seq. 2 Alignment length(with gaps) = 19 Alignment score = 0.657895

arqueas_representative:NZ_CP009528.1_Methanosarcina_barkeri_MS:82:1110653-1110749 Satlength=97 Nr of Repeats=6 RepeatLength=16 seed=AATAAGCAAA Num.seqs=6 Similarity=1.000000 tpcCG=39.168999 0 AATAA-GCAAATAATC-G-

Rev.of_arqueas_representative:NZ_CP009520.1_Methanosarcina_vacuolata_Z-761:88:2242499-2242760 Satlength=262 Nr of Repeats=14 RepeatLength=19 seed=TTCAGATTAT Num.seqs=9 Similarity=0.894737 tpcCG=39.732 1 AATAATTCAAATAATCTGA

***** ********* *

Consensus:

AATAAtgCAAATAATCtGa

>arqueas_representative_Fam_348_19_2 Nr. of seq. 2 Alignment length(with gaps) = 19 Alignment score = 0.649123

arqueas_representative:NC_003552.1_Methanosarcina_acetivorans_str._C2A:80:1718057-1718142 Satlength=86 Nr of Repeats=5 RepeatLength=17 seed=GGATAAGAGA Num.seqs=5 Similarity=0.921569 tpcCG=42.700001 0 GGATAAGAG-ATA-CTTCA

Rev.of_arqueas_representative:NZ_CP009501.1_Methanosarcina_thermophila_TM-1:87:888213-888306 Satlength=94 Nr of Repeats=5 RepeatLength=19 seed=TTTATCCTGA Num.seqs=3 Similarity=0.953216 tpcCG=41.099998 7 GGATAAAAGAATATATTCA

****** ** *** ****

Consensus:

GGATAAaAGaATAtaTTCA

>arqueas_representative_Fam_349_19_2 Nr. of seq. 2 Alignment length(with gaps) = 19 Alignment score = 0.640351

arqueas_representative:NC_003552.1_Methanosarcina_acetivorans_str._C2A:80:4834836-4834909 Satlength=74 Nr of Repeats=5 RepeatLength=17 seed=TTCATTTTAT Num.seqs=3 Similarity=0.607843 tpcCG=42.700001 0 TTCATTTTATCTTNA-TC-

arqueas_representative:NZ_CP009517.1_Methanosarcina_barkeri_3:81:1262458-1262574 Satlength=117 Nr of Repeats=6 RepeatLength=19 seed=TTTTACCTTA Num.seqs=4 Similarity=0.762500 tpcCG=39.099998 4 ATCATTTTACCTTAATTCT

******** *** * **

Consensus:

aTCATTTTAcCTTaAtTCt

>arqueas_representative_Fam_350_19_2 Nr. of seq. 2 Alignment length(with gaps) = 19 Alignment score = 0.631579

arqueas_representative:NC_003552.1_Methanosarcina_acetivorans_str._C2A:80:5029598-5029666 Satlength=69 Nr of Repeats=4 RepeatLength=17 seed=TTGAAAGAAA Num.seqs=4 Similarity=1.000000 tpcCG=42.700001 0 TTGAAAGAAAGCTCATT--

Rev.of_arqueas_representative:NZ_CP009528.1_Methanosarcina_barkeri_MS:82:3727415-3727523 Satlength=109 Nr of Repeats=6 RepeatLength=18 seed=TTCAAGCAAT Num.seqs=6 Similarity=0.787654 tpcCG=39.168999 5 TTGAAAGGAATC-CATTGC

******* ** * ****

Consensus:

TTGAAAGaAAgCtCATTgc

>arqueas_representative_Fam_351_19_2 Nr. of seq. 2 Alignment length(with gaps) = 19 Alignment score = 0.622807

arqueas_representative:NZ_CP009517.1_Methanosarcina_barkeri_3:81:1019275-1019605 Satlength=331 Nr of Repeats=14 RepeatLength=17 seed=TTTTAGATTA Num.seqs=13 Similarity=1.000000 tpcCG=39.099998 0 TTTTAGATTACA-GTTTA-

arqueas_representative:NZ_CP009506.1_Methanosarcina_siciliae_T4/M:86:1752839-1752934 Satlength=96 Nr of Repeats=5 RepeatLength=19 seed=TTCAGATTAC Num.seqs=5 Similarity=1.000000 tpcCG=42.900002 1 TTTCAGATTACACGATAAC

*** ******** * * *

Consensus:

TTTcAGATTACAcGaTaAc

>arqueas_representative_Fam_352_19_2 Nr. of seq. 2 Alignment length(with gaps) = 19 Alignment score = 0.692982

arqueas_representative:NZ_CP009528.1_Methanosarcina_barkeri_MS:82:559220-559390 Satlength=171 Nr of Repeats=10 RepeatLength=17 seed=ATCCATTTTT Num.seqs=10 Similarity=0.857081 tpcCG=39.168999 0 ATC-CATTTTTGCAATTC-

Rev.of_arqueas_representative:NZ_CP009528.1_Methanosarcina_barkeri_MS:82:3900211-3900344 Satlength=134 Nr of Repeats=7 RepeatLength=19 seed=AAAAATGAGA Num.seqs=7 Similarity=1.000000 tpcCG=39.168999 11 TTCTCATTTTTGTAATTCT

** ******** *****

Consensus:

aTCtCATTTTTGcAATTCt

>arqueas_representative_Fam_353_19_2 Nr. of seq. 2 Alignment length(with gaps) = 19 Alignment score = 0.692982

arqueas_representative:NZ_CP009528.1_Methanosarcina_barkeri_MS:82:1321572-1321810 Satlength=239 Nr of Repeats=14 RepeatLength=17 seed=TAATTAAAAT Num.seqs=14 Similarity=0.792944 tpcCG=39.168999 0 TAATTAAAAT-AACCTAC-

arqueas_representative:NC_013790.1_Methanobrevibacter_ruminantium_M1:50:2422116-2422221 Satlength=106 Nr of Repeats=5 RepeatLength=19 seed=ATTAAAATAA Num.seqs=4 Similarity=0.871345 tpcCG=32.599998 2 TAATTAAAATAAAGCTAAA

********** ** ***

Consensus:

TAATTAAAATaAAcCTAaa

>arqueas_representative_Fam_354_19_2 Nr. of seq. 2 Alignment length(with gaps) = 19 Alignment score = 0.692982

arqueas_representative:NZ_CP009515.1_Methanosarcina_lacustris_Z-7289:84:3180856-3180924 Satlength=69 Nr of Repeats=4 RepeatLength=17 seed=TTGAAGAATT Num.seqs=4 Similarity=0.895425 tpcCG=41.799999 11 AATTAA-TTAAA-TTGAAG

arqueas_representative:NZ_CP009517.1_Methanosarcina_barkeri_3:81:2682572-2682685 Satlength=114 Nr of Repeats=6 RepeatLength=19 seed=AAATTAACTT Num.seqs=5 Similarity=0.894737 tpcCG=39.099998 16 AATTAACTTAAAATTCAAA

** ** ****** *****

Consensus:

TTcAAaAATTAAcTTAAAa

>arqueas_representative_Fam_355_19_2 Nr. of seq. 2 Alignment length(with gaps) = 19 Alignment score = 0.649123

arqueas_representative:NZ_CP009512.1_Methanosarcina_mazei_S-6:85:501722-501790 Satlength=69 Nr of Repeats=4 RepeatLength=17 seed=AAATGTATAA Num.seqs=4 Similarity=0.960784 tpcCG=41.400002 0 AAATGT-ATAATCT-TTTA

Rev.of_arqueas_representative:NZ_CP009528.1_Methanosarcina_barkeri_MS:82:3159252-3159347 Satlength=96 Nr of Repeats=5 RepeatLength=19 seed=AAATTATTAC Num.seqs=5 Similarity=0.831579 tpcCG=39.168999 14 AAAGGTAATAATTTATTTA

*** ** ***** * ****

Consensus:

AAAgGTaATAATcTaTTTA

>arqueas_representative_Fam_356_19_2 Nr. of seq. 2 Alignment length(with gaps) = 19 Alignment score = 0.666667

arqueas_representative:NZ_CP009506.1_Methanosarcina_siciliae_T4/M:86:4947461-4947529 Satlength=69 Nr of Repeats=4 RepeatLength=17 seed=TCTTTTTCTT Num.seqs=4 Similarity=1.000000 tpcCG=42.900002 0 TCTTTTTCTTATGCTTA--

Rev.of_arqueas_representative:NZ_CP009515.1_Methanosarcina_lacustris_Z-7289:84:2769430-2769507 Satlength=78 Nr of Repeats=4 RepeatLength=19 seed=AAATAAGCTC Num.seqs=3 Similarity=0.953216 tpcCG=41.7999 1 TCTCTTTCTTGAGCTTATT

*** ****** *****

Consensus:

TCTcTTTCTTaaGCTTAtt

>arqueas_representative_Fam_357_19_2 Nr. of seq. 2 Alignment length(with gaps) = 19 Alignment score = 0.614035

arqueas_representative:NZ_CP009520.1_Methanosarcina_vacuolata_Z-761:88:1474004-1474072 Satlength=69 Nr of Repeats=4 RepeatLength=17 seed=ATTCAGGTTA Num.seqs=4 Similarity=0.921569 tpcCG=39.732498 0 ATTCAGG--TTAATTGTTG

Rev.of_arqueas_representative:NC_015574.1_Methanobacterium_paludis__SWAN1:47:1187479-1187574 Satlength=96 Nr of Repeats=5 RepeatLength=19 seed=CCTGAATGAG Num.seqs=5 Similarity=1.000000 tpcCG=35.700001 7 ATTCAGGAGTTAATTACTC

******* ****** *

Consensus:

ATTCAGGagTTAATTacTc

>arqueas_representative_Fam_358_19_2 Nr. of seq. 2 Alignment length(with gaps) = 19 Alignment score = 0.622807

arqueas_representative:NC_017941.2_Haloferax_mediterranei_ATCC_33500:25:2052674-2053106 Satlength=433 Nr of Repeats=4 RepeatLength=18 seed=GCCCGACTCG Num.seqs=3 Similarity=0.901235 tpcCG=60.255100 0 GCCCGACTCGCCTGCTCC-

Rev.of_arqueas_representative:NC_020388.1_Natronomonas_moolapensis_8.8.11_:104:3956-4046 Satlength=91 Nr of Repeats=5 RepeatLength=18 seed=GACAGACGAG Num.seqs=5 Similarity=1.000000 tpcCG=64.500000 16 TCCCGTCTCGTCTG-TCCC

**** **** *** ***

Consensus:

gCCCGaCTCGcCTGcTCCc

>arqueas_representative_Fam_359_19_2 Nr. of seq. 2 Alignment length(with gaps) = 19 Alignment score = 0.614035

arqueas_representative:NC_012029.1_Halorubrum_lacusprofundi_ATCC_49239_chromosome_1:34:2158278-2158368 Satlength=91 Nr of Repeats=5 RepeatLength=18 seed=CCCCTCCGAC Num.seqs=5 Similarity=0.955556 tpcCG 0 CCCCTCCGACT-CTCCGCC

arqueas_representative:NZ_CP008874.1_Halanaeroarchaeum_sulfurireducens__HSR2:18:62579-62655 Satlength=77 Nr of Repeats=4 RepeatLength=19 seed=CCGCTCCACC Num.seqs=4 Similarity=1.000000 tpcCG=62.855801 0 CCGCTCCACCTACTCCACC

** **** ** **** **

Consensus:

CCcCTCCaaCTaCTCCaCC

>arqueas_representative_Fam_360_19_2 Nr. of seq. 2 Alignment length(with gaps) = 19 Alignment score = 0.684211

arqueas_representative:NC_015574.1_Methanobacterium_paludis__SWAN1:47:44732-44840 Satlength=109 Nr of Repeats=6 RepeatLength=18 seed=ATATCATCAA Num.seqs=6 Similarity=1.000000 tpcCG=35.700001 0 ATATCA-TCAATTGAAATT

Rev.of_arqueas_representative:NZ_CP009528.1_Methanosarcina_barkeri_MS:82:1416705-1418151 Satlength=1447 Nr of Repeats=11 RepeatLength=19 seed=AATTTACTGA Num.seqs=8 Similarity=1.000000 tpcCG=39.168999 13 TTATCAGTAAATTCAAATT

***** * **** *****

Consensus:

aTATCAgTaAATTcAAATT

>arqueas_representative_Fam_361_19_2 Nr. of seq. 2 Alignment length(with gaps) = 19 Alignment score = 0.622807

arqueas_representative:NC_003552.1_Methanosarcina_acetivorans_str._C2A:80:1918081-1918189 Satlength=109 Nr of Repeats=6 RepeatLength=18 seed=GCTGTGGAGT Num.seqs=6 Similarity=0.920988 tpcCG=42.700001 0 GCTG-TGGAGTTTCTTCTA

arqueas_representative:NZ_CP009512.1_Methanosarcina_mazei_S-6:85:1865615-1865903 Satlength=289 Nr of Repeats=9 RepeatLength=18 seed=CTTCTTCTGT Num.seqs=6 Similarity=0.960494 tpcCG=41.400002 12 -CTGTTGGTGTTACTTCTT

*** *** *** *****

Consensus:

gCTGtTGGaGTTaCTTCTa

>arqueas_representative_Fam_362_19_2 Nr. of seq. 2 Alignment length(with gaps) = 19 Alignment score = 0.692982

arqueas_representative:NZ_CP009517.1_Methanosarcina_barkeri_3:81:4004152-4004314 Satlength=163 Nr of Repeats=9 RepeatLength=18 seed=AGGAGTAGAA Num.seqs=9 Similarity=0.971193 tpcCG=39.099998 20 AG-AGGAGTAGAAATAATA

arqueas_representative:NZ_CP009528.1_Methanosarcina_barkeri_MS:82:3460084-3460390 Satlength=307 Nr of Repeats=17 RepeatLength=18 seed=AAGTAATAGA Num.seqs=17 Similarity=1.000000 tpcCG=39.168999 20 AGAAGTAATAGAAAT-ATA

** * ******* *****

Consensus:

aAGgAaTAGAAATaATAAG

>arqueas_representative_Fam_363_19_2 Nr. of seq. 2 Alignment length(with gaps) = 19 Alignment score = 0.614035

arqueas_representative:NZ_CP009517.1_Methanosarcina_barkeri_3:81:4477065-4477299 Satlength=235 Nr of Repeats=13 RepeatLength=18 seed=TAATGCAGTA Num.seqs=13 Similarity=0.899335 tpcCG=39.099998 0 TAA-TGCAGTAACTTGTAC

Rev.of_arqueas_representative:NZ_CP009520.1_Methanosarcina_vacuolata_Z-761:88:1919650-1919746 Satlength=97 Nr of Repeats=5 RepeatLength=19 seed=ATTCAGGTTG Num.seqs=4 Similarity=0.964912 tpcCG=39.73249 0 TAAGTGCAGCAACCTGAAT

*** ***** *** ** *

Consensus:

TAAgTGCAGcAACcTGaAc

>arqueas_representative_Fam_364_19_2 Nr. of seq. 2 Alignment length(with gaps) = 19 Alignment score = 0.649123

arqueas_representative:NZ_CP009517.1_Methanosarcina_barkeri_3:81:4495975-4496083 Satlength=109 Nr of Repeats=6 RepeatLength=18 seed=CGAGTTTTCT Num.seqs=6 Similarity=1.000000 tpcCG=39.099998 0 CGAGTTTTCTCA-ACTTAA

arqueas_representative:NZ_CP009528.1_Methanosarcina_barkeri_MS:82:4236373-4236463 Satlength=91 Nr of Repeats=5 RepeatLength=18 seed=AATTTCACAC Num.seqs=5 Similarity=1.000000 tpcCG=39.168999 2 CGA-ATTTCACACACTTAA

*** **** ** ******

Consensus:

CGAgaTTTCaCAcACTTAA

>arqueas_representative_Fam_365_19_2 Nr. of seq. 2 Alignment length(with gaps) = 19 Alignment score = 0.719298

arqueas_representative:NZ_CP009528.1_Methanosarcina_barkeri_MS:82:1949995-1950103 Satlength=109 Nr of Repeats=6 RepeatLength=18 seed=TAGTTAAATA Num.seqs=6 Similarity=0.955556 tpcCG=39.168999 0 T-AGTTAAATAATATTAAG

arqueas_representative:NZ_CP009528.1_Methanosarcina_barkeri_MS:82:2811504-2811593 Satlength=90 Nr of Repeats=5 RepeatLength=18 seed=TAAGTTCAAT Num.seqs=4 Similarity=1.000000 tpcCG=39.168999 0 TAAGTTCAATAA-ATTAAG

* **** ***** ******

Consensus:

TaAGTTaAATAAtATTAAG

>arqueas_representative_Fam_366_19_2 Nr. of seq. 2 Alignment length(with gaps) = 19 Alignment score = 0.649123

arqueas_representative:NZ_CP009528.1_Methanosarcina_barkeri_MS:82:2221930-2222020 Satlength=91 Nr of Repeats=5 RepeatLength=18 seed=ATTCCAATAA Num.seqs=5 Similarity=0.940741 tpcCG=39.168999 0 ATTCCAATAACTCCA-ATG

Rev.of_arqueas_representative:NZ_CP009528.1_Methanosarcina_barkeri_MS:82:4498649-4498748 Satlength=100 Nr of Repeats=6 RepeatLength=18 seed=ATCTGGAGTA Num.seqs=4 Similarity=0.851852 tpcCG=39.168999 17 CTTCAAAT-ACTCCAGATG

*** *** ****** ***

Consensus:

aTTCaAATaACTCCAgATG

>arqueas_representative_Fam_367_19_2 Nr. of seq. 2 Alignment length(with gaps) = 19 Alignment score = 0.614035

arqueas_representative:NZ_CP009528.1_Methanosarcina_barkeri_MS:82:3051433-3051505 Satlength=73 Nr of Repeats=4 RepeatLength=18 seed=GCTGAAAACA Num.seqs=4 Similarity=0.962963 tpcCG=39.168999 0 GCTGA-AAACATTGAAAAT

arqueas_representative:NZ_CP009512.1_Methanosarcina_mazei_S-6:85:3450857-3451029 Satlength=173 Nr of Repeats=9 RepeatLength=19 seed=ACATTGAGAA Num.seqs=8 Similarity=1.000000 tpcCG=41.400002 8 ATTGAGAGACATTGAGAAT

*** * ******* ***

Consensus:

acTGAgAaACATTGAaAAT

>arqueas_representative_Fam_368_19_2 Nr. of seq. 2 Alignment length(with gaps) = 19 Alignment score = 0.684211

arqueas_representative:NZ_CP009528.1_Methanosarcina_barkeri_MS:82:3822533-3823160 Satlength=628 Nr of Repeats=7 RepeatLength=18 seed=AAAAATGGGG Num.seqs=5 Similarity=1.000000 tpcCG=39.168999 0 AAAAATGGGGAA-CATGAC

arqueas_representative:NZ_CP009520.1_Methanosarcina_vacuolata_Z-761:88:1146649-1146742 Satlength=94 Nr of Repeats=5 RepeatLength=19 seed=AAAAATGGGA Num.seqs=4 Similarity=1.000000 tpcCG=39.732498 0 AAAAATGGGAAATCAAGAG

********* ** ** **

Consensus:

AAAAATGGGaAAtCAaGAc

>arqueas_representative_Fam_369_19_2 Nr. of seq. 2 Alignment length(with gaps) = 19 Alignment score = 0.684211

arqueas_representative:NZ_CP009515.1_Methanosarcina_lacustris_Z-7289:84:303106-303178 Satlength=73 Nr of Repeats=4 RepeatLength=18 seed=TCTGAAAACA Num.seqs=4 Similarity=1.000000 tpcCG=41.799999 1 GTCTGAAAACA-GAAAATA

Rev.of_arqueas_representative:NZ_CP009501.1_Methanosarcina_thermophila_TM-1:87:783671-783747 Satlength=77 Nr of Repeats=4 RepeatLength=19 seed=TATTTTCTCA Num.seqs=4 Similarity=0.894737 tpcCG=41.099998 11 GTGAGAAAATAGGAAAATA

* ***** * ********

Consensus:

TcaGAAAAcAgGAAAATAG

>arqueas_representative_Fam_370_19_2 Nr. of seq. 2 Alignment length(with gaps) = 19 Alignment score = 0.684211

arqueas_representative:NZ_CP009512.1_Methanosarcina_mazei_S-6:85:3307146-3307254 Satlength=109 Nr of Repeats=6 RepeatLength=18 seed=GATTAAGTAA Num.seqs=6 Similarity=0.871605 tpcCG=41.400002 0 GATTAAGTAAA-GTTGATT

Rev.of_arqueas_representative:NZ_CP009517.1_Methanosarcina_barkeri_3:81:4475208-4475284 Satlength=77 Nr of Repeats=4 RepeatLength=19 seed=TAAGTAATCA Num.seqs=4 Similarity=0.964912 tpcCG=39.099998 9 GATTACTTAAATCTTGATT

***** **** ******

Consensus:

GATTAagTAAAtcTTGATT

>arqueas_representative_Fam_371_19_2 Nr. of seq. 2 Alignment length(with gaps) = 19 Alignment score = 0.614035

arqueas_representative:NZ_CP009512.1_Methanosarcina_mazei_S-6:85:3931199-3931407 Satlength=209 Nr of Repeats=10 RepeatLength=18 seed=TATTGTTACA Num.seqs=9 Similarity=0.983539 tpcCG=41.400002 0 TATTGTTACATTATGT-AT

arqueas_representative:NC_003552.1_Methanosarcina_acetivorans_str._C2A:80:4998928-4999042 Satlength=115 Nr of Repeats=6 RepeatLength=19 seed=TATCCTTATT Num.seqs=6 Similarity=0.915789 tpcCG=42.700001 13 TATTGGCACATTATATCCT

***** ******* * *

Consensus:

TATTGgcACATTATaTcaT

>arqueas_representative_Fam_372_19_2 Nr. of seq. 2 Alignment length(with gaps) = 19 Alignment score = 0.684211

arqueas_representative:NZ_CP009501.1_Methanosarcina_thermophila_TM-1:87:2421554-2421626 Satlength=73 Nr of Repeats=4 RepeatLength=18 seed=ATTTCTCAAA Num.seqs=4 Similarity=0.962963 tpcCG=41.099998 0 ATTTCTCAAACTATT-TAT

arqueas_representative:NZ_CP009512.1_Methanosarcina_mazei_S-6:85:1513803-1513899 Satlength=97 Nr of Repeats=6 RepeatLength=19 seed=ATTATTATAT Num.seqs=4 Similarity=1.000000 tpcCG=41.400002 9 ATTTCACATATTATTATAT

***** ** * **** ***

Consensus:

ATTTCaCAaAcTATTaTAT

>arqueas_representative_Fam_373_19_2 Nr. of seq. 2 Alignment length(with gaps) = 19 Alignment score = 0.614035

arqueas_representative:NZ_CP009520.1_Methanosarcina_vacuolata_Z-761:88:2105558-2105711 Satlength=154 Nr of Repeats=8 RepeatLength=18 seed=AGAAAACACA Num.seqs=7 Similarity=1.000000 tpcCG=39.732498 0 AGAAAACACAG-GAAACGG

arqueas_representative:NC_014729.1_Halogeometricum_borinquense_DSM_11551:27:2379543-2379638 Satlength=96 Nr of Repeats=4 RepeatLength=19 seed=CAAACGCAGA Num.seqs=3 Similarity=1.000000 tpcCG=59.970901 2 AGCAAACGCAGAGAGATGG

** **** *** ** * **

Consensus:

AGaAAACaCAGaGAaAcGG

>arqueas_representative_Fam_374_19_2 Nr. of seq. 2 Alignment length(with gaps) = 19 Alignment score = 0.614035

arqueas_representative:NZ_CP009520.1_Methanosarcina_vacuolata_Z-761:88:4429707-4429796 Satlength=90 Nr of Repeats=5 RepeatLength=18 seed=TGCTGATAAT Num.seqs=4 Similarity=1.000000 tpcCG=39.732498 0 TGCTGATA-ATTATCCATG

Rev.of_arqueas_representative:NZ_CP009517.1_Methanosarcina_barkeri_3:81:329660-329812 Satlength=153 Nr of Repeats=8 RepeatLength=19 seed=AACTTGGATA Num.seqs=8 Similarity=1.000000 tpcCG=39.099998 2 TTCTCATATTTTATCCAAG

* ** *** ******* *

Consensus:

TgCTcATAtaTTATCCAaG

>arqueas_representative_Fam_375_19_2 Nr. of seq. 2 Alignment length(with gaps) = 19 Alignment score = 0.789474

arqueas_representative:NC_013790.1_Methanobrevibacter_ruminantium_M1:50:2102487-2102755 Satlength=269 Nr of Repeats=5 RepeatLength=19 seed=TTTAAAACTT Num.seqs=3 Similarity=0.906433 tpcCG=32.599998 0 TTTAAAACTTTCTAAAGTG

Rev.of_arqueas_representative:NC_013790.1_Methanobrevibacter_ruminantium_M1:50:2148093-2148264 Satlength=172 Nr of Repeats=9 RepeatLength=19 seed=AAAGTTTTAA Num.seqs=9 Similarity=0.817593 tpcCG=32.599 11 TTTAAAACTTTCTTATGAG

************* * * *

Consensus:

TTTAAAACTTTCTaAaGaG

>arqueas_representative_Fam_376_19_2 Nr. of seq. 2 Alignment length(with gaps) = 19 Alignment score = 0.929825

arqueas_representative:NZ_CP009517.1_Methanosarcina_barkeri_3:81:1868862-1868972 Satlength=111 Nr of Repeats=6 RepeatLength=19 seed=AGTAATTTAG Num.seqs=5 Similarity=1.000000 tpcCG=39.099998 0 AGTAATTTAGCTATCAGTA

arqueas_representative:NZ_CP009528.1_Methanosarcina_barkeri_MS:82:1384391-1384486 Satlength=96 Nr of Repeats=5 RepeatLength=19 seed=GTAATTTAGC Num.seqs=5 Similarity=0.915789 tpcCG=39.168999 1 AGTAATTTAGCTATCAGTG

******************

Consensus:

AGTAATTTAGCTATCAGTa

>arqueas_representative_Fam_377_19_2 Nr. of seq. 2 Alignment length(with gaps) = 19 Alignment score = 0.649123

arqueas_representative:NZ_CP009515.1_Methanosarcina_lacustris_Z-7289:84:910562-910695 Satlength=134 Nr of Repeats=7 RepeatLength=19 seed=TTTGCGTTGA Num.seqs=7 Similarity=0.959900 tpcCG=41.799999 0 TTTGCGTTGATTTTACATA

arqueas_representative:NZ_CP009506.1_Methanosarcina_siciliae_T4/M:86:1371432-1371508 Satlength=77 Nr of Repeats=4 RepeatLength=19 seed=TTTCTTATTT Num.seqs=4 Similarity=1.000000 tpcCG=42.900002 12 TTTGCCTTTACTTTTCTTA

***** ** * *** * **

Consensus:

TTTGCcTTgAcTTTaCaTA

>arqueas_representative_Fam_378_19_2 Nr. of seq. 2 Alignment length(with gaps) = 19 Alignment score = 0.649123

arqueas_representative:NZ_CP009515.1_Methanosarcina_lacustris_Z-7289:84:2164951-2165027 Satlength=77 Nr of Repeats=4 RepeatLength=19 seed=TTGATGAATA Num.seqs=4 Similarity=0.894737 tpcCG=41.799999 0 TTGATGAATATCTCTAAAT

arqueas_representative:NZ_CP009501.1_Methanosarcina_thermophila_TM-1:87:2310974-2311088 Satlength=115 Nr of Repeats=5 RepeatLength=19 seed=CTGTAAATTT Num.seqs=4 Similarity=1.000000 tpcCG=41.099998 11 TTGTCTAAAATCTGTAAAT

*** ** **** *****

Consensus:

TTGacgAAaATCTcTAAAT

>arqueas_representative_Fam_379_18_2 Nr. of seq. 2 Alignment length(with gaps) = 18 Alignment score = 0.638889

arqueas_representative:NC_019977.1_Methanomethylovorans_hollandica_DSM_15978:75:213680-213818 Satlength=139 Nr of Repeats=5 RepeatLength=15 seed=AACGAGACTC Num.seqs=3 Similarity=0.940741 tpcCG=41.8436 0 AACGAGA-CTCCAG-GC-

Rev.of_arqueas_representative:NC_013743.1_Haloterrigena_turkmenica_DSM_5511:38:750188-750560 Satlength=373 Nr of Repeats=4 RepeatLength=18 seed=TCTCGTTGGC Num.seqs=3 Similarity=0.950617 tpcCG=64.24839 7 AACGAGAGCGCCAGCGCC

******* * **** **

Consensus:

AACGAGAgCgCCAGcGCc

>arqueas_representative_Fam_380_18_2 Nr. of seq. 2 Alignment length(with gaps) = 18 Alignment score = 0.638889

arqueas_representative:NZ_CP009515.1_Methanosarcina_lacustris_Z-7289:84:2245237-2245327 Satlength=91 Nr of Repeats=6 RepeatLength=15 seed=AATTGTATTG Num.seqs=6 Similarity=1.000000 tpcCG=41.799999 0 AATTGT--ATTGTGATT-

Rev.of_arqueas_representative:NC_003552.1_Methanosarcina_acetivorans_str._C2A:80:3694740-3694859 Satlength=120 Nr of Repeats=7 RepeatLength=17 seed=TACAATTGAA Num.seqs=7 Similarity=1.000000 tpcCG=42.7 7 AATTGTAAATT-TGATTC

****** *** *****

Consensus:

AATTGTaaATTgTGATTc

>arqueas_representative_Fam_381_18_2 Nr. of seq. 2 Alignment length(with gaps) = 18 Alignment score = 0.731481

arqueas_representative:NZ_CP009515.1_Methanosarcina_lacustris_Z-7289:84:2739921-2740011 Satlength=91 Nr of Repeats=6 RepeatLength=15 seed=ATTATCCACT Num.seqs=6 Similarity=0.970370 tpcCG=41.799999 2 AT---ATTATCCACTGAA

arqueas_representative:NZ_CP009506.1_Methanosarcina_siciliae_T4/M:86:837682-837754 Satlength=73 Nr of Repeats=4 RepeatLength=18 seed=TCTGAAATAT Num.seqs=4 Similarity=0.925926 tpcCG=42.900002 9 ATATCATTATCCTCTGAA

******* *******

Consensus:

ATTATCCaCTGAAATatc

>arqueas_representative_Fam_382_18_2 Nr. of seq. 2 Alignment length(with gaps) = 18 Alignment score = 0.685185

arqueas_representative:NZ_CP009520.1_Methanosarcina_vacuolata_Z-761:88:3618173-3618293 Satlength=121 Nr of Repeats=5 RepeatLength=15 seed=TTCTTACTTT Num.seqs=4 Similarity=0.837037 tpcCG=39.732498 0 TTCT-TACTTTAGGGA--

Rev.of_arqueas_representative:NZ_CP009515.1_Methanosarcina_lacustris_Z-7289:84:2645944-2646016 Satlength=73 Nr of Repeats=4 RepeatLength=18 seed=AACATCCCTA Num.seqs=4 Similarity=0.962963 tpcCG=41.7999 2 TTCTGTAATTTAGGGATG

**** ** ********

Consensus:

TTCTgTAaTTTAGGGAtg

>arqueas_representative_Fam_383_18_2 Nr. of seq. 2 Alignment length(with gaps) = 18 Alignment score = 0.638889

arqueas_representative:NZ_CP009517.1_Methanosarcina_barkeri_3:81:711132-711244 Satlength=113 Nr of Repeats=7 RepeatLength=16 seed=GAACTGTTAA Num.seqs=7 Similarity=0.833333 tpcCG=39.099998 0 GAACTGTTAAAGCA-AA-

Rev.of_arqueas_representative:NC_003552.1_Methanosarcina_acetivorans_str._C2A:80:2029870-2030023 Satlength=154 Nr of Repeats=9 RepeatLength=17 seed=TATGCTCAAC Num.seqs=9 Similarity=1.000000 tpcCG=42.7 15 GAACTGTT-GAGCATAAC

******** **** **

Consensus:

GAACTGTTaaAGCAtAAc

>arqueas_representative_Fam_384_18_2 Nr. of seq. 2 Alignment length(with gaps) = 18 Alignment score = 0.611111

arqueas_representative:NZ_CP009517.1_Methanosarcina_barkeri_3:81:4039906-4039986 Satlength=81 Nr of Repeats=5 RepeatLength=16 seed=TATCCACAAT Num.seqs=5 Similarity=0.966667 tpcCG=39.099998 0 TATCCACAATCGAAAC--

arqueas_representative:NZ_CP009517.1_Methanosarcina_barkeri_3:81:3824228-3824313 Satlength=86 Nr of Repeats=5 RepeatLength=17 seed=ATAGAAACTA Num.seqs=5 Similarity=0.937255 tpcCG=39.099998 7 AATCCA-AATAGAAACTA

***** *** *****

Consensus:

aATCCAcAATaGAAACta

>arqueas_representative_Fam_385_18_2 Nr. of seq. 2 Alignment length(with gaps) = 18 Alignment score = 0.694444

arqueas_representative:NZ_CP009515.1_Methanosarcina_lacustris_Z-7289:84:1100467-1100652 Satlength=186 Nr of Repeats=11 RepeatLength=16 seed=ATTAACAGAT Num.seqs=8 Similarity=0.955357 tpcCG=41.799999 0 ATTAACAGATCAT-AAA-

arqueas_representative:NZ_CP009515.1_Methanosarcina_lacustris_Z-7289:84:11372-11480 Satlength=109 Nr of Repeats=6 RepeatLength=18 seed=GAAATATTAA Num.seqs=6 Similarity=0.940741 tpcCG=41.799999 13 ATTAAAAGATCANGAAAT

***** ****** ***

Consensus:

ATTAAaAGATCAtgAAAt

>arqueas_representative_Fam_386_18_2 Nr. of seq. 2 Alignment length(with gaps) = 18 Alignment score = 0.675926

arqueas_representative:NZ_CP009512.1_Methanosarcina_mazei_S-6:85:3879760-3879840 Satlength=81 Nr of Repeats=5 RepeatLength=16 seed=TTTCTCAGCT Num.seqs=5 Similarity=1.000000 tpcCG=41.400002 0 TTTC-TCAGCTGTTTAC-

Rev.of_arqueas_representative:NZ_CP009506.1_Methanosarcina_siciliae_T4/M:86:1901755-1901827 Satlength=73 Nr of Repeats=4 RepeatLength=18 seed=GAAGAAACGG Num.seqs=4 Similarity=1.000000 tpcCG=42.900002 7 TTTCTTCAGATGTTTCCG

**** **** ***** *

Consensus:

TTTCtTCAGaTGTTTaCg

>arqueas_representative_Fam_387_18_2 Nr. of seq. 2 Alignment length(with gaps) = 18 Alignment score = 0.648148

arqueas_representative:NZ_CP009520.1_Methanosarcina_vacuolata_Z-761:88:317819-317883 Satlength=65 Nr of Repeats=4 RepeatLength=16 seed=AAAACCTTAG Num.seqs=4 Similarity=0.958333 tpcCG=39.732498 0 AAAACCTTAGAGAATG--

Rev.of_arqueas_representative:NZ_CP009517.1_Methanosarcina_barkeri_3:81:562305-562391 Satlength=87 Nr of Repeats=5 RepeatLength=18 seed=TTGTTTTGAC Num.seqs=3 Similarity=1.000000 tpcCG=39.099998 7 AAAACAATAGACAATGTC

***** **** ****

Consensus:

AAAACaaTAGAcAATGtc

>arqueas_representative_Fam_388_18_2 Nr. of seq. 2 Alignment length(with gaps) = 18 Alignment score = 0.611111

arqueas_representative:NZ_CP009520.1_Methanosarcina_vacuolata_Z-761:88:1361309-1361435 Satlength=127 Nr of Repeats=7 RepeatLength=16 seed=TATGAAAAGT Num.seqs=5 Similarity=0.827451 tpcCG=39.732498 0 TATGAAAAGTAGGACT--

Rev.of_arqueas_representative:NZ_CP009528.1_Methanosarcina_barkeri_MS:82:835804-835872 Satlength=69 Nr of Repeats=4 RepeatLength=17 seed=ATCCATTTTT Num.seqs=4 Similarity=1.000000 tpcCG=39.168999 14 TATGAAAAAT-GGATTTG

******** * *** *

Consensus:

TATGAAAAaTaGGAcTtg

>arqueas_representative_Fam_389_18_2 Nr. of seq. 2 Alignment length(with gaps) = 18 Alignment score = 0.629630

arqueas_representative:NZ_CP009520.1_Methanosarcina_vacuolata_Z-761:88:1869506-1869618 Satlength=113 Nr of Repeats=7 RepeatLength=16 seed=CAGTTAGAGT Num.seqs=7 Similarity=0.936508 tpcCG=39.732498 0 CAGTTAGA-G-TTAGGAT

Rev.of_arqueas_representative:NZ_CP009520.1_Methanosarcina_vacuolata_Z-761:88:3335404-3335512 Satlength=109 Nr of Repeats=6 RepeatLength=18 seed=TCATCCTCTA Num.seqs=6 Similarity=1.000000 tpcCG=39.7324 14 AAGTTAGAGGATGAGGAT

******* * * *****

Consensus:

aAGTTAGAgGaTgAGGAT

>arqueas_representative_Fam_390_18_2 Nr. of seq. 2 Alignment length(with gaps) = 18 Alignment score = 0.629630

arqueas_representative:NZ_CP009520.1_Methanosarcina_vacuolata_Z-761:88:2724298-2724410 Satlength=113 Nr of Repeats=7 RepeatLength=16 seed=ATATAATGTA Num.seqs=7 Similarity=1.000000 tpcCG=39.732498 0 A-TATAATGTACTCA-TA

arqueas_representative:NZ_CP009515.1_Methanosarcina_lacustris_Z-7289:84:1605361-1605469 Satlength=109 Nr of Repeats=6 RepeatLength=18 seed=ATTATGATGA Num.seqs=6 Similarity=0.881481 tpcCG=41.799999 0 ATTATGATGAACTCACTA

* *** *** ***** **

Consensus:

AtTATaATGaACTCAcTA

>arqueas_representative_Fam_391_18_2 Nr. of seq. 2 Alignment length(with gaps) = 18 Alignment score = 0.601852

arqueas_representative:NC_013922.1_Natrialba_magadii_ATCC_43099:100:1529535-1529599 Satlength=65 Nr of Repeats=4 RepeatLength=16 seed=AACTACGGAA Num.seqs=4 Similarity=1.000000 tpcCG=61.032001 0 AACTA-CGGAACTACGG-

Rev.of_arqueas_representative:NZ_CP009528.1_Methanosarcina_barkeri_MS:82:620008-620089 Satlength=82 Nr of Repeats=5 RepeatLength=18 seed=TACTTTCCGT Num.seqs=3 Similarity=1.000000 tpcCG=39.168999 5 AAGTATGGGAAGTACGGA

** ** **** *****

Consensus:

AAcTAtcGGAAcTACGGa

>arqueas_representative_Fam_392_18_2 Nr. of seq. 2 Alignment length(with gaps) = 18 Alignment score = 0.638889

arqueas_representative:NC_003552.1_Methanosarcina_acetivorans_str._C2A:80:2970646-2970749 Satlength=104 Nr of Repeats=6 RepeatLength=17 seed=GAGTTTTCTG Num.seqs=5 Similarity=1.000000 tpcCG=42.700001 0 GAGTTTTCTGGAAATAT-

arqueas_representative:NZ_CP009517.1_Methanosarcina_barkeri_3:81:243659-243731 Satlength=73 Nr of Repeats=4 RepeatLength=18 seed=AAAATACATA Num.seqs=4 Similarity=0.962963 tpcCG=39.099998 12 CATATTTCTGGAAAAATA

* ********** **

Consensus:

cAgaTTTCTGGAAAaATa

>arqueas_representative_Fam_393_18_2 Nr. of seq. 2 Alignment length(with gaps) = 18 Alignment score = 0.666667

arqueas_representative:NC_003552.1_Methanosarcina_acetivorans_str._C2A:80:5025628-5025956 Satlength=329 Nr of Repeats=5 RepeatLength=17 seed=TTTGACTTTA Num.seqs=4 Similarity=0.960784 tpcCG=42.700001 0 TTTGACTTTACTCTG-CC

Rev.of_arqueas_representative:NZ_CP009528.1_Methanosarcina_barkeri_MS:82:900890-901016 Satlength=127 Nr of Repeats=7 RepeatLength=18 seed=AATCTGAAAG Num.seqs=7 Similarity=1.000000 tpcCG=39.168999 9 TTTCAGATTACTCTGTCC

*** * ******** **

Consensus:

TTTcAcaTTACTCTGtCC

>arqueas_representative_Fam_394_18_2 Nr. of seq. 2 Alignment length(with gaps) = 18 Alignment score = 0.675926

arqueas_representative:NZ_CP009517.1_Methanosarcina_barkeri_3:81:1041754-1041907 Satlength=154 Nr of Repeats=9 RepeatLength=17 seed=TAGAGACAAT Num.seqs=9 Similarity=0.864924 tpcCG=39.099998 0 TAGAGACAATACTAAAT-

Rev.of_arqueas_representative:NZ_CP009512.1_Methanosarcina_mazei_S-6:85:1951883-1952002 Satlength=120 Nr of Repeats=7 RepeatLength=17 seed=AAGTATTTTT Num.seqs=7 Similarity=0.977591 tpcCG=41.400002 13 TAGA-AAAATACTTAATT

**** * ****** ***

Consensus:

TAGAgAaAATACTaAATt

>arqueas_representative_Fam_395_18_2 Nr. of seq. 2 Alignment length(with gaps) = 18 Alignment score = 0.666667

arqueas_representative:NZ_CP009517.1_Methanosarcina_barkeri_3:81:3171819-3171887 Satlength=69 Nr of Repeats=4 RepeatLength=17 seed=TTCAATATAG Num.seqs=4 Similarity=0.947712 tpcCG=39.099998 0 TTC-AATATAGCAGATCC

arqueas_representative:NC_003552.1_Methanosarcina_acetivorans_str._C2A:80:3898088-3898197 Satlength=110 Nr of Repeats=6 RepeatLength=18 seed=GATCCTTCAA Num.seqs=5 Similarity=0.925926 tpcCG=42.700001 13 TTCAAACTTAACAGATCC

*** ** ** *******

Consensus:

TTCaAAcaTAaCAGATCC

>arqueas_representative_Fam_396_18_2 Nr. of seq. 2 Alignment length(with gaps) = 18 Alignment score = 0.601852

arqueas_representative:NZ_CP009517.1_Methanosarcina_barkeri_3:81:4024491-4024593 Satlength=103 Nr of Repeats=6 RepeatLength=17 seed=ACACTGACTA Num.seqs=6 Similarity=0.952941 tpcCG=39.099998 0 ACACTGACTATGCTTTA-

Rev.of_arqueas_representative:NZ_CP009520.1_Methanosarcina_vacuolata_Z-761:88:95393-95461 Satlength=69 Nr of Repeats=4 RepeatLength=17 seed=AAGTGTCTAA Num.seqs=4 Similarity=1.000000 tpcCG=39.732498 6 ACACTTAC-ATCATTTAG

***** ** ** ****

Consensus:

ACACTgACtATcaTTTAg

>arqueas_representative_Fam_397_18_2 Nr. of seq. 2 Alignment length(with gaps) = 18 Alignment score = 0.703704

arqueas_representative:NZ_CP009517.1_Methanosarcina_barkeri_3:81:4141729-4141814 Satlength=86 Nr of Repeats=5 RepeatLength=17 seed=TAGAAACTTG Num.seqs=5 Similarity=0.968627 tpcCG=39.099998 0 TAGAAACTTGA-TATAAT

arqueas_representative:NZ_CP009528.1_Methanosarcina_barkeri_MS:82:3355190-3355259 Satlength=70 Nr of Repeats=4 RepeatLength=17 seed=AATTAGAAAC Num.seqs=3 Similarity=0.895425 tpcCG=39.168999 14 TAGAAAC-TGAGTTTAAT

******* *** * ****

Consensus:

TAGAAACtTGAgTaTAAT

>arqueas_representative_Fam_398_18_2 Nr. of seq. 2 Alignment length(with gaps) = 18 Alignment score = 0.638889

arqueas_representative:NZ_CP009528.1_Methanosarcina_barkeri_MS:82:588999-589119 Satlength=121 Nr of Repeats=7 RepeatLength=17 seed=AATAATATTA Num.seqs=6 Similarity=1.000000 tpcCG=39.168999 0 AATAATATTACACTATT-

Rev.of_arqueas_representative:NC_003552.1_Methanosarcina_acetivorans_str._C2A:80:5115409-5115535 Satlength=127 Nr of Repeats=7 RepeatLength=18 seed=CTGAATACTG Num.seqs=7 Similarity=1.000000 tpcCG=42.7 2 AGTAACATTTCAGTATTC

* *** *** ** ****

Consensus:

AaTAAcATTaCAcTATTc

>arqueas_representative_Fam_399_18_2 Nr. of seq. 2 Alignment length(with gaps) = 18 Alignment score = 0.666667

arqueas_representative:NZ_CP009528.1_Methanosarcina_barkeri_MS:82:3306528-3306655 Satlength=128 Nr of Repeats=5 RepeatLength=17 seed=GACTATTTTT Num.seqs=4 Similarity=0.882353 tpcCG=39.168999 0 GACT-ATTTTTGGTTCTA

Rev.of_arqueas_representative:NC_003552.1_Methanosarcina_acetivorans_str._C2A:80:1177133-1177204 Satlength=72 Nr of Repeats=4 RepeatLength=18 seed=AATCAGTATA Num.seqs=3 Similarity=1.000000 tpcCG=42.70 8 TACTGATTTATAGTTCTA

*** **** * ******

Consensus:

gACTgATTTaTaGTTCTA

>arqueas_representative_Fam_400_18_2 Nr. of seq. 2 Alignment length(with gaps) = 18 Alignment score = 0.638889

arqueas_representative:NZ_CP009515.1_Methanosarcina_lacustris_Z-7289:84:1634559-1634644 Satlength=86 Nr of Repeats=5 RepeatLength=17 seed=GAATAGGGAT Num.seqs=5 Similarity=0.921569 tpcCG=41.799999 0 GAATAGGGATTGGATTC-

Rev.of_arqueas_representative:CP009516.1_Methanosarcina_horonobensis_HB-1:83:5006612-5006681 Satlength=70 Nr of Repeats=4 RepeatLength=18 seed=TTCACAATCC Num.seqs=3 Similarity=1.000000 tpcCG=41.299999 3 GAATAAAGAATGGATTGT

***** ** ******

Consensus:

GAATAaaGAaTGGATTct

>arqueas_representative_Fam_401_18_2 Nr. of seq. 2 Alignment length(with gaps) = 18 Alignment score = 0.666667

arqueas_representative:NZ_CP009512.1_Methanosarcina_mazei_S-6:85:4126716-4126802 Satlength=87 Nr of Repeats=5 RepeatLength=17 seed=AAGTAACATA Num.seqs=3 Similarity=0.783951 tpcCG=41.400002 0 AAGTAACATATT-CTTTA

Rev.of_arqueas_representative:NZ_CP009517.1_Methanosarcina_barkeri_3:81:2584343-2584505 Satlength=163 Nr of Repeats=9 RepeatLength=18 seed=ATATGTTAAT Num.seqs=9 Similarity=1.000000 tpcCG=39.099998 11 AATTAACATATTCCCTGA

** ********* * * *

Consensus:

AAgTAACATATTcCcTgA

>arqueas_representative_Fam_402_18_2 Nr. of seq. 2 Alignment length(with gaps) = 18 Alignment score = 0.638889

arqueas_representative:NZ_CP009506.1_Methanosarcina_siciliae_T4/M:86:129732-129817 Satlength=86 Nr of Repeats=5 RepeatLength=17 seed=GTTCTATACC Num.seqs=5 Similarity=1.000000 tpcCG=42.900002 0 GTTCTATACCATCTACC-

Rev.of_arqueas_representative:NZ_CP009515.1_Methanosarcina_lacustris_Z-7289:84:4138686-4138925 Satlength=240 Nr of Repeats=13 RepeatLength=18 seed=ATTTCATAGA Num.seqs=8 Similarity=1.000000 tpcCG=41.79 12 GTTCTATGAAATCTTCCT

******* **** **

Consensus:

GTTCTATaaaATCTaCCt

>arqueas_representative_Fam_403_18_2 Nr. of seq. 2 Alignment length(with gaps) = 18 Alignment score = 0.638889

arqueas_representative:NZ_CP009506.1_Methanosarcina_siciliae_T4/M:86:295560-295628 Satlength=69 Nr of Repeats=4 RepeatLength=17 seed=AGATGAGATT Num.seqs=4 Similarity=1.000000 tpcCG=42.900002 0 AGATGAGATTTGTCATA-

arqueas_representative:NZ_CP009506.1_Methanosarcina_siciliae_T4/M:86:2362508-2362616 Satlength=109 Nr of Repeats=6 RepeatLength=18 seed=TGATGAGTTT Num.seqs=6 Similarity=0.975309 tpcCG=42.900002 0 TGATGAGTTTTGTTATCC

****** ***** **

Consensus:

aGATGAGaTTTGTcATac

>arqueas_representative_Fam_404_18_2 Nr. of seq. 2 Alignment length(with gaps) = 18 Alignment score = 0.759259

arqueas_representative:NZ_CP009520.1_Methanosarcina_vacuolata_Z-761:88:3294688-3294788 Satlength=101 Nr of Repeats=6 RepeatLength=17 seed=TTTTTATTTT Num.seqs=4 Similarity=0.960784 tpcCG=39.732498 54 TACTTTTTA-TTTTTCAC

arqueas_representative:NZ_CP009515.1_Methanosarcina_lacustris_Z-7289:84:921534-921624 Satlength=91 Nr of Repeats=5 RepeatLength=18 seed=TTTTATTTTT Num.seqs=5 Similarity=0.822222 tpcCG=41.799999 55 TTCNTTTTATTTTTTCAC

***** ********* *

Consensus:

tTTTTAtTTTTTCACTaC

>arqueas_representative_Fam_405_18_2 Nr. of seq. 2 Alignment length(with gaps) = 18 Alignment score = 0.666667

arqueas_representative:NZ_CP009520.1_Methanosarcina_vacuolata_Z-761:88:3732265-3732333 Satlength=69 Nr of Repeats=4 RepeatLength=17 seed=TTCTCAGAAA Num.seqs=4 Similarity=0.921569 tpcCG=39.732498 0 TTCTCAGAA-ATAGTCAG

Rev.of_arqueas_representative:NZ_CP009517.1_Methanosarcina_barkeri_3:81:1135575-1135665 Satlength=91 Nr of Repeats=4 RepeatLength=18 seed=TAGTATCTTC Num.seqs=3 Similarity=0.901235 tpcCG=39.099998 16 TTGTCAGAAGATACTAAG

** ****** *** * **

Consensus:

TTcTCAGAAgATAcTaAG

>arqueas_representative_Fam_406_18_2 Nr. of seq. 2 Alignment length(with gaps) = 18 Alignment score = 0.703704

arqueas_representative:NZ_CP019285.1_Halobiforma_lacisalsi_AJ5:23:3885554-3885677 Satlength=124 Nr of Repeats=5 RepeatLength=18 seed=TTCCTCCTCT Num.seqs=4 Similarity=0.901235 tpcCG=65.237297 0 TTCCTCCTCTTCAGCCGG

arqueas_representative:NZ_CP009517.1_Methanosarcina_barkeri_3:81:1221703-1221910 Satlength=208 Nr of Repeats=6 RepeatLength=18 seed=TCTTCAGGCT Num.seqs=5 Similarity=0.851852 tpcCG=39.099998 7 TACTTCCTCTTCAGGCTG

* * ********** * *

Consensus:

TaCcTCCTCTTCAGcCgG

>arqueas_representative_Fam_407_18_2 Nr. of seq. 2 Alignment length(with gaps) = 18 Alignment score = 0.703704

arqueas_representative:NZ_CP006933.1_Methanobacterium_formicicum__BRM9:45:1973895-1974021 Satlength=127 Nr of Repeats=7 RepeatLength=18 seed=ACACCAGAAA Num.seqs=7 Similarity=0.936508 tpcCG=41.299999 0 ACACCAGAAAACAACCAA

Rev.of_arqueas_representative:NZ_CP009520.1_Methanosarcina_vacuolata_Z-761:88:4399061-4399133 Satlength=73 Nr of Repeats=4 RepeatLength=18 seed=TTTATGGTGT Num.seqs=4 Similarity=0.925926 tpcCG=39.73249 10 ACACCATAAAACCAAAAA

****** ***** * **

Consensus:

ACACCAgAAAACaAaaAA

>arqueas_representative_Fam_408_18_2 Nr. of seq. 2 Alignment length(with gaps) = 18 Alignment score = 0.703704

arqueas_representative:NZ_CP014265.1_Methanobrevibacter_olleyae__YLM1:49:331003-331075 Satlength=73 Nr of Repeats=4 RepeatLength=18 seed=TATATTTGTT Num.seqs=4 Similarity=0.814815 tpcCG=26.900000 0 TATATTTGTTATCTTGAT

Rev.of_arqueas_representative:NZ_CP009517.1_Methanosarcina_barkeri_3:81:4296085-4296211 Satlength=127 Nr of Repeats=7 RepeatLength=18 seed=AACACGAACA Num.seqs=7 Similarity=0.978836 tpcCG=39.099998 12 TATGTTCGTGTTCTTGAT

*** ** ** *******

Consensus:

TATaTTcGTgaTCTTGAT

>arqueas_representative_Fam_409_18_2 Nr. of seq. 2 Alignment length(with gaps) = 18 Alignment score = 0.629630

arqueas_representative:NC_013790.1_Methanobrevibacter_ruminantium_M1:50:2864148-2864223 Satlength=76 Nr of Repeats=4 RepeatLength=18 seed=ATTCTAAAAA Num.seqs=3 Similarity=0.851852 tpcCG=32.599998 0 ATTCTAAAAAGGAGTTTG

arqueas_representative:NZ_CP009520.1_Methanosarcina_vacuolata_Z-761:88:1548544-1548670 Satlength=127 Nr of Repeats=7 RepeatLength=18 seed=AGTCTGAATC Num.seqs=7 Similarity=0.943563 tpcCG=39.732498 12 AATCTAAATAACAGTCTG

* ****** * *** **

Consensus:

AaTCTAAAaAacAGTcTG

>arqueas_representative_Fam_410_18_2 Nr. of seq. 2 Alignment length(with gaps) = 18 Alignment score = 0.703704

arqueas_representative:NC_009637.1_Methanococcus_maripaludis_C7:62:450418-450919 Satlength=502 Nr of Repeats=8 RepeatLength=18 seed=GATACAGGAG Num.seqs=6 Similarity=1.000000 tpcCG=33.299999 0 GATACAGGAGATACTGGT

Rev.of_arqueas_representative:NZ_CP009520.1_Methanosarcina_vacuolata_Z-761:88:2011259-2011412 Satlength=154 Nr of Repeats=8 RepeatLength=18 seed=TCCGTGTCTT Num.seqs=7 Similarity=1.000000 tpcCG=39.7324 17 GATACAGAAGACACGGAT

******* *** ** * *

Consensus:

GATACAGaAGAcACgGaT

>arqueas_representative_Fam_411_18_2 Nr. of seq. 2 Alignment length(with gaps) = 18 Alignment score = 0.629630

arqueas_representative:NC_003552.1_Methanosarcina_acetivorans_str._C2A:80:200940-201290 Satlength=351 Nr of Repeats=4 RepeatLength=18 seed=ATCCGGGAGG Num.seqs=3 Similarity=0.851852 tpcCG=42.700001 0 ATCCGGGAGGTGGAATGT

Rev.of_arqueas_representative:NZ_CP009520.1_Methanosarcina_vacuolata_Z-761:88:593876-594002 Satlength=127 Nr of Repeats=7 RepeatLength=18 seed=AATACTTTAC Num.seqs=7 Similarity=0.837743 tpcCG=39.732498 3 ATTCGGTAGATGTAAAGT

** *** ** ** ** **

Consensus:

ATcCGGgAGaTGgAAaGT

>arqueas_representative_Fam_412_18_2 Nr. of seq. 2 Alignment length(with gaps) = 18 Alignment score = 0.703704

arqueas_representative:NC_003552.1_Methanosarcina_acetivorans_str._C2A:80:856983-857091 Satlength=109 Nr of Repeats=6 RepeatLength=18 seed=CTACCGAGGC Num.seqs=6 Similarity=0.896296 tpcCG=42.700001 0 CTACCGAGGCGGACAACG

Rev.of_arqueas_representative:NC_019974.1_Natronococcus_occultus_SP4:103:2731938-2732028 Satlength=91 Nr of Repeats=5 RepeatLength=18 seed=CCGCGTCGGA Num.seqs=5 Similarity=0.955556 tpcCG=64.629402 12 ATTCCGACGCGGACGACG

* **** ****** ***

Consensus:

aTaCCGAcGCGGACaACG

>arqueas_representative_Fam_413_18_2 Nr. of seq. 2 Alignment length(with gaps) = 18 Alignment score = 1.000000

arqueas_representative:NC_003552.1_Methanosarcina_acetivorans_str._C2A:80:3873745-3874279 Satlength=535 Nr of Repeats=4 RepeatLength=18 seed=TCTACTCCAG Num.seqs=3 Similarity=1.000000 tpcCG=42.700001 0 TCTACTCCAGTTAATACA

Rev.of_arqueas_representative:NZ_CP009506.1_Methanosarcina_siciliae_T4/M:86:3741606-3741714 Satlength=109 Nr of Repeats=6 RepeatLength=18 seed=TAGATGTATT Num.seqs=6 Similarity=0.876543 tpcCG=42.900002 4 TCTACTCCAGTTAATACA

******************

Consensus:

TCTACTCCAGTTAATACA

>arqueas_representative_Fam_414_18_2 Nr. of seq. 2 Alignment length(with gaps) = 18 Alignment score = 0.851852

arqueas_representative:NC_003552.1_Methanosarcina_acetivorans_str._C2A:80:4386558-4386630 Satlength=73 Nr of Repeats=4 RepeatLength=18 seed=TAATCTTGGA Num.seqs=4 Similarity=0.962963 tpcCG=42.700001 0 TAATCTTGGAATTCCTTT

arqueas_representative:NZ_CP009506.1_Methanosarcina_siciliae_T4/M:86:1220107-1220216 Satlength=110 Nr of Repeats=6 RepeatLength=18 seed=AATTCCTTCT Num.seqs=5 Similarity=1.000000 tpcCG=42.900002 9 TAATCTTAGAATTCCTTC

******* *********

Consensus:

TAATCTTaGAATTCCTTc

>arqueas_representative_Fam_415_18_2 Nr. of seq. 2 Alignment length(with gaps) = 18 Alignment score = 0.629630

arqueas_representative:NZ_CP009517.1_Methanosarcina_barkeri_3:81:1347356-1347446 Satlength=91 Nr of Repeats=5 RepeatLength=18 seed=TGGTTAATCT Num.seqs=5 Similarity=0.940741 tpcCG=39.099998 0 TGGTTAATCTCTGTTATC

arqueas_representative:NZ_CP009520.1_Methanosarcina_vacuolata_Z-761:88:582105-582177 Satlength=73 Nr of Repeats=4 RepeatLength=18 seed=TTGTTAATCC Num.seqs=4 Similarity=0.950617 tpcCG=39.732498 0 TTGTTAATCCATACTATC

* ******* * ****

Consensus:

TgGTTAATCcaTacTATC

>arqueas_representative_Fam_416_18_2 Nr. of seq. 2 Alignment length(with gaps) = 18 Alignment score = 0.629630

arqueas_representative:NZ_CP009517.1_Methanosarcina_barkeri_3:81:1486819-1487266 Satlength=448 Nr of Repeats=4 RepeatLength=18 seed=ATTTATCGTA Num.seqs=3 Similarity=1.000000 tpcCG=39.099998 0 ATTTATCGTAACCTTGTA

arqueas_representative:NZ_CP009512.1_Methanosarcina_mazei_S-6:85:2548033-2548187 Satlength=155 Nr of Repeats=8 RepeatLength=18 seed=TCCTAATTTT Num.seqs=5 Similarity=0.911111 tpcCG=41.400002 5 ATTTATCCTAATTTTCTC

******* *** ** *

Consensus:

ATTTATCcTAAccTTcTa

>arqueas_representative_Fam_417_18_2 Nr. of seq. 2 Alignment length(with gaps) = 18 Alignment score = 0.648148

arqueas_representative:NZ_CP009517.1_Methanosarcina_barkeri_3:81:3233836-3233962 Satlength=127 Nr of Repeats=7 RepeatLength=18 seed=AAAGTAACCA Num.seqs=7 Similarity=1.000000 tpcCG=39.099998 0 AAAGTAACCAGAGAAAAC

Rev.of_arqueas_representative:NZ_CP009528.1_Methanosarcina_barkeri_MS:82:2721690-2721780 Satlength=91 Nr of Repeats=5 RepeatLength=18 seed=CTGTGTTTTC Num.seqs=5 Similarity=0.822222 tpcCG=39.168999 4 ACAGGAAACTNAGAAAAC

* ** ** * *******

Consensus:

AaAGgAAaCagAGAAAAC

>arqueas_representative_Fam_418_18_2 Nr. of seq. 2 Alignment length(with gaps) = 18 Alignment score = 0.629630

arqueas_representative:NZ_CP009517.1_Methanosarcina_barkeri_3:81:4328755-4328846 Satlength=92 Nr of Repeats=4 RepeatLength=18 seed=ATCTCACTAA Num.seqs=3 Similarity=0.950617 tpcCG=39.099998 0 ATCTCACTAACAATTAAT

arqueas_representative:NZ_CP009512.1_Methanosarcina_mazei_S-6:85:2505786-2505877 Satlength=92 Nr of Repeats=5 RepeatLength=18 seed=ACTTACTTCT Num.seqs=4 Similarity=0.962963 tpcCG=41.400002 5 ATCTCACTTACTTCTAAC

******** ** ***

Consensus:

ATCTCACTaACaacTAAc

>arqueas_representative_Fam_419_18_2 Nr. of seq. 2 Alignment length(with gaps) = 18 Alignment score = 0.629630

arqueas_representative:NZ_CP009528.1_Methanosarcina_barkeri_MS:82:1878987-1879059 Satlength=73 Nr of Repeats=4 RepeatLength=18 seed=ACTCCAGCTA Num.seqs=4 Similarity=0.802469 tpcCG=39.168999 0 ACTCCAGCTACGGCAGTA

Rev.of_arqueas_representative:NZ_CP009512.1_Methanosarcina_mazei_S-6:85:2940414-2940513 Satlength=100 Nr of Repeats=5 RepeatLength=18 seed=CAACTGGAGT Num.seqs=4 Similarity=0.851852 tpcCG=41.400002 10 ACTCCAGTTGTGGAAGAA

******* * ** ** *

Consensus:

ACTCCAGcTacGGaAGaA

>arqueas_representative_Fam_420_18_2 Nr. of seq. 2 Alignment length(with gaps) = 18 Alignment score = 0.777778

arqueas_representative:NZ_CP009515.1_Methanosarcina_lacustris_Z-7289:84:259703-259919 Satlength=217 Nr of Repeats=12 RepeatLength=18 seed=TCTTTAATCT Num.seqs=12 Similarity=0.873176 tpcCG=41.799999 0 TCTTTAATCTCAGCTTTT

arqueas_representative:NZ_CP009501.1_Methanosarcina_thermophila_TM-1:87:375002-375128 Satlength=127 Nr of Repeats=7 RepeatLength=18 seed=ATCTCAGGTT Num.seqs=7 Similarity=0.957672 tpcCG=41.099998 6 TCCTCAATCTCAGGTTTT

** * ******** ****

Consensus:

TCcTcAATCTCAGcTTTT

>arqueas_representative_Fam_421_18_2 Nr. of seq. 2 Alignment length(with gaps) = 18 Alignment score = 0.777778

arqueas_representative:NZ_CP009515.1_Methanosarcina_lacustris_Z-7289:84:1319285-1319393 Satlength=109 Nr of Repeats=5 RepeatLength=18 seed=AGTAGAACCG Num.seqs=4 Similarity=0.851852 tpcCG=41.799999 0 AGTAGAACCGATAATGAG

Rev.of_arqueas_representative:NZ_CP009515.1_Methanosarcina_lacustris_Z-7289:84:1867172-1867520 Satlength=349 Nr of Repeats=6 RepeatLength=18 seed=TTATCAGCTC Num.seqs=4 Similarity=0.925926 tpcCG=41.799 14 AATAGAGCTGATAATGAG

* **** * *********

Consensus:

AaTAGAaCcGATAATGAG

>arqueas_representative_Fam_422_17_2 Nr. of seq. 2 Alignment length(with gaps) = 17 Alignment score = 0.666667

arqueas_representative:NZ_CP009515.1_Methanosarcina_lacustris_Z-7289:84:826702-826758 Satlength=57 Nr of Repeats=4 RepeatLength=14 seed=ACTGGAAATT Num.seqs=4 Similarity=1.000000 tpcCG=41.799999 0 ACTGGAAAT-TCTTT--

arqueas_representative:NZ_CP009515.1_Methanosarcina_lacustris_Z-7289:84:2968250-2968369 Satlength=120 Nr of Repeats=7 RepeatLength=17 seed=TGGAAATCAC Num.seqs=7 Similarity=0.955182 tpcCG=41.799999 2 ACTGGAAATCACTTTTT

********* ****

Consensus:

ACTGGAAATcaCTTTtt

>arqueas_representative_Fam_423_17_2 Nr. of seq. 2 Alignment length(with gaps) = 17 Alignment score = 0.627451

arqueas_representative:NC_015666.1_Halopiger_xanaduensis_SH-6:30:1655129-1655189 Satlength=61 Nr of Repeats=4 RepeatLength=15 seed=TCGGAGCCGC Num.seqs=4 Similarity=1.000000 tpcCG=65.196602 0 TCGGAGCCGCTGTCC--

arqueas_representative:NZ_CP009515.1_Methanosarcina_lacustris_Z-7289:84:1003432-1003517 Satlength=86 Nr of Repeats=5 RepeatLength=17 seed=TTTCCTATCA Num.seqs=5 Similarity=0.890196 tpcCG=41.799999 10 TCAGAGCCACTTTCCTA

** ***** ** ***

Consensus:

TCaGAGCCaCTgTCCta

>arqueas_representative_Fam_424_17_2 Nr. of seq. 2 Alignment length(with gaps) = 17 Alignment score = 0.607843

arqueas_representative:NZ_CP009517.1_Methanosarcina_barkeri_3:81:3007824-3007929 Satlength=106 Nr of Repeats=5 RepeatLength=15 seed=CCTTCGTGTT Num.seqs=4 Similarity=0.955556 tpcCG=39.099998 0 CCTTCGTGTTTC--ATC

Rev.of_arqueas_representative:NZ_CP009520.1_Methanosarcina_vacuolata_Z-761:88:1326326-1326390 Satlength=65 Nr of Repeats=4 RepeatLength=16 seed=ACATGGGATT Num.seqs=4 Similarity=1.000000 tpcCG=39.73249 6 CCAT-GTGTTTCTAATC

** * ******* ***

Consensus:

CCaTcGTGTTTCtaATC

>arqueas_representative_Fam_425_17_2 Nr. of seq. 2 Alignment length(with gaps) = 17 Alignment score = 0.735294

arqueas_representative:NZ_CP009528.1_Methanosarcina_barkeri_MS:82:2114399-2114559 Satlength=161 Nr of Repeats=8 RepeatLength=15 seed=TAAAAAATAG Num.seqs=7 Similarity=1.000000 tpcCG=39.168999 0 TAAAAAATAG-TGAGA-

Rev.of_arqueas_representative:NZ_CP009512.1_Methanosarcina_mazei_S-6:85:1516003-1516105 Satlength=103 Nr of Repeats=6 RepeatLength=17 seed=CTATTTTTTC Num.seqs=6 Similarity=0.864052 tpcCG=41.400002 10 GAAAAAATAGTTGAGAT

********* *****

Consensus:

gAAAAAATAGtTGAGAt

>arqueas_representative_Fam_426_17_2 Nr. of seq. 2 Alignment length(with gaps) = 17 Alignment score = 0.705882

arqueas_representative:NZ_CP009528.1_Methanosarcina_barkeri_MS:82:2300998-2301122 Satlength=125 Nr of Repeats=8 RepeatLength=15 seed=AGTTTAAGGA Num.seqs=6 Similarity=1.000000 tpcCG=39.168999 0 AGTTTAAGGAAGTAG--

arqueas_representative:NZ_CP009528.1_Methanosarcina_barkeri_MS:82:1379546-1379625 Satlength=80 Nr of Repeats=5 RepeatLength=17 seed=GTAATTTTAA Num.seqs=3 Similarity=0.947712 tpcCG=39.168999 14 ATTTTAAGGAAGAAGTA

* ********** **

Consensus:

AgTTTAAGGAAGaAGta

>arqueas_representative_Fam_427_17_2 Nr. of seq. 2 Alignment length(with gaps) = 17 Alignment score = 0.735294

arqueas_representative:NZ_CP009515.1_Methanosarcina_lacustris_Z-7289:84:225018-225108 Satlength=91 Nr of Repeats=6 RepeatLength=15 seed=TTCTTTTATT Num.seqs=6 Similarity=0.970370 tpcCG=41.799999 0 -TTCTTTT-ATTCCTTC

arqueas_representative:NZ_CP009501.1_Methanosarcina_thermophila_TM-1:87:2742013-2742096 Satlength=84 Nr of Repeats=5 RepeatLength=17 seed=TTTCTTTTAA Num.seqs=4 Similarity=0.921569 tpcCG=41.099998 0 TTTCTTTTAATTCCTTT

******* *******

Consensus:

tTTCTTTTaATTCCTTc

>arqueas_representative_Fam_428_17_2 Nr. of seq. 2 Alignment length(with gaps) = 17 Alignment score = 0.735294

arqueas_representative:NZ_CP009515.1_Methanosarcina_lacustris_Z-7289:84:231255-231420 Satlength=166 Nr of Repeats=11 RepeatLength=15 seed=ATAGCCTAAA Num.seqs=11 Similarity=0.945050 tpcCG=41.799999 0 ATAGCCTAAAA-TCCA-

Rev.of_arqueas_representative:NZ_CP009528.1_Methanosarcina_barkeri_MS:82:2702852-2702988 Satlength=137 Nr of Repeats=8 RepeatLength=17 seed=TATCTGGAAT Num.seqs=8 Similarity=0.960784 tpcCG=39.168999 3 ATAGACTAAAATTCCAG

**** ****** ****

Consensus:

ATAGaCTAAAAtTCCAg

>arqueas_representative_Fam_429_17_2 Nr. of seq. 2 Alignment length(with gaps) = 17 Alignment score = 0.627451

arqueas_representative:NZ_CP009512.1_Methanosarcina_mazei_S-6:85:261099-261174 Satlength=76 Nr of Repeats=5 RepeatLength=15 seed=CTTTTATTTA Num.seqs=3 Similarity=0.679739 tpcCG=41.400002 0 CTTTTA-TTTATCT-TN

Rev.of_arqueas_representative:NZ_CP009512.1_Methanosarcina_mazei_S-6:85:2733900-2733969 Satlength=70 Nr of Repeats=4 RepeatLength=17 seed=GTACAGATAA Num.seqs=3 Similarity=1.000000 tpcCG=41.400002 1 CCTTTAGTTTATCTGTA

* **** ******* *

Consensus:

CcTTTAgTTTATCTgTa

>arqueas_representative_Fam_430_17_2 Nr. of seq. 2 Alignment length(with gaps) = 17 Alignment score = 0.705882

arqueas_representative:NZ_CP009512.1_Methanosarcina_mazei_S-6:85:3355777-3355942 Satlength=166 Nr of Repeats=11 RepeatLength=15 seed=GAAAAATAGA Num.seqs=11 Similarity=1.000000 tpcCG=41.400002 0 GAAAAATAGAGAGGA--

Rev.of_arqueas_representative:NZ_CP009501.1_Methanosarcina_thermophila_TM-1:87:445814-445917 Satlength=104 Nr of Repeats=6 RepeatLength=17 seed=TGTAATCTCT Num.seqs=5 Similarity=1.000000 tpcCG=41.09999 0 GAAAAATAGAGATTACA

************ *

Consensus:

GAAAAATAGAGAggAca

>arqueas_representative_Fam_431_17_2 Nr. of seq. 2 Alignment length(with gaps) = 17 Alignment score = 0.666667

arqueas_representative:NZ_CP009501.1_Methanosarcina_thermophila_TM-1:87:1636052-1636427 Satlength=376 Nr of Repeats=25 RepeatLength=15 seed=CCAAATGAGG Num.seqs=25 Similarity=0.864583 tpcCG=41.099998 4 GAAT--CCAAATGAGGA

arqueas_representative:NZ_CP009517.1_Methanosarcina_barkeri_3:81:4313226-4313354 Satlength=129 Nr of Repeats=8 RepeatLength=16 seed=CAAAGAGGAG Num.seqs=8 Similarity=1.000000 tpcCG=39.099998 5 GAATTAGCAAA-GAGGA

**** *********

Consensus:

cCAAAtGAGGAGAATta

>arqueas_representative_Fam_432_17_2 Nr. of seq. 2 Alignment length(with gaps) = 17 Alignment score = 0.696078

arqueas_representative:NZ_CP009520.1_Methanosarcina_vacuolata_Z-761:88:1891980-1892085 Satlength=106 Nr of Repeats=7 RepeatLength=15 seed=AATTTCTTAG Num.seqs=7 Similarity=1.000000 tpcCG=39.732498 9 TT-AGAG-TATAATTTC

Rev.of_arqueas_representative:NZ_CP009512.1_Methanosarcina_mazei_S-6:85:2291304-2291368 Satlength=65 Nr of Repeats=4 RepeatLength=16 seed=TCTGAAGAAA Num.seqs=4 Similarity=1.000000 tpcCG=41.400002 20 TTCAGAGCTAT-ATTTC

******* **** ***

Consensus:

aATTTCTTcAGAGcTAT

>arqueas_representative_Fam_433_17_2 Nr. of seq. 2 Alignment length(with gaps) = 17 Alignment score = 0.725490

arqueas_representative:NZ_CP009520.1_Methanosarcina_vacuolata_Z-761:88:2612685-2612775 Satlength=91 Nr of Repeats=6 RepeatLength=15 seed=TTAACAGCCT Num.seqs=6 Similarity=1.000000 tpcCG=39.732498 0 TTAACAGC--CTTATAG

arqueas_representative:NZ_CP009515.1_Methanosarcina_lacustris_Z-7289:84:617334-617470 Satlength=137 Nr of Repeats=7 RepeatLength=17 seed=AGCTACTTAT Num.seqs=6 Similarity=0.952941 tpcCG=41.799999 5 TTAACAGCTACTTATGG

******** ***** *

Consensus:

TTAACAGCtaCTTATaG

>arqueas_representative_Fam_434_17_2 Nr. of seq. 2 Alignment length(with gaps) = 17 Alignment score = 0.656863

arqueas_representative:NZ_CP009520.1_Methanosarcina_vacuolata_Z-761:88:3993588-3993678 Satlength=91 Nr of Repeats=6 RepeatLength=15 seed=ATTTGAGTTA Num.seqs=6 Similarity=1.000000 tpcCG=39.732498 2 GA-ATTTGAGTTAGT-T

Rev.of_arqueas_representative:NZ_CP009515.1_Methanosarcina_lacustris_Z-7289:84:2955176-2955295 Satlength=120 Nr of Repeats=7 RepeatLength=17 seed=TATTACACTA Num.seqs=7 Similarity=0.955182 tpcCG=41.799 3 AATATTTGATTTAGTGT

****** ***** * *

Consensus:

ATTTGAgTTAGTgTaAt

>arqueas_representative_Fam_435_17_2 Nr. of seq. 2 Alignment length(with gaps) = 17 Alignment score = 0.735294

arqueas_representative:NC_003552.1_Methanosarcina_acetivorans_str._C2A:80:1656894-1656974 Satlength=81 Nr of Repeats=5 RepeatLength=16 seed=AAATTAAGAT Num.seqs=5 Similarity=1.000000 tpcCG=42.700001 0 AAATTAAGATTGTGGT-

arqueas_representative:NC_003552.1_Methanosarcina_acetivorans_str._C2A:80:4533615-4533741 Satlength=127 Nr of Repeats=7 RepeatLength=16 seed=ATTGGGTCAA Num.seqs=5 Similarity=0.900000 tpcCG=42.700001 8 AAAATAAGATTG-GGTC

*** ******** ***

Consensus:

AAAaTAAGATTGtGGTc

>arqueas_representative_Fam_436_17_2 Nr. of seq. 2 Alignment length(with gaps) = 17 Alignment score = 0.647059

arqueas_representative:NC_003552.1_Methanosarcina_acetivorans_str._C2A:80:2815470-2815534 Satlength=65 Nr of Repeats=4 RepeatLength=16 seed=TTAAGTTAAA Num.seqs=4 Similarity=0.888889 tpcCG=42.700001 0 TTAAGTTAAAG-TCCTA

Rev.of_arqueas_representative:NZ_CP009517.1_Methanosarcina_barkeri_3:81:331629-331731 Satlength=103 Nr of Repeats=6 RepeatLength=17 seed=TTATCTTTGT Num.seqs=6 Similarity=1.000000 tpcCG=39.099998 9 CAAAGATAAAGCTCCTA

*** ***** *****

Consensus:

caAAGaTAAAGcTCCTA

>arqueas_representative_Fam_437_17_2 Nr. of seq. 2 Alignment length(with gaps) = 17 Alignment score = 0.686275

arqueas_representative:NZ_CP009517.1_Methanosarcina_barkeri_3:81:781464-781592 Satlength=129 Nr of Repeats=8 RepeatLength=16 seed=TACAAGAGAA Num.seqs=8 Similarity=0.794468 tpcCG=39.099998 0 TACAAGAGAAG-ATCTT

Rev.of_arqueas_representative:NZ_CP009512.1_Methanosarcina_mazei_S-6:85:2438393-2438586 Satlength=194 Nr of Repeats=5 RepeatLength=16 seed=TTTAATGATA Num.seqs=4 Similarity=0.784314 tpcCG=41.400002 4 TA-AAGAGAAGTATCAT

** ******** *** *

Consensus:

TAcAAGAGAAGtATCaT

>arqueas_representative_Fam_438_17_2 Nr. of seq. 2 Alignment length(with gaps) = 17 Alignment score = 0.656863

arqueas_representative:NZ_CP009517.1_Methanosarcina_barkeri_3:81:1017656-1017752 Satlength=97 Nr of Repeats=6 RepeatLength=16 seed=TTTTTAATTC Num.seqs=6 Similarity=0.872222 tpcCG=39.099998 3 TGT-TTTTTAATTCAAG

Rev.of_arqueas_representative:NZ_CP009506.1_Methanosarcina_siciliae_T4/M:86:3316757-3316853 Satlength=97 Nr of Repeats=6 RepeatLength=16 seed=ATTAAAAATA Num.seqs=6 Similarity=1.000000 tpcCG=42.900002 11 AGTATTTTTAA-TCAAT

******* **** **

Consensus:

TTTTTAAtTCAAgaGTa

>arqueas_representative_Fam_439_17_2 Nr. of seq. 2 Alignment length(with gaps) = 17 Alignment score = 0.725490

arqueas_representative:NZ_CP009517.1_Methanosarcina_barkeri_3:81:1600932-1601043 Satlength=112 Nr of Repeats=7 RepeatLength=16 seed=TTACTTGCTT Num.seqs=6 Similarity=1.000000 tpcCG=39.099998 0 TTACTTGCT-TTACTGT

Rev.of_arqueas_representative:NZ_CP009512.1_Methanosarcina_mazei_S-6:85:2252618-2252686 Satlength=69 Nr of Repeats=4 RepeatLength=17 seed=AAACAGTAAT Num.seqs=4 Similarity=0.921569 tpcCG=41.400002 2 TTACTTATTATTACTGT

****** * *******

Consensus:

TTACTTacTaTTACTGT

>arqueas_representative_Fam_440_17_2 Nr. of seq. 2 Alignment length(with gaps) = 17 Alignment score = 0.725490

arqueas_representative:NZ_CP009517.1_Methanosarcina_barkeri_3:81:1804802-1804882 Satlength=81 Nr of Repeats=5 RepeatLength=16 seed=AGTAAGAGAG Num.seqs=5 Similarity=0.966667 tpcCG=39.099998 0 AG-TAAGAGAGTTAATA

Rev.of_arqueas_representative:NZ_CP009501.1_Methanosarcina_thermophila_TM-1:87:223533-223618 Satlength=86 Nr of Repeats=4 RepeatLength=17 seed=TTATCTTCAT Num.seqs=3 Similarity=0.790850 tpcCG=41.099998 6 AGATAAGAGAGTTATGA

** *********** *

Consensus:

AGaTAAGAGAGTTAagA

>arqueas_representative_Fam_441_17_2 Nr. of seq. 2 Alignment length(with gaps) = 17 Alignment score = 0.686275

arqueas_representative:NZ_CP009517.1_Methanosarcina_barkeri_3:81:2420416-2420528 Satlength=113 Nr of Repeats=7 RepeatLength=16 seed=GATATGTGAA Num.seqs=7 Similarity=0.888889 tpcCG=39.099998 4 CTTAGA-TATGTGAATT

arqueas_representative:NZ_CP009517.1_Methanosarcina_barkeri_3:81:3871491-3871555 Satlength=65 Nr of Repeats=4 RepeatLength=16 seed=ACTATGTGAT Num.seqs=4 Similarity=0.916667 tpcCG=39.099998 5 CGTAGACTATGTG-ATT

** ****** **** **

Consensus:

GAcTATGTGaATTCgTA

>arqueas_representative_Fam_442_17_2 Nr. of seq. 2 Alignment length(with gaps) = 17 Alignment score = 0.725490

arqueas_representative:NZ_CP009517.1_Methanosarcina_barkeri_3:81:2929294-2929358 Satlength=65 Nr of Repeats=4 RepeatLength=16 seed=TGTAAATTCA Num.seqs=4 Similarity=1.000000 tpcCG=39.099998 0 TGTAAATTCATT-CTCT

Rev.of_arqueas_representative:NZ_CP009528.1_Methanosarcina_barkeri_MS:82:3716778-3716880 Satlength=103 Nr of Repeats=6 RepeatLength=17 seed=AAGAATAATG Num.seqs=6 Similarity=1.000000 tpcCG=39.168999 1 TGTAAAATCATTATTCT

****** ***** ***

Consensus:

TGTAAAaTCATTacTCT

>arqueas_representative_Fam_443_17_2 Nr. of seq. 2 Alignment length(with gaps) = 17 Alignment score = 0.656863

arqueas_representative:NZ_CP009528.1_Methanosarcina_barkeri_MS:82:225849-226149 Satlength=301 Nr of Repeats=6 RepeatLength=16 seed=CCTAACTTAT Num.seqs=5 Similarity=0.890196 tpcCG=39.168999 0 CCTAACTTATTG-CTTT

arqueas_representative:NZ_CP009512.1_Methanosarcina_mazei_S-6:85:1333618-1333698 Satlength=81 Nr of Repeats=5 RepeatLength=16 seed=AATTAATTGA Num.seqs=5 Similarity=1.000000 tpcCG=41.400002 2 -CTAATTAATTGACTTT

**** * **** ****

Consensus:

cCTAAcTaATTGaCTTT

>arqueas_representative_Fam_444_17_2 Nr. of seq. 2 Alignment length(with gaps) = 17 Alignment score = 0.647059

arqueas_representative:NZ_CP009528.1_Methanosarcina_barkeri_MS:82:3359508-3359719 Satlength=212 Nr of Repeats=12 RepeatLength=16 seed=TAAAACGTAA Num.seqs=11 Similarity=1.000000 tpcCG=39.168999 0 TAAAACGTAAA-AAGAG

Rev.of_arqueas_representative:NZ_CP009515.1_Methanosarcina_lacustris_Z-7289:84:2239849-2240368 Satlength=520 Nr of Repeats=7 RepeatLength=17 seed=ACTCATCTTT Num.seqs=5 Similarity=1.000000 tpcCG=41.799 1 TAAAATCTAAAGATGAG

***** **** * ***

Consensus:

TAAAAccTAAAgAaGAG

>arqueas_representative_Fam_445_17_2 Nr. of seq. 2 Alignment length(with gaps) = 17 Alignment score = 0.774510

arqueas_representative:CP009516.1_Methanosarcina_horonobensis_HB-1:83:1861284-1861364 Satlength=81 Nr of Repeats=5 RepeatLength=16 seed=AAGGAAGTAA Num.seqs=5 Similarity=1.000000 tpcCG=41.299999 10 GTAAAATTAA-AAGGAA

Rev.of_arqueas_representative:NZ_CP009506.1_Methanosarcina_siciliae_T4/M:86:2837076-2837144 Satlength=69 Nr of Repeats=4 RepeatLength=17 seed=CTTATTGATT Num.seqs=4 Similarity=1.000000 tpcCG=42.900002 13 GTAGAATCAATAAGGAA

********* *** **

Consensus:

AAGGAAGTAaAATcAAt

>arqueas_representative_Fam_446_17_2 Nr. of seq. 2 Alignment length(with gaps) = 17 Alignment score = 0.696078

arqueas_representative:NZ_CP009515.1_Methanosarcina_lacustris_Z-7289:84:1036702-1036774 Satlength=73 Nr of Repeats=4 RepeatLength=16 seed=AGTCTCAAAG Num.seqs=3 Similarity=1.000000 tpcCG=41.799999 0 AGTCTCAAAGTCTCAA-

Rev.of_arqueas_representative:NZ_CP009528.1_Methanosarcina_barkeri_MS:82:535877-535962 Satlength=86 Nr of Repeats=4 RepeatLength=17 seed=TACTGAGATT Num.seqs=3 Similarity=0.864198 tpcCG=39.168999 1 ACTCTCAAAATCTCAGT

* ******* *****

Consensus:

AcTCTCAAAaTCTCAat

>arqueas_representative_Fam_447_17_2 Nr. of seq. 2 Alignment length(with gaps) = 17 Alignment score = 0.725490

arqueas_representative:NZ_CP009512.1_Methanosarcina_mazei_S-6:85:1800957-1801037 Satlength=81 Nr of Repeats=5 RepeatLength=16 seed=TATTTTGTTC Num.seqs=5 Similarity=1.000000 tpcCG=41.400002 12 TTGTTCAAA-TAATATT

arqueas_representative:NZ_CP009520.1_Methanosarcina_vacuolata_Z-761:88:1444967-1445103 Satlength=137 Nr of Repeats=8 RepeatLength=17 seed=TTTAGTTCAA Num.seqs=8 Similarity=0.918768 tpcCG=39.732498 14 TAGTTCAAATTTATATT

***** ******* * *

Consensus:

TATTTaGTTCAAAtTaA

>arqueas_representative_Fam_448_17_2 Nr. of seq. 2 Alignment length(with gaps) = 17 Alignment score = 0.647059

arqueas_representative:NZ_CP009512.1_Methanosarcina_mazei_S-6:85:2979916-2979980 Satlength=65 Nr of Repeats=4 RepeatLength=16 seed=TTCATCTTTA Num.seqs=4 Similarity=0.902778 tpcCG=41.400002 0 TTCATCTTTAA-GCTCT

Rev.of_arqueas_representative:NZ_CP009515.1_Methanosarcina_lacustris_Z-7289:84:3696670-3696908 Satlength=239 Nr of Repeats=14 RepeatLength=17 seed=AAGATTGTTA Num.seqs=14 Similarity=1.000000 tpcCG=41.7 1 TTCAGCTTTAACAATCT

**** ****** ***

Consensus:

TTCAgCTTTAAcaaTCT

>arqueas_representative_Fam_449_17_2 Nr. of seq. 2 Alignment length(with gaps) = 17 Alignment score = 0.696078

arqueas_representative:NZ_CP009506.1_Methanosarcina_siciliae_T4/M:86:683704-683800 Satlength=97 Nr of Repeats=6 RepeatLength=16 seed=ATGAAATTTA Num.seqs=6 Similarity=0.916667 tpcCG=42.900002 9 TTAGAGTGA-ATGAAAT

arqueas_representative:NZ_CP009520.1_Methanosarcina_vacuolata_Z-761:88:3912412-3912548 Satlength=137 Nr of Repeats=4 RepeatLength=17 seed=AAGAGTTAAA Num.seqs=3 Similarity=0.947712 tpcCG=39.732498 17 TAAGAGTTAAATGAATT

***** ** ***** *

Consensus:

ATGAAaTTaAGAGTgAa

>arqueas_representative_Fam_450_17_2 Nr. of seq. 2 Alignment length(with gaps) = 17 Alignment score = 0.686275

arqueas_representative:NZ_CP009506.1_Methanosarcina_siciliae_T4/M:86:683765-683877 Satlength=113 Nr of Repeats=6 RepeatLength=16 seed=TGGATGAAAT Num.seqs=5 Similarity=0.816667 tpcCG=42.900002 8 ATCTG-TAGTGGATGAA

Rev.of_arqueas_representative:NZ_CP009506.1_Methanosarcina_siciliae_T4/M:86:2958521-2958585 Satlength=65 Nr of Repeats=4 RepeatLength=16 seed=ATCAGATTCA Num.seqs=4 Similarity=0.847222 tpcCG=42.900002 22 ATCTGATACTGGATG-A

****** ****** **

Consensus:

TGGATGaAATCTGaTAc

>arqueas_representative_Fam_451_17_2 Nr. of seq. 2 Alignment length(with gaps) = 17 Alignment score = 0.647059

arqueas_representative:NZ_CP009520.1_Methanosarcina_vacuolata_Z-761:88:10063-10127 Satlength=65 Nr of Repeats=4 RepeatLength=16 seed=CTGTCTTCAC Num.seqs=4 Similarity=1.000000 tpcCG=39.732498 0 CTG-TCTTCACAATAAC

arqueas_representative:NZ_CP009520.1_Methanosarcina_vacuolata_Z-761:88:685619-685704 Satlength=86 Nr of Repeats=5 RepeatLength=17 seed=TTCAAAATTA Num.seqs=5 Similarity=0.921569 tpcCG=39.732498 6 CTGTTCTTCAAAATTAT

*** ****** *** *

Consensus:

CTGtTCTTCAaAATaAc

>arqueas_representative_Fam_452_17_2 Nr. of seq. 2 Alignment length(with gaps) = 17 Alignment score = 0.725490

arqueas_representative:NZ_CP009520.1_Methanosarcina_vacuolata_Z-761:88:731921-732017 Satlength=97 Nr of Repeats=6 RepeatLength=16 seed=TCTTCTAATT Num.seqs=6 Similarity=1.000000 tpcCG=39.732498 0 TCTTCTAATT-ATAGGC

arqueas_representative:NZ_CP009520.1_Methanosarcina_vacuolata_Z-761:88:1476290-1476408 Satlength=119 Nr of Repeats=7 RepeatLength=17 seed=GTCTCTTCAA Num.seqs=6 Similarity=1.000000 tpcCG=39.732498 14 TCTTCAAATTCATAGTC

***** **** **** *

Consensus:

TCTTCaAATTcATAGgC

>arqueas_representative_Fam_453_17_2 Nr. of seq. 2 Alignment length(with gaps) = 17 Alignment score = 0.647059

arqueas_representative:NZ_CP009520.1_Methanosarcina_vacuolata_Z-761:88:2042351-2042477 Satlength=127 Nr of Repeats=8 RepeatLength=16 seed=ACTTAGAACT Num.seqs=7 Similarity=1.000000 tpcCG=39.732498 0 ACTTAGAACTA-TTAGG

Rev.of_arqueas_representative:NZ_CP009520.1_Methanosarcina_vacuolata_Z-761:88:3207719-3207787 Satlength=69 Nr of Repeats=4 RepeatLength=17 seed=AGTTTTACGT Num.seqs=4 Similarity=0.960784 tpcCG=39.73249 3 ACTTAGAACTACGTAAA

*********** **

Consensus:

ACTTAGAACTAcgTAaa

>arqueas_representative_Fam_454_17_2 Nr. of seq. 2 Alignment length(with gaps) = 17 Alignment score = 0.725490

arqueas_representative:NZ_CP009520.1_Methanosarcina_vacuolata_Z-761:88:2664370-2664482 Satlength=113 Nr of Repeats=5 RepeatLength=16 seed=TAACATATAA Num.seqs=4 Similarity=0.681373 tpcCG=39.732498 0 TAACATAT-AAACGAGT

arqueas_representative:NZ_CP009520.1_Methanosarcina_vacuolata_Z-761:88:2673495-2673699 Satlength=205 Nr of Repeats=12 RepeatLength=17 seed=TATCAATCGA Num.seqs=12 Similarity=0.902555 tpcCG=39.732498 5 TAACATATCAATCGAAT

******** ** *** *

Consensus:

TAACATATcAAaCGAaT

>arqueas_representative_Fam_455_17_2 Nr. of seq. 2 Alignment length(with gaps) = 17 Alignment score = 0.647059

arqueas_representative:NZ_CP009520.1_Methanosarcina_vacuolata_Z-761:88:2904526-2904606 Satlength=81 Nr of Repeats=5 RepeatLength=16 seed=AAGTACGGAG Num.seqs=5 Similarity=0.890196 tpcCG=39.732498 0 AAGTACGGAG-AACAGA

arqueas_representative:NZ_CP009517.1_Methanosarcina_barkeri_3:81:1312721-1312789 Satlength=69 Nr of Repeats=4 RepeatLength=17 seed=AGGAGAAACA Num.seqs=4 Similarity=0.921569 tpcCG=39.099998 5 AAGTAAGGAGAAACACT

***** **** ****

Consensus:

AAGTAaGGAGaAACAca

>arqueas_representative_Fam_456_17_2 Nr. of seq. 2 Alignment length(with gaps) = 17 Alignment score = 0.725490

arqueas_representative:NZ_CP009520.1_Methanosarcina_vacuolata_Z-761:88:3338340-3338612 Satlength=273 Nr of Repeats=17 RepeatLength=16 seed=AGTTTCAATC Num.seqs=17 Similarity=0.968137 tpcCG=39.732498 0 AGTTTCAATCATAA-TC

arqueas_representative:NZ_CP009517.1_Methanosarcina_barkeri_3:81:2872464-2872566 Satlength=103 Nr of Repeats=6 RepeatLength=17 seed=TGTTTCAATT Num.seqs=6 Similarity=0.947712 tpcCG=39.099998 0 TGTTTCAATTATAATTC

******** **** **

Consensus:

aGTTTCAATcATAAtTC

>arqueas_representative_Fam_457_17_2 Nr. of seq. 2 Alignment length(with gaps) = 17 Alignment score = 0.764706

arqueas_representative:NC_003552.1_Methanosarcina_acetivorans_str._C2A:80:3320434-3320883 Satlength=450 Nr of Repeats=12 RepeatLength=17 seed=TAGTTACTTT Num.seqs=11 Similarity=1.000000 tpcCG=42.700001 15 GTTACTTTTTATCTTTA

Rev.of_arqueas_representative:NZ_CP009506.1_Methanosarcina_siciliae_T4/M:86:2388877-2388945 Satlength=69 Nr of Repeats=4 RepeatLength=17 seed=AAAGGAAGTT Num.seqs=4 Similarity=0.960784 tpcCG=42.900002 25 CTTCCTTTTTATCTTAA

* ** ***********

Consensus:

aAcTTaCTTTTTATCTT

>arqueas_representative_Fam_458_17_2 Nr. of seq. 2 Alignment length(with gaps) = 17 Alignment score = 0.686275

arqueas_representative:NC_003552.1_Methanosarcina_acetivorans_str._C2A:80:5123539-5123624 Satlength=86 Nr of Repeats=5 RepeatLength=17 seed=TACCTTTTTT Num.seqs=5 Similarity=1.000000 tpcCG=42.700001 0 TACCTTTTTTGTTTCTC

Rev.of_arqueas_representative:NZ_CP009528.1_Methanosarcina_barkeri_MS:82:570458-570594 Satlength=137 Nr of Repeats=8 RepeatLength=17 seed=TGAAATCAAA Num.seqs=8 Similarity=0.896359 tpcCG=39.168999 16 TATCTTTTTGATTTCAC

** ****** **** *

Consensus:

TAcCTTTTTgaTTTCaC

>arqueas_representative_Fam_459_17_2 Nr. of seq. 2 Alignment length(with gaps) = 17 Alignment score = 0.607843

arqueas_representative:NZ_CP009517.1_Methanosarcina_barkeri_3:81:1286249-1286351 Satlength=103 Nr of Repeats=6 RepeatLength=17 seed=ATCAATTATA Num.seqs=6 Similarity=1.000000 tpcCG=39.099998 0 ATCAATTATATAGTGAC

Rev.of_arqueas_representative:NZ_CP009506.1_Methanosarcina_siciliae_T4/M:86:2592813-2592881 Satlength=69 Nr of Repeats=4 RepeatLength=17 seed=TTTTTATAAT Num.seqs=4 Similarity=0.921569 tpcCG=42.900002 14 ATCCATTATAAAAAGTC

*** ****** * * *

Consensus:

ATCaATTATAaAaaGaC

>arqueas_representative_Fam_460_17_2 Nr. of seq. 2 Alignment length(with gaps) = 17 Alignment score = 0.921569

arqueas_representative:NZ_CP009517.1_Methanosarcina_barkeri_3:81:2532184-2532303 Satlength=120 Nr of Repeats=7 RepeatLength=17 seed=TTAAACAAAC Num.seqs=7 Similarity=0.955182 tpcCG=39.099998 0 TTAAACAAACCTTAGAC

arqueas_representative:NZ_CP009520.1_Methanosarcina_vacuolata_Z-761:88:3564408-3564510 Satlength=103 Nr of Repeats=6 RepeatLength=17 seed=TTAAACAAAC Num.seqs=6 Similarity=1.000000 tpcCG=39.732498 0 TTAAACAAACCTTAAAC

************** **

Consensus:

TTAAACAAACCTTAaAC

>arqueas_representative_Fam_461_17_2 Nr. of seq. 2 Alignment length(with gaps) = 17 Alignment score = 0.686275

arqueas_representative:NZ_CP009517.1_Methanosarcina_barkeri_3:81:3746041-3746143 Satlength=103 Nr of Repeats=6 RepeatLength=17 seed=AGTTACAAAA Num.seqs=6 Similarity=1.000000 tpcCG=39.099998 0 AGTTACAAAAATGAATT

Rev.of_arqueas_representative:NZ_CP009520.1_Methanosarcina_vacuolata_Z-761:88:1272964-1273117 Satlength=154 Nr of Repeats=9 RepeatLength=17 seed=TGTAGCTAAT Num.seqs=9 Similarity=1.000000 tpcCG=39.7324 7 AGCTACAGAAATTCATT

** **** **** ***

Consensus:

AGcTACAaAAATgaATT

>arqueas_representative_Fam_462_17_2 Nr. of seq. 2 Alignment length(with gaps) = 17 Alignment score = 0.607843

arqueas_representative:NZ_CP009528.1_Methanosarcina_barkeri_MS:82:226846-226999 Satlength=154 Nr of Repeats=9 RepeatLength=17 seed=ATACAGGTAA Num.seqs=9 Similarity=0.925926 tpcCG=39.168999 0 ATACAGGTAAAACTTCT

arqueas_representative:NZ_CP009528.1_Methanosarcina_barkeri_MS:82:2459200-2459268 Satlength=69 Nr of Repeats=4 RepeatLength=17 seed=TACATAGCCA Num.seqs=4 Similarity=1.000000 tpcCG=39.168999 16 ACATAGCCATAACTTCT

* * ** * *******

Consensus:

AcAcAGccAaAACTTCT

>arqueas_representative_Fam_463_17_2 Nr. of seq. 2 Alignment length(with gaps) = 17 Alignment score = 0.686275

arqueas_representative:NZ_CP009528.1_Methanosarcina_barkeri_MS:82:3847588-3847674 Satlength=87 Nr of Repeats=4 RepeatLength=17 seed=TGTTATTAAC Num.seqs=3 Similarity=1.000000 tpcCG=39.168999 0 TGTTATTAACTTAATCC

arqueas_representative:NZ_CP009528.1_Methanosarcina_barkeri_MS:82:3847678-3847780 Satlength=103 Nr of Repeats=6 RepeatLength=17 seed=ATTAACTGAA Num.seqs=6 Similarity=1.000000 tpcCG=39.168999 4 TGTCATTAACTGAATAT

*** ******* ***

Consensus:

TGTcATTAACTgAATac

>arqueas_representative_Fam_464_17_2 Nr. of seq. 2 Alignment length(with gaps) = 17 Alignment score = 0.686275

arqueas_representative:NZ_CP009512.1_Methanosarcina_mazei_S-6:85:2500058-2500143 Satlength=86 Nr of Repeats=5 RepeatLength=17 seed=TTTTCTAAAT Num.seqs=5 Similarity=1.000000 tpcCG=41.400002 0 TTTTCTAAATGGATATC

arqueas_representative:NZ_CP009520.1_Methanosarcina_vacuolata_Z-761:88:3791546-3791750 Satlength=205 Nr of Repeats=12 RepeatLength=17 seed=GAAATCTTTT Num.seqs=12 Similarity=1.000000 tpcCG=39.732498 11 TTTTCTATCTAGAAATC

******* * ** ***

Consensus:

TTTTCTAaaTaGAaATC

>arqueas_representative_Fam_465_17_2 Nr. of seq. 2 Alignment length(with gaps) = 17 Alignment score = 0.607843

arqueas_representative:NZ_CP009506.1_Methanosarcina_siciliae_T4/M:86:192304-192389 Satlength=86 Nr of Repeats=5 RepeatLength=17 seed=TTATCATATT Num.seqs=5 Similarity=1.000000 tpcCG=42.900002 0 TTATCATATTGATGATG

Rev.of_arqueas_representative:NZ_CP009520.1_Methanosarcina_vacuolata_Z-761:88:3611948-3612101 Satlength=154 Nr of Repeats=9 RepeatLength=17 seed=GATTTCGTCA Num.seqs=9 Similarity=0.921569 tpcCG=39.7324 5 AAATCAGATTAATGACG

**** *** **** *

Consensus:

aaATCAgATTaATGAcG

>arqueas_representative_Fam_466_16_2 Nr. of seq. 2 Alignment length(with gaps) = 16 Alignment score = 0.625000

arqueas_representative:NC_019977.1_Methanomethylovorans_hollandica_DSM_15978:75:308332-308392 Satlength=61 Nr of Repeats=5 RepeatLength=12 seed=TTACTGTATC Num.seqs=5 Similarity=1.000000 tpcCG=41.84360 5 ATCACTTACTGT----

Rev.of_arqueas_representative:NC_018015.1_Thermococcus_cleftensis__CL1:123:909683-909891 Satlength=209 Nr of Repeats=13 RepeatLength=16 seed=TAAGGAGGTG Num.seqs=13 Similarity=1.000000 tpcCG=55.799999 3 TTACTATATCACCTCC

* **

Consensus:

aTaacaTAccacctcc

>arqueas_representative_Fam_467_16_2 Nr. of seq. 2 Alignment length(with gaps) = 16 Alignment score = 0.635417

arqueas_representative:NZ_CP009528.1_Methanosarcina_barkeri_MS:82:208439-208504 Satlength=66 Nr of Repeats=5 RepeatLength=13 seed=CATAACCTTG Num.seqs=5 Similarity=1.000000 tpcCG=39.168999 0 CATAACCTTGAC--C-

Rev.of_arqueas_representative:NZ_CP009517.1_Methanosarcina_barkeri_3:81:1997266-1997378 Satlength=113 Nr of Repeats=7 RepeatLength=16 seed=TGTGATGTCA Num.seqs=7 Similarity=1.000000 tpcCG=39.099998 2 CATAAGCTTGACATCA

***** ****** *

Consensus:

CATAAcCTTGACatCa

>arqueas_representative_Fam_468_16_2 Nr. of seq. 2 Alignment length(with gaps) = 16 Alignment score = 0.729167

arqueas_representative:NZ_CP009528.1_Methanosarcina_barkeri_MS:82:2043906-2043958 Satlength=53 Nr of Repeats=4 RepeatLength=13 seed=ATTTAGAATG Num.seqs=4 Similarity=0.948718 tpcCG=39.168999 0 ATT-TAGAATGTAA--

Rev.of_arqueas_representative:NZ_CP009517.1_Methanosarcina_barkeri_3:81:1936184-1936264 Satlength=81 Nr of Repeats=5 RepeatLength=16 seed=TATAATTCTT Num.seqs=5 Similarity=0.950000 tpcCG=39.099998 6 ATTATAGAATGTAAGA

*** **********

Consensus:

ATTaTAGAATGTAAga

>arqueas_representative_Fam_469_16_2 Nr. of seq. 2 Alignment length(with gaps) = 16 Alignment score = 0.677083

arqueas_representative:NZ_CP009515.1_Methanosarcina_lacustris_Z-7289:84:3976153-3976205 Satlength=53 Nr of Repeats=4 RepeatLength=13 seed=GGTTTAATTA Num.seqs=4 Similarity=1.000000 tpcCG=41.799999 0 -GGTTTA-ATT-AATC

Rev.of_arqueas_representative:NZ_CP009515.1_Methanosarcina_lacustris_Z-7289:84:2611577-2611705 Satlength=129 Nr of Repeats=8 RepeatLength=16 seed=GATTTAATGT Num.seqs=8 Similarity=0.964286 tpcCG=41.799 0 CGGTTTACATTAAATC

****** *** ****

Consensus:

cGGTTTAcATTaAATC

>arqueas_representative_Fam_470_16_2 Nr. of seq. 2 Alignment length(with gaps) = 16 Alignment score = 0.718750

arqueas_representative:NZ_CP009512.1_Methanosarcina_mazei_S-6:85:2508937-2508989 Satlength=53 Nr of Repeats=4 RepeatLength=13 seed=TTCAAATGAA Num.seqs=4 Similarity=1.000000 tpcCG=41.400002 0 TTCAAATGA--AATA-

arqueas_representative:NZ_CP009517.1_Methanosarcina_barkeri_3:81:3974961-3975024 Satlength=64 Nr of Repeats=4 RepeatLength=16 seed=AATGAGTAAT Num.seqs=3 Similarity=1.000000 tpcCG=39.099998 4 TTCAAATGAGTAATAA

********* ****

Consensus:

TTCAAATGAgtAATAa

>arqueas_representative_Fam_471_16_2 Nr. of seq. 2 Alignment length(with gaps) = 16 Alignment score = 0.645833

arqueas_representative:NZ_CP009506.1_Methanosarcina_siciliae_T4/M:86:1255709-1255775 Satlength=67 Nr of Repeats=4 RepeatLength=13 seed=TTGGAGAATT Num.seqs=3 Similarity=1.000000 tpcCG=42.900002 0 TTG-GAGAATTTCT--

arqueas_representative:NZ_CP009520.1_Methanosarcina_vacuolata_Z-761:88:62352-62463 Satlength=112 Nr of Repeats=4 RepeatLength=16 seed=TTTCTAGTTG Num.seqs=3 Similarity=1.000000 tpcCG=39.732498 9 TTGCGTGAATTTCTAG

*** * ********

Consensus:

TTGcGaGAATTTCTag

>arqueas_representative_Fam_472_16_2 Nr. of seq. 2 Alignment length(with gaps) = 16 Alignment score = 0.687500

arqueas_representative:NZ_CP009517.1_Methanosarcina_barkeri_3:81:2021994-2022120 Satlength=127 Nr of Repeats=9 RepeatLength=14 seed=GACAAGAATT Num.seqs=9 Similarity=0.962963 tpcCG=39.099998 0 --GACAAGAATTCATG

Rev.of_arqueas_representative:CP009516.1_Methanosarcina_horonobensis_HB-1:83:1830323-1830403 Satlength=81 Nr of Repeats=5 RepeatLength=16 seed=CATCAATTAT Num.seqs=5 Similarity=1.000000 tpcCG=41.299999 0 TAGACAATAATTGATG

***** **** ***

Consensus:

taGACAAgAATTcATG

>arqueas_representative_Fam_473_16_2 Nr. of seq. 2 Alignment length(with gaps) = 16 Alignment score = 0.718750

arqueas_representative:NZ_CP009528.1_Methanosarcina_barkeri_MS:82:3188527-3188583 Satlength=57 Nr of Repeats=4 RepeatLength=14 seed=GTTTTTTAAT Num.seqs=4 Similarity=0.952381 tpcCG=39.168999 0 GTTTTTTA-ATTTTG-

arqueas_representative:NZ_CP009528.1_Methanosarcina_barkeri_MS:82:3225914-3225978 Satlength=65 Nr of Repeats=4 RepeatLength=16 seed=TTTTATATTT Num.seqs=4 Similarity=1.000000 tpcCG=39.168999 3 GCTTTTTATATTTTGA

* ****** ******

Consensus:

GcTTTTTAtATTTTGa

>arqueas_representative_Fam_474_16_2 Nr. of seq. 2 Alignment length(with gaps) = 16 Alignment score = 0.687500

arqueas_representative:NZ_CP009515.1_Methanosarcina_lacustris_Z-7289:84:2643213-2643395 Satlength=183 Nr of Repeats=11 RepeatLength=14 seed=AAAACCATTT Num.seqs=10 Similarity=0.961905 tpcCG=41.799999 0 AAAACCATTTACTC--

arqueas_representative:NZ_CP009515.1_Methanosarcina_lacustris_Z-7289:84:130139-130219 Satlength=81 Nr of Repeats=5 RepeatLength=16 seed=TTTTCTCAAA Num.seqs=5 Similarity=0.900000 tpcCG=41.799999 7 AAAACCCTTTTCTCAA

****** *** ***

Consensus:

AAAACCaTTTaCTCaa

>arqueas_representative_Fam_475_16_2 Nr. of seq. 2 Alignment length(with gaps) = 16 Alignment score = 0.718750

arqueas_representative:NZ_CP009520.1_Methanosarcina_vacuolata_Z-761:88:2255-2353 Satlength=99 Nr of Repeats=7 RepeatLength=14 seed=AAGACGTTTT Num.seqs=7 Similarity=0.702381 tpcCG=39.732498 0 AAGACGTTTTCGT-A-

Rev.of_arqueas_representative:NZ_CP009528.1_Methanosarcina_barkeri_MS:82:4527872-4528012 Satlength=141 Nr of Repeats=8 RepeatLength=16 seed=TTTTGATGAA Num.seqs=7 Similarity=0.809524 tpcCG=39.168999 2 AAGACGTTTTCATCAA

*********** * *

Consensus:

AAGACGTTTTCaTcAa

>arqueas_representative_Fam_476_16_2 Nr. of seq. 2 Alignment length(with gaps) = 16 Alignment score = 0.718750

arqueas_representative:NZ_CP009520.1_Methanosarcina_vacuolata_Z-761:88:815119-815231 Satlength=113 Nr of Repeats=8 RepeatLength=14 seed=TTAGACTGTT Num.seqs=8 Similarity=0.846939 tpcCG=39.732498 0 TTAGACTGTT-CAAG-

Rev.of_arqueas_representative:NC_003552.1_Methanosarcina_acetivorans_str._C2A:80:160068-160148 Satlength=81 Nr of Repeats=5 RepeatLength=16 seed=GCTTGTAAAA Num.seqs=5 Similarity=1.000000 tpcCG=42.7000 0 TTAGACTTTTACAAGC

******* ** ****

Consensus:

TTAGACTgTTaCAAGc

>arqueas_representative_Fam_477_16_2 Nr. of seq. 2 Alignment length(with gaps) = 16 Alignment score = 0.687500

arqueas_representative:NZ_CP009520.1_Methanosarcina_vacuolata_Z-761:88:4495189-4495395 Satlength=207 Nr of Repeats=15 RepeatLength=14 seed=AAAACCTGGA Num.seqs=11 Similarity=0.876364 tpcCG=39.732498 5 ATAAG--AAAACCTGG

arqueas_representative:NZ_CP009515.1_Methanosarcina_lacustris_Z-7289:84:873845-873909 Satlength=65 Nr of Repeats=4 RepeatLength=16 seed=AAAACCTGAA Num.seqs=4 Similarity=1.000000 tpcCG=41.799999 5 AAAAGGTAAAACCTGA

******** * ***

Consensus:

AAAACCTGaAaAAGgt

>arqueas_representative_Fam_478_16_2 Nr. of seq. 2 Alignment length(with gaps) = 16 Alignment score = 0.708333

arqueas_representative:NZ_LN831302.1_Halobacterium_hubeiense__JI20-1:21:639949-640039 Satlength=91 Nr of Repeats=5 RepeatLength=15 seed=TGATTCGGTG Num.seqs=4 Similarity=0.911111 tpcCG=66.575996 0 TGATTCGGTGG-TGGG

Rev.of_arqueas_representative:NC_013922.1_Natrialba_magadii_ATCC_43099:100:301540-301676 Satlength=137 Nr of Repeats=8 RepeatLength=16 seed=ACCACCGAAC Num.seqs=7 Similarity=0.904762 tpcCG=61.032001 4 TGGTTCGGTGGTTCGG

** ******** * **

Consensus:

TGaTTCGGTGGtTcGG

>arqueas_representative_Fam_479_16_2 Nr. of seq. 2 Alignment length(with gaps) = 16 Alignment score = 0.666667

arqueas_representative:NZ_CP006933.1_Methanobacterium_formicicum__BRM9:45:1868757-1869027 Satlength=271 Nr of Repeats=18 RepeatLength=15 seed=GAACTAAACT Num.seqs=18 Similarity=1.000000 tpcCG=41.299999 0 GAACTAA-ACTAACTA

arqueas_representative:NZ_CP009528.1_Methanosarcina_barkeri_MS:82:1721125-1721260 Satlength=136 Nr of Repeats=9 RepeatLength=15 seed=TAACACAAAC Num.seqs=9 Similarity=1.000000 tpcCG=39.168999 3 GAA-TAACACAAACTA

*** *** ** *****

Consensus:

GAAcTAAcACaAACTA

>arqueas_representative_Fam_480_16_2 Nr. of seq. 2 Alignment length(with gaps) = 16 Alignment score = 0.625000

arqueas_representative:NZ_CP011266.1_Methanobrevibacter_millerae__SM9:48:969976-970066 Satlength=91 Nr of Repeats=6 RepeatLength=15 seed=TTCTGGTGGT Num.seqs=6 Similarity=0.893333 tpcCG=31.799999 0 TTCTG-GTGGTAGTAC
[truncated: 936,448 more chars]
