# Supplementary material for: Satellites in the prokaryote world
Source: BMC Evol Biol. 2019 Sep 18;19:181. doi: 10.1186/s12862-019-1504-2 (PMC6749651; doi:10.1186/s12862-019-1504-2)
Supplement: Supplementary file 7 — Table S6. A summary list of all satellite families. (PDF 458 kb) [file 12862_2019_1504_MOESM7_ESM.pdf]

Table S6a. Archaea satellite families

```
>Fam_1_126_16  Nr. of seq. 16 Alignment length(with gaps) = 136 Alignment score = 0.626532
GTAATTGACACtGCaACaAAcAatGTTACAGCcACnGTganTGTAGGAaantaTCCTTgnGGAGTTGCAGTcAacCCGGaTGGAACAAAgGTATATGTGgCgAAntgnaGn
nAgcAacAcTGTcTCT
>Fam_2_12_13  Nr. of seq. 13 Alignment length(with gaps) = 13 Alignment score = 0.648258
 AtTATTATTAgt
>Fam_3_12_10  Nr. of seq. 10 Alignment length(with gaps) = 14 Alignment score = 0.625132
 TAAAAntAAAAa
>Fam_4_12_10  Nr. of seq. 10 Alignment length(with gaps) = 12 Alignment score = 0.642593
 TgTTaTTgTTaT
>Fam_5_16_8  Nr. of seq. 8 Alignment length(with gaps) = 16 Alignment score = 0.667411
 aTTanttTaTTatTtt
>Fam_6_10_8  Nr. of seq. 8 Alignment length(with gaps) = 12 Alignment score = 0.679067
 ATAAAATAAt
>Fam_7_12_8  Nr. of seq. 8 Alignment length(with gaps) = 12 Alignment score = 0.777282
 tTTTCaCCcAAC
>Fam_8_16_7  Nr. of seq. 7 Alignment length(with gaps) = 17 Alignment score = 0.700280
 TgAtnAAAAACTAAAC
>Fam_9_14_7  Nr. of seq. 7 Alignment length(with gaps) = 16 Alignment score = 0.602183
 TntgAAAGGAAAAn
>Fam_10_13_7  Nr. of seq. 7 Alignment length(with gaps) = 15 Alignment score = 0.661376
 TTATTATtTTAtt
>Fam_11_14_7  Nr. of seq. 7 Alignment length(with gaps) = 15 Alignment score = 0.634392
 tTCtTTaaTCnTTA
>Fam_12_12_7  Nr. of seq. 7 Alignment length(with gaps) = 13 Alignment score = 0.636142
 aTTgATAAAtAA
>Fam_13_10_7  Nr. of seq. 7 Alignment length(with gaps) = 12 Alignment score = 0.666667
 TTAtTTTTTA
>Fam_14_10_7  Nr. of seq. 7 Alignment length(with gaps) = 12 Alignment score = 0.619048
 TatTTTttTC
>Fam_15_246_6  Nr. of seq. 6 Alignment length(with gaps) = 260 Alignment score = 0.646752

CCAAAatcCCATTTCCATTTaGTnGGtgtTCCTGTgCTtgTGTCAGTAAATTTAACgtTTAAtGGtacTtTTCCTGAgGTnGGAGAtGCaGAgAAtgCAGCAacgGGTTTT
GtTatnACtgTTATATAnTcTGtTTTTGTTaCtGTgTTACTGCCTGCaGCATTngTTACTGTAAGTnTAACaGTATAtnTTCCTGCTTTtGAATACTTATGagTTGGATTC
TGgagGaaTGActTTGtTCCaTCT
>Fam_16_141_6  Nr. of seq. 6 Alignment length(with gaps) = 141 Alignment score = 0.668558
```

AAAcTTCTGAATGCGATtATTncCtGtATCGGCAACATAAACATTgCCcGAAGAATCtACAGCAAtACCanatGGataattAAATTgtCCgTcgcCGcTGCCataaGAACC
CCATtgagTaAggaAttTaCCacTGCTaTC
>Fam_17_78_6 Nr. of seq. 6 Alignment length(with gaps) = 82 Alignment score = 0.664228
aGCACCACCATAcTcatTaGCATGgTTATTgacAAAAGAACAAtCacTTAAAatACCATTAgCACCATcCCAATaAAc
>Fam_18_12_6 Nr. of seq. 6 Alignment length(with gaps) = 14 Alignment score = 0.651587
AAGaAGAAAAAG
>Fam_19_13_6 Nr. of seq. 6 Alignment length(with gaps) = 14 Alignment score = 0.647619
TcTTCTATTTTaA
>Fam_20_12_6 Nr. of seq. 6 Alignment length(with gaps) = 13 Alignment score = 0.671795
CTCTTCnTtCTT
>Fam_21_12_6 Nr. of seq. 6 Alignment length(with gaps) = 12 Alignment score = 0.733333
gTCGTGtTCGtG
>Fam_22_11_6 Nr. of seq. 6 Alignment length(with gaps) = 11 Alignment score = 0.751515
aaAtAtaAaaA
>Fam_23_156_5 Nr. of seq. 5 Alignment length(with gaps) = 158 Alignment score = 0.831646

TCAGTCCAGCAGACCctCGATGGcGGgTATATCATTAcGGGctatACAtagTCCTTCGGGGcAGGCnAnannGATCTgTGGCTGATcAAGACCGATGAcCAGGGAAACAaG
CTATGGGAgAGGACgTTTGGCGGaGcAGAAtntGATgaGGGCnAt
>Fam_24_141_5 Nr. of seq. 5 Alignment length(with gaps) = 142 Alignment score = 0.677700

CCAGAAGTTCAcnTCGACCGGcgnaTTCaTCAcgaaaTGGgcaGTnnnGgctCnGgAgaCGGGCaGTTCaactnnCCcnatGGtaTCGCnGTgGACAgCGCcGGcAAcGT
CTACGTcancGAcncgnnnAACaaCCgGnT
>Fam_25_120_5 Nr. of seq. 5 Alignment length(with gaps) = 128 Alignment score = 0.693750

tTGTTgTtTAtcGtGTTAATtACAGtgACaGTaTTGCTGttAacaTTaGTCACATAtgCnTttgnTCCtgCnAcTGCaATTCCaGcAGGAtacTctCCAACATtAAttGTG
cCTatAACa
>Fam_26_108_5 Nr. of seq. 5 Alignment length(with gaps) = 109 Alignment score = 0.735474
TCCATGCCTgTTGtTTATCtGGtAcTTGAgAAAAtGCAGAAcCAAGAGCnntgGcaGCactAgacCTcACATcatgaTctTcATCatTggTcAGTcTAagTAAaTCaT
>Fam_27_37_5 Nr. of seq. 5 Alignment length(with gaps) = 37 Alignment score = 0.756757
CACAgAGGaCACagAGAggAtaTTcACCACgGAGGTt
>Fam_28_17_5 Nr. of seq. 5 Alignment length(with gaps) = 19 Alignment score = 0.673684
AATAAcAAAaTAAAaaG
>Fam_29_16_5 Nr. of seq. 5 Alignment length(with gaps) = 17 Alignment score = 0.603922
TAaCAGatTTAtTcnn
>Fam_30_15_5 Nr. of seq. 5 Alignment length(with gaps) = 16 Alignment score = 0.652083
aGaaGAgAAGaAAgA
>Fam_31_15_5 Nr. of seq. 5 Alignment length(with gaps) = 16 Alignment score = 0.716667

CTAAtCTTnaAGgAG
>Fam_32_12_5 Nr. of seq. 5 Alignment length(with gaps) = 15 Alignment score = 0.702222
GTTGAATCaTCt
>Fam_33_14_5 Nr. of seq. 5 Alignment length(with gaps) = 15 Alignment score = 0.666667
gAAaaAGAgAaAAt
>Fam_34_14_5 Nr. of seq. 5 Alignment length(with gaps) = 15 Alignment score = 0.624444
CTTAatnAAGtTaC
>Fam_35_15_5 Nr. of seq. 5 Alignment length(with gaps) = 15 Alignment score = 0.773333
tTAAAAaACGATAna
>Fam_36_13_5 Nr. of seq. 5 Alignment length(with gaps) = 14 Alignment score = 0.633333
AtTctTCtTnCTA
>Fam_37_14_5 Nr. of seq. 5 Alignment length(with gaps) = 14 Alignment score = 0.885714
ACTcGGGTGAcGGa
>Fam_38_14_5 Nr. of seq. 5 Alignment length(with gaps) = 14 Alignment score = 0.885714
CAACGGTTTcGggT
>Fam_39_13_5 Nr. of seq. 5 Alignment length(with gaps) = 13 Alignment score = 0.610256
TTcTaACttcTan
>Fam_40_12_5 Nr. of seq. 5 Alignment length(with gaps) = 13 Alignment score = 0.648718
tTCAgtTAATAn
>Fam_41_11_5 Nr. of seq. 5 Alignment length(with gaps) = 12 Alignment score = 0.644444
TTctTttnTaa
>Fam_42_12_5 Nr. of seq. 5 Alignment length(with gaps) = 12 Alignment score = 0.672222
nAgtTTcAGATt
>Fam_43_10_5 Nr. of seq. 5 Alignment length(with gaps) = 11 Alignment score = 0.630303
tTaanTatTt
>Fam_44_255_4 Nr. of seq. 4 Alignment length(with gaps) = 260 Alignment score = 0.755769

CCTGTgCTCTGGtCAgTAAAACtAACaGTAAGAGGcGctTTTCCTGAAGtngGaGAtGCAGAgAAncTGGnaACAGGagcATcTAaAaCGTTTGAgACAGcAATATAgCcG
GaTTTTGTcAAtGcatTACTgCCgTTTgCATtaCTTgCtGTCAATGTAACAGAaTAtagTCCTGatTTatTGTAtgTgTGtACaGGATTctTtTCTGTTGAAnTAcTtCCa
tCTCCaAAAgtCCATtTCcaTGaagTTGGngAt
>Fam_45_123_4 Nr. of seq. 4 Alignment length(with gaps) = 124 Alignment score = 0.768817

AAtAcaTtGCaACgGCGAGtgaTGACAAtACAgCACGtTTATGGaAtgcAtCTACAGGTAAAcAAATttttTGTTCTGAACCAtgatggTtcGgTaAAtAaTgtTgTaTTCA
GtCCTGATGGAA
>Fam_46_102_4 Nr. of seq. 4 Alignment length(with gaps) = 103 Alignment score = 0.779935
ttTTTCATAGCATTCcAaTGCCTCaTCATATcTCccAAgTtCtTcaAGGAcTAcTCCTTTgTTGTTCCATGcTTCtaCaagTTtTGGaTtTATTTgTAgTGC
>Fam_47_69_4 Nr. of seq. 4 Alignment length(with gaps) = 70 Alignment score = 0.722619
tAAGATTTTTcAacTCtGagATTTCaGGaGGcAgcGAAgTCAaTtGATTncaAGAtAtgTtaAGTggAG

>Fam_48_45_4 Nr. of seq. 4 Alignment length(with gaps) = 47 Alignment score = 0.725768
gGCGACGGgCCGaAGGaCCGGAGtTtGAGCaCCGcAGGTGcGant
>Fam_49_27_4 Nr. of seq. 4 Alignment length(with gaps) = 30 Alignment score = 0.642593
AAGCatTTtTtgAAAAGGgTTGCGGgc
>Fam_50_18_4 Nr. of seq. 4 Alignment length(with gaps) = 20 Alignment score = 0.627778
cTAAaAAAcAAAAAAcaG
>Fam_51_20_4 Nr. of seq. 4 Alignment length(with gaps) = 20 Alignment score = 0.613889
atACaaAAnAAGaaaaAAAT
>Fam_52_16_4 Nr. of seq. 4 Alignment length(with gaps) = 18 Alignment score = 0.643519
tTTAatAAATATAanT
>Fam_53_16_4 Nr. of seq. 4 Alignment length(with gaps) = 18 Alignment score = 0.646605
aTaATTgCTtTTTAAc
>Fam_54_18_4 Nr. of seq. 4 Alignment length(with gaps) = 18 Alignment score = 0.654321
CGgcGTcTCcGTggcCgT
>Fam_55_16_4 Nr. of seq. 4 Alignment length(with gaps) = 17 Alignment score = 0.699346
aAAAATAAAAATnCTa
>Fam_56_16_4 Nr. of seq. 4 Alignment length(with gaps) = 17 Alignment score = 0.655229
tGAtAAATGAAtgaTg
>Fam_57_15_4 Nr. of seq. 4 Alignment length(with gaps) = 17 Alignment score = 0.684641
ctAGAAtTAAAGAAA
>Fam_58_16_4 Nr. of seq. 4 Alignment length(with gaps) = 17 Alignment score = 0.673203
gATTCAATTtCaTAan
>Fam_59_15_4 Nr. of seq. 4 Alignment length(with gaps) = 17 Alignment score = 0.660131
AAAAtAAcTGGgACA
>Fam_60_17_4 Nr. of seq. 4 Alignment length(with gaps) = 17 Alignment score = 0.653595
cCTtTctcTTCtATcTt
>Fam_61_14_4 Nr. of seq. 4 Alignment length(with gaps) = 16 Alignment score = 0.630208
TGAaatTTaAtTTA
>Fam_62_14_4 Nr. of seq. 4 Alignment length(with gaps) = 16 Alignment score = 0.671875
GTtgTTAAAAAaCa
>Fam_63_14_4 Nr. of seq. 4 Alignment length(with gaps) = 15 Alignment score = 0.622222
cATATaATcTCAac
>Fam_64_14_4 Nr. of seq. 4 Alignment length(with gaps) = 15 Alignment score = 0.609259
AaAtACTAAaTAat
>Fam_65_14_4 Nr. of seq. 4 Alignment length(with gaps) = 15 Alignment score = 0.659259
ATCTcATtTTAAat
>Fam_66_14_4 Nr. of seq. 4 Alignment length(with gaps) = 15 Alignment score = 0.640741
GAAACaGatAAgAG

>Fam_67_14_4 Nr. of seq. 4 Alignment length(with gaps) = 15 Alignment score = 0.707407
AGCtAAAAaCagAT
>Fam_68_14_4 Nr. of seq. 4 Alignment length(with gaps) = 15 Alignment score = 0.751852
CTTTTTcCTTTctT
>Fam_69_14_4 Nr. of seq. 4 Alignment length(with gaps) = 15 Alignment score = 0.751852
TACaATTTTTaCAt
>Fam_70_15_4 Nr. of seq. 4 Alignment length(with gaps) = 15 Alignment score = 0.603704
ATCaGttAAtaaAGg
>Fam_71_15_4 Nr. of seq. 4 Alignment length(with gaps) = 15 Alignment score = 0.681481
CCTGAATCtattaAa
>Fam_72_15_4 Nr. of seq. 4 Alignment length(with gaps) = 15 Alignment score = 0.777778
AGAAGaACCaGTaGa
>Fam_73_13_4 Nr. of seq. 4 Alignment length(with gaps) = 14 Alignment score = 0.656746
gAaATAgAAAnTG
>Fam_74_14_4 Nr. of seq. 4 Alignment length(with gaps) = 14 Alignment score = 0.650794
tGaAgGTAAaTAta
>Fam_75_13_4 Nr. of seq. 4 Alignment length(with gaps) = 14 Alignment score = 0.648810
GaAGaAaCTAAAn
>Fam_76_14_4 Nr. of seq. 4 Alignment length(with gaps) = 14 Alignment score = 0.654762
CAGAaatCcTTAtT
>Fam_77_13_4 Nr. of seq. 4 Alignment length(with gaps) = 14 Alignment score = 0.619048
TTTTgAgTtTcgA
>Fam_78_11_4 Nr. of seq. 4 Alignment length(with gaps) = 13 Alignment score = 0.653846
TTACTTtTTnT
>Fam_79_13_4 Nr. of seq. 4 Alignment length(with gaps) = 13 Alignment score = 0.670940
tATCtGttCATTc
>Fam_80_12_4 Nr. of seq. 4 Alignment length(with gaps) = 13 Alignment score = 0.685897
AAAtCAaTCAaa
>Fam_81_11_4 Nr. of seq. 4 Alignment length(with gaps) = 13 Alignment score = 0.666667
aATcAAAATCA
>Fam_82_13_4 Nr. of seq. 4 Alignment length(with gaps) = 13 Alignment score = 0.779915
gAATTcAGGTtAa
>Fam_83_13_4 Nr. of seq. 4 Alignment length(with gaps) = 13 Alignment score = 0.858974
TATCaATCAGTtt
>Fam_84_13_4 Nr. of seq. 4 Alignment length(with gaps) = 13 Alignment score = 0.698718
AgCAAGtTActCa
>Fam_85_12_4 Nr. of seq. 4 Alignment length(with gaps) = 13 Alignment score = 0.711538
AAaTAATatcTA

```
>Fam_86_13_4  Nr. of seq. 4 Alignment length(with gaps) = 13 Alignment score = 0.698718
 AAACTAAaagCTc
>Fam_87_11_4  Nr. of seq. 4 Alignment length(with gaps) = 12 Alignment score = 0.671296
 aTTATAtTaTA
>Fam_88_12_4  Nr. of seq. 4 Alignment length(with gaps) = 12 Alignment score = 0.648148
 TTaaTaGTTtAG
>Fam_89_12_4  Nr. of seq. 4 Alignment length(with gaps) = 12 Alignment score = 0.692130
 TTTctttaTTcA
>Fam_90_12_4  Nr. of seq. 4 Alignment length(with gaps) = 12 Alignment score = 0.685185
 GGtTCagGcTCT
>Fam_91_12_4  Nr. of seq. 4 Alignment length(with gaps) = 12 Alignment score = 0.685185
 TTgCtattTGAA
>Fam_92_10_4  Nr. of seq. 4 Alignment length(with gaps) = 11 Alignment score = 0.638889
 TAAAaAgGat
>Fam_93_11_4  Nr. of seq. 4 Alignment length(with gaps) = 11 Alignment score = 0.717172
 ATTGaAAAttA
>Fam_94_11_4  Nr. of seq. 4 Alignment length(with gaps) = 11 Alignment score = 0.858586
 AtATTATGAGa
>Fam_95_258_3  Nr. of seq. 3 Alignment length(with gaps) = 264 Alignment score = 0.640152

TAcACngTaAaaCTnACAGCAACcAATGCnGCAGGcAGtAAtACgntAACAAAATcaAAtTAcATaAcAgTnACAGgaACanctgCACAAacnCCgGTTGCagnaTTTTcG
GcaTctCCnACtTCaGGAAAtgCaCCAttgAntGTnAcnTTTACTGAcAgcAGTACaGGntCtCCAACagCnTGGAacTGGaaTTTcGGAGAcGGnACanctTCAaCAgtc
CAGAatCCaAnaCACACATATTCaaCaGCAGGAAct
>Fam_96_137_3  Nr. of seq. 3 Alignment length(with gaps) = 142 Alignment score = 0.667449

AATCTTGAAACtGCtaTCngTcTTTAcgGnGAtGCCAGgAAATaTTCCCaAAAacAAGcgtAgaTTACGCTcgTGCgTTGAtgAAcGAGGGTaaTGCAAGAcAAacACTTG
CaGaaATGGGtgTTGAnAGtAgggaA
>Fam_97_126_3  Nr. of seq. 3 Alignment length(with gaps) = 132 Alignment score = 0.647306

TCTGTAATTGAcACAgCTACAAACActGTTACAGcCACtGTnaaTGTAGGAgActnaTCCTantGaaGTTGCAgTCAgtCCtgAcGGAAAAAAaGTnTATGTgACaAAnnc
AatAGcaAcaATaTt
>Fam_98_120_3  Nr. of seq. 3 Alignment length(with gaps) = 121 Alignment score = 0.666667

AAGAAGCagTgaAAAagTATAACCAgtCaCTggAAATtaaaGAAGAncTtGGagAcAAaagcGGAATTgCAnnAaCAcTgCACCAgcTTGGAAngATTtATtAtcaTCAGG
GcAAtTAcG
>Fam_99_120_3  Nr. of seq. 3 Alignment length(with gaps) = 120 Alignment score = 0.679630
```

TAagtCCATTTgAGgGTTCCGTCAGggTTcAgtGCaTAtAGtTTnTTgTCanngCTTCCgATgTAGATGGTtCCGTCngctCCnATTGCtGgtGAACtGtagATctnattT
CCgGTGGTg
>Fam_100_102_3 Nr. of seq. 3 Alignment length(with gaps) = 102 Alignment score = 0.738562
 TTATACTGTTCTTCTGCtTCtTCnaGnCGcCCCATtTcTtnaAGGAGAtTtCCGTAAtTgnAGTGtGtattnACATgTTTtGGgTCtGcTTCCAGaGCAAGc
>Fam_101_93_3 Nr. of seq. 3 Alignment length(with gaps) = 93 Alignment score = 0.930705
 TTTCCAAGTGCCTCTGCCGCACTnCtCCGCACATATGAATCTTcATCTTTGAGTGCGTTGATTAaCGGCTGCACtGCTgTATCCGAtTTaATA
>Fam_102_51_3 Nr. of seq. 3 Alignment length(with gaps) = 54 Alignment score = 0.602881
 TTCGcGtttTCTCctGCTCCgGCCCTTCGgcCcgtCGCcAgTCgAAaAcgC
>Fam_103_42_3 Nr. of seq. 3 Alignment length(with gaps) = 48 Alignment score = 0.800926
 CCTCTTCGGTAGGTTCAGGAGTCATCGTAGgCTCtGGAGTGA
>Fam_104_23_3 Nr. of seq. 3 Alignment length(with gaps) = 25 Alignment score = 0.622222
 GAAAAgTaAAGAAgAnAcAAaaA
>Fam_105_21_3 Nr. of seq. 3 Alignment length(with gaps) = 24 Alignment score = 0.625000
 TnTtTTTTAAtTaTAAGTTAa
>Fam_106_19_3 Nr. of seq. 3 Alignment length(with gaps) = 22 Alignment score = 0.601010
 atTattTATTTtatnTtTt
>Fam_107_18_3 Nr. of seq. 3 Alignment length(with gaps) = 21 Alignment score = 0.629630
 aAAGAaTAACTTcAAAAt
>Fam_108_19_3 Nr. of seq. 3 Alignment length(with gaps) = 21 Alignment score = 0.613757
 TTnGtTtTCTTAtTTTCTn
>Fam_109_17_3 Nr. of seq. 3 Alignment length(with gaps) = 20 Alignment score = 0.627778
 ttTTTaAGAATTTATcA
>Fam_110_20_3 Nr. of seq. 3 Alignment length(with gaps) = 20 Alignment score = 0.644444
 aaGAGATaGaGTaaATAAAa
>Fam_111_19_3 Nr. of seq. 3 Alignment length(with gaps) = 20 Alignment score = 0.755556
 gAaTCTAAAAAAAcGAAtG
>Fam_112_16_3 Nr. of seq. 3 Alignment length(with gaps) = 19 Alignment score = 0.631579
 AACTTacTTTTTTCcC
>Fam_113_17_3 Nr. of seq. 3 Alignment length(with gaps) = 19 Alignment score = 0.654971
 AAATTAAAAgAgAGAtn
>Fam_114_17_3 Nr. of seq. 3 Alignment length(with gaps) = 19 Alignment score = 0.649123
 AAAgaAACtAAAAACaC
>Fam_115_18_3 Nr. of seq. 3 Alignment length(with gaps) = 19 Alignment score = 0.684211
 TaATATTtGGaCTTAtCt
>Fam_116_19_3 Nr. of seq. 3 Alignment length(with gaps) = 19 Alignment score = 0.602339
 AgcaagtAATACcTGtTAA
>Fam_117_19_3 Nr. of seq. 3 Alignment length(with gaps) = 19 Alignment score = 0.789474

TCAATTTtACAnTTTcaTA
>Fam_118_18_3 Nr. of seq. 3 Alignment length(with gaps) = 19 Alignment score = 0.725146
AGgAtAAAAAAGGaAgAA
>Fam_119_18_3 Nr. of seq. 3 Alignment length(with gaps) = 19 Alignment score = 0.678363
TAATCTcATTGATttCtt
>Fam_120_18_3 Nr. of seq. 3 Alignment length(with gaps) = 19 Alignment score = 0.631579
TTTTcTTTTaActATtaT
>Fam_121_18_3 Nr. of seq. 3 Alignment length(with gaps) = 19 Alignment score = 0.608187
AAaATaAATTaGntaTTT
>Fam_122_15_3 Nr. of seq. 3 Alignment length(with gaps) = 18 Alignment score = 0.617284
CAtCGCtaCCGtCAC
>Fam_123_17_3 Nr. of seq. 3 Alignment length(with gaps) = 18 Alignment score = 0.629630
cTCAGTATTcTgTaTtc
>Fam_124_16_3 Nr. of seq. 3 Alignment length(with gaps) = 18 Alignment score = 0.691358
AAAAATGaAGAAaAcG
>Fam_125_18_3 Nr. of seq. 3 Alignment length(with gaps) = 18 Alignment score = 0.641975
TCCaGTAtttTCTTTTnn
>Fam_126_17_3 Nr. of seq. 3 Alignment length(with gaps) = 18 Alignment score = 0.611111
AaAtTTTaAttTACAaC
>Fam_127_17_3 Nr. of seq. 3 Alignment length(with gaps) = 18 Alignment score = 0.611111
TATanTTTCAAgAAtAn
>Fam_128_17_3 Nr. of seq. 3 Alignment length(with gaps) = 18 Alignment score = 0.635802
ATTtTTGtTCTnaTCtT
>Fam_129_18_3 Nr. of seq. 3 Alignment length(with gaps) = 18 Alignment score = 0.802469
CTgCAACcGAAGagATGA
>Fam_130_15_3 Nr. of seq. 3 Alignment length(with gaps) = 17 Alignment score = 0.627451
AaATTAtTTTtATCt
>Fam_131_16_3 Nr. of seq. 3 Alignment length(with gaps) = 17 Alignment score = 0.624183
AACTTTAAGTaanGag
>Fam_132_17_3 Nr. of seq. 3 Alignment length(with gaps) = 17 Alignment score = 0.647059
AAnaTGATCTTgAaAtn
>Fam_133_15_3 Nr. of seq. 3 Alignment length(with gaps) = 17 Alignment score = 0.679739
TtACTTaGTTTcAGA
>Fam_134_16_3 Nr. of seq. 3 Alignment length(with gaps) = 17 Alignment score = 0.653595
ttTtTAAGAAAnTGAG
>Fam_135_15_3 Nr. of seq. 3 Alignment length(with gaps) = 17 Alignment score = 0.738562
AAAATCAATtACAnT
>Fam_136_15_3 Nr. of seq. 3 Alignment length(with gaps) = 17 Alignment score = 0.627451

nCaTTTTCATCAGTg
>Fam_137_15_3 Nr. of seq. 3 Alignment length(with gaps) = 17 Alignment score = 0.607843
TTAAACAaAAttgCa
>Fam_138_15_3 Nr. of seq. 3 Alignment length(with gaps) = 17 Alignment score = 0.660131
AnTGAAAAtAAcACA
>Fam_139_16_3 Nr. of seq. 3 Alignment length(with gaps) = 17 Alignment score = 0.653595
TtAtAnGTTTAtTTTt
>Fam_140_16_3 Nr. of seq. 3 Alignment length(with gaps) = 17 Alignment score = 0.725490
TTcgTTTTTCCTGATa
>Fam_141_17_3 Nr. of seq. 3 Alignment length(with gaps) = 17 Alignment score = 0.614379
TTnTaCTgaGTnTTTAa
>Fam_142_16_3 Nr. of seq. 3 Alignment length(with gaps) = 17 Alignment score = 0.699346
AGnTATTCAGcTatTC
>Fam_143_17_3 Nr. of seq. 3 Alignment length(with gaps) = 17 Alignment score = 0.607843
GnAttATgATTGAAAct
>Fam_144_17_3 Nr. of seq. 3 Alignment length(with gaps) = 17 Alignment score = 0.692810
tgCTTTAaTCtGTTtTc
>Fam_145_13_3 Nr. of seq. 3 Alignment length(with gaps) = 16 Alignment score = 0.645833
tCgAAGAAAAAGA
>Fam_146_15_3 Nr. of seq. 3 Alignment length(with gaps) = 16 Alignment score = 0.604167
tAAnATCCTTtcTCt
>Fam_147_14_3 Nr. of seq. 3 Alignment length(with gaps) = 16 Alignment score = 0.625000
ATCTGGTAAtnGTt
>Fam_148_15_3 Nr. of seq. 3 Alignment length(with gaps) = 16 Alignment score = 0.618056
CTGAcATAcAAgGAn
>Fam_149_14_3 Nr. of seq. 3 Alignment length(with gaps) = 16 Alignment score = 0.625000
AGTGTTaaTTTGAt
>Fam_150_14_3 Nr. of seq. 3 Alignment length(with gaps) = 16 Alignment score = 0.659722
AaATTAcTTTCTgT
>Fam_151_15_3 Nr. of seq. 3 Alignment length(with gaps) = 16 Alignment score = 0.618056
CAAaGAntAAGAGaC
>Fam_152_15_3 Nr. of seq. 3 Alignment length(with gaps) = 16 Alignment score = 0.673611
cTATTTTTnTtACTT
>Fam_153_15_3 Nr. of seq. 3 Alignment length(with gaps) = 16 Alignment score = 0.638889
ATTcAtAGaTTGatA
>Fam_154_15_3 Nr. of seq. 3 Alignment length(with gaps) = 16 Alignment score = 0.618056
TTTTaTTtTAtaCTa
>Fam_155_15_3 Nr. of seq. 3 Alignment length(with gaps) = 16 Alignment score = 0.673611

ACCAgTatTGAAAAa
>Fam_156_16_3 Nr. of seq. 3 Alignment length(with gaps) = 16 Alignment score = 0.659722
TTttAAAAcAAncCTt
>Fam_157_15_3 Nr. of seq. 3 Alignment length(with gaps) = 16 Alignment score = 0.645833
TaTTnCtAAcCAGAT
>Fam_158_16_3 Nr. of seq. 3 Alignment length(with gaps) = 16 Alignment score = 0.729167
TTTatTTCAGgTTTct
>Fam_159_15_3 Nr. of seq. 3 Alignment length(with gaps) = 16 Alignment score = 0.673611
ATGTtgAaCTaAATT
>Fam_160_16_3 Nr. of seq. 3 Alignment length(with gaps) = 16 Alignment score = 0.638889
CTTAActangTAAtAA
>Fam_161_16_3 Nr. of seq. 3 Alignment length(with gaps) = 16 Alignment score = 0.666667
TTTAGTtaATTCCnnt
>Fam_162_12_3 Nr. of seq. 3 Alignment length(with gaps) = 15 Alignment score = 0.614815
TTTCTTCATtGg
>Fam_163_15_3 Nr. of seq. 3 Alignment length(with gaps) = 15 Alignment score = 0.762963
CCGCCGCaTCGTcaT
>Fam_164_13_3 Nr. of seq. 3 Alignment length(with gaps) = 15 Alignment score = 0.800000
TTTTAGAATCCGt
>Fam_165_12_3 Nr. of seq. 3 Alignment length(with gaps) = 15 Alignment score = 0.622222
TTTTTCTtTCCc
>Fam_166_13_3 Nr. of seq. 3 Alignment length(with gaps) = 15 Alignment score = 0.644444
tttTATTTATTCA
>Fam_167_14_3 Nr. of seq. 3 Alignment length(with gaps) = 15 Alignment score = 0.629630
AGCTTTAAGatTtn
>Fam_168_14_3 Nr. of seq. 3 Alignment length(with gaps) = 15 Alignment score = 0.718518
AATAAAtcAACAAt
>Fam_169_13_3 Nr. of seq. 3 Alignment length(with gaps) = 15 Alignment score = 0.659259
GGAATcAgTTACT
>Fam_170_14_3 Nr. of seq. 3 Alignment length(with gaps) = 15 Alignment score = 0.629630
TTTTcTGtTTGtAn
>Fam_171_14_3 Nr. of seq. 3 Alignment length(with gaps) = 15 Alignment score = 0.711111
TGAaTTTTTgTTCc
>Fam_172_14_3 Nr. of seq. 3 Alignment length(with gaps) = 15 Alignment score = 0.666667
TGgaCtCTTAAATn
>Fam_173_15_3 Nr. of seq. 3 Alignment length(with gaps) = 15 Alignment score = 0.651852
tcnAgntaTTtcat
>Fam_174_15_3 Nr. of seq. 3 Alignment length(with gaps) = 15 Alignment score = 0.711111

```
tTTTCgaCtTTTCTt
>Fam_175_14_3  Nr. of seq. 3 Alignment length(with gaps) = 15 Alignment score = 0.711111
TTAGcAaGAATAgG
>Fam_176_15_3  Nr. of seq. 3 Alignment length(with gaps) = 15 Alignment score = 0.829630
tTTGAGAaTTATcTC
>Fam_177_15_3  Nr. of seq. 3 Alignment length(with gaps) = 15 Alignment score = 0.792593
GAagTTTGATtGTGT
>Fam_178_15_3  Nr. of seq. 3 Alignment length(with gaps) = 15 Alignment score = 0.703704
TGAAAAaAAnTtAGa
>Fam_179_15_3  Nr. of seq. 3 Alignment length(with gaps) = 15 Alignment score = 0.674074
CCTaaAgTnGaAGAA
>Fam_180_15_3  Nr. of seq. 3 Alignment length(with gaps) = 15 Alignment score = 0.659259
GAaGAAGaaGtAgcT
>Fam_181_15_3  Nr. of seq. 3 Alignment length(with gaps) = 15 Alignment score = 0.733333
CnGAtGGaAAcCAGA
>Fam_182_15_3  Nr. of seq. 3 Alignment length(with gaps) = 15 Alignment score = 0.762963
aAATAAaaAAaTCAG
>Fam_183_11_3  Nr. of seq. 3 Alignment length(with gaps) = 14 Alignment score = 0.626984
GTTTACnGAAT
>Fam_184_13_3  Nr. of seq. 3 Alignment length(with gaps) = 14 Alignment score = 0.730159
TAAGtCTGAAAAn
>Fam_185_12_3  Nr. of seq. 3 Alignment length(with gaps) = 14 Alignment score = 0.650794
TActTaTTTACT
>Fam_186_12_3  Nr. of seq. 3 Alignment length(with gaps) = 14 Alignment score = 0.738095
TTGaACTCTATT
>Fam_187_14_3  Nr. of seq. 3 Alignment length(with gaps) = 14 Alignment score = 0.682540
TTTAtTnAAATTtt
>Fam_188_14_3  Nr. of seq. 3 Alignment length(with gaps) = 14 Alignment score = 0.714286
AAtAAAAtnGAACT
>Fam_189_13_3  Nr. of seq. 3 Alignment length(with gaps) = 14 Alignment score = 0.690476
atTTTTAcCTGAT
>Fam_190_13_3  Nr. of seq. 3 Alignment length(with gaps) = 14 Alignment score = 0.730159
tATAATTgCaCAG
>Fam_191_14_3  Nr. of seq. 3 Alignment length(with gaps) = 14 Alignment score = 0.698413
ATactTTatAAGTT
>Fam_192_14_3  Nr. of seq. 3 Alignment length(with gaps) = 14 Alignment score = 0.650794
TTTcacCAnTaTCT
>Fam_193_14_3  Nr. of seq. 3 Alignment length(with gaps) = 14 Alignment score = 0.746032
```

AACTggcTATTcTA
>Fam_194_14_3 Nr. of seq. 3 Alignment length(with gaps) = 14 Alignment score = 0.650794
AATCannAATCAAn
>Fam_195_14_3 Nr. of seq. 3 Alignment length(with gaps) = 14 Alignment score = 0.746032
agaTCAGTAAATtA
>Fam_196_14_3 Nr. of seq. 3 Alignment length(with gaps) = 14 Alignment score = 0.809524
TCAAAGtAgAGAAg
>Fam_197_12_3 Nr. of seq. 3 Alignment length(with gaps) = 13 Alignment score = 0.632479
ATTTTTCaatAG
>Fam_198_11_3 Nr. of seq. 3 Alignment length(with gaps) = 13 Alignment score = 0.641026
AaCaTTTTTTG
>Fam_199_12_3 Nr. of seq. 3 Alignment length(with gaps) = 13 Alignment score = 0.649573
TTctcTTACTGa
>Fam_200_11_3 Nr. of seq. 3 Alignment length(with gaps) = 13 Alignment score = 0.675214
GtTTATCTTTT
>Fam_201_13_3 Nr. of seq. 3 Alignment length(with gaps) = 13 Alignment score = 0.709402
TTTTGAATAgnga
>Fam_202_12_3 Nr. of seq. 3 Alignment length(with gaps) = 13 Alignment score = 0.632479
TCCaAnTaCAAG
>Fam_203_13_3 Nr. of seq. 3 Alignment length(with gaps) = 13 Alignment score = 0.632479
aTtnACATaTACt
>Fam_204_12_3 Nr. of seq. 3 Alignment length(with gaps) = 13 Alignment score = 0.641026
ACTTCcGtGgTa
>Fam_205_12_3 Nr. of seq. 3 Alignment length(with gaps) = 13 Alignment score = 0.709402
tGAAGATtGGTa
>Fam_206_12_3 Nr. of seq. 3 Alignment length(with gaps) = 13 Alignment score = 0.666667
cACCTTtTTcTC
>Fam_207_13_3 Nr. of seq. 3 Alignment length(with gaps) = 13 Alignment score = 0.623932
GGAgTggaAAGcA
>Fam_208_12_3 Nr. of seq. 3 Alignment length(with gaps) = 13 Alignment score = 0.675214
TATTTTCCngAa
>Fam_209_13_3 Nr. of seq. 3 Alignment length(with gaps) = 13 Alignment score = 0.794872
tATTcACAAtTGA
>Fam_210_13_3 Nr. of seq. 3 Alignment length(with gaps) = 13 Alignment score = 0.692308
TTAtTgAAAAgnG
>Fam_211_13_3 Nr. of seq. 3 Alignment length(with gaps) = 13 Alignment score = 0.658120
TTTtTATnTgnAC
>Fam_212_10_3 Nr. of seq. 3 Alignment length(with gaps) = 12 Alignment score = 0.657407

ATTTaGGAAT
>Fam_213_11_3 Nr. of seq. 3 Alignment length(with gaps) = 12 Alignment score = 0.638889
AAtTTtGAACt
>Fam_214_12_3 Nr. of seq. 3 Alignment length(with gaps) = 12 Alignment score = 0.675926
GTTTnCatTCAg
>Fam_215_11_3 Nr. of seq. 3 Alignment length(with gaps) = 12 Alignment score = 0.611111
tTGGCTTTcAn
>Fam_216_11_3 Nr. of seq. 3 Alignment length(with gaps) = 12 Alignment score = 0.685185
AtTCAgTATCt
>Fam_217_12_3 Nr. of seq. 3 Alignment length(with gaps) = 12 Alignment score = 0.712963
nAAGGGAtAaTT
>Fam_218_12_3 Nr. of seq. 3 Alignment length(with gaps) = 12 Alignment score = 0.712963
AaGaTAGATAAn
>Fam_219_12_3 Nr. of seq. 3 Alignment length(with gaps) = 12 Alignment score = 0.703704
TgTAAAAgAAnA
>Fam_220_12_3 Nr. of seq. 3 Alignment length(with gaps) = 12 Alignment score = 0.722222
AgAgGaAGgGGA
>Fam_221_12_3 Nr. of seq. 3 Alignment length(with gaps) = 12 Alignment score = 0.703704
GTGtCtGtGTCg
>Fam_222_12_3 Nr. of seq. 3 Alignment length(with gaps) = 12 Alignment score = 0.703704
TGGtTtTGgtTT
>Fam_223_12_3 Nr. of seq. 3 Alignment length(with gaps) = 12 Alignment score = 0.703704
ctTTaAGtTATT
>Fam_224_12_3 Nr. of seq. 3 Alignment length(with gaps) = 12 Alignment score = 0.851852
ATTTgATTCTTc
>Fam_225_10_3 Nr. of seq. 3 Alignment length(with gaps) = 11 Alignment score = 0.606061
TtgtTTGAAA
>Fam_226_10_3 Nr. of seq. 3 Alignment length(with gaps) = 11 Alignment score = 0.737374
AAGGAAAGat
>Fam_227_11_3 Nr. of seq. 3 Alignment length(with gaps) = 11 Alignment score = 0.676768
acAATcAaACT
>Fam_228_10_3 Nr. of seq. 3 Alignment length(with gaps) = 10 Alignment score = 0.644444
TCTTCcctTg
>Fam_229_261_2 Nr. of seq. 2 Alignment length(with gaps) = 261 Alignment score = 0.833972

GTTTCGGTATCCGTGCCGTTTGCATTGCTaACcGTCAGTTTgGCTTCATAAGTCCCTCTGGAAgTGTAaaTaTAAGCAAAGCTTGccgCATTAGAGTCcTCGACcCCGTCA
CCATTAACATCCCAGCTcaaTCtCGaaGTTGCATTCTcCGAAagGTCGGTaAAGAGaACaGTAAGaGGGTAaTAaCCGCTGGTTTTATTCAaTGTGAAGTTTGCTACGGGA
AGAAcAGGAAgTTCTTCTTCCAgCACGGTAATCACAGCA

>Fam_230_159_2 Nr. of seq. 2 Alignment length(with gaps) = 159 Alignment score = 0.781971

TATGACCTTATGATCGGTGaAtcTgaaGGAGTtACATATGGTTATGAGAATACCGGGTCTTCGGacAGCCCGGaGTGGaCTGCGAAaTCTTCcTGGAATAccCCTgaTaTA
GGTacTgcTgCAtCACCAGCTTTgGCCGAcCTTGACgGTGATGGcGAC
>Fam_231_158_2 Nr. of seq. 2 Alignment length(with gaps) = 158 Alignment score = 0.618143

TACGGaGGaAaaGGaGAgGaATatGcCttacGCgGTTGCaATaGCTCaAAAcGGgGAcATaATaGTgaCaGGcgaCACTaACAGcTTCGGCGCTGGTaAtGATGAcGTTTG
GGTTCTcAGaCTTGATnGcaAaTGGaAAcaTcAAGTGGCAaAAaACT
>Fam_232_145_2 Nr. of seq. 2 Alignment length(with gaps) = 145 Alignment score = 0.786207

AAAAAGCACTCaAAATcgAcGcAAAAACTACTCaAAAAaGACCCCGAAAAcGTAgCaTACCAATCaTACGTAGGAAtGACaCTAAACAATTTAGGAAACTTGCTTaaaaAT
ATGGGGagaATTGAAGAcGCGAAAaAaAGGTACG
>Fam_233_141_2 Nr. of seq. 2 Alignment length(with gaps) = 141 Alignment score = 0.806147

TATATGTTGCCaGcacCATCAACcGCAATACCagAcGgtcGaTCGAATTGTCCTGGcTCGtcGCCATgGCTgCCCATTGTTGTCCAGGTGTTTGTGGcTTTGTTCCATACC
TGAATCCTgTgATTAAagGTGTCGGcTACG
>Fam_234_131_2 Nr. of seq. 2 Alignment length(with gaps) = 131 Alignment score = 0.753181

CCATTTTGCCACAGGAAAGCAtGTGAttCAcCAgTATCCGTCTTGACTGAcaCCcACTACCTGCCCATTgTCATTGATTCCaCTGGCAcaGCTATatgCTaCatcCgagcG
TTCCAAgATCAGTCATCaca
>Fam_235_126_2 Nr. of seq. 2 Alignment length(with gaps) = 126 Alignment score = 0.788360

ACCTTGAGGGTCTGATCACCGGAaGCaGATACaGCTcTcaGACCGTCcGGGGTCACcGCCACCGCgCTGACCcAAgcgGAATGaCCTTTCAGcGTCCgcAgCTCcTCGCCC
CTcTCCAGGTCCCAt
>Fam_236_126_2 Nr. of seq. 2 Alignment length(with gaps) = 126 Alignment score = 0.817460

AGTGCTTTTTCGTAaTCTCCCATACTTTCATAGAGTCCTGCGAGATTaTTTAGgGTTGTTGCAACATCTGGaTGTTGcGGcCCcAGaACCTTTTCActtATtTCaAGTGcc
CgTTGAgAAAGTggG
>Fam_237_123_2 Nr. of seq. 2 Alignment length(with gaps) = 123 Alignment score = 0.691057

TCAATTGCTTTTcTcagcTCgCCTAGaTGAaTATATgCTaacCCCAAATcTCCAAGATgATTTCCTTCTCtgCaTacTTATCaTTCnATTTcTCTtGAAATTTTCAACGCC
TGcTcaTAAaAT
>Fam_238_110_2 Nr. of seq. 2 Alignment length(with gaps) = 110 Alignment score = 0.721212
TTCTTCTTTTCTTTGcCCATCCAAATaAGGaACAAgAGCaGAAAGgGCaTaTaaTCTgTGnATAaTCATCTTcAATCcTgGAGGcTGacaTCAAGGGCTTTTTCcATCAC
>Fam_239_104_2 Nr. of seq. 2 Alignment length(with gaps) = 104 Alignment score = 0.637821
AGGGccagCCCCCGGTTGTaCCAGGCcagGGCGaAGccCGGaTCGAtCTcCAGGGCCccgTCGTAgcAcTCGAcCGCCTCCTCgTaCCggCCGAGaaGgCgnCG
>Fam_240_103_2 Nr. of seq. 2 Alignment length(with gaps) = 103 Alignment score = 0.621359
GTTaTGATAAGGCTTTAGAaATaGATcCtAAtaATgagTaATgCaTGGaaTAAcAAAGGATATgCtTTagCaGAaCTTGaAAaATAcgaAGAAGCaaTAGAAt

>Fam_241_102_2 Nr. of seq. 2 Alignment length(with gaps) = 102 Alignment score = 0.686275
ATAAcAAAGGTaaTGCCCTTaaTAAtTTaGGcAaATATaAaGAGgCAATaaaAGCTTATGAtAAAGCCaTAGAGATAaACCCaAAaTATgcCtaTGCaTGGA
>Fam_242_79_2 Nr. of seq. 2 Alignment length(with gaps) = 79 Alignment score = 0.738397
GTTGCATATGAATTgTTcAaTGtTgCCTCgATTatatCCAACCAGTCCACCGACAtAaCaANaaCCNTCAACATTTCCA
>Fam_243_57_2 Nr. of seq. 2 Alignment length(with gaps) = 57 Alignment score = 0.827485
cGCGGTTCTCACTCACTTCGTTCGCTCGCGGGTCACacTcGTTCCCCGCTCGcaGcC
>Fam_244_54_2 Nr. of seq. 2 Alignment length(with gaps) = 54 Alignment score = 0.632716
gAAAAAGAAATACAAGAACAAATAAaAGGAAtgttAAAtcCAgAacCGaAAcTC
>Fam_245_45_2 Nr. of seq. 2 Alignment length(with gaps) = 45 Alignment score = 0.614815
TCgTCGTCCTcgCTGCtTTCCTCcTCcTCAGCcTCcTCaTCacTc
>Fam_246_39_2 Nr. of seq. 2 Alignment length(with gaps) = 39 Alignment score = 0.722222
CCCGAATTGCGcCTCGGGAGTACGCgGTCCTTttcgctg
>Fam_247_36_2 Nr. of seq. 2 Alignment length(with gaps) = 36 Alignment score = 1.000000
AGCGGAAGTTCTGATACAGGAGATTCAGTCTCAGAT
>Fam_248_35_2 Nr. of seq. 2 Alignment length(with gaps) = 35 Alignment score = 0.809524
gTaACCACAGAGGCACAGAGGgaCACAGAGAACgG
>Fam_249_33_2 Nr. of seq. 2 Alignment length(with gaps) = 33 Alignment score = 0.676768
cCCaaCGCGCCTGcTCGGgCCGCGcCGcCgTCG
>Fam_250_33_2 Nr. of seq. 2 Alignment length(with gaps) = 33 Alignment score = 0.969697
CCCCAGTCGGTGACNGTATCGACGGTGTCGCCG
>Fam_251_33_2 Nr. of seq. 2 Alignment length(with gaps) = 33 Alignment score = 0.686869
AaAGTaTATgcCTAAATAggcGaGGAAATAAAG
>Fam_252_33_2 Nr. of seq. 2 Alignment length(with gaps) = 33 Alignment score = 0.737374
GTcGAAGgTCGCCCCaTcAAAACcGGCAtaTCC
>Fam_253_31_2 Nr. of seq. 2 Alignment length(with gaps) = 31 Alignment score = 0.612903
GTTaGAGTTGGGGTgGGTGTagcTgTCtGGa
>Fam_254_31_2 Nr. of seq. 2 Alignment length(with gaps) = 31 Alignment score = 0.913979
CACCTGAaGGcGCTCACGCTCCGGTTCTTCG
>Fam_255_29_2 Nr. of seq. 2 Alignment length(with gaps) = 29 Alignment score = 0.637931
TaTCtTTCTaTAttTTCTaTTTCTTTTTg
>Fam_256_29_2 Nr. of seq. 2 Alignment length(with gaps) = 29 Alignment score = 0.770115
AATAATACCGTTTTTcaCAAATTAaGaaG
>Fam_257_28_2 Nr. of seq. 2 Alignment length(with gaps) = 28 Alignment score = 0.607143
TTgCTTTCTCTacTCgGCTTCTcTcCtt
>Fam_258_28_2 Nr. of seq. 2 Alignment length(with gaps) = 28 Alignment score = 0.648810
GacGAAcACGACGGccACAgCCACCACt
>Fam_259_28_2 Nr. of seq. 2 Alignment length(with gaps) = 28 Alignment score = 0.642857
TTCTTaTTTTTAcTccTTATTaaTTgAC

```
>Fam_260_27_2  Nr. of seq. 2 Alignment length(with gaps) = 27 Alignment score = 0.709877
 TTTcCaGaTTTTAaTTTTAGCGTTTct
>Fam_261_27_2  Nr. of seq. 2 Alignment length(with gaps) = 27 Alignment score = 0.802469
 GCCGCCCATGCCGCCgGGaCCGCCacc
>Fam_262_26_2  Nr. of seq. 2 Alignment length(with gaps) = 26 Alignment score = 0.615385
 CTTCAAtaCaCaTCcTGAGTaTATaT
>Fam_263_26_2  Nr. of seq. 2 Alignment length(with gaps) = 26 Alignment score = 0.621795
 TTCacTTTTAaTCTTCtTgCTTTaAt
>Fam_264_26_2  Nr. of seq. 2 Alignment length(with gaps) = 26 Alignment score = 0.615385
 AGTaAATaAATAAACaTgAaCaAaTA
>Fam_265_26_2  Nr. of seq. 2 Alignment length(with gaps) = 26 Alignment score = 0.602564
 TTagAATTTaaAAAtTAATgAaaAAA
>Fam_266_25_2  Nr. of seq. 2 Alignment length(with gaps) = 25 Alignment score = 0.686667
 TATTTAAAAAaTaAAAAActTAGAa
>Fam_267_25_2  Nr. of seq. 2 Alignment length(with gaps) = 25 Alignment score = 0.606667
 cAccAAAAcaTGAAATagTatAAAa
>Fam_268_25_2  Nr. of seq. 2 Alignment length(with gaps) = 25 Alignment score = 0.620000
 AcACaCCGACGGAaACtGAaAcCGn
>Fam_269_25_2  Nr. of seq. 2 Alignment length(with gaps) = 25 Alignment score = 0.660000
 TaGATTTTGaAaTTGACTTAAcAcg
>Fam_270_25_2  Nr. of seq. 2 Alignment length(with gaps) = 25 Alignment score = 0.633333
 AGaGAAgAaaTTAGAAAAAaaACTt
>Fam_271_24_2  Nr. of seq. 2 Alignment length(with gaps) = 24 Alignment score = 0.666667
 cTCaGTCGGCGTcgGcTCCGGCTC
>Fam_272_24_2  Nr. of seq. 2 Alignment length(with gaps) = 24 Alignment score = 0.694444
 CAATaTCAAACATTACtgAACtAA
>Fam_273_24_2  Nr. of seq. 2 Alignment length(with gaps) = 24 Alignment score = 0.618056
 tTTTAAgGTcaATTATaTTAAcCA
>Fam_274_24_2  Nr. of seq. 2 Alignment length(with gaps) = 24 Alignment score = 0.638889
 AGaAATAAGaTAAgaAAATAtTaC
>Fam_275_24_2  Nr. of seq. 2 Alignment length(with gaps) = 24 Alignment score = 0.611111
 gCggACAACgACcCCGAaCCCaCG
>Fam_276_24_2  Nr. of seq. 2 Alignment length(with gaps) = 24 Alignment score = 1.000000
 GAGGACGAGGAAGAGGACAATGAG
>Fam_277_24_2  Nr. of seq. 2 Alignment length(with gaps) = 24 Alignment score = 0.611111
 TACaGATAcagcAaCTGAAGAcAC
>Fam_278_23_2  Nr. of seq. 2 Alignment length(with gaps) = 23 Alignment score = 0.623188
 TaCAaTTTGAAGAGGATacctTA
```

>Fam_279_23_2 Nr. of seq. 2 Alignment length(with gaps) = 23 Alignment score = 0.637681
TaaGTTAGTACATTGaAtTATat
>Fam_280_23_2 Nr. of seq. 2 Alignment length(with gaps) = 23 Alignment score = 0.695652
TGAGAaTGAtTAGAAaATAAAcc
>Fam_281_23_2 Nr. of seq. 2 Alignment length(with gaps) = 23 Alignment score = 0.652174
GaTTTAGATTagCTcTAAgTTCA
>Fam_282_23_2 Nr. of seq. 2 Alignment length(with gaps) = 23 Alignment score = 0.601449
aTTCgTgTTTTAGAAGtTTGTca
>Fam_283_23_2 Nr. of seq. 2 Alignment length(with gaps) = 23 Alignment score = 0.608696
aTAAAATCAacaAaAGAAaCAAc
>Fam_284_23_2 Nr. of seq. 2 Alignment length(with gaps) = 23 Alignment score = 0.601449
TAcTCTTACCTaaCtTACCTcTc
>Fam_285_23_2 Nr. of seq. 2 Alignment length(with gaps) = 23 Alignment score = 0.630435
GTAAGATTagTAaaAgTTATTGa
>Fam_286_23_2 Nr. of seq. 2 Alignment length(with gaps) = 23 Alignment score = 0.659420
TTTaAcTTTATATTAaAgcAAGc
>Fam_287_23_2 Nr. of seq. 2 Alignment length(with gaps) = 23 Alignment score = 0.739130
CCAGATCAgCGCTTCTGcAcaCG
>Fam_288_23_2 Nr. of seq. 2 Alignment length(with gaps) = 23 Alignment score = 0.797101
AAAAGAAGaAAAAcGAAGAGGGa
>Fam_289_23_2 Nr. of seq. 2 Alignment length(with gaps) = 23 Alignment score = 0.659420
cAgTAAaCAAACAGaGTTGAGat
>Fam_290_23_2 Nr. of seq. 2 Alignment length(with gaps) = 23 Alignment score = 0.681159
cTTACaATTTTcCcAaTTTTTAC
>Fam_291_23_2 Nr. of seq. 2 Alignment length(with gaps) = 23 Alignment score = 0.739130
GAAAAAcAAAAcAGAAgAaAGGT
>Fam_292_23_2 Nr. of seq. 2 Alignment length(with gaps) = 23 Alignment score = 0.710145
ATTAAAAAATaACcTAATaTcaA
>Fam_293_23_2 Nr. of seq. 2 Alignment length(with gaps) = 23 Alignment score = 0.652174
TAaGATAaaAATAaaATCAGAaT
>Fam_294_22_2 Nr. of seq. 2 Alignment length(with gaps) = 22 Alignment score = 0.613636
tCaTTAaAAAAGGAAAtcCAAc
>Fam_295_22_2 Nr. of seq. 2 Alignment length(with gaps) = 22 Alignment score = 0.606061
GaAATcGaaaTAAAATCaAAAA
>Fam_296_22_2 Nr. of seq. 2 Alignment length(with gaps) = 22 Alignment score = 0.696970
CTTTAATTgTTcACtTcTTATT
>Fam_297_22_2 Nr. of seq. 2 Alignment length(with gaps) = 22 Alignment score = 0.674242
TaATAgaTAATTTACAGaTAAt

```
>Fam_298_22_2  Nr. of seq. 2 Alignment length(with gaps) = 22 Alignment score = 0.666667
 gTCGtGGaGTGTCTGTGCcGTg
>Fam_299_22_2  Nr. of seq. 2 Alignment length(with gaps) = 22 Alignment score = 0.666667
 aCTTTTcCTTcTAtTTTcTTCC
>Fam_300_22_2  Nr. of seq. 2 Alignment length(with gaps) = 22 Alignment score = 0.666667
 aGAATATTGAaAGAGTAtTaaA
>Fam_301_22_2  Nr. of seq. 2 Alignment length(with gaps) = 22 Alignment score = 0.613636
 TAaGTCTCATgTTaaATcTTAa
>Fam_302_22_2  Nr. of seq. 2 Alignment length(with gaps) = 22 Alignment score = 0.643939
 AATTCaAAAAGATcTgAaAaGa
>Fam_303_22_2  Nr. of seq. 2 Alignment length(with gaps) = 22 Alignment score = 0.606061
 TccTTCCAgAAGATTAcTTcgT
>Fam_304_22_2  Nr. of seq. 2 Alignment length(with gaps) = 22 Alignment score = 0.606061
 aaTaCTgaAAAcTTTCTATAAT
>Fam_305_22_2  Nr. of seq. 2 Alignment length(with gaps) = 22 Alignment score = 0.606061
 AAaTCtAAGGaAaaTTTAAGaG
>Fam_306_22_2  Nr. of seq. 2 Alignment length(with gaps) = 22 Alignment score = 0.643939
 TCTTTaAGAaAacTcATTCTAc
>Fam_307_22_2  Nr. of seq. 2 Alignment length(with gaps) = 22 Alignment score = 0.636364
 TcGTGATGTGGcgGTGagGGgG
>Fam_308_22_2  Nr. of seq. 2 Alignment length(with gaps) = 22 Alignment score = 0.636364
 AAATaaAgaGTaGAATTATaAA
>Fam_309_22_2  Nr. of seq. 2 Alignment length(with gaps) = 22 Alignment score = 0.696970
 TAAAAAcaTATTagATTAATcT
>Fam_310_21_2  Nr. of seq. 2 Alignment length(with gaps) = 21 Alignment score = 0.603175
 GAAcCTGAAGtAACCgAGacA
>Fam_311_21_2  Nr. of seq. 2 Alignment length(with gaps) = 21 Alignment score = 0.658730
 AAcCtaAGAAACCCgAAATTa
>Fam_312_21_2  Nr. of seq. 2 Alignment length(with gaps) = 21 Alignment score = 0.658730
 TCATTTTCTAcAaTTccTGTt
>Fam_313_21_2  Nr. of seq. 2 Alignment length(with gaps) = 21 Alignment score = 0.611111
 ATAAcCTGAAtAGaTTTGcat
>Fam_314_21_2  Nr. of seq. 2 Alignment length(with gaps) = 21 Alignment score = 0.626984
 GGATAAcCTcAAcACTTTaAa
>Fam_315_21_2  Nr. of seq. 2 Alignment length(with gaps) = 21 Alignment score = 0.626984
 taTAATTaAGTTTaAACTaAG
>Fam_316_21_2  Nr. of seq. 2 Alignment length(with gaps) = 21 Alignment score = 0.603175
 ggTaCTGAccTGAGAAAATCc
```

```
>Fam_317_21_2  Nr. of seq. 2 Alignment length(with gaps) = 21 Alignment score = 0.698413
 CaTTTCTTTCTaCTTTcTTct
>Fam_318_21_2  Nr. of seq. 2 Alignment length(with gaps) = 21 Alignment score = 0.722222
 aTTTTTACtTATTTTTATTat
>Fam_319_21_2  Nr. of seq. 2 Alignment length(with gaps) = 21 Alignment score = 0.650794
 CTTaaTTATTTCtTCAGaTaC
>Fam_320_21_2  Nr. of seq. 2 Alignment length(with gaps) = 21 Alignment score = 0.626984
 aTACTTaCTGcCTTCTagCCt
>Fam_321_21_2  Nr. of seq. 2 Alignment length(with gaps) = 21 Alignment score = 0.650794
 AAaAACAGAcaGGAAGcTaAG
>Fam_322_21_2  Nr. of seq. 2 Alignment length(with gaps) = 21 Alignment score = 0.619048
 CGacGACGaCGGCGAACaaaC
>Fam_323_21_2  Nr. of seq. 2 Alignment length(with gaps) = 21 Alignment score = 0.682540
 CGcCcGTCGcGgTCGTCGaGT
>Fam_324_21_2  Nr. of seq. 2 Alignment length(with gaps) = 21 Alignment score = 0.682540
 AACGAcACcGaGaCcGCTGAC
>Fam_325_21_2  Nr. of seq. 2 Alignment length(with gaps) = 21 Alignment score = 0.873016
 TGCTgGAgAAACAGGATCAGA
>Fam_326_21_2  Nr. of seq. 2 Alignment length(with gaps) = 21 Alignment score = 0.682540
 TTTaAATaAaaAATgGTATAC
>Fam_327_21_2  Nr. of seq. 2 Alignment length(with gaps) = 21 Alignment score = 0.634921
 cTCTTCAaCTaCaTCcAAgTC
>Fam_328_21_2  Nr. of seq. 2 Alignment length(with gaps) = 21 Alignment score = 0.682540
 CcGTTAcaTTcTCCTCaGGTA
>Fam_329_20_2  Nr. of seq. 2 Alignment length(with gaps) = 20 Alignment score = 0.641667
 TAaTCTTTTTAAATCcagcg
>Fam_330_20_2  Nr. of seq. 2 Alignment length(with gaps) = 20 Alignment score = 0.750000
 AATAAATaTAATcAAAaTAT
>Fam_331_20_2  Nr. of seq. 2 Alignment length(with gaps) = 20 Alignment score = 0.716667
 CCaTGCAATATcAATAATca
>Fam_332_20_2  Nr. of seq. 2 Alignment length(with gaps) = 20 Alignment score = 0.641667
 AcTTaCTGgTTaATTCTTCa
>Fam_333_20_2  Nr. of seq. 2 Alignment length(with gaps) = 20 Alignment score = 0.683333
 aAAAATAAcCaGAATTAGnt
>Fam_334_20_2  Nr. of seq. 2 Alignment length(with gaps) = 20 Alignment score = 0.700000
 ATCaAATTTAATaCCAgGAG
>Fam_335_20_2  Nr. of seq. 2 Alignment length(with gaps) = 20 Alignment score = 0.608333
 AAAAACtGAACaTgATaCGa
```

```
>Fam_336_20_2  Nr. of seq. 2 Alignment length(with gaps) = 20 Alignment score = 0.633333
 aaAgTcAAAAAAAGaATATG
>Fam_337_20_2  Nr. of seq. 2 Alignment length(with gaps) = 20 Alignment score = 0.608333
 aaAAATTCATaTATAaAcAg
>Fam_338_20_2  Nr. of seq. 2 Alignment length(with gaps) = 20 Alignment score = 0.666667
 TAAAtATATaaTTTAAtAGT
>Fam_339_20_2  Nr. of seq. 2 Alignment length(with gaps) = 20 Alignment score = 0.633333
 aAAAAaGAgcTAAGAaGCAA
>Fam_340_20_2  Nr. of seq. 2 Alignment length(with gaps) = 20 Alignment score = 0.666667
 TTaTaTGGAAGTTTaGTGaT
>Fam_341_20_2  Nr. of seq. 2 Alignment length(with gaps) = 20 Alignment score = 0.666667
 TAAAAATcATGcAACgTATg
>Fam_342_20_2  Nr. of seq. 2 Alignment length(with gaps) = 20 Alignment score = 0.700000
 TaAAAgTCAGcTaGAATAAA
>Fam_343_20_2  Nr. of seq. 2 Alignment length(with gaps) = 20 Alignment score = 0.766667
 aTAAAAaAAGGaTAAAAGAA
>Fam_344_20_2  Nr. of seq. 2 Alignment length(with gaps) = 20 Alignment score = 0.675000
 TACaAaATTcAAGTCAATat
>Fam_345_20_2  Nr. of seq. 2 Alignment length(with gaps) = 20 Alignment score = 0.666667
 TTTTTAagTTTaTTAacTAA
>Fam_346_20_2  Nr. of seq. 2 Alignment length(with gaps) = 20 Alignment score = 0.666667
 TTgaTaCTTaTTaTGTATAT
>Fam_347_19_2  Nr. of seq. 2 Alignment length(with gaps) = 19 Alignment score = 0.657895
 AATAAtgCAAATAATCtGa
>Fam_348_19_2  Nr. of seq. 2 Alignment length(with gaps) = 19 Alignment score = 0.649123
 GGATAAaAGaATAtaTTCA
>Fam_349_19_2  Nr. of seq. 2 Alignment length(with gaps) = 19 Alignment score = 0.640351
 aTCATTTTAcCTTaAtTCt
>Fam_350_19_2  Nr. of seq. 2 Alignment length(with gaps) = 19 Alignment score = 0.631579
 TTGAAAGaAAgCtCATTgc
>Fam_351_19_2  Nr. of seq. 2 Alignment length(with gaps) = 19 Alignment score = 0.622807
 TTTcAGATTACAcGaTaAc
>Fam_352_19_2  Nr. of seq. 2 Alignment length(with gaps) = 19 Alignment score = 0.692982
 aTCtCATTTTTGcAATTCt
>Fam_353_19_2  Nr. of seq. 2 Alignment length(with gaps) = 19 Alignment score = 0.692982
 TAATTAAAATaAAcCTAaa
>Fam_354_19_2  Nr. of seq. 2 Alignment length(with gaps) = 19 Alignment score = 0.692982
 TTcAAaAATTAAcTTAAAa
```

>Fam_355_19_2 Nr. of seq. 2 Alignment length(with gaps) = 19 Alignment score = 0.649123
AAAgGTaATAATcTaTTTA
>Fam_356_19_2 Nr. of seq. 2 Alignment length(with gaps) = 19 Alignment score = 0.666667
TCTcTTTCTTaaGCTTAtt
>Fam_357_19_2 Nr. of seq. 2 Alignment length(with gaps) = 19 Alignment score = 0.614035
ATTCAGGagTTAATTacTc
>Fam_358_19_2 Nr. of seq. 2 Alignment length(with gaps) = 19 Alignment score = 0.622807
gCCCGaCTCGcCTGcTCCc
>Fam_359_19_2 Nr. of seq. 2 Alignment length(with gaps) = 19 Alignment score = 0.614035
CCcCTCCaaCTaCTCCaCC
>Fam_360_19_2 Nr. of seq. 2 Alignment length(with gaps) = 19 Alignment score = 0.684211
aTATCAgTaAATTcAAATT
>Fam_361_19_2 Nr. of seq. 2 Alignment length(with gaps) = 19 Alignment score = 0.622807
gCTGtTGGaGTTaCTTCTa
>Fam_362_19_2 Nr. of seq. 2 Alignment length(with gaps) = 19 Alignment score = 0.692982
aAGgAaTAGAAATaATAAG
>Fam_363_19_2 Nr. of seq. 2 Alignment length(with gaps) = 19 Alignment score = 0.614035
TAAgTGCAGcAACcTGaAc
>Fam_364_19_2 Nr. of seq. 2 Alignment length(with gaps) = 19 Alignment score = 0.649123
CGAgaTTTCaCAcACTTAA
>Fam_365_19_2 Nr. of seq. 2 Alignment length(with gaps) = 19 Alignment score = 0.719298
TaAGTTaAATAAtATTAAG
>Fam_366_19_2 Nr. of seq. 2 Alignment length(with gaps) = 19 Alignment score = 0.649123
aTTCaAATaACTCCAgATG
>Fam_367_19_2 Nr. of seq. 2 Alignment length(with gaps) = 19 Alignment score = 0.614035
acTGAgAaACATTGAaAAT
>Fam_368_19_2 Nr. of seq. 2 Alignment length(with gaps) = 19 Alignment score = 0.684211
AAAAATGGGaAAtCAaGAc
>Fam_369_19_2 Nr. of seq. 2 Alignment length(with gaps) = 19 Alignment score = 0.684211
TcaGAAAAcAgGAAAATAG
>Fam_370_19_2 Nr. of seq. 2 Alignment length(with gaps) = 19 Alignment score = 0.684211
GATTAagTAAAtcTTGATT
>Fam_371_19_2 Nr. of seq. 2 Alignment length(with gaps) = 19 Alignment score = 0.614035
TATTGgcACATTATaTcaT
>Fam_372_19_2 Nr. of seq. 2 Alignment length(with gaps) = 19 Alignment score = 0.684211
ATTTCaCAaAcTATTaTAT
>Fam_373_19_2 Nr. of seq. 2 Alignment length(with gaps) = 19 Alignment score = 0.614035
AGaAAACaCAGaGAaAcGG

>Fam_374_19_2 Nr. of seq. 2 Alignment length(with gaps) = 19 Alignment score = 0.614035
TgCTcATAtaTTATCCAaG
>Fam_375_19_2 Nr. of seq. 2 Alignment length(with gaps) = 19 Alignment score = 0.789474
TTTAAAACTTTCTaAaGaG
>Fam_376_19_2 Nr. of seq. 2 Alignment length(with gaps) = 19 Alignment score = 0.929825
AGTAATTTAGCTATCAGTa
>Fam_377_19_2 Nr. of seq. 2 Alignment length(with gaps) = 19 Alignment score = 0.649123
TTTGCcTTgAcTTTaCaTA
>Fam_378_19_2 Nr. of seq. 2 Alignment length(with gaps) = 19 Alignment score = 0.649123
TTGacgAAaATCTcTAAAT
>Fam_379_18_2 Nr. of seq. 2 Alignment length(with gaps) = 18 Alignment score = 0.638889
AACGAGAgCgCCAGcGCc
>Fam_380_18_2 Nr. of seq. 2 Alignment length(with gaps) = 18 Alignment score = 0.638889
AATTGTaaATTgTGATTc
>Fam_381_18_2 Nr. of seq. 2 Alignment length(with gaps) = 18 Alignment score = 0.731481
ATTATCCaCTGAAATatc
>Fam_382_18_2 Nr. of seq. 2 Alignment length(with gaps) = 18 Alignment score = 0.685185
TTCTgTAaTTTAGGGAtg
>Fam_383_18_2 Nr. of seq. 2 Alignment length(with gaps) = 18 Alignment score = 0.638889
GAACTGTTaaAGCAtAAc
>Fam_384_18_2 Nr. of seq. 2 Alignment length(with gaps) = 18 Alignment score = 0.611111
aATCCAcAATaGAAACta
>Fam_385_18_2 Nr. of seq. 2 Alignment length(with gaps) = 18 Alignment score = 0.694444
ATTAAaAGATCAtgAAAt
>Fam_386_18_2 Nr. of seq. 2 Alignment length(with gaps) = 18 Alignment score = 0.675926
TTTCtTCAGaTGTTTaCg
>Fam_387_18_2 Nr. of seq. 2 Alignment length(with gaps) = 18 Alignment score = 0.648148
AAAACaaTAGAcAATGtc
>Fam_388_18_2 Nr. of seq. 2 Alignment length(with gaps) = 18 Alignment score = 0.611111
TATGAAAAaTaGGAcTtg
>Fam_389_18_2 Nr. of seq. 2 Alignment length(with gaps) = 18 Alignment score = 0.629630
aAGTTAGAgGaTgAGGAT
>Fam_390_18_2 Nr. of seq. 2 Alignment length(with gaps) = 18 Alignment score = 0.629630
AtTATaATGaACTCAcTA
>Fam_391_18_2 Nr. of seq. 2 Alignment length(with gaps) = 18 Alignment score = 0.601852
AAcTAtcGGAAcTACGGa
>Fam_392_18_2 Nr. of seq. 2 Alignment length(with gaps) = 18 Alignment score = 0.638889
cAgaTTTCTGGAAAaATa

>Fam_393_18_2 Nr. of seq. 2 Alignment length(with gaps) = 18 Alignment score = 0.666667
TTTcAcaTTACTCTGtCC
>Fam_394_18_2 Nr. of seq. 2 Alignment length(with gaps) = 18 Alignment score = 0.675926
TAGAgAaAATACTaAATt
>Fam_395_18_2 Nr. of seq. 2 Alignment length(with gaps) = 18 Alignment score = 0.666667
TTCaAAcaTAaCAGATCC
>Fam_396_18_2 Nr. of seq. 2 Alignment length(with gaps) = 18 Alignment score = 0.601852
ACACTgACtATcaTTTAg
>Fam_397_18_2 Nr. of seq. 2 Alignment length(with gaps) = 18 Alignment score = 0.703704
TAGAAACtTGAgTaTAAT
>Fam_398_18_2 Nr. of seq. 2 Alignment length(with gaps) = 18 Alignment score = 0.638889
AaTAAcATTaCAcTATTc
>Fam_399_18_2 Nr. of seq. 2 Alignment length(with gaps) = 18 Alignment score = 0.666667
gACTgATTTaTaGTTCTA
>Fam_400_18_2 Nr. of seq. 2 Alignment length(with gaps) = 18 Alignment score = 0.638889
GAATAaaGAaTGGATTct
>Fam_401_18_2 Nr. of seq. 2 Alignment length(with gaps) = 18 Alignment score = 0.666667
AAgTAACATATTcCcTgA
>Fam_402_18_2 Nr. of seq. 2 Alignment length(with gaps) = 18 Alignment score = 0.638889
GTTCTATaaaATCTaCCt
>Fam_403_18_2 Nr. of seq. 2 Alignment length(with gaps) = 18 Alignment score = 0.638889
aGATGAGaTTTGTcATac
>Fam_404_18_2 Nr. of seq. 2 Alignment length(with gaps) = 18 Alignment score = 0.759259
tTTTTAtTTTTTCACTaC
>Fam_405_18_2 Nr. of seq. 2 Alignment length(with gaps) = 18 Alignment score = 0.666667
TTcTCAGAAgATAcTaAG
>Fam_406_18_2 Nr. of seq. 2 Alignment length(with gaps) = 18 Alignment score = 0.703704
TaCcTCCTCTTCAGcCgG
>Fam_407_18_2 Nr. of seq. 2 Alignment length(with gaps) = 18 Alignment score = 0.703704
ACACCAgAAAACaAaaAA
>Fam_408_18_2 Nr. of seq. 2 Alignment length(with gaps) = 18 Alignment score = 0.703704
TATaTTcGTgaTCTTGAT
>Fam_409_18_2 Nr. of seq. 2 Alignment length(with gaps) = 18 Alignment score = 0.629630
AaTCTAAAaAacAGTcTG
>Fam_410_18_2 Nr. of seq. 2 Alignment length(with gaps) = 18 Alignment score = 0.703704
GATACAGaAGAcACgGaT
>Fam_411_18_2 Nr. of seq. 2 Alignment length(with gaps) = 18 Alignment score = 0.629630
ATcCGGgAGaTGgAAaGT

```
>Fam_412_18_2  Nr. of seq. 2 Alignment length(with gaps) = 18 Alignment score = 0.703704
 aTaCCGAcGCGGACaACG
>Fam_413_18_2  Nr. of seq. 2 Alignment length(with gaps) = 18 Alignment score = 1.000000
 TCTACTCCAGTTAATACA
>Fam_414_18_2  Nr. of seq. 2 Alignment length(with gaps) = 18 Alignment score = 0.851852
 TAATCTTaGAATTCCTTc
>Fam_415_18_2  Nr. of seq. 2 Alignment length(with gaps) = 18 Alignment score = 0.629630
 TgGTTAATCcaTacTATC
>Fam_416_18_2  Nr. of seq. 2 Alignment length(with gaps) = 18 Alignment score = 0.629630
 ATTTATCcTAAccTTcTa
>Fam_417_18_2  Nr. of seq. 2 Alignment length(with gaps) = 18 Alignment score = 0.648148
 AaAGgAAaCagAGAAAAC
>Fam_418_18_2  Nr. of seq. 2 Alignment length(with gaps) = 18 Alignment score = 0.629630
 ATCTCACTaACaacTAAc
>Fam_419_18_2  Nr. of seq. 2 Alignment length(with gaps) = 18 Alignment score = 0.629630
 ACTCCAGcTacGGaAGaA
>Fam_420_18_2  Nr. of seq. 2 Alignment length(with gaps) = 18 Alignment score = 0.777778
 TCcTcAATCTCAGcTTTT
>Fam_421_18_2  Nr. of seq. 2 Alignment length(with gaps) = 18 Alignment score = 0.777778
 AaTAGAaCcGATAATGAG
>Fam_422_17_2  Nr. of seq. 2 Alignment length(with gaps) = 17 Alignment score = 0.666667
 ACTGGAAATcaCTTTtt
>Fam_423_17_2  Nr. of seq. 2 Alignment length(with gaps) = 17 Alignment score = 0.627451
 TCaGAGCCaCTgTCCta
>Fam_424_17_2  Nr. of seq. 2 Alignment length(with gaps) = 17 Alignment score = 0.607843
 CCaTcGTGTTTCtaATC
>Fam_425_17_2  Nr. of seq. 2 Alignment length(with gaps) = 17 Alignment score = 0.735294
 gAAAAAATAGtTGAGAt
>Fam_426_17_2  Nr. of seq. 2 Alignment length(with gaps) = 17 Alignment score = 0.705882
 AgTTTAAGGAAGaAGta
>Fam_427_17_2  Nr. of seq. 2 Alignment length(with gaps) = 17 Alignment score = 0.735294
 tTTCTTTTaATTCCTTc
>Fam_428_17_2  Nr. of seq. 2 Alignment length(with gaps) = 17 Alignment score = 0.735294
 ATAGaCTAAAAtTCCAg
>Fam_429_17_2  Nr. of seq. 2 Alignment length(with gaps) = 17 Alignment score = 0.627451
 CcTTTAgTTTATCTgTa
>Fam_430_17_2  Nr. of seq. 2 Alignment length(with gaps) = 17 Alignment score = 0.705882
 GAAAAATAGAGAggAca
```

>Fam_431_17_2 Nr. of seq. 2 Alignment length(with gaps) = 17 Alignment score = 0.666667
cCAAAtGAGGAGAATta

>Fam_432_17_2 Nr. of seq. 2 Alignment length(with gaps) = 17 Alignment score = 0.696078
aATTTCTTcAGAGcTAT

>Fam_433_17_2 Nr. of seq. 2 Alignment length(with gaps) = 17 Alignment score = 0.725490
TTAACAGCtaCTTATaG

>Fam_434_17_2 Nr. of seq. 2 Alignment length(with gaps) = 17 Alignment score = 0.656863
ATTTGAgTTAGTgTaAt

>Fam_435_17_2 Nr. of seq. 2 Alignment length(with gaps) = 17 Alignment score = 0.735294
AAAaTAAGATTGtGGTc

>Fam_436_17_2 Nr. of seq. 2 Alignment length(with gaps) = 17 Alignment score = 0.647059
caAAGaTAAAGcTCCTA

>Fam_437_17_2 Nr. of seq. 2 Alignment length(with gaps) = 17 Alignment score = 0.686275
TAcAAGAGAAGtATCaT

>Fam_438_17_2 Nr. of seq. 2 Alignment length(with gaps) = 17 Alignment score = 0.656863
TTTTTAAtTCAAgaGTa

>Fam_439_17_2 Nr. of seq. 2 Alignment length(with gaps) = 17 Alignment score = 0.725490
TTACTTacTaTTACTGT

>Fam_440_17_2 Nr. of seq. 2 Alignment length(with gaps) = 17 Alignment score = 0.725490
AGaTAAGAGAGTTAagA

>Fam_441_17_2 Nr. of seq. 2 Alignment length(with gaps) = 17 Alignment score = 0.686275
GAcTATGTGaATTCgTA

>Fam_442_17_2 Nr. of seq. 2 Alignment length(with gaps) = 17 Alignment score = 0.725490
TGTAAAaTCATTacTCT

>Fam_443_17_2 Nr. of seq. 2 Alignment length(with gaps) = 17 Alignment score = 0.656863
cCTAAcTaATTGaCTTT

>Fam_444_17_2 Nr. of seq. 2 Alignment length(with gaps) = 17 Alignment score = 0.647059
TAAAAccTAAAgAaGAG

>Fam_445_17_2 Nr. of seq. 2 Alignment length(with gaps) = 17 Alignment score = 0.774510
AAGGAAGTAaAATcAAt

>Fam_446_17_2 Nr. of seq. 2 Alignment length(with gaps) = 17 Alignment score = 0.696078
AcTCTCAAAaTCTCAat

>Fam_447_17_2 Nr. of seq. 2 Alignment length(with gaps) = 17 Alignment score = 0.725490
TATTTaGTTCAAAtTaA

>Fam_448_17_2 Nr. of seq. 2 Alignment length(with gaps) = 17 Alignment score = 0.647059
TTCAgCTTTAAcaaTCT

>Fam_449_17_2 Nr. of seq. 2 Alignment length(with gaps) = 17 Alignment score = 0.696078
ATGAAaTTaAGAGTgAa

```
>Fam_450_17_2  Nr. of seq. 2 Alignment length(with gaps) = 17 Alignment score = 0.686275
 TGGATGaAATCTGaTAc
>Fam_451_17_2  Nr. of seq. 2 Alignment length(with gaps) = 17 Alignment score = 0.647059
 CTGtTCTTCAaAATaAc
>Fam_452_17_2  Nr. of seq. 2 Alignment length(with gaps) = 17 Alignment score = 0.725490
 TCTTCaAATTcATAGgC
>Fam_453_17_2  Nr. of seq. 2 Alignment length(with gaps) = 17 Alignment score = 0.647059
 ACTTAGAACTAcgTAaa
>Fam_454_17_2  Nr. of seq. 2 Alignment length(with gaps) = 17 Alignment score = 0.725490
 TAACATATcAAaCGAaT
>Fam_455_17_2  Nr. of seq. 2 Alignment length(with gaps) = 17 Alignment score = 0.647059
 AAGTAaGGAGaAACAca
>Fam_456_17_2  Nr. of seq. 2 Alignment length(with gaps) = 17 Alignment score = 0.725490
 aGTTTCAATcATAAtTC
>Fam_457_17_2  Nr. of seq. 2 Alignment length(with gaps) = 17 Alignment score = 0.764706
 aAcTTaCTTTTTATCTT
>Fam_458_17_2  Nr. of seq. 2 Alignment length(with gaps) = 17 Alignment score = 0.686275
 TAcCTTTTTgaTTTCaC
>Fam_459_17_2  Nr. of seq. 2 Alignment length(with gaps) = 17 Alignment score = 0.607843
 ATCaATTATAaAaaGaC
>Fam_460_17_2  Nr. of seq. 2 Alignment length(with gaps) = 17 Alignment score = 0.921569
 TTAAACAAACCTTAaAC
>Fam_461_17_2  Nr. of seq. 2 Alignment length(with gaps) = 17 Alignment score = 0.686275
 AGcTACAaAAATgaATT
>Fam_462_17_2  Nr. of seq. 2 Alignment length(with gaps) = 17 Alignment score = 0.607843
 AcAcAGccAaAACTTCT
>Fam_463_17_2  Nr. of seq. 2 Alignment length(with gaps) = 17 Alignment score = 0.686275
 TGTcATTAACTgAATac
>Fam_464_17_2  Nr. of seq. 2 Alignment length(with gaps) = 17 Alignment score = 0.686275
 TTTTCTAaaTaGAaATC
>Fam_465_17_2  Nr. of seq. 2 Alignment length(with gaps) = 17 Alignment score = 0.607843
 aaATCAgATTaATGAcG
>Fam_466_16_2  Nr. of seq. 2 Alignment length(with gaps) = 16 Alignment score = 0.625000
 aTaacaTAccacctcc
>Fam_467_16_2  Nr. of seq. 2 Alignment length(with gaps) = 16 Alignment score = 0.635417
 CATAAcCTTGACatCa
>Fam_468_16_2  Nr. of seq. 2 Alignment length(with gaps) = 16 Alignment score = 0.729167
 ATTaTAGAATGTAAga
```

>Fam_469_16_2 Nr. of seq. 2 Alignment length(with gaps) = 16 Alignment score = 0.677083
cGGTTTAcATTaAATC
>Fam_470_16_2 Nr. of seq. 2 Alignment length(with gaps) = 16 Alignment score = 0.718750
TTCAAATGAgtAATAa
>Fam_471_16_2 Nr. of seq. 2 Alignment length(with gaps) = 16 Alignment score = 0.645833
TTGcGaGAATTTCTag
>Fam_472_16_2 Nr. of seq. 2 Alignment length(with gaps) = 16 Alignment score = 0.687500
taGACAAgAATTcATG
>Fam_473_16_2 Nr. of seq. 2 Alignment length(with gaps) = 16 Alignment score = 0.718750
GcTTTTTAtATTTTGa
>Fam_474_16_2 Nr. of seq. 2 Alignment length(with gaps) = 16 Alignment score = 0.687500
AAAACCaTTTaCTCaa
>Fam_475_16_2 Nr. of seq. 2 Alignment length(with gaps) = 16 Alignment score = 0.718750
AAGACGTTTTCaTcAa
>Fam_476_16_2 Nr. of seq. 2 Alignment length(with gaps) = 16 Alignment score = 0.718750
TTAGACTgTTaCAAGc
>Fam_477_16_2 Nr. of seq. 2 Alignment length(with gaps) = 16 Alignment score = 0.687500
AAAACCTGaAaAAGgt
>Fam_478_16_2 Nr. of seq. 2 Alignment length(with gaps) = 16 Alignment score = 0.708333
TGaTTCGGTGGtTcGG
>Fam_479_16_2 Nr. of seq. 2 Alignment length(with gaps) = 16 Alignment score = 0.666667
GAAcTAAcACaAACTA
>Fam_480_16_2 Nr. of seq. 2 Alignment length(with gaps) = 16 Alignment score = 0.625000
cTCTGaaTGGTAGTAc
>Fam_481_16_2 Nr. of seq. 2 Alignment length(with gaps) = 16 Alignment score = 0.677083
TATTgTGcAGTAAcAt
>Fam_482_16_2 Nr. of seq. 2 Alignment length(with gaps) = 16 Alignment score = 0.677083
TccTTGTATTcCAGAt
>Fam_483_16_2 Nr. of seq. 2 Alignment length(with gaps) = 16 Alignment score = 0.625000
CTTAaCtCaAATcAAG
>Fam_484_16_2 Nr. of seq. 2 Alignment length(with gaps) = 16 Alignment score = 0.708333
cGATGTTATcTTTTAa
>Fam_485_16_2 Nr. of seq. 2 Alignment length(with gaps) = 16 Alignment score = 0.791667
ATTGATTTGTaTTgAG
>Fam_486_16_2 Nr. of seq. 2 Alignment length(with gaps) = 16 Alignment score = 0.708333
TGTAaTTCTCTaTTCa
>Fam_487_16_2 Nr. of seq. 2 Alignment length(with gaps) = 16 Alignment score = 0.718750
taTTCATAGAtCTTGA

>Fam_488_16_2 Nr. of seq. 2 Alignment length(with gaps) = 16 Alignment score = 0.677083
tCTGAaTTTCAaAAaA
>Fam_489_16_2 Nr. of seq. 2 Alignment length(with gaps) = 16 Alignment score = 0.708333
TTaAAAAAACATaTTa
>Fam_490_16_2 Nr. of seq. 2 Alignment length(with gaps) = 16 Alignment score = 0.677083
AAcCTATTGAaATTga
>Fam_491_16_2 Nr. of seq. 2 Alignment length(with gaps) = 16 Alignment score = 0.760417
ATTTgcTTCCTGCTTa
>Fam_492_16_2 Nr. of seq. 2 Alignment length(with gaps) = 16 Alignment score = 0.760417
ACAATaAAAGGAAaAc
>Fam_493_16_2 Nr. of seq. 2 Alignment length(with gaps) = 16 Alignment score = 0.666667
AATAAgAAAGcAgAAT
>Fam_494_16_2 Nr. of seq. 2 Alignment length(with gaps) = 16 Alignment score = 0.708333
ATaAATCAAAgTAAAa
>Fam_495_16_2 Nr. of seq. 2 Alignment length(with gaps) = 16 Alignment score = 0.708333
AAAAGAGTAgAgAAaG
>Fam_496_16_2 Nr. of seq. 2 Alignment length(with gaps) = 16 Alignment score = 0.760417
AAAaTGAAATGAAgTc
>Fam_497_16_2 Nr. of seq. 2 Alignment length(with gaps) = 16 Alignment score = 0.708333
AATATAAAaTGacACA
>Fam_498_16_2 Nr. of seq. 2 Alignment length(with gaps) = 16 Alignment score = 0.635417
ATTTCAaTATaaCCGg
>Fam_499_16_2 Nr. of seq. 2 Alignment length(with gaps) = 16 Alignment score = 0.760417
AcAaTTACCTGAAATt
>Fam_500_16_2 Nr. of seq. 2 Alignment length(with gaps) = 16 Alignment score = 0.677083
TGAGGaAaTcAATCCc
>Fam_501_16_2 Nr. of seq. 2 Alignment length(with gaps) = 16 Alignment score = 0.666667
cTACcAAAaAAaCTAG
>Fam_502_16_2 Nr. of seq. 2 Alignment length(with gaps) = 16 Alignment score = 0.666667
GGaTTaGaTAGTTaAT
>Fam_503_16_2 Nr. of seq. 2 Alignment length(with gaps) = 16 Alignment score = 0.666667
aTTTaCcgGTTAACGG
>Fam_504_16_2 Nr. of seq. 2 Alignment length(with gaps) = 16 Alignment score = 0.666667
ATaTaTaaGATCTGAA
>Fam_505_16_2 Nr. of seq. 2 Alignment length(with gaps) = 16 Alignment score = 0.750000
GAAGaAgTCTATaCAG
>Fam_506_16_2 Nr. of seq. 2 Alignment length(with gaps) = 16 Alignment score = 0.750000
CAaTAATCTAGaaCTC

>Fam_507_16_2 Nr. of seq. 2 Alignment length(with gaps) = 16 Alignment score = 0.833333
TGACTTCaTACCTTAc
>Fam_508_16_2 Nr. of seq. 2 Alignment length(with gaps) = 16 Alignment score = 0.666667
cTTTTATTGAaACaAg
>Fam_509_16_2 Nr. of seq. 2 Alignment length(with gaps) = 16 Alignment score = 0.833333
ATTaGCTAAaTTATGG
>Fam_510_16_2 Nr. of seq. 2 Alignment length(with gaps) = 16 Alignment score = 0.750000
AcTTATaTCGAAcTTT
>Fam_511_16_2 Nr. of seq. 2 Alignment length(with gaps) = 16 Alignment score = 0.750000
TTAaCcAGCTCTAAAa
>Fam_512_16_2 Nr. of seq. 2 Alignment length(with gaps) = 16 Alignment score = 0.666667
TTAaTCAgAACTTcaG
>Fam_513_16_2 Nr. of seq. 2 Alignment length(with gaps) = 16 Alignment score = 0.666667
TTCaTTAgaTaTATTC
>Fam_514_16_2 Nr. of seq. 2 Alignment length(with gaps) = 16 Alignment score = 0.854167
CTCTAATCAAaAGTcA
>Fam_515_16_2 Nr. of seq. 2 Alignment length(with gaps) = 16 Alignment score = 0.916667
TCCcAACCGCGCTCGC
>Fam_516_15_2 Nr. of seq. 2 Alignment length(with gaps) = 15 Alignment score = 0.677778
TaTCGTTCTCGCcgt
>Fam_517_15_2 Nr. of seq. 2 Alignment length(with gaps) = 15 Alignment score = 0.677778
tctTCGGATTCgGAG
>Fam_518_15_2 Nr. of seq. 2 Alignment length(with gaps) = 15 Alignment score = 0.611111
cTAATTTCTAGtagG
>Fam_519_15_2 Nr. of seq. 2 Alignment length(with gaps) = 15 Alignment score = 0.700000
CGTGGTCGTCGtgat
>Fam_520_15_2 Nr. of seq. 2 Alignment length(with gaps) = 15 Alignment score = 0.677778
aCGACGACGTTGagg
>Fam_521_15_2 Nr. of seq. 2 Alignment length(with gaps) = 15 Alignment score = 0.677778
GATAGAcATAGAgat
>Fam_522_15_2 Nr. of seq. 2 Alignment length(with gaps) = 15 Alignment score = 0.700000
AGCTaAACaAAGGAt
>Fam_523_15_2 Nr. of seq. 2 Alignment length(with gaps) = 15 Alignment score = 0.700000
aTTTAATAAcTGCTa
>Fam_524_15_2 Nr. of seq. 2 Alignment length(with gaps) = 15 Alignment score = 0.733333
TCTATgAAAGATgAA
>Fam_525_15_2 Nr. of seq. 2 Alignment length(with gaps) = 15 Alignment score = 0.666667
TaATATCGATTGagg

>Fam_526_15_2 Nr. of seq. 2 Alignment length(with gaps) = 15 Alignment score = 0.666667
ACAcaTTGTCATCag
>Fam_527_15_2 Nr. of seq. 2 Alignment length(with gaps) = 15 Alignment score = 0.655556
TgAAAaATGCTAaAt
>Fam_528_15_2 Nr. of seq. 2 Alignment length(with gaps) = 15 Alignment score = 0.744444
aAGTGTTTCTcAAAc
>Fam_529_15_2 Nr. of seq. 2 Alignment length(with gaps) = 15 Alignment score = 0.688889
TTATGGAcCcAtTTA
>Fam_530_15_2 Nr. of seq. 2 Alignment length(with gaps) = 15 Alignment score = 0.777778
TTATTTTCTTCaGtG
>Fam_531_15_2 Nr. of seq. 2 Alignment length(with gaps) = 15 Alignment score = 0.688889
TTgGGAATAtGTAAa
>Fam_532_15_2 Nr. of seq. 2 Alignment length(with gaps) = 15 Alignment score = 0.744444
aATgACATATTGAGg
>Fam_533_15_2 Nr. of seq. 2 Alignment length(with gaps) = 15 Alignment score = 0.611111
aACTTAcAaATCTGg
>Fam_534_15_2 Nr. of seq. 2 Alignment length(with gaps) = 15 Alignment score = 0.700000
tTCAAAgAGAACaTA
>Fam_535_15_2 Nr. of seq. 2 Alignment length(with gaps) = 15 Alignment score = 0.655556
AcATAGcAATTaTAa
>Fam_536_15_2 Nr. of seq. 2 Alignment length(with gaps) = 15 Alignment score = 0.666667
taTCTAATAAaTTCa
>Fam_537_15_2 Nr. of seq. 2 Alignment length(with gaps) = 15 Alignment score = 0.688889
cTACTAAGAAaAGtG
>Fam_538_15_2 Nr. of seq. 2 Alignment length(with gaps) = 15 Alignment score = 0.611111
tTTaCAGgGTAGtCA
>Fam_539_15_2 Nr. of seq. 2 Alignment length(with gaps) = 15 Alignment score = 0.744444
TCCCTTaAATcCAGt
>Fam_540_15_2 Nr. of seq. 2 Alignment length(with gaps) = 15 Alignment score = 0.744444
AAaTgAAAACTTGGa
>Fam_541_15_2 Nr. of seq. 2 Alignment length(with gaps) = 15 Alignment score = 0.688889
TGTcAAGcTgTGTAT
>Fam_542_15_2 Nr. of seq. 2 Alignment length(with gaps) = 15 Alignment score = 0.733333
TCAAGcCTTAAgTTA
>Fam_543_15_2 Nr. of seq. 2 Alignment length(with gaps) = 15 Alignment score = 0.655556
AACcAAGaAGAcCCa
>Fam_544_15_2 Nr. of seq. 2 Alignment length(with gaps) = 15 Alignment score = 0.644444
TTgATGCTGcTTaAC

>Fam_545_15_2 Nr. of seq. 2 Alignment length(with gaps) = 15 Alignment score = 0.655556
AcAAacAAGTTCAGa
>Fam_546_15_2 Nr. of seq. 2 Alignment length(with gaps) = 15 Alignment score = 0.655556
GAAaAcTAAGAATac
>Fam_547_15_2 Nr. of seq. 2 Alignment length(with gaps) = 15 Alignment score = 0.688889
TaAGAGTTTcATCAa
>Fam_548_15_2 Nr. of seq. 2 Alignment length(with gaps) = 15 Alignment score = 0.655556
aCAgcTTAAAGTCTg
>Fam_549_15_2 Nr. of seq. 2 Alignment length(with gaps) = 15 Alignment score = 0.655556
GAGAaTAGgTATCct
>Fam_550_15_2 Nr. of seq. 2 Alignment length(with gaps) = 15 Alignment score = 0.688889
TTTTTaAGtATCcCT
>Fam_551_15_2 Nr. of seq. 2 Alignment length(with gaps) = 15 Alignment score = 0.744444
ATaTTTTCTTcTTAc
>Fam_552_15_2 Nr. of seq. 2 Alignment length(with gaps) = 15 Alignment score = 0.733333
CGAaGACGACaCcGA
>Fam_553_15_2 Nr. of seq. 2 Alignment length(with gaps) = 15 Alignment score = 0.822222
CGCCGTTCTCcTCgT
>Fam_554_15_2 Nr. of seq. 2 Alignment length(with gaps) = 15 Alignment score = 0.733333
CaCCAGAaTCATCAg
>Fam_555_15_2 Nr. of seq. 2 Alignment length(with gaps) = 15 Alignment score = 0.733333
TTTAAGTaATcTAaT
>Fam_556_15_2 Nr. of seq. 2 Alignment length(with gaps) = 15 Alignment score = 0.644444
TTGaTTCTaTaCCTa
>Fam_557_15_2 Nr. of seq. 2 Alignment length(with gaps) = 15 Alignment score = 0.755556
AGAGAAAGAAaGacT
>Fam_558_15_2 Nr. of seq. 2 Alignment length(with gaps) = 15 Alignment score = 0.644444
cCTTACTTaCCaaAC
>Fam_559_15_2 Nr. of seq. 2 Alignment length(with gaps) = 15 Alignment score = 0.911111
GGAACCAGTGACAAc
>Fam_560_15_2 Nr. of seq. 2 Alignment length(with gaps) = 15 Alignment score = 0.733333
AGATTCATcCAaTTa
>Fam_561_15_2 Nr. of seq. 2 Alignment length(with gaps) = 15 Alignment score = 0.644444
TTaTGAAaCgATGTa
>Fam_562_15_2 Nr. of seq. 2 Alignment length(with gaps) = 15 Alignment score = 0.644444
aTAcTCTCaaCTAAA
>Fam_563_15_2 Nr. of seq. 2 Alignment length(with gaps) = 15 Alignment score = 0.733333
CaTTTTTTCATAacT

>Fam_564_15_2 Nr. of seq. 2 Alignment length(with gaps) = 15 Alignment score = 0.644444
aaAGGTcATTTCGgT
>Fam_565_15_2 Nr. of seq. 2 Alignment length(with gaps) = 15 Alignment score = 0.733333
TaaTTACgGATCAAA
>Fam_566_15_2 Nr. of seq. 2 Alignment length(with gaps) = 15 Alignment score = 0.644444
TCaATCCcTCTATga
>Fam_567_15_2 Nr. of seq. 2 Alignment length(with gaps) = 15 Alignment score = 0.644444
aTacTTTCATTTCCa
>Fam_568_15_2 Nr. of seq. 2 Alignment length(with gaps) = 15 Alignment score = 1.000000
AGCTACAATTTGCTA
>Fam_569_15_2 Nr. of seq. 2 Alignment length(with gaps) = 15 Alignment score = 0.733333
CTcTTCTaCTaCATC
>Fam_570_15_2 Nr. of seq. 2 Alignment length(with gaps) = 15 Alignment score = 0.733333
TTCATCAaCaTaGAT
>Fam_571_15_2 Nr. of seq. 2 Alignment length(with gaps) = 15 Alignment score = 0.644444
TaacTGAGAGAAaGT
>Fam_572_15_2 Nr. of seq. 2 Alignment length(with gaps) = 15 Alignment score = 0.733333
CAATTTCCGAacCTg
>Fam_573_15_2 Nr. of seq. 2 Alignment length(with gaps) = 15 Alignment score = 0.644444
CaTCTaCTCTTAaaG
>Fam_574_14_2 Nr. of seq. 2 Alignment length(with gaps) = 14 Alignment score = 0.750000
catAACTCTAAATG
>Fam_575_14_2 Nr. of seq. 2 Alignment length(with gaps) = 14 Alignment score = 0.654762
aAATGaAAGTGAac
>Fam_576_14_2 Nr. of seq. 2 Alignment length(with gaps) = 14 Alignment score = 0.773810
GAACgGGGAACGGg
>Fam_577_14_2 Nr. of seq. 2 Alignment length(with gaps) = 14 Alignment score = 0.678571
CTACcGaCCACCAa
>Fam_578_14_2 Nr. of seq. 2 Alignment length(with gaps) = 14 Alignment score = 0.678571
ATTGAcCATcCTTa
>Fam_579_14_2 Nr. of seq. 2 Alignment length(with gaps) = 14 Alignment score = 0.773810
TGAGtTTATTCAGa
>Fam_580_14_2 Nr. of seq. 2 Alignment length(with gaps) = 14 Alignment score = 0.642857
tgAGAcTCTAACTg
>Fam_581_14_2 Nr. of seq. 2 Alignment length(with gaps) = 14 Alignment score = 0.678571
ATaGTGGAATcAGg
>Fam_582_14_2 Nr. of seq. 2 Alignment length(with gaps) = 14 Alignment score = 0.678571
TTTGtCGCTTAcTt

>Fam_583_14_2 Nr. of seq. 2 Alignment length(with gaps) = 14 Alignment score = 0.726190
cgAaTTTATCTATC
>Fam_584_14_2 Nr. of seq. 2 Alignment length(with gaps) = 14 Alignment score = 0.761905
TCATTTaTCgCTTC
>Fam_585_14_2 Nr. of seq. 2 Alignment length(with gaps) = 14 Alignment score = 0.761905
TgAGGtTATTAGAG
>Fam_586_14_2 Nr. of seq. 2 Alignment length(with gaps) = 14 Alignment score = 0.678571
aAAACgTAAATGGc
>Fam_587_14_2 Nr. of seq. 2 Alignment length(with gaps) = 14 Alignment score = 0.726190
GTTCATaAaATTCa
>Fam_588_14_2 Nr. of seq. 2 Alignment length(with gaps) = 14 Alignment score = 0.666667
AaAcTCTGATAcTT
>Fam_589_14_2 Nr. of seq. 2 Alignment length(with gaps) = 14 Alignment score = 0.726190
aATAACCAGAATaa
>Fam_590_14_2 Nr. of seq. 2 Alignment length(with gaps) = 14 Alignment score = 0.726190
TCTcACTTTTGaGa
>Fam_591_14_2 Nr. of seq. 2 Alignment length(with gaps) = 14 Alignment score = 0.726190
TTTCAGAAaCaACc
>Fam_592_14_2 Nr. of seq. 2 Alignment length(with gaps) = 14 Alignment score = 0.666667
aTTTCgCAAGcAAT
>Fam_593_14_2 Nr. of seq. 2 Alignment length(with gaps) = 14 Alignment score = 0.666667
cTGAATaAATAgTC
>Fam_594_14_2 Nr. of seq. 2 Alignment length(with gaps) = 14 Alignment score = 0.726190
gCAGACaTTAAaAC
>Fam_595_14_2 Nr. of seq. 2 Alignment length(with gaps) = 14 Alignment score = 0.666667
AgAAACaTTAGgAG
>Fam_596_14_2 Nr. of seq. 2 Alignment length(with gaps) = 14 Alignment score = 0.726190
TGAAaaATAAAACt
>Fam_597_14_2 Nr. of seq. 2 Alignment length(with gaps) = 14 Alignment score = 0.726190
AaGAAATGTTcAGg
>Fam_598_14_2 Nr. of seq. 2 Alignment length(with gaps) = 14 Alignment score = 0.738095
TagAATAATAAcTT
>Fam_599_14_2 Nr. of seq. 2 Alignment length(with gaps) = 14 Alignment score = 0.714286
GGATTATTCgcaAC
>Fam_600_14_2 Nr. of seq. 2 Alignment length(with gaps) = 14 Alignment score = 0.714286
TcAcTAAACTgAAT
>Fam_601_14_2 Nr. of seq. 2 Alignment length(with gaps) = 14 Alignment score = 0.619048
TcAAcGAAaGCAAa

>Fam_602_14_2 Nr. of seq. 2 Alignment length(with gaps) = 14 Alignment score = 0.809524
GTAAaCTGTAAaCT
>Fam_603_14_2 Nr. of seq. 2 Alignment length(with gaps) = 14 Alignment score = 0.809524
AAGaaATCAATTGA
>Fam_604_14_2 Nr. of seq. 2 Alignment length(with gaps) = 14 Alignment score = 0.619048
aAaaAaTgaaTGTc
>Fam_605_14_2 Nr. of seq. 2 Alignment length(with gaps) = 14 Alignment score = 0.714286
TTTGaAGAAAcCAg
>Fam_606_14_2 Nr. of seq. 2 Alignment length(with gaps) = 14 Alignment score = 0.714286
GAAGGaATTTaAcA
>Fam_607_14_2 Nr. of seq. 2 Alignment length(with gaps) = 14 Alignment score = 0.809524
ATaAAaTACCAAGA
>Fam_608_14_2 Nr. of seq. 2 Alignment length(with gaps) = 14 Alignment score = 0.904762
CAACTCTGCTGGCa
>Fam_609_14_2 Nr. of seq. 2 Alignment length(with gaps) = 14 Alignment score = 0.619048
AAaAGgTTGaAaTG
>Fam_610_14_2 Nr. of seq. 2 Alignment length(with gaps) = 14 Alignment score = 0.714286
aAAAaTgGTTAGTT
>Fam_611_14_2 Nr. of seq. 2 Alignment length(with gaps) = 14 Alignment score = 0.714286
TGATgaACTCAaAC
>Fam_612_13_2 Nr. of seq. 2 Alignment length(with gaps) = 13 Alignment score = 0.717949
AAaAACAAAGGcg
>Fam_613_13_2 Nr. of seq. 2 Alignment length(with gaps) = 13 Alignment score = 0.653846
cTCGgCATCATcA
>Fam_614_13_2 Nr. of seq. 2 Alignment length(with gaps) = 13 Alignment score = 0.756410
tGTTCaTGATTCA
>Fam_615_13_2 Nr. of seq. 2 Alignment length(with gaps) = 13 Alignment score = 0.743590
AaAAGAAGtGAAT
>Fam_616_13_2 Nr. of seq. 2 Alignment length(with gaps) = 13 Alignment score = 0.705128
AaGACcCAAGTCt
>Fam_617_13_2 Nr. of seq. 2 Alignment length(with gaps) = 13 Alignment score = 0.705128
TCCCTaGTTaCTa
>Fam_618_13_2 Nr. of seq. 2 Alignment length(with gaps) = 13 Alignment score = 0.743590
CATTTCAtTTgCT
>Fam_619_13_2 Nr. of seq. 2 Alignment length(with gaps) = 13 Alignment score = 0.653846
aTATCTTcTaCAA
>Fam_620_13_2 Nr. of seq. 2 Alignment length(with gaps) = 13 Alignment score = 0.641026
CAtTCTaAcCATC

>Fam_621_13_2 Nr. of seq. 2 Alignment length(with gaps) = 13 Alignment score = 0.705128
aATATTTGTgTCg
>Fam_622_13_2 Nr. of seq. 2 Alignment length(with gaps) = 13 Alignment score = 0.705128
AGGTAaTTTgATc
>Fam_623_13_2 Nr. of seq. 2 Alignment length(with gaps) = 13 Alignment score = 0.705128
GTCAGGaAAaCTg
>Fam_624_13_2 Nr. of seq. 2 Alignment length(with gaps) = 13 Alignment score = 0.705128
CAaTGAAAAaATc
>Fam_625_13_2 Nr. of seq. 2 Alignment length(with gaps) = 13 Alignment score = 0.705128
AaATcTTATCAAg
>Fam_626_13_2 Nr. of seq. 2 Alignment length(with gaps) = 13 Alignment score = 0.743590
ATgCcTTTTTATC
>Fam_627_13_2 Nr. of seq. 2 Alignment length(with gaps) = 13 Alignment score = 0.705128
TTACTGTTcTGgg
>Fam_628_13_2 Nr. of seq. 2 Alignment length(with gaps) = 13 Alignment score = 0.705128
ATTaCCCTGTTgt
>Fam_629_13_2 Nr. of seq. 2 Alignment length(with gaps) = 13 Alignment score = 0.692308
GaTAAAAaAAcTG
>Fam_630_13_2 Nr. of seq. 2 Alignment length(with gaps) = 13 Alignment score = 0.692308
TGATCCAGAaCaa
>Fam_631_13_2 Nr. of seq. 2 Alignment length(with gaps) = 13 Alignment score = 0.794872
ATTcTGTaTTAAG
>Fam_632_13_2 Nr. of seq. 2 Alignment length(with gaps) = 13 Alignment score = 0.794872
CACTTTCaTTcCC
>Fam_633_13_2 Nr. of seq. 2 Alignment length(with gaps) = 13 Alignment score = 0.692308
aAAcATTAaATCG
>Fam_634_13_2 Nr. of seq. 2 Alignment length(with gaps) = 13 Alignment score = 0.794872
GaAAGAAAGAcAT
>Fam_635_13_2 Nr. of seq. 2 Alignment length(with gaps) = 13 Alignment score = 0.692308
agACAGGcAAAAA
>Fam_636_13_2 Nr. of seq. 2 Alignment length(with gaps) = 13 Alignment score = 0.692308
aCAAgTAaTAAAG
>Fam_637_13_2 Nr. of seq. 2 Alignment length(with gaps) = 13 Alignment score = 0.692308
TTgTTTATgGAaG
>Fam_638_13_2 Nr. of seq. 2 Alignment length(with gaps) = 13 Alignment score = 0.692308
CAaAgcTTAATTA
>Fam_639_12_2 Nr. of seq. 2 Alignment length(with gaps) = 12 Alignment score = 0.694444
TATCaTAAAAtg

>Fam_640_12_2 Nr. of seq. 2 Alignment length(with gaps) = 12 Alignment score = 0.666667
TTTTTGGaCtAC
>Fam_641_12_2 Nr. of seq. 2 Alignment length(with gaps) = 12 Alignment score = 0.680556
aCCTGATAAcTc
>Fam_642_12_2 Nr. of seq. 2 Alignment length(with gaps) = 12 Alignment score = 0.680556
TTaAATaTTCGg
>Fam_643_12_2 Nr. of seq. 2 Alignment length(with gaps) = 12 Alignment score = 0.680556
AGGAgAaACTGg
>Fam_644_12_2 Nr. of seq. 2 Alignment length(with gaps) = 12 Alignment score = 0.680556
CAgCAAGAgTTc
>Fam_645_12_2 Nr. of seq. 2 Alignment length(with gaps) = 12 Alignment score = 0.722222
TcCTACCaGTTC
>Fam_646_12_2 Nr. of seq. 2 Alignment length(with gaps) = 12 Alignment score = 0.722222
ACTTTTCtCcAT
>Fam_647_12_2 Nr. of seq. 2 Alignment length(with gaps) = 12 Alignment score = 0.611111
aCCGcGCAaGCT
>Fam_648_12_2 Nr. of seq. 2 Alignment length(with gaps) = 12 Alignment score = 1.000000
CAGGCGTGTCCT
>Fam_649_12_2 Nr. of seq. 2 Alignment length(with gaps) = 12 Alignment score = 0.666667
GAGcGCGAcGaC
>Fam_650_12_2 Nr. of seq. 2 Alignment length(with gaps) = 12 Alignment score = 0.666667
CTCaaACTCAaA
>Fam_651_12_2 Nr. of seq. 2 Alignment length(with gaps) = 12 Alignment score = 0.666667
TcTcTCTGgTTT
>Fam_652_12_2 Nr. of seq. 2 Alignment length(with gaps) = 12 Alignment score = 0.666667
CCAGaaCCATCa
>Fam_653_12_2 Nr. of seq. 2 Alignment length(with gaps) = 12 Alignment score = 0.777778
CCAACgCCaACA
>Fam_654_12_2 Nr. of seq. 2 Alignment length(with gaps) = 12 Alignment score = 0.666667
acAACCaCAACC
>Fam_655_12_2 Nr. of seq. 2 Alignment length(with gaps) = 12 Alignment score = 0.777778
ATCcTGAaAACA
>Fam_656_12_2 Nr. of seq. 2 Alignment length(with gaps) = 12 Alignment score = 0.777778
ATAaTTTGTaTT
>Fam_657_12_2 Nr. of seq. 2 Alignment length(with gaps) = 12 Alignment score = 0.777778
ATcAAAaAGGAG
>Fam_658_12_2 Nr. of seq. 2 Alignment length(with gaps) = 12 Alignment score = 0.888889
TGTGAcGTTATT

>Fam_659_12_2 Nr. of seq. 2 Alignment length(with gaps) = 12 Alignment score = 0.777778
CAGGTCAAaTCa
>Fam_660_12_2 Nr. of seq. 2 Alignment length(with gaps) = 12 Alignment score = 0.666667
TgcCTCgGTTGG
>Fam_661_12_2 Nr. of seq. 2 Alignment length(with gaps) = 12 Alignment score = 0.777778
CccTTTAAAAGT
>Fam_662_12_2 Nr. of seq. 2 Alignment length(with gaps) = 12 Alignment score = 0.666667
ATcAAAgGAACc
>Fam_663_12_2 Nr. of seq. 2 Alignment length(with gaps) = 12 Alignment score = 0.805556
GAtATTAaTACT
>Fam_664_11_2 Nr. of seq. 2 Alignment length(with gaps) = 11 Alignment score = 0.696970
gTAAGCAgAAA
>Fam_665_11_2 Nr. of seq. 2 Alignment length(with gaps) = 11 Alignment score = 0.651515
ACAaTaTAAGg
>Fam_666_11_2 Nr. of seq. 2 Alignment length(with gaps) = 11 Alignment score = 0.772727
GGAAcAATCAa
>Fam_667_11_2 Nr. of seq. 2 Alignment length(with gaps) = 11 Alignment score = 0.772727
aAAGTAAAGGc
>Fam_668_11_2 Nr. of seq. 2 Alignment length(with gaps) = 11 Alignment score = 0.636364
aCcTCGcTGCC
>Fam_669_11_2 Nr. of seq. 2 Alignment length(with gaps) = 11 Alignment score = 0.636364
ATCgGGAcgGG
>Fam_670_11_2 Nr. of seq. 2 Alignment length(with gaps) = 11 Alignment score = 0.757576
AaTTATCTAaG
>Fam_671_11_2 Nr. of seq. 2 Alignment length(with gaps) = 11 Alignment score = 0.757576
AgAAGTcACTA
>Fam_672_11_2 Nr. of seq. 2 Alignment length(with gaps) = 11 Alignment score = 0.878788
gAATTGTTGAA
>Fam_673_11_2 Nr. of seq. 2 Alignment length(with gaps) = 11 Alignment score = 0.757576
TCAAaTATATa
>Fam_674_271_1 Nr. of seq. 1 Alignment length(with gaps) = 271 Alignment score = 0.000000

GGAACCATCGACGCAAAAGGCATCTTCACGGCACTCACCGCAGAAGGATCAACCACAGTCACAGCCACTGCNGGGGACATCTCCGGGACAGGCTGAGGCGACNGTGAACCC
GGCTCCTCCAGTCATAACCAGCATTGCGGTATAGCCCTGCCAGCCCCAACCATTGATATAGGAGACACGCAAGCAGTTTACCGCCACCTGCTACGATCAGTATGACAATGA
GATGCCCGANGTGACNNNTGTTTCCTGGTCAAGTGAGAATACAACAGTA
>Fam_675_264_1 Nr. of seq. 1 Alignment length(with gaps) = 264 Alignment score = 0.000000

TGGGACTTTGATAACGATGGAACTGTAGACAGTACTAAGCAGAATCCAGTCTACACTTATGCTGCATCTGGTAACTACACTGTCAATCTTACTGTTTCCAATGCAGGTGGA

AGTGATTCTGAAGTCAAGACTGATTACATTGTCGTAAGTGAACCACTACCTGGAGTACCAGTTGCAAACTTTACTGCAACGCCTACTTCTGGCGATGCACCACTTAGTGTG
AACTTCACTGATGCTTCAACCGGCACTGTCTCCTCATATGCA
>Fam_676_258_1 Nr. of seq. 1 Alignment length(with gaps) = 258 Alignment score = 0.000000

ATGTATTATCGTGACGGAACAAACTACACTATCACATTGAAAAACACGAAAGGAACACCACTAACTAATGCCGATGTAACATTCACAATCAATAATGAAAACTACACAAGA
ACAACTAATAAAAATGGTATCGCATCCATTACAATCAACCTGCCGGAAGGAACATACACAATAAAAGCAACATATAAAAACGCATCAACAACCAATAAAATTATTGTTAAA
GACTACGAACCAGTAATCACTGGAAAAGATGTGGAA
>Fam_677_258_1 Nr. of seq. 1 Alignment length(with gaps) = 258 Alignment score = 0.000000

TGCAGATTTCGTTGGTTCTCCAGTCTCAGGAAGTTCCCCATTAAAAGTGCAGTTTACTGACAAGAGTACAGGGTCCCCGACTTATTGGAAATGGAACTTCGGAGATGGATC
GGATCTGGTAACTGAATATAATCCAACACATACATACTCAAAACCCGGAACGTATACTGTGAAAGAGACCGTGAGTAATGCAGCAGGTAAGGATACGGAAATAAAAACGGA
TTATATAACCGTGACAGAAGCTTCACAAACTCCTGA
>Fam_678_255_1 Nr. of seq. 1 Alignment length(with gaps) = 255 Alignment score = 0.000000

TATGCCAGGGNGCNGCNGCAACATTCNCTGTTCAAGCANANAGCAGTGAGCCATTGAGCTATCAGTGGCTCAAAGATGGAATGAAGATNCCNGGTGCTACGTCTGATACGT
ACACCATCCCTGCCGCTAACCTNANCGATACGGGAAGCTACTCNGTNCAGGTGACAAATAANTGCAGCCAGATCGAATCGGAGGCTGCTGNTCTGGATATCATCGCNATGC
CTGANATCCTGNCCCAACCGACCAGCCAGAAGG
>Fam_679_254_1 Nr. of seq. 1 Alignment length(with gaps) = 254 Alignment score = 0.000000

GATGAAAACGGTACTGCNAGATTAAACATTTGGTTACATCCNGGCAGCTATATTATAACTGCTTANAATCCAANTAATAATGAAGCAANNNCAAACAATGTAACTGTTTTA
TCNACAATTAATGCAAAAGATGTTACANTATANTACAAAATGGTACTCANTATTATGCAGTTGTTTTAGACGGTCAAGGTAATGCTTTAGCTAATCAGACTGTTAAATTNA
ATATTAATGGTGTATTCTATACTAAAACTACT
>Fam_680_254_1 Nr. of seq. 1 Alignment length(with gaps) = 254 Alignment score = 0.000000

AATTCTTCCTGTAGCGAATTTCACTGCAAATCCGACAGAGGGTTTTGCTCCTCTCACTGTCCAGTTTAACGACAGTTCTAAAAATGCAACTTCTGTAAGCTGGGACTTTGA
CAATAATGGAGTTAGTGACTCCACAGAACGAATGATCCAATTTATGAGTTCACAGTTGCAGGACTCTATACCGTTAATCTGACTGCAATCAATGTAAACGGTACGAATTCA
ACGTCAGCTATAATCAACGTTACTGAGAAACC
>Fam_681_252_1 Nr. of seq. 1 Alignment length(with gaps) = 252 Alignment score = 0.000000

CCATGCTGTCGGGTTGTTTTCACTTTTGTCCTTGAATTGAACGGTAAGAGGAACCGGTCCTGTAGTTGTATTTGCTTCAAAATCTGCTGTNGGTTCAACTATTGNATATGC
CCGGATGTAAGAAAGAGCTTCCGAGGTATCGGANCCGTACNCATTTTTTACTGTCAGCTCCACGGTATANTTTCCTGCTGAGGAATATGTATGGGAAGGATTTTGTTTTGT
TGAAGTAGTTCCGTCTCCGAAGTTCCAGTT
>Fam_682_249_1 Nr. of seq. 1 Alignment length(with gaps) = 249 Alignment score = 0.000000

TTCTGGTTTCTCTTTCTTGTCCTTATCTGGCAGTAAAACCTGAGCACCAGGCTTGGTACCGGTTGTACCGTCATAATTCGGAGTGGCAAGAGTTGTACCTTCATCAGTGAT
CTCCTGAGAACCGGAAGGAGTAGTAACCTGGGCACCTTTCTTCTCCTTATCTTCCTCAGTCTCCTGAACAGGGATTATAGTCGACGTGGCCTCAGGACTAACAGTTACAAC
TGGCTCGACATCCGAAGTCTTTTCAGA
>Fam_683_211_1 Nr. of seq. 1 Alignment length(with gaps) = 211 Alignment score = 0.000000

GACCTCCAGAACGGTTTCATCGCAGGAGGGGCGGAGGCCGCTGGCGTGGGAGTCCTGGCTGAAGGCNGNAGAAACNGGATCGCNAGCGAGTACCCTGGCGGCCGGAACCGG
ATGGTATGGAACCGGCGCCTACGGCGGAGGCATNGAGGCNTCCAACAGGGAGGGTGCNGTTGNNGCNGCCGCNGCCGCCGGNGGCCTGGGCGCAANCNTC
>Fam_684_195_1 Nr. of seq. 1 Alignment length(with gaps) = 195 Alignment score = 0.000000

ATGTTATCTGAAACATTTATCGTCTTGTCACCGCTTGCATTGTAATTACCTGCATCGTCATAGAAGGTAGCGTTGAAGACAATGTCCTCCACTGAATCCATTGGGACATTC
AGGGTATAGTTGTACCAGTTATCGTTCTTTGTGAGTTCAGCAGTATCGTTAGTTGAAATGTTGAACTCTGTACTAGTAATTCCA
>Fam_685_192_1 Nr. of seq. 1 Alignment length(with gaps) = 192 Alignment score = 0.000000

TTGATCTTGGCGACAAAAGCGTCAAACGAACCGGCATTCGATGCCTGCAATGGATTTTTTGTCGGGAAGTTGGCGGAGNCTGTATACCCGGTGACATAGGCGTTGCCGCTC
CCATCTACAGCGATGCCTTCGCCATAGTCATAGCCGCTTCCTCCCAGGTAGGTGGAATAAGACAGTGCCGACCCGGCGGAA
>Fam_686_176_1 Nr. of seq. 1 Alignment length(with gaps) = 176 Alignment score = 0.000000

CTGTCGCCATCATCAGGGGTGAGTTTGGCCTGCTGACTCCAGGATCCGTCNTCGGCCTCGAACACGTACGCCGAGCCCGCCTCCTCGCCGTTGGAGGTCTTCGTCGACTGT
GGGCNCCGATAATNGCAGTGGTCCCGTCACTCGACATTGCCACTGACNNGCCAAACCAGTCTTCA
>Fam_687_159_1 Nr. of seq. 1 Alignment length(with gaps) = 159 Alignment score = 0.000000

GTGTTCTCATAACCATATGCNNNACCACTNGAANCNCCTATTAGTAGGTCATAGTCTCCATCGCCGTCNAGGTCAGCTAAACATGGTGCTGCNNTACTTCCTATGTCAGGA
GCATCCCATTCAATTTTTCTTGTCCACACTGGACTGCTTGGAGTCCCT
>Fam_688_156_1 Nr. of seq. 1 Alignment length(with gaps) = 156 Alignment score = 0.000000

ATAGTAGACACGCGGATCTCCAACGTAAGTTATTGATGAAAGCNGACTTANAGNCAATGCTGGCTGTCCACCAGAATCTACAGTCACATCTCTGTTATGCCAGGTACTGTC
AGAGCTATGCCATGCATATTCGTAGACATGATAATTATTGCCAAN
>Fam_689_156_1 Nr. of seq. 1 Alignment length(with gaps) = 156 Alignment score = 0.000000

GATGCCTGGCTAATAAAGACNGANTCNAATGGCAANGAGCNNTGGAACAGAACCTTTGGAGGGNCNAANGACGATTGTGGCTANTCGGTACANGAGATNAANGATGGCGGC
TATATCATTGCNGGTGNGACGAGNTCCTATGGNGCAGGNGGAANN
>Fam_690_154_1 Nr. of seq. 1 Alignment length(with gaps) = 154 Alignment score = 0.000000

CCGTACGTCTCGTCGAGGTACTCGACGATGTCAGACCCACCTTTTCCTCCTCGGGTTCGGTCACTCCGTTCCCGAACCACTCGTCGCAAAACGTGGGCGAAAAGACCGCCC
ACTCGCTTCGCTCGTGGACGAAGAGAACCCTTACGCTACGGTT
>Fam_691_154_1 Nr. of seq. 1 Alignment length(with gaps) = 154 Alignment score = 0.000000

TCCAGACCTCCCGNCCCTTCGAGTCGGCCTTGAGGAGCCAGAGGTCCTTNCCNCCGGCGCCGAAGGANTCGGTNCTGCCCGACGATGACGAAGCCNTCGGCCGTCTCCTGG
ANCGAGNAGCCGNCATCGTCTCCGGGCCCCCCGAAGGTCCTGT
>Fam_692_154_1 Nr. of seq. 1 Alignment length(with gaps) = 154 Alignment score = 0.000000

GTGAAGTGGCAAAAAACCTACGGAGGAGNATGGNNTGATNANGCCTNCGCGATTGCTCTGNTACTAACGAGNGANATCATTATNGCCGGANNAACNNATAGTTTCGGTGCN
AGAAGGAGATGTNTGGGTTCTCAGGCTCGACGAGTATGGAAAT
>Fam_693_153_1 Nr. of seq. 1 Alignment length(with gaps) = 153 Alignment score = 0.000000

TATGATGTAGCCNCCGTCGNCTGTCTGCTGGACCGAATNTCCCTCCTCTTGNCCNGCTCCTCCGAAGGTTCTGTCCCATACCCTGTATCCGGANGAGTCCGTCTTGATCAG
CCAGAGGTCTTTNCCGCCNAAGTCGTCGAGGGTATAGCCTGT
>Fam_694_150_1 Nr. of seq. 1 Alignment length(with gaps) = 150 Alignment score = 0.000000

TCGTAGAATAGGAGGTCGGTGAATCCGTCNCCGTTGAANTCTCCGGGGACGATCGTCGACCATGTCCTCCTCCATCCGCTGTGAGATTTCAGGAGCCTGATGTTCCCGCTC
CCGTCGGTGGCGTAGAACTCGCCCTGGCCTGCGGCGGGG
>Fam_695_149_1 Nr. of seq. 1 Alignment length(with gaps) = 149 Alignment score = 0.000000

TCGACCGCCACACATCGTTCTGANAGGTGTTNCCGTCTAACCGCCCATGAGTACGATGCTNCCGTCCNGCATCGCGACACNGCTCTGTTCGGNTCGTCCCGACCACCCGGA
ACTCGGGTTCTGCTCTGTCCACGTTNTGCCGTTATCGG
>Fam_696_147_1 Nr. of seq. 1 Alignment length(with gaps) = 147 Alignment score = 0.000000

CACAGCAGTGTTGTTCTTCCCGACGGCAGTATCGTGCTCATGGGCGGTNATGNTAGCAACGTTAAGAATGACACGTGGCGGTCAACTGATAACGGTGCAACCTGGACCGAG
GTCAACTCAAGCGCCGGCTGGTCTGCAAGGTATTCT
>Fam_697_145_1 Nr. of seq. 1 Alignment length(with gaps) = 145 Alignment score = 0.000000

TGGAGGGAACTGATATGATCTTCGAGATGTTCTATAAAAATGTCAAAATCGCCTACGAATCAACTGTATTCAGCATGTTTTACTGCTTCTAATTAAGCTGTAAGACAGGTG
CATAACGGGAAACGACCAAAATCCAGGAAAATAT
>Fam_698_142_1 Nr. of seq. 1 Alignment length(with gaps) = 142 Alignment score = 0.000000

CAAAAAGCTCTAGAAATATATACAGTTAGATAAATATCCACTTGACTATGCCATGGTTCAAAATAATCTTGGAAATGCATATAGATGTTTAGCTGAGGTTAGAGATAAGGA
AAAAAATGCTCAAAATGCTATTAATGCATAC
>Fam_699_141_1 Nr. of seq. 1 Alignment length(with gaps) = 141 Alignment score = 0.000000

CCCAGTGGGGATCAGCAGGATCAGGAAACGGGCAGTTTAACCAGCCCTAGGGGATCGCCATCAACACGACCGGGTATGTCTACGTGACAGACTAGGACAACAACCGGATCC
AGGTATTTGATCCGAGCGGAAACTATGTTA
>Fam_700_141_1 Nr. of seq. 1 Alignment length(with gaps) = 141 Alignment score = 0.000000

TTCCAGGCAATGTATAGACTATTGTTATGTACACAAAGAGCCGGGGATTCAGGACTTGTCTCAGAAGAAGTGTATTTGTTCCCAAAGGTCATTCCGTTGTCCGCCGAGTAC
ATCACATTCAAATTGTTGTTGCCGTCGCCT
>Fam_701_141_1 Nr. of seq. 1 Alignment length(with gaps) = 141 Alignment score = 0.000000

AAGGTTACAGCCACGGTAAAAGTAGGAAATCGTCCTTATGGAATTGCAGTAAGTCCTAATGGAAAAAAGGTATATGTTACAAACTCAGGTACCTATAATGATCCGGGAAAT
ACTGTCTCTGTAATTGACACAGCAACAAAC
>Fam_702_141_1 Nr. of seq. 1 Alignment length(with gaps) = 141 Alignment score = 0.000000

TAACTGATCCGGGGGTTGCCGGCGCTGTCCAGCGCCAGGGANGTGTACCACCCGACANACCCCTCTGAATCCACCGTTTCATTGTGCCAGCCCGAGTCATCGTGCCACGCA
TACTTCAGGTCGTTATTCGTNTNATCGAAG
>Fam_703_138_1 Nr. of seq. 1 Alignment length(with gaps) = 138 Alignment score = 0.000000

GCATCGTTGTTGTAATCCAGATACCAACCCCGATTAGAAGGCCTGAAAACACCTATTGTGTCCTTACCATCACCATTCCAATCGCCTGCAACTGCCAGGTCACCATTCAAG
CCGTACACAATGATAAAGTCAGGAACA
>Fam_704_137_1 Nr. of seq. 1 Alignment length(with gaps) = 137 Alignment score = 0.000000

TTTTCCAATTTCTGATGGTAACTGTNTTAGATTATTATGACAAAGNAAAGTTTTATAAGNTTTNTAAGTTCCCCAATTTCTGGAGGTAGTAGAGTCAATTGATTNCTAGAT
AAGTCAAGTTCTCTAAGGTTCTTAAG
>Fam_705_133_1 Nr. of seq. 1 Alignment length(with gaps) = 133 Alignment score = 0.000000

CGGTGTCCTTTTCGCTCGCTTTGCTCTCTCAAGAGGACTAATTGACATATTTATGAATCAGCCGTGAATCAAACGTGGAAGGCATCGAGTACAAACCCACATGCTCTCCAT
CCCGAGTTCCTTCTCGGGAGGA
>Fam_706_129_1 Nr. of seq. 1 Alignment length(with gaps) = 129 Alignment score = 0.000000

TGCTGATCCACTGGTTGTAATTTGAGTTTCTACGGAGGTGGACAAATTGTACATGTAGANCTCAGATTGATTCCATCCGTTGCGATTATCCTTCCACACGATTTTATCCCC
GTAAATTGCAGGATAATC
>Fam_707_126_1 Nr. of seq. 1 Alignment length(with gaps) = 126 Alignment score = 0.000000

AATCTTGCAGGACTTTACNCNAAAATAGGAAGATATGAAGAAGCTNTACNTCTTTATAATCGTGCTTTAGAGATCNATAAAAAATTNTTTGGAGAGGAACATCCCTATATT
GCNATNACTCTAAAC
>Fam_708_126_1 Nr. of seq. 1 Alignment length(with gaps) = 126 Alignment score = 0.000000

CCAGGGCAAATATTCTGATGCCGAACCACTTTACACTCGTGCCCTGGAAATCCACGAGAAGGTTTTGGGGTCAGAACACCCTTACGTAGCAACTTCTCTTAATAATTTAGC
AGGACTCTACGTACA
>Fam_709_126_1 Nr. of seq. 1 Alignment length(with gaps) = 126 Alignment score = 0.000000

AATAATCTAGCGGAACTATATAGAAATTCAGGCAGATATTCTGAAGCTGAACCTCTGTATACCCGTGCCTTGGAAATTACAGAAAGAGCACTGGGACCGGAACATCCAGAT
GTAGGCACTCGTCTT
>Fam_710_126_1 Nr. of seq. 1 Alignment length(with gaps) = 126 Alignment score = 0.000000

CCTAGCGATTTGCGCCAAATTTCTGTTGCCTGGCGGAAAAGCGGCTCAGCATCGCTGTATCTGCCCATCGAATCGTACAGCNATGCGAGGTTGTTCAGGCTNGNGGCGTAG
TCGGGATGATCTTCG
>Fam_711_126_1 Nr. of seq. 1 Alignment length(with gaps) = 126 Alignment score = 0.000000

ACGTATATGTATCCATTACTAGGATCATANAANATACTCNATGGATCTTGTCTNATNGATATATTAGCTATNACANTGTTNGTAGTAGAATTAATTACAGAAACTGTATTA
GACCTAGAATCTGCA
>Fam_712_122_1 Nr. of seq. 1 Alignment length(with gaps) = 122 Alignment score = 0.000000

AATTGCTTTTTTAGATTCNCCCTTTGTAAATGAATGCTAGACCTATATTNCCGATNCGAATTCCTTCACTTNACCATATTGTTTATNTCCTTTGAAATTTTCAAAGCTTGT
TCATAATATTC
>Fam_713_121_1 Nr. of seq. 1 Alignment length(with gaps) = 121 Alignment score = 0.000000

CCTTCCAATGGTTACGTGTATGTAACGAACTTACATTCAAATNACAGTCTCTGTTATCTCAGGTACTAATGTTGTAGCTAACGTCCCTGTTGGTGTTGGCCCGANCTATGC
TCTTTACGAC
>Fam_714_120_1 Nr. of seq. 1 Alignment length(with gaps) = 120 Alignment score = 0.000000

TATGATCCTGAGAATGGGTATATGTATGTTACAAACTTTGGTTCTAATACAGTTTCTGTAATCTCTAATACATCAGTAATTGAAAATATTAATGTTGGGAATGGACCTATA
GGTATTGCT
>Fam_715_120_1 Nr. of seq. 1 Alignment length(with gaps) = 120 Alignment score = 0.000000

TAAATCCTGACGGAACAGAAAAATGGAGATTTAATACGGGAAAGTCGATTGATTCAACTCCNGCNATTGNAAAAGATGGTACAATCTATTTTGGTTCANATGATGGTAATT
TATATGCCA
>Fam_716_120_1 Nr. of seq. 1 Alignment length(with gaps) = 120 Alignment score = 0.000000

TGTATGCGATCGATGCCGNNACNGGAAAAGAGAAGTGGCGGTTCGCTACGGNAGATNGNGTGGTTTCATCTCCNGCNGTAGCGAACGGNATTGTCTATGTCGGGAGTGAGG
ATAANAACC
>Fam_717_120_1 Nr. of seq. 1 Alignment length(with gaps) = 120 Alignment score = 0.000000

AATATTATGAGCAGGCTCTTTTAATTTCTCGAGAAATTGGTGATCGACGCGGAGAAGGAANNCATCTTGGCAACCTTGGTAGTTCCTACNTCCATCTTGGTGAAGTTACTA
AAGCTATCG
>Fam_718_120_1 Nr. of seq. 1 Alignment length(with gaps) = 120 Alignment score = 0.000000

CTCGATGATTCNAAATTTCAGTCAATTATCCTGAACAGTTTGGGAAGCATTCTTCAGAAACAGGGTAGATTGAGGGAAGCAGAAACTACTTTTCAAGAGAGCTTGCATATA
GCGAAAAAA
>Fam_719_120_1 Nr. of seq. 1 Alignment length(with gaps) = 120 Alignment score = 0.000000

```
TCCATTTGAGGGTGCCGTTGGCGTTGAGCGCATAGAGACTGTTATCNTAACTCCCNGCGTACACGGTCCCGTCCGAAGCGATCGCAGGGGAGCTATAAATGCGTCCACCGG
TAGCGTACG
>Fam_720_120_1  Nr. of seq. 1 Alignment length(with gaps) = 120 Alignment score = 0.000000

CACCGTCTTCGTCGGGAGCAACGACGCCAATCTGTACGCCGTAGACGCCGAAACGGGTACCGAACAGTGGCGTTTCGAAACCGACGACCGGGTCCANTCGTCGCCGACAGT
GGCCGACGG
>Fam_721_117_1  Nr. of seq. 1 Alignment length(with gaps) = 117 Alignment score = 0.000000

CTAAATCCTATGTTTCTAATGGTAAGGTTGCTTTCAAAGTTAATGATAAAACTGTTGGTTATGGTAGTGTTTCTAATGGTAAGGCTTATTATACTTATGATTCTAGTAAGT
TATCTG
>Fam_722_117_1  Nr. of seq. 1 Alignment length(with gaps) = 117 Alignment score = 0.000000

AATTCCCAAATCTCCCTACCAGTTTTTAAATCCAAGGCGTAGGTGTATCCTNCANNACATCCCAATACAACAATATCGTCTTTTATGGATATTNCCNATACACTCTCAGCA
GCCTTA
>Fam_723_113_1  Nr. of seq. 1 Alignment length(with gaps) = 113 Alignment score = 0.000000

GCGTGACAAAAACGACACGGTAAAGCGAATACGGTTGTACTGGTCCTACATATTATTCAGTAGTCCTCTTGAGCGCAGCGAAAAGGACCTCGTGCTCCCGAAGCGAAACTC
GG
>Fam_724_109_1  Nr. of seq. 1 Alignment length(with gaps) = 109 Alignment score = 0.000000
 TTATCTGGCACGTCAGAAAATACAGCCATAAGAACTTTAGCGGCCCACGTTCTCACTTCATCGNTCTTGATCAGTGAGAAGTCTAATTAAGTCATTGTATGCCTGTTGC
>Fam_725_108_1  Nr. of seq. 1 Alignment length(with gaps) = 108 Alignment score = 0.000000
 GCTTTATCGTATTCTTCTTTTAANNGATAACAGAAACCCANATTGTTATTTATCTGCCATTTTTCTTTATCGGGGCTGATTTCCAGTGCTTTCTTATAATATTTAATG
>Fam_726_108_1  Nr. of seq. 1 Alignment length(with gaps) = 108 Alignment score = 0.000000
 TTTTCAATATGTACCGGANAAAAATCAGGCATGGGAAGANTTAGTTAGATTGACNTCAGATAAAGANAGTGATNTACGTTGGANAGTAGCAGAGGCNNTTGGAANTGT
>Fam_727_105_1  Nr. of seq. 1 Alignment length(with gaps) = 105 Alignment score = 0.000000
 AAGCAGAAGAATTTTACAGAAAAGCTCTGGAAATTGANCCAGANNACGCTAATAACCTTGGAAATTATGCAAATTTCCTNNANNATATTCGNAAAGATTACAATA
>Fam_728_103_1  Nr. of seq. 1 Alignment length(with gaps) = 103 Alignment score = 0.000000
 CAAGCTTATAATGTTTTTCAGCTTCTTCCATACGTCCCATTCCGCTNCAGGAAAAGTCCGTAATTTAANTGTATGGATACAATTTTTGGATCGATTTTCAAAG
>Fam_729_102_1  Nr. of seq. 1 Alignment length(with gaps) = 102 Alignment score = 0.000000
 CTTTTTCATATCTCTCTAATTTATATAACGAATCTGCTTTATAATACCAAGCGGCTGGATTATTTGGATCGAGTTTTATTGCTTTATCAAAACATTCTATCG
>Fam_730_102_1  Nr. of seq. 1 Alignment length(with gaps) = 102 Alignment score = 0.000000
 TCTATCGCTTCATTGTACCTTTCAAGATTACAAAGTGCATCGCCCTTGTTATTNCAATATGAACTAAAATCTGGATCTATCTCAATTGCTTTATCATAGCAC
>Fam_731_102_1  Nr. of seq. 1 Alignment length(with gaps) = 102 Alignment score = 0.000000
 ATTGCTTCTTCGTATCTTCCCAAGCTACTAAGTGCCAAACCCTTGCTATCCCATGNACTATCAGACNTTGGGTTTATTTCTATGGCTTTATCATATGCCTGT
>Fam_732_102_1  Nr. of seq. 1 Alignment length(with gaps) = 102 Alignment score = 0.000000
 GACCGCCTCCGAGAGCCGCCCCAGGTCCCNGAGGGCGACCCCCTTGTTGTTCCAGGCGTGGGGGTANNCGGGGTCGAGGCTCAANGCCATATCGTANGCCTC
>Fam_733_102_1  Nr. of seq. 1 Alignment length(with gaps) = 102 Alignment score = 0.000000
```

```
TCAGGATTCAGCCTCATAACCTCATCAAAAGCCTGGATGGCCTCGTCGTACCTGCCCAGTGAATCGAGAGCNGCACCTTTGCTGTTCCAAGCATCGACATAC
>Fam_734_102_1  Nr. of seq. 1 Alignment length(with gaps) = 102 Alignment score = 0.000000
AAAGCGCAAACTCATCGTCAGGATTTAGTTCCAAAACCTTATTAAAAATCTCAACAGCTTCCTCATATCTATCTAATAAAACTAAAGAAGATCCTTTGTTAG
>Fam_735_102_1  Nr. of seq. 1 Alignment length(with gaps) = 102 Alignment score = 0.000000
TCAATTGCTTTATCAAAGGCTTTTACTGCTTCCTCGTAATTGCCTAAACACTCAAGAGAATATCCTTTATTGTACCATGCATCTGAATTTTGAGAGTTAATT
>Fam_736_102_1  Nr. of seq. 1 Alignment length(with gaps) = 102 Alignment score = 0.000000
ATGGCCTCATCGTATTTGCCTAGGGCCNNGAGCGCANTGCCTTTGTTGGACCAGGCNTAGGCATACTGNGGATCNATGCTGATAGCCTGATCGNAAGCCTTG
>Fam_737_99_1  Nr. of seq. 1 Alignment length(with gaps) = 99 Alignment score = 0.000000
GTTTAGTTGGTTATAATCACGGTTATTATGGTGGTNATTTGACAATCACAAACAGCTATGCTACAGGAAATGTAACAAGTTATGGCGACCGTGCAGGTG
>Fam_738_99_1  Nr. of seq. 1 Alignment length(with gaps) = 99 Alignment score = 0.000000
TTAATATTCAGGATGAAAACGGCGANACNGCTTTGATTTCTGCGGCAAANGGNGGAAANAGGAACATTGTTGAGTTGCTTATTAAAAGTGGTGCCGANC
>Fam_739_98_1  Nr. of seq. 1 Alignment length(with gaps) = 98 Alignment score = 0.000000
AAAAATAGCGAGGAGAAGTTCCAAACGAAAGGGCTACGCCCCTTTCAACCCTAGCGGGATAACAAGTTATCCCACTGGTAAAGTTTTAAGTTTTACAA
>Fam_740_87_1  Nr. of seq. 1 Alignment length(with gaps) = 87 Alignment score = 0.000000
GACAACCACAATCCGGATCAGAACAAGAATCCTCATCCTCATGCCCATGATGATGCTCATGCTCGTGCCCATGGCCATGCTCATCAT
>Fam_741_86_1  Nr. of seq. 1 Alignment length(with gaps) = 86 Alignment score = 0.000000
AGATGATTCCCGGAACCGCCGTTACTCGAAAACGCTTCGCGTTTTCTCGAACTCCTGCCGGTCGCTCCGCTCCCGCCAGTCGTGGA
>Fam_742_84_1  Nr. of seq. 1 Alignment length(with gaps) = 84 Alignment score = 0.000000
CTACAACAGGAGGAACTTCAGAAGGTTCATCTGATGCATCATCAACAGAAGATGTGATTTCAACAGATTCCTCTGACACATCAT
>Fam_743_84_1  Nr. of seq. 1 Alignment length(with gaps) = 84 Alignment score = 0.000000
ACCAACAAGGCCACCTACATAATCTCCAGTTCCGGTCACATCACCTGTGGAATATGAATCTGTAATAGTACCNCNATAATTTAT
>Fam_744_84_1  Nr. of seq. 1 Alignment length(with gaps) = 84 Alignment score = 0.000000
ACTTGATCGGTTAAACAAGGCGATAGAACTCGTNAATACCGAACTGGACCGGTTTAATAAGACACCGCAATTGATAGATACGGA
>Fam_745_84_1  Nr. of seq. 1 Alignment length(with gaps) = 84 Alignment score = 0.000000
TGATGAGAGAATAACTAAATTGGAGGAGTCTACGAAGAAGTTGGAGCAAGCTGTACAAGAGCTTATTGAGGCTCAGAAAAAACA
>Fam_746_79_1  Nr. of seq. 1 Alignment length(with gaps) = 79 Alignment score = 0.000000
TTGTTTATTATCTCTCCACTNCTCTACTNTTATGNTGATGGCNCCTCCTCNGTAGGATGCGNTGTTGTTTTTGAATGTG
>Fam_747_78_1  Nr. of seq. 1 Alignment length(with gaps) = 78 Alignment score = 0.000000
TTACAGCTTCAAGACGAACANTCNAATCAGNATCATTTTTAGCAANATNNNCTAAAACAGANTNATCATTTATCTTCC
>Fam_748_78_1  Nr. of seq. 1 Alignment length(with gaps) = 78 Alignment score = 0.000000
AAGAAAATGACTCAACGAGTTCAAAAACTAATGTATCTAACGGGTATAAACTAATAACAGTAAATAAAACAGTCGCTC
>Fam_749_75_1  Nr. of seq. 1 Alignment length(with gaps) = 75 Alignment score = 0.000000
CCACAGTCAAACTACCACCATTGCAGATAGCACCACCACTAACATCGGCTTTATTACCCGTGAATGTACTATTAC
>Fam_750_75_1  Nr. of seq. 1 Alignment length(with gaps) = 75 Alignment score = 0.000000
GGTGAACGTACACGAACGCTGCGCTGTACCGGCTATCACATTACTATGATACTCTCAAACTGTTATCTCTGTGGA
>Fam_751_75_1  Nr. of seq. 1 Alignment length(with gaps) = 75 Alignment score = 0.000000
CCGCTCGTGCCGCAACCCGTTCGACTCGCTCAGGCCGCCGCCTTCGCTTCGTCGAAGTCACAGCGTCACGTCACT
>Fam_752_73_1  Nr. of seq. 1 Alignment length(with gaps) = 73 Alignment score = 0.000000
```

```
 CCCCGCGAGTCCACTCCTCTCACTTCGTTCGAGTTGTTTCCTCGCTTGCCCCCGCTCGCTTCGCTCGCGGGGA
>Fam_753_73_1  Nr. of seq. 1 Alignment length(with gaps) = 73 Alignment score = 0.000000
 ATAGCTCCACCNACTGTTGCGGTGTTGTTGTNGAATGTACTGTTAGTTACAATTAAAGTTCCATNATTCGCTN
>Fam_754_73_1  Nr. of seq. 1 Alignment length(with gaps) = 73 Alignment score = 0.000000
 ATTCGAGAGCAAGATCCACTAAAACAAGGATTGAAACCNGTAATCATGACTNNTGANATTTAANCNTAACATT
>Fam_755_72_1  Nr. of seq. 1 Alignment length(with gaps) = 72 Alignment score = 0.000000
 TGAAATTGTTGAAAATCCGGAAAATAACACTTCCAATCAAACAGCTGCTGAAAACCTGACAGTTCCGGAAAC
>Fam_756_71_1  Nr. of seq. 1 Alignment length(with gaps) = 71 Alignment score = 0.000000
 CCCGCTCGCCCTTTCGCGGCCCGCTGCTCGCTTCGCTCCCAGCGCGCCGCGCTACTCACGGATCGTTCCTC
>Fam_757_71_1  Nr. of seq. 1 Alignment length(with gaps) = 71 Alignment score = 0.000000
 GCGGGCGACGCCATGTCGCTCGCTCGGAGACTATGCCGTGGCTGTGTGTAGCCGAACGTGGCACACGACCA
>Fam_758_71_1  Nr. of seq. 1 Alignment length(with gaps) = 71 Alignment score = 0.000000
 TTTTAGTAGATTAGCTTTTGCAATACANTAGANTAAACATNNNAAATGAGNGNANAATGTCGCAATCCTTG
>Fam_759_71_1  Nr. of seq. 1 Alignment length(with gaps) = 71 Alignment score = 0.000000
 TTCAATCCTTGTTTTCGTGGAGTCTACTTCTCGACATTANAAATGACTTANATGGTANGAGANATACGNGT
>Fam_760_69_1  Nr. of seq. 1 Alignment length(with gaps) = 69 Alignment score = 0.000000
 CTCGACGACGGTTCCGAAGTCGACGAGCTCGGGACGGATCCAGTGGTCGCCGACACGGACGATGACGGT
>Fam_761_69_1  Nr. of seq. 1 Alignment length(with gaps) = 69 Alignment score = 0.000000
 TTTGCGAATATGAAGTTGTNTTCGATTTCGGTGAAGTCAGATCCGTATACGCTTATTCCGTCATCTTCG
>Fam_762_69_1  Nr. of seq. 1 Alignment length(with gaps) = 69 Alignment score = 0.000000
 CAGCAGAAAATAAATTTGAANANGCAACAAAATATGCAGTGATGGCATTGGGTCAAATTGGGGAAAAAG
>Fam_763_69_1  Nr. of seq. 1 Alignment length(with gaps) = 69 Alignment score = 0.000000
 GGTCACGGATGCGAGCGATGAACAATCGTNGAACGCANNNTCCCCGATGGATGTTACCGAGTCGGGGAT
>Fam_764_69_1  Nr. of seq. 1 Alignment length(with gaps) = 69 Alignment score = 0.000000
 GAACTTAAAAACCTTACAACACTTGACTTATCTAGGAACCAGCTGACACAATTGCCACCNGAAATTGCA
>Fam_765_69_1  Nr. of seq. 1 Alignment length(with gaps) = 69 Alignment score = 0.000000
 CGATGCTGGTGACNCTGTTCGGNATGGTCACCGAGGTCAGGGCGGTGCAGCCGTAGAACGCACNGNNGC
>Fam_766_69_1  Nr. of seq. 1 Alignment length(with gaps) = 69 Alignment score = 0.000000
 ATGACGGCAATCCTTGCACTGCCGACTCCTGTGTGAATGGAGAATGCTACCATAGGCGAAAGAACTGCA
>Fam_767_68_1  Nr. of seq. 1 Alignment length(with gaps) = 68 Alignment score = 0.000000
 ATCTTAGTCTGATTTTAACGGCGNGCANCGAGNTCAAGATCGTCAAGGNAGGAGANGCTTTCAATCCT
>Fam_768_68_1  Nr. of seq. 1 Alignment length(with gaps) = 68 Alignment score = 0.000000
 CCCTCGCTTCGCTCGGCATGCCCTTCGCTGCTCGCGTGTCGCTACGCTCCCGCTCGCAAAAACGAGAC
>Fam_769_68_1  Nr. of seq. 1 Alignment length(with gaps) = 68 Alignment score = 0.000000
 AAAATAACGCCCCTAAGGGGATGGAAACATNATCTCNCNTCTTCACAACATNCAANNTGTTGTATTNC
>Fam_770_68_1  Nr. of seq. 1 Alignment length(with gaps) = 68 Alignment score = 0.000000
 TTATTCCAGAATCNGTTAAGTAGTATCGGAGAATNATGCTTTTAAAAATAATAAATTAACAGGCATAA
>Fam_771_68_1  Nr. of seq. 1 Alignment length(with gaps) = 68 Alignment score = 0.000000
```

```
 AGGATGCGACTGCCGGAACGGCAGGAGCTCGAGAAGATGCGAAGCATCTTCGAATGGCGACTGTCCGT
>Fam_772_68_1  Nr. of seq. 1 Alignment length(with gaps) = 68 Alignment score = 0.000000
 ATGGAGCAAATTAAGGACGGCTTGAAAACCGATCCAGAAATCTACTTCNTTGACGCGTAAGCTGCAAG
>Fam_773_68_1  Nr. of seq. 1 Alignment length(with gaps) = 68 Alignment score = 0.000000
 GTCTTATTGCAACATNTCGTCANACAGCAGAACCTNCAACTAGACCGTNCGGTTTCAATTCTCCTCGA
>Fam_774_68_1  Nr. of seq. 1 Alignment length(with gaps) = 68 Alignment score = 0.000000
 AGAAGAATTGAAATAATTGACCTTTTAGAGGACTATGGCATAAGTAAGGTTAGTTTCAATAAGACTTT
>Fam_775_67_1  Nr. of seq. 1 Alignment length(with gaps) = 67 Alignment score = 0.000000
 GTTCATTTCAAACCTTGTTATAGTGGATGTTGCCAAGCAACTGATAGATATTTTGTTAAATGAAATA
>Fam_776_67_1  Nr. of seq. 1 Alignment length(with gaps) = 67 Alignment score = 0.000000
 CTTTCAATTCTATTTTAGTCTTATTGGAACNNTNANNGATNCACNTGCTCNNAANNAGCNNNTNANN
>Fam_777_67_1  Nr. of seq. 1 Alignment length(with gaps) = 67 Alignment score = 0.000000
 CTCTTAGAGTCTTATTGAAACCANTACTCTGTNNANATGANCTNNTGAANCTTCANTNCTTTCAATT
>Fam_778_66_1  Nr. of seq. 1 Alignment length(with gaps) = 66 Alignment score = 0.000000
 ACCACATGGTACTTCTTCTACTGATNTGAAAANGANATANGNTAAAGTGANAGAAATNGTTTCCAC
>Fam_779_66_1  Nr. of seq. 1 Alignment length(with gaps) = 66 Alignment score = 0.000000
 CGTCAGTGTCNGCGNCTGTCGGGTTCGTGCCNATGGACAGCTCNCGCCCGTCGGNNACCCCGTCGN
>Fam_780_66_1  Nr. of seq. 1 Alignment length(with gaps) = 66 Alignment score = 0.000000
 GTTAAAATAAGACCGTCTCGGTATCTAATACAAAAANTNCTCATAGCTTNGATTNCGTCTTTAATT
>Fam_781_66_1  Nr. of seq. 1 Alignment length(with gaps) = 66 Alignment score = 0.000000
 GGAATAACCAAACTCTTCAACTGGTTATNTGAAAAAGCACCTCTNCCAATACTAGTTACNGAATCT
>Fam_782_66_1  Nr. of seq. 1 Alignment length(with gaps) = 66 Alignment score = 0.000000
 AACCAGATCAGCGATCTGAGCCCCCTCGCCGGACTGACCAACCTGGNGTATTTTCTAGATATATCC
>Fam_783_66_1  Nr. of seq. 1 Alignment length(with gaps) = 66 Alignment score = 0.000000
 GAGAATGCTGACGTTATTGATTCTGACGCTACTAATGATGATTTAAACGGAAATTCTGATTTAAAT
>Fam_784_65_1  Nr. of seq. 1 Alignment length(with gaps) = 65 Alignment score = 0.000000
 TCTACTATAGAATTGAAAGAAAGTTNNNAGAAGANTTTAATTACCATTCCGCNTGATGACGATAA
>Fam_785_65_1  Nr. of seq. 1 Alignment length(with gaps) = 65 Alignment score = 0.000000
 CCACAAGGGTTCGTCTGTAACTACGGTGAGGACGTGNACCGATNNCGTNCGACGNTGCTTCAACC
>Fam_786_65_1  Nr. of seq. 1 Alignment length(with gaps) = 65 Alignment score = 0.000000
 TCTGATTCTAACNNTTAANNTNNNTNANNANNTNNANNTNTAANNNAATTTCAATCCTAAAATAG
>Fam_787_65_1  Nr. of seq. 1 Alignment length(with gaps) = 65 Alignment score = 0.000000
 CTTTCAATCCTATTTAGGTTATTATTTAACNNNATNGGATTTCNNNNCCNTATCGAGATACTATA
>Fam_788_64_1  Nr. of seq. 1 Alignment length(with gaps) = 64 Alignment score = 0.000000
 GTTGCATCCCAAAAGGGATTGAAAGNAGATTNAGANNAGTAGTANCTGNNCTTNATGTCGGTTN
>Fam_789_64_1  Nr. of seq. 1 Alignment length(with gaps) = 64 Alignment score = 0.000000
 TTAAAATCAGACCTTAGAGGGATTGAAACNNANANNTNAANNTTNNAGNNNNTGNACATNATNG
>Fam_790_64_1  Nr. of seq. 1 Alignment length(with gaps) = 64 Alignment score = 0.000000
```

```
TCCGGAATCTTCAGCTCGTGACACACTCTTTAAAAAAAATCCTCAGCAGCTGAGTGACCGATTT
>Fam_791_64_1  Nr. of seq. 1 Alignment length(with gaps) = 64 Alignment score = 0.000000
CAATAAGACTCTAGGAGAATTGAAAATNATCANTGACGTNCTCAANGCNAGCTNANGCNNGTTG
>Fam_792_63_1  Nr. of seq. 1 Alignment length(with gaps) = 63 Alignment score = 0.000000
GAGGCACAGAAGAGAAGCGAGGAGAGACTAACTAGACTCGAGTCCGCGGTTGAGAAACTAGCC
>Fam_793_63_1  Nr. of seq. 1 Alignment length(with gaps) = 63 Alignment score = 0.000000
ACTAAAATCAGACCATTTTGGGATTGAAACTNANTNANANNATNTNNAGNNNNTNNATTNANN
>Fam_794_63_1  Nr. of seq. 1 Alignment length(with gaps) = 63 Alignment score = 0.000000
TTTTCGACCGGCGACTGGTGTTTCCGACAAGCGCCCAGCAGGAGCTGGAGAAAATGCGCAGCA
>Fam_795_63_1  Nr. of seq. 1 Alignment length(with gaps) = 63 Alignment score = 0.000000
TCTTATTGCAACGNACAGCACACTCAGGGGGNATCAGGCCCCTCTCTTTCAATTCTCTTAGAG
>Fam_796_63_1  Nr. of seq. 1 Alignment length(with gaps) = 63 Alignment score = 0.000000
GTTTGAGAGTGTTGTCAAATACGAGGTAACTCAATCANTATNGGCCGGGNNTANNCAGNCTTC
>Fam_797_62_1  Nr. of seq. 1 Alignment length(with gaps) = 62 Alignment score = 0.000000
CTAACAAAAGTAGCACAAACTTCTTTGATCAACCTTTCTGTCTAAGAAAGGTTGGCTTACAG
>Fam_798_61_1  Nr. of seq. 1 Alignment length(with gaps) = 61 Alignment score = 0.000000
TAAAAAGAAATAGTGTTTTTTAGTTAAATAAGGTGAAAATTGTTATTTTTAATTAAAAAGT
>Fam_799_61_1  Nr. of seq. 1 Alignment length(with gaps) = 61 Alignment score = 0.000000
GTTCACCCCCACGCCTGTGGGGACTTTNTCNNNNNNGNNANNGNNTNNNCTGNNNCNTNCG
>Fam_800_61_1  Nr. of seq. 1 Alignment length(with gaps) = 61 Alignment score = 0.000000
GAAGCCCCCCTTCAGTCACTAGATTTAGAAGATGTGGTGTTTAACCATCCATTATCTCAAA
>Fam_801_61_1  Nr. of seq. 1 Alignment length(with gaps) = 61 Alignment score = 0.000000
TTCATCCCCACGTGTGTGGGGAACTCNANTANNNTCANANNTNTCNNNANNNNTNAANCGG
>Fam_802_61_1  Nr. of seq. 1 Alignment length(with gaps) = 61 Alignment score = 0.000000
AACTCTGAAATATATTTCGCTCACGCTTTTTGATCAAACTTTTTTGGAAAAAGTTTGTTGA
>Fam_803_60_1  Nr. of seq. 1 Alignment length(with gaps) = 60 Alignment score = 0.000000
TTTTTAATGTTTGATTTGTTTTTGTAATTTATTTATTTTTTTTCTTCTTTTCTTTTTCAT
>Fam_804_60_1  Nr. of seq. 1 Alignment length(with gaps) = 60 Alignment score = 0.000000
CCTCCAGGCCTACTCGATCGGTTCCGGGAGCCCGTATCAGTACACATCGGAGTCCAGCGG
>Fam_805_60_1  Nr. of seq. 1 Alignment length(with gaps) = 60 Alignment score = 0.000000
TGTGGGGAACTCNNANTTCNCNTANCANTTTGACNNNNANNANCGGTTCATCCCCACGCC
>Fam_806_60_1  Nr. of seq. 1 Alignment length(with gaps) = 60 Alignment score = 0.000000
CAGGGCGACAGGCCCTACAAGCCCCGCGAAGGAGCAGCACCCACATCCGGCGACCGCCCC
>Fam_807_59_1  Nr. of seq. 1 Alignment length(with gaps) = 59 Alignment score = 0.000000
AAATTGACCAAATAGACGTTAATTTGATCAATAGATGTAAATCGATCAAATAAATTGTT
>Fam_808_59_1  Nr. of seq. 1 Alignment length(with gaps) = 59 Alignment score = 0.000000
TCACCTCGTGACGGTTCGTGTGCCCGGGACGCCCTCGGCGGCCAGACGTTCACGAAGCG
>Fam_809_58_1  Nr. of seq. 1 Alignment length(with gaps) = 58 Alignment score = 0.000000
```

GAGTTCCCCATGCATGTGGGGATAAACCGNNNATNNCATNNNANCNNNCNNANNNNTN
>Fam_810_58_1 Nr. of seq. 1 Alignment length(with gaps) = 58 Alignment score = 0.000000
TGGGGATGAACCGNNGANNNGACATGACATCCGNGNCNCANNGAGTTCCCCACGCATG
>Fam_811_58_1 Nr. of seq. 1 Alignment length(with gaps) = 58 Alignment score = 0.000000
TGTGGGGAACTCNCNNNTNNGNTCNGNNNANNNNNNGANNNCGGTTCATCCCCACGCT
>Fam_812_57_1 Nr. of seq. 1 Alignment length(with gaps) = 57 Alignment score = 0.000000
GAGTCGTCACCACTATCCTGGTTCGAGGAGTTATCACCGTCGTCGTTTTGACTCGAA
>Fam_813_57_1 Nr. of seq. 1 Alignment length(with gaps) = 57 Alignment score = 0.000000
ACTTCTGAAAGGTACTTTTGTGAATCTTCAATTGCCTTGAACATTGAAGGGGAATAA
>Fam_814_57_1 Nr. of seq. 1 Alignment length(with gaps) = 57 Alignment score = 0.000000
AATAAGTCAAAAACCGGAACCGGAACAAGTACCGAAACCCCGGAAACAGTAATAGAT
>Fam_815_57_1 Nr. of seq. 1 Alignment length(with gaps) = 57 Alignment score = 0.000000
GGTTCATCCCCATACACACGGGGAACTCNNTNNATNNNCNNNTNNGNNTNNTNTTNC
>Fam_816_56_1 Nr. of seq. 1 Alignment length(with gaps) = 56 Alignment score = 0.000000
ATCAGTGAAGTTGTTTTGAACGTCTGCATCATTGCTGAACTCACAGCTCTATTCAC
>Fam_817_54_1 Nr. of seq. 1 Alignment length(with gaps) = 54 Alignment score = 0.000000
TAGGGATGATATCTGTGATTCTAATGTGGAGTTAACGTTTGATAGTGAAGTGCT
>Fam_818_54_1 Nr. of seq. 1 Alignment length(with gaps) = 54 Alignment score = 0.000000
TCGCCGTCCTTGGTTCGAACATCGGCGTCNCCTGTCGTCTTGCGGGCGGTGGCG
>Fam_819_54_1 Nr. of seq. 1 Alignment length(with gaps) = 54 Alignment score = 0.000000
CCGCGAGGTCGACCAGCGGGAGACCTCGGAACGAGCGAGCGGTGAAACCGTGAG
>Fam_820_54_1 Nr. of seq. 1 Alignment length(with gaps) = 54 Alignment score = 0.000000
CCAAAGCCCCAGCCGCTCGGTAGTACGTGATCGACGACTCCGCGGTGAACACCG
>Fam_821_54_1 Nr. of seq. 1 Alignment length(with gaps) = 54 Alignment score = 0.000000
GATTCTCCTTTAATGTCTGATTCTGTCATTTCTCCTTCCTTACCTGATCCATTG
>Fam_822_53_1 Nr. of seq. 1 Alignment length(with gaps) = 53 Alignment score = 0.000000
AAATAAAAAATTAGATTAAATTCCTAAGGTAAAAAATACCAATATAGGAATTA
>Fam_823_52_1 Nr. of seq. 1 Alignment length(with gaps) = 52 Alignment score = 0.000000
CATATGAAATTATGTTTAGAAGCAGATCTTAGGAACCTAATTTTCTAAGTTA
>Fam_824_52_1 Nr. of seq. 1 Alignment length(with gaps) = 52 Alignment score = 0.000000
TTGTTTTGTAATGAGTTTTGGTATGGTTTTTTTAGTTGTTTGGGGATTTGT
>Fam_825_51_1 Nr. of seq. 1 Alignment length(with gaps) = 51 Alignment score = 0.000000
ATAAAGATTTAGAAAAATAGTTTAAAAAAAATAACTAAAATAACTTAAATT
>Fam_826_50_1 Nr. of seq. 1 Alignment length(with gaps) = 50 Alignment score = 0.000000
TTTGAACCTTATCTGGAATACTTTTGACCCTGGTCTGCTGACAGAATGCG
>Fam_827_50_1 Nr. of seq. 1 Alignment length(with gaps) = 50 Alignment score = 0.000000
CTAGAACATCTCCCTCTCATGCTCCAGATGGTGTTCCGAAGTAACCTCTG
>Fam_828_49_1 Nr. of seq. 1 Alignment length(with gaps) = 49 Alignment score = 0.000000

GTATTTCGAAACCCTTTGGGTTTCTCAAACTCCGTTCTGTCGGAACGTC
>Fam_829_49_1 Nr. of seq. 1 Alignment length(with gaps) = 49 Alignment score = 0.000000
GAGGAGAAGCTTGAATTTAAGAGGAAGTGGGGTATTGAGTAGTTTTTT
>Fam_830_48_1 Nr. of seq. 1 Alignment length(with gaps) = 48 Alignment score = 0.000000
GGTTCCACGAGCCCCCGCTCGCAGGCTCGCGGGGACGCCGTAGAACAC
>Fam_831_48_1 Nr. of seq. 1 Alignment length(with gaps) = 48 Alignment score = 0.000000
AAAAAATAGGCGAATTTTATTTTTGTTTATTTAAACTTTAAGAAAAGG
>Fam_832_48_1 Nr. of seq. 1 Alignment length(with gaps) = 48 Alignment score = 0.000000
TAAAATAAGTAAAATGTCTACTTTATTAATTAATGAAGTATAAAGCCT
>Fam_833_48_1 Nr. of seq. 1 Alignment length(with gaps) = 48 Alignment score = 0.000000
ATAAAAATAAAACCTAAAATAGAACAAAAAAAAAGATTCAAAAACTGA
>Fam_834_48_1 Nr. of seq. 1 Alignment length(with gaps) = 48 Alignment score = 0.000000
CAATTAATGAAGATGAATTTACAGCAACTGAGGAACCTGTGTTCCCAT
>Fam_835_48_1 Nr. of seq. 1 Alignment length(with gaps) = 48 Alignment score = 0.000000
GAGAAGACGAAGTCTTCGAGATTCGAGAGGTTATCAAACCTCGAGACG
>Fam_836_48_1 Nr. of seq. 1 Alignment length(with gaps) = 48 Alignment score = 0.000000
CATCGGCTCGCCGATAGATCCGCTGAAGGACTCGTTCATCGATCCATT
>Fam_837_48_1 Nr. of seq. 1 Alignment length(with gaps) = 48 Alignment score = 0.000000
GTTTCATTATCAGATTCTATTTTTGTATTTTCCGAGGAATTTGTTTCG
>Fam_838_48_1 Nr. of seq. 1 Alignment length(with gaps) = 48 Alignment score = 0.000000
GGAACAGGACTCGGAACAGCGGTAGCATCTACAACCGGAACAGCAGTG
>Fam_839_47_1 Nr. of seq. 1 Alignment length(with gaps) = 47 Alignment score = 0.000000
GTTTTTTAATAATTTGAAATAATATTAATTTTTATTTTTAATTAGGT
>Fam_840_47_1 Nr. of seq. 1 Alignment length(with gaps) = 47 Alignment score = 0.000000
TCGCGGCTCGAAGCCTACGGCTTCTCACGCTCCCGTCCTGTGGACAG
>Fam_841_47_1 Nr. of seq. 1 Alignment length(with gaps) = 47 Alignment score = 0.000000
TTTGTATTCCTTATTTCAGATCTTCAGTGTTTCTCATTTCTAGACAA
>Fam_842_46_1 Nr. of seq. 1 Alignment length(with gaps) = 46 Alignment score = 0.000000
AAAAATTAATGAAATTATGATTGAAGGAATAAAAAAATTTTATCTA
>Fam_843_46_1 Nr. of seq. 1 Alignment length(with gaps) = 46 Alignment score = 0.000000
GTAATAATTCTATGTCTCTTTATCAATATAGAAAGTAATGATAAAA
>Fam_844_45_1 Nr. of seq. 1 Alignment length(with gaps) = 45 Alignment score = 0.000000
TGGACGTCTCCGGGACGACGGTCCCGATCCAGGAAGACACCGCTC
>Fam_845_45_1 Nr. of seq. 1 Alignment length(with gaps) = 45 Alignment score = 0.000000
ACGTCGAACACGAGGTGTTCCATCGACTCGAGAACTACGGGCACG
>Fam_846_45_1 Nr. of seq. 1 Alignment length(with gaps) = 45 Alignment score = 0.000000
CAACAACTACTGTAGAACCGACTACCACTCAAACAACCGAAACCA
>Fam_847_45_1 Nr. of seq. 1 Alignment length(with gaps) = 45 Alignment score = 0.000000

GCTCTCTTCGGTCGCCCACATCGCTCGCGGCGTCCCTCGCGCGGC
>Fam_848_45_1 Nr. of seq. 1 Alignment length(with gaps) = 45 Alignment score = 0.000000
ATTAGTTTTTTAAAATGAAAAAATTAAGATAGCTATTTTTATATT
>Fam_849_45_1 Nr. of seq. 1 Alignment length(with gaps) = 45 Alignment score = 0.000000
TGATTCTTTTTTTTTAAAATTGTTTTTAATATTTAATCACTATTC
>Fam_850_45_1 Nr. of seq. 1 Alignment length(with gaps) = 45 Alignment score = 0.000000
CCTAATTTAGTTTTTAAAAATATTATAAAATTTAATTTTAATTAG
>Fam_851_45_1 Nr. of seq. 1 Alignment length(with gaps) = 45 Alignment score = 0.000000
AAGATAATCTTAAAATAATTTAAAATCTTATAAATTAAGAATTAA
>Fam_852_45_1 Nr. of seq. 1 Alignment length(with gaps) = 45 Alignment score = 0.000000
CCGCACCTACGGTGCCTCAACTCCGCTCTTTCAGAGCATCGCACT
>Fam_853_45_1 Nr. of seq. 1 Alignment length(with gaps) = 45 Alignment score = 0.000000
ATGGTTCTGTTTCCGTTTCAGTGGGCTCTTCTAATGGCTCTTCCA
>Fam_854_45_1 Nr. of seq. 1 Alignment length(with gaps) = 45 Alignment score = 0.000000
ACTTCAAGTGTCAACCTTAATGGAGAAGATAATNCAGGAATAAAT
>Fam_855_45_1 Nr. of seq. 1 Alignment length(with gaps) = 45 Alignment score = 0.000000
ATGATAGGATAGCCCAGTCCCAGTCAGCAATCTCAGATCAAGTCA
>Fam_856_44_1 Nr. of seq. 1 Alignment length(with gaps) = 44 Alignment score = 0.000000
TTTTTAAAGTTAATTAGATTTAAAAAGGATTAATTAAGTTTTCT
>Fam_857_44_1 Nr. of seq. 1 Alignment length(with gaps) = 44 Alignment score = 0.000000
GTTTTGTTATGACTTACCTATTACTTTATAGTTGCTTAATGGTA
>Fam_858_44_1 Nr. of seq. 1 Alignment length(with gaps) = 44 Alignment score = 0.000000
TCTCCTCTGTCATTGCTTCCTAAATTCAACAATAGCCTTTGTGT
>Fam_859_43_1 Nr. of seq. 1 Alignment length(with gaps) = 43 Alignment score = 0.000000
TTTATAATATTTTACTAAAACAAACTAACATTATGTAAACATA
>Fam_860_43_1 Nr. of seq. 1 Alignment length(with gaps) = 43 Alignment score = 0.000000
AACCAAATTAAATTAAGTAAATTGTATCAGATCAATTAAATTA
>Fam_861_42_1 Nr. of seq. 1 Alignment length(with gaps) = 42 Alignment score = 0.000000
GACGACACCTTCGAGGAGACGGCAACGGAAACGGTCACCGAG
>Fam_862_42_1 Nr. of seq. 1 Alignment length(with gaps) = 42 Alignment score = 0.000000
GGGTCCAGGGAGTAGACAACTCTCCTCAGAGGGTAGACAACA
>Fam_863_42_1 Nr. of seq. 1 Alignment length(with gaps) = 42 Alignment score = 0.000000
AACCAAGAAAAAACAAACAAATTCATAGAAAAATACGAACAC
>Fam_864_42_1 Nr. of seq. 1 Alignment length(with gaps) = 42 Alignment score = 0.000000
TTGTCTTTTTGTAGGGTTTCTATTTGATTATTGAGCTTATTT
>Fam_865_42_1 Nr. of seq. 1 Alignment length(with gaps) = 42 Alignment score = 0.000000
CTAATTCATATAATGATTTAAGTGTTGGCTTTGAATCTGATG
>Fam_866_42_1 Nr. of seq. 1 Alignment length(with gaps) = 42 Alignment score = 0.000000

AGAAATTAAGTGATGCCTTAGAGAATAGTGAAAAGGAACGGC
>Fam_867_42_1 Nr. of seq. 1 Alignment length(with gaps) = 42 Alignment score = 0.000000
GTCTTTTCAGTGTTGACTTTNGCTGAACTGTTTGTAGTGTTA
>Fam_868_42_1 Nr. of seq. 1 Alignment length(with gaps) = 42 Alignment score = 0.000000
TTCTAGATTCCGAAGCTGAAGCTGATTTTGGTAACGACATAA
>Fam_869_42_1 Nr. of seq. 1 Alignment length(with gaps) = 42 Alignment score = 0.000000
CTTTCTGAATTTAGCTCAATCATTCCAACCTTAATTCCTATA
>Fam_870_42_1 Nr. of seq. 1 Alignment length(with gaps) = 42 Alignment score = 0.000000
TTAAAAACTAAAATTAGTAACCTACATACACGACTATCTTTA
>Fam_871_42_1 Nr. of seq. 1 Alignment length(with gaps) = 42 Alignment score = 0.000000
TCTTCCTTTTCAGTTGCAGTTTCTGCTTCAGTCTCTACTTTT
>Fam_872_42_1 Nr. of seq. 1 Alignment length(with gaps) = 42 Alignment score = 0.000000
ACTGGAAATTAAACAAACTTTAATTATATAAACTGTAATTAT
>Fam_873_42_1 Nr. of seq. 1 Alignment length(with gaps) = 42 Alignment score = 0.000000
TTTTCTCTTTTTTCAGTTATATTCTCACTTAAATTTGATAAA
>Fam_874_42_1 Nr. of seq. 1 Alignment length(with gaps) = 42 Alignment score = 0.000000
AAAAGTTCAACTCNTCTGAAATTGGAACNATGCAGAACTCTN
>Fam_875_42_1 Nr. of seq. 1 Alignment length(with gaps) = 42 Alignment score = 0.000000
TTTCCGCTGTTCTTTGTTCCGCAGTGGGTGTGAGAGAGTACG
>Fam_876_42_1 Nr. of seq. 1 Alignment length(with gaps) = 42 Alignment score = 0.000000
CCGATTGCTCCGTAGCTACTGACTCTACCGTCTCCGACAACT
>Fam_877_42_1 Nr. of seq. 1 Alignment length(with gaps) = 42 Alignment score = 0.000000
TCACTCCAAGAAGAGGTGAAGAGACAAGGTGAAGCCATTGTA
>Fam_878_42_1 Nr. of seq. 1 Alignment length(with gaps) = 42 Alignment score = 0.000000
TCCTGCCATAGCCTCTCNATGTTTTCGTTAATNCTCCTATTC
>Fam_879_41_1 Nr. of seq. 1 Alignment length(with gaps) = 41 Alignment score = 0.000000
CGATCCGGCTCGCTTGACTCACGCCGGATCGGTGGAGATGT
>Fam_880_41_1 Nr. of seq. 1 Alignment length(with gaps) = 41 Alignment score = 0.000000
ATTATTCCAATCATTTTTAAGAGTATTTCAGAGGCATTTTT
>Fam_881_41_1 Nr. of seq. 1 Alignment length(with gaps) = 41 Alignment score = 0.000000
TGAAATTACTCATTTACCTTACGTTTCCTTAATGATCTAAA
>Fam_882_41_1 Nr. of seq. 1 Alignment length(with gaps) = 41 Alignment score = 0.000000
TCTTCGAAGAATCTTCTGGATTCTTCTCACGTCTTTGAGAC
>Fam_883_40_1 Nr. of seq. 1 Alignment length(with gaps) = 40 Alignment score = 0.000000
AAGTTATATAAACAAGTATCTTTACTAATTGTTTATAAGA
>Fam_884_40_1 Nr. of seq. 1 Alignment length(with gaps) = 40 Alignment score = 0.000000
AGTTTATTTTACTTTAATTACTCTCTATTTTTCTAAAATC
>Fam_885_40_1 Nr. of seq. 1 Alignment length(with gaps) = 40 Alignment score = 0.000000

```
 TTTCTAAAATTAGTTAATATTTTTTTTATTTAGATTATTT
>Fam_886_40_1  Nr. of seq. 1 Alignment length(with gaps) = 40 Alignment score = 0.000000
 TAATAATTTCGAAAACAAGAGTCCTGGTATATCTTGGAAC
>Fam_887_40_1  Nr. of seq. 1 Alignment length(with gaps) = 40 Alignment score = 0.000000
 AGACTACTTCATGGACATTGATATTGAAATTCGTTCAAGC
>Fam_888_40_1  Nr. of seq. 1 Alignment length(with gaps) = 40 Alignment score = 0.000000
 CGGCCTCGCCCCGGTAAGTCGATCCGCGTCGGCCCGACAT
>Fam_889_39_1  Nr. of seq. 1 Alignment length(with gaps) = 39 Alignment score = 0.000000
 GGGGTGTGTCGGGGCGGGGCCGAGCGGCGTGGGCTCGAT
>Fam_890_39_1  Nr. of seq. 1 Alignment length(with gaps) = 39 Alignment score = 0.000000
 CACGAGACGATAATGCGGCGGAGACGAACGCGAGTACGC
>Fam_891_39_1  Nr. of seq. 1 Alignment length(with gaps) = 39 Alignment score = 0.000000
 CCGTCACCGTCNGTGTCAGATNCTGCAGTATCAACGTAG
>Fam_892_39_1  Nr. of seq. 1 Alignment length(with gaps) = 39 Alignment score = 0.000000
 TTAAATAAATGAGATATTCCTTTTTTATATGGAAAATCT
>Fam_893_39_1  Nr. of seq. 1 Alignment length(with gaps) = 39 Alignment score = 0.000000
 TCTGTCCAGTTGANTGTTAGGCTGTCTCCNCCTAATAGG
>Fam_894_39_1  Nr. of seq. 1 Alignment length(with gaps) = 39 Alignment score = 0.000000
 CGCCGCTGCTTCCACCACTGCTACGGTCGCTGCTTCTTC
>Fam_895_39_1  Nr. of seq. 1 Alignment length(with gaps) = 39 Alignment score = 0.000000
 GTCGAGGTTCCGGTCGTCGCTGATACCGAGGCCGAGGAT
>Fam_896_39_1  Nr. of seq. 1 Alignment length(with gaps) = 39 Alignment score = 0.000000
 CAGAGGATCTTGCGCCTGAGGGATCTGAGGAGCCGGTTG
>Fam_897_39_1  Nr. of seq. 1 Alignment length(with gaps) = 39 Alignment score = 0.000000
 TCTCCACCATTATTGGTCTCCGTGACGGTGGCCGTATCC
>Fam_898_39_1  Nr. of seq. 1 Alignment length(with gaps) = 39 Alignment score = 0.000000
 TTTTGTTTTTGAGCATCTGCTACGGTACTAGTATCTGTG
>Fam_899_39_1  Nr. of seq. 1 Alignment length(with gaps) = 39 Alignment score = 0.000000
 TCACAGCAGTCGCCTTCACAGTCTGCCTCGGTACAGTCG
>Fam_900_39_1  Nr. of seq. 1 Alignment length(with gaps) = 39 Alignment score = 0.000000
 CAGCATTGTCACATGTTTTTTCTTCTGTTGAGGTATCTG
>Fam_901_39_1  Nr. of seq. 1 Alignment length(with gaps) = 39 Alignment score = 0.000000
 GGTTTTTTATTTGGTTTCACATTTTCGCTATTGATATCT
>Fam_902_39_1  Nr. of seq. 1 Alignment length(with gaps) = 39 Alignment score = 0.000000
 AATACAGTCATACACAATAATTCATGCAGTTGTCATCAC
>Fam_903_39_1  Nr. of seq. 1 Alignment length(with gaps) = 39 Alignment score = 0.000000
 TCCTGAATCATCATCCCAAGAACTGCCTCCATCATCACT
>Fam_904_39_1  Nr. of seq. 1 Alignment length(with gaps) = 39 Alignment score = 0.000000
```

```
AACCGGCACAGAGCCCGAAGCGCCCGACGACGGCGTCGA
>Fam_905_38_1  Nr. of seq. 1 Alignment length(with gaps) = 38 Alignment score = 0.000000
TGGACCGTTGTCCCAGTGCGGTTGTCAATCACCGCCCA
>Fam_906_38_1  Nr. of seq. 1 Alignment length(with gaps) = 38 Alignment score = 0.000000
AAAATTAAAAAATTAAAAATAATATACTATAAAAATAG
>Fam_907_38_1  Nr. of seq. 1 Alignment length(with gaps) = 38 Alignment score = 0.000000
TAATAAATAGTAAACCAAGCTTCCACTTAAAAAATTTC
>Fam_908_38_1  Nr. of seq. 1 Alignment length(with gaps) = 38 Alignment score = 0.000000
TTAAACTTTTAAAACTGATTAAATTCTTGAATAATTAT
>Fam_909_38_1  Nr. of seq. 1 Alignment length(with gaps) = 38 Alignment score = 0.000000
CAAGGTTTATTGGTTAGATCAAGATTTACTGATTAGAC
>Fam_910_38_1  Nr. of seq. 1 Alignment length(with gaps) = 38 Alignment score = 0.000000
TTAAACTTTATACGGTTTTAACAGTACTGAAATTTCTC
>Fam_911_37_1  Nr. of seq. 1 Alignment length(with gaps) = 37 Alignment score = 0.000000
CGGGGCGTATTCTTCCACCACCCCGGCTGTCCGGTGC
>Fam_912_37_1  Nr. of seq. 1 Alignment length(with gaps) = 37 Alignment score = 0.000000
ATTTTCTTATTTTAACAAATCATATTTATTTTATTTA
>Fam_913_37_1  Nr. of seq. 1 Alignment length(with gaps) = 37 Alignment score = 0.000000
AAAGAATAAAAAATATTCTCTAAAAATGTTTAAAACT
>Fam_914_37_1  Nr. of seq. 1 Alignment length(with gaps) = 37 Alignment score = 0.000000
AAAAACATGAAACACCAGAAAAAATCACAATGCCCCA
>Fam_915_37_1  Nr. of seq. 1 Alignment length(with gaps) = 37 Alignment score = 0.000000
ATTTATTAAATATCTTTATAATATTTTTTAGAACTAG
>Fam_916_36_1  Nr. of seq. 1 Alignment length(with gaps) = 36 Alignment score = 0.000000
GGTCAGCCGGCGATGTCGGGAGTAGAAGCCGACAAC
>Fam_917_36_1  Nr. of seq. 1 Alignment length(with gaps) = 36 Alignment score = 0.000000
ATCTTTAGACCTGTCTTTCCTAGATTTATTAAAATT
>Fam_918_36_1  Nr. of seq. 1 Alignment length(with gaps) = 36 Alignment score = 0.000000
AATTTAAAATATTTATACTTAATAAGTTTTTTAAAG
>Fam_919_36_1  Nr. of seq. 1 Alignment length(with gaps) = 36 Alignment score = 0.000000
TTCTTTATTGGAGGTTATGGTTGTGTTCTTGTTTTC
>Fam_920_36_1  Nr. of seq. 1 Alignment length(with gaps) = 36 Alignment score = 0.000000
AATTAAAACTAGAATTAAGTGAAATTTGAATAATTC
>Fam_921_36_1  Nr. of seq. 1 Alignment length(with gaps) = 36 Alignment score = 0.000000
CTGCTGATTCCGATGACATTGTTGAATCTGTTGATG
>Fam_922_36_1  Nr. of seq. 1 Alignment length(with gaps) = 36 Alignment score = 0.000000
AAACCTTCACTCTGACNTCCATCGGTTNTTGCNTNA
>Fam_923_36_1  Nr. of seq. 1 Alignment length(with gaps) = 36 Alignment score = 0.000000
```

```
 CGAAACAGACAGCGTACTGCAGAGCCGCAACTCTGC
>Fam_924_36_1  Nr. of seq. 1 Alignment length(with gaps) = 36 Alignment score = 0.000000
 TTGTCTTCAACTGTCATATTGTCAGCTGCAGAGTCA
>Fam_925_36_1  Nr. of seq. 1 Alignment length(with gaps) = 36 Alignment score = 0.000000
 CTGTAAGCTGACCTATAAGCTAACTTATAAGTTGAC
>Fam_926_36_1  Nr. of seq. 1 Alignment length(with gaps) = 36 Alignment score = 0.000000
 CCAACCACTGTGTCGACTCCAATACCGACTGCAATC
>Fam_927_36_1  Nr. of seq. 1 Alignment length(with gaps) = 36 Alignment score = 0.000000
 CGTCTTCGTACGGTTCGATGTCGGTCGGCTGATCCT
>Fam_928_36_1  Nr. of seq. 1 Alignment length(with gaps) = 36 Alignment score = 0.000000
 TCGCTTTCGTCTCCGTCCTCTCTCTCACCGTCTTCC
>Fam_929_35_1  Nr. of seq. 1 Alignment length(with gaps) = 35 Alignment score = 0.000000
 GGCGGCGTCGACAGACGGGACGCCGGCACCTCTCA
>Fam_930_35_1  Nr. of seq. 1 Alignment length(with gaps) = 35 Alignment score = 0.000000
 AAATATAATAAAACTAAATAGCCCATTAAGTATTC
>Fam_931_35_1  Nr. of seq. 1 Alignment length(with gaps) = 35 Alignment score = 0.000000
 AAAAATATCATACTCATTCGAGTTAATATAATATT
>Fam_932_35_1  Nr. of seq. 1 Alignment length(with gaps) = 35 Alignment score = 0.000000
 CAGTTTTGAATATTATATCAGTGTGTAAGTTTCTT
>Fam_933_35_1  Nr. of seq. 1 Alignment length(with gaps) = 35 Alignment score = 0.000000
 AAATAAGATTCTGTTTTTGAGAGATTCAGTAAATA
>Fam_934_35_1  Nr. of seq. 1 Alignment length(with gaps) = 35 Alignment score = 0.000000
 GTTTTATTCTTAGATTCATATTTTGTTGTCTTCAG
>Fam_935_34_1  Nr. of seq. 1 Alignment length(with gaps) = 34 Alignment score = 0.000000
 CGTTCAGCCGAGTCGTCTGTTCGATGCGACTCGA
>Fam_936_34_1  Nr. of seq. 1 Alignment length(with gaps) = 34 Alignment score = 0.000000
 CATTGGAGTGTAATAAAAAAGGTGATTTTTATTA
>Fam_937_34_1  Nr. of seq. 1 Alignment length(with gaps) = 34 Alignment score = 0.000000
 TTATTTTTTTATCCAATCTTTTATATACTATTGG
>Fam_938_34_1  Nr. of seq. 1 Alignment length(with gaps) = 34 Alignment score = 0.000000
 AAAATAGTATAGGAGTATATAAAGGCTTATACGA
>Fam_939_34_1  Nr. of seq. 1 Alignment length(with gaps) = 34 Alignment score = 0.000000
 CTTGATCGAAACTCACTTTTCCAATAATAGTCTG
>Fam_940_34_1  Nr. of seq. 1 Alignment length(with gaps) = 34 Alignment score = 0.000000
 TCCTTTCTATTCTTCTCTTCTTTTCTAAGACAAA
>Fam_941_34_1  Nr. of seq. 1 Alignment length(with gaps) = 34 Alignment score = 0.000000
 AAAGTAAGTAACCAAAGGAGTTCTTTAAAGAAAT
>Fam_942_33_1  Nr. of seq. 1 Alignment length(with gaps) = 33 Alignment score = 0.000000
```

CGTCGAAGGTCGCATCCCCGAAGANCGCCGTAT
>Fam_943_33_1 Nr. of seq. 1 Alignment length(with gaps) = 33 Alignment score = 0.000000
TCATCTATCCTCCTGTTTAGCCTNGCTTCAACN
>Fam_944_33_1 Nr. of seq. 1 Alignment length(with gaps) = 33 Alignment score = 0.000000
AAATCTAAAGCCGAAGATATNTCTGAAGATGTN
>Fam_945_33_1 Nr. of seq. 1 Alignment length(with gaps) = 33 Alignment score = 0.000000
AACGGAACCAGTATAGACATATCNAGTCTATTN
>Fam_946_33_1 Nr. of seq. 1 Alignment length(with gaps) = 33 Alignment score = 0.000000
CAGCTACAGAACCCCGAACATCCTCCAAATCAC
>Fam_947_33_1 Nr. of seq. 1 Alignment length(with gaps) = 33 Alignment score = 0.000000
ACAAAAGAAAAACTAGACCACAGAATAGACAAC
>Fam_948_33_1 Nr. of seq. 1 Alignment length(with gaps) = 33 Alignment score = 0.000000
TCGGTTAGATGATTTTTAATATGAAAGCAATGC
>Fam_949_33_1 Nr. of seq. 1 Alignment length(with gaps) = 33 Alignment score = 0.000000
AATCTTCAGCTNGCTGAGCCACACATTTTCTGA
>Fam_950_33_1 Nr. of seq. 1 Alignment length(with gaps) = 33 Alignment score = 0.000000
ACTGAGCTAAAGGAAGACTGAGACGGAAGGAAA
>Fam_951_33_1 Nr. of seq. 1 Alignment length(with gaps) = 33 Alignment score = 0.000000
GCAGAATCGCCAAAAGTAGAATACCGAAAANAG
>Fam_952_33_1 Nr. of seq. 1 Alignment length(with gaps) = 33 Alignment score = 0.000000
TTTGTATATCAGATTGCGCTTATTAGGTCAGTA
>Fam_953_33_1 Nr. of seq. 1 Alignment length(with gaps) = 33 Alignment score = 0.000000
TAATAGCCTGTTAGAACAGCTTACTTAAAACAA
>Fam_954_33_1 Nr. of seq. 1 Alignment length(with gaps) = 33 Alignment score = 0.000000
AAATTCTGCTCTTAAACTCTTAGTAAGTTCACT
>Fam_955_33_1 Nr. of seq. 1 Alignment length(with gaps) = 33 Alignment score = 0.000000
ATAACCAGTCCCCCAGAGGTCACNAGTCCCGGC
>Fam_956_33_1 Nr. of seq. 1 Alignment length(with gaps) = 33 Alignment score = 0.000000
TTCAACATTTAAAGGAGGAGTTTATTTTATTTT
>Fam_957_33_1 Nr. of seq. 1 Alignment length(with gaps) = 33 Alignment score = 0.000000
GACCCGTCCGAATCATCCGATCCATCATCGTCC
>Fam_958_33_1 Nr. of seq. 1 Alignment length(with gaps) = 33 Alignment score = 0.000000
GACGACGAAGATGACGAGATGAACGACGAGGTT
>Fam_959_33_1 Nr. of seq. 1 Alignment length(with gaps) = 33 Alignment score = 0.000000
GTCGAATCGCGTTACTCGGCGTGAAAAGCGGCG
>Fam_960_33_1 Nr. of seq. 1 Alignment length(with gaps) = 33 Alignment score = 0.000000
CGTCGGCGTCTTCCGCGTCGGCACCGTGTTCGT
>Fam_961_33_1 Nr. of seq. 1 Alignment length(with gaps) = 33 Alignment score = 0.000000

GTCGTCCTTCTTGTCGTCAGCGTGCTTGCGGTC
>Fam_962_32_1 Nr. of seq. 1 Alignment length(with gaps) = 32 Alignment score = 0.000000
TTAATTTAAAGTAACTAAAAATTATAAATCAG
>Fam_963_32_1 Nr. of seq. 1 Alignment length(with gaps) = 32 Alignment score = 0.000000
AATCCATTATTTCATAATTTAACCACTAAAAG
>Fam_964_32_1 Nr. of seq. 1 Alignment length(with gaps) = 32 Alignment score = 0.000000
TAACCACAACGGCACTACGGCACGGCGATTGG
>Fam_965_32_1 Nr. of seq. 1 Alignment length(with gaps) = 32 Alignment score = 0.000000
TTCTGAGAATCCAATTTAATCTTTTTAAATAA
>Fam_966_32_1 Nr. of seq. 1 Alignment length(with gaps) = 32 Alignment score = 0.000000
TGAAAACTTAGTCTCCTTAAACCTTAAACTCC
>Fam_967_32_1 Nr. of seq. 1 Alignment length(with gaps) = 32 Alignment score = 0.000000
GATCTTTTCTTGCCTTCTTACATTCTTCTTTT
>Fam_968_31_1 Nr. of seq. 1 Alignment length(with gaps) = 31 Alignment score = 0.000000
ATCAATCCATGTCTACTTAAGAAATGCTATT
>Fam_969_31_1 Nr. of seq. 1 Alignment length(with gaps) = 31 Alignment score = 0.000000
AAAAAAAGATAATTGGGTGTAGTTTCTATTT
>Fam_970_31_1 Nr. of seq. 1 Alignment length(with gaps) = 31 Alignment score = 0.000000
TGTGGCTTTTATTTTTTTGGGCATTATATGT
>Fam_971_31_1 Nr. of seq. 1 Alignment length(with gaps) = 31 Alignment score = 0.000000
CCACAGAGGACACAGAGGAATTAGATAATCA
>Fam_972_31_1 Nr. of seq. 1 Alignment length(with gaps) = 31 Alignment score = 0.000000
GTTTACAACCCCTAATGCCAATAAAAATATG
>Fam_973_31_1 Nr. of seq. 1 Alignment length(with gaps) = 31 Alignment score = 0.000000
CTGGTTTACTTCCGGCTTATTCTAGCTTATT
>Fam_974_31_1 Nr. of seq. 1 Alignment length(with gaps) = 31 Alignment score = 0.000000
CCTACTATAATTAGAGTAGACATATACCAAG
>Fam_975_31_1 Nr. of seq. 1 Alignment length(with gaps) = 31 Alignment score = 0.000000
TTTGGAGGTTTTACCGGTTTTTTGTTGGATT
>Fam_976_31_1 Nr. of seq. 1 Alignment length(with gaps) = 31 Alignment score = 0.000000
TTATTATTTCTCTCTTACTTCCCTTTTTCTC
>Fam_977_31_1 Nr. of seq. 1 Alignment length(with gaps) = 31 Alignment score = 0.000000
ATGTTAAGTCCATAAACTAAGTCCATGTACT
>Fam_978_30_1 Nr. of seq. 1 Alignment length(with gaps) = 30 Alignment score = 0.000000
CTCGCCAAACCCTCAACCGACAACCATCTA
>Fam_979_30_1 Nr. of seq. 1 Alignment length(with gaps) = 30 Alignment score = 0.000000
GTGGTCGAACGGATGGTAGATGGACCGGCA
>Fam_980_30_1 Nr. of seq. 1 Alignment length(with gaps) = 30 Alignment score = 0.000000

```
GCGGAGGCGAAGGCGAAGGAGAATGCAGAT
>Fam_981_30_1  Nr. of seq. 1 Alignment length(with gaps) = 30 Alignment score = 0.000000
CACGACCACTCTCACGGCGCAGGCCACACG
>Fam_982_30_1  Nr. of seq. 1 Alignment length(with gaps) = 30 Alignment score = 0.000000
GGCTGATCTGGCGTGTCAGTTGGCGTGTCG
>Fam_983_30_1  Nr. of seq. 1 Alignment length(with gaps) = 30 Alignment score = 0.000000
CATCGTCTCGTTCTCCATCGACCCATCGTC
>Fam_984_30_1  Nr. of seq. 1 Alignment length(with gaps) = 30 Alignment score = 0.000000
GGATTGATTAGGTGATCATGTTCTTTTATG
>Fam_985_30_1  Nr. of seq. 1 Alignment length(with gaps) = 30 Alignment score = 0.000000
AATAAATTTATTATNAATTGTTTTTATGTG
>Fam_986_30_1  Nr. of seq. 1 Alignment length(with gaps) = 30 Alignment score = 0.000000
ATAGTTAAAAACTAAATGAATACTAATTAA
>Fam_987_30_1  Nr. of seq. 1 Alignment length(with gaps) = 30 Alignment score = 0.000000
AAAGATAAAAAGAATTAAAATAATTAAAAG
>Fam_988_30_1  Nr. of seq. 1 Alignment length(with gaps) = 30 Alignment score = 0.000000
AACACCAACGGAAACGGCCACGACAACCCC
>Fam_989_30_1  Nr. of seq. 1 Alignment length(with gaps) = 30 Alignment score = 0.000000
AAATCGGCTCCATAAAGAGATGCACAAGAG
>Fam_990_30_1  Nr. of seq. 1 Alignment length(with gaps) = 30 Alignment score = 0.000000
ATTTATTTAAGCATTTTTTCACTTGATAAC
>Fam_991_30_1  Nr. of seq. 1 Alignment length(with gaps) = 30 Alignment score = 0.000000
GATTCAGGTTTTTTTTCAGTTTTGGTTTCT
>Fam_992_30_1  Nr. of seq. 1 Alignment length(with gaps) = 30 Alignment score = 0.000000
AGACTTACGGAGATGAACCGACTAGGGATC
>Fam_993_30_1  Nr. of seq. 1 Alignment length(with gaps) = 30 Alignment score = 0.000000
AGTCTAAGTAATAACAGTTAGAGCATTAAC
>Fam_994_30_1  Nr. of seq. 1 Alignment length(with gaps) = 30 Alignment score = 0.000000
TTTTTTAATAAATTATTACTCATCTAGTTC
>Fam_995_30_1  Nr. of seq. 1 Alignment length(with gaps) = 30 Alignment score = 0.000000
CTCTAAACAAAGTNTNTCTGAACAAAATAA
>Fam_996_30_1  Nr. of seq. 1 Alignment length(with gaps) = 30 Alignment score = 0.000000
TTAACACGTGATATAGAGATAATTAGGTAG
>Fam_997_30_1  Nr. of seq. 1 Alignment length(with gaps) = 30 Alignment score = 0.000000
AGCAGTGTCGTCAGCTGCAACATCTTCTGC
>Fam_998_30_1  Nr. of seq. 1 Alignment length(with gaps) = 30 Alignment score = 0.000000
TACACTTTCCTGACTCCACTCCAGGGTTGT
>Fam_999_30_1  Nr. of seq. 1 Alignment length(with gaps) = 30 Alignment score = 0.000000
```

ACAGGAAATTTAACTGATTATTTAAACTTC
>Fam_1000_30_1 Nr. of seq. 1 Alignment length(with gaps) = 30 Alignment score = 0.000000
TCTTAAGAACTATTTCCTTCTAAAGCTGTA
>Fam_1001_30_1 Nr. of seq. 1 Alignment length(with gaps) = 30 Alignment score = 0.000000
ATCTTGAAGGAGCTAACCTTNATGAAGCTC
>Fam_1002_30_1 Nr. of seq. 1 Alignment length(with gaps) = 30 Alignment score = 0.000000
CCTCAGATAAAGGCAAACACAGATGCCATA
>Fam_1003_30_1 Nr. of seq. 1 Alignment length(with gaps) = 30 Alignment score = 0.000000
GCCCTCGAGCGAGACATCGCCAAACTGGTC
>Fam_1004_30_1 Nr. of seq. 1 Alignment length(with gaps) = 30 Alignment score = 0.000000
CCCGGCGACTCGCCGTCGTCACCACTCTGG
>Fam_1005_30_1 Nr. of seq. 1 Alignment length(with gaps) = 30 Alignment score = 0.000000
GTGTCGTGGAAGTGGTGGTGTGGCGAGTGC
>Fam_1006_30_1 Nr. of seq. 1 Alignment length(with gaps) = 30 Alignment score = 0.000000
TCTTCGTATTCGTCATCCTCGTGCTCCTCA
>Fam_1007_29_1 Nr. of seq. 1 Alignment length(with gaps) = 29 Alignment score = 0.000000
GTCAGCGATCGAGAACCAGGACGTACCGC
>Fam_1008_29_1 Nr. of seq. 1 Alignment length(with gaps) = 29 Alignment score = 0.000000
GTTTTTTATTATTTAAATGGGAAATGTGA
>Fam_1009_29_1 Nr. of seq. 1 Alignment length(with gaps) = 29 Alignment score = 0.000000
TTTTTATACTAAACNCTAGTTAGTATANT
>Fam_1010_29_1 Nr. of seq. 1 Alignment length(with gaps) = 29 Alignment score = 0.000000
AAATTATTTTTTAAATAATTAATNTTAAT
>Fam_1011_29_1 Nr. of seq. 1 Alignment length(with gaps) = 29 Alignment score = 0.000000
TTATTTAAAACTAGTTAAACTTTTATAAC
>Fam_1012_29_1 Nr. of seq. 1 Alignment length(with gaps) = 29 Alignment score = 0.000000
TTTATACGGTACAATATACGGAATGATTA
>Fam_1013_29_1 Nr. of seq. 1 Alignment length(with gaps) = 29 Alignment score = 0.000000
CCTTTTCTTTAACTTGCTTTTTTAGCTGA
>Fam_1014_29_1 Nr. of seq. 1 Alignment length(with gaps) = 29 Alignment score = 0.000000
AGATTTTTTTAGGCAATTGTAATTAAACT
>Fam_1015_29_1 Nr. of seq. 1 Alignment length(with gaps) = 29 Alignment score = 0.000000
TTCAGGAGTTCTAAACTCTAAACCAGCCC
>Fam_1016_29_1 Nr. of seq. 1 Alignment length(with gaps) = 29 Alignment score = 0.000000
GAAAGAATCCCAATAATAAAAATTTACCA
>Fam_1017_29_1 Nr. of seq. 1 Alignment length(with gaps) = 29 Alignment score = 0.000000
CATGTTATTTTTCAGGAACTATGATTTTA
>Fam_1018_29_1 Nr. of seq. 1 Alignment length(with gaps) = 29 Alignment score = 0.000000

TCCAATGATTATTGCTTAGATAATTCAAA
>Fam_1019_29_1 Nr. of seq. 1 Alignment length(with gaps) = 29 Alignment score = 0.000000
GAATAAGTTGTACGAAAAACCGGAGAAAA
>Fam_1020_29_1 Nr. of seq. 1 Alignment length(with gaps) = 29 Alignment score = 0.000000
TGAGTTTACTATATTCCTCAATTAATTCG
>Fam_1021_29_1 Nr. of seq. 1 Alignment length(with gaps) = 29 Alignment score = 0.000000
GAAAGGAAAGTGAAAATTTAGAAAATTGA
>Fam_1022_29_1 Nr. of seq. 1 Alignment length(with gaps) = 29 Alignment score = 0.000000
CTGTCTTTCGTGTCGTCGTTCCCACAACG
>Fam_1023_28_1 Nr. of seq. 1 Alignment length(with gaps) = 28 Alignment score = 0.000000
CCAACCGCTAGTCTCACAACTGCTCTGA
>Fam_1024_28_1 Nr. of seq. 1 Alignment length(with gaps) = 28 Alignment score = 0.000000
ACGACCGAGGTGCGTCGGTTAAGGACAT
>Fam_1025_28_1 Nr. of seq. 1 Alignment length(with gaps) = 28 Alignment score = 0.000000
CTGCTCTCCGATAAGAGGCGACGTGTCT
>Fam_1026_28_1 Nr. of seq. 1 Alignment length(with gaps) = 28 Alignment score = 0.000000
TCACCTCCGCTTCTCGCAGTCACCTCGT
>Fam_1027_28_1 Nr. of seq. 1 Alignment length(with gaps) = 28 Alignment score = 0.000000
CGGTGGAGTGGGTAGCAGGCTGGTTTGG
>Fam_1028_28_1 Nr. of seq. 1 Alignment length(with gaps) = 28 Alignment score = 0.000000
TTATTTTAAAAGAATATTATTATATTGG
>Fam_1029_28_1 Nr. of seq. 1 Alignment length(with gaps) = 28 Alignment score = 0.000000
CAAGATTTAATTCGTGTTTGATACCGAC
>Fam_1030_28_1 Nr. of seq. 1 Alignment length(with gaps) = 28 Alignment score = 0.000000
CAGATTCAATTCTTTAGATCCAATCCTT
>Fam_1031_28_1 Nr. of seq. 1 Alignment length(with gaps) = 28 Alignment score = 0.000000
ATCAGTTAATCATAATTGGTTAATTGTT
>Fam_1032_28_1 Nr. of seq. 1 Alignment length(with gaps) = 28 Alignment score = 0.000000
GAAGTAATCGATATCTGAAGCGTTTCAA
>Fam_1033_28_1 Nr. of seq. 1 Alignment length(with gaps) = 28 Alignment score = 0.000000
GTTGCAGATATCTATTCTTCTATACGTA
>Fam_1034_28_1 Nr. of seq. 1 Alignment length(with gaps) = 28 Alignment score = 0.000000
TTTATTGCCATATTACATTTTGCCACTT
>Fam_1035_28_1 Nr. of seq. 1 Alignment length(with gaps) = 28 Alignment score = 0.000000
TTTCCTTAACCTATATTTACAGGCTTAT
>Fam_1036_28_1 Nr. of seq. 1 Alignment length(with gaps) = 28 Alignment score = 0.000000
GTGCAATACTAACTAGTGATATCCAGCA
>Fam_1037_28_1 Nr. of seq. 1 Alignment length(with gaps) = 28 Alignment score = 0.000000

TAATGGAATCTGGTTAATAGAGTCTAGT
>Fam_1038_28_1 Nr. of seq. 1 Alignment length(with gaps) = 28 Alignment score = 0.000000
TTTTATTTTGAGTCTTTTCTTTAGAATC
>Fam_1039_28_1 Nr. of seq. 1 Alignment length(with gaps) = 28 Alignment score = 0.000000
TATATTATCCAAATATCAGGTAATACTC
>Fam_1040_28_1 Nr. of seq. 1 Alignment length(with gaps) = 28 Alignment score = 0.000000
ATTATCAGTAACATGCTTAATATGCTTA
>Fam_1041_28_1 Nr. of seq. 1 Alignment length(with gaps) = 28 Alignment score = 0.000000
AATTTACTAATTTTAATTCAATAGTTTT
>Fam_1042_28_1 Nr. of seq. 1 Alignment length(with gaps) = 28 Alignment score = 0.000000
GCTGTTTATCTGGACAGCTCTTCCTCTG
>Fam_1043_28_1 Nr. of seq. 1 Alignment length(with gaps) = 28 Alignment score = 0.000000
CAGAGCAAGAAACTGAGAACAAAAAGTC
>Fam_1044_28_1 Nr. of seq. 1 Alignment length(with gaps) = 28 Alignment score = 0.000000
TTGATTACAGAATTATNAAATACAGAAC
>Fam_1045_28_1 Nr. of seq. 1 Alignment length(with gaps) = 28 Alignment score = 0.000000
AGTGGAGTGCGGGCGGTGGGATTGCAGG
>Fam_1046_27_1 Nr. of seq. 1 Alignment length(with gaps) = 27 Alignment score = 0.000000
GGAGTTGGGGTAACCGGTGGCTTGGTT
>Fam_1047_27_1 Nr. of seq. 1 Alignment length(with gaps) = 27 Alignment score = 0.000000
CCGCCGACGAGCCGTCCCATGCTTCGA
>Fam_1048_27_1 Nr. of seq. 1 Alignment length(with gaps) = 27 Alignment score = 0.000000
CTTCCCCACTCTTCACTTCTCCCGCCT
>Fam_1049_27_1 Nr. of seq. 1 Alignment length(with gaps) = 27 Alignment score = 0.000000
AGGCCGACAGGCGAGAGTGAAATACAC
>Fam_1050_27_1 Nr. of seq. 1 Alignment length(with gaps) = 27 Alignment score = 0.000000
TCACTGTCCGTTGGTTCTTCGGTCTCC
>Fam_1051_27_1 Nr. of seq. 1 Alignment length(with gaps) = 27 Alignment score = 0.000000
GAGAACGGTGAGAACGGTGAGAACGGC
>Fam_1052_27_1 Nr. of seq. 1 Alignment length(with gaps) = 27 Alignment score = 0.000000
CCATGGCGACCACGAACACGGACACGG
>Fam_1053_27_1 Nr. of seq. 1 Alignment length(with gaps) = 27 Alignment score = 0.000000
ATGCTGAATGGTCCCGCGACGAGTCAC
>Fam_1054_27_1 Nr. of seq. 1 Alignment length(with gaps) = 27 Alignment score = 0.000000
CAACCAGACGCCGCCCGGTCAAGCTGG
>Fam_1055_27_1 Nr. of seq. 1 Alignment length(with gaps) = 27 Alignment score = 0.000000
CAGTCTCGGCATCCAACTCATCGGACA
>Fam_1056_27_1 Nr. of seq. 1 Alignment length(with gaps) = 27 Alignment score = 0.000000

CGTAGGGACTGTCGGTNGNAATGCCCT
>Fam_1057_27_1 Nr. of seq. 1 Alignment length(with gaps) = 27 Alignment score = 0.000000
TCCCGATCGATGATTTCGCGTGTGGGC
>Fam_1058_27_1 Nr. of seq. 1 Alignment length(with gaps) = 27 Alignment score = 0.000000
TGGGACTGGGGAGACAGTGACAGCAGT
>Fam_1059_27_1 Nr. of seq. 1 Alignment length(with gaps) = 27 Alignment score = 0.000000
CTCCTCCCCATTCTCTTCCTCTCCATT
>Fam_1060_27_1 Nr. of seq. 1 Alignment length(with gaps) = 27 Alignment score = 0.000000
TGTTGTTGTGGCTGGTGTTGACGCAGT
>Fam_1061_27_1 Nr. of seq. 1 Alignment length(with gaps) = 27 Alignment score = 0.000000
GGCTGCTGGAAAGCCTGCTGCCCCAGN
>Fam_1062_27_1 Nr. of seq. 1 Alignment length(with gaps) = 27 Alignment score = 0.000000
ACAGGGGATACTGGAGATACCGGTGAC
>Fam_1063_27_1 Nr. of seq. 1 Alignment length(with gaps) = 27 Alignment score = 0.000000
GACACTTATATATTAACATCCAGAAAT
>Fam_1064_27_1 Nr. of seq. 1 Alignment length(with gaps) = 27 Alignment score = 0.000000
CCATATCTTCCTTTATAGAGATCTAAG
>Fam_1065_27_1 Nr. of seq. 1 Alignment length(with gaps) = 27 Alignment score = 0.000000
AGATACAGAATCACTATATCCCCCAGT
>Fam_1066_27_1 Nr. of seq. 1 Alignment length(with gaps) = 27 Alignment score = 0.000000
TCTGACAACAATACTGGAGATAACTCC
>Fam_1067_27_1 Nr. of seq. 1 Alignment length(with gaps) = 27 Alignment score = 0.000000
AATATTTACTTCATTGAACCTAGCTGC
>Fam_1068_27_1 Nr. of seq. 1 Alignment length(with gaps) = 27 Alignment score = 0.000000
AACAATAAGAAAACAAATCAACTCTAT
>Fam_1069_27_1 Nr. of seq. 1 Alignment length(with gaps) = 27 Alignment score = 0.000000
TTGAGCCGGAATCCGAGCTTCCATCAC
>Fam_1070_27_1 Nr. of seq. 1 Alignment length(with gaps) = 27 Alignment score = 0.000000
CAATCCTTTAACAAATCTTCCAATCAC
>Fam_1071_27_1 Nr. of seq. 1 Alignment length(with gaps) = 27 Alignment score = 0.000000
ACTTAATTCATTTTTGTTTTTAAACTA
>Fam_1072_27_1 Nr. of seq. 1 Alignment length(with gaps) = 27 Alignment score = 0.000000
GGATATACAGTATGATGTAAACGTGAT
>Fam_1073_27_1 Nr. of seq. 1 Alignment length(with gaps) = 27 Alignment score = 0.000000
TCGTCAGAGGAATTACTGTTTCCGCCC
>Fam_1074_27_1 Nr. of seq. 1 Alignment length(with gaps) = 27 Alignment score = 0.000000
CTGAGAGGATTGAAAAGTCTGGAAAAG
>Fam_1075_27_1 Nr. of seq. 1 Alignment length(with gaps) = 27 Alignment score = 0.000000

```
 TTTATCAAAAACTTTTTAGAAAAAAGT
>Fam_1076_27_1  Nr. of seq. 1 Alignment length(with gaps) = 27 Alignment score = 0.000000
 GATCCAGGGTCTAAACTTCCATTATCT
>Fam_1077_27_1  Nr. of seq. 1 Alignment length(with gaps) = 27 Alignment score = 0.000000
 TCTGGCCTATCAAACCAGTACTCTAAA
>Fam_1078_27_1  Nr. of seq. 1 Alignment length(with gaps) = 27 Alignment score = 0.000000
 AACAGATAGTCGGCATTATTTATCTAA
>Fam_1079_27_1  Nr. of seq. 1 Alignment length(with gaps) = 27 Alignment score = 0.000000
 ATTTAAACGAGGATCCAGTTGATGATG
>Fam_1080_27_1  Nr. of seq. 1 Alignment length(with gaps) = 27 Alignment score = 0.000000
 TTAATTCTTTTTAACCTGGGTTCTACC
>Fam_1081_27_1  Nr. of seq. 1 Alignment length(with gaps) = 27 Alignment score = 0.000000
 AAAAAGAGAAGGAAAAACAGGAAATNG
>Fam_1082_27_1  Nr. of seq. 1 Alignment length(with gaps) = 27 Alignment score = 0.000000
 CTCAATGCAACTGCCAACGCAACTGCG
>Fam_1083_27_1  Nr. of seq. 1 Alignment length(with gaps) = 27 Alignment score = 0.000000
 CGTCGTCGGAGTCCGAGTCAGTGTCGC
>Fam_1084_27_1  Nr. of seq. 1 Alignment length(with gaps) = 27 Alignment score = 0.000000
 ACGGTGACGACGGCGACGACGGCACCG
>Fam_1085_27_1  Nr. of seq. 1 Alignment length(with gaps) = 27 Alignment score = 0.000000
 GAAGATGGCTACTAAGGAAGACCTCCA
>Fam_1086_26_1  Nr. of seq. 1 Alignment length(with gaps) = 26 Alignment score = 0.000000
 TTCAGCTTTCAATTCCGTCTTTTCCG
>Fam_1087_26_1  Nr. of seq. 1 Alignment length(with gaps) = 26 Alignment score = 0.000000
 GTATGAATATGTGGTATGACTTTCAG
>Fam_1088_26_1  Nr. of seq. 1 Alignment length(with gaps) = 26 Alignment score = 0.000000
 ACCTGAAATCTGAATTGAAGGTTTAT
>Fam_1089_26_1  Nr. of seq. 1 Alignment length(with gaps) = 26 Alignment score = 0.000000
 TGATAACATGGATATTCTTAACATGT
>Fam_1090_26_1  Nr. of seq. 1 Alignment length(with gaps) = 26 Alignment score = 0.000000
 TTCATCTAATTTTGTACTATAAGTTC
>Fam_1091_26_1  Nr. of seq. 1 Alignment length(with gaps) = 26 Alignment score = 0.000000
 TTTTCACTTTGATGTTTCATTTTGAG
>Fam_1092_26_1  Nr. of seq. 1 Alignment length(with gaps) = 26 Alignment score = 0.000000
 TATCATTGAATTACAGATAATCATCT
>Fam_1093_26_1  Nr. of seq. 1 Alignment length(with gaps) = 26 Alignment score = 0.000000
 TCCACTAATCCACTAATAATACAGAT
>Fam_1094_26_1  Nr. of seq. 1 Alignment length(with gaps) = 26 Alignment score = 0.000000
```

```
 TTTTCAGTTTTCAGTCTATAATCATT
>Fam_1095_26_1  Nr. of seq. 1 Alignment length(with gaps) = 26 Alignment score = 0.000000
 ATCAAGATTTCGAAAAAGAGAGTCAG
>Fam_1096_26_1  Nr. of seq. 1 Alignment length(with gaps) = 26 Alignment score = 0.000000
 GAATTATTGACTGGAATTGTTGGCTA
>Fam_1097_26_1  Nr. of seq. 1 Alignment length(with gaps) = 26 Alignment score = 0.000000
 CAATAAAGAAAATCGATCAAGAAAAC
>Fam_1098_26_1  Nr. of seq. 1 Alignment length(with gaps) = 26 Alignment score = 0.000000
 TTTTACTGATTAATTCNTTGGATTGA
>Fam_1099_26_1  Nr. of seq. 1 Alignment length(with gaps) = 26 Alignment score = 0.000000
 ATATTCCAGCTTAAGTCTTAAAATCC
>Fam_1100_26_1  Nr. of seq. 1 Alignment length(with gaps) = 26 Alignment score = 0.000000
 GTTGGATTGTGGGGTAATATAGTCCG
>Fam_1101_26_1  Nr. of seq. 1 Alignment length(with gaps) = 26 Alignment score = 0.000000
 AAAATTCAAGATTCAGGGAATCATTT
>Fam_1102_26_1  Nr. of seq. 1 Alignment length(with gaps) = 26 Alignment score = 0.000000
 AGATATTATTGTTTATTTTGCTTGTT
>Fam_1103_26_1  Nr. of seq. 1 Alignment length(with gaps) = 26 Alignment score = 0.000000
 TTTACAGCTATCATTTCATATCTGTC
>Fam_1104_26_1  Nr. of seq. 1 Alignment length(with gaps) = 26 Alignment score = 0.000000
 GGAATTTCCATATCCTTTATTTACCC
>Fam_1105_26_1  Nr. of seq. 1 Alignment length(with gaps) = 26 Alignment score = 0.000000
 CCATTGATAACTCGTTATTAATTTAC
>Fam_1106_25_1  Nr. of seq. 1 Alignment length(with gaps) = 25 Alignment score = 0.000000
 CTATGAGACGCCAACGAACGAAGAA
>Fam_1107_25_1  Nr. of seq. 1 Alignment length(with gaps) = 25 Alignment score = 0.000000
 TTATAAATATGAATAGATTTAGTTT
>Fam_1108_25_1  Nr. of seq. 1 Alignment length(with gaps) = 25 Alignment score = 0.000000
 TTAAAGGTCCGATTGAACGGATACG
>Fam_1109_25_1  Nr. of seq. 1 Alignment length(with gaps) = 25 Alignment score = 0.000000
 ATAGCCATAATTCAGTGTTTATCAG
>Fam_1110_25_1  Nr. of seq. 1 Alignment length(with gaps) = 25 Alignment score = 0.000000
 TAAATTACTCATTCGTCATCTGCAG
>Fam_1111_25_1  Nr. of seq. 1 Alignment length(with gaps) = 25 Alignment score = 0.000000
 TTTAGCCTACTCAGCTTTATGAATC
>Fam_1112_25_1  Nr. of seq. 1 Alignment length(with gaps) = 25 Alignment score = 0.000000
 TAATATTTGACGATTTATCATTTGT
>Fam_1113_25_1  Nr. of seq. 1 Alignment length(with gaps) = 25 Alignment score = 0.000000
```

```
AAGTATAATATAAGTCTCTATATCA
>Fam_1114_25_1  Nr. of seq. 1 Alignment length(with gaps) = 25 Alignment score = 0.000000
GTAAGTGTAATTGTCTATATTATGC
>Fam_1115_25_1  Nr. of seq. 1 Alignment length(with gaps) = 25 Alignment score = 0.000000
ACTTTAATTTCATTTTTGCTAATCA
>Fam_1116_25_1  Nr. of seq. 1 Alignment length(with gaps) = 25 Alignment score = 0.000000
CTTTGGATTTCAGAAAGAATTAACA
>Fam_1117_25_1  Nr. of seq. 1 Alignment length(with gaps) = 25 Alignment score = 0.000000
TGTTTTTACTCCTGCTTCTTAATTA
>Fam_1118_25_1  Nr. of seq. 1 Alignment length(with gaps) = 25 Alignment score = 0.000000
AATTAGTAAGAAAATCTCATTCTGG
>Fam_1119_25_1  Nr. of seq. 1 Alignment length(with gaps) = 25 Alignment score = 0.000000
CTGTAAAATATAGGTGTAAAATGAA
>Fam_1120_25_1  Nr. of seq. 1 Alignment length(with gaps) = 25 Alignment score = 0.000000
GTGAACTATCTATCTTTAGATTAGC
>Fam_1121_25_1  Nr. of seq. 1 Alignment length(with gaps) = 25 Alignment score = 0.000000
TCATGTAAGTTGTCCCTCCAAAAAC
>Fam_1122_25_1  Nr. of seq. 1 Alignment length(with gaps) = 25 Alignment score = 0.000000
GTTTAGTAACATTGTTATTTTACAG
>Fam_1123_24_1  Nr. of seq. 1 Alignment length(with gaps) = 24 Alignment score = 0.000000
GAACGGATCGGGGGAACGGTAGGT
>Fam_1124_24_1  Nr. of seq. 1 Alignment length(with gaps) = 24 Alignment score = 0.000000
TCGGTTTCGTTCGCNGGCGGTTCT
>Fam_1125_24_1  Nr. of seq. 1 Alignment length(with gaps) = 24 Alignment score = 0.000000
CCGTCAGTCCGGACATCGTTCCCA
>Fam_1126_24_1  Nr. of seq. 1 Alignment length(with gaps) = 24 Alignment score = 0.000000
CGATTCCGGAACTGACGCAGAAGA
>Fam_1127_24_1  Nr. of seq. 1 Alignment length(with gaps) = 24 Alignment score = 0.000000
CGATAACTGACTGCTGTTCGAGTT
>Fam_1128_24_1  Nr. of seq. 1 Alignment length(with gaps) = 24 Alignment score = 0.000000
AACGAGACCAGTACTGACGATCTG
>Fam_1129_24_1  Nr. of seq. 1 Alignment length(with gaps) = 24 Alignment score = 0.000000
AACGAGACGGCCGAAGACGGCCTC
>Fam_1130_24_1  Nr. of seq. 1 Alignment length(with gaps) = 24 Alignment score = 0.000000
CTCGTCGGCGGTGGAGCCGTCTGT
>Fam_1131_24_1  Nr. of seq. 1 Alignment length(with gaps) = 24 Alignment score = 0.000000
GACGATGAGGGCGAAAGCGACGGT
>Fam_1132_24_1  Nr. of seq. 1 Alignment length(with gaps) = 24 Alignment score = 0.000000
```

```
 TTCCTGTGACTCCGGAGAATCCTG
>Fam_1133_24_1  Nr. of seq. 1 Alignment length(with gaps) = 24 Alignment score = 0.000000
 GGTATTGAGGAAGGTAGATTGGAA
>Fam_1134_24_1  Nr. of seq. 1 Alignment length(with gaps) = 24 Alignment score = 0.000000
 ATGCTCATGCTCATGCTCCTGATG
>Fam_1135_24_1  Nr. of seq. 1 Alignment length(with gaps) = 24 Alignment score = 0.000000
 TGATTCTAATCCAAAACTTGATGC
>Fam_1136_24_1  Nr. of seq. 1 Alignment length(with gaps) = 24 Alignment score = 0.000000
 ACTGCTTCTTCAGAAGATTCCTCA
>Fam_1137_24_1  Nr. of seq. 1 Alignment length(with gaps) = 24 Alignment score = 0.000000
 CACAGAAGACCAACCAGGAGAACA
>Fam_1138_24_1  Nr. of seq. 1 Alignment length(with gaps) = 24 Alignment score = 0.000000
 ACGGTGAACGTTACGCCGACTCCG
>Fam_1139_24_1  Nr. of seq. 1 Alignment length(with gaps) = 24 Alignment score = 0.000000
 AAGATCAATAAAATTAGCTTAAGA
>Fam_1140_24_1  Nr. of seq. 1 Alignment length(with gaps) = 24 Alignment score = 0.000000
 ATATCGAAGTGGATCTGAAGGAGC
>Fam_1141_24_1  Nr. of seq. 1 Alignment length(with gaps) = 24 Alignment score = 0.000000
 TCAGTTTTTCTGTATCTCTGTTTC
>Fam_1142_24_1  Nr. of seq. 1 Alignment length(with gaps) = 24 Alignment score = 0.000000
 CCAGGTAGTGGGGGATGGGGTACA
>Fam_1143_24_1  Nr. of seq. 1 Alignment length(with gaps) = 24 Alignment score = 0.000000
 ACGGTTAATCACAGATATAGAGCC
>Fam_1144_24_1  Nr. of seq. 1 Alignment length(with gaps) = 24 Alignment score = 0.000000
 ACTAGTAAATGAAGTATATTTTTC
>Fam_1145_24_1  Nr. of seq. 1 Alignment length(with gaps) = 24 Alignment score = 0.000000
 TTTCTTCTTTAAGGGAATTCTGAT
>Fam_1146_24_1  Nr. of seq. 1 Alignment length(with gaps) = 24 Alignment score = 0.000000
 TAATATTGAAATTAAACTGTAGAN
>Fam_1147_24_1  Nr. of seq. 1 Alignment length(with gaps) = 24 Alignment score = 0.000000
 GCATATGAAGCAATTACAATACAG
>Fam_1148_24_1  Nr. of seq. 1 Alignment length(with gaps) = 24 Alignment score = 0.000000
 TTAATCTCAATTGTAATACAGTGT
>Fam_1149_24_1  Nr. of seq. 1 Alignment length(with gaps) = 24 Alignment score = 0.000000
 GATAATCATGTAAGGAGAATATCA
>Fam_1150_24_1  Nr. of seq. 1 Alignment length(with gaps) = 24 Alignment score = 0.000000
 CAGTTAAGGGTACAACTTAACAAC
>Fam_1151_24_1  Nr. of seq. 1 Alignment length(with gaps) = 24 Alignment score = 0.000000
```

AAAGATTCCAAAAAGTATGGAGAG
>Fam_1152_24_1 Nr. of seq. 1 Alignment length(with gaps) = 24 Alignment score = 0.000000
TTCTTGAGATTTAAAAAGAACGTT
>Fam_1153_24_1 Nr. of seq. 1 Alignment length(with gaps) = 24 Alignment score = 0.000000
ATCTGAACAAAATATGAAGATATT
>Fam_1154_24_1 Nr. of seq. 1 Alignment length(with gaps) = 24 Alignment score = 0.000000
ACTTTAGTTAGATCTCAGTTTTTT
>Fam_1155_24_1 Nr. of seq. 1 Alignment length(with gaps) = 24 Alignment score = 0.000000
CAGATACAGGATGAGCCTGCCCAT
>Fam_1156_24_1 Nr. of seq. 1 Alignment length(with gaps) = 24 Alignment score = 0.000000
ATTCAGGTCTACTTTATGATTCGG
>Fam_1157_24_1 Nr. of seq. 1 Alignment length(with gaps) = 24 Alignment score = 0.000000
TTTATTCCTTATCTAAAAGNATCT
>Fam_1158_24_1 Nr. of seq. 1 Alignment length(with gaps) = 24 Alignment score = 0.000000
ATTGGAAAGTTAAGTGATTACTTG
>Fam_1159_24_1 Nr. of seq. 1 Alignment length(with gaps) = 24 Alignment score = 0.000000
AATGCAATTAGTAGTACAATCAGC
>Fam_1160_24_1 Nr. of seq. 1 Alignment length(with gaps) = 24 Alignment score = 0.000000
TTGATTCGTTTCCTTCACTCTTTG
>Fam_1161_24_1 Nr. of seq. 1 Alignment length(with gaps) = 24 Alignment score = 0.000000
GTGTATGAGATAAGTTTGATTCTG
>Fam_1162_24_1 Nr. of seq. 1 Alignment length(with gaps) = 24 Alignment score = 0.000000
AAGAATGGATAATTACGATCATTC
>Fam_1163_24_1 Nr. of seq. 1 Alignment length(with gaps) = 24 Alignment score = 0.000000
GGATTAGAAACAGGATAAGTAAAA
>Fam_1164_24_1 Nr. of seq. 1 Alignment length(with gaps) = 24 Alignment score = 0.000000
TTTCATTTCTTGGCTCAGCAAGAN
>Fam_1165_24_1 Nr. of seq. 1 Alignment length(with gaps) = 24 Alignment score = 0.000000
CGTCGTGGTGGCCCTCGTGATGAT
>Fam_1166_24_1 Nr. of seq. 1 Alignment length(with gaps) = 24 Alignment score = 0.000000
GGCGAGACGGCTGCGGACGACAGC
>Fam_1167_24_1 Nr. of seq. 1 Alignment length(with gaps) = 24 Alignment score = 0.000000
CAGCCACAGTCGCAGTCGCAATCG
>Fam_1168_24_1 Nr. of seq. 1 Alignment length(with gaps) = 24 Alignment score = 0.000000
CGTCTGTGTGTGCGTGGTGGTCGT
>Fam_1169_24_1 Nr. of seq. 1 Alignment length(with gaps) = 24 Alignment score = 0.000000
GTGAGCGGGACGACGTTCCGCTAG
>Fam_1170_24_1 Nr. of seq. 1 Alignment length(with gaps) = 24 Alignment score = 0.000000

GGTAATGTAACTGTGCCACCGGGA
>Fam_1171_23_1 Nr. of seq. 1 Alignment length(with gaps) = 23 Alignment score = 0.000000
CGGTCAGGTGCGGGTGCGTCTGG
>Fam_1172_23_1 Nr. of seq. 1 Alignment length(with gaps) = 23 Alignment score = 0.000000
CATCTATCTTCGTACGACCCAAC
>Fam_1173_23_1 Nr. of seq. 1 Alignment length(with gaps) = 23 Alignment score = 0.000000
AGCCAGATGTTTATAGCTAACCC
>Fam_1174_23_1 Nr. of seq. 1 Alignment length(with gaps) = 23 Alignment score = 0.000000
TATAAAATAAATANGATGAAAAG
>Fam_1175_23_1 Nr. of seq. 1 Alignment length(with gaps) = 23 Alignment score = 0.000000
TATTTATATACTTTTTATTTTAG
>Fam_1176_23_1 Nr. of seq. 1 Alignment length(with gaps) = 23 Alignment score = 0.000000
GAGCCTGCACTCAGGCTATATAA
>Fam_1177_23_1 Nr. of seq. 1 Alignment length(with gaps) = 23 Alignment score = 0.000000
ATTTGAATTACAAATCTGAGTTT
>Fam_1178_23_1 Nr. of seq. 1 Alignment length(with gaps) = 23 Alignment score = 0.000000
AAGTAAGGTTGAAAGGTTTTGGA
>Fam_1179_23_1 Nr. of seq. 1 Alignment length(with gaps) = 23 Alignment score = 0.000000
AATCCTTTACTTACTCTTACTTT
>Fam_1180_23_1 Nr. of seq. 1 Alignment length(with gaps) = 23 Alignment score = 0.000000
TTTGACTTAATGGAAAACACATA
>Fam_1181_23_1 Nr. of seq. 1 Alignment length(with gaps) = 23 Alignment score = 0.000000
ATTTTGAGTTCAGCTTTAGGTTT
>Fam_1182_23_1 Nr. of seq. 1 Alignment length(with gaps) = 23 Alignment score = 0.000000
TGGGTAATGTTTGTTTTTTAAGG
>Fam_1183_23_1 Nr. of seq. 1 Alignment length(with gaps) = 23 Alignment score = 0.000000
TTTCTGTTCAGCCTTGCTATATT
>Fam_1184_23_1 Nr. of seq. 1 Alignment length(with gaps) = 23 Alignment score = 0.000000
TGAAGATTATTACTGTCATTTGT
>Fam_1185_23_1 Nr. of seq. 1 Alignment length(with gaps) = 23 Alignment score = 0.000000
TGAAAGAGGTAGAGAGAAGATTG
>Fam_1186_23_1 Nr. of seq. 1 Alignment length(with gaps) = 23 Alignment score = 0.000000
ATAATTTCATGTATCACAACATC
>Fam_1187_23_1 Nr. of seq. 1 Alignment length(with gaps) = 23 Alignment score = 0.000000
ATAACGAAATTTCTCGAGATTCA
>Fam_1188_23_1 Nr. of seq. 1 Alignment length(with gaps) = 23 Alignment score = 0.000000
GTTACTTTTTGCAAATCAGAGAG
>Fam_1189_23_1 Nr. of seq. 1 Alignment length(with gaps) = 23 Alignment score = 0.000000

```
 GATTAATTTGTCTGACTGATTTT
>Fam_1190_23_1  Nr. of seq. 1 Alignment length(with gaps) = 23 Alignment score = 0.000000
 CTTTCTGCAGGTTTTAATTCAAT
>Fam_1191_23_1  Nr. of seq. 1 Alignment length(with gaps) = 23 Alignment score = 0.000000
 ATATTGAGATCAATCCATAACTA
>Fam_1192_23_1  Nr. of seq. 1 Alignment length(with gaps) = 23 Alignment score = 0.000000
 TTTCTTGCCTGGTTTTTAGTGGT
>Fam_1193_23_1  Nr. of seq. 1 Alignment length(with gaps) = 23 Alignment score = 0.000000
 TCCTTAGTTATGAATCATTATAA
>Fam_1194_23_1  Nr. of seq. 1 Alignment length(with gaps) = 23 Alignment score = 0.000000
 TCGCGTCCAGTCGTTCGTTTCTG
>Fam_1195_22_1  Nr. of seq. 1 Alignment length(with gaps) = 22 Alignment score = 0.000000
 GCGGTGGACGGAGATCGTGAAG
>Fam_1196_22_1  Nr. of seq. 1 Alignment length(with gaps) = 22 Alignment score = 0.000000
 TTGTTCGGCGGTTGTTGGCCGG
>Fam_1197_22_1  Nr. of seq. 1 Alignment length(with gaps) = 22 Alignment score = 0.000000
 AGTGTGTTACGAGTATGCCACG
>Fam_1198_22_1  Nr. of seq. 1 Alignment length(with gaps) = 22 Alignment score = 0.000000
 TTATTATTTTATAGTATCAGTC
>Fam_1199_22_1  Nr. of seq. 1 Alignment length(with gaps) = 22 Alignment score = 0.000000
 TCAGTAAGGTGGATGAGGTGTG
>Fam_1200_22_1  Nr. of seq. 1 Alignment length(with gaps) = 22 Alignment score = 0.000000
 GGGGTGGATCAGTAAGGTGCTT
>Fam_1201_22_1  Nr. of seq. 1 Alignment length(with gaps) = 22 Alignment score = 0.000000
 TCCAATTGATTTTCGATTAGTT
>Fam_1202_22_1  Nr. of seq. 1 Alignment length(with gaps) = 22 Alignment score = 0.000000
 AGGTTAATTCAAGCCTATTCAA
>Fam_1203_22_1  Nr. of seq. 1 Alignment length(with gaps) = 22 Alignment score = 0.000000
 TAATTTTGATCTATAGAACGTT
>Fam_1204_22_1  Nr. of seq. 1 Alignment length(with gaps) = 22 Alignment score = 0.000000
 AACCTGAAAATGTGTACATTCA
>Fam_1205_22_1  Nr. of seq. 1 Alignment length(with gaps) = 22 Alignment score = 0.000000
 GGGATAGTTGAACTTAACTTTT
>Fam_1206_22_1  Nr. of seq. 1 Alignment length(with gaps) = 22 Alignment score = 0.000000
 TAAAATATCAAATAACATAGTC
>Fam_1207_22_1  Nr. of seq. 1 Alignment length(with gaps) = 22 Alignment score = 0.000000
 AGTTAATAACGAGAAGTGGCAG
>Fam_1208_22_1  Nr. of seq. 1 Alignment length(with gaps) = 22 Alignment score = 0.000000
```

CAGGAATCTCAATTAACCTAAC
>Fam_1209_22_1 Nr. of seq. 1 Alignment length(with gaps) = 22 Alignment score = 0.000000
TTAGCTTCAGGAGAGTTTAATG
>Fam_1210_22_1 Nr. of seq. 1 Alignment length(with gaps) = 22 Alignment score = 0.000000
AAGTAAAAGTGAAAGTTGAAGG
>Fam_1211_22_1 Nr. of seq. 1 Alignment length(with gaps) = 22 Alignment score = 0.000000
CTCTTTTAAAACCTGTACCTTT
>Fam_1212_22_1 Nr. of seq. 1 Alignment length(with gaps) = 22 Alignment score = 0.000000
TTTTAGCATTACTGTATTGGAG
>Fam_1213_22_1 Nr. of seq. 1 Alignment length(with gaps) = 22 Alignment score = 0.000000
TTTCTAAATAACTTTTATCATA
>Fam_1214_22_1 Nr. of seq. 1 Alignment length(with gaps) = 22 Alignment score = 0.000000
AATTCCATAGTATTCAACCAGA
>Fam_1215_22_1 Nr. of seq. 1 Alignment length(with gaps) = 22 Alignment score = 0.000000
TACCTTATTCAAAGCAAAACCT
>Fam_1216_22_1 Nr. of seq. 1 Alignment length(with gaps) = 22 Alignment score = 0.000000
TTGATTTATTTAGAATACATAC
>Fam_1217_22_1 Nr. of seq. 1 Alignment length(with gaps) = 22 Alignment score = 0.000000
CTTTTTACTGGTATACTGCTTA
>Fam_1218_22_1 Nr. of seq. 1 Alignment length(with gaps) = 22 Alignment score = 0.000000
TTTTTCCTGAATTGTGTTATTG
>Fam_1219_22_1 Nr. of seq. 1 Alignment length(with gaps) = 22 Alignment score = 0.000000
TATATTTACTGAGTTTAGAGGA
>Fam_1220_22_1 Nr. of seq. 1 Alignment length(with gaps) = 22 Alignment score = 0.000000
AAAATAACTGCTGTTTGAGGAG
>Fam_1221_22_1 Nr. of seq. 1 Alignment length(with gaps) = 22 Alignment score = 0.000000
AGTAGAAGGAGAAGGAAAACTT
>Fam_1222_22_1 Nr. of seq. 1 Alignment length(with gaps) = 22 Alignment score = 0.000000
TCACAAAGAAATCCGAGAATAC
>Fam_1223_22_1 Nr. of seq. 1 Alignment length(with gaps) = 22 Alignment score = 0.000000
TAATTAAAAATTAANTAAAAGC
>Fam_1224_22_1 Nr. of seq. 1 Alignment length(with gaps) = 22 Alignment score = 0.000000
AATACATGGAGCACAACCGGAA
>Fam_1225_22_1 Nr. of seq. 1 Alignment length(with gaps) = 22 Alignment score = 0.000000
TTTCAGGTTAATACTCTGATTA
>Fam_1226_22_1 Nr. of seq. 1 Alignment length(with gaps) = 22 Alignment score = 0.000000
AAAAGTCTTCATGTCCGGTAAC
>Fam_1227_22_1 Nr. of seq. 1 Alignment length(with gaps) = 22 Alignment score = 0.000000

```
 ACAATCCAGGATGATTTGTTGA
>Fam_1228_22_1  Nr. of seq. 1 Alignment length(with gaps) = 22 Alignment score = 0.000000
 AGCTGGAAGAAAATTACAAAAG
>Fam_1229_22_1  Nr. of seq. 1 Alignment length(with gaps) = 22 Alignment score = 0.000000
 TAAAGGAAAGCATCTTCACTAA
>Fam_1230_22_1  Nr. of seq. 1 Alignment length(with gaps) = 22 Alignment score = 0.000000
 TTCAATTATTTTACTGGTAGAA
>Fam_1231_22_1  Nr. of seq. 1 Alignment length(with gaps) = 22 Alignment score = 0.000000
 CACGTAAAAAGTAATAGAATTT
>Fam_1232_22_1  Nr. of seq. 1 Alignment length(with gaps) = 22 Alignment score = 0.000000
 TCAAACCAAATCAAAAACACCT
>Fam_1233_22_1  Nr. of seq. 1 Alignment length(with gaps) = 22 Alignment score = 0.000000
 TCTAACCTTTTTTAAGGTTTTC
>Fam_1234_22_1  Nr. of seq. 1 Alignment length(with gaps) = 22 Alignment score = 0.000000
 GTTGTTTAGAAATACTTGCTTA
>Fam_1235_22_1  Nr. of seq. 1 Alignment length(with gaps) = 22 Alignment score = 0.000000
 TATAATTAACTAAAGTATAACA
>Fam_1236_22_1  Nr. of seq. 1 Alignment length(with gaps) = 22 Alignment score = 0.000000
 ATAACTTATAATGTTAAAACAA
>Fam_1237_22_1  Nr. of seq. 1 Alignment length(with gaps) = 22 Alignment score = 0.000000
 ATTAACAATTGAATACAGAGGG
>Fam_1238_22_1  Nr. of seq. 1 Alignment length(with gaps) = 22 Alignment score = 0.000000
 TCGCTGCGGTAACGCCACTGTC
>Fam_1239_22_1  Nr. of seq. 1 Alignment length(with gaps) = 22 Alignment score = 0.000000
 GCGTTTCGAGGCGTAACTGAAG
>Fam_1240_21_1  Nr. of seq. 1 Alignment length(with gaps) = 21 Alignment score = 0.000000
 ACAACCTGTGGAACGCGACTA
>Fam_1241_21_1  Nr. of seq. 1 Alignment length(with gaps) = 21 Alignment score = 0.000000
 GATGAGACTGTGGAACTTAAG
>Fam_1242_21_1  Nr. of seq. 1 Alignment length(with gaps) = 21 Alignment score = 0.000000
 CGATCGTCGTCGATGTCGGAC
>Fam_1243_21_1  Nr. of seq. 1 Alignment length(with gaps) = 21 Alignment score = 0.000000
 CGACGCCGAGGGGGGTATGGA
>Fam_1244_21_1  Nr. of seq. 1 Alignment length(with gaps) = 21 Alignment score = 0.000000
 GTTGGTTGTAAATGCCCAAGA
>Fam_1245_21_1  Nr. of seq. 1 Alignment length(with gaps) = 21 Alignment score = 0.000000
 AGAAGCACCAGATTNCACCTT
>Fam_1246_21_1  Nr. of seq. 1 Alignment length(with gaps) = 21 Alignment score = 0.000000
```

GGTTCTGTTCAATTGGCTAAC
>Fam_1247_21_1 Nr. of seq. 1 Alignment length(with gaps) = 21 Alignment score = 0.000000
CCATTACCACCAGGANNAGTA
>Fam_1248_21_1 Nr. of seq. 1 Alignment length(with gaps) = 21 Alignment score = 0.000000
GTTTAAACAAGAATATCTAAT
>Fam_1249_21_1 Nr. of seq. 1 Alignment length(with gaps) = 21 Alignment score = 0.000000
CCACTGTCCTCCACCACCCAT
>Fam_1250_21_1 Nr. of seq. 1 Alignment length(with gaps) = 21 Alignment score = 0.000000
TCATGCCATAAAATAACATGG
>Fam_1251_21_1 Nr. of seq. 1 Alignment length(with gaps) = 21 Alignment score = 0.000000
AGCGCTCCGCGCGGTTACTAG
>Fam_1252_21_1 Nr. of seq. 1 Alignment length(with gaps) = 21 Alignment score = 0.000000
TTGTTTTCTGATATGGGAGAC
>Fam_1253_21_1 Nr. of seq. 1 Alignment length(with gaps) = 21 Alignment score = 0.000000
TTTATTATTGTGTCTTGTCCC
>Fam_1254_21_1 Nr. of seq. 1 Alignment length(with gaps) = 21 Alignment score = 0.000000
ATCCTATAAACAATACAGTAG
>Fam_1255_21_1 Nr. of seq. 1 Alignment length(with gaps) = 21 Alignment score = 0.000000
TCCTGTTTATGTAATCTTAAT
>Fam_1256_21_1 Nr. of seq. 1 Alignment length(with gaps) = 21 Alignment score = 0.000000
TCCGTTTGGTTATCCAGAGAT
>Fam_1257_21_1 Nr. of seq. 1 Alignment length(with gaps) = 21 Alignment score = 0.000000
ATTTTCTCTTATCGTTATCCC
>Fam_1258_21_1 Nr. of seq. 1 Alignment length(with gaps) = 21 Alignment score = 0.000000
TTCAGTAGAGAACCCTGTTAG
>Fam_1259_21_1 Nr. of seq. 1 Alignment length(with gaps) = 21 Alignment score = 0.000000
TTTCCTTTTTACAACAGAATG
>Fam_1260_21_1 Nr. of seq. 1 Alignment length(with gaps) = 21 Alignment score = 0.000000
TCTGCATTTTTAACTCTCTAC
>Fam_1261_21_1 Nr. of seq. 1 Alignment length(with gaps) = 21 Alignment score = 0.000000
AATTATGTTTCTTATTGAGTA
>Fam_1262_21_1 Nr. of seq. 1 Alignment length(with gaps) = 21 Alignment score = 0.000000
CTGTCATTTCTAGTGTTTCAC
>Fam_1263_21_1 Nr. of seq. 1 Alignment length(with gaps) = 21 Alignment score = 0.000000
TAAGGTATAAGGAAGTTGGGT
>Fam_1264_21_1 Nr. of seq. 1 Alignment length(with gaps) = 21 Alignment score = 0.000000
TAAAAAATGGCAAAATACCTA
>Fam_1265_21_1 Nr. of seq. 1 Alignment length(with gaps) = 21 Alignment score = 0.000000

ATAAATCATTGATCTGTCCTT
>Fam_1266_21_1 Nr. of seq. 1 Alignment length(with gaps) = 21 Alignment score = 0.000000
GAGGTAGTAGAACCCACTGAA
>Fam_1267_21_1 Nr. of seq. 1 Alignment length(with gaps) = 21 Alignment score = 0.000000
CGTCTTCTTCGACAACATCAG
>Fam_1268_21_1 Nr. of seq. 1 Alignment length(with gaps) = 21 Alignment score = 0.000000
CCGGAAAAAAACTTTAATGAG
>Fam_1269_21_1 Nr. of seq. 1 Alignment length(with gaps) = 21 Alignment score = 0.000000
AACAGAGGGTAGAGCCTGTGA
>Fam_1270_21_1 Nr. of seq. 1 Alignment length(with gaps) = 21 Alignment score = 0.000000
GAATTTCAGGATTATGGCTGA
>Fam_1271_21_1 Nr. of seq. 1 Alignment length(with gaps) = 21 Alignment score = 0.000000
TGAGGAATGAAGAATAAAACA
>Fam_1272_21_1 Nr. of seq. 1 Alignment length(with gaps) = 21 Alignment score = 0.000000
GATAATTGCTGGTATCTTCAA
>Fam_1273_21_1 Nr. of seq. 1 Alignment length(with gaps) = 21 Alignment score = 0.000000
AATTTCAAATTAAAACAGGCA
>Fam_1274_21_1 Nr. of seq. 1 Alignment length(with gaps) = 21 Alignment score = 0.000000
AATGCATAATCGGTTAGTTTG
>Fam_1275_21_1 Nr. of seq. 1 Alignment length(with gaps) = 21 Alignment score = 0.000000
ATAATATACTGAAATAATTCT
>Fam_1276_21_1 Nr. of seq. 1 Alignment length(with gaps) = 21 Alignment score = 0.000000
TTAATTTTAANTACATAAGTC
>Fam_1277_21_1 Nr. of seq. 1 Alignment length(with gaps) = 21 Alignment score = 0.000000
ATTTTGCATTCCAAATTTCAC
>Fam_1278_21_1 Nr. of seq. 1 Alignment length(with gaps) = 21 Alignment score = 0.000000
GGATGATAACAAAACGATTTG
>Fam_1279_21_1 Nr. of seq. 1 Alignment length(with gaps) = 21 Alignment score = 0.000000
TTGCAGTTGAATAATAGACTA
>Fam_1280_21_1 Nr. of seq. 1 Alignment length(with gaps) = 21 Alignment score = 0.000000
CCCTTCATCTCTTTCATATCA
>Fam_1281_21_1 Nr. of seq. 1 Alignment length(with gaps) = 21 Alignment score = 0.000000
GTGCACACTCCACTGTGAGCC
>Fam_1282_21_1 Nr. of seq. 1 Alignment length(with gaps) = 21 Alignment score = 0.000000
GACGACGATGACGACCCAGCT
>Fam_1283_21_1 Nr. of seq. 1 Alignment length(with gaps) = 21 Alignment score = 0.000000
CTGACGACGCTGGAAACGGTG
>Fam_1284_21_1 Nr. of seq. 1 Alignment length(with gaps) = 21 Alignment score = 0.000000

TTGAAGCGTTTATGCTTATCA
>Fam_1285_20_1 Nr. of seq. 1 Alignment length(with gaps) = 20 Alignment score = 0.000000
AAATATTTTAGNCCTATTTT
>Fam_1286_20_1 Nr. of seq. 1 Alignment length(with gaps) = 20 Alignment score = 0.000000
TTCAAACTGATAGGTATCTG
>Fam_1287_20_1 Nr. of seq. 1 Alignment length(with gaps) = 20 Alignment score = 0.000000
ACTCAAGTGATTTTTCTCTT
>Fam_1288_20_1 Nr. of seq. 1 Alignment length(with gaps) = 20 Alignment score = 0.000000
TATTACCTGAGAGAGGTGTG
>Fam_1289_20_1 Nr. of seq. 1 Alignment length(with gaps) = 20 Alignment score = 0.000000
TAGTTGTTAATAGCTATCAG
>Fam_1290_20_1 Nr. of seq. 1 Alignment length(with gaps) = 20 Alignment score = 0.000000
AGTTACAGTAAAACACATTT
>Fam_1291_20_1 Nr. of seq. 1 Alignment length(with gaps) = 20 Alignment score = 0.000000
AACAGTTTATCTCATATTTC
>Fam_1292_20_1 Nr. of seq. 1 Alignment length(with gaps) = 20 Alignment score = 0.000000
CCTTTAAGTCACTATTTTCA
>Fam_1293_20_1 Nr. of seq. 1 Alignment length(with gaps) = 20 Alignment score = 0.000000
CCTTTAAACAATCCTATCAT
>Fam_1294_20_1 Nr. of seq. 1 Alignment length(with gaps) = 20 Alignment score = 0.000000
ATTTTGATAGACACTACATG
>Fam_1295_20_1 Nr. of seq. 1 Alignment length(with gaps) = 20 Alignment score = 0.000000
GTTTACTTTCACTCACTTTT
>Fam_1296_20_1 Nr. of seq. 1 Alignment length(with gaps) = 20 Alignment score = 0.000000
TTATTTACTGCTTTTTACTC
>Fam_1297_20_1 Nr. of seq. 1 Alignment length(with gaps) = 20 Alignment score = 0.000000
AATAATCAAAAGGTAAGCTT
>Fam_1298_20_1 Nr. of seq. 1 Alignment length(with gaps) = 20 Alignment score = 0.000000
TAATCTCCGATAATCTCGGG
>Fam_1299_20_1 Nr. of seq. 1 Alignment length(with gaps) = 20 Alignment score = 0.000000
TACTTTGGTATCAGACATTT
>Fam_1300_20_1 Nr. of seq. 1 Alignment length(with gaps) = 20 Alignment score = 0.000000
AAAAATTAAAGACAGANCAG
>Fam_1301_20_1 Nr. of seq. 1 Alignment length(with gaps) = 20 Alignment score = 0.000000
TTTCTTATTTTTTTATGCAG
>Fam_1302_20_1 Nr. of seq. 1 Alignment length(with gaps) = 20 Alignment score = 0.000000
CGATTTTTCCTTTTCCCATA
>Fam_1303_20_1 Nr. of seq. 1 Alignment length(with gaps) = 20 Alignment score = 0.000000

```
 ACCTCACCATTTTGACGTTC
>Fam_1304_20_1  Nr. of seq. 1 Alignment length(with gaps) = 20 Alignment score = 0.000000
 ATTTCTAACTTCTAGGGACA
>Fam_1305_20_1  Nr. of seq. 1 Alignment length(with gaps) = 20 Alignment score = 0.000000
 ACTCGACATTACAACCATTT
>Fam_1306_20_1  Nr. of seq. 1 Alignment length(with gaps) = 20 Alignment score = 0.000000
 CGAAGCGAGTGCGTCCTGAA
>Fam_1307_20_1  Nr. of seq. 1 Alignment length(with gaps) = 20 Alignment score = 0.000000
 TTCATTTCGANNACACCCCC
>Fam_1308_19_1  Nr. of seq. 1 Alignment length(with gaps) = 19 Alignment score = 0.000000
 GTAGAGAAGGGTGGGTAGA
>Fam_1309_19_1  Nr. of seq. 1 Alignment length(with gaps) = 19 Alignment score = 0.000000
 CGACGACCCGCCACCGGTC
>Fam_1310_19_1  Nr. of seq. 1 Alignment length(with gaps) = 19 Alignment score = 0.000000
 CTGATTCGTGTGCTCGAGA
>Fam_1311_19_1  Nr. of seq. 1 Alignment length(with gaps) = 19 Alignment score = 0.000000
 ACAATTTTACGAAGCCACG
>Fam_1312_19_1  Nr. of seq. 1 Alignment length(with gaps) = 19 Alignment score = 0.000000
 ATCCTAATTTTTCATGAGA
>Fam_1313_19_1  Nr. of seq. 1 Alignment length(with gaps) = 19 Alignment score = 0.000000
 AAGAAAGGGATTATGGAGT
>Fam_1314_19_1  Nr. of seq. 1 Alignment length(with gaps) = 19 Alignment score = 0.000000
 TAGATTTTAGGGCTTGTTC
>Fam_1315_19_1  Nr. of seq. 1 Alignment length(with gaps) = 19 Alignment score = 0.000000
 TTAATTGAAGATATTAAAG
>Fam_1316_19_1  Nr. of seq. 1 Alignment length(with gaps) = 19 Alignment score = 0.000000
 TAAGTCCTATCAATCTGCA
>Fam_1317_19_1  Nr. of seq. 1 Alignment length(with gaps) = 19 Alignment score = 0.000000
 CTATTTCAAATGACATTCT
>Fam_1318_19_1  Nr. of seq. 1 Alignment length(with gaps) = 19 Alignment score = 0.000000
 ATGTCAAACTGAGATAAGG
>Fam_1319_19_1  Nr. of seq. 1 Alignment length(with gaps) = 19 Alignment score = 0.000000
 ATTCTTAAGATCCTAATCA
>Fam_1320_19_1  Nr. of seq. 1 Alignment length(with gaps) = 19 Alignment score = 0.000000
 CCAGTTAAAGTACAGTAAC
>Fam_1321_19_1  Nr. of seq. 1 Alignment length(with gaps) = 19 Alignment score = 0.000000
 CACTACTCAATGTTAAAAT
>Fam_1322_19_1  Nr. of seq. 1 Alignment length(with gaps) = 19 Alignment score = 0.000000
```

GTTACCTTAATACAGATGA
>Fam_1323_19_1 Nr. of seq. 1 Alignment length(with gaps) = 19 Alignment score = 0.000000
TAAGGCTAACTGCTGAAAG
>Fam_1324_19_1 Nr. of seq. 1 Alignment length(with gaps) = 19 Alignment score = 0.000000
TTTGTTTGAAATCATCTGT
>Fam_1325_19_1 Nr. of seq. 1 Alignment length(with gaps) = 19 Alignment score = 0.000000
CTGATAGCAATTCTTTGAT
>Fam_1326_19_1 Nr. of seq. 1 Alignment length(with gaps) = 19 Alignment score = 0.000000
TTCAGTTTCAACCTCGACN
>Fam_1327_19_1 Nr. of seq. 1 Alignment length(with gaps) = 19 Alignment score = 0.000000
AAATTAAATCAGGGGATAC
>Fam_1328_19_1 Nr. of seq. 1 Alignment length(with gaps) = 19 Alignment score = 0.000000
ATTCTGTATTCTGGAGGAG
>Fam_1329_19_1 Nr. of seq. 1 Alignment length(with gaps) = 19 Alignment score = 0.000000
CTCATATTCAATAATAAAG
>Fam_1330_19_1 Nr. of seq. 1 Alignment length(with gaps) = 19 Alignment score = 0.000000
TCCTTCTTTTATCACTTCG
>Fam_1331_19_1 Nr. of seq. 1 Alignment length(with gaps) = 19 Alignment score = 0.000000
CAAGTTTCTTAGGAAAGGA
>Fam_1332_19_1 Nr. of seq. 1 Alignment length(with gaps) = 19 Alignment score = 0.000000
CTACTCTTAAAATATTCAG
>Fam_1333_19_1 Nr. of seq. 1 Alignment length(with gaps) = 19 Alignment score = 0.000000
AAATTTAGACCAGGATAGA
>Fam_1334_19_1 Nr. of seq. 1 Alignment length(with gaps) = 19 Alignment score = 0.000000
ATTTTACTATCCATTTACC
>Fam_1335_19_1 Nr. of seq. 1 Alignment length(with gaps) = 19 Alignment score = 0.000000
TTACAGGAGATAGATTGAG
>Fam_1336_19_1 Nr. of seq. 1 Alignment length(with gaps) = 19 Alignment score = 0.000000
ATATTCCAGTAATTTTACC
>Fam_1337_19_1 Nr. of seq. 1 Alignment length(with gaps) = 19 Alignment score = 0.000000
TAATTTTTACATTGTTAAC
>Fam_1338_19_1 Nr. of seq. 1 Alignment length(with gaps) = 19 Alignment score = 0.000000
TGGTACGTTGGGAGCACAA
>Fam_1339_19_1 Nr. of seq. 1 Alignment length(with gaps) = 19 Alignment score = 0.000000
AATGGCACTCTAAGTACCA
>Fam_1340_19_1 Nr. of seq. 1 Alignment length(with gaps) = 19 Alignment score = 0.000000
AAAATAATCCCTGATTCTT
>Fam_1341_19_1 Nr. of seq. 1 Alignment length(with gaps) = 19 Alignment score = 0.000000

AAATTTGGAAAACTTATAA
>Fam_1342_19_1 Nr. of seq. 1 Alignment length(with gaps) = 19 Alignment score = 0.000000
AAAGGAAGGACTAAACAGA
>Fam_1343_19_1 Nr. of seq. 1 Alignment length(with gaps) = 19 Alignment score = 0.000000
CTTATCTTGAGAAGTGAGG
>Fam_1344_19_1 Nr. of seq. 1 Alignment length(with gaps) = 19 Alignment score = 0.000000
CTCTTTACATCTTGTTTTG
>Fam_1345_19_1 Nr. of seq. 1 Alignment length(with gaps) = 19 Alignment score = 0.000000
TCTAAGCTCATTTTTGTTC
>Fam_1346_19_1 Nr. of seq. 1 Alignment length(with gaps) = 19 Alignment score = 0.000000
GGATATCGTTATTTTGTGC
>Fam_1347_19_1 Nr. of seq. 1 Alignment length(with gaps) = 19 Alignment score = 0.000000
TAAATTGAGGTCTTGAAGT
>Fam_1348_19_1 Nr. of seq. 1 Alignment length(with gaps) = 19 Alignment score = 0.000000
ATTTAAAGCTAAAATCGGT
>Fam_1349_19_1 Nr. of seq. 1 Alignment length(with gaps) = 19 Alignment score = 0.000000
ATCACCGTTATGATTACAA
>Fam_1350_18_1 Nr. of seq. 1 Alignment length(with gaps) = 18 Alignment score = 0.000000
GCCGTACTCGTTGTTACT
>Fam_1351_18_1 Nr. of seq. 1 Alignment length(with gaps) = 18 Alignment score = 0.000000
GAGGACGACTCCTCCACC
>Fam_1352_18_1 Nr. of seq. 1 Alignment length(with gaps) = 18 Alignment score = 0.000000
CTGAGGTTCCAGCTCCGA
>Fam_1353_18_1 Nr. of seq. 1 Alignment length(with gaps) = 18 Alignment score = 0.000000
TGGAATTATTTGGTTAAG
>Fam_1354_18_1 Nr. of seq. 1 Alignment length(with gaps) = 18 Alignment score = 0.000000
AGTTAGAACTAGAATTAG
>Fam_1355_18_1 Nr. of seq. 1 Alignment length(with gaps) = 18 Alignment score = 0.000000
TCATCGACTACTACGTCG
>Fam_1356_18_1 Nr. of seq. 1 Alignment length(with gaps) = 18 Alignment score = 0.000000
GTGGTAGGTACAATAGGT
>Fam_1357_18_1 Nr. of seq. 1 Alignment length(with gaps) = 18 Alignment score = 0.000000
TCCCGTTCCACATTCCAT
>Fam_1358_18_1 Nr. of seq. 1 Alignment length(with gaps) = 18 Alignment score = 0.000000
CCTGTATTCTCATGCTTC
>Fam_1359_18_1 Nr. of seq. 1 Alignment length(with gaps) = 18 Alignment score = 0.000000
TTAGGAAGATCTCCAATA
>Fam_1360_18_1 Nr. of seq. 1 Alignment length(with gaps) = 18 Alignment score = 0.000000

TATGCAGCTACCTCCGTA
>Fam_1361_18_1 Nr. of seq. 1 Alignment length(with gaps) = 18 Alignment score = 0.000000
TTTTTTGTGAACACGATT
>Fam_1362_18_1 Nr. of seq. 1 Alignment length(with gaps) = 18 Alignment score = 0.000000
ATAGTGACCCAGAATCAA
>Fam_1363_18_1 Nr. of seq. 1 Alignment length(with gaps) = 18 Alignment score = 0.000000
TTACCAGTTTTAGGCTTT
>Fam_1364_18_1 Nr. of seq. 1 Alignment length(with gaps) = 18 Alignment score = 0.000000
CTGTTAACTCTACCAATT
>Fam_1365_18_1 Nr. of seq. 1 Alignment length(with gaps) = 18 Alignment score = 0.000000
CTCTTCATCAACAGTGTT
>Fam_1366_18_1 Nr. of seq. 1 Alignment length(with gaps) = 18 Alignment score = 0.000000
GTGATTTCCGTGATTTCT
>Fam_1367_18_1 Nr. of seq. 1 Alignment length(with gaps) = 18 Alignment score = 0.000000
CAAGGTCATACAGGTTTG
>Fam_1368_18_1 Nr. of seq. 1 Alignment length(with gaps) = 18 Alignment score = 0.000000
TTCCTTAGACTCCATAGA
>Fam_1369_18_1 Nr. of seq. 1 Alignment length(with gaps) = 18 Alignment score = 0.000000
AGATAATCAACTTCGAGC
>Fam_1370_18_1 Nr. of seq. 1 Alignment length(with gaps) = 18 Alignment score = 0.000000
TTGGAGTATCCAGAGTGT
>Fam_1371_18_1 Nr. of seq. 1 Alignment length(with gaps) = 18 Alignment score = 0.000000
AATACCACATTCCTGTCA
>Fam_1372_18_1 Nr. of seq. 1 Alignment length(with gaps) = 18 Alignment score = 0.000000
CAGTATTCTATATTAGAC
>Fam_1373_18_1 Nr. of seq. 1 Alignment length(with gaps) = 18 Alignment score = 0.000000
TTATTTCACAAGTCTCTC
>Fam_1374_18_1 Nr. of seq. 1 Alignment length(with gaps) = 18 Alignment score = 0.000000
CTGAGTCCAGTACGTTTA
>Fam_1375_18_1 Nr. of seq. 1 Alignment length(with gaps) = 18 Alignment score = 0.000000
GAATTGACCTGCTTCAGA
>Fam_1376_18_1 Nr. of seq. 1 Alignment length(with gaps) = 18 Alignment score = 0.000000
TGTTGAAACACCTGCAAA
>Fam_1377_18_1 Nr. of seq. 1 Alignment length(with gaps) = 18 Alignment score = 0.000000
TCTTGAGCTGCCGCTACT
>Fam_1378_18_1 Nr. of seq. 1 Alignment length(with gaps) = 18 Alignment score = 0.000000
TCCAGTTTCGGCTTCAAT
>Fam_1379_18_1 Nr. of seq. 1 Alignment length(with gaps) = 18 Alignment score = 0.000000

ACCGGATTTGATTTAAAC
>Fam_1380_18_1 Nr. of seq. 1 Alignment length(with gaps) = 18 Alignment score = 0.000000
GAAGAACTTGAAGATGTC
>Fam_1381_18_1 Nr. of seq. 1 Alignment length(with gaps) = 18 Alignment score = 0.000000
ACTGTATAATCTGGCTGC
>Fam_1382_18_1 Nr. of seq. 1 Alignment length(with gaps) = 18 Alignment score = 0.000000
CAGATGCAGGTNCAGGTG
>Fam_1383_18_1 Nr. of seq. 1 Alignment length(with gaps) = 18 Alignment score = 0.000000
ACAAAAAGATTACGCAAA
>Fam_1384_18_1 Nr. of seq. 1 Alignment length(with gaps) = 18 Alignment score = 0.000000
AAACTTGTTGTAAATCGG
>Fam_1385_18_1 Nr. of seq. 1 Alignment length(with gaps) = 18 Alignment score = 0.000000
GAACCTGAACTAAGACTT
>Fam_1386_18_1 Nr. of seq. 1 Alignment length(with gaps) = 18 Alignment score = 0.000000
TGGAATGGTGCTACACCC
>Fam_1387_18_1 Nr. of seq. 1 Alignment length(with gaps) = 18 Alignment score = 0.000000
CGTTTATTGACATTCTGC
>Fam_1388_18_1 Nr. of seq. 1 Alignment length(with gaps) = 18 Alignment score = 0.000000
TGGTGCAGAAGCCGGTGT
>Fam_1389_18_1 Nr. of seq. 1 Alignment length(with gaps) = 18 Alignment score = 0.000000
TTTATAAGAAAGGTTCGA
>Fam_1390_18_1 Nr. of seq. 1 Alignment length(with gaps) = 18 Alignment score = 0.000000
TTCAACCGGAGTTACAGT
>Fam_1391_18_1 Nr. of seq. 1 Alignment length(with gaps) = 18 Alignment score = 0.000000
CAGATTGATCATCGAACC
>Fam_1392_18_1 Nr. of seq. 1 Alignment length(with gaps) = 18 Alignment score = 0.000000
CACTTGCCTTTCCAGTCA
>Fam_1393_18_1 Nr. of seq. 1 Alignment length(with gaps) = 18 Alignment score = 0.000000
GAATCTTCGTTGGAGGAA
>Fam_1394_18_1 Nr. of seq. 1 Alignment length(with gaps) = 18 Alignment score = 0.000000
AATAACTCTCATTCTCTC
>Fam_1395_18_1 Nr. of seq. 1 Alignment length(with gaps) = 18 Alignment score = 0.000000
TGTTTTATTTCCAGATCC
>Fam_1396_18_1 Nr. of seq. 1 Alignment length(with gaps) = 18 Alignment score = 0.000000
TTTAGTAAGACTGTTCTG
>Fam_1397_18_1 Nr. of seq. 1 Alignment length(with gaps) = 18 Alignment score = 0.000000
CTGGATTCTCTTTTTCTG
>Fam_1398_18_1 Nr. of seq. 1 Alignment length(with gaps) = 18 Alignment score = 0.000000

ATTTTTAAAAAGCTAACC
>Fam_1399_18_1 Nr. of seq. 1 Alignment length(with gaps) = 18 Alignment score = 0.000000
ACAGTTTTCACATTCAGA
>Fam_1400_18_1 Nr. of seq. 1 Alignment length(with gaps) = 18 Alignment score = 0.000000
CAAATGATGCTGGAATAT
>Fam_1401_18_1 Nr. of seq. 1 Alignment length(with gaps) = 18 Alignment score = 0.000000
GATAAACTTCCATACCCG
>Fam_1402_18_1 Nr. of seq. 1 Alignment length(with gaps) = 18 Alignment score = 0.000000
ATTAAACTCGACAATTCA
>Fam_1403_18_1 Nr. of seq. 1 Alignment length(with gaps) = 18 Alignment score = 0.000000
TGAATGAGACGGTAGCTC
>Fam_1404_18_1 Nr. of seq. 1 Alignment length(with gaps) = 18 Alignment score = 0.000000
CGGGTTCGTCACCGTTGT
>Fam_1405_18_1 Nr. of seq. 1 Alignment length(with gaps) = 18 Alignment score = 0.000000
GCCGTCGTTCGGCTGGAT
>Fam_1406_18_1 Nr. of seq. 1 Alignment length(with gaps) = 18 Alignment score = 0.000000
CGGTTTCGGGCTCGGAGT
>Fam_1407_18_1 Nr. of seq. 1 Alignment length(with gaps) = 18 Alignment score = 0.000000
GAGAACGGCGCCGACGAC
>Fam_1408_18_1 Nr. of seq. 1 Alignment length(with gaps) = 18 Alignment score = 0.000000
CCACCATCTCCTCCGCGT
>Fam_1409_18_1 Nr. of seq. 1 Alignment length(with gaps) = 18 Alignment score = 0.000000
GTTTGGTTTCCTGTAGTC
>Fam_1410_18_1 Nr. of seq. 1 Alignment length(with gaps) = 18 Alignment score = 0.000000
TTGTGGTAGTTGAAGTAC
>Fam_1411_18_1 Nr. of seq. 1 Alignment length(with gaps) = 18 Alignment score = 0.000000
GCTTGCTGGTTGTNGTCG
>Fam_1412_18_1 Nr. of seq. 1 Alignment length(with gaps) = 18 Alignment score = 0.000000
ACTACAAGTACCACCGAA
>Fam_1413_17_1 Nr. of seq. 1 Alignment length(with gaps) = 17 Alignment score = 0.000000
TCATCCGTTCGAGCGCA
>Fam_1414_17_1 Nr. of seq. 1 Alignment length(with gaps) = 17 Alignment score = 0.000000
CGCGATCACCACGTCAC
>Fam_1415_17_1 Nr. of seq. 1 Alignment length(with gaps) = 17 Alignment score = 0.000000
ACCTCCGCTCGTACTGC
>Fam_1416_17_1 Nr. of seq. 1 Alignment length(with gaps) = 17 Alignment score = 0.000000
TAAGTAAAATACATGCT
>Fam_1417_17_1 Nr. of seq. 1 Alignment length(with gaps) = 17 Alignment score = 0.000000

TGCATTTTGAGTACGGT
>Fam_1418_17_1 Nr. of seq. 1 Alignment length(with gaps) = 17 Alignment score = 0.000000
TTTGTGTAATTGACCCG
>Fam_1419_17_1 Nr. of seq. 1 Alignment length(with gaps) = 17 Alignment score = 0.000000
TAGTTTTTGTGTGATAG
>Fam_1420_17_1 Nr. of seq. 1 Alignment length(with gaps) = 17 Alignment score = 0.000000
AGAAGAGTTTTGAGTTA
>Fam_1421_17_1 Nr. of seq. 1 Alignment length(with gaps) = 17 Alignment score = 0.000000
TATGTCTATTATCCGAT
>Fam_1422_17_1 Nr. of seq. 1 Alignment length(with gaps) = 17 Alignment score = 0.000000
ACCGAATGAGTCCAGTC
>Fam_1423_17_1 Nr. of seq. 1 Alignment length(with gaps) = 17 Alignment score = 0.000000
CTACAGGTTAAGTAATT
>Fam_1424_17_1 Nr. of seq. 1 Alignment length(with gaps) = 17 Alignment score = 0.000000
AACGAAGTTATTCCATG
>Fam_1425_17_1 Nr. of seq. 1 Alignment length(with gaps) = 17 Alignment score = 0.000000
TTAGAGAGAAGTAAGCT
>Fam_1426_17_1 Nr. of seq. 1 Alignment length(with gaps) = 17 Alignment score = 0.000000
GAATGAGATGATCTAAA
>Fam_1427_17_1 Nr. of seq. 1 Alignment length(with gaps) = 17 Alignment score = 0.000000
ACATAAACCACCCAACN
>Fam_1428_17_1 Nr. of seq. 1 Alignment length(with gaps) = 17 Alignment score = 0.000000
AAAATCAAAGTAAGTCG
>Fam_1429_17_1 Nr. of seq. 1 Alignment length(with gaps) = 17 Alignment score = 0.000000
ACCCTGTTTCTAAAGTT
>Fam_1430_17_1 Nr. of seq. 1 Alignment length(with gaps) = 17 Alignment score = 0.000000
TAATATTCTGNTTNCTC
>Fam_1431_17_1 Nr. of seq. 1 Alignment length(with gaps) = 17 Alignment score = 0.000000
TATTTCACAGAGAGATT
>Fam_1432_17_1 Nr. of seq. 1 Alignment length(with gaps) = 17 Alignment score = 0.000000
AAAAGATGTGGATCAGG
>Fam_1433_17_1 Nr. of seq. 1 Alignment length(with gaps) = 17 Alignment score = 0.000000
CTGATTTGGCTTACTGA
>Fam_1434_17_1 Nr. of seq. 1 Alignment length(with gaps) = 17 Alignment score = 0.000000
CCAACTAACTATACTGA
>Fam_1435_17_1 Nr. of seq. 1 Alignment length(with gaps) = 17 Alignment score = 0.000000
GTTTCCTACTAGTTTCA
>Fam_1436_17_1 Nr. of seq. 1 Alignment length(with gaps) = 17 Alignment score = 0.000000

TTTCTTTAAAGGTACGA
>Fam_1437_17_1 Nr. of seq. 1 Alignment length(with gaps) = 17 Alignment score = 0.000000
TTCTTTATCGTCTTGTA
>Fam_1438_17_1 Nr. of seq. 1 Alignment length(with gaps) = 17 Alignment score = 0.000000
AGGAATCAATCATTTTC
>Fam_1439_17_1 Nr. of seq. 1 Alignment length(with gaps) = 17 Alignment score = 0.000000
TCAATTTGTTCCGGATC
>Fam_1440_17_1 Nr. of seq. 1 Alignment length(with gaps) = 17 Alignment score = 0.000000
AGTGGAAGCTGTGTATT
>Fam_1441_17_1 Nr. of seq. 1 Alignment length(with gaps) = 17 Alignment score = 0.000000
GCATTGAGTTTTCTAAC
>Fam_1442_17_1 Nr. of seq. 1 Alignment length(with gaps) = 17 Alignment score = 0.000000
TCATCGTCCATCATACG
>Fam_1443_16_1 Nr. of seq. 1 Alignment length(with gaps) = 16 Alignment score = 0.000000
AAAATCTATGCTGCAA
>Fam_1444_16_1 Nr. of seq. 1 Alignment length(with gaps) = 16 Alignment score = 0.000000
CAACTTTATTTGACTG
>Fam_1445_16_1 Nr. of seq. 1 Alignment length(with gaps) = 16 Alignment score = 0.000000
CTGTTGTTAAACGAAG
>Fam_1446_16_1 Nr. of seq. 1 Alignment length(with gaps) = 16 Alignment score = 0.000000
TATCCTTAGATACTCC
>Fam_1447_16_1 Nr. of seq. 1 Alignment length(with gaps) = 16 Alignment score = 0.000000
GGAATTGACACATTAT
>Fam_1448_16_1 Nr. of seq. 1 Alignment length(with gaps) = 16 Alignment score = 0.000000
AATTGATAATTGGACT
>Fam_1449_16_1 Nr. of seq. 1 Alignment length(with gaps) = 16 Alignment score = 0.000000
TGCCCACTCGCTTGTT
>Fam_1450_16_1 Nr. of seq. 1 Alignment length(with gaps) = 16 Alignment score = 0.000000
TTTTGGGATAAAATAC
>Fam_1451_16_1 Nr. of seq. 1 Alignment length(with gaps) = 16 Alignment score = 0.000000
TCCTCGTCTTCTGCGA
>Fam_1452_16_1 Nr. of seq. 1 Alignment length(with gaps) = 16 Alignment score = 0.000000
TTGAACACTCCTAACG
>Fam_1453_16_1 Nr. of seq. 1 Alignment length(with gaps) = 16 Alignment score = 0.000000
GGCTGTAAACAGATCA
>Fam_1454_16_1 Nr. of seq. 1 Alignment length(with gaps) = 16 Alignment score = 0.000000
GCCATTATCTCAAGTT
>Fam_1455_16_1 Nr. of seq. 1 Alignment length(with gaps) = 16 Alignment score = 0.000000

```
 TTCTAATGGAATCTAT
>Fam_1456_16_1  Nr. of seq. 1 Alignment length(with gaps) = 16 Alignment score = 0.000000
 ACACTTCTAATCCAGC
>Fam_1457_16_1  Nr. of seq. 1 Alignment length(with gaps) = 16 Alignment score = 0.000000
 TTCAAACAGTTAATAN
>Fam_1458_16_1  Nr. of seq. 1 Alignment length(with gaps) = 16 Alignment score = 0.000000
 GTTCTGAAGCGTATAA
>Fam_1459_16_1  Nr. of seq. 1 Alignment length(with gaps) = 16 Alignment score = 0.000000
 TTATAGATCTACGATG
>Fam_1460_16_1  Nr. of seq. 1 Alignment length(with gaps) = 16 Alignment score = 0.000000
 CCAAATCATATTCTTC
>Fam_1461_16_1  Nr. of seq. 1 Alignment length(with gaps) = 16 Alignment score = 0.000000
 ACATGGGATTAGAAAC
>Fam_1462_16_1  Nr. of seq. 1 Alignment length(with gaps) = 16 Alignment score = 0.000000
 CTTTAGTCCTTAGTAG
>Fam_1463_16_1  Nr. of seq. 1 Alignment length(with gaps) = 16 Alignment score = 0.000000
 ACTCGCAGCCAACAGT
>Fam_1464_15_1  Nr. of seq. 1 Alignment length(with gaps) = 15 Alignment score = 0.000000
 GACCGGACCAACACC
>Fam_1465_15_1  Nr. of seq. 1 Alignment length(with gaps) = 15 Alignment score = 0.000000
 ACGACGGCGACAGGA
>Fam_1466_15_1  Nr. of seq. 1 Alignment length(with gaps) = 15 Alignment score = 0.000000
 CCGACCTGCGAGAGA
>Fam_1467_15_1  Nr. of seq. 1 Alignment length(with gaps) = 15 Alignment score = 0.000000
 TCCGGCCGATCGCTC
>Fam_1468_15_1  Nr. of seq. 1 Alignment length(with gaps) = 15 Alignment score = 0.000000
 AGTGGGGAGCAGGGA
>Fam_1469_15_1  Nr. of seq. 1 Alignment length(with gaps) = 15 Alignment score = 0.000000
 CTGAGTACCCGGAGC
>Fam_1470_15_1  Nr. of seq. 1 Alignment length(with gaps) = 15 Alignment score = 0.000000
 TTACGAACCATCGCG
>Fam_1471_15_1  Nr. of seq. 1 Alignment length(with gaps) = 15 Alignment score = 0.000000
 TTATCCGAACCATTT
>Fam_1472_15_1  Nr. of seq. 1 Alignment length(with gaps) = 15 Alignment score = 0.000000
 ACCGTGTAGAGTCAT
>Fam_1473_15_1  Nr. of seq. 1 Alignment length(with gaps) = 15 Alignment score = 0.000000
 GCTAACCTTCATCTG
>Fam_1474_15_1  Nr. of seq. 1 Alignment length(with gaps) = 15 Alignment score = 0.000000
```

GTATCATAGGATCTA
>Fam_1475_15_1 Nr. of seq. 1 Alignment length(with gaps) = 15 Alignment score = 0.000000
TTTAGCCATCGTTAT
>Fam_1476_15_1 Nr. of seq. 1 Alignment length(with gaps) = 15 Alignment score = 0.000000
TTTGGGCTGTAAACC
>Fam_1477_15_1 Nr. of seq. 1 Alignment length(with gaps) = 15 Alignment score = 0.000000
CTTAATAGTCTATGG
>Fam_1478_15_1 Nr. of seq. 1 Alignment length(with gaps) = 15 Alignment score = 0.000000
CCATGAGCATTATTC
>Fam_1479_15_1 Nr. of seq. 1 Alignment length(with gaps) = 15 Alignment score = 0.000000
AGTAAAAGTGCATTC
>Fam_1480_15_1 Nr. of seq. 1 Alignment length(with gaps) = 15 Alignment score = 0.000000
GCTTTCATCGATCTC
>Fam_1481_15_1 Nr. of seq. 1 Alignment length(with gaps) = 15 Alignment score = 0.000000
TAACAGCAAAACTCG
>Fam_1482_15_1 Nr. of seq. 1 Alignment length(with gaps) = 15 Alignment score = 0.000000
TGTAGGACTTGCACT
>Fam_1483_15_1 Nr. of seq. 1 Alignment length(with gaps) = 15 Alignment score = 0.000000
CCTGAAGAGAAGCCT
>Fam_1484_15_1 Nr. of seq. 1 Alignment length(with gaps) = 15 Alignment score = 0.000000
ATCTTCAGGTACCGT
>Fam_1485_15_1 Nr. of seq. 1 Alignment length(with gaps) = 15 Alignment score = 0.000000
CTCTTTTTCAGAGAC
>Fam_1486_15_1 Nr. of seq. 1 Alignment length(with gaps) = 15 Alignment score = 0.000000
CAGGAAGAAGAGCCG
>Fam_1487_15_1 Nr. of seq. 1 Alignment length(with gaps) = 15 Alignment score = 0.000000
TACTCTGTTGCATTC
>Fam_1488_15_1 Nr. of seq. 1 Alignment length(with gaps) = 15 Alignment score = 0.000000
TGGTTATAAATTAGC
>Fam_1489_15_1 Nr. of seq. 1 Alignment length(with gaps) = 15 Alignment score = 0.000000
TCTGTTACAAATAAC
>Fam_1490_15_1 Nr. of seq. 1 Alignment length(with gaps) = 15 Alignment score = 0.000000
CGAAGCTTATCTGAC
>Fam_1491_15_1 Nr. of seq. 1 Alignment length(with gaps) = 15 Alignment score = 0.000000
TATTGGAAGGGTCTT
>Fam_1492_15_1 Nr. of seq. 1 Alignment length(with gaps) = 15 Alignment score = 0.000000
TACGGAAATAACACC
>Fam_1493_15_1 Nr. of seq. 1 Alignment length(with gaps) = 15 Alignment score = 0.000000

```
 CACAGATCCACAGAC
>Fam_1494_15_1  Nr. of seq. 1 Alignment length(with gaps) = 15 Alignment score = 0.000000
 GTGGGATGTGAGAGT
>Fam_1495_15_1  Nr. of seq. 1 Alignment length(with gaps) = 15 Alignment score = 0.000000
 TTCGGCGTCTTCGAT
>Fam_1496_14_1  Nr. of seq. 1 Alignment length(with gaps) = 14 Alignment score = 0.000000
 GCGTCCCGCGTCCT
>Fam_1497_14_1  Nr. of seq. 1 Alignment length(with gaps) = 14 Alignment score = 0.000000
 GGCAGGTCGTAAGC
>Fam_1498_14_1  Nr. of seq. 1 Alignment length(with gaps) = 14 Alignment score = 0.000000
 CTGAGGGAGAGAGC
>Fam_1499_14_1  Nr. of seq. 1 Alignment length(with gaps) = 14 Alignment score = 0.000000
 AAGAAAGATCTGCC
>Fam_1500_14_1  Nr. of seq. 1 Alignment length(with gaps) = 14 Alignment score = 0.000000
 TTCGTGAGCACTTA
>Fam_1501_14_1  Nr. of seq. 1 Alignment length(with gaps) = 14 Alignment score = 0.000000
 ATAGAGTCACCATC
>Fam_1502_14_1  Nr. of seq. 1 Alignment length(with gaps) = 14 Alignment score = 0.000000
 TTTTGTTGCACTGC
>Fam_1503_14_1  Nr. of seq. 1 Alignment length(with gaps) = 14 Alignment score = 0.000000
 GTATGAAAATGACC
>Fam_1504_14_1  Nr. of seq. 1 Alignment length(with gaps) = 14 Alignment score = 0.000000
 ACCAGTTGGATTTT
>Fam_1505_14_1  Nr. of seq. 1 Alignment length(with gaps) = 14 Alignment score = 0.000000
 AACTCGGGAAGCGA
>Fam_1506_14_1  Nr. of seq. 1 Alignment length(with gaps) = 14 Alignment score = 0.000000
 TCACATTACGTTTC
>Fam_1507_14_1  Nr. of seq. 1 Alignment length(with gaps) = 14 Alignment score = 0.000000
 CTTATTATTCCACT
>Fam_1508_14_1  Nr. of seq. 1 Alignment length(with gaps) = 14 Alignment score = 0.000000
 TGGTATTTATGAGT
>Fam_1509_14_1  Nr. of seq. 1 Alignment length(with gaps) = 14 Alignment score = 0.000000
 AGATTACTGAGCAG
>Fam_1510_14_1  Nr. of seq. 1 Alignment length(with gaps) = 14 Alignment score = 0.000000
 AATCAAAAACTGCG
>Fam_1511_14_1  Nr. of seq. 1 Alignment length(with gaps) = 14 Alignment score = 0.000000
 TCACATAGAATCTG
>Fam_1512_14_1  Nr. of seq. 1 Alignment length(with gaps) = 14 Alignment score = 0.000000
```

TAGCTGTAGCTCGG
>Fam_1513_13_1 Nr. of seq. 1 Alignment length(with gaps) = 13 Alignment score = 0.000000
CCAGTCAGCTTTC
>Fam_1514_13_1 Nr. of seq. 1 Alignment length(with gaps) = 13 Alignment score = 0.000000
AGTTTAGCAGGCA
>Fam_1515_13_1 Nr. of seq. 1 Alignment length(with gaps) = 13 Alignment score = 0.000000
ATCACTATTTCTA
>Fam_1516_13_1 Nr. of seq. 1 Alignment length(with gaps) = 13 Alignment score = 0.000000
TGTCAGCTATTGA
>Fam_1517_13_1 Nr. of seq. 1 Alignment length(with gaps) = 13 Alignment score = 0.000000
TTATAGTGAATTT
>Fam_1518_13_1 Nr. of seq. 1 Alignment length(with gaps) = 13 Alignment score = 0.000000
TTGCACTCACACC
>Fam_1519_13_1 Nr. of seq. 1 Alignment length(with gaps) = 13 Alignment score = 0.000000
TCTTTTATAAGTG
>Fam_1520_13_1 Nr. of seq. 1 Alignment length(with gaps) = 13 Alignment score = 0.000000
AAAACGAAATCCG
>Fam_1521_13_1 Nr. of seq. 1 Alignment length(with gaps) = 13 Alignment score = 0.000000
AACAGATCACTAA
>Fam_1522_13_1 Nr. of seq. 1 Alignment length(with gaps) = 13 Alignment score = 0.000000
TTAGGTTTTATGC
>Fam_1523_12_1 Nr. of seq. 1 Alignment length(with gaps) = 12 Alignment score = 0.000000
TCGACGGTCTGT
>Fam_1524_12_1 Nr. of seq. 1 Alignment length(with gaps) = 12 Alignment score = 0.000000
GTGTCAGTCGGN
>Fam_1525_12_1 Nr. of seq. 1 Alignment length(with gaps) = 12 Alignment score = 0.000000
TGTACTTCCATG
>Fam_1526_12_1 Nr. of seq. 1 Alignment length(with gaps) = 12 Alignment score = 0.000000
AACTCGGGCCGT
>Fam_1527_12_1 Nr. of seq. 1 Alignment length(with gaps) = 12 Alignment score = 0.000000
GGTTTAAAATNG
>Fam_1528_12_1 Nr. of seq. 1 Alignment length(with gaps) = 12 Alignment score = 0.000000
GAGAGGGTGTGT
>Fam_1529_12_1 Nr. of seq. 1 Alignment length(with gaps) = 12 Alignment score = 0.000000
TTCCTCTCCGAA
>Fam_1530_12_1 Nr. of seq. 1 Alignment length(with gaps) = 12 Alignment score = 0.000000
TGTGCCCGACTT
>Fam_1531_12_1 Nr. of seq. 1 Alignment length(with gaps) = 12 Alignment score = 0.000000

TTGCTCCCTCAA
>Fam_1532_12_1 Nr. of seq. 1 Alignment length(with gaps) = 12 Alignment score = 0.000000
ACTTGCTAATCG
>Fam_1533_12_1 Nr. of seq. 1 Alignment length(with gaps) = 12 Alignment score = 0.000000
ACAGGTGCAGGG
>Fam_1534_11_1 Nr. of seq. 1 Alignment length(with gaps) = 11 Alignment score = 0.000000
TTATCAAAGTG
>Fam_1535_11_1 Nr. of seq. 1 Alignment length(with gaps) = 11 Alignment score = 0.000000
AGGAATGAGAC
>Fam_1536_11_1 Nr. of seq. 1 Alignment length(with gaps) = 11 Alignment score = 0.000000
AAATTCACAGC
>Fam_1537_10_1 Nr. of seq. 1 Alignment length(with gaps) = 10 Alignment score = 0.000000
ACCTTACCTC
>Fam_1538_10_1 Nr. of seq. 1 Alignment length(with gaps) = 10 Alignment score = 0.000000
GCACCACAAC
>Fam_1539_10_1 Nr. of seq. 1 Alignment length(with gaps) = 10 Alignment score = 0.000000
AGATCCCAGG
>Fam_1540_10_1 Nr. of seq. 1 Alignment length(with gaps) = 10 Alignment score = 0.000000
TAATTTACTC
>Fam_1541_10_1 Nr. of seq. 1 Alignment length(with gaps) = 10 Alignment score = 0.000000
GCGGGCAGTC

Table S6b. Bacteria satellite families

```
>Fam_1_14_23  Nr. of seq. 23 Alignment length(with gaps) = 16 Alignment score = 0.673254
 GATAGaAgATAGGA
>Fam_2_14_12  Nr. of seq. 12 Alignment length(with gaps) = 16 Alignment score = 0.641256
 CGGCAagCGGCAaG
>Fam_3_12_12  Nr. of seq. 12 Alignment length(with gaps) = 13 Alignment score = 0.636364
 CAAcaGCAAnaG
>Fam_4_14_11  Nr. of seq. 11 Alignment length(with gaps) = 16 Alignment score = 0.636364
 GaGgCGCGaGgCGC
>Fam_5_12_10  Nr. of seq. 10 Alignment length(with gaps) = 14 Alignment score = 0.652910
 GCtgGTGCtgGT
>Fam_6_14_10  Nr. of seq. 10 Alignment length(with gaps) = 14 Alignment score = 0.860317
 tCGGGAAtcGGGAA
>Fam_7_20_9  Nr. of seq. 9 Alignment length(with gaps) = 22 Alignment score = 0.613216
 GATATAttTnGAganTCGCt
>Fam_8_18_9  Nr. of seq. 9 Alignment length(with gaps) = 18 Alignment score = 0.754630
 GAcAGcGAcTCnGAcTCa
>Fam_9_15_9  Nr. of seq. 9 Alignment length(with gaps) = 16 Alignment score = 0.837963
 CGCGCcGGCCAGGTT
>Fam_10_16_9  Nr. of seq. 9 Alignment length(with gaps) = 16 Alignment score = 0.814815
 GCcGAaCCGCcGAaCC
>Fam_11_14_9  Nr. of seq. 9 Alignment length(with gaps) = 14 Alignment score = 0.809524
 AGCtAcAAGCtAcA
>Fam_12_12_9  Nr. of seq. 9 Alignment length(with gaps) = 13 Alignment score = 0.638177
 CcgACGCcgACg
>Fam_13_96_8  Nr. of seq. 8 Alignment length(with gaps) = 111 Alignment score = 0.661197
 CGCGcAAAGCcGCcGTcGAAGCcGCTATtGCCCGcGCCAAAGCaCGcAAGCtGGAaCAGcaaCAGGCtaAtGCgGAACCaGaaGAACaGGTCGAtC
>Fam_14_39_8  Nr. of seq. 8 Alignment length(with gaps) = 39 Alignment score = 0.941392
 GGCTGCTGATAcTGCgGCTGCGGCGCaACCGGtTGTTGC
>Fam_15_14_8  Nr. of seq. 8 Alignment length(with gaps) = 14 Alignment score = 0.746599
 gCcGAACGCcGAac
>Fam_16_14_8  Nr. of seq. 8 Alignment length(with gaps) = 14 Alignment score = 0.720663
 cTAGAAcCTAGAAC
>Fam_17_100_7  Nr. of seq. 7 Alignment length(with gaps) = 108 Alignment score = 0.661817
 GGATGCGGCGTgAACGCCTTATCCGGCCTACGgatGGCgCnaGAaTnTGTAGGCcTGATAAgacGCGntAGCgtcGCATCAGGCATntGgCaCnAtTGCC
>Fam_18_14_7  Nr. of seq. 7 Alignment length(with gaps) = 16 Alignment score = 0.646825
```

cATCGCAcATCGCA
>Fam_19_16_7 Nr. of seq. 7 Alignment length(with gaps) = 16 Alignment score = 0.904762
gTAGGGGAgTAGGGGA
>Fam_20_12_7 Nr. of seq. 7 Alignment length(with gaps) = 14 Alignment score = 0.688209
GCTTCGtCtTCg
>Fam_21_12_7 Nr. of seq. 7 Alignment length(with gaps) = 14 Alignment score = 0.664399
TGGCTCTGGCtC
>Fam_22_14_7 Nr. of seq. 7 Alignment length(with gaps) = 14 Alignment score = 0.746032
GAGcCGtGAGcCGt
>Fam_23_69_6 Nr. of seq. 6 Alignment length(with gaps) = 70 Alignment score = 0.773651
ACAGAATTAGGAATtGTAACtGATGTTAAACCAGAACAAnCTgcAAAAGCaCCaTcTCCAATAGtAGTC
>Fam_24_46_6 Nr. of seq. 6 Alignment length(with gaps) = 51 Alignment score = 0.627233
TGTGGGAACTCTTACAAATTGaGATTTTACggttaAACTTTAAAaT
>Fam_25_46_6 Nr. of seq. 6 Alignment length(with gaps) = 49 Alignment score = 0.650340
ACAtTTTagAAgTttnaCTGTAAAaTCacGaTTTGTGGTAGTTCCC
>Fam_26_37_6 Nr. of seq. 6 Alignment length(with gaps) = 38 Alignment score = 0.693567
cAaAACGCTtTccTATGGgTCGCGTTgGaaAACTCAg
>Fam_27_14_6 Nr. of seq. 6 Alignment length(with gaps) = 16 Alignment score = 0.728472
CCgGTCGCCgGTCG
>Fam_28_15_6 Nr. of seq. 6 Alignment length(with gaps) = 15 Alignment score = 0.717037
GCCGatGTTGnCGNt
>Fam_29_14_6 Nr. of seq. 6 Alignment length(with gaps) = 14 Alignment score = 0.692064
CCAcCGACCAcCGA
>Fam_30_12_6 Nr. of seq. 6 Alignment length(with gaps) = 12 Alignment score = 0.644444
gntGCCGCCGCC
>Fam_31_69_5 Nr. of seq. 5 Alignment length(with gaps) = 70 Alignment score = 0.729048
tgtAAATTcTgtAgTTGTCCnATTTCtTTnGGAAGnGttGTGAGTtGGTTATnatnCAAATncAAcnnT
>Fam_32_45_5 Nr. of seq. 5 Alignment length(with gaps) = 45 Alignment score = 0.911111
CTgACTTCGTCAGTTctATCtgCAACCTCAAAACaGTGTTTTGAg
>Fam_33_30_5 Nr. of seq. 5 Alignment length(with gaps) = 30 Alignment score = 0.698889
CCGCTGCCcacaTTGgnGnTaCCGntgTTt
>Fam_34_15_5 Nr. of seq. 5 Alignment length(with gaps) = 17 Alignment score = 0.698039
gtAGGGGCGGGTTtg
>Fam_35_14_5 Nr. of seq. 5 Alignment length(with gaps) = 16 Alignment score = 0.725000
cGTTTGGcGTTTGG
>Fam_36_16_5 Nr. of seq. 5 Alignment length(with gaps) = 16 Alignment score = 0.716667
GCaCgaCnGCAcGAcc
>Fam_37_14_5 Nr. of seq. 5 Alignment length(with gaps) = 15 Alignment score = 0.728889

```
 cggGCGTcgGGCGT
>Fam_38_13_5  Nr. of seq. 5 Alignment length(with gaps) = 14 Alignment score = 0.711905
 CCCGCAcCCGcAn
>Fam_39_14_5  Nr. of seq. 5 Alignment length(with gaps) = 14 Alignment score = 0.723810
 nCCaAGCnCCaAGC
>Fam_40_12_5  Nr. of seq. 5 Alignment length(with gaps) = 12 Alignment score = 0.822222
 CAGCGtCCCaGg
>Fam_41_12_5  Nr. of seq. 5 Alignment length(with gaps) = 12 Alignment score = 0.711111
 nGGCGAgGGcgA
>Fam_42_93_4  Nr. of seq. 4 Alignment length(with gaps) = 103 Alignment score = 0.703344
 GCCGCATCCGgCAaTCAaTGCCTGATGCGACGCTGaCGCGTCTTATCAgGCCTACAactattGCCnGAaCcGTAGGcCGGATAAGGCacTcgc
>Fam_43_39_4  Nr. of seq. 4 Alignment length(with gaps) = 39 Alignment score = 0.965812
 TTTTCTTTCTTcACTTGCGGtGATACTATATTTCCTACA
>Fam_44_36_4  Nr. of seq. 4 Alignment length(with gaps) = 36 Alignment score = 0.845679
 GGtGGTTGaTTgTTATCCATgATcGGCATaATgTTt
>Fam_45_36_4  Nr. of seq. 4 Alignment length(with gaps) = 36 Alignment score = 0.870370
 TTGtTCTTCTACGACcGGtGcTTCcTCTgCAACTgG
>Fam_46_36_4  Nr. of seq. 4 Alignment length(with gaps) = 36 Alignment score = 0.882716
 CTTctGtTTCtGCAATTACTTCcACTTCTTCTgACT
>Fam_47_33_4  Nr. of seq. 4 Alignment length(with gaps) = 34 Alignment score = 0.602941
 CCCCAGCgGggGaATaCaCcctaCaaTTCCTCC
>Fam_48_18_4  Nr. of seq. 4 Alignment length(with gaps) = 18 Alignment score = 1.000000
 GAGCTTTAGCTGCTTCTT
>Fam_49_15_4  Nr. of seq. 4 Alignment length(with gaps) = 16 Alignment score = 0.661458
 CCgGCCAAGaAGgca
>Fam_50_14_4  Nr. of seq. 4 Alignment length(with gaps) = 14 Alignment score = 0.650794
 GaCtCAGGnCtCAg
>Fam_51_14_4  Nr. of seq. 4 Alignment length(with gaps) = 14 Alignment score = 0.746032
 GATgcGCGATgcGC
>Fam_52_14_4  Nr. of seq. 4 Alignment length(with gaps) = 14 Alignment score = 0.682540
 GCGgggAGCGgggA
>Fam_53_12_4  Nr. of seq. 4 Alignment length(with gaps) = 12 Alignment score = 0.627315
 GcCGGgGcCGgg
>Fam_54_12_4  Nr. of seq. 4 Alignment length(with gaps) = 12 Alignment score = 0.703704
 CcGCgACcGCgA
>Fam_55_12_4  Nr. of seq. 4 Alignment length(with gaps) = 12 Alignment score = 0.888889
 CCGAgCTAAGcG
>Fam_56_150_3  Nr. of seq. 3 Alignment length(with gaps) = 170 Alignment score = 0.649673
```

```
GACGCgGAGCGTCCAGAAcgGCATacCcACGCGGAGCaTggGtACGATGGTGTccTTnaGnnCACCTATCGTTCCTCACGCTCCAGCGTgGGAATGCtnTgcGTGACGCTC
tGCGTCAcAgaTCTGCGCCgCGCtgCaCAtTCAgGaccG
>Fam_57_156_3  Nr. of seq. 3 Alignment length(with gaps) = 156 Alignment score = 0.933048

TTTATTATGTAAAAATCAgCAtATCCTGCACCAAAAGAATaTGTcnaTCCTGCAACTATATATCCTCCATCAcTTGTCTGCTGTATGGAAnAAGCCCaATCATAAtTGCTT
CCTCCATAGGTTTTCTCCCAAACCTTATcCCCATTACTaTCAAGC
>Fam_58_114_3  Nr. of seq. 3 Alignment length(with gaps) = 126 Alignment score = 0.760141

GACGCAGAGCGTCGaGAaCTGCATTCCCACGCtGGAGCGTagGGAACGAtaAtCtCAACTATCGTGCGgCGCTCCGCGTCGCATGCCtTTCnGGAcGCTCtGCGTCCTCtT
TGC
>Fam_59_108_3  Nr. of seq. 3 Alignment length(with gaps) = 108 Alignment score = 0.720165
 cCTTCGCGAaCAAGtTCGCtCCTACgGGaGttcgCgGgnccTTGTaGGAGCGAaCTTGctCGCGAAgAGGncGtTaCAgTCgATGgAtTTtCTGCGTCaGgaATATaG
>Fam_60_60_3  Nr. of seq. 3 Alignment length(with gaps) = 65 Alignment score = 0.675214
 ATGGTACTACCTcAACGCtAAtGGTGCtATGGCnACAGGtTGGCTcaAAgacaAtGGTTc
>Fam_61_56_3  Nr. of seq. 3 Alignment length(with gaps) = 60 Alignment score = 0.688889
 ccGTGnCGATCGCaAGCgCGGCGCaGCCGgGCGcaGCGGGTCGgCACCATCggaCc
>Fam_62_46_3  Nr. of seq. 3 Alignment length(with gaps) = 49 Alignment score = 0.643991
 TTTtAAaTtgAtCtGTAAAAnCtTtaAcTTGTgAGAGTTCCCACAt
>Fam_63_48_3  Nr. of seq. 3 Alignment length(with gaps) = 48 Alignment score = 0.791667
 TACGGCAGCACcCAGACcGCnGGnnNNGACAGCnCgCTGACCGCcGGc
>Fam_64_45_3  Nr. of seq. 3 Alignment length(with gaps) = 45 Alignment score = 0.881481
 TCTTCTTGTTGtTTACGAGCTTGtTCATCAGCctGgCGTTTTTGt
>Fam_65_42_3  Nr. of seq. 3 Alignment length(with gaps) = 42 Alignment score = 0.830688
 TCTTCCACTTGTGGTTCcACTACtACtTGtTGCAtTGGtttT
>Fam_66_39_3  Nr. of seq. 3 Alignment length(with gaps) = 39 Alignment score = 0.954416
 TCAgGGGCAACAGAAACACTCTAAGCAAGACGACTCCgA
>Fam_67_39_3  Nr. of seq. 3 Alignment length(with gaps) = 39 Alignment score = 0.931624
 GGTCAAGGAAATACaACcCCTCCAGCAAATAAcGGAGGA
>Fam_68_36_3  Nr. of seq. 3 Alignment length(with gaps) = 36 Alignment score = 0.824074
 TCTTCCTTAGgTTTNGCTAcTTCTTGgaCTTTcaCT
>Fam_69_27_3  Nr. of seq. 3 Alignment length(with gaps) = 27 Alignment score = 0.806584
 TGTCGTTGCCGncgCCGCCaTACAGct
>Fam_70_27_3  Nr. of seq. 3 Alignment length(with gaps) = 27 Alignment score = 0.802469
 GGAATAACGGGAGtgACaGGTcCaACa
>Fam_71_24_3  Nr. of seq. 3 Alignment length(with gaps) = 24 Alignment score = 0.740741
 CAGgtAAGgATCcAggAACAGAtA
>Fam_72_21_3  Nr. of seq. 3 Alignment length(with gaps) = 22 Alignment score = 0.727273
```

tCtCCTATtTCCTaTCTtCTC
>Fam_73_21_3 Nr. of seq. 3 Alignment length(with gaps) = 21 Alignment score = 0.788360
CGCGTgcCTGGAcGGaCGaCT
>Fam_74_21_3 Nr. of seq. 3 Alignment length(with gaps) = 21 Alignment score = 0.873016
AAGaAACAGaAAAgCCAGGTG
>Fam_75_16_3 Nr. of seq. 3 Alignment length(with gaps) = 18 Alignment score = 0.722222
GCTTCGgCGCTTCGnC
>Fam_76_18_3 Nr. of seq. 3 Alignment length(with gaps) = 18 Alignment score = 0.641975
CanAAgaAAgcaGAAGAA
>Fam_77_12_3 Nr. of seq. 3 Alignment length(with gaps) = 16 Alignment score = 0.680556
GACTCGGACTCG
>Fam_78_13_3 Nr. of seq. 3 Alignment length(with gaps) = 16 Alignment score = 0.652778
tTGGTGTTGGTGg
>Fam_79_14_3 Nr. of seq. 3 Alignment length(with gaps) = 16 Alignment score = 0.791667
CCTGAAACCTGAAA
>Fam_80_15_3 Nr. of seq. 3 Alignment length(with gaps) = 16 Alignment score = 0.736111
GCCAaGAAGGcCGtA
>Fam_81_15_3 Nr. of seq. 3 Alignment length(with gaps) = 16 Alignment score = 0.791667
ggCGGTGCTTCCGGT
>Fam_82_16_3 Nr. of seq. 3 Alignment length(with gaps) = 16 Alignment score = 0.777778
TGATTgCtTGATTgCt
>Fam_83_16_3 Nr. of seq. 3 Alignment length(with gaps) = 16 Alignment score = 0.777778
GCaaGGCTGCaaGGCT
>Fam_84_16_3 Nr. of seq. 3 Alignment length(with gaps) = 16 Alignment score = 0.777778
GgtCTTCGGgtCTTCG
>Fam_85_16_3 Nr. of seq. 3 Alignment length(with gaps) = 16 Alignment score = 0.833333
CGcCACGcCGcCACGC
>Fam_86_16_3 Nr. of seq. 3 Alignment length(with gaps) = 16 Alignment score = 0.833333
TTTAAagCTTTATTTa
>Fam_87_12_3 Nr. of seq. 3 Alignment length(with gaps) = 15 Alignment score = 0.718518
GGTGGaGACGGC
>Fam_88_12_3 Nr. of seq. 3 Alignment length(with gaps) = 14 Alignment score = 0.674603
AAAcAGAAAcAG
>Fam_89_12_3 Nr. of seq. 3 Alignment length(with gaps) = 14 Alignment score = 0.761905
TGGCTGTGGCTG
>Fam_90_12_3 Nr. of seq. 3 Alignment length(with gaps) = 13 Alignment score = 0.777778
AAgGTaTAGAAC
>Fam_91_13_3 Nr. of seq. 3 Alignment length(with gaps) = 13 Alignment score = 0.692308

CGAaGCGaCtGaT
>Fam_92_13_3 Nr. of seq. 3 Alignment length(with gaps) = 13 Alignment score = 0.794872
GActCGAACCgCC
>Fam_93_12_3 Nr. of seq. 3 Alignment length(with gaps) = 12 Alignment score = 0.703704
GtCGnTGTCGaT
>Fam_94_12_3 Nr. of seq. 3 Alignment length(with gaps) = 12 Alignment score = 0.777778
ATCTTnATCTTn
>Fam_95_220_2 Nr. of seq. 2 Alignment length(with gaps) = 220 Alignment score = 0.769697

GACGTCAACGGCGACGGaCTcGAcGAccTcATcaTCGGGGCTCCcGGTGCCGACCCGGACaGaCGGTaCgaAtGCcGGcCGCAGCTACGTGGTcTTCGGCAAGCGcaaCGG
CCAACCGGTCGAaTTGGCcaCaaTCGAAAGCGGCACcaGcCGcaACGGCTTTGcCATCAACGGCAGCAATGACTACGATacCgCCGGCgTTgCTGTCAGCGGAGCGGGG
>Fam_96_155_2 Nr. of seq. 2 Alignment length(with gaps) = 155 Alignment score = 0.638710

CTCCAGCGTaGGaAcGCcGTTCcGGACGCTCTGCGTCCGaTCTTGAAcaTGTGcaacGCGACgCaGAAtTTGTGACGCAGAGCGTCACGaAACgGCATGaCaACGCGGAGC
agTcGCACGATAGtcatcTcGaGGcacaaGTcATCGTTCCcaCG
>Fam_97_153_2 Nr. of seq. 2 Alignment length(with gaps) = 153 Alignment score = 0.612200

TTGCGCCAGAGaATGTCcGGcTTGCCGTCGaaagCGanAAaTCGcCcGCCCCGaCGATCTGCCAGcccaGATCGCCCACaGaaCgGtAGaGaaACcGaCGaacAGaAGaCc
GtGCCGTTCATcAaCCAGACGcaaTTGGCACCGcTgGcCtTG
>Fam_98_141_2 Nr. of seq. 2 Alignment length(with gaps) = 141 Alignment score = 0.971631

AATGCCATGCTCTGCAAGATGCTGCATCAGACGCTGAGCCACATCAGGCAaAGGTCTTGATTGTTCATTTTGTACTGGCGGcGgTGGCGCAGGCCAGTTAGGTGCCGGAGG
AATATGTTCAGCCATATTCCGGGCTGGTTG
>Fam_99_138_2 Nr. of seq. 2 Alignment length(with gaps) = 138 Alignment score = 0.978261

CCAACCCTGGAGGGCAACACCTGCCAGGGGAACAAGgaGAGCGGCATCGCCTACGTTGGCAGTGCAGGGGGCATCGCTCGCCAGAACACCTGCAGCGGCAATGAGTACCAT
GGCATCTATGTGGGCgAGCAGGCGCAA
>Fam_100_130_2 Nr. of seq. 2 Alignment length(with gaps) = 130 Alignment score = 0.621795

gCCGGaTGGCGGCTaaCGCCTTAcCCGGCCTACaGaGcCCtgtaTaTCAaAcGGttaCTCAtACacCGcAGGCCCGCGCAAGCGCAGCGCCGCCGGGCAAGctaTAcCAGa
CaCcGTaCCcGTaATTgTc
>Fam_101_130_2 Nr. of seq. 2 Alignment length(with gaps) = 130 Alignment score = 0.989744

CCTTCGCGGACAAGTCCGCTCCTACGCCCGTTGGGCAGAATCAAAAACGGCCTCCTGCCCGAGGGGTAGGAGCGAACTTGTTCGCGAAGACGGTCGCTCAGGCGATGCATG
TcCGGAAAGCTTGGCGGCC
>Fam_102_118_2 Nr. of seq. 2 Alignment length(with gaps) = 118 Alignment score = 0.830508

```
CTTGTTCGCGAAGaCATTGTTTcaAACGaCaAATCTTccGCGACTGTACGGGCCCTTTCGCGAACAAGTTCGCTCCTACGTTcGaGCaTGGGAgaCGGatGGCTTTTGTAG
GAGCGAA
>Fam_103_114_2  Nr. of seq. 2 Alignment length(with gaps) = 114 Alignment score = 0.859649

GcCTGATGCGAcGCtTaACGcGTCTTATCAGGCCTACGcCAGACAGCGCAATAGCCTGATTTaGCGTGAtTTTTGTAGGTCGGATAAGGCGTTTAcGCCGCATCCGaCATC
AAc
>Fam_104_112_2  Nr. of seq. 2 Alignment length(with gaps) = 112 Alignment score = 0.625000

GAGCGCGCCGGAGGCGcGCGCTCCCAGGgGATactGacaCGCCcCCGCcGGGcGCGGGaAccaCCGaTGCCGGATGGcTcCCGCTGtcGCGaGttGaGCTGGggcAcGTGG
g
>Fam_105_111_2  Nr. of seq. 2 Alignment length(with gaps) = 111 Alignment score = 1.000000

CGTGGCGATCGCAAACCCCGCGCCTGGCGACAATGCGGCCCGCAAAACGGGCCGAGGAGGAGCCAGGCAATCACCCCAGAGCCGGGTGCAGCGGGTCGCCACCATCAGCCC
>Fam_106_88_2  Nr. of seq. 2 Alignment length(with gaps) = 88 Alignment score = 0.765152
 GCCCGGATAAGGCGcaAGCCGCcATCCGGGaAcCtCTCCCCGGTGGCGCTGaCGCTTACCGGGGCTACaAACCGaAtGCcaAaCcGTA
>Fam_107_66_2  Nr. of seq. 2 Alignment length(with gaps) = 66 Alignment score = 0.797980
 ATAGTTAATCTAAAACACTTTATgTATTNATNTCATCCAtTTAnGNNacTANaATTTACATTCCAT
>Fam_108_63_2  Nr. of seq. 2 Alignment length(with gaps) = 63 Alignment score = 1.000000
 TGGTATCTCTTTGATCGCTCTTCTGGAATCATGAGAAAAGGACAGGTCAATGATGCTGGGAAT
>Fam_109_60_2  Nr. of seq. 2 Alignment length(with gaps) = 60 Alignment score = 0.933333
 CCGATGTTGTAGCTGCCGGNGTTGGCAAAGCCCAGGTTGTCGNTGCCGANGTTNGCGAAG
>Fam_110_60_2  Nr. of seq. 2 Alignment length(with gaps) = 60 Alignment score = 0.966667
 CGGCAGGCTGGTCAGCTGGTTACCAGAGACCNACAGCTCCTNTAGTCCTGACGGCAGCGC
>Fam_111_57_2  Nr. of seq. 2 Alignment length(with gaps) = 57 Alignment score = 1.000000
 CTAGCGTGGCGACGATGCGGGCTGGGATGGGCCGCTGAGGAGCCGCGCGGTCGAGCT
>Fam_112_54_2  Nr. of seq. 2 Alignment length(with gaps) = 54 Alignment score = 0.759259
 TGAccTCgCGCCGGcGACGATGCAGAGCGaAGCGATGAGcAGGAGCGGCGCaga
>Fam_113_54_2  Nr. of seq. 2 Alignment length(with gaps) = 54 Alignment score = 0.833333
 CCTTTTTCACCTTTaGCaCCTTGcGCTCCAGTcAGACCaCGTTCaCCTcGGTCT
>Fam_114_50_2  Nr. of seq. 2 Alignment length(with gaps) = 50 Alignment score = 0.613333
 AGGCTCGGGAAAaAAcaaTccACaaaACtcAGCCTCGCTaTcTcATTTTC
>Fam_115_45_2  Nr. of seq. 2 Alignment length(with gaps) = 45 Alignment score = 0.940741
 ACTGGACCTCAAGGTGTTCAAGGACCAGCAGGTGCTACcGGTGCc
>Fam_116_45_2  Nr. of seq. 2 Alignment length(with gaps) = 45 Alignment score = 1.000000
 GAACGATTAGAGGCAGAGAGAAAAGAGCAAGAAAGATTGGAAGCA
>Fam_117_45_2  Nr. of seq. 2 Alignment length(with gaps) = 45 Alignment score = 0.911111
 AAGAAGAAAGCGGAgGCCGAGGCGGCGAAAGCaGCGGCGGAcGCG
```

>Fam_118_44_2 Nr. of seq. 2 Alignment length(with gaps) = 44 Alignment score = 0.772727
GCGGGGACAGGCACCgcaAGGAaTcGGCGGGTGCCAGTccCCCT
>Fam_119_42_2 Nr. of seq. 2 Alignment length(with gaps) = 42 Alignment score = 0.809524
GGaATaACTGGTCCTACTGGaGTTACAGGacCTACCGGAATc
>Fam_120_41_2 Nr. of seq. 2 Alignment length(with gaps) = 41 Alignment score = 0.837398
CCaGCcGACTcCCGGGGTGCCTGTCCCcACGaGGACAGGCA
>Fam_121_39_2 Nr. of seq. 2 Alignment length(with gaps) = 39 Alignment score = 0.662393
GGATCTTCCGGTTTCACTTCTGGcTCcTTCggctctttc
>Fam_122_39_2 Nr. of seq. 2 Alignment length(with gaps) = 39 Alignment score = 0.606838
GTGGccGTcCGgCGCgGCCGcgTCCaCGcTCGTCGGcGC
>Fam_123_39_2 Nr. of seq. 2 Alignment length(with gaps) = 39 Alignment score = 0.606838
TgcAAAATGTaGGAACTCaCaCaAAGaTcTAAGTTcaaC
>Fam_124_39_2 Nr. of seq. 2 Alignment length(with gaps) = 39 Alignment score = 1.000000
AATCCGGAACAAAATCCAGCAACTAAACCTGCTACTGAC
>Fam_125_31_2 Nr. of seq. 2 Alignment length(with gaps) = 31 Alignment score = 0.967742
CCCGAGTTGAAGNCAGCCCGTATTCAAGGCG
>Fam_126_30_2 Nr. of seq. 2 Alignment length(with gaps) = 30 Alignment score = 1.000000
TTGTTGTTGCCGAGATTCGCAATGCCCAGG
>Fam_127_30_2 Nr. of seq. 2 Alignment length(with gaps) = 30 Alignment score = 0.966667
CCGAGTTGAACNAGCCGGTGTTGATGTTGC
>Fam_128_30_2 Nr. of seq. 2 Alignment length(with gaps) = 30 Alignment score = 0.933333
AAACCGGTGTTCGAGTCACCCGCATTNNCG
>Fam_129_26_2 Nr. of seq. 2 Alignment length(with gaps) = 26 Alignment score = 0.602564
GGaCaAgGCGGCTaCGGCGGcGGTca
>Fam_130_24_2 Nr. of seq. 2 Alignment length(with gaps) = 24 Alignment score = 1.000000
AGCTTCTTCTTCAGCTTTTTTTGT
>Fam_131_24_2 Nr. of seq. 2 Alignment length(with gaps) = 24 Alignment score = 1.000000
CATCTTCTTTCACATTCTCTTCTA
>Fam_132_23_2 Nr. of seq. 2 Alignment length(with gaps) = 23 Alignment score = 0.739130
TTTTCGGGGATaaCCtATCCCCc
>Fam_133_22_2 Nr. of seq. 2 Alignment length(with gaps) = 22 Alignment score = 0.863636
AGAAGNTGAAAGANCCAAAGAN
>Fam_134_21_2 Nr. of seq. 2 Alignment length(with gaps) = 21 Alignment score = 0.809524
GCCGAGCAaGAaCGcCAACGC
>Fam_135_21_2 Nr. of seq. 2 Alignment length(with gaps) = 21 Alignment score = 0.809524
TGTcTTcTGTcTTTCTTGTTC
>Fam_136_21_2 Nr. of seq. 2 Alignment length(with gaps) = 21 Alignment score = 0.952381
GGTACCGGTGCGCCcGGGATC

>Fam_137_19_2 Nr. of seq. 2 Alignment length(with gaps) = 19 Alignment score = 0.614035
GGCgcGCAGGGCAGAgccg
>Fam_138_19_2 Nr. of seq. 2 Alignment length(with gaps) = 19 Alignment score = 0.622807
CGaACGaCGcGaACGGCCg
>Fam_139_18_2 Nr. of seq. 2 Alignment length(with gaps) = 18 Alignment score = 0.731481
gtACGACCCGGGcCGGCa
>Fam_140_18_2 Nr. of seq. 2 Alignment length(with gaps) = 18 Alignment score = 0.666667
TcGcCGGGTaaCGCTGGG
>Fam_141_18_2 Nr. of seq. 2 Alignment length(with gaps) = 18 Alignment score = 0.629630
GCTAaAcAAcATAGAtTA
>Fam_142_18_2 Nr. of seq. 2 Alignment length(with gaps) = 18 Alignment score = 0.851852
CCTTGGTTGTcGTTTCCa
>Fam_143_18_2 Nr. of seq. 2 Alignment length(with gaps) = 18 Alignment score = 0.722222
AGTTAAATAATtcaCAGa
>Fam_144_18_2 Nr. of seq. 2 Alignment length(with gaps) = 18 Alignment score = 0.703704
AACgGCAGaGaAAAGGTc
>Fam_145_16_2 Nr. of seq. 2 Alignment length(with gaps) = 16 Alignment score = 0.718750
TgACTTATTgACTTAt
>Fam_146_16_2 Nr. of seq. 2 Alignment length(with gaps) = 16 Alignment score = 0.635417
cCACCATgcCACCATg
>Fam_147_16_2 Nr. of seq. 2 Alignment length(with gaps) = 16 Alignment score = 0.635417
GCCGaGAAGAcAGGct
>Fam_148_16_2 Nr. of seq. 2 Alignment length(with gaps) = 16 Alignment score = 1.000000
GAGGCGGTGAGGCAGT
>Fam_149_16_2 Nr. of seq. 2 Alignment length(with gaps) = 16 Alignment score = 0.666667
cGCTTcTCcGCTTcTC
>Fam_150_16_2 Nr. of seq. 2 Alignment length(with gaps) = 16 Alignment score = 1.000000
CCGCAGCACCGCAACG
>Fam_151_16_2 Nr. of seq. 2 Alignment length(with gaps) = 16 Alignment score = 0.666667
GAACTGacGAACTGac
>Fam_152_16_2 Nr. of seq. 2 Alignment length(with gaps) = 16 Alignment score = 0.666667
CaGCTaAACaGCTaAA
>Fam_153_15_2 Nr. of seq. 2 Alignment length(with gaps) = 15 Alignment score = 0.644444
GAgCcGGCGGCTcaG
>Fam_154_15_2 Nr. of seq. 2 Alignment length(with gaps) = 15 Alignment score = 0.822222
CCAGAACCGTTaCCa
>Fam_155_15_2 Nr. of seq. 2 Alignment length(with gaps) = 15 Alignment score = 0.644444
TAAACaaGAaAATAa

>Fam_156_15_2 Nr. of seq. 2 Alignment length(with gaps) = 15 Alignment score = 1.000000
TTTAAATGAAGAAAA
>Fam_157_15_2 Nr. of seq. 2 Alignment length(with gaps) = 15 Alignment score = 1.000000
TGGACGGGCGCTTGC
>Fam_158_15_2 Nr. of seq. 2 Alignment length(with gaps) = 15 Alignment score = 0.822222
TGCCCGTACTGGgcC
>Fam_159_14_2 Nr. of seq. 2 Alignment length(with gaps) = 14 Alignment score = 0.773810
ATAGAGtATAGAGt
>Fam_160_14_2 Nr. of seq. 2 Alignment length(with gaps) = 14 Alignment score = 0.619048
CGTTaaCCGTTaaC
>Fam_161_14_2 Nr. of seq. 2 Alignment length(with gaps) = 14 Alignment score = 0.809524
CaTTCGACaTTCGA
>Fam_162_14_2 Nr. of seq. 2 Alignment length(with gaps) = 14 Alignment score = 0.619048
aAAGAaGaAAGAaG
>Fam_163_14_2 Nr. of seq. 2 Alignment length(with gaps) = 14 Alignment score = 0.619048
cGATTAacGATTAa
>Fam_164_14_2 Nr. of seq. 2 Alignment length(with gaps) = 14 Alignment score = 0.714286
CATTgCTCATTgcT
>Fam_165_14_2 Nr. of seq. 2 Alignment length(with gaps) = 14 Alignment score = 0.809524
AGTcCCTAGTcCCT
>Fam_166_13_2 Nr. of seq. 2 Alignment length(with gaps) = 13 Alignment score = 0.653846
gGAGGAgGTaGTT
>Fam_167_13_2 Nr. of seq. 2 Alignment length(with gaps) = 13 Alignment score = 0.705128
CgCTGCTaCGGGg
>Fam_168_13_2 Nr. of seq. 2 Alignment length(with gaps) = 13 Alignment score = 0.602564
TGAGcaAGGaCGc
>Fam_169_13_2 Nr. of seq. 2 Alignment length(with gaps) = 13 Alignment score = 0.705128
CGaTGTGACGcCt
>Fam_170_12_2 Nr. of seq. 2 Alignment length(with gaps) = 12 Alignment score = 0.722222
CacCGGTCGGGT
>Fam_171_12_2 Nr. of seq. 2 Alignment length(with gaps) = 12 Alignment score = 0.666667
GCAGTCGgcGaT
>Fam_172_12_2 Nr. of seq. 2 Alignment length(with gaps) = 12 Alignment score = 0.777778
GTCTCcGTCTCc
>Fam_173_12_2 Nr. of seq. 2 Alignment length(with gaps) = 12 Alignment score = 1.000000
GAAAAGCCTAAC
>Fam_174_12_2 Nr. of seq. 2 Alignment length(with gaps) = 12 Alignment score = 1.000000
GACCACGCCGGT

>Fam_175_12_2 Nr. of seq. 2 Alignment length(with gaps) = 12 Alignment score = 0.777778
GAACaCAAGcCG
>Fam_176_12_2 Nr. of seq. 2 Alignment length(with gaps) = 12 Alignment score = 0.777778
CCATaACCATaA
>Fam_177_12_2 Nr. of seq. 2 Alignment length(with gaps) = 12 Alignment score = 0.777778
TTTACcTTTACc
>Fam_178_11_2 Nr. of seq. 2 Alignment length(with gaps) = 11 Alignment score = 0.696970
AGCGCaGGGgA
>Fam_179_10_2 Nr. of seq. 2 Alignment length(with gaps) = 10 Alignment score = 1.000000
GCGAGCAAAG
>Fam_180_10_2 Nr. of seq. 2 Alignment length(with gaps) = 10 Alignment score = 1.000000
CGACGCAGGC
>Fam_181_10_2 Nr. of seq. 2 Alignment length(with gaps) = 10 Alignment score = 1.000000
CGCGTGTCGT
>Fam_182_297_1 Nr. of seq. 1 Alignment length(with gaps) = 297 Alignment score = 0.000000

GGTTACTGGATAAACACCTGGTATGGTACCTACTGCTACGTTGATAGCACCTGTTGTTGGATTTAAAGTTACTCCTACTGGCCATACGCCTGAGGTTGCTATTGTCGCATT
ACCGCTTACTCCTAAAGTCGCTGGTAATCCATTTACAACATCGTTTAATGCTACATTTGGTATGGCTTCCCCTCCTGTTGATGGTGCTGTACCGCTATCTGCTACTGGATT
TAATACCGCTGTTACAGTGACTGTATTTACTACTGTAGCACATGTTTGTGGCGTTAGTTTATCACACAATTGGTA
>Fam_183_291_1 Nr. of seq. 1 Alignment length(with gaps) = 291 Alignment score = 0.000000

TTTTCTTCTTTTCCTTCTACTATTGCTGGTTCATCTGCTCGATAGTAGATGTCTACCACTGCGTCCAAACTTTCTGAAGTTACTTCTACTTCATCTACTGTGCCTGGAATT
GCTGTGTAGCCTTCTTGCTCTGGGGCTGTAAAGCTTTCCCAACGACCTACGGTCCATGGTCCATATTTCTTCTCGTCGCTGCCTGCTACACTCGTGTACTTCCTTTCCAAG
GTTACTGGCTGTCTTGTTGTTTGAACTTCACCATTTGGCAAGTGTACATTAATTGTTCGCGTGATTGTC
>Fam_184_276_1 Nr. of seq. 1 Alignment length(with gaps) = 276 Alignment score = 0.000000

GGTGACCTCGTCGGCGAAGGCGGAGAGCTGGTCGACCATGGTGTTGATGGTCGTCTTCAGCTCCAGGATCTCGCCGCGGGCGTCCACGGTGATCTTCTGGGACAGGTCGCC
CCGGGCCACGGCGGTGGCCACCTGGGCGATGTTGCGGACCTGGTTGGTCAGGTTGNNCGCCATCAAGTTGACGTTGTCGGTGAGGTCCTTCCAGGTCCCCGNGACGCCCGG
CACGTNGGCCTGGCCGCCGAGCCGCCCGTCGGTGCCGACCTCGCGGGCGACGCG
>Fam_185_261_1 Nr. of seq. 1 Alignment length(with gaps) = 261 Alignment score = 0.000000

TTTATCTGAACCTTTTCCTGTTATTTCAACACTTGTTGTTGCTTCATCATCAGTACGAGCAACTGTAAAGTCTACATAATCAGTTCCATTTACTAATCCAGCTGCAGTTAA
CACAGTTTCAATTTGTGTTTTAACAGTTGAAACATCATTTCCTTTAGCAGCTGAAATTTTTAAATCATCAGCTTTAATAATATCTGCTAAATCTTTTGAAAAGTCTGTTGA
TCAAGTTACAACAAAAGTTACTTTAGAACCATCAACAAC
>Fam_186_258_1 Nr. of seq. 1 Alignment length(with gaps) = 258 Alignment score = 0.000000

GCTGTAAAGTTTTGACCATTGANATCTAATATAATATTTTCAATCGGATTTGGNGCAATATCATCAATCAANGCATTCTGCTTGACCTCTGTACTGCGGTTACCCGCTCGG

TCAACGACAACAAAAGTAAGCTCTTCGCCATGTAAATGCACTTGATANAAATAACCTGAAACATTACCNGCGCTATCNGCTGAACCAGAACCTACGACNTCACCANTAGCA
TTTTTGACTTCAATTTGAGTATTTGCTTCTGCCTGA
>Fam_187_255_1 Nr. of seq. 1 Alignment length(with gaps) = 255 Alignment score = 0.000000

TCACCCAAGACACTGGCAGCTCCAGCAGTGACTTTATCACCAACGACAACACCCTGATTTTCAAAGGTACCGTCGATTTAGGTGACAACAGCACCTTAGCCGTCACCATCA
ACGGCGTGGTCTACACCACCGCCAACGGCTTAGTGATTGATGCACAAGGCAACTGGAGTGTTGACCTGACNGGCACCGTGCTGCCTGACGGCATTTACCCTGTCAGCGCCA
CCGTCACCGACGTGGCCGGCAACAGCAAAACCG
>Fam_188_255_1 Nr. of seq. 1 Alignment length(with gaps) = 255 Alignment score = 0.000000

CTTCGCTTGGCGTTACTGTGAATGAACCATCATTCGTTAANAAATCACCATTACTAATACCNGAATCAGTATCCAATGTGATTGTCGGNGCATTCGGAGCCTGAGTATCTA
AAACAAAATCTAGTGTACTCGAGCCAGACACATTACCCGCAGCATCAGTTTCTCGAACCGTAATTGAGTTGTCGCCTTCAACCACAGCAGGAGGGGTTGTTGACCAACTGC
CATCCGCAGCCTGATATTCAACTGTATTGCCAA
>Fam_189_252_1 Nr. of seq. 1 Alignment length(with gaps) = 252 Alignment score = 0.000000

CTGATTTGGCAGACGGCACGAAAGCCACTCCAACGCAGCCAGCTAAGACAGAAGTGAAGACCGCTGATGGCACTTGGACCTTCAAGTCTTACGACAAGGCTGAAGAGACCA
TCAATGGAGCAGACGCTCACTTTGTTGGTACATGGGACTTTACACCAGCGCCAACCTATAAGGCGACTCACGAGTTCGTTAGTGGTACAGCAGGTAAGGAATTGCCACAAG
AAGTGAAAGCCTTGCTTCCAACCGACAAAC
>Fam_190_243_1 Nr. of seq. 1 Alignment length(with gaps) = 243 Alignment score = 0.000000

TAGTAACCGTAGATAAAACCGCACCTACTGCTACTGCTGGTACAGCACAAACCCTTACTTGTACTATTCCTACTGTAACTTTAGGTGGAACAACTACTTCTACAGGAACTA
ATTTTACACACAGCTGGAGCAATGGTACTACAACGGTAGGAACTACGCCTACAATAACGGTAAGCACAGCAGGTACTTATACCTTAACGGTAACCAACACCTCTAACGGAT
GTACAAGCACCAATAGCGTAA
>Fam_191_243_1 Nr. of seq. 1 Alignment length(with gaps) = 243 Alignment score = 0.000000

ACCTGGTAGGTCAGGCTCGGATCGGCATCGCCGTAGACCTTGGTCTTGGCGTCGGCGATCACATTCAGCAGTGCCTTGGTGATGGTCAGGTTGTTGCCCTGGTAAGCCAGG
TCATAGTTACCGCTGACCAGACCCAGGCCACCCTGCTGAATCCCATACACGCCGACGTTCTCGCCAGACACACGAACCAGGCCGCCGCCATTGAGCACCGCGCCAGCGGTA
TCGCCATTCTTCAGGCCGCTG
>Fam_192_237_1 Nr. of seq. 1 Alignment length(with gaps) = 237 Alignment score = 0.000000

CAGATGCCGATAAGAATGATCCAGCAGGTAAAGATCAGCAAGTCAATGTAGGTGAGACACCGAAGGCAGAAGATTCTATTGGTAACTTACCAGATCTTCCGAAAGGTACAA
CAGTAGCCTTTGAAACTCCAGTTGATACGGCAACACCGGGAGACAAACCAGCAAAAGTTGTTGTGACTTACCCAGATGGTTCAAAAGATACTGTAGATGTGACTGTTAAGG
TTGTCGATCCACGTA
>Fam_193_228_1 Nr. of seq. 1 Alignment length(with gaps) = 228 Alignment score = 0.000000

GCCTCGCCACCTGGTTGCGCACTTGCCACATCCGATGCACCATGACCTGGGTCTACCGGTGTCCCAGCTTTCGCTAACGATGCCGGTTCACCTTGTGGGTTATGGCCTTGT
GCCACCTGTGGGCGACCGTCGTCTTCCCCCCCTTGTGTGCCTTGTGCTCCGCCAGTTTGAGTCGCGGTCGAGATTGGCTCGCCCTGAGTGTGCAGACTCGGTGTCTGAGCA
CCTTTA
>Fam_194_227_1 Nr. of seq. 1 Alignment length(with gaps) = 227 Alignment score = 0.000000

```
CTCGTCACAGGGATGAGAGCAGCGCCGCGTCTGCGGCGCGGAAGAGTCTTTTCAGCCCAAGGACTTGGTCTGGCTGGATTCCTGTGACGAGCACAGGAATGAGGGAGGCGG
GGATGCACGGGCGCTNCCGCAACCTCGTTTGGGGACTGCGCGCCATCTCCTCGATATCCGCGCACCTATGAGAACTATTCCGAGGGACCGGGACCCTCTCTCCCCTCATCC
CTGTG
>Fam_195_219_1  Nr. of seq. 1 Alignment length(with gaps) = 219 Alignment score = 0.000000

TGCTGATGTTTGAGTTATATAATCAATTGAAATATCAGAAGCTAATAAATTTGAGTTTTTTGCTAAAATACTTGCTTGAATTGTTGAAGCATTATTATCTTGTAGATTACC
TAAATTAGTATTAATTAAAACTGAACTTAAATTTTGTTTAACAACTTGAATAGTAAATGTTACATTTACAGAACCTGTATATCTTCCAGAAGCTGATGAATTAACTCT
>Fam_196_219_1  Nr. of seq. 1 Alignment length(with gaps) = 219 Alignment score = 0.000000

TATCTGTTCAGCTTTACCGACACGACGTTTTCGGGTGGCACGCTCGAGGCGGTGATCGGCAAGGGCTATACGGGCGGCAAGAATGTCGATGTCGCCGCGCTCGNTGNANAT
GATGNGTTCGGTGNTTCNGTCTCGCTGAACGCGGCGGGGGACCGTCTGGCGGTCGGGGCGNATCTGGACGACGGGTCCGGCAACNGCGTGANAGATTCGGGCGCGGTG
>Fam_197_207_1  Nr. of seq. 1 Alignment length(with gaps) = 207 Alignment score = 0.000000

CTCGCGTTGCTCGGTACGGCCTACTTGGCATGCTCAGCAACGTTTGACATGTGACCCATCCGTAGGCCGGACCGCAGCGTCAGCGGAGTTCCGGCAATCAGTCGGCACGAT
TTGATTCCGACCGAGTGTTTGATCGCGGGTTTTGTTCTTTGGTGGGTGGTTCACGTTGGGGACCAACGTGCGACAGGTTAGGTGTGGATGCCGTAA
>Fam_198_204_1  Nr. of seq. 1 Alignment length(with gaps) = 204 Alignment score = 0.000000

GTGAAAAAAGGTGCTTCAGGTAACACTGGTGGCTCAAGCAACGGTGGTTCTAACAATAATCAATCAGGAACGAATACGTACTACACNATTAAATCAGGCGATACCTTGAAC
AAAATTGCCGCCCAATATGGCGTGAGTGTTGCTAATTTACGCTCATGGAATGGCATCTCTGGCGATTTAATTTTCGCTGGTCAAAAAATTATC
>Fam_199_198_1  Nr. of seq. 1 Alignment length(with gaps) = 198 Alignment score = 0.000000

TGTCTATGTGGCGGGGGAGACCTNTTCCACCGACTTCCCCACCACGGCGGGAGCGTATGATAGGACGTACAATGGTGGTGGCAGCGATGCGTTCGTGAGCCGCTTGAGCGG
CGACCTGGGCAGCCTGCTCGCCAGTACCTTCCTCGGCGGGAGTGATTGGGACTCNGCCACCGCCCTCGCGCTGGACGGGCAGGGGAN
>Fam_200_193_1  Nr. of seq. 1 Alignment length(with gaps) = 193 Alignment score = 0.000000

GACACAGAGGACACAGCGAGGATAAATACCGAGGTCTCCCACAGCGGCAAGAATACCCTTCCCGCCAGGATTGCCCGTGTTTGGAGTGCGCCAGCCATGCTGCCGCGCCCA
CCACACTCACCGTGGCGGGCGTGTTCCAGCGCTAACTCCGCTGGCAATGGGAAGTCTTGACACGTCTGTATTGCACCACCGA
>Fam_201_189_1  Nr. of seq. 1 Alignment length(with gaps) = 189 Alignment score = 0.000000

ACTTTATCATAATTAAATTTNAAATTATGAATTTTATTTGCCTCTTCAATGAATTCTTGCCTAGTTATTTTCTTTTTTCCATTACACTCAACACAGCCATTACCTCTTAAA
TGGTCTTGAGGTACTTGTTCAAAATCNCCATGTTTTTTACAAGTAATAATTACTTTTGAGTTGCTATTTACATAAACG
>Fam_202_189_1  Nr. of seq. 1 Alignment length(with gaps) = 189 Alignment score = 0.000000

TCAGCGTACCGCCGATAATGGTGGTGCCGCCGGAGTAACTGTTGTCGCCGCTTAACGTCAGCTCGCCNGTACCGGTTTTGACCAGCGAACCGCTGCCGGAAAGCGTATTTT
CCAGCTCGCCCTCGCCGACCTGAAGCACGCCGCTGTTGGCAACAGCTCCGGTACCCAGCGAATCCGCATGGTCAGCCG
>Fam_203_186_1  Nr. of seq. 1 Alignment length(with gaps) = 186 Alignment score = 0.000000
```

GATTCGCCTGTTTGGCAGAGGTGTCGTATGCGTGACTGTGACCGCCATCCGTGACGGCGGCACACANCACGTAGGAGCGCGCTTGCGCGCGATGAAGCTGTCNCGGTGACG
CCCCATCGCGCGCAAGCGCGCTCCTACGACCGCCGATGCGTGCGGGAGTGTCGTGACATCAGACCAGNCACAGCA
>Fam_204_185_1 Nr. of seq. 1 Alignment length(with gaps) = 185 Alignment score = 0.000000

ATGGCGGTGAGGGGGGGATTGACTCGCTGCGCTCGCCCTTCGGGCAGCCCGTTCGCTGCGCGCCCGGTCTGTCCAACTGGCTGCGCCAGTTGTCGAACCCCGGTCGGTGGT
TCTCATCCCCCCTTGGTTTGGGGGATACATATAAGCAAAAAGCCTGTACTTCTGTACAGGCTCTCAACTTGAAG
>Fam_205_182_1 Nr. of seq. 1 Alignment length(with gaps) = 182 Alignment score = 0.000000

GCTGACGCGTCGGGTTGGGACAGAAATGTAGGCCAGGTTGTACCTGGCGGGGTGAATGGACGGACATCGCTGAGTCTTGCGTGACCGGCTGGACGGCTCGCTTTGTTCCAA
AGCATCGGCTGGGTGCCAGGTAATGACCTGGCCTACGTTGGTGGCTTCGCCAGAATTCAGACGTGTCTCTC
>Fam_206_178_1 Nr. of seq. 1 Alignment length(with gaps) = 178 Alignment score = 0.000000

ATGGTGGTGGGGGAAGGATTACTCAGCGCTGCGCGCTTCGCCCTTCGGGTCGTTGCCTGCGGCAACGCTCTCTCGCTGGCGCTCGAGTCGAACCTTGGTCGAAGCTTCTCA
TCCTTCCCCGCATGGGCAGAATATTTGATTGCGGATTCGNTTGAGAGTTCAGGGACTTTTGAAAGTG
>Fam_207_178_1 Nr. of seq. 1 Alignment length(with gaps) = 178 Alignment score = 0.000000

ATTTTATTTCTGCCGAAGAAGGGACTCGCCCCTTCACAGTCTCGCGTCGTGCTCGACTGGGTTCCGGGGCGTCTCGAACTTGCCTCAGCACATCGTGTGCTTCGGTCGCGT
TTCTCGCTTTTTAGGAAAGCTCAAAACGCTTTTCAAGTTCTCGCTTCGAGTCCCCGTTATGTTGTTA
>Fam_208_178_1 Nr. of seq. 1 Alignment length(with gaps) = 178 Alignment score = 0.000000

TCGCGCCCGAGGGCGCTCCTACGGGAGATCCGTTGCGAGGTGAGAGCTTGCCGATCACGTGGCTCGTGCATCGATTTGCAGCGGAGTGGATCACGACTAAACCAGCGACGG
CTGTGAGATGGCGTTGGCCGTGTAGGAGCGCCCTTGGGCGCGATGGGGCGTTACCGGGAAGGCCTCA
>Fam_209_177_1 Nr. of seq. 1 Alignment length(with gaps) = 177 Alignment score = 0.000000

TGTAGCTGGTGACTTTAATGGTGATGGTAAANCTGATATTGCTGCTATGTACGATTATGGCGGNGGTGAAACTAGAATCCATGTATTNACTTCAACTGGAGATTCNTTTAC
CTACACTGGNGCAAATGGTTGGTGGAACACTACTGGTTATGATGCTAACAGAGTTACTGGAAGGGT
>Fam_210_177_1 Nr. of seq. 1 Alignment length(with gaps) = 177 Alignment score = 0.000000

AAAAGTCTTGTCCATTGTTTGTTTCCACCACTATCATACTTTGTCACAAATAGATCTTGGGTTCCACTTAAGGCTTGACCGTCTAAATTTCCGGAAGTCANTCCGGTTATA
TATACATTTCCAAAACTATCAGATGTAATTCCATTNGCCTAAGTAGTTTGAACTCCCGCCACTCCT
>Fam_211_174_1 Nr. of seq. 1 Alignment length(with gaps) = 174 Alignment score = 0.000000

TTAAGTTAGGTAAATGTAAGATTTCATAGAAAGCATTTTGTTGTTCTTTGTTGAATTTGTTATCAGCTTTCGGTGCTTGAGATTCATTTAACTTTTTAGCTTCTGCTAAAA
GGTTAGCGCTTTGGCTTGGGTCATCTTTTAGGCTTTGGATGAAACCATTGCGTTGTTCTTCGT
>Fam_212_174_1 Nr. of seq. 1 Alignment length(with gaps) = 174 Alignment score = 0.000000

AATCCTTCCCCCACCACCATCTATTAATTATTCACCATCTCCTCAGTAAATAACTAAATCAAAAAATAGCACCCATCAGGGGAAGGTAAGAACCTTCGACCAAGGTTCGCG
CCGAGCATAGCGAGACAACGACGCGCAGCGGCGGCCCGAAGGGCGAGGAACAACGTGACGAGT
>Fam_213_173_1 Nr. of seq. 1 Alignment length(with gaps) = 173 Alignment score = 0.000000

TGGTTGCGGGGGCCGGATTTGAACCGACGACCTTCGGGTTATGAGCCCGACGAGCTACCGAGCTGCTCCACCCCGCGTCCGAATTGTACTGCCACCACAAGAGACAGAGCA
GTTGCCCCTTGCTGTCTTCGTTTCCACCGCAGGCGCTAGCGCTTGCCATGGACACAAATAGT
>Fam_214_173_1 Nr. of seq. 1 Alignment length(with gaps) = 173 Alignment score = 0.000000

CCGTCGGCCGAGATCGACGGNAGNCGCTGGCGNNCCGTGCCCTCCGCGCCGTTCGCGTCGACGCTGACCCGGTCGGTCGCGGTGNCNGCTGGTCGTACACGAACACGTCNC
GGNCGCGGTTCGTGTCGACCGGCACGAGGNTCCNGGCGAAGGAGTCGAACGCGATGTAACGG
>Fam_215_173_1 Nr. of seq. 1 Alignment length(with gaps) = 173 Alignment score = 0.000000

CGCCCGGCAGCGCATGCCACGCTATAAACGAGTCAACTCGCACTCGGTGTGAAACCCGGTTTCTGTAGGAGCGGACTTGTCCGCGAAAGGGCCTGTACATTCACAACATTT
CTGCTGCCCAAACGCCCTCTTCGCGAACAAGTTCGCGCCTGCACAAGCGTCCGATTTCGTAG
>Fam_216_169_1 Nr. of seq. 1 Alignment length(with gaps) = 169 Alignment score = 0.000000

CGTAGGCCAGGTTCCACCTGGCGATGGTTGCTTGCCGGTGAACGTCCCCTTGCGATGGCTTATAAATCACGCCTCGCTTTGTTCCAAAGCATCGGCGCGGATGCCATGTGT
AACATGGCCTACGAGTGGCGATTGCAAACTGCGAAATGCACTTTGCAATGGTTTCCAA
>Fam_217_168_1 Nr. of seq. 1 Alignment length(with gaps) = 168 Alignment score = 0.000000

CAACTGAGCAGTGCCACCAACAACACGTCTGAAACGCTGGCCGCCACACCGAAAGCGGTCAAGGCTGCATACGACCTGGCAGCCGGCAAGGCGCCTGCCAGCCACACTCAC
CCGTGGAGTCAGATAACGGGAGTGCCTGCGGCTTCACTGACGGCAAAAGGCACCGTA
>Fam_218_162_1 Nr. of seq. 1 Alignment length(with gaps) = 162 Alignment score = 0.000000

AAAACACAAAATCACGCCAACAAGCCATTAAGGATCTAAACGAATTCTTAAAAAACAATCCCAATGACGCCCAGGCCTCTAAAACTTTAGCTCAAGCTAATAAAATACAAC
ACCTGGAGGACCTTAAATCTAAGGTTCATTCAATAAAACCCATTGATCTTG
>Fam_219_159_1 Nr. of seq. 1 Alignment length(with gaps) = 159 Alignment score = 0.000000

TTTAGCTTCTTGAACCTCTTGTGCCTCTTGTGATGTATCACTTAAATTATTTGCACTTGCTTCTTCTTTTATCGCTGCTTGTTGTGCTTTCAATGCCACTGCTTTTGGTTC
TTCATTTGATACAGCTGCACTTTTATCTGTTTCTGCTTGTGCTTCTTT
>Fam_220_156_1 Nr. of seq. 1 Alignment length(with gaps) = 156 Alignment score = 0.000000

CAGACCGACGTGTTCGCCGTCGACNACAACGGCGCGTTGAACGTNGCNTGGGTNGTCAGCGCCGANCGCTGGAACGGNCCGATTCCGATCAGCCCNGCGGGGCTCTTCCCG
NCCGGCGCGGCGGTCGCGGCATCGAACCAGTTCGGCATCCCGAAT
>Fam_221_156_1 Nr. of seq. 1 Alignment length(with gaps) = 156 Alignment score = 0.000000

AATTTAGTGGTTGATGGAACGACTAACTTAAACAATTCTTTATCAGTAAATAATCAGTCTCCAGCCAATTTAACGGGAACATTAAATGTAGATGGTGCTACAACTTTAAAT
AATACATTTACCGTAGCAAATCAATCGCTATCACGTTTAACTGGC
>Fam_222_154_1 Nr. of seq. 1 Alignment length(with gaps) = 154 Alignment score = 0.000000

ACCGGCTGATGCCTGTACTATGTGAATGATTGAACGAGTCAGTACTCGCGTTTGCCCCGCTCCTGCGGCCGCCGGATGAAAAGCCTGCCATTCGTCGGAGAGAGAGGGCTG
GCGCGTCAAACACGCTGAGGTTACACTGGTACGGGAATGGAGC
>Fam_223_150_1 Nr. of seq. 1 Alignment length(with gaps) = 150 Alignment score = 0.000000

TGGGGCGGCATCNCCGACNCGCATGCNCCANCNCGACNACNCCGTCGCGGGTGGCGNCNACGNCACCNACGGCGACGACGCCGTCGTCGGCGGCANCGTCGTCGACGCGCC
GGTGGCGNNGCCGGCCAGGTCANCNGCANCGCCGGCGCG
>Fam_224_148_1 Nr. of seq. 1 Alignment length(with gaps) = 148 Alignment score = 0.000000

CATGTTCCACATGGCATCCACGCCGATGCTTTGGAACAAAGCGAGTCGTGTTATTTGAGTGCTCACGCAAGGTCGATGATGGTCGTCCAGTCACCCCGCCAGGTAGAACCT
GGCCTACGTGGTTGGCTGCGTTGATTGGGTCGTAGGC
>Fam_225_145_1 Nr. of seq. 1 Alignment length(with gaps) = 145 Alignment score = 0.000000

CGCTGGTCTTCTGACGGAAATGGCAGCAGCGGATCACATCCTGGCGGCCAGGGTCGGCCTGACTGCTTACAGCACGCACGGTCATTGCGAGAGCGTTGTAACCGGGGACAC
CTGGCTAGCCTGCCGGGTCGTGAGCCATTCGCGG
>Fam_226_143_1 Nr. of seq. 1 Alignment length(with gaps) = 143 Alignment score = 0.000000

ATAGGCTATTTTACTTGCCATTTTGGGCTTGAGCAGTGCGCGGCGCCGTCACGTACTGGGTGTACGCTCCGTTGCCTGTGCGCTGGTCGCACCCAAACTGTCTGCGACAAT
AACGCCTATTGGATAAGTTGGATTTTTACACA
>Fam_227_141_1 Nr. of seq. 1 Alignment length(with gaps) = 141 Alignment score = 0.000000

TGCCGTTGTCGGCGNACTGGACGACNNGCGCGCTGTCNGCNGTGGACATCCGGTCGACGCCGAGCACCTTGCCCGAGTGGNGGTTGCGGATGCGGNACCAGCCGTCGCCGT
TGTCGATCAGCTGCCAGAGGTGGTCGTCGG
>Fam_228_141_1 Nr. of seq. 1 Alignment length(with gaps) = 141 Alignment score = 0.000000

GCAGAAAGCAGCCAACCAGAAGTTCATGTCTCACCAACAGAAGAAGAAAATCCAGATGAAAGTGAAACACTAGGCACGATTGAACCAATCATACCTGAAAAACCAAGTGTG
ACAACTGAAGAGAATGGCACAACAGAAACT
>Fam_229_141_1 Nr. of seq. 1 Alignment length(with gaps) = 141 Alignment score = 0.000000

GGATTCGAACCTCCGACCGCTCGGTTCGTAGCCGAGTACTCTATCCAGCTGAGCTACGGATGCATCGGGAAACTTACTTTACTGCAGATTTTTTGATACCGCTACTAAAGC
CGTATCAAGTAAGAGATGGTGCATCCGGGA
>Fam_230_139_1 Nr. of seq. 1 Alignment length(with gaps) = 139 Alignment score = 0.000000

ACAACTTAAAAGTNTNAAGAATCTGNNTTTGAATCACAATNAANTNACTACTCTTCCGAAAGAAGTTANGACANTTGNAAAATTTACAAAAGTTGGATTTANGANNCAATA
AACTTACGACNCTTCCNAAAGAGATAAN
>Fam_231_138_1 Nr. of seq. 1 Alignment length(with gaps) = 138 Alignment score = 0.000000

GAGCGCGCCGGAGGCGCACGCTCCCAGTGCTGCCGGTGAGTGAGTCGGCGGCGTGCTCGCAGGGTATGCAGACCCGCCCCTACCCTGCCCTGCACCCGCTGGGGGCAGGAA
CCGGCGGCGCCGAATGGCGCAGGTGGG
>Fam_232_138_1 Nr. of seq. 1 Alignment length(with gaps) = 138 Alignment score = 0.000000

AGGAGCGAACTTGTTCGCGAAGGCAGTGTTTTGAACGCCAAATCTTCTGCGGCTGTACGGGCCCTTTCGCGAACAAGCGAAGCGTCGCCCGGTTCGCTCCTACATTTTTGC
CGTACGCTCAAGCATGGGGGCTTTTGT
>Fam_233_138_1 Nr. of seq. 1 Alignment length(with gaps) = 138 Alignment score = 0.000000

CAGTTAGAGCCTGCCGCAGAATCGGAAGTTGAGCCAGAGCTTGAGCTGGCAAGTGAGCCAGNNGAGGAAGAAGCCTTTACCGAGCTTGATGAGCTCGATTTGCCTGAGTAC
ACCGAAGAAGACGCCTTGGCGGATGCG
>Fam_234_134_1 Nr. of seq. 1 Alignment length(with gaps) = 134 Alignment score = 0.000000

GGATTCGAACCTCCGACCGCCTGGTTCGTAGCCAGGTACTCTATCCAGCTGAGCTAAGGACGCGCAGTTTTCGATATCAGTATTACCAATATCAGGAGTTCACAAGGAAAC
CCTAAAGAGTGGCGCGTCCTGGA
>Fam_235_133_1 Nr. of seq. 1 Alignment length(with gaps) = 133 Alignment score = 0.000000

AAGAACTTCTAGCTTTTCTCTTTCGTTCAAAGAGAAGCAGCTGTTCGCAGTTTAATCAAAACCACATAAAGCTTTTAACTTTACTCTTTGATTTAAAGAGTGATAAATGTT
TACAGTTTAATTAAAACTGCAT
>Fam_236_132_1 Nr. of seq. 1 Alignment length(with gaps) = 132 Alignment score = 0.000000

GCGCCCGCGAGGCGCAGACCACGCTGGTCACCGCCTCCTCCGATGCCGCCAACCAGATCAAGGCGCTCGCCGCCGACGTGCAGCGCTCGCTCTCGCTGGCCGGCNCCACGA
CCGCCGAGACCATCACGACCG
>Fam_237_132_1 Nr. of seq. 1 Alignment length(with gaps) = 132 Alignment score = 0.000000

TTGGGCGGATACTCGTGGATAGCTCCACTCCGCACCCCCCTCCGCGCCGATCCGCGTCATTCGCGTGTATCCGCGTTCCGCATCACCCGTGCCTACTCCTGGATGCGCCGG
ATGCACCCGGAACAACGCGGC
>Fam_238_132_1 Nr. of seq. 1 Alignment length(with gaps) = 132 Alignment score = 0.000000

AATCTTGGTTTTATCTCAAATTTGATGGGAAAATAGCTGAAAAAGAATGGGTCTACGATTCTCATAGTCAAGCTTGGTACTACTTCAAATCCGGTGGTTACATGACAGCCA
ATGAATGGATTTGGGATAAGG
>Fam_239_132_1 Nr. of seq. 1 Alignment length(with gaps) = 132 Alignment score = 0.000000

TCTTCTTCAAGAGCTTCNGTANTTTCAACGTCAGATGCNACTGCTTCTTCAACAGTATNTTCTGCTATTCCTTCAGATTCTGTTGGCTCTTCAAGTTCAGAAACTTTTGTC
TCTTCTACTTCTGACTCAAGC
>Fam_240_129_1 Nr. of seq. 1 Alignment length(with gaps) = 129 Alignment score = 0.000000

TTCAGCCCCGACGGCAAGCATCNTGGCCACCGGCAGCTCCGATCAGACCGTGCGGCTGTGGGAGACCACCACCGGCCAATGCCTGAGAATCCTGCAGGGCNACATGCCAAC
GGCATCTAGTCGGTGGCC
>Fam_241_129_1 Nr. of seq. 1 Alignment length(with gaps) = 129 Alignment score = 0.000000

GCATCTTCTTCGTCGAACTCTGGCAGTTCCAGTTCGTCGAAGTTGAATTCTTCTTCCGCGTCAGCGCTNGGCGCTGCNGCTTGNACTTCAGCTTCTGGTAGCTCAGGCTCA
TCGGCCATNGATGCCAAT
>Fam_242_127_1 Nr. of seq. 1 Alignment length(with gaps) = 127 Alignment score = 0.000000

TTGAATAAATAGTTTTCACCCCAGAGCTTAACTGACCNGCGACTGCGCTGCTGTCACCGCCAGAGATCGCNATCCCCCCAAGTAACCACCGAGCCATCATCTTTTACGGCC
GCAAAGGCANTAATAG
>Fam_243_126_1 Nr. of seq. 1 Alignment length(with gaps) = 126 Alignment score = 0.000000

GTCGGCGGGACGCCTGAACGAAGCAATCGCCCTGTACGAGCAGACCCTCGCCGATCGCGAGCGGCTGTTGGGAGACGAACACCCGAGCACCCTGACCTCGCGAAACAACCT
CGCCNGCGCCTACAN
>Fam_244_126_1 Nr. of seq. 1 Alignment length(with gaps) = 126 Alignment score = 0.000000

CGATGGCGGCGGCCCAGCACGGCCTCCTTAATCGCCAGCGCTTCTTCGTAGTAGCGGCGGGCGGTGGGGTAGTCGCCTGCGGCTTGCGCGACCTTCCCCAGTGCGTGCAGG
GTGACGGCGGTCTCA
>Fam_245_126_1 Nr. of seq. 1 Alignment length(with gaps) = 126 Alignment score = 0.000000

AAAGAACTAGACAATTCCTTGCTGAAAGCCATCGCTCAAAATGCCAATCGGTTTAATCCTCAAGATATCGCCAATACCCTCTGGGCATTAGCTACCATGGGGATCAACTGG
CGTGATATTCAAGAA
>Fam_246_126_1 Nr. of seq. 1 Alignment length(with gaps) = 126 Alignment score = 0.000000

TAAAGCCCATAATGCGTTAGCAATACCTTGGGGGTTGAATCGCTCAGCGTTGCGGCGAACAGCNCCAAGCAAGCGATCACTTAATCNTTGGTCTTCCAACTCTCGCCACCT
CATACCCATCGTGGC
>Fam_247_126_1 Nr. of seq. 1 Alignment length(with gaps) = 126 Alignment score = 0.000000

AGCGGCTCGGCCTCGGCGTACTCGCCCCGCTCGGAGTACAGGAGGGCCAAGTTGTCGAGGCTCTTCGCCATCTCCGGGTGCTCCGGCCCGAAGGCTTTCTCCCGGATAGCC
AAAGCGCGCCTGAAA
>Fam_248_126_1 Nr. of seq. 1 Alignment length(with gaps) = 126 Alignment score = 0.000000

GCCTCCCGGTAGCGGCCCTGATCGACGTACAGAAAAGCCAGGTTGTTGAGGGTTTTGGCCACCTCGGGGTGATCAGGACCGAGGACTTTTTGGTGAATGGCCAGGGCCCGC
TCGTACAGCGGCTCC
>Fam_249_126_1 Nr. of seq. 1 Alignment length(with gaps) = 126 Alignment score = 0.000000

CCGTCCGGGGCAAACGCCACCGAGCGGATCCAGCCGGTGTGCCCNNGGAGCGTGGCGAGGCACCTTCCCGTGGCCGCATCCCAGAGTTTGACGGTGCNGTCCTGNCCGGCG
CTTGCGAGGAGGCTC
>Fam_250_126_1 Nr. of seq. 1 Alignment length(with gaps) = 126 Alignment score = 0.000000

CCACCGCCGACAGCCGTGCTCCCGCCAACAGAACCCGAACCACCCAACGACTCACTAATGCCACCACCAACATGAGAACCAACACCCACACCAGTGCTCACACCCCCACCA
ACACCGGTATGAGCA
>Fam_251_126_1 Nr. of seq. 1 Alignment length(with gaps) = 126 Alignment score = 0.000000

AGGACCATCCCGACACGCTCACTTCCGCCNGCAACCTCGCCACTANCCTGAACTCCTTGGGGGAGCATGCGGAGGCNCGTCGCATGCAGGAGGATGTTCTTGCGCGTCGTG
GTCTTGTCCTGGGTC
>Fam_252_126_1 Nr. of seq. 1 Alignment length(with gaps) = 126 Alignment score = 0.000000

GGTGGTCGTCGCCGAGGGTGCGGCGTNGGCGGGTGAGGGTGTCNTCGANGATGCGGNGGGCTTCGGTGTGCTCGCCGAGGTTGTGCAGGGNGNCGGCGAGGTTGTGGGCGG
ACTGGAGGGTGTGGG
>Fam_253_123_1 Nr. of seq. 1 Alignment length(with gaps) = 123 Alignment score = 0.000000

TAAAAGTAACTGATTTAGTACCAGGCAGTTACCAATTTGTCGAAACAAGTGCTCCAACAGGCTACAAGTTAGACAACAGTCCAGTAAGTTTTGAAGTTGTCGCTGGTGAGA
CAGACCAAGTAG
>Fam_254_123_1 Nr. of seq. 1 Alignment length(with gaps) = 123 Alignment score = 0.000000

GTGAACCACAGGGCGCCGTCCGGCCCCGCGGTGATCCCGANCGGCCCGGCCNCNGGGGTCGGCAGCGGGTACGCGGTGATGGCCGTCGGGGGTGATCCGGCCGATNGCGNT
GCCCCGGTCTCG
>Fam_255_123_1 Nr. of seq. 1 Alignment length(with gaps) = 123 Alignment score = 0.000000

TCTTTCAAATTGCAGGGGTGTTGTCGGCTCTCGCTTACCCGAATCACTTACCAGTGTAAGCTCATCGGGATGCACTCTTTTGCCGCCTACCTGTAATCCGAAATCTATAGG
GGATACTTATTT
>Fam_256_121_1 Nr. of seq. 1 Alignment length(with gaps) = 121 Alignment score = 0.000000

AAGTAGGCCGTATCAAGGACCAAAGGGACGCAGCTACGGCATCACCCTGAACCCCACAACACTCTCGCCATCCTTCCGGAACTCCACTTCGTTCCGGTCCGGCCTACAAAG
CTTTCCAACC
>Fam_257_116_1 Nr. of seq. 1 Alignment length(with gaps) = 116 Alignment score = 0.000000

CTCTCGCGGGTGGACGGGCGGGTTGAGGACGGGCCTGGCTAGCGCGCCGGGGGTGGCGGGACTGGAGGCGGTGTTATCCATACCATCAGCAAACCGTGCTGCAAGCGCGGC
AAGCG
>Fam_258_116_1 Nr. of seq. 1 Alignment length(with gaps) = 116 Alignment score = 0.000000

CCGCTTTCTTTCGGGTATAGCGTCGTGGACAGTCATTCATCCTGCCGCCCTGTAAAAGCAAAAACCCGCCAGCTTACACTGACGGGTTTTATGAAGAGATGAAACTGACCG
ATAAG
>Fam_259_115_1 Nr. of seq. 1 Alignment length(with gaps) = 115 Alignment score = 0.000000

AGTTCCCACAGATCATTAAGTCATTTTTTCGATTCTAAAATATTCGTTTTTTATAATTTAGTTTGTAATAGTTCCTAGAATTTCAAAACTAATTTATAAAATCTAAATTTG
TGAG
>Fam_260_115_1 Nr. of seq. 1 Alignment length(with gaps) = 115 Alignment score = 0.000000

AGGGCGAATAACCGCTCGCGGTCATCCGCCGCTCATCCGCGACATCGGCGGATAACGCCCATGGCGTTATTCGCCCTACACACCCCGAAAAAGCCGGACTGCGCCCCAGCT
CCGT
>Fam_261_114_1 Nr. of seq. 1 Alignment length(with gaps) = 114 Alignment score = 0.000000

ACCAAGGTGGGCGACGGCANGGCGCTGGCCGCCATGNTGGGCAAGGCCAACNTNTTCACCCANGTCGGCNACGGCGANNCCTGGCGCTGNNNGCCGGGCNAGGCCAACNTG
NTC
>Fam_262_114_1 Nr. of seq. 1 Alignment length(with gaps) = 114 Alignment score = 0.000000

TGCTTGCCGTCCACGTCGAGCCGTCCCGTTGCCGACAGCTGGTCGGCCCGGAGCGCGGCGCCCGCGCTCACGGACAACGCGTCGCCGCTGGCGGCCGAACCCAGCGTGACG
GCC
>Fam_263_114_1 Nr. of seq. 1 Alignment length(with gaps) = 114 Alignment score = 0.000000

AAGAAAGCCGCGGTCGCTGCTGCTATTGCTCGTGCAAAAGCGCGTAAAGCGCAACAAGAGACTGAATCTCAACCTGTTGAAGAAACAGCTTCTCAAGAGCCAGCCGAAGAT
CCG
>Fam_264_113_1 Nr. of seq. 1 Alignment length(with gaps) = 113 Alignment score = 0.000000

CACCCCGTAGGTCGGATAAGGCGGTTACGCCGCATCCGACATCCAACGCCCGAGCCGGTTGCCTGATGCGACGCTGGCGCGTCTTATCAGGCCTACACCGCTGTGAAGTGC
TC
>Fam_265_113_1 Nr. of seq. 1 Alignment length(with gaps) = 113 Alignment score = 0.000000

CAAAAACCCGCCGAAGCGGGATTTTGTGGAATGAGTGGCGTTGACCGGTAAGCCGCTTTCTTTTATAAAAAGTGTCGTGGACAGTCATTCCCCTCACGCCCTATCCTCCAA
AG
>Fam_266_112_1 Nr. of seq. 1 Alignment length(with gaps) = 112 Alignment score = 0.000000

```
GCGGGGACACGGATAGACGCAGATTAGGCGGATGACCGCGGATAGGTTGTCTGTAGAGCAGGTTGAAGGATGGGCCATTGGCATATCCACCAGCGGCCGTGGGCAGCATTT
G
>Fam_267_111_1  Nr. of seq. 1 Alignment length(with gaps) = 111 Alignment score = 0.000000

TGGAGCAGGGCGGTGATTACCGGCTCTCTCAGCTCTCCAGCATCAGCCAGCACTGATCCCGCCTGCACCCGCACGTACGGGTCGATGTCGGGGTTGGAGTCGGTGGCGAGC
>Fam_268_111_1  Nr. of seq. 1 Alignment length(with gaps) = 111 Alignment score = 0.000000

GTGGACCTGGCCACGGATGGTGCACTGCTCAGGCTTCGGCCGGATCGGGCGGCAGGCATTGCCNTTCCGCACCTCGGTNCCCCGCGGGCAGACACAATCGCCATCCGCATC
>Fam_269_108_1  Nr. of seq. 1 Alignment length(with gaps) = 108 Alignment score = 0.000000
 CGCGACGATGCTCACTTTGCGCCGGCGCATCCTGCCAGGGCTCGTTCAGACACCTACGCTGGTCTTGAGCCATTTGTTGATCTGAATGCGCCGACGCCGTCCGAGTTG
>Fam_270_108_1  Nr. of seq. 1 Alignment length(with gaps) = 108 Alignment score = 0.000000
 AAAAAACAAGCTTTTTATTGGTACACAAAAGCTGCCGAACAGGGATATGCAGNAGCACAGTTTAATCTTGGTCTTATGTATTATAATGGAGATGGAATACTGGCTGAC
>Fam_271_105_1  Nr. of seq. 1 Alignment length(with gaps) = 105 Alignment score = 0.000000
 GGTAACAATGCCGATCCTGACGATGACAATGACAGCGTCAATGATGCACAAGATGCCTTCCCACTCGACCCAAATGAAACTGTCGACACCGACAAAGACGGTATC
>Fam_272_105_1  Nr. of seq. 1 Alignment length(with gaps) = 105 Alignment score = 0.000000
 CATTAACGGCGGCCAGCTTCACAGCTTAAGCAGCAACATCGCCAACTACTTTGGGGGNGATGCTTCTGTGGGTGATGACGGTACGTTTACCGGCCCGACCTATAA
>Fam_273_103_1  Nr. of seq. 1 Alignment length(with gaps) = 103 Alignment score = 0.000000
 GTAGGAATTCTTACGAAGCTAAATTAGAATATATGAGTATTTAAAATTCGTAATGTGACTAAAACTGTGGGAACTACCGCAAATTTGGGTTTATTGTTAAGTT
>Fam_274_102_1  Nr. of seq. 1 Alignment length(with gaps) = 102 Alignment score = 0.000000
 CGCGGATGTCGGGTCCCGTGCGGGGAGACATACCGTCTTGGCCGGCCAGCGTCCATGGCGTACGGCGGCGTGCCTGTCCCGGCGGGGACAGGCACACTCTGC
>Fam_275_101_1  Nr. of seq. 1 Alignment length(with gaps) = 101 Alignment score = 0.000000
 AATTTTGGTATGTACTCAATTTAGGCTTCACTTTAGTTTCTCCTTAGTGAAAACTGCTCTACTATAACCCTATGTATGTACCATAATTCTTTGATGATTAG
>Fam_276_99_1  Nr. of seq. 1 Alignment length(with gaps) = 99 Alignment score = 0.000000
 CTCGAAGAACTGGGCCTGTCACCAGAAGAGATTGCCCAACTTGAAGGCCAGGCGCCCGCTGCTGCGGCCAGCGCCGAACCGAACCTGAAACCCTTCTCC
>Fam_277_99_1  Nr. of seq. 1 Alignment length(with gaps) = 99 Alignment score = 0.000000
 GCTTCCCAAGCAATCGGTGAGGGTTCTTATCGGACAATCGCTTTTGTGAGCGAGCTTTACTAGCAATACGTGCGGGTATAGTTGAATGGTACAACTATT
>Fam_278_98_1  Nr. of seq. 1 Alignment length(with gaps) = 98 Alignment score = 0.000000
 GTGATTTGTGGGAGTTCCCACATTTTAGGAATCGAGATTACAACTTTTTTTAGAAAAATGAACCATGGATTTCCTTACGTCGAACTCACGTTAAAATC
>Fam_279_97_1  Nr. of seq. 1 Alignment length(with gaps) = 97 Alignment score = 0.000000
 GAAGCCGGTCACACAGTTCGGCGCGCAGGCGTAACGCGCAGATCGTTCGCCACGCAGCACTGACGCATGGCCACGCACGCGAAGCGCTCCATGCGAT
>Fam_280_96_1  Nr. of seq. 1 Alignment length(with gaps) = 96 Alignment score = 0.000000
 CAGGTGGGGGGCGAGGGCGGCCAGCGCGTTGCTGCGCCACACCCCATCGGCAATCTCACGGGCGGCGGCGAGGGCCTCGGGCAGCAGCGCCGGCGG
>Fam_281_95_1  Nr. of seq. 1 Alignment length(with gaps) = 95 Alignment score = 0.000000
 TTATGGTGCGGGCTATATATTTTAGTTTCAATAGGTTTATTAACTTTTTCAAAGTAAGAATAAGACCAAAAGAAAAAATCACAATCAACAAGTGA
>Fam_282_93_1  Nr. of seq. 1 Alignment length(with gaps) = 93 Alignment score = 0.000000
 GAAACAACGTGGGAGTTCCCACAATTTCAAGGTCTAACCCTAAAATCAAAACGGCAGAAGCCACTACAATTTTTTAACAACAAGCTTCAATTA
>Fam_283_93_1  Nr. of seq. 1 Alignment length(with gaps) = 93 Alignment score = 0.000000
```

```
 CAGTCAAACCACTGCCCAGGTCAGCAGAAGATTCGGAGCCAGTTCTATCGGCTGGTTTATCCGAAACGAAACTGCTTAAGCTGTCCGGATTAG
>Fam_284_92_1  Nr. of seq. 1 Alignment length(with gaps) = 92 Alignment score = 0.000000
 AAAAAACACGTCATTCAAAAAAGAAAATAAACTAATTAAGGATGTCGTTAATAAAGAGGTTTGGGCGGAGCCCATTATATTCATGAACTTAG
>Fam_285_90_1  Nr. of seq. 1 Alignment length(with gaps) = 90 Alignment score = 0.000000
 GCCTTGCTGGCGGCAGTGGAGCTGGCAAACAGTGAATCATGGTTTTTGAGGAAAGNGTTCACAACGGAGTTGCGTGTCATTGCCTCGAAT
>Fam_286_90_1  Nr. of seq. 1 Alignment length(with gaps) = 90 Alignment score = 0.000000
 GCCATCAGCAATGATGGCCTTTTTGTTTACAGAATTTGAACCTNGGGTTCCTTATTCTAAGCGCAGCTCCTGCGCCATGTTTCATGGAAA
>Fam_287_90_1  Nr. of seq. 1 Alignment length(with gaps) = 90 Alignment score = 0.000000
 TTCTCTTTATTAGCCATTTGTTCTTGAGGTGTTACGACCATGTCGTTCACAATGCTTGGCATCTGTTTTTCAGAGATTACAACCTTATCG
>Fam_288_89_1  Nr. of seq. 1 Alignment length(with gaps) = 89 Alignment score = 0.000000
 TCCGTGCCGCTGTGGTGCACCTACCACCCTGCCACCGGAAACACGCCGGAGGAGGAAAGCATGCCGCAGCGCTACATCCCTCTATGCCA
>Fam_289_88_1  Nr. of seq. 1 Alignment length(with gaps) = 88 Alignment score = 0.000000
 GGTGCGAATGCAGGTGCATTCTGGCAAACCCGACAGACGCAAAGTGCGCCGGAGGCGCGCGCTCCCAGTGCTGCGCNGGGTGGTGATC
>Fam_290_88_1  Nr. of seq. 1 Alignment length(with gaps) = 88 Alignment score = 0.000000
 CAACGCGCCGCCATCCGGGGGTATTCCCTGCTCGCGCTGCGCTTAGCAGGGCTGCGGTTCCGTGCCGTTAGGTAGCCCGGATAAGGCG
>Fam_291_88_1  Nr. of seq. 1 Alignment length(with gaps) = 88 Alignment score = 0.000000
 CGGTCAGGTGTACACCGAACTTGTGNGAGCTTGCTCGCGAAGAGGCCCGTGCAGTCGCTGGATGTCTGCTGGGTAGCTGCTCACCTGT
>Fam_292_86_1  Nr. of seq. 1 Alignment length(with gaps) = 86 Alignment score = 0.000000
 TTCTCCATTTGCAGAAGTGTAGAAGTAACAATCCGTCAAAGGAGACAGTGAAGGAAAGCCATAAGGCATATACGGTTCACTGCCCA
>Fam_293_84_1  Nr. of seq. 1 Alignment length(with gaps) = 84 Alignment score = 0.000000
 ATTATAAGATTCTATCTTTGTAGTGCTATGTGCAACTACAGCTTTTAAAATATCTAAATTGCCTTTATCTATAGCCCTATCAAT
>Fam_294_82_1  Nr. of seq. 1 Alignment length(with gaps) = 82 Alignment score = 0.000000
 TGGGACACGGATGCACGCGGATTGGGCGGATGTGCGCGGATATGGCAGAAGTACCGGTCATCCGCAGGGTGGGTATTACCGG
>Fam_295_81_1  Nr. of seq. 1 Alignment length(with gaps) = 81 Alignment score = 0.000000
 TGTTCGGCCGGCGCCTGCTCGGTCTGCTGCTGCTGGTCACGCTTCTTCCTGCGCGGCTGCTGTTCCTCTGCGGGTGCTGCC
>Fam_296_81_1  Nr. of seq. 1 Alignment length(with gaps) = 81 Alignment score = 0.000000
 GTAACAACACATGCAAACGGCCAAGTATCATACGGAGCTCGCCCGACACAAAACAAGCCAAGCAAAACAAATGCATACAAC
>Fam_297_79_1  Nr. of seq. 1 Alignment length(with gaps) = 79 Alignment score = 0.000000
 TGGATGTAAGTAATTTTGATACCAGTTCAGTAACTAATATGTATNACATGTTTAAAGATTGTAGAAATCTTGAAGAAGC
>Fam_298_78_1  Nr. of seq. 1 Alignment length(with gaps) = 78 Alignment score = 0.000000
 GTTTGTAATCCNCTACAATCANCAAACATATTACTCATATNTGTCACTTGACTCGTATCAAAATTNGATACATCTAAT
>Fam_299_78_1  Nr. of seq. 1 Alignment length(with gaps) = 78 Alignment score = 0.000000
 ACGGTGATGTTGGCTGGTGTGGTGGCGGCCGGGATGTTCAACGACGGCAACGTCAACCCGGGCAGGCTGAAGGCGCCG
>Fam_300_77_1  Nr. of seq. 1 Alignment length(with gaps) = 77 Alignment score = 0.000000
 TACGAAAGCGATGATTTTTACAACTCCGTTGAAAGCAAAAGACTACGCTACGCTTGTCTTTTTGCAACATCGCTTTT
>Fam_301_77_1  Nr. of seq. 1 Alignment length(with gaps) = 77 Alignment score = 0.000000
 GATGACCTGCGCCGACGACGATGCAGAGCGTAGCGATGAGGTGGGGGCACCACCCGCTTGCGGGGGAGAGTGGCGCT
>Fam_302_75_1  Nr. of seq. 1 Alignment length(with gaps) = 75 Alignment score = 0.000000
```

```
CCCAACGGGATTGCCGGAAGTGAGTAGCCATCCGGGAACACCGTAAACGGGCCTAACCCTCCGCCCACATCAATA
>Fam_303_74_1  Nr. of seq. 1 Alignment length(with gaps) = 74 Alignment score = 0.000000
GTTGGTAATTATAAGCTAAAATACAATTTTGAAAGCAATTCACAACNNAATATNTATNTANTTANNATNTTNNT
>Fam_304_74_1  Nr. of seq. 1 Alignment length(with gaps) = 74 Alignment score = 0.000000
GTTTCAACCCTCGCCCGGCATGGAAGCCGGGCGCGACGANNACNTNCCNNANGTNNNCCNTNGGCNATNGNNNN
>Fam_305_73_1  Nr. of seq. 1 Alignment length(with gaps) = 73 Alignment score = 0.000000
TGTCTGTAGTTCCGACCTAAGGTTTTACTGTTAAAATCTTGTGATTTGTGGTAGTTCCCACGTTTTTCAAATC
>Fam_306_73_1  Nr. of seq. 1 Alignment length(with gaps) = 73 Alignment score = 0.000000
CCGCGGAGCGGTGACAGTTGTCAGCCTCGGGTTTTCAACCCGAGGTCTCGGTTGCAAAACCGCGTCCTCCAAG
>Fam_307_72_1  Nr. of seq. 1 Alignment length(with gaps) = 72 Alignment score = 0.000000
TGCTTTAGGTGGTGNNGCTGGTNNTGCAGTTGGTAAAAGCATNGGCGGTACANAAGGTGNNNCAATTGGTGC
>Fam_308_72_1  Nr. of seq. 1 Alignment length(with gaps) = 72 Alignment score = 0.000000
GCCAGCGCCGTATCAGCAGCCGGCTTACGATCCGCATGCCGGTCAACCTGCGCCGCAGGCCTATCAGCCTGA
>Fam_309_72_1  Nr. of seq. 1 Alignment length(with gaps) = 72 Alignment score = 0.000000
GTCAGTAAAAAGTGACTGAATAAGGAGCATTTTATCGGCAAATAAAAGAAAGATCAGCTTGCTGAACTTTCT
>Fam_310_72_1  Nr. of seq. 1 Alignment length(with gaps) = 72 Alignment score = 0.000000
TGCGATTGAATAGTGAGCATTTTAGTTGTAGATAAAAGAACCGTCAGCGCAGCTGACAGTTCTGTCAGTAAT
>Fam_311_72_1  Nr. of seq. 1 Alignment length(with gaps) = 72 Alignment score = 0.000000
ATAGGCGGAACATTGATTGGCCCCACCAACGCCCCCGAACTACTCACACCCAAACCGATGGCGGGAACAGTA
>Fam_312_70_1  Nr. of seq. 1 Alignment length(with gaps) = 70 Alignment score = 0.000000
TAAGATATTACTATCAAGTAGTGAATTATATTGAACGATATATCAATAATTGGCAGTAAATTATATTAAA
>Fam_313_70_1  Nr. of seq. 1 Alignment length(with gaps) = 70 Alignment score = 0.000000
GTTTAAAAAAGCGAATTTTATCGACACATAGCNAGAANGTGTCGATGCCATAAGCATATTCCGGCAACAT
>Fam_314_69_1  Nr. of seq. 1 Alignment length(with gaps) = 69 Alignment score = 0.000000
CCACTGCCACCGTACAGCAGCAGGCCACCAAGGCCGTGACCGAACCGGCCAAGCAGGCGGCCAGCAGCG
>Fam_315_69_1  Nr. of seq. 1 Alignment length(with gaps) = 69 Alignment score = 0.000000
GCTCAACGGAACATCCAACCCAAACGGATTAATCGCGAAACCAGGGATCGTGACCGCGCTCGTGCCCCC
>Fam_316_69_1  Nr. of seq. 1 Alignment length(with gaps) = 69 Alignment score = 0.000000
GCTCAACGGAATACCAGGAATAGTAATATCCGGCACCACAATCGGACCGACACCACCCAGCGCGTTCAG
>Fam_317_69_1  Nr. of seq. 1 Alignment length(with gaps) = 69 Alignment score = 0.000000
ACTTGTTAAGCCCGTACAGTCGGAAAAAGCATACTCGCCTATTTTGGTAAGATTTGCAGGCAGGCTTAT
>Fam_318_68_1  Nr. of seq. 1 Alignment length(with gaps) = 68 Alignment score = 0.000000
AGCGGTTCGAGGGAGCTGATGTCGGTGCGTNGATACCCAGNGCGTTGAGAGATGTCAGAATTTCGNGC
>Fam_319_68_1  Nr. of seq. 1 Alignment length(with gaps) = 68 Alignment score = 0.000000
ATAATTCCTTAGAGGTATGGAAACANCTGTCGTGNATNNGGNNNATCTNTGCAANGAATNTNGTTTCA
>Fam_320_67_1  Nr. of seq. 1 Alignment length(with gaps) = 67 Alignment score = 0.000000
ATTTATTAAATTTGTGCTAACTGTACAAAATATATTGATTAAGTTTAGATTAGTGATAAAAAGATAT
>Fam_321_67_1  Nr. of seq. 1 Alignment length(with gaps) = 67 Alignment score = 0.000000
```

```
AGATAATATCCTAATCAAATTCTAGTCATTTAAACATAACATCATAAGTAATTTATCTGACAAATTA
>Fam_322_67_1  Nr. of seq. 1 Alignment length(with gaps) = 67 Alignment score = 0.000000
GTTTTAGAGCTATGCTGTTTTGAATGGTCCCAAAACTGNGCATANGGANNACTTAATTGCGCTTTNT
>Fam_323_66_1  Nr. of seq. 1 Alignment length(with gaps) = 66 Alignment score = 0.000000
AATTCCATAAGGTACAATTAATACACNAGGCNTNNGCGTNTNCATNNTTNNNNNNACNTATATTTC
>Fam_324_66_1  Nr. of seq. 1 Alignment length(with gaps) = 66 Alignment score = 0.000000
GCAACCAGATCACCGACATTGCGCCGCTCGCATCGCTCAAATCGCTCACGGAACTCTNGCTCTCCA
>Fam_325_66_1  Nr. of seq. 1 Alignment length(with gaps) = 66 Alignment score = 0.000000
TTACTGCTTCTAAACAAATTTNTTCTGTTTGNTTNTTTACATANTNTAAAGCTTTATAATTTTGTT
>Fam_326_66_1  Nr. of seq. 1 Alignment length(with gaps) = 66 Alignment score = 0.000000
TTTACATACCACATAGTTAATATAAAACNANAANTATTTNTAGGGAANATACTTNGTTATNNAACC
>Fam_327_66_1  Nr. of seq. 1 Alignment length(with gaps) = 66 Alignment score = 0.000000
TACCACTTAGTTAATATAAAACNNATTANTACTNTNANANNNNTAGNNNACNTANTNNNATTTACA
>Fam_328_66_1  Nr. of seq. 1 Alignment length(with gaps) = 66 Alignment score = 0.000000
GAGGCCAGCCAGGGGGGCGAGGTCGGTGACCGCAGTGTAGCTGAGAGTGAGGTTTTGGAGGTTTTC
>Fam_329_66_1  Nr. of seq. 1 Alignment length(with gaps) = 66 Alignment score = 0.000000
GCTCTAAAACTGACNATNATGACTNAATAGCTTTTCANNNGTTTTGGAACCATTCGAAACAACACA
>Fam_330_66_1  Nr. of seq. 1 Alignment length(with gaps) = 66 Alignment score = 0.000000
TTCCTTAGAGGTATGGAAACAGACNTNCTATCTTNTAGNNTCGAAACNNNTTCTACGTTTCAATAC
>Fam_331_66_1  Nr. of seq. 1 Alignment length(with gaps) = 66 Alignment score = 0.000000
TTTGAGAGTTGTGTAATTTAAGATGGATCTCAAACNNNNTNNAANTNNNNNATNNGNNAATNNNNG
>Fam_332_65_1  Nr. of seq. 1 Alignment length(with gaps) = 65 Alignment score = 0.000000
GTTTTAGAGCTGTGCTGTTTCGAATGGTTCCAAAACNNNTNCTNNTNNTTNNAATTGNANAGNNT
>Fam_333_64_1  Nr. of seq. 1 Alignment length(with gaps) = 64 Alignment score = 0.000000
AAGATTTTGATATGAAATTAAAAAATGCAGTAGTTCCCACAGAATTACGCCTCTTAGAACGATC
>Fam_334_64_1  Nr. of seq. 1 Alignment length(with gaps) = 64 Alignment score = 0.000000
GTGTGGGACGGGGCCATGGAACNGNAGNAGNGCGNCCNATCNCGGCANGNACTCTACCATGGCG
>Fam_335_63_1  Nr. of seq. 1 Alignment length(with gaps) = 63 Alignment score = 0.000000
GCACGCGCGGGGCTGGTCCNACGGGTGCAGCCGCATGNCCCAGGCGGCCGGCGTGTTGTCCCC
>Fam_336_63_1  Nr. of seq. 1 Alignment length(with gaps) = 63 Alignment score = 0.000000
AAACATGGGAGCGCTCTCATCCCATTAGAACCGCCTGTTTCCTCCCGCGAGACTTTGCCCCAC
>Fam_337_63_1  Nr. of seq. 1 Alignment length(with gaps) = 63 Alignment score = 0.000000
GAACAGGCCCGCCGCGAAGCCGAGGCGCGGGCGGCGGAAGCGGCAGCACGAGCTGTCGCCGAA
>Fam_338_63_1  Nr. of seq. 1 Alignment length(with gaps) = 63 Alignment score = 0.000000
GGTTTATCCCCGCTGGCGCGGGGAACACNACGGCTCNNGCAANAGCAGNACCCNTGTCAGCTC
>Fam_339_63_1  Nr. of seq. 1 Alignment length(with gaps) = 63 Alignment score = 0.000000
ACGAAAGTTTTGATGAATCAACAAGTGTGAGTTACAAAGAGACGTAATGTTGCGGGAACTCCC
>Fam_340_63_1  Nr. of seq. 1 Alignment length(with gaps) = 63 Alignment score = 0.000000
```

```
 CTAACATCTAAATTTGTTAACTGATTNTTAGNACAAGCCAAATNNNCTAAAGCNGGATTTTTA
>Fam_341_63_1  Nr. of seq. 1 Alignment length(with gaps) = 63 Alignment score = 0.000000
 AGTGAGGTAAGTTNATTTTCGGAACAGTCCAGTTCCTNTAAAGCNGTTAANCCCTGTACATCG
>Fam_342_62_1  Nr. of seq. 1 Alignment length(with gaps) = 62 Alignment score = 0.000000
 GGGCTGGTCCCACGANNGCCAGCGCCCGNCGNGCNNCNNGNTNNGTCGTCCCCGCGTACGCG
>Fam_343_62_1  Nr. of seq. 1 Alignment length(with gaps) = 62 Alignment score = 0.000000
 GGGATAAACCGNACTGNCAGNNNGTGNCNNCNNNAACNNGTNANGAGTTCCCCGCGCCAGCG
>Fam_344_62_1  Nr. of seq. 1 Alignment length(with gaps) = 62 Alignment score = 0.000000
 GTTTCAGAAGTATGTTAAATCAATAAGGTTAAGACCNNATANTANTNNAAANTNNNNATNNA
>Fam_345_61_1  Nr. of seq. 1 Alignment length(with gaps) = 61 Alignment score = 0.000000
 CGGTTTATCCCCGCTGGCGCGGGGAACTCTAGNTGTCNNGCATTNCANCCGGCTNCNGCNG
>Fam_346_61_1  Nr. of seq. 1 Alignment length(with gaps) = 61 Alignment score = 0.000000
 GCGCGGGGAACACNNNNNNNCAANNTNNNNNNTNCNANNANCNTNCGGTTTATCCCCGCTG
>Fam_347_61_1  Nr. of seq. 1 Alignment length(with gaps) = 61 Alignment score = 0.000000
 TTTCTAAGCTGCCTGTGCGGCAGTGAACATAANGTNAANNATAGCANTCGANTTNCCACAG
>Fam_348_61_1  Nr. of seq. 1 Alignment length(with gaps) = 61 Alignment score = 0.000000
 GTTCACTGCCGCACAGGCAGCTTAGAAACCGANATNATCANTTCTANNTNNGTTCTTTNCN
>Fam_349_60_1  Nr. of seq. 1 Alignment length(with gaps) = 60 Alignment score = 0.000000
 AAAAGGCGGAGAAGCCACTAGCAAAAACCATGACAAAGAATTCTATCAAGAAATCGGTGA
>Fam_350_60_1  Nr. of seq. 1 Alignment length(with gaps) = 60 Alignment score = 0.000000
 ATACTGTCATTGAGGACAATCGCGGTAATAGGAAAACCATCAAGAAAGACATCTTTGGAG
>Fam_351_60_1  Nr. of seq. 1 Alignment length(with gaps) = 60 Alignment score = 0.000000
 GGCGACCGTGACCGCGGCGGCTTCCGCCCCCGCGAAGACCGTGGCGAGCGCAGCTTCGGC
>Fam_352_60_1  Nr. of seq. 1 Alignment length(with gaps) = 60 Alignment score = 0.000000
 GTTCACTGCCGTACAGGCAGCTTAGAAANGAANNCGGNNNNANNNGCACGNCGNNATNNN
>Fam_353_60_1  Nr. of seq. 1 Alignment length(with gaps) = 60 Alignment score = 0.000000
 CCGCATACGCGGGGGTGATCCCGATCNNTANNGNNGNTNNAANGCNTNNTNANGTATTTC
>Fam_354_60_1  Nr. of seq. 1 Alignment length(with gaps) = 60 Alignment score = 0.000000
 CATCCCCGCGCATGCGGGGAACAGNCGGCCAAGGCNNNCNNNGNCNGNTNNNGGGCGGTT
>Fam_355_60_1  Nr. of seq. 1 Alignment length(with gaps) = 60 Alignment score = 0.000000
 CCCGCGTGTGCGGGGAACAGNGTNGCCNNNNCNGCNNGANNGNCGCNNGANCGGTTCATC
>Fam_356_60_1  Nr. of seq. 1 Alignment length(with gaps) = 60 Alignment score = 0.000000
 GTTTATCCCCGCTGGCGCGGGGAACACNGAAACGCCGTNTNCGCGACCTCGTGANGANCG
>Fam_357_60_1  Nr. of seq. 1 Alignment length(with gaps) = 60 Alignment score = 0.000000
 GTTTATCCCCGCTGGCGCGGGGAACACNNTNNTTNCNNATNNTANNCNGNNNANCNGNCG
>Fam_358_60_1  Nr. of seq. 1 Alignment length(with gaps) = 60 Alignment score = 0.000000
 CTTTAACTTAAGTTATTAGAGCCTCTTATGCAGTTGCTCAGTCAACTGTATACCTTTTGC
>Fam_359_60_1  Nr. of seq. 1 Alignment length(with gaps) = 60 Alignment score = 0.000000
```

```
 CTTTAACCCAACCAGTAGCCATTGAACCTGATTCGTTTAAGTAATACCATAAGCCTTTGT
>Fam_360_59_1  Nr. of seq. 1 Alignment length(with gaps) = 59 Alignment score = 0.000000
 CCTCCGGCGCGCTCATCACCGGCAACACTTCCTCGTGAGGCGCAGACTGGGAGCGCGCG
>Fam_361_59_1  Nr. of seq. 1 Alignment length(with gaps) = 59 Alignment score = 0.000000
 CCGGCTTCCAGAGGGGTGCGCCGGAGGCGCGCGCTCCCAGCTTGCAGGCGAGCGCGTGC
>Fam_362_59_1  Nr. of seq. 1 Alignment length(with gaps) = 59 Alignment score = 0.000000
 GTTCACTGCCGTACAGGCAGCTTAGAAAANGCANCNTGNNGATNCGCGNTNNTNNCNNN
>Fam_363_59_1  Nr. of seq. 1 Alignment length(with gaps) = 59 Alignment score = 0.000000
 GGGCCCCAACACAGAAGCTGGCCAATAGTCAGCTTTCAATAATGTGCAAGTTGGGGTAA
>Fam_364_58_1  Nr. of seq. 1 Alignment length(with gaps) = 58 Alignment score = 0.000000
 GTGTTCCCCGCATGCGCGGGGATGAACCGNNNNCNGNCNNNNCNGNNCNGNCNGNNGN
>Fam_365_58_1  Nr. of seq. 1 Alignment length(with gaps) = 58 Alignment score = 0.000000
 GCCCGACGACGATGCAGAGCGCGCAGCGCGATGAGAAGGAGTTGGGCGGTTAGGTCGA
>Fam_366_58_1  Nr. of seq. 1 Alignment length(with gaps) = 58 Alignment score = 0.000000
 TCGTCGCCGAGCTCGATTTGATTGCCCGGCTCCTCCTCACCCCGTTACCCGGGGCGCA
>Fam_367_58_1  Nr. of seq. 1 Alignment length(with gaps) = 58 Alignment score = 0.000000
 CGTGGGGATAGACCCNCNCGNCNNNNTCNNGNNCANGCGNNGGCGGTTCCCCCGCACT
>Fam_368_57_1  Nr. of seq. 1 Alignment length(with gaps) = 57 Alignment score = 0.000000
 GGTGCGGGGGCGGAAACCGCCGCGGCCGAAGCTTCCTCGGCCAACGCAGTGGCGCCC
>Fam_369_57_1  Nr. of seq. 1 Alignment length(with gaps) = 57 Alignment score = 0.000000
 TCTACCACAAGTGATAGCACGGACACATCAGCTTCAAGCGACAGTACAGATACTACC
>Fam_370_57_1  Nr. of seq. 1 Alignment length(with gaps) = 57 Alignment score = 0.000000
 GGATCACCTCCACATACGTGGAGAAAANCATTTNNTNTNACNAAAANTCNGNATNGA
>Fam_371_57_1  Nr. of seq. 1 Alignment length(with gaps) = 57 Alignment score = 0.000000
 GTTTTCCCCGCACATGCGGGGGTGATCCCGNNAATNCNANTNTTNCCAGCGNCAANN
>Fam_372_57_1  Nr. of seq. 1 Alignment length(with gaps) = 57 Alignment score = 0.000000
 TTCAGTGAGATGTCTTCACCTCAGTTTATGGAGCGTCAGCTTGCCACCAGCAAGATG
>Fam_373_56_1  Nr. of seq. 1 Alignment length(with gaps) = 56 Alignment score = 0.000000
 TGATCGACGTGCTGGCGAGGGACGAGCCGGTAGGAGATCAGGCAGTCGGCACGGCC
>Fam_374_56_1  Nr. of seq. 1 Alignment length(with gaps) = 56 Alignment score = 0.000000
 GGGGCGGGTTAGGGTGGGGGCGTATCGCAACCCACACGGCAGACCCGGCTACCGTA
>Fam_375_56_1  Nr. of seq. 1 Alignment length(with gaps) = 56 Alignment score = 0.000000
 CCGGCCCGCATCGTCACACGGCCTAAGCCCATTGCCGTCCTCCTCAACGGGCCGAC
>Fam_376_56_1  Nr. of seq. 1 Alignment length(with gaps) = 56 Alignment score = 0.000000
 TGCAAGTTGGCGGGGCCCCAACATAGAGAAATTGGATCACCAATTTCAACAGACAA
>Fam_377_55_1  Nr. of seq. 1 Alignment length(with gaps) = 55 Alignment score = 0.000000
 AGATAAACTTCAATATGGGTTTAACTTGTGGTTAATAGAGTAAGAGTGTAAATAG
>Fam_378_55_1  Nr. of seq. 1 Alignment length(with gaps) = 55 Alignment score = 0.000000
```

```
TATGATGAAAGATGGATTTAATGTATAAGGAATCAATGAANTTACAAAAAGANCT
>Fam_379_54_1  Nr. of seq. 1 Alignment length(with gaps) = 54 Alignment score = 0.000000
CCATATAGATAGTCCGTACCTGTATTAGAAATTAGTGTGTCATTCCCATCACCA
>Fam_380_54_1  Nr. of seq. 1 Alignment length(with gaps) = 54 Alignment score = 0.000000
GTGCAGAGCCAGAACGGCGCCAATATCGGCTCGGGCGCCAATGGCATCAGCGTC
>Fam_381_54_1  Nr. of seq. 1 Alignment length(with gaps) = 54 Alignment score = 0.000000
TGTCGTTGCCGGCGCCACCATCAAGGGTGTCCGCACCTGCACCACCNTAAAGCG
>Fam_382_51_1  Nr. of seq. 1 Alignment length(with gaps) = 51 Alignment score = 0.000000
CGGCGAGCGGGGGCGGGAGTTTGAGCGAGAACCACGGGCACGGCGCGATAG
>Fam_383_51_1  Nr. of seq. 1 Alignment length(with gaps) = 51 Alignment score = 0.000000
TACTTATTAATAGCATTTAGATATTAATTGTATTTACCTATTTGAGTGAAT
>Fam_384_51_1  Nr. of seq. 1 Alignment length(with gaps) = 51 Alignment score = 0.000000
CTAGTAAGATATACTAATATATAAATACTTAAATTTAATTTTATTTAAACA
>Fam_385_51_1  Nr. of seq. 1 Alignment length(with gaps) = 51 Alignment score = 0.000000
GTACAAGCAAACAAGAAGCCAACAGTACCAAAAATACTAATCAAACAACCA
>Fam_386_50_1  Nr. of seq. 1 Alignment length(with gaps) = 50 Alignment score = 0.000000
TATTATAGATGAATATTAATATTGATATTCTAGGTAATTGCTAGAGCTAA
>Fam_387_49_1  Nr. of seq. 1 Alignment length(with gaps) = 49 Alignment score = 0.000000
GCATGGGATCGGGGCAAGCGCCGAAGCGCTAACTCCGATCGACAGCTTT
>Fam_388_49_1  Nr. of seq. 1 Alignment length(with gaps) = 49 Alignment score = 0.000000
CCCATGCTTCACGCTCGACCAGAGTTGGCGCTTGAGCACTTACTCTGGT
>Fam_389_49_1  Nr. of seq. 1 Alignment length(with gaps) = 49 Alignment score = 0.000000
AATCTTAGAAAATGTAGGAACTACTACTTTCAAACAAAGAACCGCAATC
>Fam_390_48_1  Nr. of seq. 1 Alignment length(with gaps) = 48 Alignment score = 0.000000
GCAACCACGCCGGTACGTCGCTACCCGCCGCTGGAGCNGCCTCTTCCC
>Fam_391_48_1  Nr. of seq. 1 Alignment length(with gaps) = 48 Alignment score = 0.000000
GCTGCTGAACTGCCCGCACTCGATGCTGACGAACTAGCAGCACTGGAT
>Fam_392_48_1  Nr. of seq. 1 Alignment length(with gaps) = 48 Alignment score = 0.000000
AAACCTGGGACTACCGAACCCGAGAAACCCGGAGTCACCGAACCAGAA
>Fam_393_48_1  Nr. of seq. 1 Alignment length(with gaps) = 48 Alignment score = 0.000000
TGTAGGCGGTGATGAAGACTGGCTGTCGCTGCCACTGCTGCTACTGTC
>Fam_394_48_1  Nr. of seq. 1 Alignment length(with gaps) = 48 Alignment score = 0.000000
ACCATCTTGCAGACGGTGTAGTTGCAAGTCTTGGTGCGGCACTCAGGA
>Fam_395_48_1  Nr. of seq. 1 Alignment length(with gaps) = 48 Alignment score = 0.000000
GTGTAGTTGATCACTTTCGTACGAGTTTCGTACACAGGCTTCTTGACT
>Fam_396_48_1  Nr. of seq. 1 Alignment length(with gaps) = 48 Alignment score = 0.000000
GTGCTTGCTGAGGCTGAAGCACTTGTTGAGGCTGACTCAGATGCNGAT
>Fam_397_48_1  Nr. of seq. 1 Alignment length(with gaps) = 48 Alignment score = 0.000000
```

TGAAGCACTTTGGCTTGCNCTTGTTGANGCGGACTTAGACGCGCTTGT
>Fam_398_47_1 Nr. of seq. 1 Alignment length(with gaps) = 47 Alignment score = 0.000000
TTTTCAAAGTTAGTCCGTAAAATACAGATTTGTGGGAACTATTACAC
>Fam_399_45_1 Nr. of seq. 1 Alignment length(with gaps) = 45 Alignment score = 0.000000
CATATGGATTTTGTTGATATTGTTGTTCTTGGTTTTGTGGTGCTA
>Fam_400_45_1 Nr. of seq. 1 Alignment length(with gaps) = 45 Alignment score = 0.000000
AACGGAACCCAGTACGAAAGAAACCAGCAGGAAGAACCCAAGCAC
>Fam_401_45_1 Nr. of seq. 1 Alignment length(with gaps) = 45 Alignment score = 0.000000
AAGTAAGAAATTTAGGAAGTAGGAAGTTTAGTAATAATTTGTAGT
>Fam_402_45_1 Nr. of seq. 1 Alignment length(with gaps) = 45 Alignment score = 0.000000
AAAGAAAATAATTAATATAGAGTAAATCTATTGCAATGAATTAAC
>Fam_403_45_1 Nr. of seq. 1 Alignment length(with gaps) = 45 Alignment score = 0.000000
TTTTTATTATCTGTTGACTTATCTTCTTCTGGGGTCTGCTCTGGC
>Fam_404_45_1 Nr. of seq. 1 Alignment length(with gaps) = 45 Alignment score = 0.000000
AATTAGTTTATTATACTTCTTTAGATTAATTTTCTATACTTCCTA
>Fam_405_45_1 Nr. of seq. 1 Alignment length(with gaps) = 45 Alignment score = 0.000000
CGGGAGCTGCAGGCCCGGCAGGNCCACAGGGACCGAAAGGGGATA
>Fam_406_45_1 Nr. of seq. 1 Alignment length(with gaps) = 45 Alignment score = 0.000000
GTCGGAACTGGAGTCCACAGCGCTTGAACTTTCCGAACTAGAATC
>Fam_407_45_1 Nr. of seq. 1 Alignment length(with gaps) = 45 Alignment score = 0.000000
CAGGGATTACCNGGACCTAAAGGAGACCAAGGGGAGGCAGGACCG
>Fam_408_45_1 Nr. of seq. 1 Alignment length(with gaps) = 45 Alignment score = 0.000000
TGCTTGTCCTGATTTTGCTGATCCTGGTTCTGTTGATCTTTGTTT
>Fam_409_45_1 Nr. of seq. 1 Alignment length(with gaps) = 45 Alignment score = 0.000000
CTGATCGAACGTTCTGGCCGCGTTGCTGAAGGTGGCTCCGATCGT
>Fam_410_44_1 Nr. of seq. 1 Alignment length(with gaps) = 44 Alignment score = 0.000000
CGCCGTACGCGGCTCTCCCCGCCATCGCTCCGGGTACCCCTCCT
>Fam_411_43_1 Nr. of seq. 1 Alignment length(with gaps) = 43 Alignment score = 0.000000
GAGCACTGCTCACNNGCGGCGCCGGCGNAGTGCTCNGCNTCGA
>Fam_412_42_1 Nr. of seq. 1 Alignment length(with gaps) = 42 Alignment score = 0.000000
CCCGCAAACTTCGAGGGAGTCTGGCCCGAAGGGGGACAGGCA
>Fam_413_42_1 Nr. of seq. 1 Alignment length(with gaps) = 42 Alignment score = 0.000000
AACAGCACGGCGANCGGGACGAACTCGACCGCGTCGGGCGAG
>Fam_414_42_1 Nr. of seq. 1 Alignment length(with gaps) = 42 Alignment score = 0.000000
AGCACGGCAACGGGCCAAGGNTCGCAAGCGACGGGNNGCAAC
>Fam_415_42_1 Nr. of seq. 1 Alignment length(with gaps) = 42 Alignment score = 0.000000
TCGCTGTCGACGGGGCTCAGCACGACGAACAGCAGCGTCGCG
>Fam_416_42_1 Nr. of seq. 1 Alignment length(with gaps) = 42 Alignment score = 0.000000

ATTTCCTATCTCCTCACTCCTATCTCTTACCCTCTCACTCCT
>Fam_417_42_1 Nr. of seq. 1 Alignment length(with gaps) = 42 Alignment score = 0.000000
GAAGAGCAGGCGCGGCGCGAAGCCGAGGCGCGCGCTGCTGCC
>Fam_418_42_1 Nr. of seq. 1 Alignment length(with gaps) = 42 Alignment score = 0.000000
CTTGCATATCGCTAATAGTGCCATTCATACTACTGATACTGC
>Fam_419_42_1 Nr. of seq. 1 Alignment length(with gaps) = 42 Alignment score = 0.000000
TTGCTTGTTAGTATTAGTATTTCAATTGTCAATATTAGTGTT
>Fam_420_42_1 Nr. of seq. 1 Alignment length(with gaps) = 42 Alignment score = 0.000000
TAAAAAATAATTGAGTTAATTATGCATTAGATGTATATTATA
>Fam_421_42_1 Nr. of seq. 1 Alignment length(with gaps) = 42 Alignment score = 0.000000
TCAGCTTTTGGATNTNATAATANAGCTANTGGANTNNATAGT
>Fam_422_42_1 Nr. of seq. 1 Alignment length(with gaps) = 42 Alignment score = 0.000000
TGGCGCTCGGCNCCGGTGCGCAGGCCACCNNCGTCAACGNCG
>Fam_423_42_1 Nr. of seq. 1 Alignment length(with gaps) = 42 Alignment score = 0.000000
TGCGGCCAAACCGGCTGCCAAGGCGACCGCTGCCGCCAAGCC
>Fam_424_42_1 Nr. of seq. 1 Alignment length(with gaps) = 42 Alignment score = 0.000000
GGACAAGAAGCGGGCCCCGCGCCCCGGTGTGCCGGCTCGCGG
>Fam_425_42_1 Nr. of seq. 1 Alignment length(with gaps) = 42 Alignment score = 0.000000
TCCGCGCCCGCTCCCGGTCCCTCCGAACCCGCATCCGGGCCT
>Fam_426_41_1 Nr. of seq. 1 Alignment length(with gaps) = 41 Alignment score = 0.000000
AATTAAAAGTGAATGGAGAGTAAGAATAAGTTCTAATGAAG
>Fam_427_40_1 Nr. of seq. 1 Alignment length(with gaps) = 40 Alignment score = 0.000000
TTAATCTATAGTATAATAGTTTAATTTGAAGTACAACGAT
>Fam_428_39_1 Nr. of seq. 1 Alignment length(with gaps) = 39 Alignment score = 0.000000
GCTTCTGCTTGNCGTTTNGCTTCAGCNGCTTTTTTGTCT
>Fam_429_39_1 Nr. of seq. 1 Alignment length(with gaps) = 39 Alignment score = 0.000000
TAAGCAACAAAGTNCTAAACAAGANGATTCCTCTCAGGA
>Fam_430_39_1 Nr. of seq. 1 Alignment length(with gaps) = 39 Alignment score = 0.000000
TTATCTTTAGATTTCCACCAATCGTCATCACAATTGTGC
>Fam_431_39_1 Nr. of seq. 1 Alignment length(with gaps) = 39 Alignment score = 0.000000
TGCTCTTCTTGCTTACGTTTTTCTTCTTCTACTCTTTGC
>Fam_432_39_1 Nr. of seq. 1 Alignment length(with gaps) = 39 Alignment score = 0.000000
ATTCCCCTCCTCTGGAGGGGTGCCCGTCAGGGCAGTTTT
>Fam_433_39_1 Nr. of seq. 1 Alignment length(with gaps) = 39 Alignment score = 0.000000
TCCCAGGTATCTCGGTCGGTGCGCGCCGGAGGCGTGCGC
>Fam_434_39_1 Nr. of seq. 1 Alignment length(with gaps) = 39 Alignment score = 0.000000
CTTTGAATATTCTACAAGACTATAGATTAGTTTAAATCC
>Fam_435_39_1 Nr. of seq. 1 Alignment length(with gaps) = 39 Alignment score = 0.000000

```
GCAGATATAAGGGAAGTATTGAGGGAACTTGGGAAGAAG
>Fam_436_39_1  Nr. of seq. 1 Alignment length(with gaps) = 39 Alignment score = 0.000000
CCGCCGTCTAAACCATCAACACCTTCTAGCTCAGTGACA
>Fam_437_39_1  Nr. of seq. 1 Alignment length(with gaps) = 39 Alignment score = 0.000000
TATGTGAAGTTCAAGCCATTTGAAGTCGCGGGTCACCCA
>Fam_438_39_1  Nr. of seq. 1 Alignment length(with gaps) = 39 Alignment score = 0.000000
GCGGCGACGACCGGCGGAGACAGCGCGCCACCGTCGAGA
>Fam_439_38_1  Nr. of seq. 1 Alignment length(with gaps) = 38 Alignment score = 0.000000
GGGGAGCGACATCTGCGGCCAGGGAGCCTGTCCCTGCC
>Fam_440_38_1  Nr. of seq. 1 Alignment length(with gaps) = 38 Alignment score = 0.000000
AAAATATTTAAATAATATATCATAATACAGAGTGANGA
>Fam_441_38_1  Nr. of seq. 1 Alignment length(with gaps) = 38 Alignment score = 0.000000
GTCGCACGATAGTCGAGATTACGGCCTGCCGACGCGGC
>Fam_442_36_1  Nr. of seq. 1 Alignment length(with gaps) = 36 Alignment score = 0.000000
CCGCAGAAGAGGCGCGTATTGCTGCCGAGGCCCGTG
>Fam_443_36_1  Nr. of seq. 1 Alignment length(with gaps) = 36 Alignment score = 0.000000
AAAACCTGATACGGGAGGAAATCCAGACACAGGAGG
>Fam_444_36_1  Nr. of seq. 1 Alignment length(with gaps) = 36 Alignment score = 0.000000
ACCGGAGCAACAGGCCCGACTGGAATAACGGGCCCA
>Fam_445_36_1  Nr. of seq. 1 Alignment length(with gaps) = 36 Alignment score = 0.000000
CCAGTCGGTCCAGTAATACCAGTCCTTCCAGTAACA
>Fam_446_36_1  Nr. of seq. 1 Alignment length(with gaps) = 36 Alignment score = 0.000000
GCGGGTTCACCTCGGGGCTCGACCGCAGCGCCTTCT
>Fam_447_36_1  Nr. of seq. 1 Alignment length(with gaps) = 36 Alignment score = 0.000000
GCGAGACGGTCGGTGTTGCCGTTTCCGTTGGTGTCG
>Fam_448_36_1  Nr. of seq. 1 Alignment length(with gaps) = 36 Alignment score = 0.000000
TACTCATTAAAGCGACTACTATAGAGTAGATAAAAT
>Fam_449_36_1  Nr. of seq. 1 Alignment length(with gaps) = 36 Alignment score = 0.000000
TCTATATTTATATCTGGTTTTCCATCCCCATCTGTA
>Fam_450_36_1  Nr. of seq. 1 Alignment length(with gaps) = 36 Alignment score = 0.000000
GGTGGATTTANCACTGCATCATCCATTGGTGGCACT
>Fam_451_36_1  Nr. of seq. 1 Alignment length(with gaps) = 36 Alignment score = 0.000000
CAAGTGGCGAACCAGACAAAGATAAAGAACCAGATG
>Fam_452_36_1  Nr. of seq. 1 Alignment length(with gaps) = 36 Alignment score = 0.000000
CACAAGAAGAAAGCGGCCAAGAAACATAAGAAGTCG
>Fam_453_36_1  Nr. of seq. 1 Alignment length(with gaps) = 36 Alignment score = 0.000000
TGGTTGCTCAGGTTGACCCGGTTCTTCCGGTTGACT
>Fam_454_36_1  Nr. of seq. 1 Alignment length(with gaps) = 36 Alignment score = 0.000000
```

GGCCTTGCCCTGGGCCTGCTGGTCGGTGGTGGTGCT
>Fam_455_36_1 Nr. of seq. 1 Alignment length(with gaps) = 36 Alignment score = 0.000000
GCCATACCGGGGTGAGGCGGCAAACCAGGACGAGGA
>Fam_456_36_1 Nr. of seq. 1 Alignment length(with gaps) = 36 Alignment score = 0.000000
GCAGATAAAAAAGCAGCAGCTGAAAGGAAGGCCGCT
>Fam_457_36_1 Nr. of seq. 1 Alignment length(with gaps) = 36 Alignment score = 0.000000
CAAGTACGTCNGCNTCNACGAGTGCATCAGTAAGNG
>Fam_458_34_1 Nr. of seq. 1 Alignment length(with gaps) = 34 Alignment score = 0.000000
TCGAGCGCCCATAGAAGCGAGATGCTGAGTTATT
>Fam_459_33_1 Nr. of seq. 1 Alignment length(with gaps) = 33 Alignment score = 0.000000
TATTCACAAAACAGAGACAACAGAACTGGTGGA
>Fam_460_33_1 Nr. of seq. 1 Alignment length(with gaps) = 33 Alignment score = 0.000000
ATGGTTTCTCCCCATCTGGTATCTGACCATCTG
>Fam_461_33_1 Nr. of seq. 1 Alignment length(with gaps) = 33 Alignment score = 0.000000
CTACTGGGTCTCCTATAAATTTCCCTTGGAATT
>Fam_462_33_1 Nr. of seq. 1 Alignment length(with gaps) = 33 Alignment score = 0.000000
GAATTAAAAGATGANGCAGNAGNAAAAGCAGAG
>Fam_463_33_1 Nr. of seq. 1 Alignment length(with gaps) = 33 Alignment score = 0.000000
GGTTTACTTGGTTCAGTCGTCCCTGGCTCACCT
>Fam_464_33_1 Nr. of seq. 1 Alignment length(with gaps) = 33 Alignment score = 0.000000
GGTCAGCTTTTGCTCCACTGCATCAATACGCTG
>Fam_465_33_1 Nr. of seq. 1 Alignment length(with gaps) = 33 Alignment score = 0.000000
CGATGAGCCGCTTGCGCGAAGAGGTTCAGGGCC
>Fam_466_33_1 Nr. of seq. 1 Alignment length(with gaps) = 33 Alignment score = 0.000000
ACCCCGCCCGACGATAGCGGCGATGACGATGTG
>Fam_467_32_1 Nr. of seq. 1 Alignment length(with gaps) = 32 Alignment score = 0.000000
TTCGGACCGCGGTACTTTCGGACTCCGGTAGG
>Fam_468_31_1 Nr. of seq. 1 Alignment length(with gaps) = 31 Alignment score = 0.000000
GGGTGCCTGTCCCTGTACGGGAGAAGTTTGC
>Fam_469_31_1 Nr. of seq. 1 Alignment length(with gaps) = 31 Alignment score = 0.000000
CCCCCAGCGGGAAACCTCTCCCCACCTCCTC
>Fam_470_30_1 Nr. of seq. 1 Alignment length(with gaps) = 30 Alignment score = 0.000000
CGCGACATCTCCACGCGCGGGATATCCGGC
>Fam_471_30_1 Nr. of seq. 1 Alignment length(with gaps) = 30 Alignment score = 0.000000
GGGCTGGGCGTCGTGCTCGCCGTGGCCGAT
>Fam_472_30_1 Nr. of seq. 1 Alignment length(with gaps) = 30 Alignment score = 0.000000
ACGGCCACCAACACACCGACCAATACACCC
>Fam_473_30_1 Nr. of seq. 1 Alignment length(with gaps) = 30 Alignment score = 0.000000

TTGCTATTTCCTCCATTACCTGAATCCGGT
>Fam_474_30_1 Nr. of seq. 1 Alignment length(with gaps) = 30 Alignment score = 0.000000
GGCGGTGATACGCCTGTTCCGCCAGACGAC
>Fam_475_30_1 Nr. of seq. 1 Alignment length(with gaps) = 30 Alignment score = 0.000000
TTCACATCCGGCACGTTCACGCCGGGCACA
>Fam_476_30_1 Nr. of seq. 1 Alignment length(with gaps) = 30 Alignment score = 0.000000
GTTTCGCCGCCTCCGGTCCCCGGGTCGACG
>Fam_477_30_1 Nr. of seq. 1 Alignment length(with gaps) = 30 Alignment score = 0.000000
CTGCTGGTTTGTTTTCTGCCGGCTTATTGT
>Fam_478_30_1 Nr. of seq. 1 Alignment length(with gaps) = 30 Alignment score = 0.000000
GAATCCGAAGAATCTGAACCGAAACCGCCG
>Fam_479_30_1 Nr. of seq. 1 Alignment length(with gaps) = 30 Alignment score = 0.000000
CCCGGCGGCGGGGCCCTCAGGTCGGGACAG
>Fam_480_30_1 Nr. of seq. 1 Alignment length(with gaps) = 30 Alignment score = 0.000000
CCACACCGCCGGAGCCAGCACCACCAGGCC
>Fam_481_30_1 Nr. of seq. 1 Alignment length(with gaps) = 30 Alignment score = 0.000000
GACCGTCCGGCGCGGGGTCGCGCTCGGGCT
>Fam_482_28_1 Nr. of seq. 1 Alignment length(with gaps) = 28 Alignment score = 0.000000
ATTGCAAAGTGCAAATTGATCAATGCAA
>Fam_483_28_1 Nr. of seq. 1 Alignment length(with gaps) = 28 Alignment score = 0.000000
TACATTTCAGAAGAAAATGCTGCGCCGT
>Fam_484_28_1 Nr. of seq. 1 Alignment length(with gaps) = 28 Alignment score = 0.000000
TCACTTACTTGAGTAAGCTCATCGGGAT
>Fam_485_27_1 Nr. of seq. 1 Alignment length(with gaps) = 27 Alignment score = 0.000000
GTTACCTGCACCACCTGCTATTGTATC
>Fam_486_27_1 Nr. of seq. 1 Alignment length(with gaps) = 27 Alignment score = 0.000000
CCAAGAAGGCGGCAAGCAAGACCGCCN
>Fam_487_27_1 Nr. of seq. 1 Alignment length(with gaps) = 27 Alignment score = 0.000000
GGCGTCACCGACGGCGTAGGGGTGGTC
>Fam_488_27_1 Nr. of seq. 1 Alignment length(with gaps) = 27 Alignment score = 0.000000
CTGCATTTCCTGCATTAGCATCTCCTT
>Fam_489_27_1 Nr. of seq. 1 Alignment length(with gaps) = 27 Alignment score = 0.000000
GATGAACTTGAACCACCTGTACTTGAT
>Fam_490_27_1 Nr. of seq. 1 Alignment length(with gaps) = 27 Alignment score = 0.000000
CCGGTGGTGCCGCCTGGTGGGTTTCCC
>Fam_491_27_1 Nr. of seq. 1 Alignment length(with gaps) = 27 Alignment score = 0.000000
GATACTAAAGAAGTGAAGGAAGCTAAA
>Fam_492_27_1 Nr. of seq. 1 Alignment length(with gaps) = 27 Alignment score = 0.000000

```
 ACGCGGAGCGTCCAGCACTGCATTCCC
>Fam_493_27_1  Nr. of seq. 1 Alignment length(with gaps) = 27 Alignment score = 0.000000
 TGAGGCGTCCCGCCTCAGAAGACAGCT
>Fam_494_27_1  Nr. of seq. 1 Alignment length(with gaps) = 27 Alignment score = 0.000000
 CGCCATAGCCCGACGCGCCGGGAGCGG
>Fam_495_26_1  Nr. of seq. 1 Alignment length(with gaps) = 26 Alignment score = 0.000000
 CTGGACGCGGCACGCGTCGGTTCGGT
>Fam_496_25_1  Nr. of seq. 1 Alignment length(with gaps) = 25 Alignment score = 0.000000
 CCTCCGCCAGGACTGGAAGCACGCG
>Fam_497_25_1  Nr. of seq. 1 Alignment length(with gaps) = 25 Alignment score = 0.000000
 GGCACGCGCTAATGGTGGTCGTGCA
>Fam_498_24_1  Nr. of seq. 1 Alignment length(with gaps) = 24 Alignment score = 0.000000
 CCTGGAACACCAGATCCAGAAAAA
>Fam_499_24_1  Nr. of seq. 1 Alignment length(with gaps) = 24 Alignment score = 0.000000
 CGGTCGCCGAAGTCGCGCTGGGGN
>Fam_500_24_1  Nr. of seq. 1 Alignment length(with gaps) = 24 Alignment score = 0.000000
 ACCGGGCTGGTCGTTACCGTCGGC
>Fam_501_24_1  Nr. of seq. 1 Alignment length(with gaps) = 24 Alignment score = 0.000000
 GCACGCGCACCGCTACGCCAACCG
>Fam_502_24_1  Nr. of seq. 1 Alignment length(with gaps) = 24 Alignment score = 0.000000
 CCAACTACAGGTGGAAACACAACA
>Fam_503_24_1  Nr. of seq. 1 Alignment length(with gaps) = 24 Alignment score = 0.000000
 TCTGGTTTTTCTTCTGGGTCTACC
>Fam_504_24_1  Nr. of seq. 1 Alignment length(with gaps) = 24 Alignment score = 0.000000
 GGCAGGAAGGNCATCAAATTGGCT
>Fam_505_24_1  Nr. of seq. 1 Alignment length(with gaps) = 24 Alignment score = 0.000000
 CAAACTTGAGGAAGAGCAACGAAT
>Fam_506_24_1  Nr. of seq. 1 Alignment length(with gaps) = 24 Alignment score = 0.000000
 TGAATCTCTTCTATCTCCACGACC
>Fam_507_24_1  Nr. of seq. 1 Alignment length(with gaps) = 24 Alignment score = 0.000000
 CTGGTGTTGCCGGACGTGCCACCA
>Fam_508_24_1  Nr. of seq. 1 Alignment length(with gaps) = 24 Alignment score = 0.000000
 TGGTCCATCCCAGCCATGTTGCCG
>Fam_509_24_1  Nr. of seq. 1 Alignment length(with gaps) = 24 Alignment score = 0.000000
 CGCAGCAAGGTGCGGGAGCTGGCT
>Fam_510_24_1  Nr. of seq. 1 Alignment length(with gaps) = 24 Alignment score = 0.000000
 GGCTTCGCCACCACCGGTTCGGCG
>Fam_511_24_1  Nr. of seq. 1 Alignment length(with gaps) = 24 Alignment score = 0.000000
```

CCAGGCTTGTTGTTGTCTTCTTTG
>Fam_512_24_1 Nr. of seq. 1 Alignment length(with gaps) = 24 Alignment score = 0.000000
TTAACGTCTGGCTTAGCTTCTGGC
>Fam_513_24_1 Nr. of seq. 1 Alignment length(with gaps) = 24 Alignment score = 0.000000
CCCGCACGCGCACGTCCCCACCGC
>Fam_514_24_1 Nr. of seq. 1 Alignment length(with gaps) = 24 Alignment score = 0.000000
GCGTACGGCGGGNCCCGTTCCGAC
>Fam_515_24_1 Nr. of seq. 1 Alignment length(with gaps) = 24 Alignment score = 0.000000
TCCGGTGCCGGGTCCGGTGCTACT
>Fam_516_24_1 Nr. of seq. 1 Alignment length(with gaps) = 24 Alignment score = 0.000000
CGCCCGGTCCGTCATCCGGCGTCA
>Fam_517_23_1 Nr. of seq. 1 Alignment length(with gaps) = 23 Alignment score = 0.000000
GGGGTTGCGGAGAGCGGCAAGGC
>Fam_518_23_1 Nr. of seq. 1 Alignment length(with gaps) = 23 Alignment score = 0.000000
ATCCGGACACCCATTCCACNGGC
>Fam_519_23_1 Nr. of seq. 1 Alignment length(with gaps) = 23 Alignment score = 0.000000
GGTGGCAGCCGTCGACCCGCACG
>Fam_520_23_1 Nr. of seq. 1 Alignment length(with gaps) = 23 Alignment score = 0.000000
AGGGGTGTCGGCCTCACGCGGTA
>Fam_521_23_1 Nr. of seq. 1 Alignment length(with gaps) = 23 Alignment score = 0.000000
GCGGCTCCTCGCCAGGCGTCCGT
>Fam_522_22_1 Nr. of seq. 1 Alignment length(with gaps) = 22 Alignment score = 0.000000
TTACGGTGCTGCGTAGCCAAAG
>Fam_523_22_1 Nr. of seq. 1 Alignment length(with gaps) = 22 Alignment score = 0.000000
ACAAAGTAAAACCACAAAGTTC
>Fam_524_22_1 Nr. of seq. 1 Alignment length(with gaps) = 22 Alignment score = 0.000000
TTCGGGGCTGTTGATATTGCAT
>Fam_525_22_1 Nr. of seq. 1 Alignment length(with gaps) = 22 Alignment score = 0.000000
GAACCATCAGATTTTGAGCGAA
>Fam_526_22_1 Nr. of seq. 1 Alignment length(with gaps) = 22 Alignment score = 0.000000
TCAACTATCGTGCCACGCCCTT
>Fam_527_22_1 Nr. of seq. 1 Alignment length(with gaps) = 22 Alignment score = 0.000000
CGACGAACGAGCAGCGGACCAC
>Fam_528_21_1 Nr. of seq. 1 Alignment length(with gaps) = 21 Alignment score = 0.000000
TCAGAAGCAGCAGCAGCAGCT
>Fam_529_21_1 Nr. of seq. 1 Alignment length(with gaps) = 21 Alignment score = 0.000000
CAATCACGCGAGTTGCCTCCN
>Fam_530_21_1 Nr. of seq. 1 Alignment length(with gaps) = 21 Alignment score = 0.000000

TTCAAATCTTTTATCNACNTG
>Fam_531_21_1 Nr. of seq. 1 Alignment length(with gaps) = 21 Alignment score = 0.000000
GCCAACCACCGTGTCCGCCTT
>Fam_532_21_1 Nr. of seq. 1 Alignment length(with gaps) = 21 Alignment score = 0.000000
GTGTCAGAGTCNGCATTNAAA
>Fam_533_21_1 Nr. of seq. 1 Alignment length(with gaps) = 21 Alignment score = 0.000000
TATCTGCTCATTATAACCAGT
>Fam_534_21_1 Nr. of seq. 1 Alignment length(with gaps) = 21 Alignment score = 0.000000
AGCTCGCCGACGGCACCGGCG
>Fam_535_21_1 Nr. of seq. 1 Alignment length(with gaps) = 21 Alignment score = 0.000000
CCTCCTCGGCGGCGCTCTCGC
>Fam_536_21_1 Nr. of seq. 1 Alignment length(with gaps) = 21 Alignment score = 0.000000
CTGTTCTTCCCACCGCCGAGC
>Fam_537_21_1 Nr. of seq. 1 Alignment length(with gaps) = 21 Alignment score = 0.000000
TCGGGGCGGGTTGGGTACCGG
>Fam_538_21_1 Nr. of seq. 1 Alignment length(with gaps) = 21 Alignment score = 0.000000
TACACAATATATAATAGCAAT
>Fam_539_21_1 Nr. of seq. 1 Alignment length(with gaps) = 21 Alignment score = 0.000000
CCTGACGATGGCGGCAATACT
>Fam_540_21_1 Nr. of seq. 1 Alignment length(with gaps) = 21 Alignment score = 0.000000
TTGGTGAACTTTCTTGCTCTT
>Fam_541_21_1 Nr. of seq. 1 Alignment length(with gaps) = 21 Alignment score = 0.000000
AAATCTTTCTGTTCTTTAACC
>Fam_542_21_1 Nr. of seq. 1 Alignment length(with gaps) = 21 Alignment score = 0.000000
TCATAATTAACCCTCAAATCA
>Fam_543_21_1 Nr. of seq. 1 Alignment length(with gaps) = 21 Alignment score = 0.000000
GCCGGCTTCATGGCATCNGCN
>Fam_544_21_1 Nr. of seq. 1 Alignment length(with gaps) = 21 Alignment score = 0.000000
TCTATGGAAAGTCGTTTAGAC
>Fam_545_21_1 Nr. of seq. 1 Alignment length(with gaps) = 21 Alignment score = 0.000000
GGCGGCATGGGAGGCATGGGC
>Fam_546_21_1 Nr. of seq. 1 Alignment length(with gaps) = 21 Alignment score = 0.000000
GGCAAGGGCAGCCAGGACAAC
>Fam_547_21_1 Nr. of seq. 1 Alignment length(with gaps) = 21 Alignment score = 0.000000
TCAATTCTTTTATTTGTTTCA
>Fam_548_19_1 Nr. of seq. 1 Alignment length(with gaps) = 19 Alignment score = 0.000000
TAGATATTTAATTAAGTGG
>Fam_549_19_1 Nr. of seq. 1 Alignment length(with gaps) = 19 Alignment score = 0.000000

TATATAAAATTTANTAATT
>Fam_550_19_1 Nr. of seq. 1 Alignment length(with gaps) = 19 Alignment score = 0.000000
AACTGGTTTAATCGTGACT
>Fam_551_18_1 Nr. of seq. 1 Alignment length(with gaps) = 18 Alignment score = 0.000000
GCGCGGTTGTGGACGACG
>Fam_552_18_1 Nr. of seq. 1 Alignment length(with gaps) = 18 Alignment score = 0.000000
CCTGTTACTCCAGTTGGA
>Fam_553_18_1 Nr. of seq. 1 Alignment length(with gaps) = 18 Alignment score = 0.000000
ACGGGGAACACCGGACCC
>Fam_554_18_1 Nr. of seq. 1 Alignment length(with gaps) = 18 Alignment score = 0.000000
CCTGATACAGGAGAAAAA
>Fam_555_18_1 Nr. of seq. 1 Alignment length(with gaps) = 18 Alignment score = 0.000000
GTGATGCCTGTAGGACCA
>Fam_556_18_1 Nr. of seq. 1 Alignment length(with gaps) = 18 Alignment score = 0.000000
CGTCTGGTTGTTTGTCTC
>Fam_557_18_1 Nr. of seq. 1 Alignment length(with gaps) = 18 Alignment score = 0.000000
CTGGACAAGCAAGACAAT
>Fam_558_18_1 Nr. of seq. 1 Alignment length(with gaps) = 18 Alignment score = 0.000000
GACGGCAAGCCNGGCGCN
>Fam_559_18_1 Nr. of seq. 1 Alignment length(with gaps) = 18 Alignment score = 0.000000
CCCGAGCCGGTGNTCGAG
>Fam_560_18_1 Nr. of seq. 1 Alignment length(with gaps) = 18 Alignment score = 0.000000
GGCGGGTTTAGCACCCAC
>Fam_561_18_1 Nr. of seq. 1 Alignment length(with gaps) = 18 Alignment score = 0.000000
GTGCGGGTTGGTGTCGGC
>Fam_562_18_1 Nr. of seq. 1 Alignment length(with gaps) = 18 Alignment score = 0.000000
TGACCCTGGTTTTTCTGT
>Fam_563_18_1 Nr. of seq. 1 Alignment length(with gaps) = 18 Alignment score = 0.000000
GTGATCGGGGTTCCCGGC
>Fam_564_18_1 Nr. of seq. 1 Alignment length(with gaps) = 18 Alignment score = 0.000000
CGCCTGGCCAGTACGGCC
>Fam_565_18_1 Nr. of seq. 1 Alignment length(with gaps) = 18 Alignment score = 0.000000
TGCTCGGGGTAACCGCGT
>Fam_566_18_1 Nr. of seq. 1 Alignment length(with gaps) = 18 Alignment score = 0.000000
AAGCTGCCCAAGAACTTA
>Fam_567_18_1 Nr. of seq. 1 Alignment length(with gaps) = 18 Alignment score = 0.000000
GAACCAGGTAAACCAGCG
>Fam_568_18_1 Nr. of seq. 1 Alignment length(with gaps) = 18 Alignment score = 0.000000

CCGTCCCGGGCGCCGTAN
>Fam_569_18_1 Nr. of seq. 1 Alignment length(with gaps) = 18 Alignment score = 0.000000
GCCGCGGCGGGCTCCGGG
>Fam_570_18_1 Nr. of seq. 1 Alignment length(with gaps) = 18 Alignment score = 0.000000
TACGGCCAGCAGCCCCCN
>Fam_571_18_1 Nr. of seq. 1 Alignment length(with gaps) = 18 Alignment score = 0.000000
GGGTGGCCGGGCGGCCGT
>Fam_572_18_1 Nr. of seq. 1 Alignment length(with gaps) = 18 Alignment score = 0.000000
GAAGCGGCACTGGCTTCC
>Fam_573_18_1 Nr. of seq. 1 Alignment length(with gaps) = 18 Alignment score = 0.000000
AGGAGCCTTTGGAGCCTC
>Fam_574_18_1 Nr. of seq. 1 Alignment length(with gaps) = 18 Alignment score = 0.000000
TTCAAACGGCTTGCAGCG
>Fam_575_18_1 Nr. of seq. 1 Alignment length(with gaps) = 18 Alignment score = 0.000000
GTCGGCTCTGGCGTTACC
>Fam_576_18_1 Nr. of seq. 1 Alignment length(with gaps) = 18 Alignment score = 0.000000
ATCGGTAGCCCTGTGCAA
>Fam_577_18_1 Nr. of seq. 1 Alignment length(with gaps) = 18 Alignment score = 0.000000
TTTCAAGCTGAATGTGTG
>Fam_578_18_1 Nr. of seq. 1 Alignment length(with gaps) = 18 Alignment score = 0.000000
ACGGCACCGCCGGAAACA
>Fam_579_17_1 Nr. of seq. 1 Alignment length(with gaps) = 17 Alignment score = 0.000000
CAGGAGCAACAGGTCNA
>Fam_580_17_1 Nr. of seq. 1 Alignment length(with gaps) = 17 Alignment score = 0.000000
GGGCGGAGCGGTGCGAG
>Fam_581_17_1 Nr. of seq. 1 Alignment length(with gaps) = 17 Alignment score = 0.000000
TAGGATGTAAAAGTAAA
>Fam_582_17_1 Nr. of seq. 1 Alignment length(with gaps) = 17 Alignment score = 0.000000
TAAATATAAATAATGTA
>Fam_583_17_1 Nr. of seq. 1 Alignment length(with gaps) = 17 Alignment score = 0.000000
CAGGCCAGAAAGCACCA
>Fam_584_17_1 Nr. of seq. 1 Alignment length(with gaps) = 17 Alignment score = 0.000000
TTACTGATATGGGCTAA
>Fam_585_16_1 Nr. of seq. 1 Alignment length(with gaps) = 16 Alignment score = 0.000000
GCGAATGGGCGAATGA
>Fam_586_16_1 Nr. of seq. 1 Alignment length(with gaps) = 16 Alignment score = 0.000000
CCCGAGAGCCCGAGAG
>Fam_587_16_1 Nr. of seq. 1 Alignment length(with gaps) = 16 Alignment score = 0.000000

```
 TATATTGGTATATTGG
>Fam_588_16_1  Nr. of seq. 1 Alignment length(with gaps) = 16 Alignment score = 0.000000
 AAAGATATTTGTAGAA
>Fam_589_16_1  Nr. of seq. 1 Alignment length(with gaps) = 16 Alignment score = 0.000000
 GCCGGTGTTGNTAGTN
>Fam_590_16_1  Nr. of seq. 1 Alignment length(with gaps) = 16 Alignment score = 0.000000
 ATGTGATGATGTGATG
>Fam_591_15_1  Nr. of seq. 1 Alignment length(with gaps) = 15 Alignment score = 0.000000
 GCCAGCGCAGTCCCC
>Fam_592_15_1  Nr. of seq. 1 Alignment length(with gaps) = 15 Alignment score = 0.000000
 CTGCCGGATTCTGCG
>Fam_593_15_1  Nr. of seq. 1 Alignment length(with gaps) = 15 Alignment score = 0.000000
 ATGCCACTGCCCGGG
>Fam_594_15_1  Nr. of seq. 1 Alignment length(with gaps) = 15 Alignment score = 0.000000
 GGCATCTGGCCGCTG
>Fam_595_15_1  Nr. of seq. 1 Alignment length(with gaps) = 15 Alignment score = 0.000000
 CGTTTCGACCGGAGC
>Fam_596_15_1  Nr. of seq. 1 Alignment length(with gaps) = 15 Alignment score = 0.000000
 CGAGCCGGTCCCAGA
>Fam_597_15_1  Nr. of seq. 1 Alignment length(with gaps) = 15 Alignment score = 0.000000
 CCGTCGTCACCGTTC
>Fam_598_15_1  Nr. of seq. 1 Alignment length(with gaps) = 15 Alignment score = 0.000000
 GGTTGATCGACACAC
>Fam_599_15_1  Nr. of seq. 1 Alignment length(with gaps) = 15 Alignment score = 0.000000
 ACCTGCGCGGNGCGG
>Fam_600_15_1  Nr. of seq. 1 Alignment length(with gaps) = 15 Alignment score = 0.000000
 GCCCGATGCANGGTG
>Fam_601_15_1  Nr. of seq. 1 Alignment length(with gaps) = 15 Alignment score = 0.000000
 CTTAAATTTGCAAAN
>Fam_602_15_1  Nr. of seq. 1 Alignment length(with gaps) = 15 Alignment score = 0.000000
 CCCGACATTGCCCAA
>Fam_603_15_1  Nr. of seq. 1 Alignment length(with gaps) = 15 Alignment score = 0.000000
 CTGAATCGGACGGGA
>Fam_604_14_1  Nr. of seq. 1 Alignment length(with gaps) = 14 Alignment score = 0.000000
 CGCAGGCTGCAGAA
>Fam_605_14_1  Nr. of seq. 1 Alignment length(with gaps) = 14 Alignment score = 0.000000
 TATAATCTAATAAA
>Fam_606_13_1  Nr. of seq. 1 Alignment length(with gaps) = 13 Alignment score = 0.000000
```

CGTGCGCACCCGC
>Fam_607_12_1 Nr. of seq. 1 Alignment length(with gaps) = 12 Alignment score = 0.000000
ATTGAAAAAGGC
>Fam_608_12_1 Nr. of seq. 1 Alignment length(with gaps) = 12 Alignment score = 0.000000
CCTGTTTCACAA
>Fam_609_12_1 Nr. of seq. 1 Alignment length(with gaps) = 12 Alignment score = 0.000000
GAAGCGCCGCAA
>Fam_610_12_1 Nr. of seq. 1 Alignment length(with gaps) = 12 Alignment score = 0.000000
ATGGCGGCGGTA
>Fam_611_12_1 Nr. of seq. 1 Alignment length(with gaps) = 12 Alignment score = 0.000000
AGCTTGGTTGGA
>Fam_612_12_1 Nr. of seq. 1 Alignment length(with gaps) = 12 Alignment score = 0.000000
GCCGCTGAAGTT
>Fam_613_12_1 Nr. of seq. 1 Alignment length(with gaps) = 12 Alignment score = 0.000000
TCCTCTTTAGCC
>Fam_614_12_1 Nr. of seq. 1 Alignment length(with gaps) = 12 Alignment score = 0.000000
ATAATAACAATA
>Fam_615_12_1 Nr. of seq. 1 Alignment length(with gaps) = 12 Alignment score = 0.000000
ATTAATGAAGAT
>Fam_616_12_1 Nr. of seq. 1 Alignment length(with gaps) = 12 Alignment score = 0.000000
TAATGCTAATGC
>Fam_617_11_1 Nr. of seq. 1 Alignment length(with gaps) = 11 Alignment score = 0.000000
TCCCGGGCCGC
>Fam_618_11_1 Nr. of seq. 1 Alignment length(with gaps) = 11 Alignment score = 0.000000
CGATTGCCCGG
>Fam_619_10_1 Nr. of seq. 1 Alignment length(with gaps) = 10 Alignment score = 0.000000
GCTGATATTC
>Fam_620_10_1 Nr. of seq. 1 Alignment length(with gaps) = 10 Alignment score = 0.000000
CAACTATCAC
